# A systematic approach to estimate the distribution and total abundance of British mammals

**DOI:** 10.1371/journal.pone.0176339

**Published:** 2017-06-28

**Authors:** Simon Croft, Alienor L. M. Chauvenet, Graham C. Smith

**Affiliations:** National Wildlife Management Centre, Animal and Plant Health Agency, Sand Hutton, York, United Kingdom; Centre for Cellular and Molecular Biology, INDIA

## Abstract

Robust policy decisions regarding the protection and management of terrestrial mammals require knowledge of where species are and in what numbers. The last comprehensive review, presenting absolute estimates at a national scale, was published nearly 20 years ago and was largely based on expert opinion. We investigated and propose a systematic data driven approach combing publically available occurrence data with published density estimates to predict species distribution maps and derive total abundance figures for all terrestrial mammals inhabiting Britain. Our findings suggest that the methodology has potential; generally producing plausible predictions consistent with existing information. However, inconsistencies in the availability and recording of data impact the certainty of this output limiting its current application for policy. Restrictions on access and use of occurrence data at a local level produces “data deserts” for which models cannot compensate. This leads to gaps in spatial distribution of species and consequently underestimates abundance. For many species the limited number of geo-referenced densities hampered the extrapolation from habitat suitability to absolute abundance. Even for well-studied species, further density estimates are required. Many density estimates used were pre-1995 and therefore the derived abundance should not be considered a current estimate. To maximise a systematic approach in the future we make the following recommendations:

1. To mitigate the attitudes of a minority of local data providers occurrence records must be submitted to national surveys such as the Mammal Society’s Mammal Tracker.
2. Studies are required to estimate density for common species and in areas of low or no abundance.
3. To ensure such studies can be collated and used efficiently we propose a standardised approach reporting density estimates based on the 1km resolution British National Grid, or habitat representative of the 1km square, with digital maps to accompany publications.

## Introduction

Great Britain (GB) is home to many mammal species that are of conservation or management concern. Some need to be protected from threats such as diseases [1] or invasive species [2]. Others need to be managed to protect other native species from diseases, or prevent damage to the economy [3–5]. In order to inform such management decisions it is important to know where species are and in what abundance.

To date, the most widely used reference for species distribution and abundance in Great Britain is a report that was published by Harris et al. [6]. Whilst laudable, these results are now 20 years out of date. Moreover, they were obtained in large parts through expert solicitation, instead of a systematic and transparent process. Though often accepted practice throughout science this method of quantitative estimation can be subject to significant uncertainty and is particularly difficult to reproduce in a reliable and consistent manner. More recent studies have instead used data driven approaches to assess relative changes in abundance. However, due to the availability of suitable data this has been limited to a handful of species [7, 8]. As yet no studies have proposed a method by which absolute abundance estimates can be determined.

The emergence of large scale citizen science projects and data collation services, such as the National Biodiversity Network (NBN), has made this type of data driven process viable [9]. Previously, the identification and collation of data from such a wide range of sources would have been impossible particularly on a national scale. This coupled with the development and accessibility of species distribution modelling tools in computational platforms such as R [10] provides an avenue by which associations of observed occurrence with habitat can be quantified and related to known density estimates to provide an estimate of abundance. Species distribution models have become a popular tool in ecology allowing researchers to predict the spatial distribution of species where available data is lacking. There are many variations of model each applying a distinct set of assumptions. The merits of each model has been the subject of much conjecture in recent years [11, 12] and currently there is no definitive consensus as to the best single approach. Instead, a combined approach considering multiple models is often advocated to mitigate any undesirable effects which may be exhibited by individual models in particular scenarios [13, 14].

In this paper we outline a systematic modelling approach to predict species distributions and derive total abundance, using publically available data for 63 terrestrial mammal species across Great Britain. The purpose of this explorative study is to provide a transparent and reproducible method by which abundance can be assessed on a regular basis. The results of the study are used to discuss the feasibility of such an approach in the context of data availability and its potential use to inform policy decisions for species conservation and management: from directing conservation effort, through assessing disease risk and mitigation strategies, to addressing human-wildlife conflict. Additionally, we identify areas (occurrence records or density estimates) where improved data recording would most benefit any repeat of this approach and make recommendations for further work.

## Method

### Data

All maps produced as part of this publication, including supporting material, contain data (GB coastline) obtained from the OS Strategi® dataset 2016. This data is freely available under an open government license (@ Crown copyright 2016 100051110).

#### Occurrence data

Occurrence records were sourced via the NBN Gateway (https://data.nbn.org.uk/); the largest repository for UK biodiversity information collating datasets from a wide range of providers such as local records centres, national enthusiast groups, wildlife charities, government and environmental consultants. Whilst other similar repositories are available, for example the Global Biodiversity Information Facility (GBIF), the NBN was selected on the basis that it could potentially provide access to a greater volume of records and is updated more frequently (currently GBIF can only offers access to publicly available NBN records prior to 2013). Furthermore, records were spatially referenced using the British National Grid (BNG) and as a consequence inherently contain the information required to access recording accuracy. However, in order to use any of the datasets supplied by the NBN for scientific research purposes, even that which is freely accessible at a public level, written permission must be received from each data provider.

Across all 63 terrestrial mammal species, 62 whose abundance was estimated by Harris et al. [6] with the addition of wild boar (*Sus scrofa*), we identified and applied for access to 264 datasets from 87 providers containing 1.21 million records (non-sensitive records only) between 1960 and 2015 (this temporal period was necessary to ensure sufficient records were available for each species and reflects a similar period to that of Harris et al. [6] who also considered data post-1960). Of these, written permission was received to use 221 datasets from 65 providers, representing approximately 75% engagement, with a total 1.09 million records downloaded between 27/02/2015 and 30/06/2015. Datasets from two providers which granted permission after this date were not included in the analysis. In addition to the data downloaded via the NBN, three datasets were supplied directly through correspondence with providers. Overall, the reporting resolution of records obtained varied considerably with 38% given at 100m, 38% at 1km, 1% at 2km and 23% at 10km. A significant proportion of this variation was due to access restrictions imposed by providers who for a variety of reasons limited the resolution of available records. Further details of all datasets are provided in the supporting information accompanying this publication (S1 File: Section A).

Using the collected records point maps for all species were generated in ArcGIS v10.2. For quality control, each point was checked for geographical plausibility against the reported recording resolution. In order to achieve this buffered outlines of the land boundary for Great Britain, one for each resolution (100m, 1km, 2km and 10km), were used with any points falling outside of the corresponding boundary discarded. The remaining point locations were then used to generate various raster maps based on the BNG coordinate system describing: recording effort, the total number of records per cell; occurrence, whether a species had been recorded in a cell within the period of interest; and the decade of last sighting in cells where occurrence had been recorded. Initially, we assumed a raster cell size of 10km in which all records could be considered with equivalent uncertainty. However, at this resolution it would only be possible to describe the habitat of each cell at a broad scale and it was our concern that this may not fully capture sufficient variation to infer realistic associations. To examine the impact of cell size we also produced maps based on a 1km raster grid, closer to the home range of most species. Similar to the 10km case, to maintain equivalent uncertainty in recording accuracy, we only considered point locations recorded at a resolution of 1km or higher (i.e. 100m).

**Fig 1:**
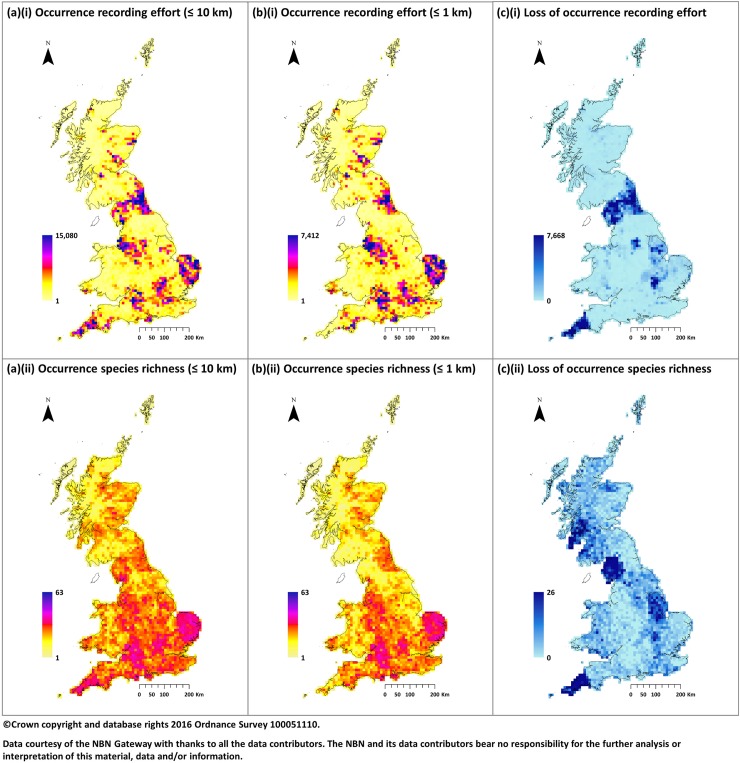
Summary of available occurrence records across all species. Maps displaying available occurrence records based on: (a) a 10 km raster grid considering all records; (b) a 1km raster using only records with resolution of 1km or higher (aggregated to 10km for comparison); combined across all species to give: (i) total recording effort; (ii) species richness. (c) maps the loss of information incurred by considering a higher precision model. This highlights the emergence of regional data “deserts” where the resolution of records has been limited by local providers.

Fig 1 presents the resulting raster maps for recording effort and occurrence across all species to give total recording effort and species richness (decade of last record is not shown as for both cases the majority of cells contain at least one recent record). The maps generated using the 1km raster have been aggregated to a 10km for direct comparison. Whilst generally there was a good agreement between the maps at each resolution it was noticeable that there were localised differences both in recording effort and richness. This was largely due to restrictions on the resolution of records imposed by providers creating regional data “deserts” at a 1km resolution. This was particularly evident in Cumbria and Cornwall where both the number of records and importantly the number of species recorded was significantly reduced. The implications of this with regards to the model prediction at the 1km scale are discussed later.

#### Environmental data

In order to perform Species Distribution Modelling, and map the suitability of habitat throughout Great Britain for terrestrial mammals, we downloaded spatial data on environmental factors from free online resources (hereafter spatial dataset). With the aim to design a systematic method, we chose layers that we believed *a priori* to be influential for the distribution of all terrestrial mammal species in Great Britain. Our spatial dataset thus included climatic variables in the form 19 temperature and precipitation related variables (average 1950–2000; worldclim.org, Hijmans et al. [15]), altitude (worldclim.org, Hijmans et al. [15]), human density (in 2011; UK census data, Office for National Statistics [16] and National Records of Scotland [17], and dominant target land cover class 2007 (CEH, Morton [18]). The spatial layers were processed to the resolution of the corresponding species occurrence data, both 1km and 10km, in ArcGIS v10.2. Full details of this process are included in the supporting information (S1 File: Section C). In this analysis we did not explicitly consider interactions between species, where presence data from associated species may inform the model, although this may be informative [19].

#### Density estimates

We searched the published literature for geo-referenced estimates of density for all species of interest. This was done by performing a systematic search through Web of Knowledge using the following search term: ‘density’ AND ‘Britain’ AND species name (common and scientific); the same search terms were also entered into Google Scholar. Only densities estimated for wild populations in Great Britain after 1960 were recorded and subsequently considered. Using the site description in each publication a representative polygon was estimated and mapped in ArcGIS v10.2 according to a standard extraction protocol (for details see S1 File: Section B). In a significant proportion of publications we found that the description provided insufficient detail to allow a site map to be estimated and in several cases even basic geo-referencing information was lacking. No publications were accompanied by electronic resources containing geographic representations of the study site or raw data. Furthermore, we also noted that it was often unclear how density estimates had been calculated and to what geographical extent they applied. In general there was also a lack of consideration for habitat specific variation within study sites with most reporting an overall estimate for the entire survey area. This may have important implications particularly for small mammals due to their relative size and movements.

**Fig 2:**
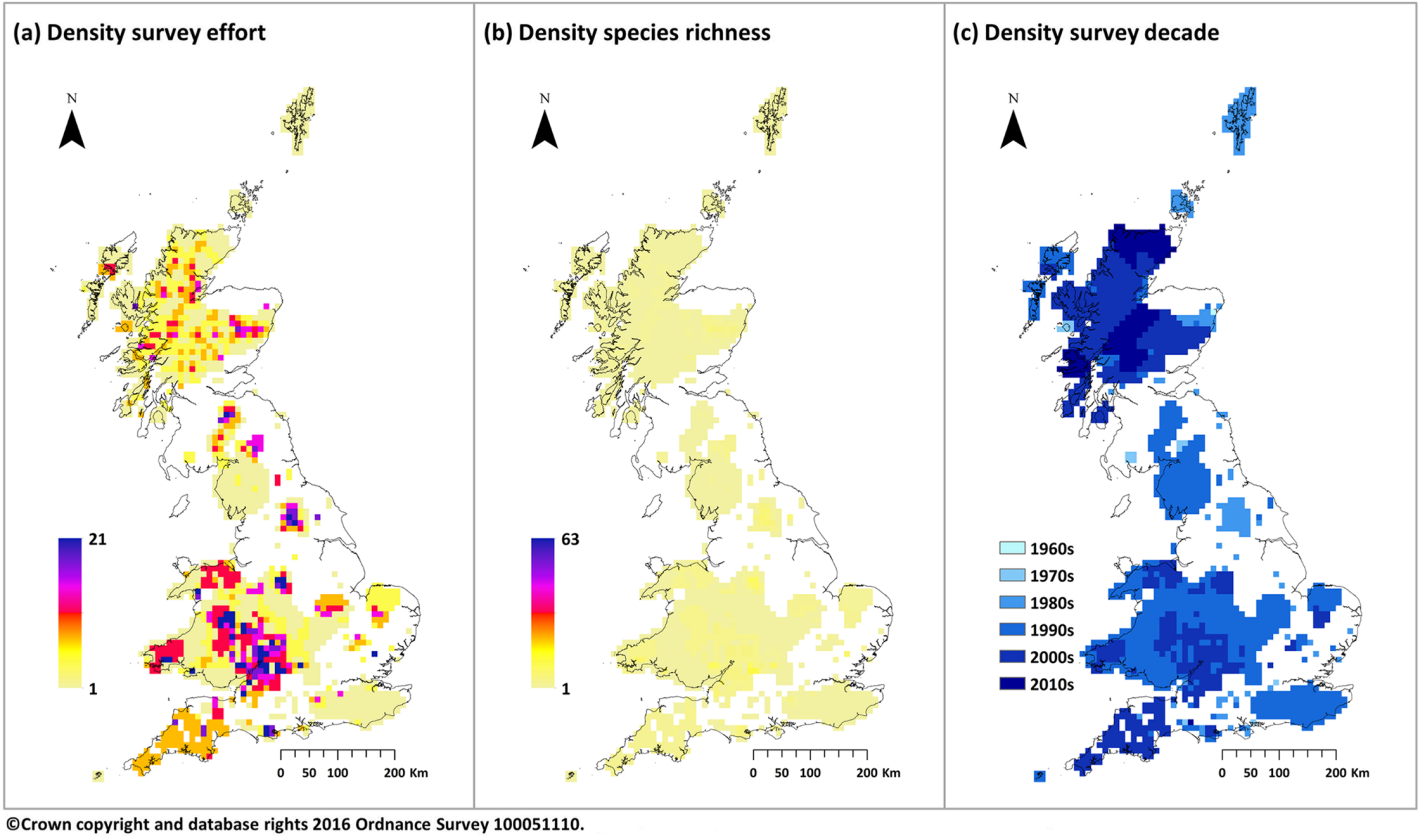
Summary of available density estimates across all species. Maps displaying extracted density estimates combined across all species to give: (a) total surveying effort (the number of density estimates in each square, with some species contributing more than a single estimate); (b) species richness (the number of species for which a usable density estimate exists); and (c) recording recentness (decade of last survey). Values derived from original polygon representations by converting to 25m raster grids and then aggregating cells to a display resolution of 10km.

In total we only identified density estimates in 95 publications spanning 53 species which could be geo-referenced and mapped. A full breakdown of these estimates by species is included in the supporting information (S2 File: Section B). In particular, this shows a substantial lack of recording for common species, such as rabbit (*Oryctolagus cuniculus*), and in areas of low or no density particularly for larger mammal species. Raster maps outlining the geographic location of these sites are present in Fig 2 detailing the surveying effort, species richness and recording recentness of studies within each cell. This highlights extensive gaps in surveying effort across Great Britain, predominantly in northern part of England.

### Modelling framework

**Fig 3:**
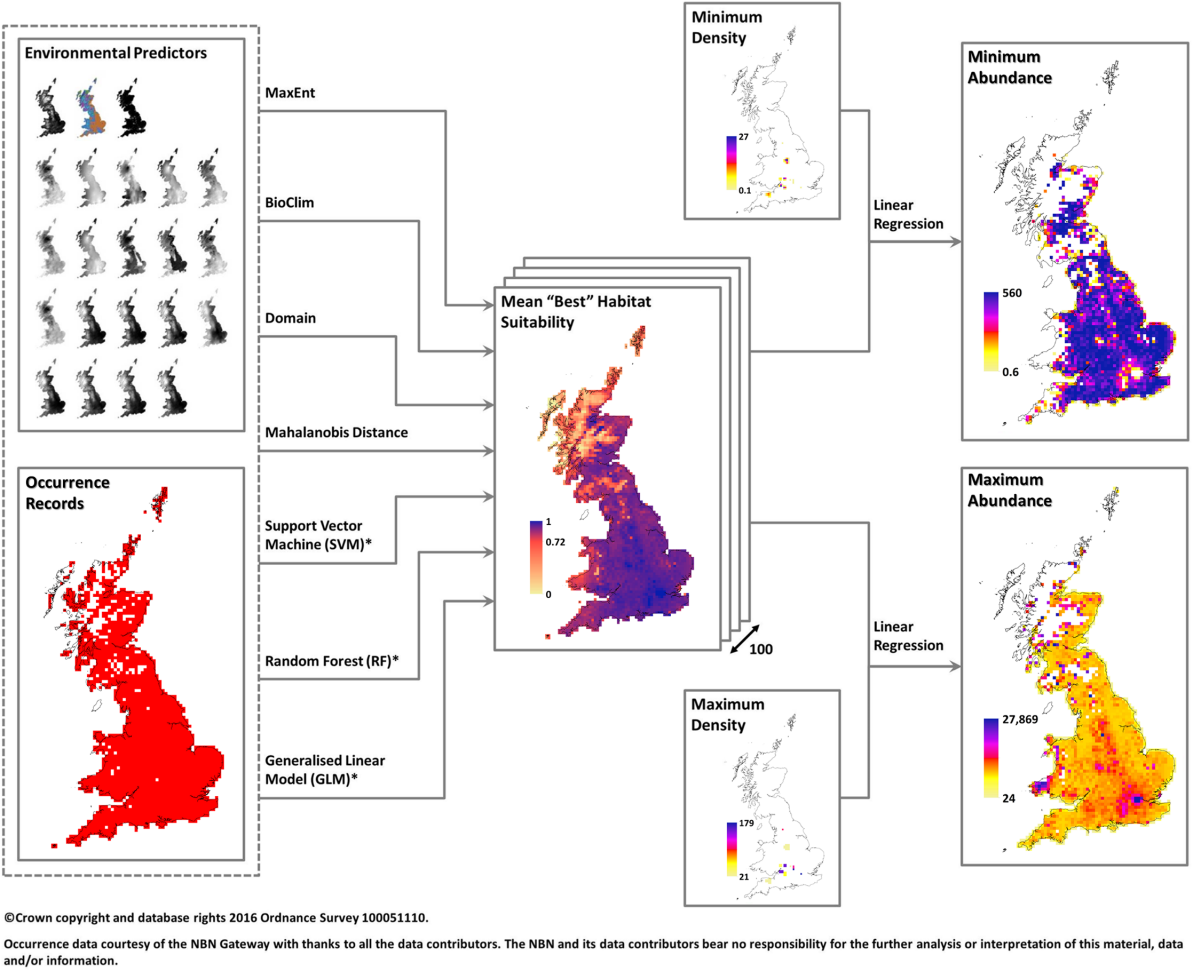
Diagram of the model framework. Outline of modelling process for each species (maps show output for the hedgehog: *Erinaceus europaeus*; for detailed discussion of these results refer to the species specific report in S6 File). Initially, occurrence is coupled with environmental data comparing 7 SDMs to identify the “best” habitat suitability map based on AUC. This is repeated 100 times with the best maps combined to produce an overall mean habitat suitability; the mid value indicates the threshold above which occurrence is assumed. For these cells, habitat suitability scores are then matched with extracted density estimates and linear regression performed to predict abundance.

The modelling approach consisted of a two stage process. Firstly, occurrence was coupled with environmental data using species distribution models to produce habitat suitability maps, representing the likelihood of observing occurrence, for each species. Then, these habitat suitability scores were matched with reported density estimates using linear regression to predict abundance. A graphical illustration of this process is shown in Fig 3. This method was applied to each species where sufficient data was available based on a 10km and 1km raster grid.

#### Species distribution modelling

Species distribution modelling (SDM) was performed using the R packages “dismo” [20], “randomForest” [21] and “kernlab” [22]. We implemented and compared seven habitat suitability algorithms using the Area Under the Curve (AUC). The AUC is a commonly-used method for comparing SDMs. It yields a value between 0 and 1, with 1 representing perfect model predictions, and a value ≤ 0.5 indicating a model that does not perform better than random.

The SDM methods compared here were both presence-(pseudo-)absence and presence-only algorithms: GLM (regression approach, pseudo-absence), Random Forest (Machine Learning method, pseudo-absence), Support Vector Machines (Machine Learning method, pseudo-absence), MaxEnt (Machine Learning method, used as presence-only with a default routine producing pseudo-absences as part of the method), Mahalanobis distance (profile method, presence-only), Domain (profile method, presence-only) and Bioclim (profile method, presence-only). Because our original species datasets were presence-only, we generated pseudo-absences for each species by selecting a fixed number of random cells from the GB outline and recording absence where there was no occurrence. The number of cells selected varied depending on the raster resolution to account for the difference in sample size. At 10km we selected 500 cells and at 1km we selected 5000, 10 times more which corresponded to the average increase in occurrence records across all species. Moreover, we divided each species presence-(pseudo-)absence datasets into training and testing sets by randomly setting aside 25% of the data for testing purposes (i.e. estimating the AUC). We adjusted for any spatial sorting bias in the test data, which has been shown to skew the evaluation of AUC, according to the method outlined in Hijmans [23]. All layers in the spatial dataset were initially used with each SDM. For GLM, however, we performed model selection using the “glmulti” function in the “glmulti” R package [24]; we then used only the best predictor variables for predictions using that SDM.

The comparison of all seven algorithms using the AUC yielded the best SDM given the selected testing data. This SDM was then used to map habitat suitability with occurrence predicted using the suitability threshold that maximised the sum of sensitivity and specificity [25]. This threshold has been shown to perform better than arbitrary thresholds by Liu et al. [25] whose findings demonstrated that it isn’t sensitive to the selection of pseudo-absence, it optimises discrimination between presence and absence and performed well for all algorithms tested. Due to the stochastic subsetting of the data for training and testing we found that the process did not necessarily produce the same result each time. To ensure a stable output we performed 100 repetitions combining the standardised output (habitat suitability and threshold scaled to values between 0 and 1) from each to produce the mean habitat suitability score in each cell and suitability threshold (whilst chosen arbitrarily testing showed that 100 repetitions was sufficient, producing a mean result with maximum relative standard error across all cells of <10%; typically a value of <25% is considered acceptable).

#### Species abundance

For each species where geo-referenced density estimates were available, we converted our mapped polygon layer to a raster grid based on a 25m cell size. Where polygons overlapped the most recent estimate was taken. We then aggregated these base maps to each of our chosen resolutions operating under two different assumptions designed to capture the potential uncertainty relating to partial coverage within cells. Firstly, we assumed that unsurveyed areas contributed a value of zero towards the mean density within each cell. This formed our lower or minimum density estimate. This would tend to occur where species are habitat specialists and all suitable habitat was surveyed in the study. Secondly, we assumed that unsurveyed cells were representative of those where estimates had been recorded with a value equal to the mean density of the surveyed cells. This formed our upper or maximum density estimate. This would tend to occur where species are generalists and have limited difference in density in the habitats within each grid square. We then identified the exact cells in which estimates were located, and extracted the corresponding suitability score from the map created using the best SDM. Any cells in which occurrence was not predicted based on our threshold value were discarded from this part of the analysis.

Linear regression was then used to model each species density as a function of suitability score. As a first step, we checked that the density data was normally distributed using a Shapiro test [26] from the core R package “stats”; this criteria is required to fit linear models. If the raw data was not considered normal (p value < 0.1) then an optimal power transformation was identified using the “box-cox” function from the R package “MASS” [27]. This was applied and the transformed data retested. If the newly transformed data was considered normal then we proceeded with the analysis. If however it was again rejected by the Shapiro test then we performed a further assessment on the original data by fitting a generalised linear model (“glm”) with various distributions (Gaussian; gamma; and Poisson) using the standard AIC statistic for comparison.

If the data could be considered normally distributed then we looked for spatial correlation in our dataset by running a simple model with different spatial autocorrelation structure (i.e. Gaussian, Exponential, and Spherical) and compare its AICc [28, 29] to a model without spatial autocorrelation. This is done by using a linear mixed model (“lme”) [30] with a dummy variable for random effect (i.e. all values set to 1). When autocorrelation was found, “lme” was used with the best autocorrelation structure; when it wasn’t we used the core “lm” function. Where the density estimates could not be considered normally distributed we used “glm” instead with the fitted distribution.

Once the correct modelling procedure was identified, we used the model to predict species density in cells without published estimates. If the data used for regression had been transformed to satisfy the normality criteria then the inverse was applied to this output. The resulting maps were finally converted from density to abundance by multiplying by the area of land mass contained within each cell; derived using a fine scale (25m) raster representation of the GB land boundary. The total abundance was calculated as the sum of the predicted abundance across all cells.

In some instances at the 1km level the density dataset was too large to perform the process outlined above. In such cases we generated random subsets containing 2000 density data points to perform analysis. This was repeated 10 times and the mean result computed (similar to the habitat suitability modelling, testing showed that 10 repetitions produced a mean with relative standard error <10%; it is likely that fewer repetitions were required here as the process contained fewer layers of stochastic sampling).

## Results and analysis

Due to the number of species considered in this study it is not be feasible to present all of the output here. A full breakdown of the available data (S1 File) and model results (S2 File) is provided in the supporting information. This includes an individual analysis and interpretation for each of the 63 species considered (S3 File). In this section we outline an example analysis of the available data and model predictions, based on a 10km raster grid, for a nominal species (red fox: *Vulpes vulpes*) and discuss the model performance. Top level metrics are then presented for all species.

**Fig 4:**
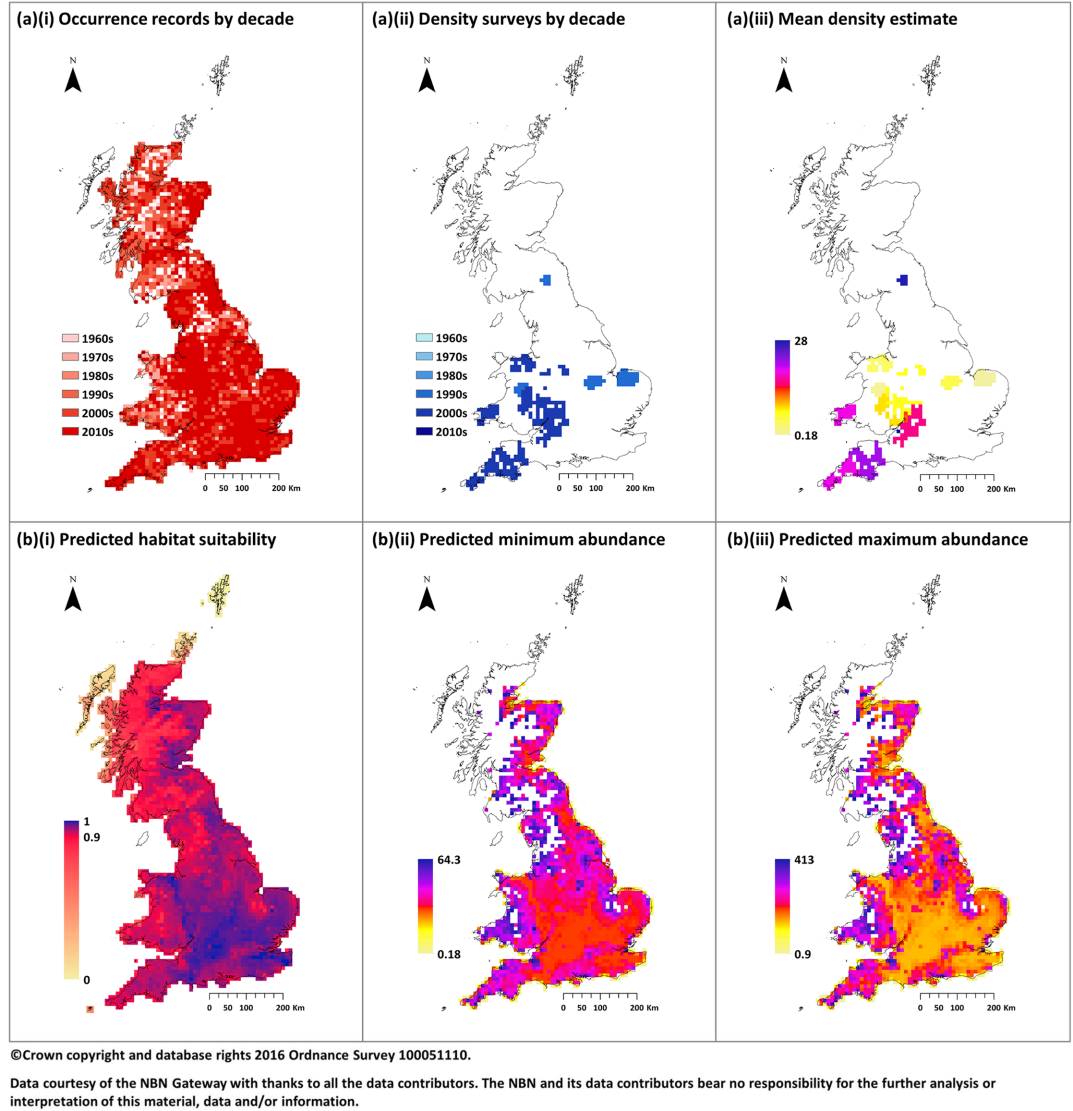
Example maps for the red fox. Plot (a) presents the available data showing: (i) species occurrence; and (ii) density; by the decade of last sighting; (iii) mean density (estimates are assumed to be representative of entire cell, considered the upper limit of observed density). Plot (b) presents the corresponding model predictions based on a 10 km grid showing: (i) habitat suitability; (ii) the lower bound (Minimum); and (iii) the upper bound (Maximum); for abundance. Maps show the fox is a highly reported and surveyed species. Habitat suitability modelling reflects this predicting widespread occurrence across a variety of landscapes. Maps of abundance show similar spatial distributions suggesting a negative correlation between habitat suitability and density. Uncoloured areas indicate absence (i.e. zero abundance) which is assumed where habitat suitability scores are lower than a threshold value; in this case 0.9.

The maps shown in Fig 4 present example analysis for the red fox. Fig 4(A)(i) shows that this species is highly recorded throughout GB (sightings cover approximately 84% of the area) with most cells containing at least one sighting from the last decade (median year of reporting is 2011). Fig 4(A)(ii)-(iii) also show that the species is well surveyed; red fox is amongst the most surveyed in terms of the number of estimates identified here with a total of 26 (S1 File: Table C). This is perhaps unsurprising given recent meta-analysis of scientific publications which showed that the red fox has the fourth highest H-index of all recognised terrestrial mammals in GB [31]. A total of 4 publications were identified during the literature search providing 26 geo-referenced estimates of density (12 unique sites). Compared with many other species these estimates spanned a large proportion of the occurrence extent (approximately 10%) and geographically provided a reasonable sample of locations across the distribution. However, when stratified by dominant land cover, which we argue is an important factor in species distribution, estimates were not available for several classes in which occurrence had been recorded (Table 1). It is also important to note that the temporal range of the estimates was more limited (1995–2006). The implications of this in terms of interpreting model output is that whilst the predicted extent of the species represents an average distribution from 1960 to 2015 (for the red fox this is likely to have been relatively stable) predictions of abundance only reflect an average across a period equivalent to that of the available density estimates. Consequently, the reader should note that all the abundance figures presented in this publication are not considered current estimates but may be regarded as an approximate estimate for the mean year of the studies used to estimate density.

**Table 1:** Summary of observed data and model predictions by land cover for the red fox.

	Observed					Predicted		
	Occurrence		Density					
LCM2007 class	Records	Year	Estimates	Year	Range	Habitat suitability	Density	Abundance
1 (Broadleaved woodland)	717 (11)	2013	0 (0)	-	-	0.95 (11)	0.4–2.2	436–2,387
2 (Coniferous woodland)	1,213 (144)	2006	9 (10)	1998	0.9–3.8	0.9 (75)	0.4–2.2	2,969–16,389
3 (Arable and Horticultural)	25,478 (934)	2013	157 (151)	2006	0.4–2.3	0.95 (938)	0.4–1.9	33,024–177,346
4 (Improved grassland)	15,559 (687)	2012	125 (118)	2006	0.5–2.5	0.9 (619)	0.4–2.2	24,059–134,884
5 (Rough grassland)	141 (21)	2003	0 (0)	-	-	0.42 (0)	-	-
6 (Neutral grassland)	0 (0)	-	0 (0)	-	-	0 (0)	-	-
7 (Calcareous grassland)	66 (2)	2008	0 (0)	-	-	0.97 (2)	0.3–1.5	63–303
8 (Acid grassland)	1,129 (170)	2002	12 (11)	2006	0.1–1.5	0.86 (69)	0.5–3.1	3,507–21,176
9 (Fen, Marsh, and Swamp)	0 (0)	-	0 (0)	-	-	-	-	-
10 (Heather)	188 (45)	2007	0 (0)	-	-	0.84 (22)	0.5–3.3	1,185–7,344
11 (Heather grassland)	760 (83)	2006	0 (0)	-	-	0.66 (15)	0.6–3.5	836–5,195
12 (Bog)	331 (72)	2003	2 (2)	2006	0.1–2.5	0.56 (9)	0.5–2.8	429–2,536
13 (Montane habitat)	140 (39)	1998	0 (0)	-	-	0.84 (2)	0.6–3.9	123–783
14 (Inland rock)	4 (1)	2002	0 (0)	-	-	0.65 (0)	-	-
15 (Saltwater)	75 (8)	2011	0 (0)	-	-	0.84 (1)	0.3–1.8	28–183
16 (Freshwater)	4 (2)	1994	0 (0)	-	-	0.69 (0)	-	-
17 (Supra-littoral rock)	0 (0)	-	0 (0)	-	-	0.08 (0)	-	-
18 (Supra-littoral sediment)	30 (3)	2011	1 (1)	2006	2.7–3.6	0.64 (0)	-	-
19 (Littoral rock)	2 (1)	2006	0 (0)	-	-	0.43 (0)	-	-
20 (Littoral sediment)	453 (27)	2012	2 (2)	2006	0.1–1.0	0.84 (4)	0.5–2.8	183–1,122
21 (Saltmarsh)	0 (0)	-	0 (0)	-	-	-	-	-
22 (Urban)	830 (8)	2014	0 (0)	-	-	0.93 (7)	0.3–1.7	213–1,198
23 (Suburban)	9,431 (76)	2014	2 (2)	2004	0.8–16.0	0.93 (67)	0.4–2.2	2,570–14,863
**Total**	**56,551 (2,334)**	**2011**	**310 (297)**	**2006**	**0.4–2.5**	**0.86 (1,841)**	**0.4–2.1**	**69,626–385,710**

Values shown in brackets denote the spatial coverage based on a 10km resolution raster map (number of grid cells). Years represent the median of records within each land class. Ranges for density and abundance are derived using the respective minimum and maximum raster maps (lower bound is mean of values across minimum raster map with upper across the maximum) which capture the spatial uncertainty generate by projecting irregular polygons describing survey sites onto a raster grid.

Encouragingly, the habitat suitability map shown in Fig 4(B)(i) appears to reflect the underlying data well, predicting a similar distribution of occurrence (although the default threshold for presence results in a contraction in Scotland and Cumbria where the species is known to be present–compare Fig 4(B)(ii) with Fig 4(A)(i)). The map also suggests, shown by the lack of variability in suitability score across most of the range, that the red fox is a generalist species which is consistent with ecological evidence. This is reflected in the analysis of the mean habitat suitability scores by land cover which highlights many different classes where observation is highly likely including woodland, arable farmland and urban landscapes (Table 1). In addition, the breakdown also confirms that, consistent with recorded sightings, the highest number of foxes is in arable and improved grassland; although this is perhaps understandable for such a ubiquitous species given these are the most common dominant land covers at a 10km scale. Even for such a well reported species, it can be noted that many of the occurrence records in areas of low human population density are a few decades old. This implies that, assuming a species is actually present, we require more occurrence records in areas of low human density, and further records here may adjust the threshold and thus the population distribution. In terms of the total population size, the areas below the threshold will be at a lower density and thus for many species would not substantially increase the predicted population size.

In this case, linear regression suggested both minimum and maximum density estimates were best fitted to models using a second order polynomial of habitat suitability accounting for spherical spatial autocorrelation. Interestingly, these models predicted a negative correlation in which squares with a higher likelihood of observation contain a lower density of individuals. Whilst this may seem counterintuitive the prediction matches other published estimates confirming the observation [32]. The areas which our model places below the threshold for presence are almost identical to the areas predicted to have the lowest density by Webbon et al. [32]. The resulting abundance range does contain the estimate from Harris et al. [6] with a mid-point of approximately 230,000. This figure, slightly lower than the Harris et al. [6] estimate, is in agreement with the most recent trend analysis [6], indicating that the population may have decreased slightly since 1995 (up until 2006 which is median of the published estimates). The full results for each species and a brief comment are available in the supplementary material. Since this is an investigation of the methods, detailed analysis of each individual species is not the objective of this paper.

Table 2 presents summary statistics relating to the analysis described above for 36 of the most surveyed species, i.e. those for which the full modelling process could be applied. This shows that a majority (61%) of predicted abundance ranges contain the 1995 estimate. However, for most species, the range is very large. This is a reflection of the uncertainty produced by a mismatch between the scales of occurrence and density reporting. It is particularly noticeable for riverine species such as American mink (*Neovison vison*) and water voles (*Arvicola terrestris*) whose density is typically reported using linear features which are not well represented by environmental descriptors on a two dimensional grid with our chosen resolutions. Perhaps unsurprisingly when modelling was performed based on a finer scale grid abundance ranges were substantially smaller offering a more accurate prediction of abundance; the size of range was on average 50% less for predictions based on a 1km raster grid (S2 File: Table A). This demonstrates the need for modelling on finer, ecologically relevant, scales but issues relating to data access prevented this here.

**Table 2:** Summary of model predictions and analysis of observed data for the 36 “most” surveyed species.

Common name (grouped by species order)	1995 abundance (reliability score)	Predicted abundance range (10km model analysis)	Recording rate (% 1995 est. yr^-1^)	Published densities (sites; median yr)	LCM2007 dominant target land classes requiring density estimates (* denotes 10km only & ** 1km only)
Hedgehog	1,555,000 (4)	731,546–11,979,363	0.091 (-)	21–179 (10;2002)	1–2*,5*,6,7*,9**,10,11*,12–13,14*,15,16*,18–20,21**,22*
Mole	31,000,000 (3)	126,065–109,231,019	0.003 (-)	420–850 (7;1987)	1,2*,4*,5,6**,7–8,9**,10–13,14**,15–16,17**,18,20,21**,22–23
Common shrew	41,700,000 (3)	1,838,490–223,651,088	0.001 (-)	0–9718 (38;2003)	2*,5*,6**,7,9**,10,12*,13–16,17**,18,20*,21**,22
Pygmy shrew	8,600,000 (4)	11,566–36,213,441	0.001 (-)	0–2852 (8;1996)	2,5,6**,7–8,9**,10–16,18,19**,20,21**,22–23
Water shrew	1,900,000 (4)	22,166–406,189	0.004 (-)	2.9–3.1 (2;1995)	1,2,4*,5,7–8,9**,10,13,14**,15–16,18,19**,20,21**,22–23
Natterer’s bat	100,000 (4)	76,593–1,709,679	0.248 (+)	1.8–24 (2;1990)	1*,2*,5,6**,7–8,9**,10–11*,12–13,15–16,18**,20,21**,22,23*
Daubenton’s bat	150,000 (4)	39,292–245,200	0.238 (+)	1–2.4 (2;1990)	1*,5*,6–7**,8*,9**,12,13*,14**,15–16,18,20,21**,22–23*
Serotine	15,000 (4)	5,419–20,733	0.574 (+)	0.16–0.59 (4;1991)	1,2,4*,5,6**,7–8,9–11**,12*,15,16**,18**,20,21**,22,23*
Leisler’s bat	10,000 (4)	30,319–356,068	0.095 (-)	4.4–6.7 (2;1994)	1*,2,5**,8*,9**,10*,11,12*,16**,20*,22
Pipistrelle	2,000,000 (3)	454,098–1,849,199	0.027 (-)	1.6–18.2 (3;1987)	1*,5*,6**,7,8*,9**,12–13*,14–15,16*,18–20,21**,22*
Brown long-eared bat	200,000 (4)	133,497–374,147	0.244 (+)	1.4–14.9 (3;1990)	1*,5*,7,8*,9**,12–13*,14**,15–16,18**,20,21**,22–23*
Rabbit	37,500,000 (3)	2,069,527–255,508,540	0.005 (-)	19.8–5000 (4;1989)	1*,5–7,8–9**,10–14,16,17**,18*,19,20*,22–23
Brown hare	817,500 (2)	118,829–3,393,442	0.122 (+)	0–77.3 (62;2006)	1*,5*,6**,9**,10–11*,13,16*,22*
Mountain hare	350,000 (3)	2,633–1,186,763	0.023 (-)	3.7–89 (7;1971)	1**,3–4,5*,8*,12,14,15*,16,23
Red squirrel	160,000 (3)	305,073–11,237,141	1.216 (+)	3.2–422 (14;1996)	1*,5–7**,8*,9**,10–12*,13,14**,16,19–21**,22,23*
Grey squirrel	2,520,000 (3)	1,545,851–14,511,831	0.108 (-)	8–169 (7;1997)	1*,6**,7,8*,9**,10–12*,13,14**,15–16,18**,19–20,21**,22,23*
Bank vole	23,000,000 (3)	189,622–204,426,956	0.001 (-)	0–15309 (38;1995)	1*,6**,7–8,9**,10–13,14**,15,18,19**,20,22
Field vole	75,000,000 (4)	4,875,844–463,671,721	<0.001 (-)	1.4–30923 (15;1997)	1*,5*,6**,7,8*,9**,10–12*,13–15,16*,17**,18–20,22,23*
Water vole	1,169,000 (3)	544,441–7,995,892,846	0.075 (-)	0–293450 (18;1999)	1*,3**,5*,6**,7,8*,9**,10*,15,18,20,22*,23**
Wood mouse	38,000,000 (3)	809,118–218,026,188	0.001 (-)	0–11975 (38;1995)	1*,6–8,9**,10–16,17**,18,19**,20,22
Yellow-necked mouse	750,000 (4)	55,873–592,033	0.003 (-)	0–88.7 (6;1996)	1*,2,5**,8,11**,16**,22*,23
Harvest mouse	1,425,000 (5)	12,941–279,861	0.005 (-)	0–3.49 (5;1996)	1–2,5,6**,7,9–11**,12,14**,15,16**,18,20,21**,22–23
House mouse	5,192,000 (5)	1,449–500,536	0.001 (-)	0–3750 (5;1996)	1–2,4**,7–8,9**,10–12,13*,15,18**,19*,20,21–22**,23
Common rat	6,790,000 (4)	1,724,587–31,562,005	0.007 (-)	6.6–238 (2;2003)	1*,2,3*,5,6**,7–8,9**,10–13,14**,15–16,17**,18–20,22
Red fox	240,000 (4)	69,625–385,710	0.429 (+)	0.14–27.6 (12;2006)	1*,5*,6**,7,9**,10–11*,12,14–16*,17**,19*,21**,22*
Pine marten	3650 (2)	2,019–25,177	1.499 (+)	0.12–0.82 (12;1998)	1*,5*,9**,12*16*18**,20,22**,23*
Weasel	450,000 (4)	1,056,431–24,999,091	0.054 (-)	13–275 (3;2000)	1*,4*,5,6**,7,8*,9**,10–12*,13,14**,15,16*,18,19,20*,21**,22–23
Polecat	15,000 (3)	52,011–53,475	0.582 (+)	0–1.86 (10;1996)	7*,22*
American mink	110,000 (3)	13,714–2,724,393	0.223 (+)	1.6–70 (10;2003)	1–2*,7,8*,10*,13,14–15*,20*,21**,22,23*
Badger	250,000 (1)	79,544–968,740	0.327 (+)	1.2–43 (20;2006)	1*,5*,6**,7,9**,10–11*,13,15–16*,17**,19*,21**,22*
Wildcat	3,500 (3)	1,740–16,255	0.436 (+)	0.3–0.68 (2;1978)	3–5*,7**,8*,12*,15**,16,19–20**,22**,23
Red deer	360,000 (2)	379,297–780,812	0.065 (-)	0.1–29.8 (40;2009)	1*,6**,7*,15*,20*,22–23*
Sika deer	11,500 (2)	21,477–357,800	0.312 (+)	0–25.6 (44;1997)	1*,5*,7*,12–13*,15*,18**,23*
Fallow deer	100,000 (4)	303,314–4,635,453	0.154 (+)	38–46 (3;1994)	1–2*,5,6–7**,8,9**,10–11,12–13*,14–15**,16,18**,20,21**,22,23*
Roe deer	500,000 (3)	563,932–4,500,284	0.154 (+)	16–76 (5;2002)	6**,7,8*,9**,10–11*,12–16,17**,18–20,21**,22,23*
Chinese muntjac	40,000 (3)	1,962,152–5,046,501	0.757 (+)	20–120 (3;2002)	1*,4*6**,7–8,9**,10–12,14**,15*,16**,18**,20*,21**,22,23*

Omission from this list indicates too few geo-referenced density estimates (≤1) could be identified from the literature to perform a full model analysis.

Of the ranges which do not match the 1995 estimate there appears to be a tendency to overestimate (64%; 75% taking into account all modelled species). This may be due to an expansion of some populations (which could explain the increases predicted for the Artiodactyla), the lack of ability to account for spatial contraction (e.g. the red squirrel: *Sciurus vulgaris*) and a bias within density reporting towards higher estimates, are likely the main causes. Statistics relating to the available data are also shown (Table 2). The aim of this work is to highlight any deficiencies in reporting so that efforts can be targeted where they are most needed. For example, almost all species lack density estimates in a majority of recognised land classes.

The recording rate describes the mean annual frequency that each species is recorded on the occurrence database, for its estimated (1995) population size (Table 2 and S1 File: Table B). This shows clear differences across taxa with the most recorded species per capita, either weighing approximately 10kg or are bats. Small mammals are typically the least recorded despite large populations; likely reflect the relative effort required to identify the species. In particular, this analysis also suggests that there is a recording bias favouring “rare” or “at risk” species (e.g. horseshoe bats: *Rhinolophus ferrumequinum* & *R*. *hipposideros*, pine marten: *Martes martes*, red squirrel). In contrast rates for smaller common species tend to be low (in particular rabbits and moles: *Talpa europaea*) (S1 File: Table B). These same trends are not necessarily reflected in density reporting. However, it is notable that the number of estimates for some common species such as rabbit and brown rats (*Rattus norvegicus*) remain low (Table 2 and S1 File: Table C). There also appears a tendency in a number of cases, particularly for rarer species, to focus on high density populations; this is most likely driven by interest lead research to address a specific ecological question where guaranteed observation is necessary (particularly apparent for hedgehog, mole and some deer species).

Assessing the results of the habitat suitability modelling across all species we found that at both resolutions (1km and 10km) MaxEnt was the optimal individual modelling approach selected as the best predictor for 66% and 45% of species based on the 10km and 1km raster grids respectively. However, based on the mean AUC across all species it does not perform statistically better than Random Forest, or at the 1km resolution SVM. The strictly presence-only profile methods (Bioclim, Domain and Mahalanobis distance) were the worst performers; never selected as the best predictor at the 10km resolution and only for 3% of species at 1km. There were no identifiable patterns at either resolution between species, status or origin and optimal modelling approach; only 43% of methods were retained over both resolutions. Overall, the mean AUC of the best habitat suitability models was 0.73 and 0.68 for 10km and 1km respectively suggesting a good agreement between observed and predicted data. The reduction in AUC for models at the higher resolution is likely due to the increased variability of environmental factors.

**Fig 5:**
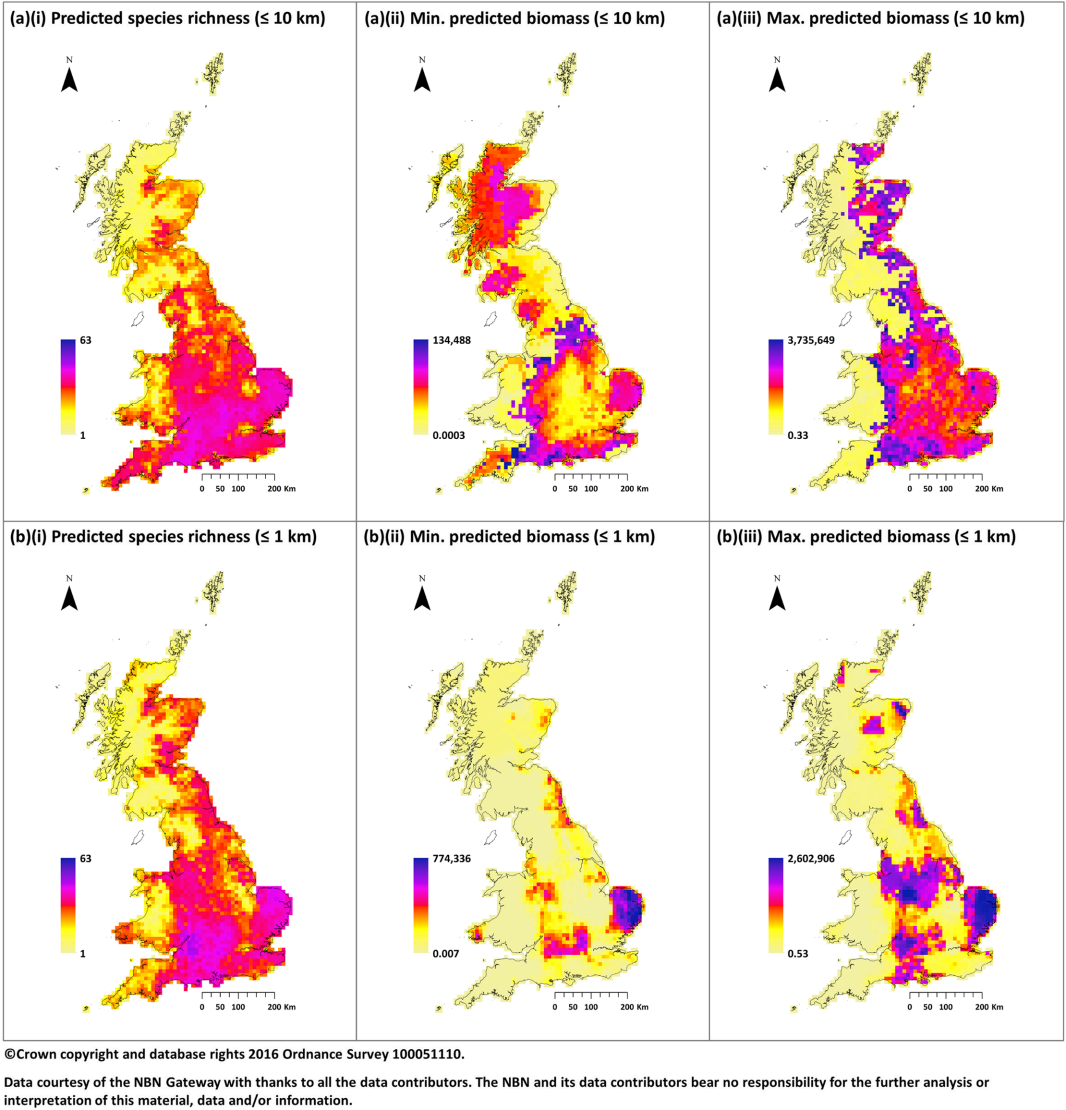
Summary of model predictions across all species. Maps displaying model outputs based on: (a) a 10 km raster grid considering all records; (b) a 1km raster using only records with resolution of 1km or higher (aggregated to 10km for comparison); combined across all species to give: (i) predicted species richness; (ii) Minimum predicted biomass; (iii) Maximum predicted biomass. There is good agreement between predicted richness and that observed in Fig 1. However, there is a degree of inconsistency, particularly based on a 10km raster, between the spatial distributions of minimum and maximum biomass.

Spatially, the maps shown in Fig 5(A)(i) and 5(B)(i) present the species richness based on predicted occurrence for each species. As indicated by the AUC these demonstrate a good comparison with that of the observed data presented in Fig 1. However, whilst this suggests that models can successfully reproduce observed data the extent to which they can compensate for gaps in data availability at a regional scale is clearly limited. Despite a mean increase in coverage of approximately 136 time (136km^2^ per observation) at 1km, compared to 1.8 times (180km^2^ per observation) at 10km, Fig 5(B)(i) highlights this with poor species richness predicted in regions where the resolution of records was restricted. This factor means that whilst models based on a finer spatial scale produce more accurate estimates the relative magnitude will likely be artificially reduced due to gaps in predicted distributions confounding the results and limiting their usefulness.

To visualise the predictions of abundance across all species we computed the total biomass in each cell by multiplying the abundance for each species by the standard weight outlined in Harris et al. [6] and summing the result (shown in Fig 5: plots marked (ii)-(iii)). For models based on 10km we observe a degree of inconsistency in the predicted maps for minimum and maximum biomass, largely driven by an overestimation in the minimum abundance of Artiodactyla species. Whereas when using a finer resolution there is better agreement; again, making a case for modelling based on a finer scale grid. However, this agreement may be due to a reduced dependence on habitat suitability with most predictions based on a constant application of density in all cells where occurrence is predicted; probably driven by a lack of variation in reported density within study sites over a wide geographic area and should therefore be treated with caution.

## Discussion and conclusions

In order to develop informed policy decisions regarding the management of wild mammals in Great Britain it is important to establish the location and abundance of species. In this paper we proposed a systematic and reproducible method by which this information could be determined. Our findings suggest that at present data driven modelling approaches of this type are unable to compensate for serious deficiencies in data availability within specific regions and for certain species both of occurrence and density estimation. This increases the uncertainty surrounding our predictions of distribution and total abundance and thus decreases their usefulness for making decisions. Nonetheless, in the majority of cases a plausible population estimate is generated with 61% of estimates including the 1995 estimate. Additionally, all deer estimates are greater than the 1995 estimates and all are known to be expanding in range, with a prediction that roe deer (*Capreolus capreolus*) would occupy 79% of all 10km squares by 2015 [33]. From this work we predict that roe occupy 74% of all 10km squares (S2 File: Table A).

At a national scale it is often impractical to gather data directly. Instead, projects such as this rely upon the provision of data from several different sources. We have argued that the NBN Gateway provides a valuable resource for this type of modelling where suitable data can be identified and collated into a standard format for processing. However, the currently data use requires written permission from each provider and our experiences have highlighted that the response of providers to data requests is varied and in some cases there is a lack of engagement with this process. The NBN recognises that this process is a major barrier in the use of biological records in research and are launching their new NBN Atlas platform (for England and Wales; the Atlas for Scotland is already live and can be accessed at http://www.als.scot/) on the 1^st^ April 2017 which will require all datasets to be assigned a standard Open Government License (OGLs), thereby reducing the need to contact providers for permission [34]. Whilst this change in policy will improve the situation with regard to access and usage it is unclear whether there will be an impact on the volume or quality (resolution) of available data compared with that currently held on the Gateway.

Local Environmental Record Centres (LERCS) form a significant proportion of the potential data providers and are a key source of records. ALERC, the accreditation body for these organisations (setup in collaboration with Natural England), encourages members to share data at a national level primarily through the NBN [35]. Nevertheless, our understanding is that there are no recommendations for the standard of data provision and as a result both the quality and quantity of data provided to the NBN is variable. Through communications with providers there is also an acknowledgment from many that records on the NBN are either outdated or incomplete and to obtain better data applications should be submitted directly. This compromises the utility of the NBN and as a result the viability of national projects which would benefit from a unified data source.

In addition to the issues affecting the availability of occurrence records there we also highlight deficiencies in the collection and reporting of density estimates. In particular, Fig 2 shows that there are substantial geographic areas where no density surveys have been conducted and across all species only 5% of the area covered by predicted species distributions contained a viable estimate. To compound this issue, many of the publications which we identified through our systematic literature search contained insufficient information to accurately map study sites and none were accompanied by electronic resources outlining the survey boundaries or raw data which would allow for reanalysis or interpretation at a later date. Consequently, it was often unclear as to how density estimates had been calculated and to what area or extent they applied. This was particularly evident for larger mammal species which are typically recorded over a broad geographic range comprising a varied habitat but with only a single estimate reported. It is likely that this lack of spatial variation is a significant factor contributing to the low levels of correlation between habitat suitability and density of model predictions based on a 1km raster. Amongst the estimates which could be mapped we note that there was a surprising lack of reporting on common species, such as rabbit, with a bias towards specialist less well known species which could perhaps be considered academically more interesting. Similarly, there was also a lack of low density estimates with studies tending to concentrate on high density populations where observation is easier and more rewarding in terms of scientific impact. However, for modelling purposes at a national scale such estimates are required to balance predictions and prevent overestimation of populations.

Despite these constraints on data quantity and quality the modelling approach outlined demonstrates an ability to accurately reproduce occurrence and through association with predicted habitat suitability scores infer total abundance comparable to that of Harris et al. [6]. It is clear however that there are limitations to the extent that the proposed modelling approach can account for deficiencies in the data, particularly regional gaps in occurrence records at the 1km level. There is also evidence to suggest for a few species that the lack of a representative set of density estimates may lead to overestimation of population sizes. Naturally, there are improvements which could be made to the modelling process. Some of which are dependent on data availability, for example, using a smaller temporal scale to determine more recent trends or using a higher resolution to bridge the gap between scales of density and occurrence recording. A reduced temporal data set should improve the estimates for species declining in range (e.g. red squirrel, which was over-estimated and hedgehog which is becoming more heterogeneous in range [36]). However, others could be implemented irrespective of improvements in data, for example consideration for percentage land class rather than solely relying on dominant classifications to better describe habitat types for specialists such as American mink or basing grid cell sizes on reported home range. There is also scope to increase the pool of species distribution models to consider more recent developments [37]. It may also be possible to explore rationalising the difference between density survey techniques and occurrence recording by instead computing the mean habitat suitability score for each density polygon, considering all cell values regardless of whether the score lie below the computed occurrence threshold as has been applied in this publication, and relating this to recorded density estimates (although this may simply average any variation in habitat suitability masking any clear dependence with density). If this is not successful then the development of species distribution models which allow for an uncertainty weighting, distinguishing between records of various resolutions, may provide a new avenue by which higher resolution grids could be implemented without the need to discard unsuitable records.

By utilising only presence data this approach could be used more widely across Europe to predict the range of many mammalian species, even where data may be sparse (e.g. Savi’s pipistrelle: *Hypsugo savii* [38]) and where estimates of abundance exist [39], absolute population size.

However, the issues relating to data remain the most significant constraint on this process. In order to maximise the predictive power and therefore the impact of a systematic approach in the future we propose several recommendations aimed at improving data collation and collection.

1. To mitigate the attitudes of a minority of local data providers **occurrence records must be submitted directly to national surveys** such as the Mammal Society’s Mammal Tracker (this should not prohibit the submission of duplicate records to local organisations). This would significantly reduce the overhead time associated with negotiating access to data and facilitate a more standardised level of access to data at an appropriate resolution corresponding to habitat variability and species movement.
2. **We encourage all data holders** (Record Centres and academics) **to submit full species presence data to the NBN.** We are aware that many additional records exist within the organisations that are already listed on the NBN and that many people hold additional records collected during surveys that could help contribute to the national database.
3. **More focus is required to provide density estimates for common species, such as rabbits, and for species in areas of low or no abundance**. We encourage the publication of small-scale local studies of species density, although we admit that with current trends these may be hard to publish.
4. To ensure that such studies can be collated and used efficiently, where possible, we suggest a standardised approach reporting **density estimates based on the 1km resolution British National Grid, or areas representative of that 1km square, with digital maps to accompany publications**. This would directly complement the existing method of recording for occurrence and would reduce the uncertainty produced by converting irregular study sites to a standard raster grid for processing. For most species this cell size would adequately capture any associations with the dominant land class and therefore we anticipate would produce a better correlation with habitat suitability.

## Supporting information

S1 File Data descriptions (.pdf). A full description of the data identified and used in the modelling process including: a breakdown of the datasets available via the NBN, the permission received and a full list of acknowledgments; a breakdown of occurrence records by species; a breakdown of density estimates by species and a full list of references from which estimates were extract; full details of the environment data used for the habitat suitability modelling and a methodology for standardising this information to correspond with this process.
(PDF)

S2 File Additional model results (.pdf). A summary breakdown of the model output for each species and additional maps showing predicted species richness and total biomass for a variety of species subsets, specifically, by origin (native or non-native), by status (common, local and rare) and by species order (Marsupialia, Insectivora, Carnivora, Artiodactyla, Chiroptera, Rodentia and Lagomorpha).
(PDF)

S3 File Species reports: Artiodactyla (.zip). Individual reports for each of the Artiodactyla species presenting analysis of the available data and subsequent model predictions based on a 10km raster grid. Reports also include expert comment assessing the reliability (and plausibility) of results in the context of existing evidence and popular opinion.
(ZIP)

S4 File Species reports: Carnivora (.zip). Individual reports for each of the Carnivora species presenting analysis of the available data and subsequent model predictions based on a 10km raster grid. Reports also include expert comment assessing the reliability (and plausibility) of results in the context of existing evidence and popular opinion.
(ZIP)

S5 File Species reports: Chiroptera (.zip). Individual reports for each of the Chiroptera species presenting analysis of the available data and subsequent model predictions based on a 10km raster grid. Reports also include expert comment assessing the reliability (and plausibility) of results in the context of existing evidence and popular opinion.
(ZIP)

S6 File Species reports: Insectivora (.zip). Individual reports for each of the Insectivora species presenting analysis of the available data and subsequent model predictions based on a 10km raster grid. Reports also include expert comment assessing the reliability (and plausibility) of results in the context of existing evidence and popular opinion.
(ZIP)

S7 File Species reports: Lagomorpha (.zip). Individual reports for each of the Lagomorpha species presenting analysis of the available data and subsequent model predictions based on a 10km raster grid. Reports also include expert comment assessing the reliability (and plausibility) of results in the context of existing evidence and popular opinion.
(ZIP)

S8 File Species reports: Marsupialia (.zip). Individual reports for each of the Marsupialia species presenting analysis of the available data and subsequent model predictions based on a 10km raster grid. Reports also include expert comment assessing the reliability (and plausibility) of results in the context of existing evidence and popular opinion.
(ZIP)

S9 File Species reports: Rodentia (.zip). Individual reports for each of the Rodentia species presenting analysis of the available data and subsequent model predictions based on a 10km raster grid. Reports also include expert comment assessing the reliability (and plausibility) of results in the context of existing evidence and popular opinion.
(ZIP)

S1 Table Excel version of S1 File: Table D (.xlsx). Excel spreadsheet containing Table S1 File: Table D for enhance readability.
(XLSX)
