# Supplementary material for: A systematic approach to estimate the distribution and total abundance of British mammals
Source: PLoS One. 2017 Jun 28;12(6):e0176339. doi: 10.1371/journal.pone.0176339 (PMC5489149; doi:10.1371/journal.pone.0176339)
Supplement: S1 File — A full description of the data identified and used in the modelling process including: a breakdown of the datasets available via the NBN, the permission received and a full list of acknowledgments; a breakdown of occurrence records by species; a breakdown of density estimates by species and a full list of references from which estimates were extract; full details of the environment data used for the habitat suitability modelling and a methodology for standardising this information to correspond with this process. (PDF) [file pone.0176339.s001.pdf]

Supplement 1: Data descriptions

A Occurrence data

A.1 National Biodiversity Network (NBN)

Table A. Occurrence records obtained via the NBN Gateway website summarised by provider detailing recording effort (total number of occurrence records), species richness (number of unique mammal species represented - max. 63), temporal range (range of recording year spanned by data), quality (resolution breakdown categorised as proportion of records at 100m;1km;2km;10km - it should be noted that the proportions listed are based on downloaded records for which the accuracy may be restricted and differ from that of the original), spatial extent (number of 10km grid cells containing records) and engagement (date of initial response to data request). Initial application to use the data was made on 27/02/2015 via the NBN platform (an additional email request was sent on 16/03/2015) and downloaded as access was granted until 30/06/2015. Values in brackets give the effort and richness of public access records available on 27/02/2015 where (-) denotes no public access. Response dates marked by ($^{+}$) indicate where enhance access to higher resolution data was granted. Similarly, (*) indicates where permission to use records was declined. NA and NR denote that no application was made or required to use the dataset(s) respectively.

**Data Provider**	**Records**	**Species**	**Period**	**Resolution**	**Extent**	**Response**
Aggregate Industries	0 (1)	0 (1)	-	-	0	NA
BATS & The Millennium Link	151 (151)	2 (2)	2001 - 2005	0;0;1;0	10	16/03/2015
Bedfordshire and Luton Biodiversity Recording and Monitoring Centre	13,420 (13,426)	32 (32)	1987 - 2014	0;0;0.9;0.1	23	16/03/2015
Biodiversity Information Service for Powys and Brecon Beacons National Park	4,150 (4,150)	37 (37)	1960 - 2008	0.22;0.02;0;0.76	75	02/03/2015
Biological Records Centre	110,930 (112,675)	58 (57)	1960 - 2014	0.12;0.5;0;0.37	2755	10/03/2015
Bristol Regional Environmental Records Centre	0 (17,826)	0 (45)	-	-	0	16/03/2015*
British Trust for Ornithology	106,184 (105,985)	38 (38)	1995 - 2011	0;1;0;0	1854	11/03/2015
Broadland Environmental Services Limited	1,017 (1,017)	1 (1)	2003 - 2006	1;0;0;0	10	NR
Buckinghamshire and Milton Keynes Environmental Records Centre	16,993 (17,050)	48 (50)	1960 - 2015	0.73;0.26;0;0^{+}	40	17/04/2015
Cambridgeshire & Peterborough Environmental Records Centre	4,698 (4,699)	13 (13)	1960 - 2014	0;0.06;0;0.94	47	02/03/2015
Central Scotland Green Network Trust	16 (16)	5 (5)	2013 - 2013	1;0;0;0	4	27/02/2015
Cofnod (North Wales Environmental Information Service)	1,431 (1,431)	37 (37)	1960 - 2014	0.27;0;0;0.73	79	04/03/2015
Cresswell Associates	0 (6)	0 (2)	-	-	0	NA
Cumbria Biodiversity Data Centre	53,951 (52,542)	46 (47)	1960 - 2014	0;0;0;1	90	27/04/2015
Derbyshire Biological Records Centre	7,443 (15,308)	2 (12)	1964 - 2014	0.8;0.2;0;0^{+}	46	15/04/2015
Devon Biodiversity Records Centre	23,267 (23,463)	44 (44)	1960 - 2014	0.86;0.14;0;0^{+}	106	11/03/2015
Dorset Environmental Records Centre	3,981 (2,905)	38 (26)	1960 - 2008	0.66;0.34;0;0.01^{+}	44	12/03/2015
Dr Francis Rose Field Notebook Project	0 (2)	0 (2)	-	-	0	NR
Dumfries and Galloway Environmental Resources Centre	62 (5,260)	2 (39)	1996 - 2013	0;0;0;1	22	22/12/2015
East Ayrshire Countryside Ranger Service	0 (76)	0 (19)	-	-	0	-
EcoRecord	0 (4,439)	0 (40)	-	-	0	-
Environment Agency (Biodiversity staff)	24 (25)	1 (2)	2008 - 2009	1;0;0;0	6	NR
Environmental Records Centre for Cornwall and the Isles of Scilly	47,729 (47,745)	43 (45)	1960 - 2013	0;0;0;1	69	09/03/2015
Environmental Records Information Centre North East	63,421 (62,015)	40 (42)	1960 - 2014	0;0;0;1	119	02/03/2015
Essex Wildlife Trust Biological Records Centre	656 (660)	2 (3)	1972 - 2013	0.98;0.02;0;0^{+}	52	02/03/2015
Fife Nature Records Centre	0 (4)	0 (4)	-	-	0	13/11/2015
Glasgow Museums BRC	1,978 (1,980)	31 (32)	1961 - 2008	0.81;0.16;0.02;0.01	75	02/04/2015
Gloucestershire Centre for Environmental Records	12,653 (12,655)	45 (45)	1965 - 2014	0.97;0.02;0;0^{+}	41	27/02/2015
Greater Lincolnshire Nature Partnership	25,050 (25,204)	43 (43)	1960 - 2014	0.26;0.03;0;0.71^{+}	110	20/03/2015
Greater Manchester Ecology Unit	5,420 (5,422)	38 (38)	1963 - 2015	0.91;0.09;0;0^{+}	31	09/03/2015
Greenspace Information for Greater London	0 (-)	0 (-)	-	-	0	02/03/2015*
Hampshire Biodiversity Information Centre	2,137 (2,137)	25 (25)	1964 - 2014	0;0.85;0;0.15	56	25/03/2015
Herefordshire Biological Records Centre	0 (8,756)	0 (44)	-	-	0	-
Hertfordshire Bat Group	0 (268)	0 (4)	-	-	0	-
Herts Environmental Records Centre	937 (622)	28 (23)	1983 - 2014	0.12;0;0;0.88	28	26/03/2015
Highland Biological Recording Group	24,417 (24,499)	39 (38)	1960 - 2015	0.6;0.25;0;0.15	527	02/03/2015
Humber Environmental Data Centre	0 (90)	0 (17)	-	-	0	-

Isle of Wight Local Records Centre	7,043 (7,043)	20 (20)	1980 - 2014	0.58;0.42;0;0[+]	12	18/03/2015
John Muir Trust	92 (92)	16 (16)	2007 - 2010	1;0;0;0	16	24/03/2015
Joint Nature Conservation Committee	11,977 (12,759)	1 (1)	1977 - 2002	1;0;0;0	1560	18/03/2015
Kent & Medway Biological Records Centre	0 (-)	0 (-)	-	-	0	-
Lancashire Environment Record Network	8,236 (8,236)	39 (39)	1960 - 2014	0.74;0.25;0;0.01[+]	45	16/03/2015
Leicestershire and Rutland Environmental Records Centre	6,576 (6,627)	41 (41)	1960 - 2014	0.89;0.11;0;0[+]	51	06/03/2015
Lorn Natural History Group	313 (219)	19 (17)	2004 - 2014	0.69;0.29;0.01;0	58	17/03/2015
Marine Conservation Society	1 (1)	1 (1)	2007 - 2007	1;0;0;0	1	NA
Merseyside BioBank	11,499 (11,503)	39 (40)	1960 - 2015	0.81;0.18;0.01;0[+]	14	16/03/2015
Ministry of Justice	98 (98)	20 (20)	2005 - 2014	1;0;0;0	14	NR
National Trust	7,467 (7,404)	52 (51)	1960 - 2014	0.78;0.22;0;0	352	12/03/2015
National Trust for Scotland	1,653 (2,062)	29 (33)	1979 - 2014	0.84;0.11;0;0.05	53	27/02/2015
Natural England	8,641 (8,641)	16 (16)	1960 - 2011	0.98;0.02;0;0	369	17/03/2015
Natural Resources Wales	8,391 (8,390)	26 (25)	1960 - 2006	0.15;0.85;0;0	216	27/02/2015
Nature Locator	0 (5)	0 (2)	-	-	0	NA
Norfolk Biodiversity Information Service	63,112 (64,090)	50 (48)	1960 - 2014	0.43;0.53;0;0.04[+]	80	10/03/2015
North & East Yorkshire Ecological Data Centre	0 (10,125)	0 (32)	-	-	0	-
North Ayrshire Countryside Ranger Service	284 (270)	21 (20)	1984 - 2010	1;0;0;0	2	29/04/2015
North East Scotland Biological Records Centre	21,596 (21,469)	36 (34)	1960 - 2014	0.85;0.12;0;0.03[+]	245	27/02/2015
Northamptonshire Biodiversity Records Centre	0 (176)	0 (1)	-	-	0	-
Northumberland Wildlife Trust	0 (8,679)	0 (2)	-	-	0	-
Nottinghamshire Biological and Geological Records Centre	1,953 (3,155)	2 (12)	1970 - 2014	0.91;0.09;0;0[+]	36	03/03/2015
Outer Hebrides Biological Recording Project	395 (309)	13 (12)	1969 - 2015	0.75;0.23;0;0.03[+]	47	16/03/2015
People's Trust for Endangered Species	97,138 (97,138)	18 (18)	1969 - 2014	0;1;0;0	1935	06/03/2015
Pond Conservation	0 (4)	0 (2)	-	-	0	NA
Record, the Biodiversity Information System for Cheshire, Halton, Warrington and the Wirral	31,182 (34,530)	47 (46)	1960 - 2015	0.62;0.31;0;0.07[+]	43	26/03/2015
Rotherham Biological Records Centre	0 (13,431)	0 (30)	-	-	0	-
Royal Horticultural Society	173 (171)	32 (32)	1983 - 2011	0.19;0.8;0;0.01	6	02/03/2015
RSPB	0 (71)	0 (2)	-	-	0	-
Scottish Natural Heritage	5,237 (5,259)	14 (16)	1962 - 2012	0.73;0.12;0;0.15[+]	702	27/02/2015
Scottish Wildlife Trust	68,763 (61,639)	26 (27)	1960 - 2015	0.66;0.33;0;0.01	655	13/04/2015
Sheffield Biological Records Centre	9,144 (8,476)	30 (29)	1960 - 2013	0;0;0;1	47	19/03/2015
Shire Group of Internal Drainage Boards	819 (819)	22 (22)	2000 - 2010	0.97;0.03;0;0	49	13/03/2015
Shropshire Ecological Data Network	6,896 (6,563)	43 (42)	1961 - 2014	0.9;0.1;0;0	50	NR
Somerset Environmental Records Centre	0 (350)	0 (1)	-	-	0	17/04/2015*
South East Wales Biodiversity Records Centre	1,753 (1,743)	36 (35)	1965 - 2011	0;0;0;1	61	09/09/2015 (NR)
St Helens Wildlife Recording Group	246 (246)	16 (16)	1996 - 2007	0;1;0;0	4	NR
Staffordshire Ecological Record	18,942 (18,760)	46 (46)	1960 - 2015	0.57;0.41;0;0.02[+]	56	14/04/2015
Suffolk Biological Records Centre	43,861 (44,803)	48 (48)	1960 - 2015	0.77;0.21;0.02;0[+]	57	01/03/2015
Surrey Biodiversity Information Centre	0 (13,993)	0 (36)	-	-	0	17/04/2015
Sussex Biodiversity Record Centre	404 (397)	24 (23)	1976 - 2009	0.95;0.04;0;0[+]	23	27/02/2015
Thames Valley Environmental Records Centre	1745 (545)	38 (38)	1973 - 2012	0.9;0.03;0;0.07[+]	51	03/03/2015
The Bat Conservation Trust	46,907 (44,618)	37 (36)	1974 - 2014	0.3;0.52;0;0.18	1676	23/04/2015
The Mammal Society	27,893 (27,544)	57 (54)	1970 - 2015	0.87;0.09;0.04;0.01	2576	05/03/2015
The Wildlife Information Centre	2,844 (2,096)	24 (20)	1992 - 2013	0.9;0.1;0;0[+]	73	16/03/2015
Tullie House Museum	69 (68)	25 (24)	1960 - 2006	0;0;0.93;0.07	31	16/03/2015
Warwickshire Biological Records Centre	0 (4,824)	0 (18)	-	-	0	-
Welsh Government	1,231 (1,466)	8 (9)	2009 - 2012	0;1;0;0	116	27/03/2015
West Wales Biodiversity Information Centre	736 (735)	32 (31)	1968 - 2009	0.11;0.01;0;0.88	50	11/03/2015
Wiltshire and Swindon Biological Records Centre	37,859 (37,236)	46 (46)	1960 - 2014	0.78;0.22;0;0[+]	57	06/05/2015
Worcestershire Biological Records Centre	0 (26,361)	0 (46)	-	-	0	-
Yorkshire Wildlife Trust	919 (1,023)	24 (31)	1976 - 2014	0.44;0.56;0;0[+]	42	16/03/2015

The information used in this publication to create maps and perform analysis included the following resources sourced via the NBN Gateway website.

BATS & The Millennium Link[1]: "Limited bat records from the Union & Forth and Clyde canals 2000-5". **Bedfordshire and Luton Biodiversity Recording and Monitoring Centre:** "Bedfordshire Brown Hares (BNHS) - 1990-2013"; "Bedfordshire Dormice (BNHS/BDG) - 2000-2012"; "Bedfordshire Mammals (BNHS) 1987-2013". **Biodiversity Information Service for Powys and Brecon Beacons National Park:** "Natural Resources Wales Regional Data: Mid-Wales"; "Natural Resources Wales Regional Data: Mid-Wales, sensitive species at 10km square resolution". **Biological Records Centre:** "Mammal records from Britain from the Atlas of Mammals (1993), with some subsequent records"; "RISC Non-Native Species Records for Muntjac". **British Trust for Ornithology:** "BTO/JNCC/RSPB Breeding Bird Survey (Mammals)"; "Non-native-bird records from WeBS and BBS"[2]. **Broadland Environmental Services Limited:** "Water vole distribution within Broadland (2003-2006): based upon field signs, sightings and trapping data". **Buckinghamshire and Milton Keynes Environmental Records Centre:** "Mammals in Buckinghamshire". **Cambridgeshire & Peterborough Environmental Records Centre:** "CPERC Recorders day at Waterbeach barracks and airfield"; "Habitats and Species Directive Annexes

2, 4 & 5 Species Records"; "Invasive Non-Native Species"; "NERC S41 species records". **Central Scotland Green Network Trust:** "South Lanarkshire peatland records 2013**". Cofnod (North Wales Environmental Information Service):** "NRW Regional Data: North Wales (Non-Sensitive Records)"; "NRW Regional Data: North Wales (Sensitive Records)". **Cumbria Biodiversity Data Centre:** "Cumbria Biodiversity Data Centre vertebrate species observations for Cumbria for the period 1512 to 2012"; "Wildwatch North Pennines AONB project records for Cumbria". **Derbyshire Biological Records Centre:** "Derbyshire Wildlife Trust Otter Records up to Dec 2013"; "Derbyshire Wildlife Trust Water Vole Records up to Dec 2013"; **Devon Biodiversity Records Centre:** "Devon bat roost data"; "Devon Climate Change survey 2002-2008"; "Devon incidental species records 1950-2002"; "Habitats Directive (Annex 2, 4, 5) Species Devon"; "Invasive Non-Native Species (INNS) Devon"; "NERC Act Species Devon". **Dorset Environmental Records Centre:** "Dorset Sites of Nature Conservation Interest (SNCI) species records 2000-2008"; "Dorset Sites of Nature Conservation Interest (SNCI) species records pre 2000"; "Dorset SSSI Species Records 1952 - 2004 (Natural England)"; "Dorset SW Pilot species dataset"; "Dorset Wildlife Trust Reserve Records"; "Tracking the Dorset Hare - NBN South West Pilot Project Case Studies". **Environment Agency (Biodiversity staff):** "Environment Agency Rare and Protected Species records v1". **Environmental Records Centre for Cornwall and the Isles of Scilly:** "Terrestrial mammal records for Cornwall and the Isles of Scilly from 1800 to 2013". **Environmental Records Information Centre North East:** "ERIC North East Species Records - 2000-2014"; "ERIC North East Species Records - Up to 1999". **Essex Wildlife Trust Biological Records Centre:** "Invasive non-native species records for Essex from 1979 to January 2014"; "Selected species records for Essex, 1958 - 2013". **Glasgow Museums BRC:** "Mammal records for Clyde Faunal Area, 1850 to 2007". **Gloucestershire Centre for Environmental Records[3]:** "Gloucestershire Wildlife Observations historic data"; "Gloucestershire Wildlife Observations since 1st January 2004". **Greater Lincolnshire Nature Partnership[4]:** "Badger records in Lincolnshire"; "Lincolnshire Bats (roosts)"; "Lincolnshire Bats (sightings)"; "Lincolnshire Mammals (excluding bats)". **Greater Manchester Ecology Unit:** "Distribution of Badgers in Greater Manchester"; "Distribution of Bats in Greater Manchester"; "Distribution of Hares in Greater Manchester"; "Distribution of Invasive and Non-Native Species in Greater Manchester"; "Distribution of Mammals in Greater Manchester"; "Distribution of Species of Conservation Interest in Greater Manchester"; "Distribution of Water Voles in Greater Manchester". **Hampshire Biodiversity Information Centre:** "HBIC and partners species records"; "HBIC Non-Native Invasive Species". **Herts Environmental Records Centre:** "Hertfordshire Amphibian Survey 2002"; "Hertfordshire Habitat Survey 1995 - 1997"; "Hertfordshire Local Wildlife Site Surveys"; "Hertfordshire Miscellaneous records"; "Hertfordshire Otter Records 2002 -"; "Hertfordshire Pond Survey 1986"; "Hertfordshire Urban Surveys (incomplete)"; "Hertfordshire Water Vole Monitoring 1996"; "Hertfordshire Water Vole Records 1995-2008"; "HMWT - Water Vole Project"; "South Herts Phase 1 Survey 1984-1989 (Hertfordshire)". **Highland Biological Recording Group:** "HBRG Badger dataset"; "HBRG Vertebrates (not Badger) Dataset". **Isle of Wight Local Records Centre:** "Isle of Wight rare and protected species". **John Muir Trust:** "Species Records for John Muir Trust Properties 2007-2009"; "Species Records for John Muir Trust Properties Nevis, Sandwood, Quinag and Schiehallion 2010". **Joint Nature Conservation Committee:** "England Otter Survey Database"; "Marine Nature Conservation Review (MNCR) and associated benthic marine data held and managed by JNCC"; "Scotland Otter Survey Database"; "Wales Otter Survey Database". **Lancashire Environment Record Network:** "LERN Invasive Non-native Species Records"; "LERN Records". **Leicestershire and Rutland Environmental Records Centre:** "Leicestershire and Rutland Bats"; "Leicestershire and Rutland Invasive Non-Native Species"; "Leicestershire and Rutland Otter Records"; "Leicestershire and Rutland Protected and Conservation Priority/BAP Species"; "Leicestershire and Rutland Water Vole Records"; "Leicestershire Mammal Records to end of 2009". **Lorn Natural History Group:** "LNHG Biological Records Dataset". **Merseyside BioBank:** "Merseyside BioBank (unverified)"; "Merseyside BioBank (verified)"; "Merseyside BioBank Active Naturalists (unverified)"; "Merseyside BioBank Active Naturalists (verified)". **Ministry of Justice:** "Species found in the NOMS estate 2005 - Present". **The National Trust:** "Anglesey Abbey wildlife species data held by The National Trust"; "Hatfield Forest species data held by The National Trust"; "Heigham Holmes species data held by The National Trust"; "Ickworth species data held by The National Trust"; "National Trust Species Records"; "Sutton Hoo species data held by The National Trust"; "Wicken Fen nature reserve species data held by The National Trust"; "Wimpole Estate species data held by The National Trust". **National Trust for Scotland:** "National Trust for Scotland Sensitive Species Records 1800-2014"; "National Trust for Scotland Species Records 1800-2014". **Natural England:** "Batsites inventory for England (1949-2011)"; "Invertebrate Site Register - England (1738-2005)". **Natural Resources Wales:** "Bat Records - Montgomeryshire"; "Bat Roosts Database - Pembrokeshire"; "Bat Roosts Database- South Wales"; "Dormouse Licence Return Data"; "Important Welsh Bat Roosts Database (BATSITES-WALES)"; "Marine records from Pembrokeshire Marine Species Atlas"; "Natural Resources Wales North Wales Bat Roosts"; "Squirrel Collation for Wales"; "Welsh Invertebrate Database (WID)"; "Welsh Lesser Horseshoe Bat Summer Roost Surveillance Database". **Norfolk Biodiversity Information Service:** "NBIS records to Nov 2014". **North Ayrshire Countryside Ranger Service:** "Species within North Ayrshire from 1984 - Present". **North East Scotland Biological Records Centre:** "NE Scotland Wildcat records 1800-2011"; "North East Scotland Terrestrial Mammals 1900-2014 (excluding squirrels, wild cats and marine mammals)". **Nottinghamshire Biological and Geological Records Centre:** "Nottinghamshire Biodiversity Action Group Brown Hare Survey"; "Records of Bats in Nottinghamshire"; "Records of Water Vole in Nottinghamshire". **Outer Hebrides Biological Recording Project:** "Invasive Non-Native Species, Outer Hebrides"; "Otter (Lutra lutra), Outer Hebrides"; "Vertebrates (except birds, INNS and restricted records), Outer Hebrides". **People's Trust for Endangered Species:** "Hedgehog Hibernation Survey records"; "Hedgehog records 2005-2006: Hogwatch and Rural Landowner surveys"; "Living with Mammals survey: sightings from 2003 to 2011"; "National Dormouse Database (NDD)". **Record, the Biodiversity Information System for Cheshire, Halton, Warrington and the Wirral:** "RECORD Chiroptera Data"; "RECORD Mammals excl badgers & bats". **Royal Horticultural Society:** "RHS monitoring of native and naturalised plants and animals at its gardens and surrounding areas". **Scottish Natural Heritage:** "Bat Records for Scotland 1970-2007"; "Compilation of records of 12 Article 17 terrestrial mammal species in Scotland"; "Distribution of Scottish wildcats (Felis silvestris) in Scotland (2006-2008) û database extract (breeding and non-breeding records)"; "SNH Species Repository". **Scottish Wildlife Trust:** "Casual records for Scottish Wildlife Trust reserves - Verified data"; "Commissioned surveys and staff surveys and reports for Scottish Wildlife Trust reserves - Unassessed data"; "Commissioned surveys and staff surveys and reports for Scottish Wildlife Trust reserves - Verified data"; "Survey and monitoring records for Scottish Wildlife Trust reserves from reserve convenors and Trust volunteers - Unassessed data"; "Survey and monitoring records for Scottish Wildlife Trust reserves from reserve convenors and Trust volunteers - Verified data"; "The Scottish Squirrel Database**". Sheffield Biological Records Centre:** "Sheffield Biological Records Centre- Non-sensitive Records from all taxonomic groups". **Shire Group of Internal Drainage Boards:** "Shire Group IDB species data 2004 to present". **Shropshire Ecological Data Network:** "Shropshire Ecological Data Network database". **South East Wales Biodiversity Records Centre:** "NRW Regional Data: South East Wales Non-sensitive Species Records". **St Helens Wildlife Recording Group:** "St Helens Wildlife Dataset". **Staffordshire Ecological Record:** "Data from Consultancy Reports supplied to Staffordshire Ecological Record"; "SER Site-based Surveys"; "SER Species-based Surveys"; "Staffordshire Wildlife Trust Nature Reserves Inventory"; "Stoke-on-Trent Environmental Survey results (1982-1984)". **Suffolk Biological Records Centre:** "Suffolk Biological Records Centre (SBRC) dataset". **Sussex**

Biodiversity Record Centre: "Patrick Roper's Notebooks". **Thames Valley Environmental Records Centre:** "2000-2003 RBWM WHS Surveys"; "BBOWT Local Wildlife Sites Surveys 1994-2003"; "English Nature and NCC Oxfordshire Surveys"; "English Nature Berkshire SSSI Records"; "Local Wildlife Site Surveys Berkshire"; "Local Wildlife Site Surveys Oxfordshire"; "Natural England Bat Records for Berkshire and Oxfordshire (Held by Thames Valley Environmental Records Centre)"; "Nature Conservancy Council Oxfordshire Fen Survey 1983 (as held by Thames Valley Environmental Records Centre)"; "Nature Conservancy Council Survey of Ancient Woodlands in Berkshire"; "NCC Berkshire Ancient Woodland Survey 1977-87"; "NCC Berkshire Chalk Grassland Survey 1985"; "NCC Berkshire Grassland Survey 1984-1987". **The Bat Conservation Trust:** "Bechstein's Bat Survey Project"; "Field Survey"; "Hibernation Survey"; "Nathusius' Pipistrelle Survey"; "Natural England bot roost visit records from 2013 onwards"; "Roost Count"; "The BCT/MTUK Bats & Roadside Mammals Survey"; "Waterway Survey"; "Woodland Survey". **The Mammal Society:** "National Mammal Atlas Project, online recording". **The Wildlife Information Centre:** "Borders Backyard Biodiversity"; "Lothian Wildlife Information Centre Secret Garden Survey"; "TWIC Biodiversity Field Trip Data (1995-present)"; "TWIC Hedgehog Public Survey ". **Tullie House Museum:** "Tullie House Museum Natural History Collections". **Welsh Government:** "Tir Cynnal and Tir Gofal Monitoring and Evaluation Programme: Brown Hare Farm-scale surveys"; "Tir Cynnal and Tir Gofal Monitoring and Evaluation Programme: Field Survey; farm-scale survey for noctule, common pipistrelle and soprano pipistrelle"; "Tir Cynnal and Tir Gofal Monitoring and Evaluation Programme: Water Vole Farm-scale Surveys". **West Wales Biodiversity Information Centre:** "NRW Regional Data: all taxa (excluding sensitive species), West Wales"; "NRW Regional Data: all taxa (sensitive species only), West Wales". **Wiltshire and Swindon Biological Records Centre:** "Wiltshire & Swindon Incidental Species Records"; "Wiltshire & Swindon Site-based Survey Records". **Yorkshire Wildlife Trust:** "Yorkshire Wildlife Trust - Non-sensitive records from all taxonomic groups"; "Yorkshire Wildlife Trust - Sensitive records from all taxonomic groups".

[1]Specific acknowledgement to Neil Middleton (Bats & The Millennium Link, www.batml.org.uk).
[2]Specific acknowledgement to BTO/JNCC/RSPB (for BBS) and BTO/RSPB/JNCC in association with WWT (for WeBS).
[3]Prepared using historic data from Gloucestershire Centre for Environmental Records as supplied by the National Biodiversity Network on 1/3/2015.
[4]Additional acknowledgment to The Lincolnshire Naturalists' Union (LNU) and Lincolnshire Bat Group (LBG).

The data providers and NBN Trust bear no responsibility for the further analysis or interpretation of this material, data and/or information. - See more at: http://www.nbn.org.uk/Use-Data/Using-Maps-or-Data/Using-and-referencing-data-from-the-Gateway.aspx#sthash.goMHyenU.dpuf.

S1.1.2 Additional datasets

Following application via the NBN several data providers offered access to more up to date and/or comprehensive datasets than were currently held on the platform. We would like to acknowledge the support of these organisations and provide summary details of each dataset below.

Environmental Records Information Centre North East:
Records: 68,848; Species: 41; Period: 1960-2015; Resolution: (0.71, 0.28, 0.01, 0); Coverage: 118.

Glasgow Museums BRC:
Records: 4,291; Species: 33; Period: 1961-2015; Resolution: (0.9, 0.09, 0.01, 0); Coverage: 142.

Highland Biological Recording Group:
Records: 1,744; Species: 1 (Badger); Period: 1961-2015; Resolution: (0, 0.92, 0, 0.08); Coverage: 192.

S1.1.3 Occurrence records by species

Table B. Occurrence records obtained via the NBN Gateway website summarised by species detailing the estimate of abundance as presented by Harris et al. (1995) (numbers in subscript indicate the reliability of the estimate, where 5 denotes the least, and 1 most, reliable), recording effort (total number of occurrence records), temporal range (range of recording year spanned by data; median shown in brackets), data quality (resolution breakdown categorised as proportion of records at 100m;1km;2km;10km - it should be noted that the proportions listed are based on downloaded records for which the accuracy may be restricted and differ from that of the original), spatial extent (number of 10km grid cells containing records) and recording rate (% of estimated 1995 population recorded per year between 1960 and 2015; ranking shown in brackets). The mean body mass (from Harris & Yalden 2008) is also given. Species are ordered by recording rate (highest to lowest).

Species Name	1995 abundance	Records	Period	Resolution	Extent	Recording rate	Mass (kg)
Otter	7,350 (3)	60,258	1960 - 2015 (2000)	0.63;0.10;0.01;0.25	2,345	14.91 (1)	8
Chinese water deer	650 (2)	3,957	1961 - 2015 (2004)	0.53;0.28;0.13;0.05	144	11.07 (2)	15
Red-necked wallaby	29 (1)	116	1961 - 2014 (2003)	0.06;0.75;0.08;0.11	25	7.273 (3)	12
Greater horseshoe bat	4,000 (2)	10,103	1960 - 2014 (1996)	0.51;0.29;0;0.2	282	4.592 (4)	0.02
Lesser horseshoe bat	14,000 (2)	24,194	1960 - 2014 (1999)	0.34;0.41;0;0.25	438	3.142 (5)	0.005
Pine marten	3,650 (2)	3,009	1960 - 2015 (2004)	0.55;0.32;0;0.14	495	1.499 (6)	1.5
Red squirrel	160,000 (3)	106,973	1960 - 2015 (2006)	0.52;0.14;0;0.35	1,234	1.216 (7)	0.2
Bechstein's bat	1,500 (4)	957	1960 - 2014 (2007)	0.72;0.22;0;0.06	100	1.16 (8)	0.01
Chinese muntjac	40,000 (3)	16,645	1960 - 2015 (2005)	0.32;0.42;0.08;0.18	990	0.757 (9)	13
Park cattle	45 (1)	16	1995 - 2011 (2009)	0.31;0.56;0;0.13	8	0.646 (10)	150
Barbastelle	5,000 (5)	1,769	1960 - 2014 (2008)	0.57;0.23;0;0.2	281	0.643 (11)	0.008
Polecat	15,000 (3)	4,798	1960 - 2015 (1996)	0.61;0.24;0.03;0.12	682	0.582 (12)	0.9
Serotine	15,000 (4)	4,735	1960 - 2014 (2005)	0.52;0.45;0;0.04	561	0.574 (13)	0.032
Feral ferret	2,500 (5)	690	1960 - 2014 (1998)	0.27;0.27;0.01;0.44	353	0.502 (14)	0.9
Wildcat	3,500 (3)	839	1960 - 2011 (1995)	0.32;0.19;0;0.49	265	0.436 (15)	4.5
Red fox	240,000 (4)	56,658	1960 - 2015 (2005)	0.3;0.5;0.03;0.18	2,334	0.429 (16)	6
Noctule	50,000 (3)	10,568	1960 - 2014 (2007)	0.5;0.35;0;0.15	1,126	0.384 (17)	0.032
Badger	250,000 (1)	44,958	1960 - 2015 (2003)	0.36;0.23;0;0.41	2,149	0.327 (18)	10.5
Sika deer	11,500 (2)	1,971	1960 - 2014 (1993)	0.10;0.35;0.01;0.55	436	0.312 (19)	50
Grey long-eared bat	1,000 (3)	166	1960 - 2014 (1998)	0.69;0.28;0;0.02	38	0.302 (20)	0.009
Feral goat	3,565 (2)	576	1960 - 2015 (1999)	0.39;0.37;0.01;0.24	137	0.294 (21)	45
Ship rat	1,300 (2)	186	1960 - 2014 (1971)	0.06;0.4;0;0.54	86	0.260 (22)	0.175
Natterer's bat	100,000 (4)	13,625	1960 - 2015 (2005)	0.46;0.29;0;0.25	1,103	0.248 (23)	0.008
Brown long-eared bat	200,000 (4)	26,804	1960 - 2015 (2004)	0.54;0.2;0;0.26	1,650	0.244 (24)	0.008
Daubenton's bat	150,000 (4)	19,622	1960 - 2015 (2005)	0.66;0.17;0.01;0.16	1,375	0.238 (25)	0.08
American mink	110,000 (3)	13,506	1960 - 2015 (2000)	0.49;0.24;0.02;0.25	1,568	0.223 (26)	0.8
Roe deer	500,000 (3)	42,459	1960 - 2015 (2003)	0.31;0.37;0.01;0.31	2,047	0.154 (27)	20
Fallow deer	100,000 (4)	8,447	1960 - 2015 (2000)	0.28;0.37;0.04;0.32	1,045	0.154 (28)	50
Brown hare	817,500 (2)	54,989	1960 - 2015 (2003)	0.34;0.45;0;0.21	2,151	0.122 (29)	3.3
Whiskered bat	40,000 (4)	2,543	1960 - 2015 (1999)	0.56;0.23;0;0.21	608	0.116 (30)	0.006
Grey squirrel	2,520,000 (3)	149,368	1960 - 2015 (2007)	0.28;0.52;0.01;0.19	1,954	0.108 (31)	0.5
Leisler's bat	10,000 (4)	524	1961 - 2014 (2006)	0.68;0.15;0;0.16	193	0.095 (32)	0.025
Hedgehog	1,555,000 (4)	77,902	1960 - 2015 (2006)	0.33;0.52;0.01;0.14	2,347	0.091 (33)	0.5
Water vole	1,169,000 (3)	48,476	1960 - 2015 (2003)	0.7;0.14;0.01;0.15	1,609	0.075 (34)	0.3
Stoat	462,000 (4)	17,897	1960 - 2015 (1998)	0.36;0.37;0.02;0.25	1,987	0.070 (35)	0.25
Feral sheep	2,100 (1)	78	1960 - 2014 (1997)	0.58;0.26;0;0.17	29	0.068 (36)	20
Red deer	360,000 (2)	12,910	1960 - 2015 (1997)	0.31;0.34;0.01;0.34	1,277	0.065 (37)	75
Fat dormouse	10,000 (3)	309	1963 - 2014 (2001)	0.31;0.57;0.11;0.02	32	0.056 (38)	0.14
Weasel	450,000 (4)	13,367	1960 - 2015 (1994)	0.32;0.42;0.02;0.23	1,893	0.054 (39)	0.075
Brandt's bat	30,000 (5)	868	1960 - 2015 (2000)	0.54;0.23;0;0.23	228	0.053 (40)	0.006
Common dormouse	500,000 (3)	11,541	1960 - 2014 (2001)	0.55;0.30;0.02;0.13	621	0.042 (41)	0.018
Pipistrelle	2,000,000 (3)	29,184	1960 - 2014 (2000)	0.42;0.4;0;0.17	1,919	0.027 (42)	0.006
Mountain hare	350,000 (3)	4,374	1960 - 2014 (1999)	0.36;0.37;0;0.27	572	0.023 (43)	2.8
Lesser white-toothed shrew	14,000 (4)	104	1961 - 2012 (1998)	0;0.15;0;0.85	4	0.014 (44)	0.005
Feral cat	813,000 (4)	6,028	1976 - 2014 (2005)	0.04;0.96;0;0	794	0.0135 (45)	3.5
Common rat	6,790,000 (4)	27,686	1960 - 2015 (2007)	0.16;0.56;0.01;0.27	1,888	0.0074 (46)	0.25
Rabbit	37,500,000 (3)	102,381	1960 - 2015 (2003)	0.19;0.67;0.01;0.13	2,539	0.005 (47)	1.5
Harvest mouse	1,425,000 (5)	3,784	1960 - 2014 (1989)	0.41;0.37;0.02;0.19	843	0.0048 (48)	0.006
Water shrew	1,900,000 (4)	3,852	1960 - 2015 (1999)	0.37;0.31;0.03;0.29	1,080	0.0037 (49)	0.015
Mole	31,000,000 (3)	56,294	1960 - 2015 (2002)	0.32;0.38;0.02;0.28	2,384	0.003 (50)	0.1
Yellow-necked mouse	750,000 (4)	1,354	1960 - 2014 (1996)	0.63;0.27;0.05;0.06	320	0.0033 (51)	0.03
Pygmy shrew	8,600,000 (4)	5,080	1960 - 2015 (1988)	0.32;0.37;0.04;0.28	1,309	0.0011 (52)	0.004
House mouse	5,192,000 (5)	2,536	1960 - 2015 (2001)	0.25;0.38;0.04;0.32	461	0.0009 (53)	0.015
Wood mouse	38,000,000 (3)	16,396	1960 - 2015 (2001)	0.43;0.27;0.03;0.26	1,831	0.0008 (54)	0.02
Bank vole	23,000,000 (3)	9,591	1960 - 2015 (2000)	0.38;0.29;0.04;0.28	1,459	0.0008 (55)	0.02
Common shrew	41,700,000 (3)	12,513	1960 - 2015 (1994)	0.34;0.36;0.03;0.27	1,877	0.0005 (56)	0.01
Field vole	75,000,000 (4)	12,668	1960 - 2015 (2000)	0.38;0.31;0.02;0.29	1,851	0.0003 (57)	0.02
Orkney vole	1,000,000 (1)	8	2002 - 2002 (2002)	1;0;0;0	1	0.00002 (58)	0.025
Skomer vole	7,000 (1)	0	-	0;0;0;0	0	-	0.025
Coypu	0 (1)	2,312	1960 - 2012 (1977)	0.01;0.04;0;0.95	193	-	6.25
Greater mouse-eared bat	0 (1)	61	1960 - 2013 (2004)	0.36;0.31;0;0.33	8	-	0.032
Nathusius' pipistrelle	-	659	1985 - 2014 (2011)	0.58;0.24;0;0.18	214	-	0.006
Wild boar	-	60	1976 - 2014 (2008)	0.82;0.17;0;0.02	23	-	60*

B Density data

B.1 Density mapping process

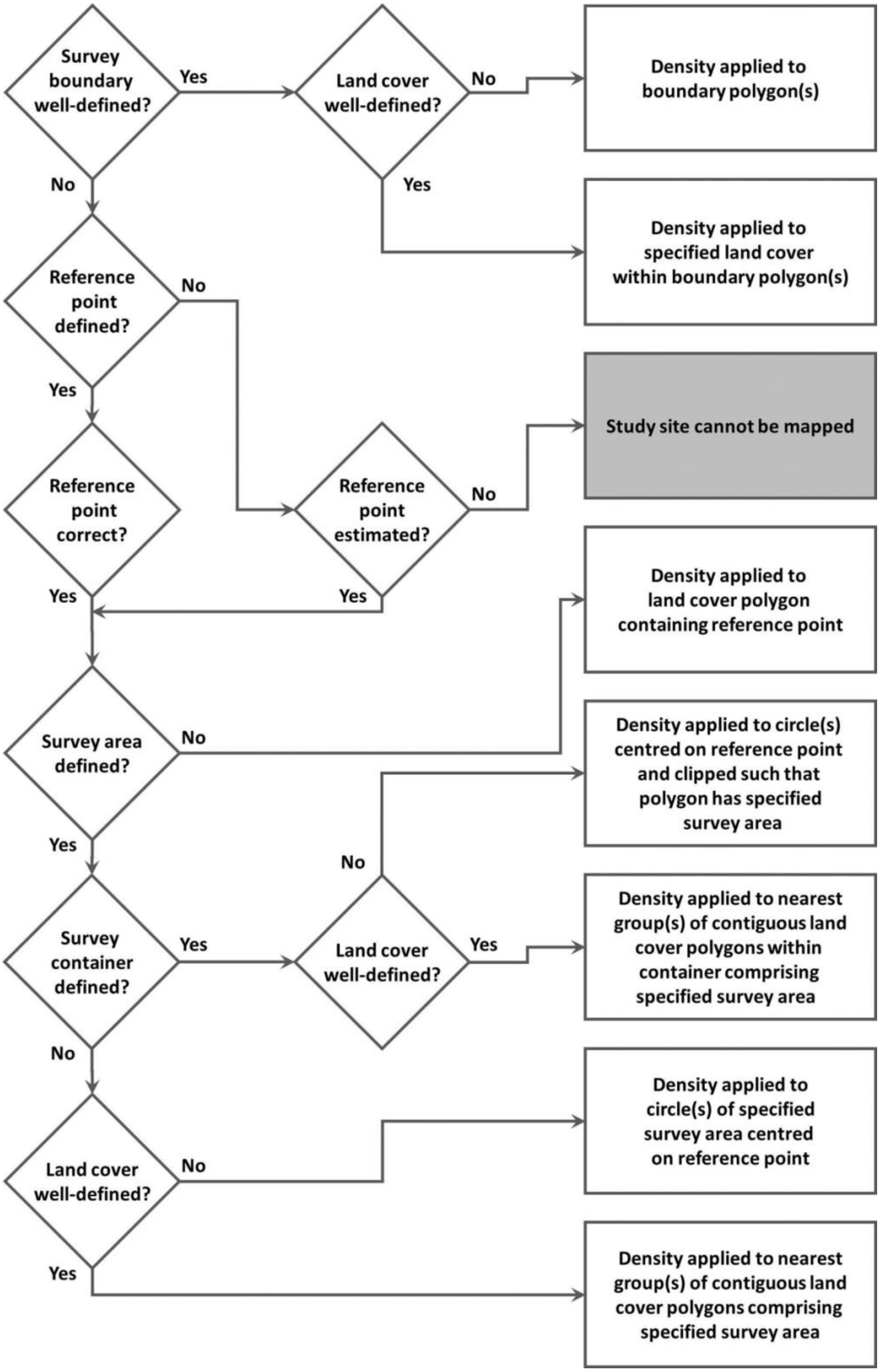

Fig A. Decision tree outlining the process followed to map a survey site (approximate a geo-referenced polygon) from the description given in a peer-review publication. Possible outcomes are shown on the right and represent varying levels of assumption dependent on the quality of geographic information available.

B.2 Density estimates by species

Table C. Density estimates obtained via a comprehensive search of peer-reviewed literature by species detailing the number of available estimates (values in brackets indicate the number of unique study sites), the temporal period over which they span, the range of densities recorded (per km^2), the range of survey areas (km^2), the spatial extent of surveyed areas (number of 10km grid cells containing a density estimate - values in brackets indicate the spatial extent of corresponding occurrence records) and references. Species are ordered by the number of available density estimates (highest to lowest).

Species Name	Estimates	Period	Density	Area	Extent	References
Wood mouse	418 (38)	1960 - 2010	0 - 11,975	0.1 - 6.9	38 (1,835)	Baker et al. (2005); Bush et al. (2012); Flowerdew & Elwood (2001); Flowerdew et al. (2004); Forman (2005); Gelling et al. (2007); Kotzageorgis & Mason (1997); Mallorie & Flowerdew (1994); Shore et al. (2005); Tattersall et al. (2002); Telfer et al. (2007)
Bank vole	413 (38)	1960 - 2010	0 - 15,309	0.01 - 6.9	38 (1,463)	Baker et al. (2005); Bush et al. (2012); Flowerdew & Elwood (2001); Flowerdew et al. (2004); Forman (2005); Gelling et al. (2007); Kotzageorgis & Mason (1997); Mallorie & Flowerdew (1994); Shore et al. (2005); Tattersall et al. (2002); Telfer et al. (2007)
Brown hare	112 (62)	1982 - 2006	0 - 77.3	0.4 - 1,458	338 (2,175)	Forman (2005); Heydon et al. (2000); Langbein et al. (1999); Parrott et al. (2012); Reynolds et al. (2009); Smith et al. (2004); Tapper & Barnes (1986)
Field vole	100 (15)	1984 - 2004	1.4 - 30,923	0.01 - 6.9	25 (1,853)	Burthe et al. (2006); Forman (2005); Gelling et al. (2007); Kotzageorgis & Mason (1997); Lambin et al. (2000); O'Mahoney et al. (1998); Shore et al. (2005); Tattersall et al. (2002)
Red deer	96 (40)	1960 - 2013	0.1 - 29.8	107 - 2,636	493 (1,304)	Edwards et al. (2013); Lowe (1969)
Red squirrel	89 (14)	1984 - 2000	3.2 - 422	0.06 - 2	16 (1,234)	Bryce et al. (2002); Gurnell et al. (2004b); Halliwell (1997); Holm (1991); Kenward et al. (1998); Shuttleworth (1996); Wauters et al. (2000)
Water vole	88 (18)	1996 - 2004	0 - 293,450	0.0002 - 25	21 (1,609)	Aar et al. (2001); Benge (2004); Forman (2005); Telfer et al. (2003)
Common shrew	77 (38)	1969 - 2005	0 - 9,718	0.004 - 6.9	32 (1,883)	Baker et al. (2005); Churchfield & Brown (1987); Gelling et al. (2007); Kotzageorgis & Mason (1997); Pernetta (1977); Shore et al. (2005); Tattersall et al. (2002); White & Searle (2007)
Pygmy shrew	44 (8)	1969 - 2000	0 - 2,852	0.004 - 6.9	7 (1,310)	Churchfield & Brown (1987); Kotzageorgis & Mason (1997); Pernetta (1977); Shore et al. (2005); Tattersall et al. (2002)
Sika deer	44 (44)	1993 - 1998	0 - 25.6	0.85 - 99.6	50 (455)	Marques et al. (2001); Putman & Clifton-Bligh (1997)
Mountain hare	32 (7)	1960 - 1971	3.7 - 89	1 - 5.9	8 (574)	Watson et al. (1973)
Brown long-eared bat	30 (3)	1987 - 2009	1.4 - 14.9	15.5 - 625	47 (1,672)	Speakman et al. (1991); Jones et al. (1996); Fairless (2013)
Natterer's bat	28 (2)	1990 - 2009	1.8 - 24	15.5 - 175	12 (1,107)	Jones et al. (1996); Fairless (2013)
Badger	26 (20)	1992 - 2006	1.2 - 43	2.4 - 887	270 (2,153)	Delahay et al. (2006); Heydon et al. (2000); Hounsome et al. (2005); Hutchings et al. (2001); Hutchings et al. (2002); Macdonald & Newman (2002); Macdonald et al. (2009); Palphramand et al. (2007); Parrott et al. (2012); Rogers et al. (1997); Roper et al. (1993)
Red fox	26 (12)	1993 - 2006	0.14 - 27.6	1.5 - 1,458	297 (2,338)	Baker et al. (2004); Heydon et al. (2000); Lambin (2000); Parrott et al. (2012)
Wild boar	25 (2)	1995 - 2000	0.02 - 0.65	46 - 6,478	98 (114)	Wilson (2003)
House mouse	19 (5)	1960 - 1997	0 - 3,750	0.02 - 1	4 (463)	Berry (1968); Tattersall et al. (2002)
Pipistrelle	19 (3)	1987 - 2009	1.6 - 18.2	15.5 - 875	54 (1,931)	Speakman et al. (1991); Jones et al. (1996); Fairless (2013)
Feral sheep	18 (5)	1961 - 1990	24 - 173	1 - 6.4	9 (38)	Bullock (1985); Clutton-Brock et al. (1991); Harris et al. (1995)

Species Name	Estimates	Period	Density	Area	Extent	References
Roe deer	18 (5)	1967 - 2002	16 - 76	1.5 - 185	16 (2,047)	Gill et al. (1996); Gill et al. (1997); Hemami et al. (2005); Hemami et al. (2007); Malyle (1996)
Feral goat	15 (15)	1978 - 1991	0.9 - 11.4	1.4 - 109	17 (141)	Bullock (1985); Harris et al. (1995); Hellawell (1991)
Grey squirrel	12 (7)	1987 - 2000	8 - 169	0.27 - 1	13 (1,954)	Bryce et al. (2002); Gurnell et al. (2004a); Gurnell et al. (2004b); Kenward et al. (1998); Wauters et al. (2000)
Pine marten	12 (12)	1989 - 1998	0.12 - 0.82	24 - 110	55 (499)	Bright et al. (1999); Halliwell (1997)
Bechstein's bat	11 (1)	2001 - 2011	245 - 329	0.38	1 (100)	Fairless (2013)
American mink	10 (10)	1974 - 2006	1.6 - 70	0.2 - 186	17 (1,577)	Birks (1981); Bonesi & Macdonald (2004); Harrington et al. (2008); Moore et al. (2003)
Hedgehog	10 (10)	1979 - 2002	21 - 179	0.3 - 100	52 (2,348)	Doncaster (1994); Morris et al. (1992); Reeve (1981); Young et al. (2006)
Polecat	10 (10)	1996 - 1996	0 - 1.86	Apr-18	584 (790)	Birks (1997)
Yellow-necked mouse	8 (6)	1990 - 2003	0 - 88.7	0.02 - 6.9	6 (322)	Gelling et al. (2007); Kotzageorgis & Mason (1997); Tattersall et al. (2002)
Mole	7 (7)	1982 - 1990	420 - 850	0.04 - 0.09	4 (2,384)	Macdonald et al. (1997); Stone (1986)
Harvest mouse	7 (5)	1990 - 1997	0 - 3.49	0.2 - 6.9	4 (844)	Kotzageorgis & Mason (1997); Tattersall et al. (2002)
Common dormouse	6 (1)	2002 - 2007	14 - 311	0.12	1 (621)	Trout et al. (2012)
Chinese muntjac	5 (3)	1993 - 2002	20 - 120	0.6 - 185	13 (990)	Cooke & Farrell (2001); Hemami et al. (2005); Hemami et al. (2007)
Orkney vole	4 (4)	1990 - 1990	0 - 27,500	18.5 - 191	14 (14)	Gorman & Reynolds (1993)
Rabbit	4 (4)	1986 - 2004	19.8 - 5,000	0.06 - 32.8	6 (2,539)	Flux et al. (1992); Forman (2005); Kolb (1991a); Kolb (1991b)
Serotine	4 (4)	1991 - 1991	0.16 - 0.59	24.1 - 77.2	5 (562)	Robinson & Stebbings (1997)
Skomer vole	4 (1)	1960 - 1992	2,414 - 7,426	2.9	2 (2)	Harris et al. (1995)
Fallow deer	3 (3)	1994 - 1994	38 - 46	3.7 - 10.2	5 (1,045)	Gill et al. (1997)
Feral cat	3 (3)	1977 - 1983	6 - 200	0.9- 1.8	4 (795)	Dards (1981;); Page et al. (1992)
Weasel	3 (3)	1973 - 2000	13 - 275	0.5 - 6	9 (1,896)	Graham (2002); Harris et al. (1995); Moors (1975)
Common rat	2 (2)	2002 - 2004	6.6 - 238	1.2 - 4.2	2 (1,888)	Baker et al. (2005); Forman (2005)
Daubenton's bat	2 (2)	1987 - 1990	1 - 2.4	100 - 275	20 (1,383)	Speakman et al. (1991); Jones et al. (1996)
Leisler's bat	2 (2)	1994 - 1994	4.4 - 6.7	13 - 30	6 (195)	Waters et al. (1999)
Water shrew	2 (2)	1990 - 2000	2.9 - 3.1	1.6 - 6.9	2 (1,082)	Kotzageorgis & Mason (1997); Shore et al. (2005)
Wildcat	2 (2)	1978 - 2010	0.3 - 0.68	15.6 - 118	9 (265)	Corbett (1979); Kilshaw et al. (2014)
Brandt's bat	1 (1)	1990 - 1990	1.7	100	8 (234)	Jones et al. (1996)
Chinese water deer	1 (1)	1991 - 1991	30	2.1	1 (144)	Harris et al. (1995)
Fat dormouse	1 (1)	2001 - 2001	235	0.58	1 (32)	Burgess et al. (2003)
Lesser white-toothed shrew	1 (1)	1997 - 1997	1,969	20.3	4 (4)	Battersby et al. (2005)
Noctule	1 (1)	1990 - 1990	1.5	200	8 (1,130)	Jones et al. (1996)
Otter	1 (1)	1988 - 1988	4.42	162.5	43 (2,351)	Kruuk et al. (1989)
Park cattle	1 (1)	1981 - 1981	36.57	1.34	1 (8)	Hall (1982)
Red-necked wallaby	1 (1)	1992 - 1992	57.8	0.45	1 (26)	Weir et al. (1995)
Ship rat	1 (1)	1996 - 1996	162	1.42	1 (86)	McDonald et al. (1997)
Whiskered bat	1 (1)	1990 - 1990	1.5	275	13 (619)	Jones et al. (1996)
Barbastelle	0 (0)	-	-	-	0 (281)	
Coypu	0 (0)	-	-	-	0 (193)	
Feral ferret	0 (0)	-	-	-	0 (353)	
Greater horseshoe bat	0 (0)	-	-	-	0 (282)	
Greater mouse-eared bat	0 (0)	-	-	-	0 (8)	
Grey long-eared bat	0 (0)	-	-	-	0 (38)	
Lesser horseshoe bat	0 (0)	-	-	-	0 (438)	
Nathusius' pipistrelle	0 (0)	-	-	-	0 (214)	
Stoat	0 (0)	-	-	-	0 (1,987)	

Table D. (also provided as a separate .xlsx file for enhance viewing; Table S1) Breakdown of density estimates for each species by land cover (LCM2007 target class). Values shown were derived from survey maps generated according to process outlined in Fig A. From top to bottom values denote estimates generated using a 25m, 1km and 10km dominant target land cover raster maps respectively. The median year of estimates across each land class is given in brackets. For the 25m estimates the total area surveyed in each land class is also provided. (*) denotes where there are no estimates but occurrence has been observed. (-) denotes at 25m where no estimates were available and additionally at other resolutions where no occurrence has been observed. Cells highlighted in grey show where no estimates are available at any resolution but occurrence has been recorded. Cells highlighted in black shown were no occurrence has been recorded. Using this table as a guide it may be possible to predict species density within any given survey site by combing estimates for land class in a weighted average according to the proportion of each habitat comprising the site. It should be noted that for some species estimates in aquatic land classes such as freshwater do not strictly apply to this environment but to habitats dominated by this land class.

	Land cover (Target class LCM 2007)										
Common name	1 (Broadleaved woodland)	2 (Coniferous woodland)	3 (Arable and horticulture)	4 (Improved grassland)	5 (Rough grassland)	6 (Neutral grassland)	7 (Calcareous grassland)	8 (Acid grassland)	9 (Fen, Marsh and Swamp)	10 (Heather)	11 (Heather grassland)
American mink	9.9 (1.3,1980)<br>0.5 - 9.9 (2000)<br>*	11 (0.1,2000)<br>0.5 - 10 (2000)<br>*	24 (1,2005)<br>1 - 26 (2005)<br>0.1 - 19 (2005)	27 (1.4,2005)<br>1.5 - 26 (2005)<br>0.1 - 11 (2000)	21 (0.6,2003)<br>2.4 - 44 (2003)<br>0.1 - 67 (2003)	29 (0.3,2005)<br>*<br>-	-<br>*<br>*	1.6 (0.003,1980)<br>*<br>*	1.6 (0.3,1980)<br>*<br>-	5.3 (0.3,2003)<br>*<br>*	7.9 (1.2,2003)<br>0.7 - 20 (2003)<br>0.1 - 43 (2003)
Badger	3.4 (336,2006)<br>3.2 - 4.9 (2006)<br>*	2.9 (94,2006)<br>1.7 - 3 (2006)<br>0.1 - 3 (2006)	2.3 (1767,2006)<br>1.7 - 2.4 (2006)<br>0.5 - 3 (2006)	3 (2004,2006)<br>2.2 - 3.2 (2006)<br>0.7 - 3.6 (2006)	3 (281,2006)<br>2.3 - 3.4 (2006)<br>*	2.6 (22,2006)<br>*<br>-	4.6 (1.3,2006)<br>*<br>*	2.5 (216,1996)<br>1.9 - 2.4 (1996)<br>0.3 - 2.7 (2006)	1.2 (0.7,1996)<br>*<br>-	3 (26,2006)<br>1.8 - 2.9 (2006)<br>*	2.6 (49,1996)<br>1.9 - 2.7 (2006)<br>*
Bank vole	326 (2.6,2002)<br>15 - 920 (2003)<br>*	178 (0.3,2000)<br>24 - 1605 (1987)<br>0.3 - 1605 (1987)	40 (12,1990)<br>19 - 534 (2000)<br>0.6 - 1069 (1994)	41 (44,2003)<br>18 - 211 (2003)<br>1.3 - 1367 (2003)	45 (1.8,2002)<br>0.3 - 21 (1995)<br>0.2 - 1235 (1994)	41 (0.1,2003)<br>*<br>-	41 (0.03,2003)<br>*<br>*	41 (0.2,2003)<br>*<br>*	-<br>*<br>-	33 (0.01,2003)<br>*<br>*	40 (0.1,2003)<br>*<br>*
Barbastelle	-<br>*<br>*	-<br>*<br>*	-<br>*<br>*	-<br>*<br>*	-<br>*<br>*		-<br>*<br>-	-<br>*<br>*		-<br>*<br>-	-<br>*<br>*
Bechsteins bat	329 (0.2,2011)<br>*<br>*	-<br>*<br>*	329 (0.02,0)<br>35 - 329 (2011)<br>*	329 (0.04,2011)<br>8.9 - 329 (2011)<br>0.9 - 329 (2011)	329 (0.02,2011)<br>*<br>-	329 (0.004,2011)<br>-<br>-	-<br>-<br>*	-<br>-<br>*			
Brandts bat	1.7 (9.2,1990)<br>1.3 - 1.7 (1990)<br>*	1.7 (2,1990)<br>0.7 - 1.7 (1990)<br>*	1.7 (21,1990)<br>1.1 - 1.7 (1990)<br>0.2 - 1.7 (1990)	1.7 (47,1990)<br>1.1 - 1.7 (1990)<br>0.3 - 1.7 (1990)	1.7 (5.2,1990)<br>*<br>*	1.7 (0.6,1990)<br>-<br>-	-<br>*<br>*	1.7 (3.5,1990)<br>0.4 - 1.7 (1990)<br>*		1.7 (6.7,1990)<br>1.2 - 1.7 (1990)<br>-	1.7 (3.5,1990)<br>0.6 - 1.7 (1990)<br>*
Brown hare	10 (421,2006)<br>6.7 - 8.3 (2006)<br>*	10 (123,1997)<br>7.3 - 8.8 (2006)<br>0.2 - 3.9 (2006)	16 (2774,1997)<br>14 - 15 (1997)<br>3.7 - 9.7 (2006)	6.2 (2267,2006)<br>2.8 - 3.6 (2006)<br>0.4 - 2.7 (2006)	7 (325,2006)<br>1.5 - 2 (2006)<br>*	19 (40,1997)<br>*<br>-	12 (6.3,1984)<br>9 - 14 (1984)<br>0.1 - 16 (1990)	2.1 (221,1997)<br>1.5 - 2 (1997)<br>0.2 - 2.1 (2006)	13.8 (0.7,1997)<br>*<br>-	3.3 (26,2006)<br>2.4 - 4 (2006)<br>*	2.6 (50,1997)<br>2.1 - 3.2 (2006)<br>*
Brown long-eared bat	1.5 (64.91,1990)<br>0.7 - 1.7 (1990)<br>*	1.7 (155,1987)<br>1.4 - 1.8 (1987)<br>0.8 - 1.7 (1987)	1.4 (293,1990)<br>1.1 - 1.4 (1990)<br>0.3 - 1.5 (1990)	1.5 (379,1990)<br>1.1 - 1.5 (1990)<br>0.4 - 1.7 (1990)	1.5 (44,1990)<br>1.2 - 1.7 (1987)<br>*	1.4 (9.2,1990)<br>-<br>-	-<br>*<br>*	1.6 (24,1987)<br>1.2 - 1.6 (1987)<br>*	2.7 (0.3,2009)<br>*<br>-	1.6 (84,1987)<br>1.1 - 1.6 (1987)<br>0.3 - 1.6 (1987)	1.7 (57,1987)<br>1.1 - 1.7 (1987)<br>0.3 - 1.7 (1987)
Chinese muntjac	63 (25,2002)<br>39 - 62 (2002)<br>*	64 (125,2002)<br>54 - 64 (2002)<br>36 - 64 (2002)	64 (9.2,2002)<br>14 - 63 (2002)<br>6.1 - 57 (2002)	64 (7.2,2002)<br>14 - 64 (2002)<br>*	64 (11,2002)<br>26 - 64 (2002)<br>12 - 64 (2002)	64 (0.7,2002)<br>*<br>-	64 (0.005,2002)<br>*<br>*	-<br>*<br>*	64 (0.004,2002)<br>*<br>-	64 (0.2,2002)<br>*<br>*	64 (0.2,2002)<br>*<br>*
Chinese water deer	30 (0.8,1991)<br>*<br>*	-<br>*<br>*	30 (0.8,1991)<br>11 - 30 (1991)<br>0.7 - 30 (1991)	30 (0.2,1991)<br>*<br>*	-<br>*<br>*	30 (0.01,1991)<br>-<br>-			30 (0.4,1991)<br>*<br>-	-<br>*<br>-	30 (0.05,1991)<br>-<br>-
Common dormouse	-<br>*<br>*	235 (0.2,2007)<br>37 - 235 (2007)<br>*	-<br>*<br>0.4 - 235 (2007)	-<br>*<br>*	-<br>*<br>-			-<br>*<br>*	-<br>*<br>-	-<br>*<br>-	-<br>*<br>-
Common rat	123 (0.5,2002)<br>10 - 122 (2003)<br>*	195 (0.03,2002)<br>*<br>*	209 (0.3,2002)<br>43 - 238 (2002)<br>*	222 (0.6,2002)<br>16 - 238 (2002)<br>0.1 - 6.6 (2004)	156 (0.2,2002)<br>*<br>*	-<br>*<br>-	-<br>*<br>*	-<br>*<br>*	-<br>*<br>-	-<br>*<br>*	-<br>*<br>*
Common shrew	310 (2.8,2004)<br>66 - 340 (2003)<br>0.1 - 1309 (1986)	786 (1.3,2004)<br>201 - 696 (2005)<br>*	79 (13,1990)<br>25 - 139 (2003)<br>0.1 - 3.7 (1997)	134 (49,2003)<br>76 - 237 (2003)<br>16 - 570 (2003)	633 (3.2,2005)<br>48 - 716 (2005)<br>*	3.8 (0.2,1972)<br>*<br>-	5.8 (0.03,0)<br>*<br>*	582 (1.4,2005)<br>134 - 700 (2005)<br>3 - 1067 (2005)	-<br>*<br>-	250 (0.4,2004)<br>*<br>*	372 (1.8,2005)<br>56 - 353 (2004)<br>9.9 - 509 (2005)
Coypu	-<br>*<br>*	-<br>*<br>*	-<br>*<br>*	-<br>*<br>*	-<br>*<br>*				-<br>*<br>-		
Daubentons bat	1.3 (26,1990)<br>0.9 - 1.4 (1990)<br>*	2.2 (37,1987)<br>1.5 - 2.3 (1987)<br>0.6 - 2.4 (1987)	1.1 (109,1990)<br>0.8 - 1.1 (1990)<br>0.2 - 1 (1990)	1.2 (111,1990)<br>0.8 - 1.2 (1990)<br>0.3 - 1.2 (1990)	1.3 (12,1990)<br>1.1 - 2.4 (1987)<br>*	1 (3.6,1990)<br>*<br>-	-<br>*<br>-	1.9 (4.4,1987)<br>0.4 - 1.9 (1987)<br>*	2.4 (0.01,1987)<br>*<br>-	2.3 (39,1987)<br>1.6 - 2.3 (1987)<br>0.3 - 2.4 (1987)	2.2 (13,1987)<br>1.3 - 2.2 (1987)<br>0.1 - 2.4 (1987)
Fallow deer	41 (12,1994)<br>27 - 41 (1994)<br>*	40 (14,1994)<br>32 - 41 (1994)<br>*	46 (1.8,1994)<br>6.8 - 43 (1994)<br>3.4 - 42 (1994)	46 (4.2,1994)<br>11 - 44 (1994)<br>0.3 - 45 (1994)	45 (0.5,1994)<br>*<br>*	-<br>*<br>-	-<br>*<br>-	46 (0.3,1994)<br>*<br>*	-<br>*<br>-	43 (0.1,1994)<br>*<br>*	46 (0.3,1994)<br>*<br>*
Fat dormouse	235 (0.73,2001)<br>166 - 235 (2001)<br>-	235 (0.14,2001)<br>-<br>-	235 (0.056,0)<br>15 - 235 (2001)<br>2.3 - 235 (2001)	235 (0.052,0)<br>17 - 235 (2001)<br>*	235 (0.002,0)<br>*<br>-						
Feral cat	13 (0.004,1983)<br>*<br>*	-<br>*<br>*	13 (0.01,1983)<br>*<br>*	6 (0.01,1977)<br>*<br>0 - 13 (1983)	6 (0.8,1977)<br>6 (1977)<br>-			-<br>*<br>*		-<br>*<br>*	-<br>*<br>*
Feral ferret	-<br>*<br>-	-<br>*<br>*	-<br>*<br>*	-<br>*<br>*	-<br>*<br>*			-<br>*<br>*		-<br>*<br>*	-<br>*<br>*
Feral goat	2.5 (4.9,1979)<br>-<br>*	3.3 (7.4,1979)<br>2.6 - 7.7 (1979)<br>0.4 - 8.2 (1979)	2.2 (0.3,1979)<br>-<br>*	2.1 (2.1,1979)<br>0.2 - 1.5 (1979)<br>*	2.4 (42,1978)<br>1.8 - 2.6 (1978)<br>0.9 - 2.2 (1978)	2 (0.1,1978)<br>-<br>-		2.1 (69,1979)<br>1.2 - 2.1 (1979)<br>0.6 - 2.5 (1979)		3.8 (32,1979)<br>2.8 - 3.4 (1979)<br>*	3.3 (50,1979)<br>2.9 - 4 (1979)<br>*
Feral sheep	105 (2,1979)<br>*<br>*	76 (3.3,1979)<br>10 - 40 (1979)<br>5.3 - 55 (1979)	101 (0.23,1979)<br>-<br>*	101 (1.4,1979)<br>11 - 105 (1979)<br>*	78 (3.2,1979)<br>-<br>*			99 (37,1979)<br>63 - 99 (1979)<br>19 - 105 (1979)		728 (12,1979)<br>70 - 87 (1979)<br>*	78 (18,1979)<br>58 - 69 (1979)<br>*
Field vole	576 (2.4,2003)<br>286 - 452 (2003)<br>*	5260 (17,1997)<br>758 - 5812 (1997)<br>187 - 7476 (1997)	34 (12,1990)<br>11 - 541 (2000)<br>0.2 - 8.5 (1999)	20 (44,2003)<br>5.6 - 236 (2003)<br>0.5 - 14 (2003)	1929 (3,1997)<br>233 - 17234)<br>*	9 (0.1,2003)<br>*<br>-	8.83 (0.03,2003)<br>*<br>*	3081 (5.7,1997)<br>621 - 5895<br>*	-<br>*<br>-	4039 (1.3,1997)<br>349 - 3048<br>*	3388 (8.5,1997)<br>730 - 3048 (1997)<br>*
Greater horseshoe bat	-<br>*<br>-	-<br>*<br>*	-<br>*<br>*	-<br>*<br>*	-<br>*<br>*		-<br>*<br>*	-<br>*<br>*		-<br>*<br>-	-<br>*<br>*
Greater mouse-eared bat	-<br>*<br>-		-<br>*<br>*	-<br>*<br>*							
Grey long-eared bat	-<br>*<br>-		-<br>*<br>*	-<br>*<br>*	-<br>*<br>-						
Grey squirrel	70 (6.4,1998)<br>28 - 107 (1997)<br>*	80 (118,1997)<br>59 - 80 (1997)<br>22 - 45 (1999)	90 (2.2,1998)<br>18 - 89 (1997)<br>9.1 - 99 (1997)	81 (6,1997)<br>11 - 82 (1997)<br>3.1 - 81 (1998)	82 (9,1997)<br>31 - 82 (1997)<br>11 - 82 (1997)	87 (0.6,1997)<br>*<br>-	82 (0.01,1997)<br>*<br>*	26 (0.3,1998)<br>4.3 - 20 (1998)<br>*	169.3 (0.03,0)<br>*<br>-	83 (0.6,1997)<br>6.3 - 26 (1998)<br>*	146 (0.6,1997)<br>4.2 - 26 (1998)<br>*
Harvest mouse	2.7 (0.2,1996)<br>*<br>*	-<br>*<br>*	3.3 (8.5,1990)<br>1.6 - 3.2 (1990)<br>0.1 - 2.8 (1996)	3.2 (0.4,1990)<br>0.3 - 3 (1994)<br>0 - 2.5 (1996)	3 (0.1,1990)<br>*<br>*	2.6 (0.001,1996)<br>*<br>-	-<br>*<br>*		-<br>*<br>-	-<br>*<br>-	-<br>*<br>-
Hedgehog	34 (18,2002)<br>13 - 51 (2002)<br>*	27.1 (2.65,2002)<br>4.4 - 22 (2002)<br>*	49 (38,2002)<br>15 - 58 (2002)<br>5.3 - 66 (2002)	35 (736,2002)<br>20 - 33 (2002)<br>6.7 - 29 (2002)	43 (16,2002)<br>21 - 76 (2002)<br>*	66 (8.4,2002)<br>*<br>*	31 (0.4,2002)<br>0.1 - 45 (2002)<br>*	25 (7.4,2002)<br>5.8 - 25 (2002)<br>0.2 - 67 (1989)	-<br>*<br>-	27 (0.5,1989)<br>*<br>*	30 (1.7,2002)<br>2.9 - 21 (2002)<br>*
House mouse	68 (0.1,1996)<br>*<br>*	-<br>*<br>*	0 (1.5,1996)<br>1.2 - 3.1 (1996)<br>0 - 3.5 (1997)	23 (0.1,1997)<br>*<br>0 - 4.7 (1996)	680 (0.8,1967)<br>669 - 700 (1967)<br>44 - 700 (1967)	0 (0.001,1997)<br>-<br>-	-<br>*<br>*	-<br>*<br>*	700 (0.01,1967)<br>*<br>-	-<br>*<br>*	-<br>*<br>*

	Land cover (Target class LCM 2007)											
Common name	12 (Bog)	13 (Montane habitats)	14 (Inland rock)	15 (Saltwater)	16 (Freshwater)	17 (Supra-littoral rock)	18 (Supra-littoral sediment)	19 (Littoral rock)	20 (Littoral sediment)	21 (Saltmarsh)	22 (Urban)	23 (Suburban)
American mink	8 (1.9,2003)<br>1.3 - 18 (2003)<br>0.4 - 32 (2003)	-<br>*<br>*	7.3 (0.9,2003)<br>0.5 - 5.5 (2003)<br>*	12 (0.04,2003)<br>*<br>*	5.3 (3.3,2003)<br>2.6 - 24 (2003)<br>*	72 (0.01,2003)<br>-<br>-	15 (0.1,1980)<br>0.02 - 5.8 (2003)<br>*	71 (1.4,2003)<br>6.6 - 71 (2003)<br>*	9.8 (0.2,1980)<br>0.1 - 5.6 (2003)<br>*	6.6 (0.04,1980)<br>*<br>-	65 (0.02,2004)<br>*<br>*	29 (0.1,1980)<br>4.6 - 65 (2004)<br>*
Badger	2.6 (3,1996)<br>1.5 - 2.8 ('06)<br>0.2 - 3.6 ('06)	-<br>*<br>*	2.3 (12,2006)<br>1.2 (1996)<br>-	2.8 (8.7,2006)<br>1.9 - 3.5 (2006)<br>*	1.9 (27,1996)<br>1.4 - 1.7 (1996)<br>*	4.8 (0.1,2006)<br>*<br>-	4.7 (5,2006)<br>3.9 - 4.8 (2006)<br>3.7 - 4.8 (2006)	2.5 (3.2,2006)<br>2 - 3.2 (2006)<br>*	2.5 (5.7,2006)<br>2.4 - 3.4 (2006)<br>0.2 - 2.8 (2006)	3.1 (0.5,2006)<br>*<br>-	2.6 (17,2006)<br>2 - 3.6 (2006)<br>*	2.6 (100,2006)<br>1.8 - 3.1 (2006)<br>0 - 2.9 (2006)
Bank vole	-<br>*<br>*	-<br>*<br>*	41 (0.004,2003)<br>*<br>-	238 (0.01,2002)<br>*<br>*	27 (0.2,2004)<br>5.6 - 370 (1986)<br>0.06 - 370 (1986)		-<br>*<br>*	-<br>*<br>-	7 (0.001,1987)<br>*<br>*	11 (0.7,2004)<br>1.7 - 6.6 (2004)<br>-	117 (0.1,2002)<br>*<br>*	223 (4.5,2002)<br>108 - 220 (2002)<br>7 - 2011 (2003)
Barbastelle	-<br>-<br>*		-<br>*<br>-	-<br>-<br>*	-<br>*<br>-		-<br>*<br>-		-<br>*<br>*		-<br>*<br>-	-<br>*<br>*
Bechsteins bat			-<br>*<br>-	-<br>*<br>*	-<br>*<br>-					-<br>*<br>-		329 (0.001,2011)<br>*<br>*
Brandts bat	1.7 (0.7,1990)<br>-<br>*	-<br>-<br>*	1.7 (0.04,1990)<br>*<br>-	-<br>*<br>*	1.7 (0.5,1990)<br>-<br>-		-<br>*<br>-		-<br>-<br>*		1.7 (0.8,1990)<br>*<br>-	1.7 (7.3,1990)<br>1.4 - 1.7 (1990)<br>*
Brown hare	2.2 (3.4,1997)<br>2.5 - 4.6 (2006)<br>0.1 - 3.7 (2006)	-<br>*<br>*	10 (14,1997)<br>15 (1997)<br>-	1.2 (8.9,2006)<br>0.4 - 1.8 (2006)<br>0.2 - 3.7 (1990)	14 (33,1997)<br>12 (1997)<br>*	0.5 (0.13,2006)<br>-<br>-	0.6 (5,2006)<br>0.5 - 1 (2006)<br>0.4 - 0.5 (2006)	5.3 (3.7,2006)<br>3 - 6.7 (2006)<br>-	2 (5.7,2006)<br>0.9 - 1.4 (2006)<br>0.3 - 4.6 (2006)	4.1 (1.2,2004)<br>1.7 - 6.6 (2004)<br>-	16 (28,1997)<br>11 - 12 (1997)<br>*	11 (130,2006)<br>5.6 - 8.2 (2006)<br>0 - 0.4 (2006)
Brown long-eared bat	1.6 (1.9,1987)<br>0.6 - 1.5 (1989)<br>*	1.7 (0.3,1987)<br>0.02 - 1.7 (1987)<br>*	1.7 (1.4,1990)<br>*<br>-	-<br>*<br>*	1.5 (8.2,1990)<br>*<br>*		-<br>*<br>-		-<br>*<br>*	-<br>*<br>-	1.5 (5.4,1990)<br>0 - 1.4 (1990)<br>*	1.5 (29,1990)<br>1.1 - 1.5 (1990)<br>*
Chinese muntjac	-<br>*<br>*		64 (0.03,2002)<br>*<br>-	-<br>*<br>*	64 (0.2,2002)<br>*<br>-		-<br>*<br>-		-<br>*<br>*	-<br>*<br>-	64 (0.2,2002)<br>*<br>*	64 (1.4,2002)<br>21 - 64 (2002)<br>*
Chinese water deer				-<br>*<br>-	30 (0.03,1991)<br>*<br>-		-<br>*<br>-		-<br>*<br>-	-<br>*<br>-		30 (0.003,1991)<br>*<br>*
Common dormouse	-<br>*<br>*		-<br>*<br>-	-<br>*<br>-	-<br>*<br>-			-<br>*<br>-	-<br>*<br>*	-<br>*<br>-	-<br>*<br>-	-<br>*<br>*
Common rat	-<br>*<br>*	-<br>*<br>*	-<br>*<br>-	238 (0.01,2002)<br>*<br>*	24 (0.2,2004)<br>*<br>*	-<br>*<br>-	-<br>*<br>-	-<br>*<br>*	6.6 (0.001,0)<br>*<br>-	11.3 (0.71,2004)<br>1.7 - 6.6 (2004)<br>-	238 (0.03,'02)<br>*<br>*	236.6 (4.2,2002)<br>116 - 238 (2002)<br>13 - 238 (2002)
Common shrew	886 (0.1,2004)<br>87 - 850 (2004)<br>*	-<br>*<br>*	523 (0.1,2005)<br>*<br>*	1477 (0.01,2005)<br>*<br>*	489 (0.1,2004)<br>*<br>*	-<br>*<br>-	579 (0.01,2004)<br>*<br>*	398 (0.2,2004)<br>98 - 3800 (2005)<br>-	1306 (0.2,2004)<br>605 - 1500 (2004)<br>*	1382 (0.01,2002)<br>*<br>-	495 (0.1,2002)<br>*<br>*	1288 (4.55,2002)<br>615 - 1257 (2002)<br>75 - 1382 (2002)
Coypu				-<br>*<br>*	-<br>*<br>-		-<br>*<br>-		-<br>*<br>*		-<br>*<br>-	-<br>*<br>*
Daubentons bat	2.4 (0.07,1987)<br>*<br>*	2.4 (0.3,1987)<br>0.03 - 2.4 (1987)<br>*	1.2 (0.5,1990)<br>*<br>-	-<br>*<br>*	1.4 (4,1990)<br>*<br>*		-<br>*<br>*		-<br>*<br>*	-<br>*<br>-	1 (3.7,1990)<br>1 (1990)<br>*	1 (16,1990)<br>0.7 - 1.1 (1990)<br>*
Fallow deer	-<br>-<br>*	-<br>-<br>*	-<br>*<br>-	-<br>*<br>-	41 (0.02,1994)<br>*<br>*		-<br>*<br>-		-<br>*<br>*	-<br>*<br>-	40 (0.01,1994)<br>*<br>*	41 (0.03,1994)<br>4.6 - 41 (1994)<br>*
Fat dormouse												-<br>*<br>*
Feral cat	-<br>*<br>*		13 (0.01,1983)<br>-<br>-	103 (0.1,1979)<br>30 - 153 (1979)<br>*	34 (0.2,1977)<br>*<br>-	6 (0.07,1977)<br>-<br>-	6 (2.8,1977)<br>5.6 - 6 (1977)<br>-	6.2 (0.7,1977)<br>5.7 - 6 (1977)<br>*	16 (0.4,1977)<br>*<br>*	-<br>*<br>-	111 (1.8,1979)<br>14 - 75 (1983)<br>*	110 (0.3,1979)<br>*<br>1.2 - 106 (1981)
Feral ferret	-<br>*<br>*	-<br>-<br>*		-<br>*<br>*	-<br>*<br>*		-<br>*<br>*		-<br>*<br>*		-<br>*<br>-	-<br>*<br>*
Feral goat	2.8 (24,1979)<br>1.7 - 2.8 (1979)<br>*	8.3 (1.9,1979)<br>10 - 11 (1979)<br>*	1.5 (14,1978)<br>-<br>-	1.3 (0.1,1978)<br>*<br>*	2 (1.1,1978)<br>0.02 - 0.9 (1991)<br>*	1.3 (0.01,1978)<br>*<br>-	1.3 (0.1,1978)<br>-<br>-	1.3 (2.9,1978)<br>1.3 (1978)<br>-	1.3 (0.3,1978)<br>-<br>*	1.3 (0.1,1978)<br>-<br>-	4 (0.1,1991)<br>-<br>-	2.1 (0.05,1991)<br>-<br>-
Feral sheep	96 (21,1979)<br>-<br>*	24 (1.3,1979)<br>21 - 24 (1979)<br>*	105 (0.1,1979)<br>-<br>-		-<br>*<br>-		-<br>*<br>-		-<br>-<br>*		105 (0.01,1979)<br>-<br>-	
Field vole	3162 (6,1997)<br>583 - 3652<br>*	-<br>*<br>*	3022 (0.03,1997)<br>*<br>*	-<br>*<br>*	88 (0.2,2004)<br>185 - 3048 (1997)<br>*	3048 (0.01,1997)<br>*<br>-	-<br>*<br>*	-<br>*<br>*	69 (0.001,2004)<br>*<br>*	69 (0.7,2004)<br>17 - 69 (2004)<br>-	1625 (0.1,1997)<br>*<br>*	397 (0.4,2000)<br>3.9 - 8.8 (2003)<br>*
Greater horseshoe bat	-<br>*<br>*			-<br>*<br>-			-<br>*<br>-	-<br>*<br>-	-<br>*<br>*	-<br>*<br>-	-<br>*<br>-	-<br>*<br>*
Greater mouse-eared bat											-<br>*<br>-	-<br>*<br>-
Grey long-eared bat				-<br>*<br>*							-<br>*<br>-	-<br>*<br>*
Grey squirrel	26 (0.04,1998)<br>5 - 26 (1998)<br>*	-<br>*<br>*	124 (0.1,1997)<br>*<br>-	-<br>*<br>*	76 (0.3,1997)<br>*<br>*		162 (0.03,1990)<br>*<br>-	-<br>*<br>*	-<br>*<br>*	-<br>*<br>-	79 (0.1,1997)<br>*<br>*	94 (0.7,1997)<br>21 - 88 (1997)<br>*
Harvest mouse	-<br>*<br>*		-<br>*<br>-	-<br>*<br>*	3.5 (0.02,1990)<br>*<br>-		-<br>*<br>*		-<br>*<br>*	-<br>*<br>-	3.5 (0.02,1990)<br>*<br>*	3.5 (0.03,1990)<br>*<br>*
Hedgehog	-<br>*<br>*	-<br>*<br>*	56 (0.2,2002)<br>25 - 90 (2002)<br>*	-<br>*<br>*	43 (0.4,2002)<br>12 - 74 (1979)<br>*		-<br>*<br>*	-<br>*<br>*	-<br>*<br>*	-<br>*<br>-	40 (1.1,2002)<br>9 - 29 (2002)<br>*	50 (4.7,2002)<br>11 - 66 (2002)<br>0.5 - 100 (1979)
House mouse	-<br>*<br>*	-<br>-<br>*		700 (0.01,1967)<br>*<br>*			-<br>*<br>-	-<br>-<br>*	700 (0.03,1967)<br>*<br>*	-<br>*<br>-	-<br>*<br>-	600 (0.009,1967)<br>*<br>*

	Land cover (Target class LCM 2007)																						
**Common name**	**1 (Broadleaved woodland)**	**2 (Coniferous woodland)**	**3 (Arable and horticulture)**	**4 (Improved grassland)**	**5 (Rough grassland)**	**6 (Neutral grassland)**	**7 (Calcareous grassland)**	**8 (Acid grassland)**	**9 (Fen, Marsh and Swamp)**	**10 (Heather)**	**11 (Heather grassland)**	**12 (Bog)**	**13 (Montane habitats)**	**14 (Inland rock)**	**15 (Saltwater)**	**16 (Freshwater)**	**17 (Supra-littoral rock)**	**18 (Supra-littoral sediment)**	**19 (Littoral rock)**	**20 (Littoral sediment)**	**21 (Saltmarsh)**	**22 (Urban)**	**23 (Suburban)**
**Leislers bat**	5 (9.1,1994)<br>4 - 5.1 (1994)<br>*	6.3 (0.4,1994)<br>*<br>*	5.7 (8.9,1994)<br>2.4 - 5.5 (1994)<br>0.2 - 4.4 (1994)	6 (11,1994)<br>4.4 - 5.9 (1994)<br>0.4 - 6.7 (1994)	6.2 (1.1,1994)<br>*<br>-	6.7 (0.01,1994)<br>-<br>-		6.7 (0.04,1994)<br>-<br>*	-<br>*<br>-	-<br>-<br>*	-<br>*<br>*	-<br>-<br>*			6.7 (0.3,1994)<br>-<br>-	6.6 (0.6,1994)<br>*<br>-		6.7 (0.01,1994)<br>-<br>-	6.7 (0.1,1994)<br>-<br>-	6.7 (0.01,1994)<br>-<br>*	6.7 (0.2,1994)<br>-<br>-	6.3 (1.7,1994)<br>*<br>*	6.5 (10,1994)<br>4.2 - 6.7 (1994)<br>0.8 - 5.5 (1994)
**Lesser horseshoe bat**	-<br>*<br>-	-<br>*<br>*	-<br>*<br>*	-<br>*<br>*	-<br>*<br>*	-<br>*<br>-	-<br>-<br>*	-<br>*<br>*		-<br>*<br>-	-<br>*<br>*	-<br>*<br>*		-<br>*<br>-	-<br>*<br>*	-<br>*<br>-		-<br>*<br>-		-<br>*<br>*	-<br>*<br>-	-<br>*<br>-	-<br>*<br>*
**Lesser white-toothed shrew**	1969 (1,1997)<br>-<br>-	1969 (0.2,1997)<br>-<br>-	1969 (4.7,1997)<br>1935 - 1969 (1997)<br>*	1969 (1.3,1997)<br>-<br>-	1969 (0.7,1997)<br>-<br>-			1969 (0.02,1997)<br>-<br>-		1969 (0.7,1997)<br>-<br>-	1969 (4.3,1997)<br>-<br>*			1969 (0.1,1997)<br>-<br>-	1969 (0.2,1997)<br>-<br>-	1969 (0.2,1997)<br>-<br>-		1969 (0.4,1997)<br>-<br>-	1969 (4,1997)<br>1380 - 1970<br>*	1969 (1.9,1997)<br>-<br>-	1969 (0.1,1997)<br>-<br>-	1969 (0.2,1997)<br>-<br>-	1969 (0.5,1997)<br>-<br>-
**Mole**	515 (0.01,1983)<br>*<br>*	500 (0.01,1983)<br>67 - 518 (1983)<br>*	494 (0.2,1982)<br>30 - 531 (1984)<br>0.7 - 585 (1987)	594 (0.2,1983)<br>26 - 556 (1983)<br>*	550 (0.02,1983)<br>*<br>*	600 (0.1,1990)<br>*<br>-	-<br>*<br>*	-<br>*<br>*	-<br>*<br>-	-<br>*<br>*	-<br>*<br>*	-<br>*<br>*	-<br>*<br>*	-<br>*<br>-	-<br>*<br>*	510 (0.001,1983)<br>*<br>*	-<br>*<br>-	-<br>*<br>*	-<br>*<br>*	-<br>*<br>*	-<br>*<br>-	-<br>*<br>*	-<br>*<br>*
**Mountain hare**	-<br>*<br>-	6.3 (2.3,1971)<br>2.3 - 6.3 (1971)<br>0.1 - 6.3 (1971)	6.3 (0.2,1971)<br>*<br>*	6.3 (0.1,1971)<br>*<br>*	6.3 (0.6,1971)<br>2.5 - 6.3 (1971)<br>*			4.1 (0.01,1961)<br>0.04 - 3.8 (1961)<br>*		6.5 (0.7,1971)<br>2 - 6.3 (1971)<br>0 - 58 (1971)	6.4 (1.6,1971)<br>0.9 - 26 (1971)<br>0 - 31 (1961)	-<br>*<br>*	6 (0.002,1961)<br>0.01 - 6 (1961)<br>0 - 14 (1961)	-<br>*<br>*	-<br>-<br>*	-<br>*<br>*							-<br>*<br>*
**Nathusius pipistrelle**	-<br>*<br>*	-<br>*<br>*	-<br>*<br>*	-<br>*<br>*	-<br>*<br>*			-<br>-<br>*	-<br>*<br>-	-<br>-<br>*	-<br>*<br>*	-<br>-<br>*		-<br>*<br>-	-<br>*<br>-	-<br>*<br>-			-<br>*<br>-	-<br>*<br>*	-<br>*<br>-	-<br>*<br>*	-<br>*<br>*
**Natterers bat**	3.3 (15,1990)<br>2.9 - 10 (1990)<br>*	19 (15,2009)<br>16 - 22 (2009)<br>*	2.2 (42,1990)<br>1.3 - 2.9 (1990)<br>0.8 - 7.4 (1990)	1.9 (67,1990)<br>1.3 - 3.2 (1990)<br>0.5 - 10 (1990)	3.1 (7.6,1990)<br>*<br>*	2.6 (2.4,1990)<br>*<br>-	-<br>*<br>*	1.8 (1.1,1990)<br>*<br>*	24 (0.3,2009)<br>*<br>-	9.4 (2.2,1990)<br>0.6 - 1.8 (1990)<br>*	17 (3.4,2009)<br>2.5 - 24 (2009)<br>*	-<br>*<br>*	-<br>*<br>*	17 (0.3,2009)<br>-<br>-	-<br>*<br>*	1.8 (1.6,1990)<br>*<br>*		-<br>*<br>-		-<br>*<br>*	-<br>*<br>-	1.8 (1.3,1990)<br>*<br>*	2.4 (8.4,1990)<br>1.5 - 3.7 (1990)<br>*
**Noctule**	1.5 (11,1990)<br>1 - 1.5 (1990)<br>*	1.5 (2.7,1990)<br>*<br>*	1.5 (55,1990)<br>1 - 1.5 (1990)<br>0.2 - 1.5 (1990)	1.5 (55,1990)<br>0.9 - 1.5 (1990)<br>0.4 - 1.5 (1990)	1.5 (5.5,1990)<br>*<br>*	1.5 (2.2,1990)<br>*<br>-	-<br>*<br>*	-<br>*<br>*	-<br>*<br>-	1.5 (0.2,1990)<br>*<br>*	1.5 (0.1,1990)<br>*<br>*	-<br>*<br>*	-<br>-<br>*	1.5 (0.3,1990)<br>*<br>-	-<br>*<br>*	1.5 (1.6,1990)<br>*<br>-	-<br>*<br>-	-<br>*<br>-		-<br>*<br>*	-<br>*<br>-	1.5 (3.1,1990)<br>1.5 (1990)<br>*	1.5 (14,1990)<br>1.2 - 1.5 (1990)<br>*
**Orkney vole**		12396 (0.4,1990)<br>-<br>-	39 (18,1990)<br>-<br>-	47 (191,1990)<br>8527 - 9112<br>*	27427 (164,1990)<br>-<br>-			27408 (1.3,1990)<br>-<br>-	11486 (1,1990)<br>-<br>-	6263 (9,1990)<br>-<br>-	6273 (42,1990)<br>-<br>-	6252 (49,1990)<br>-<br>-		13074 (0.1,1990)<br>-<br>-	12132 (1.6,1990)<br>-<br>-	12866 (1.9,1990)<br>-<br>-	18563 (0.03,1990)<br>-<br>-	11192 (0.8,1990)<br>-<br>-	11647 (0.6,1990)<br>-<br>-	12481 (0.2,1990)<br>-<br>-	13750 (0.004)<br>-<br>-	11005 (1.2)<br>-<br>-	11368 (1.1,1990)<br>-<br>-
**Otter**	-<br>*<br>*	-<br>*<br>*	4.4 (1.2,1988)<br>*<br>*	4.4 (6.7,1988)<br>0.9 - 4.4 (1988)<br>*	4.4 (65,1988)<br>1.7 - 4.4 (1988)<br>1.5 - 4.4 (1988)	4.4 (1.9,1988)<br>1.4 - 4.4 (1988)<br>0.7 - 4.4 (1988)	4.4 (0.02,1988)<br>*<br>*	4.4 (2.4,1988)<br>1.1 - 4.4 (1988)<br>*	4.4 (0.1,1988)<br>*<br>-	4.4 (8.9,1988)<br>1.6 - 4.4 (1988)<br>0.3 - 4.4 (1988)	4.4 (34,1988)<br>1.6 - 4.4 (1988)<br>1.2 - 4.4 (1988)	4.4 (8,1988)<br>1 - 4.4 (1988)<br>0.5 - 4.4 (1988)	-<br>*<br>*	4.4 (0.3,1988)<br>2.5 - 4.4<br>*	4.4 (6.3,1988)<br>2 - 4.4 (1988)<br>*	4.4 (1.3,1988)<br>*<br>*	4.4 (27,1988)<br>3.9 - 4.4 (1988)<br>-	4.4 (1.3,1988)<br>2.5 - 4.4 (1988)<br>*	4.4 (11,1988)<br>4 - 4.4 (1988)<br>*	4.4 (4.5,1988)<br>3.5 - 4.4 ('88)<br>*	4.4 (0.6,1988)<br>*<br>-	4.4 (1.4,1988)<br>2.1 - 4.4 (1988)<br>*	4.4 (2.3,1988)<br>2.5 - 4.4 (1988)<br>*
**Park cattle**	37 (0.4,1981)<br>27 - 37 (1981)<br>-	37 (0.01,1981)<br>-<br>-	-<br>*<br>*	37 (0.3,1981)<br>2.9 - 37 (1981)<br>*	37 (0.4,1981)<br>-<br>-	37 (0.03,1981)<br>-<br>-		37 (0.1,1981)<br>-<br>-		37 (0.1,1981)<br>-<br>-	37 (0.1,1981)<br>-<br>-												37 (0.02,1981)<br>-<br>-
**Pine marten**	0.4 (82,1998)<br>0.3 - 0.4 (1998)<br>*	0.4 (517,1998)<br>0.3 - 0.4 (1998)<br>0.1 - 0.4 (1998)	0.3 (66,1998)<br>0.2 - 0.3 (1998)<br>0.1 - 0.2 (1998)	0.4 (178,1998)<br>0.3 - 0.4 (1998)<br>0.1 - 0.3 (1998)	0.4 (83,1998)<br>0.3 - 0.4 (1998)<br>*	0.2 (0.1,1998)<br>-<br>-		0.4 (83,1998)<br>0.2 - 0.4 (1998)<br>0.1 - 0.4 (1998)	0.4 (0.3,1998)<br>*<br>-	0.4 (984,1998)<br>0.3 - 0.4 (1998)<br>0.1 - 0.4 (1998)	0.4 (105,1998)<br>0.2 - 0.4 (1998)<br>0.1 - 0.4 (1989)	0.4 (37,1998)<br>0.2 - 0.4 (1998)<br>*	0.5 (1.6,1989)<br>0.2 - 0.5 (1998)<br>0.1 - 0.6 (1998)	0.5 (3.5,1998)<br>0.36 - 0.46<br>-	0.2 (5.2,1998)<br>0.1 - 0.4 (1998)<br>0.1 - 0.5 (1998)	0.4 (37,1998)<br>0.2 - 0.4 (1998)<br>*		0.4 (0.03,1998)<br>*<br>-	0.25 (0.67,'98)<br>0.1 - 0.3 ('98)<br>-	0.24 (0.13,'98)<br>*<br>*	0.26 (0.27,1998)<br>-<br>-	0.33 (1.66,'98)<br>*<br>-	0.36 (5.48,1998)<br>0.1 - 0.2 (1998)<br>*
**Pipistrelle**	15 (101,1990)<br>9.4 - 15 (1987)<br>*	17 (228,1987)<br>13 - 17 (1987)<br>8.9 - 18 (1987)	15 (455,1990)<br>11 - 14 (1990)<br>4.5 - 13 (1990)	15 (575,1990)<br>12 - 15 (1990)<br>5.1 - 14 (1990)	16 (72,1987)<br>12 - 18 (1987)<br>*	13 (9.8,1990)<br>*<br>-	-<br>*<br>*	17 (24,1987)<br>12 - 17 (1987)<br>*	8.1 (0.6,2009)<br>*<br>-	17 (109,1987)<br>12 - 17 (1987)<br>3.6 - 18 (1987)	17 (67,1987)<br>11 - 17 (1987)<br>2.7 - 18 (1987)	16 (3.8,1987)<br>4.6 - 17 (1987)<br>*	18 (0.3,1987)<br>-<br>*	15 (2.6,1987)<br>*<br>*	18 (0.5,1987)<br>*<br>*	16 (13,1987)<br>8.5 - 14 (1990)<br>*	18 (0.1,1987)<br>-<br>-	-<br>*<br>*	18.2 (0.03,'87)<br>*<br>*	18.2 (0.08,'87)<br>*<br>*	-<br>*<br>-	15.7 (15.8,'87)<br>11 - 17 (1987)<br>*	15.53 (77.1,1987)<br>13 - 16 (1987)<br>9.3 - 18 (1987)
**Polecat**	0.8 (2830,1996)<br>0.7 (1996)<br>1.3 (1996)	1.1 (1933,1996)<br>1.1 (1996)<br>0.8 - 1.2 (1996)	0.6 (11333,1996)<br>0.5 (1996)<br>0.4 - 0.5 (1996)	0.9 (16122,1996)<br>1 (1996)<br>1 - 1.1 (1996)	0.9 (3212,1996)<br>1.1 (1996)<br>1 - 1.4 (1996)	0.8 (264,1996)<br>1.1 (1996)<br>-	0.3 (8.7,1996)<br>0.2 (1996)<br>*	1.4 (3554,1996)<br>1.4 (1996)<br>1.3 - 1.4 (1996)	1 (4.4,1996)<br>-<br>-	1.3 (473,1996)<br>1.4 - 1.5 (1996)<br>-	1.4 (1015,1996)<br>1.4 (1996)<br>0.5 - 1.4 (1996)	1.4 (635,1996)<br>1.3 - 1.4 (1996)<br>0.6 - 1.4 (1996)	1.4 (256,1996)<br>-<br>1.2 - 1.3 (1996)	1 (180,1996)<br>-<br>-	0.8 (128,1996)<br>0.7 - 0.9 (1996)<br>1 - 1.3 (1996)	1 (278,1996)<br>1.1 (1996)<br>-	1.1 (5.6,1996)<br>-<br>-	0.1 (61,1996)<br>0.6 (1996)<br>-	0.6 (28,1996)<br>0.8 (1996)<br>-	1 (359,1996)<br>0.9 - 1 (1996)<br>0.9 - 1.2 (1996)	0.9 (98,1996)<br>0.9 - 1 (1996)<br>-	0.6 (551,1996)<br>0.5 (1996)<br>*	0.5 (2282,1996)<br>0.5 (1996)<br>0.2 - 0.3 (1996)
**Pygmy shrew**	14 (0.4,1997)<br>2 - 218 (1972)<br>0.1 - 1309 (1986)	3.1 (0.1,2000)<br>*<br>*	2.9 (9.9,1990)<br>1.5 - 3.1 (1990)<br>0.1 - 3.1 (1996)	15 (0.8,1972)<br>0.3 - 2.5 (1994)<br>0 - 5.5 (1996)	8.7 (0.3,2000)<br>*<br>*	0.1 (0.1,1972)<br>*<br>-	-<br>*<br>*	-<br>*<br>*	-<br>*<br>-	-<br>*<br>*	-<br>*<br>*	-<br>*<br>*	-<br>*<br>*	-<br>*<br>*	-<br>*<br>*	2.9 (0.02)<br>*<br>*		-<br>*<br>*	-<br>*<br>-	-<br>*<br>*	-<br>*<br>-	2.9 (0.02,1990)<br>*<br>*	2.8 (0.03,2000)<br>*<br>*
**Rabbit**	20 (0.2,2004)<br>12 - 20 (2004)<br>*	167 (0.3,1989)<br>34 - 170 (1989)<br>0.4 - 170 (1989)	4622 (0.6,1986)<br>16 - 1230 (1989)<br>26 - 3115 (1988)	4767 (1.2,1986)<br>4205 - 5000 (1986)<br>0.3 - 20 (2004)	4022 (0.4,1986)<br>*<br>*	5000 (0.02,1986)<br>*<br>*	-<br>*<br>*	170 (0.1,1989)<br>*<br>0.3 - 170 (1989)	-<br>*<br>-	-<br>*<br>*	278 (0.04,1989)<br>*<br>*	-<br>*<br>*	-<br>*<br>*	5000 (0.01,1986)<br>*<br>*	5000 (0.2,1986)<br>918 - 3115 (1988)<br>1463 - 5000 ('86)	987 (0.2,2004)<br>*<br>*	-<br>*<br>-	4931 (2.4,1986)<br>3958 - 5000<br>*	-<br>*<br>*	4984 (0.2,1986)<br>2095 - 5000<br>*	1008 (0.9,2004)<br>5 - 20 (2004)<br>-	5000 (0.1,1986)<br>*<br>*	4184 (0.2,1986)<br>*<br>*
**Red deer**	8.7 (573,2008)<br>7.1 - 8.6 (2008)<br>*	8.5 (2806,2009)<br>8 - 8.6 (2009)<br>5.4 - 8.8 (2010)	8.6 (149,2010)<br>5.9 - 8.5 (2010)<br>1.6 - 7.9 (2010)	7.8 (1020,2009)<br>6.4 - 8 (2009)<br>2.3 - 8.9 (2009)	7 (1989,2008)<br>6.5 - 6.8 (2006)<br>5.7 - 7 (2003)	5 (0.6,2006)<br>*<br>-	17 (8.8,2010)<br>16 - 19 (2010)<br>*	9 (5992,2008)<br>8.9 - 9 (2008)<br>8 - 8.9 (2009)	6.9 (3.7,2010)<br>1.3 - 7.1 (2012)<br>-	9.1 (3538,2009)<br>9.1 - 9.2 (2009)<br>6.9 - 9.3 (2009)	8.2 (6034,2008)<br>7.8 - 8 (2008)<br>6.1 - 8.1 (2008)	7 (4706,2008)<br>6.5 - 6.7 (2008)<br>4.7 - 5.4 (2007)	11 (4267,2010)<br>11 (2010)<br>11 (2010)	6.9 (463,2003)<br>5.2 - 5.3 (2003)<br>0.7 (2003)	4.5 (7.6,2002)<br>2.5 - 7.4 (2008)<br>*	8 (849,2007)<br>7.1 - 8.6 (2009)<br>0.9 - 1.2 (2000)	3.2 (4.7,2000)<br>-<br>-	4.3 (46,2000)<br>2.2 - 3.7 (2000)<br>-	6.4 (59,2003)<br>4 - 6.6 (2003)<br>-	4.5 (24,2003)<br>1.7 - 4.1 (2000)<br>*	5.6 (6.6,2003)<br>1.5 - 5.8 (2006)<br>-	8.7 (8,2010)<br>3.8 - 7.5 (2008)<br>*	9.7 (22,2010)<br>4.3 - 11 (2010)<br>*
**Red fox**	2 (417,2006)<br>1.5 - 2.4 (2006)<br>*	3.1 (223,1998)<br>2.7 - 3.8 (1998)<br>0.9 - 3.8 (1998)	1.42 (2713.6,1997)<br>1.1 - 1.5 (1997)<br>0.4 - 2.3 (2006)	2 (2230,2006)<br>1.5 - 2.3 (2006)<br>0.5 - 2.5 (2006)	2 (321,2006)<br>1.7 - 2.5 (2006)<br>*	1.2 (39,1997)<br>*<br>-	2.14 (1.2,2006)<br>*<br>*	1.2 (222,1997)<br>0.8 - 1.3 (1997)<br>0.1 - 1.5 (2006)	1.2 (0.71,1997)<br>*<br>-	3.2 (45,1998)<br>2.1 - 2.8 (2006)<br>*	1.6 (57,1998)<br>0.9 - 2.1 (1998)<br>*	4.6 (31,1998)<br>2.8 - 4.9 (1998)<br>0.1 - 2.5 (2006)	-<br>*<br>*	1.7 (13,1998)<br>1.17 (1997)<br>*	3.1 (8.7,2006)<br>2.1 - 3.6 (2006)<br>*	1.3 (32,1997)<br>1.2 - 2.1 (1997)<br>*	4.2 (0.21,2006)<br>*<br>-	3.6 (5,2006)<br>2.9 - 3.6 (2006)<br>2.7 - 3.6 (2006)	2.8 (3.2,2006)<br>1.3 - 2.3 (2006)<br>*	2.3 (5.7,2006)<br>2 - 2.8 (2006)<br>0.1 - 1 (2006)	3.8 (0.6,2006)<br>*<br>-	1.4 (27,1997)<br>1.2 - 1.7 (2006)<br>*	2.7 (130,2006)<br>2.4 - 4.2 (2006)<br>0.8 - 16 (2004)
**Red squirrel**	77 (3.9,1998)<br>6.9 - 82 (1993)<br>*	39 (37,1998)<br>19 - 47 (1998)<br>1.6 - 64 (1998)	30 (0.8,1995)<br>6.6 - 27 (1991)<br>0.1 - 86 (1987)	28 (1.4,1995)<br>6.8 - 43 (1997)<br>1.3 - 49 (1997)	35 (0.5,1995)<br>*<br>0.2 - 26 (1998)	-<br>*<br>-	-<br>*<br>-	25 (0.7,1998)<br>6.1 - 43 (1998)<br>*	-<br>*<br>-	32 (0.4,1998)<br>6.2 - 26 (1998)<br>*	21 (0.5,1995)<br>2.3 - 26 (1998)<br>*	26 (0.5,1998)<br>4.6 - 26 (1998)<br>*	-<br>*<br>*	61 (0.02,1998)<br>*<br>-	223 (0.1,1993)<br>34 - 247 (1993)<br>7.4 - 219 (1995)	86 (0.1,1995)<br>*<br>*		245 (0.03,1996)<br>15 - 230 (1996)<br>-	-<br>*<br>-	1901 (0.01,1994)<br>*<br>4.1 - 230 (1996)	233 (0.03,1993)<br>*<br>-	161 (0.02,1996)<br>*<br>*	95 (0.2,1995)<br>0.9 - 142 (2000)<br>*
**Red-necked wallaby**	57.78 (0.43,1992)<br>*<br>-	-<br>-<br>*	-<br>*<br>*	-<br>*<br>*							-<br>*<br>-	-<br>*<br>-				58 (0.1,1992)<br>-<br>-							
**Roe deer**	27.02 (29.07,2002)<br>16 - 25 (1999)<br>0.8 - 23 (1995)	27.8 (135.7,2002)<br>23 - 28 (2002)<br>9.3 - 27 (1998)	27.96 (9.34,2002)<br>6.1 - 28 (2002)<br>3 - 28 (2002)	28 (7.5,2002)<br>5.7 - 27 (2002)<br>0 - 34 (1980)	28 (12,2002)<br>11 - 28 (2002)<br>5.3 - 28 (2002)	28 (0.7,2002)<br>*<br>-	28 (0.01,2002)<br>*<br>*	25 (0.2,1994)<br>4.4 - 25 (1994)<br>*	28 (0.004,2002)<br>*<br>-	27 (0.3,2002)<br>1.7 - 25 (1994)<br>*	26 (0.7,1994)<br>7.1 - 25 (1994)<br>*	-<br>*<br>*	-<br>*<br>*	27 (0.04,2002)<br>*<br>*	-<br>*<br>*	27 (0.2,2002)<br>*<br>*	-<br>*<br>-	-<br>*<br>*	-<br>*<br>*	-<br>*<br>*	-<br>*<br>-	28 (0.2,2002)<br>*<br>*	28 (1.5,2002)<br>7.5 - 27 (2002)<br>*
**Serotine**	0.25 (4.48,1991)<br>*<br>*	0.26 (0.13,1991)<br>*<br>*	0.28 (105.6,1991)<br>0.2 - 0.3 (1991)<br>0.1 - 0.3 (1991)	0.3 (29,1991)<br>0.2 - 0.3 (1991)<br>*	0.2 (1.3,1991)<br>*<br>*	0.2 (0.1,1991)<br>*<br>-	-<br>*<br>*	-<br>*<br>*	-<br>*<br>-	-<br>*<br>-	0.2 (0.01,1991)<br>*<br>-	-<br>-<br>*		0.2 (0.2,1991)<br>-<br>-	-<br>*<br>*	0.3 (0.2,1991)<br>*<br>-		-<br>*<br>-		-<br>*<br>*	-<br>*<br>-	0.2 (1.4,1991)<br>*<br>*	0.2 (8.5,1991)<br>0.1 - 0.2 (1991)<br>*
**Ship rat**	-<br>*<br>-		-<br>*<br>*	162 (0.3,1996)<br>147 - 162 (1996)<br>*	162 (0.3,1996)<br>-<br>*					162 (0.44,1996)<br>-<br>-	162 (0.12,1996)<br>-<br>*	-<br>-<br>*			162 (0.1,1996)<br>*<br>-		162 (0.07,'96)<br>-<br>-	162 (0.06,'96)<br>-<br>-	162 (0.2,1996)<br>-<br>123 - 162 (1996)	-<br>*<br>*		-<br>*<br>*	-<br>*<br>*
**Sika deer**	2.5 (47,1997)<br>1.4 - 3.1 (1997)<br>*	1.2 (241,1997)<br>1 - 1.3 (1997)<br>0.1 - 0.5 (1997)	1.1 (106,1997)<br>0.4 - 1 (1997)<br>0.2 - 7.2 (1998)	1.8 (190,1997)<br>1.4 - 2.2 (1997)<br>0.8 - 3.6 (1997)	1 (48,1997)<br>0.3 - 0.8 (1997)<br>*	5.9 (1.3,1997)<br>-<br>-	12 (1.5,1993)<br>-<br>*	1.8 (209,1997)<br>1.3 - 2 (1998)<br>0.6 - 2.2 (1998)	0.2 (0.1,1998)<br>-<br>-	2.5 (30,1998)<br>0.5 - 1.5 (1998)<br>0.1 - 1.3 (1998)	2.3 (57,1998)<br>1.7 - 2.6 (1998)<br>0.1 - 1.8 (1998)	0.7 (17,1998)<br>0.5 - 0.7 (1998)<br>*	2.5 (18,1997)<br>1.8 - 2.5 (1997)<br>*	1.4 (8.7,1997)<br>0 - 0 (1998)<br>-	0.3 (5.1,1997)<br>0.7 - 12 (1993)<br>*	1.4 (7.5,1997)<br>0.5 - 0.6 (1998)<br>-	12 (0.1,1993)<br>-<br>-	0.2 (0.7,1997)<br>*<br>-	0.2 (0.1,1997)<br>-<br>-	0.2 (45,1997)<br>-<br>0.1 - 0.3 (1997)	0.2 (3.5,1997)<br>0.1 - 0.2 (1997)<br>-	2.6 (3.5,1997)<br>-<br>-	1.5 (11,1997)<br>1 - 1.2 (1997)<br>*
**Skomer vole**	2414 (0.01,1992)<br>-<br>-			2414 (0.2,1992)<br>-<br>-	2414 (2.5,1992)<br>-<br>-									2414 (0.04,1992)<br>-<br>-	2414 (0.1,1992)<br>-<br>-		2414 (0.1,1992)<br>-<br>-		2414 (0.1,1992)<br>-<br>-	2414 (0.1,1992)<br>-<br>-	2414 (0.03,1992)<br>-<br>-		
**Stoat**	-<br>*<br>*	-<br>*<br>*	-<br>*<br>*	-<br>*<br>*	-<br>*<br>*	-<br>*<br>-	-<br>*<br>*	-<br>*<br>*	-<br>*<br>-	-<br>*<br>*	-<br>*<br>*	-<br>*<br>*	-<br>*<br>*	-<br>*<br>*	-<br>*<br>*	-<br>*<br>*	-<br>*<br>-	-<br>*<br>*	-<br>*<br>*	-<br>*<br>*	-<br>*<br>-	-<br>*<br>*	-<br>*<br>*
**Water shrew**	3 (0.2,2000)<br>*<br>*	3.1 (0.1,2000)<br>*<br>*	3 (8.4,1990)<br>1.6 - 3 (1990)<br>0.1 - 3 (1995)	3 (0.5,2000)<br>0.4 - 2.9 (1990)<br>*	3.1 (0.2,2000)<br>*<br>*		-<br>*<br>*	-<br>*<br>*	-<br>*<br>-	-<br>*<br>*	-<br>*<br>*	-<br>*<br>*	-<br>*<br>*	-<br>*<br>-	-<br>*<br>*	2.9 (0.02,1990)<br>*<br>*		-<br>*<br>*	-<br>*<br>-	-<br>*<br>*	-<br>*<br>-	2.9 (0.02,1990)<br>*<br>*	2.9 (0.03,2000)<br>*<br>*
**Water vole**	28.88 (0.4,2004)<br>14 - 24 (2004)<br>*	479 (0.2,1999)<br>38 - 3532 (1999)<br>26 - 2613 (1998)	2161 (0.1,2004)<br>*<br>17 - 167686	7039 (0.3,1998)<br>419 - 41923 (1999)<br>7.4 - 886 (1999)	9 (0.4,2004)<br>0.1 - 2.8 (1999)<br>*	0 (0.01,1999)<br>*<br>-	2.8 (0.001,1999)<br>*<br>*	2.7 (0.3,1999)<br>0.1 - 2.5 (1999)<br>*	-<br>*<br>-	2.3 (0.4,1999)<br>0.1 - 2.8 (1999)<br>*	182 (1.6,1999)<br>0.1 - 1.7 (1999)<br>0 - 1.9 (1999)	2.8 (1.8,1999)<br>0.1 - 2.8 (1999)<br>0 - 2.8 (1999)	2.8 (2,1999)<br>0.1 - 2.8 (1999)<br>0 - 2.8 (1999)	2.8 (0.02,1999)<br>0.01 - 2.8<br>-	-<br>*<br>*	3.5 (5.2,1999)<br>0.9 - 2.8 (1999)<br>-		-<br>*<br>*	2610 (0.2,1998)<br>-<br>-	2555 (0.03,1998)<br>*<br>*	93 (0.7,2004)<br>12 - 48 (2004)<br>-	-<br>57 - 9139 (2000)<br>*	377 (0.1,2004)<br>*<br>1 - 15160 (2000)
**Weasel**	253 (4.1,1981)<br>158 - 242 (1981)<br>*	75 (15,2000)<br>14 - 75 (2000)<br>4 - 75 (2000)	86 (1.3,1973)<br>28 - 163 (1981)<br>6.4 - 144 (1977)	84 (1.2,1973)<br>34 - 170 (1981)<br>*	77 (1.5,2000)<br>*<br>*	275 (0.07,1981)<br>*<br>-	-<br>*<br>*	75 (5.4,2000)<br>17 - 75 (2000)<br>*	-<br>*<br>-	75 (1.2,2000)<br>8.5 - 75 (2000)<br>*	75 (8.2,2000)<br>18 - 75 (2000)<br>*	75 (6,2000)<br>12 - 75 (2000)<br>*	-<br>*<br>*	75 (0.027,0)<br>*<br>-	13 (0.001,1973)<br>*<br>*	237 (0.01,1981)<br>4.5 - 75 (2000)<br>*	75 (0.01,2000)<br>-<br>-	13 (0.1,1973)<br>*<br>*	-<br>*<br>*	13 (0.07,1973)<br>4.4 - 13 (1973)<br>*	13 (0.02,1973)<br>*<br>-	75 (0.05,2000)<br>*<br>*	70 (0.1,1973)<br>*<br>*
**Whiskered bat**	1.5 (17,1990)<br>0.5 - 1.5 (1990)<br>*	1.5 (5.7,1990)<br>1.3 - 1.5 (1990)<br>*	1.5 (133.8,1990)<br>1.1 - 1.5 (1990)<br>0.3 - 1.5 (1990)	1.5 (88,1990)<br>1 - 1.5 (1990)<br>0.4 - 1.5 (1990)	1.5 (8.6,1990)<br>*<br>*	1.5 (4.4,1990)<br>-<br>-	1.5 (0.04,1990)<br>*<br>*	1.5 (0.3,1990)<br>0.7 - 1.5 (1990)<br>*		1.5 (0.3,1990)<br>*<br>*	1.5 (0.4,1990)<br>*<br>*	-<br>*<br>*	-<br>*<br>*	1.5 (0.2,1990)<br>*<br>-	-<br>*<br>*	1.5 (1.8,1990)<br>*<br>-		-<br>*<br>-		-<br>*<br>*	-<br>*<br>-	1.5 (2.6,1990)<br>0.6 - 1.5 (1990)<br>-	1.5 (12,1990)<br>1 - 1.5 (1990)<br>*
**Wild boar**	0.02 (1158,1997)<br>0.02 (1997)<br>-	0.02 (144,1997)<br>0.02 (1997)<br>*	0.022 (1886.2,1997)<br>0.02 (1997)<br>0.01 - 0.07 (1997)	0.02 (2421,1997)<br>0.02 - 0.03 (1997)<br>0 - 0.03 (1997)	0.03 (190,1997)<br>-<br>-	0.02 (84,1997)<br>-<br>-	0.03 (2.5,1997)<br>-<br>-	0.05 (1.3,1997)<br>-<br>*	0.02 (0.86,1997)<br>-<br>-	0.02 (18,1997)<br>-<br>-	0.02 (22,1997)<br>-<br>-			0.02 (11,1997)<br>-<br>-	0.02 (4.3,1997)<br>-<br>-	0.02 (31,1997)<br>0.02 (1997)<br>-	0.02 (0.1,1997)<br>-<br>-	0.02 (33.89,1997)<br>-<br>-	0.02 (0.01,1997)<br>-<br>-	0.02 (9,1997)<br>-<br>-	0.02 (1.1,1997)<br>-<br>-	0.02 (67,1997)<br>-<br>-	0.02 (439,1997)<br>-<br>-
**Wildcat**	0.4 (3.6,1978)<br>0.1 - 0.3 (1978)<br>-	0.4 (37,1978)<br>0.3 - 0.4 (1978)<br>0.01 - 0.6 (2010)	0.36 (0.57,1978)<br>0 - 0.3 (1978)<br>*	0.4 (5.5,1978)<br>0.3 (1978)<br>*	0.3 (2,1978)<br>0.2 - 0.3 (1978)<br>*		-<br>*<br>-	0.4 (2.7,1978)<br>0.3 (1978)<br>*		0.3 (52,1978)<br>0.3 (1978)<br>0.2 - 0.3 (1978)	0.3 (13,1978)<br>0.2 - 0.5 (1978)<br>0 - 0.6 (2010)	0.4 (4.9,1978)<br>0.3 - 0.5 (1978)<br>*	0.3 (14,1978)<br>0.2 - 0.3 (1978)<br>0 - 0.3 (1978)	0.3 (0.01,1978)<br>-<br>-	-<br>*<br>-	0.3 (0.9,1978)<br>*<br>*			-<br>*<br>-	-<br>*<br>-		0.4 (0.1,1978)<br>*<br>-	0.6 (0.1,2010)<br>*<br>*
**Wood mouse**	516 (2.6,2002)<br>23 - 814 (2003)<br>*	387 (0.3,2000)<br>13 - 864 (1987)<br>0.1 - 864 (1987)	247.17 (12.29,1990)<br>74 - 610 (2000)<br>2.1 - 1357 (1994)	72 (44,2003)<br>22 - 194 (2003)<br>1.4 - 883 (2003)	162 (1.8,2002)<br>9.6 - 640 (1995)<br>0 - 247 (1994)	46 (0.1,2003)<br>*<br>*	46 (0.03)<br>*<br>*	46 (0.2,2003)<br>*<br>*	-<br>*<br>-	46 (0.01,2003)<br>*<br>*	46 (0.1,2003)<br>*<br>*	-<br>*<br>*	-<br>*<br>*	46 (0.004,2003)<br>*<br>*	2051 (0.01,2002)<br>*<br>*	174 (0.2,2004)<br>*<br>*	-<br>*<br>-	-<br>*<br>*	-<br>*<br>-	9.9 (0.001,2004)<br>*<br>*	51 (0.7,2004)<br>2.5 - 9.9 (2004)<br>-	889 (0.1,2002)<br>*<br>*	1906 (4.5,2002)<br>914 - 1869 (2002)<br>56 - 1039 (2003)
**Yellow-necked mouse**	39 (0.6,1996)<br>9.2 - 29 (2003)<br>*	29 (0.04,2003)<br>*<br>*	38.29 (9.33,1990)<br>13 - 32 (1996)<br>1.2 - 22 (1997)	29 (7.2,2003)<br>9.8 - 28 (2003)<br>0 - 4.7 (1996)	29 (0.5,2003)<br>*<br>-	0 (0.001,1997)<br>-<br>-		29 (0.01,2003)<br>*<br>*		29 (0.004,2003)<br>-<br>-	29 (0.01,2003)<br>*<br>-					41 (0.03,1996)<br>*<br>-						47 (0.02,1990)<br>-<br>*	36 (0.1,1990)<br>*<br>*

B.3 Reference list

Aars, J., X. Lambin, R. Denny and A. C. Griffin (2001). Water vole in the Scottish uplands: distribution patterns of disturbed and pristine populations ahead and behind the American mink invasion front. *Animal Conservation* 4(3): 187-194.

Baker, P. J., A. J. Bentley, R. J. Ansell and S. Harris (2005). Impact of predation by domestic cats *Felis catus* in an urban area. *Mammal Review* 35(3-4): 302-312.

Baker, P. J., S. M. Funk, M. W. Bruford and S. Harris (2004). Polygynandry in a red fox population: implications for the evolution of group living in canids? *Behavioral Ecology* 15(5): 766-778.

Battersby, J. and Tracking Mammals Partnership (2005). UK mammals: species status and population trends. First report by the Tracking Mammals Partnership, JNCC/Tracking Mammals Partnership, Peterborough.

Benge, J. M. (2004). The ecology of the water vole (*Arvicola terrertris*) in Southern England. Ph.D. Thesis, University of Hertfordshire.

Berry, R. J. (1968). The ecology of an island population of the house mouse. *Journal of Animal Ecology* 37(2): 445-470.

Birks, J. D. S. (1981). Home range and territorial behaviour of the feral mink (*Mustela vision*) in Devon. Ph.D. Thesis, University of Exeter.

Birks, J. D. S. (1997). A volunteer-based system for sampling variations in the abundance of polecats (*Mustela putorius*). *Journal of Zoology* 243(4): 857-863.

Bonesi, L. and D. W. Macdonald (2004). Evaluation of sign surveys as a way to estimate the relative abundance of American mink (*Mustela vison*). *Journal of Zoology* 262(1): 65-72.

Bright, P. W. and E. C. Halliwell (1999). Species recovery programme for the pine marten in England: 1996-1998. English Nature.

Bryce, J., P. J. Johnson and D. W. Macdonald (2002). Can niche use in red and grey squirrels offer clues for their apparent coexistence*? Journal of Applied Ecology* 39(6): 875-887.

Bullock, D. J. (1985). Annual diets of hill sheep and feral goats in southern Scotland. *Journal of Applied Ecology* 22(2): 423-433.

Burgess, M., P. Morris and P. Bright (2003). Population dynamics of the edible dormouse (*Glis glis*) in England*. Acta Zoologica Academiae Scientarum Hungaricae* 49(1): 27-31.

Burthe, S., S. Telfer, X. Lambin, M. Bennett, D. Carslake, A. Smith and M. Begon (2006). Cowpox virus infection in natural field vole *Microtus agrestis* populations: delayed density dependence and individual risk. *Journal of Animal Ecology* 75(6): 1416-1425.

Bush, E. R., C. D. Buesching, E. M. Slade and D. W. Macdonald (2012). Woodland recovery after suppression of deer: cascade effects for small mammals, wood mice (*Apodemus sylvaticus*) and bank voles (*Myodes glareolus*). *PLOS ONE* 7(2): e31404.

Churchfield, S. and V. K. Brown (1987). The trophic impact of small mammals in successional grasslands. *Biological Journal of the Linnean Society* 31(3): 273-290.

Clutton-Brock, T. H., O. F. Price, S. D. Albon and P. A. Jewell (1991). Persistent instability and population regulation in Soay Sheep. *Journal of Animal Ecology* 60(2): 593-608.

Cooke, A. S. and L. Farrell (2001). Impact of muntjac deer (*Muntiacus reevesi*) at Monks Wood National Nature Reserve, Cambridgeshire, eastern England. *Forestry* 74(3): 241-250.

Corbett, L. K. (1979). Feeding ecology and social organization of wildcats. Ph.D. Thesis, University of Aberdeen.

Dards, J. L. (1981). Habitat utilisation by feral cats in Portsmouth dockyard. *The Ecology And Control of Feral Cats*, Royal Holloway College, University of London, The Universities Federation for Animal Welfare.

Delahay, R. J., N. Walker, G. J. Forrester, B. Harmsen, P. Riordan, D. W. Macdonald, C. Newman and C. L. Cheeseman (2006). Demographic correlates of bite wounding in European badgers (*Meles meles*) in stable and perturbed populations. *Animal Behaviour* 71(5): 1047-1055.

Doncaster, C. P. (1994). Factors regulating local variations in abundance: field tests on hedgehogs, *Erinaceus europaeus*. *Oikos* 69(2): 182-192.

Edwards, T. and W. Kenyon (2013). SPICe briefing : wild deer in Scotland. Scottish Natural Heritage.

Fairless, L. (2013). Ecology and ecophysiology of social structure and population dynamics in bats (*Vespertilionidae*). Ph.D. Thesis, University of Southampton.

Flowerdew, J. R. and S. A. Ellwood (2001). Impacts of woodland deer on small mammal ecology. *Forestry* 74(3): 277-287.

Flowerdew, J. R., R. F. Shore, S. M. C. Poulton and T. H. Sparks (2004). Live trapping to monitor small mammals in Britain. *Mammal Review* 34(1-2): 31-50.

Flux, J. E. C. and P. J. Fullagar (1992). World distribution of the rabbit *Oryctolagus funiculus* on islands. *Mammal Review* 22(3-4): 151-205.

Forman, D. W. (2005). An assessment of the local impact of native predators on an established population of British water voles (*Arvicola terrestris*). *Journal of Zoology* 266(3): 221-226.

Gelling, M., D. W. Macdonald and F. Mathews (2007). Are hedgerows the route to increased farmland small mammal density? Use of hedgerows in British pastoral habitats. *Landscape Ecology* 22(7): 1019-1032.

Gill, R. M. A., A. L. Johnson, A. Francis, K. Hiscocks and A. J. Peace (1996). Changes in roe deer (*Capreolus capreolus*) population density in response to forest habitat succession. *Forest Ecology and Management* 88(1-2): 31-41.

Gill, R. M. A., M. L. Thomas and D. Stocker (1997). The use of portable thermal imaging for estimating deer population density in forest habitats. *Journal of Applied Ecology* 34(5): 1273-1286.

Gorman, M. L. and P. Reynolds (1993). The impact of land-use change on voles and raptors. *Mammal Review* 23(3-4): 121-126.

Graham, I. M. (2002). Estimating weasel *Mustela nivalis* abundance from tunnel tracking indices at fluctuating field vole *Microtus agrestis* density. *Wildlife Biology* 8(4): 279-287.

Gurnell, J., P. W. W. Lurz, M. D. F. Shirley, S. Cartmel, P. J. Garson, L. Magris and J. Steele (2004a). Monitoring red squirrels *Sciurus vulgaris* and grey squirrels *Sciurus carolinensis* in Britain. *Mammal Review* 34(1-2): 51-74.

Gurnell, J., L. A. Wauters, P. W. W. Lurz and G. Tosi (2004b). Alien species and interspecific competition: effects of introduced eastern grey squirrels on red squirrel population dynamics. *Journal of Animal Ecology* 73(1): 26-35.

Hall, S. J. G. (1982). The Chillingham herd of wild white cattle. *Applied Animal Ethology* 9(1): 96-97.

Halliwell, E. C. (1997). The ecology of red squirrels in Scotland in relation to pine marten predation. Ph.D. Thesis, University of Aberdeen.

Harrington, L. A., A. L. Harrington and D. W. Macdonald (2008). Estimating the relative abundance of American mink *Mustela vison* on lowland rivers: evaluation and comparison of two techniques. *European Journal of Wildlife Research* 54(1): 79-87.

Harris, S., P. Morris, S. Wray and D. W. Yalden (1995). A review of British mammals: population estimates and conservation status of British mammals other than cetaceans. JNCC.

Harris, S. & Yalden, D.W. (2008) Mammals of the British Isles: Handbook. pp. 799. The Mammal Society, Southampton.

Hellawell, T. C. (1991). Aspects of the ecology and management of the feral goat (*Capra hircus*): populations of the Rhinogau and Maentwrog areas, North Wales. Ph.D. Thesis, University of Wales, Cardiff.

Hemami, M. R., A. R. Watkinson and P. M. Dolman (2005). Population densities and habitat associations of introduced muntjac *Muntiacus reevesi* and native roe deer Capreolus capreolus in a lowland pine forest. *Forest Ecology and Management* 215(1–3): 224-238.

Hemami, M. R., A. R. Watkinson, R. M. A. Gill and P. M. Dolman (2007). Estimating abundance of introduced Chinese muntjac Muntiacus reevesi and native roe deer *Capreolus capreolus* using portable thermal imaging equipment. *Mammal Review* 37(3): 246-254.

Heydon, M. J., J. C. Reynolds and M. J. Short (2000). Variation in abundance of foxes (*Vulpes vulpes*) between three regions of rural Britain, in relation to landscape and other variables. *Journal of Zoology* 251(2): 253-264.

Holm, J. L. (1990). The ecology of red squirrels (*Sciurus vulgaris*) in deciduous woodlands. Ph.D. Thesis, Royal Holloway, University of London.

Hounsome, T. D., R. P. Young, J. Davison, R. W. Yarnell, I. D. Trewby, B. T. Garnett, R. J. Delahay and G. J. Wilson (2005). An evaluation of distance sampling to estimate badger (*Meles meles*) abundance. *Journal of Zoology* 266(1): 81-87.

Hutchings, M. R., K. M. Service and S. Harris (2001). Defecation and urination patterns of badgers *Meles meles* at low density in south west England. *Acta Theriologica* 46(1): 87-96.

Hutchings, M. R., K. M. Service and S. Harris (2002). Is population density correlated with faecal and urine scent marking in European badgers (*Meles meles*) in the UK? *Mammalian Biology* 67(5): 286-293.

Jones, K. E., J. D. Altringham and R. Deaton (1996). Distribution and population densities of seven species of bat in northern England. *Journal of Zoology* 240(4): 788-798.

Kenward, R. E., K. H. Hodder, R. J. Rose, C. A. Walls, T. Parish, J. L. Holm, P. A. Morris, S. S. Walls and F. I. Doyle (1998). Comparative demography of red squirrels (*Sciurus vulgaris*) and grey squirrels (*Sciurus carolinensis*) in deciduous and conifer woodland. *Journal of Zoology* 244(1): 7-21.

Kilshaw, K., P. J. Johnson, A. C. Kitchener and D. W. Macdonald (2014). Detecting the elusive Scottish wildcat *Felis silvestris* using camera trapping. *Oryx* 49(2): 207-215.

Kolb, H. H. (1991a). Use of burrows and movements by wild rabbits (*Oryctolagus cuniculus*) on an area of sand dunes. *Journal of Applied Ecology* 28(3): 879-891.

Kolb, H. H. (1991b). Use of burrows and movements of wild rabbits (*Oryctolagus cuniculus*) in an area of hill grazing and forestry. *Journal of Applied Ecology* 28(3): 892-905.

Kotzageorgis, G. C. and C. F. Mason (1997). Small mammal populations in relation to hedgerow structure in an arable landscape. *Journal of Zoology* 242(3): 425-434.

Kruuk, H., A. Moorhouse, J. W. H. Conroy, L. Durbin and S. Frears (1989). An estimate of numbers and habitat preferences of otters *Lutra lutra* in Shetland, UK. *Biological Conservation* 49(4): 241-254.

Lambin, X., S. J. Petty and J. L. Mackinnon (2000). Cyclic dynamics in field vole populations and generalist predation. *Journal of Animal Ecology* 69(1): 106-119.

Langbein, J., M. R. Hutchings, S. Harris, C. Stoate, S. C. Tapper and S. Wray (1999). Techniques for assessing the abundance of Brown Hares *Lepus europaeus*. *Mammal Review* 29(2): 93-116.

Lowe, V. P. W. (1969). Population Dynamics of the Red Deer (*Cervus elaphus*) on Rhum. *Journal of Animal Ecology* 38(2): 425-457.

Macdonald, D. W., R. P. D. Atkinson and G. Blanchard (1996). Spatial and temporal patterns in the activity of European moles. *Oecologia* 109(1): 88-97.

Macdonald, D. W. and C. Newman (2002). Population dynamics of badgers (*Meles meles*) in Oxfordshire, U.K.: numbers, density and cohort life histories, and a possible role of climate change in population growth. *Journal of Zoology* 256(1): 121-138.

Macdonald, D. W., C. Newman, P. M. Nouvellet and C. D. Buesching (2009). An analysis of Eurasian badger (*Meles meles*) population dynamics: implications for regulatory mechanisms. *Journal of Mammalogy* 90(6): 1392-1403.

Mallorie, H. C. and J. R. Flowerdew (1994). Woodland small mammal population ecology in Britain: a preliminary review of the Mammal Society survey of Wood Mice *Apodemus sylvaticus* and Bank Voles *Clethrionomys glareolus*, 1982–87. *Mammal Review* 24(1): 1-15.

Marques, F. F. C., S. T. Buckland, D. Goffin, C. E. Dixon, D. L. Borchers, B. A. Mayle and A. J. Peace (2001). Estimating deer abundance from line transect surveys of dung: sika deer in southern Scotland. *Journal of Applied Ecology* 38(2): 349-363.

Mayle, B. A. (1996). Progress in predictive management of deer populations in British woodlands. *Forest Ecology and Management* 88(1–2): 187-198.

McDonald, R. A., M. R. Hutchings and J. G. M. Keeling (1997). The status of ship rats *Rattus rattus* on the Shiant Islands, Outer Hebrides, Scotland. *Biological Conservation* 82(1): 113-117.

Moore, N. P., S. S. Roy and A. Helyar (2003). Mink (*Mustela vison*) eradication to protect ground-nesting birds in the Western Isles, Scotland, United Kingdom. *New Zealand Journal of Zoology* 30(4): 443-452.

Moors, P. J. (1975). The annual energy budget of a weasel (*Mustela nivalis*) population in farmland. Ph.D. Thesis, University of Aberdeen.

Morris, P. A., S. Munn and S. Craig-Wood (1992). The effects of releasing captive hedgehogs (*Erinaceus europaeus*) into the wild. *Field Studies* 8: 89-99.

O'Mahony, D., X. Lambin, J. L. MacKinnon and C. Coles (1999). Fox predation on cyclic field vole populations in Britain. *Ecography* 22(5): 575-581.

Page, R. J. C., J. Ross and D. H. Bennet (1992). A study of the home ranges, movements and behaviour of the feral cat population at Avonmouth Docks. *Wildlife Research* 19(3): 263-277.

Palphramand, K. L., G. Newton-Cross and P. C. L. White (2007). Spatial organization and behaviour of badgers (*Meles meles*) in a moderate-density population. *Behavioral Ecology and Sociobiology* 61(3): 401-413.

Parrott, D., A. Prickett, S. Pietravalle, T. R. Etherington and M. Fletcher (2012). Estimates of regional population densities of badger *Meles meles*, fox *Vulpes vulpes* and hare *Lepus europaeus* using walked distance sampling. *European Journal of Wildlife Research* 58(1): 23-33.

Pernetta, J. C. (1977). Population ecology of British shrews in grassland. *Acta Theriologica* 22(20): 279-296.

Putman, R. J. and J. R. Clifton-Bligh (1997). Age-related body weight, fecundity and population change in a south Dorset sika population (*Cervus nippon*); 1985–1993. *Journal of Natural History* 31(4): 649-660.

Reeve, N. J. (1981). A field study of the hedgehog (*Erinaceus europaeus*) with particular reference to movements and behaviour. Ph.D. Thesis, University of London.

Reynolds, J. C., C. Stoate, M. H. Brockless, N. J. Aebischer and S. C. Tapper (2010). The consequences of predator control for brown hares (*Lepus europaeus*) on UK farmland. *European Journal of Wildlife Research* 56(4): 541-549.

Robinson, M. F. and R. E. Stebbings (1997). Home range and habitat use by the serotine bat, *Eptesicus serotinus*, in England. *Journal of Zoology* 243(1): 117-136.

Rogers, L. M., C. L. Cheeseman, P. J. Mallinson and R. Clifton-Hadley (1997). The demography of a high-density badger (*Meles meles*) population in the west of England. *Journal of Zoology* 242(4): 705-728.

Roper, T. J., L. Conradt, J. Butler, S. E. Christian, J. Ostler and T. K. Schmid (1993). Territorial marking with faeces in badgers (*Meles meles*): A comparison of boundary and hinterland latrine use. *Behaviour* 127(3-4): 289-307.

Shore, R. F., W. R. Meek, T. H. Sparks, R. F. Pywell and M. Nowakowski (2005). Will environmental stewardship enhance small mammal abundance on intensively managed farmland? *Mammal Review* 35(3-4): 277-284.

Shuttleworth, C. M. (1996). The effect of supplemental feeding on the red squirrel (*Sciurus vulgaris*). Ph.D. Thesis, University of London.

Smith, R. K., N. V. Jennings, A. Robinson and S. Harris (2004). Conservation of European hares *Lepus europaeus* in Britain: is increasing habitat heterogeneity in farmland the answer? *Journal of Applied Ecology* 41(6): 1092-1102.

Speakman, J. R., P. A. Racey, C. M. C. Catto, P. I. Webb, S. M. Swift and A. M. Burnett (1991). Minimum summer populations and densities of bats in NE Scotland, near the northern borders of their distributions. *Journal of Zoology* 225(2): 327-345.

Stone, R. D. (1986). The social ecology of the European mole, *Talpa europaea* and the Pyrenean desman, *Galemys pyrenaicus*: a comparative study. Ph.D. Thesis, University of Aberdeen.

Tapper, S. C. and R. F. W. Barnes (1986). Influence of Farming Practice on the Ecology of the Brown Hare (*Lepus europaeus*). *Journal of Applied Ecology* 23(1): 39-52.

Tattersall, F. H., D. W. Macdonald, B. J. Hart, P. Johnson, W. Manley and R. Feber (2002). Is habitat linearity important for small mammal communities on farmland? *Journal of Applied Ecology* 39(4): 643-652.

Telfer, S., H. E. Clough, R. J. Birtles, M. Bennett, D. Carslake, S. Helyar and M. Begon (2007). Ecological differences and coexistence in a guild of microparasites: *Bartonella* in wild rodents. *Ecology* 88(7): 1841-1849.

Telfer, S., J. F. Dallas, J. Aars, S. B. Piertney, W. A. Stewart and X. Lambin (2003). Demographic and genetic structure of fossorial water voles (*Arvicola terrestris*) on Scottish islands. *Journal of Zoology* 259(1): 23-29.

Trout, R. C., S. E. Brooks, P. Rudlin and J. Neil (2012). The effects of restoring a conifer Plantation on an Ancient Woodland Site (PAWS) in the UK on the habitat and local population of the Hazel Dormouse (*Muscardinus avellanarius*). *European Journal of Wildlife Research* 58(4): 635-643.

Waters, D., G. Jones and M. Furlong (1999). Foraging ecology of Leisler's bat (*Nyctalus leisleri*) at two sites in southern Britain. *Journal of Zoology* 249(2): 173-180.

Watson, A., R. Hewson, D. Jenkins and R. Parr (1973). Population densities of mountain hares compared with red grouse on Scottish moors. *Oikos* 24(2): 225-230.

Wauters, L. A., P. W. W. Lurz and J. Gurnell (2000). Interspecific effects of grey squirrels (*Sciurus carolinensis*) on the space use and population demography of red squirrels (*Sciurus vulgaris*) in conifer plantations. *Ecological Research* 15(3): 271-284.

Weir, A., J. McLeod and C. E. Adams (1995). The winter diet and parasitic fauna of a population of red-necked wallabies *Macropus rufogriseus* recently introduced to Scotland. *Mammal Review* 25(3): 111-116.

White, T. A. and J. B. Searle (2007). Genetic diversity and population size: island populations of the common shrew, *Sorex araneus*. *Molecular Ecology* 16(10): 2005-2016.

Wilson, C. J. (2003). Distribution and status of feral wild boar *Sus scrofa* in Dorset, southern England. *Mammal Review* 33(3-4): 302-307.

Young, R. P., J. Davison, I. D. Trewby, G. J. Wilson, R. J. Delahay and C. P. Doncaster (2006). Abundance of hedgehogs (*Erinaceus europaeus*) in relation to the density and distribution of badgers (*Meles meles*). *Journal of Zoology* 269(3): 349-356.

C Environmental data

Environmental data to predict habitat suitability was sourced, with the exception of land cover, from publically available sources. In each case maps were converted to a standard 25m raster based on the British National grid as described below. Whilst not all of the maps used were originally available at this level of precision this standard resolution was adopted to preserve the information in those that were, predominantly land cover. The intersection of these maps was then produced and used as a mask in the "extract raster by mask" tool in ArcGIS v10.2 to ensure all maps had a uniform coverage. Using these maps as a standard reference we generated a set of rasters for each of our chosen resolutions using the "zonal statistics" tool to apply the mean (or majority in the case of land cover) value to each block of 25m cells corresponding to a new cell and then applying the "aggregate" tool to combine these into a single entity.

C.1 Altitude and bioclimatic variables

Raster based maps for the GB region describing altitude and 19 bioclimatic variables projected over the period 1950-2000 were downloaded from WorldClim - Global Climate Data (http://www.worldclim.org) at the highest available resolution (30 arcseconds) on 28/02/2015. These dataset are freely available for academic and other non-commercial use. All raster maps were originally referenced in a global spatial referencing frame. In order to standardise these maps we initially projected them onto the British National Grid using the "project raster" tool in ArcGIS v10.2 before using the "resample" tool to convert them to a standard 25m raster matching the land cover map.

The bioclimatic variables are coded as follows:

BIO1 = Annual Mean Temperature

BIO2 = Mean Diurnal Range (Mean of monthly (max temp - min temp))

BIO3 = Isothermality (BIO2/BIO7) (* 100)

BIO4 = Temperature Seasonality (standard deviation *100)

BIO5 = Max Temperature of Warmest Month

BIO6 = Min Temperature of Coldest Month

BIO7 = Temperature Annual Range (BIO5-BIO6)

BIO8 = Mean Temperature of Wettest Quarter

BIO9 = Mean Temperature of Driest Quarter

BIO10 = Mean Temperature of Warmest Quarter

BIO11 = Mean Temperature of Coldest Quarter

BIO12 = Annual Precipitation

BIO13 = Precipitation of Wettest Month

BIO14 = Precipitation of Driest Month

BIO15 = Precipitation Seasonality (Coefficient of Variation)

BIO16 = Precipitation of Wettest Quarter

BIO17 = Precipitation of Driest Quarter

BIO18 = Precipitation of Warmest Quarter

BIO19 = Precipitation of Coldest Quarter

For more information on how these maps were generated readers should refer to the following publication:

Hijmans, R.J., S.E. Cameron, J.L. Parra, P.G. Jones and A. Jarvis (2005). Very high resolution interpolated climate surfaces for global land areas. International Journal of Climatology 25: 1965-1978.

C.2 Land Cover

Land cover was represented by the 25m target land cover raster 2007 which categorises dominant coverage into 23 different classes. This maps was used under the core Defra license agreement. Whilst it would be possible to apply the methods outlined here using the publically available resolution of 1km the 25m raster provides the most accurate representation of land cover within Great Britain and would be required in future to perform analysis at a 100m resolution should more precise occurrence data become available.

Full details of this product is provided in the following publication:

Morton, R.D.; Rowland, C.; Wood, C.; Meek, L.; Marston, G.; Smith, G.; Wadsworth, R.; Simpson, I. (2011). Land Cover Map 2007 (25m raster, GB) (WITHDRAWN). NERC Environmental Information Data Centre. doi:10.5285/a382af78-129e-4326-a561-d3034b72c4a3

C.3 Human Population

Census output from 2011 was used as the basis for human density. A GB polygon map was produced in ArcGIS v10.2 by combining publically available geographic information for England and Wales from the Office of National Statistics Scotland from the Scottish Office of Statistics. In both cases maps were downloaded describing the output area level on the 28/02/2015. The combined maps was then converted to a 25m raster maps using the “polygon to raster” tool in ArcGIS v10.2 with the 25m land cover maps as a reference grid.
