# Supplementary material for: A systematic approach to estimate the distribution and total abundance of British mammals
Source: PLoS One. 2017 Jun 28;12(6):e0176339. doi: 10.1371/journal.pone.0176339 (PMC5489149; doi:10.1371/journal.pone.0176339)
Supplement: S2 File — A summary breakdown of the model output for each species and additional maps showing predicted species richness and total biomass for a variety of species subsets, specifically, by origin (native or non-native), by status (common, local and rare) and by species order (Marsupialia, Insectivora, Carnivora, Artiodactyla, Chiroptera, Rodentia and Lagomorpha). (PDF) [file pone.0176339.s002.pdf]

Supplement 2: Model results and predictions

S2.1 Summary of modelling results and predictions

Table A. Summary of modelling results for each species grouped by taxonomic order. Statistics for habitat suitability predictions include: the mean Area Under Curve of the "best" model across 100 repetitions (AUC describes the capability of species distribution models to correctly predict occurrence and absence with a random model displaying an AUC of 0.5 and the "best" model as that with the highest value); the most commonly selected "best" model (Mode) across all repetitions; the spatial extent of predicted occurrence (number of grid cells where occurrence is predicted - values in brackets indicate the corresponding extent of observed occurrence); based on a resolution of 10km (all records) and 1km (occurrence records <= 1km only) respectively. Statistics for abundance include: the estimate as presented by Harris et al. 1995 [1] (numbers in subscript indicate the reliability of the estimate, where 5 denotes the least, and 1 most, reliable.); the predicted range of abundance (upper and lower bound produced when projecting density polygons onto a raster grid assuming estimate applies to entire cell and estimate applies only to the proportion of area surveyed respectively) and the minimum predicted change from the 1995 estimate (trend direction denoted by arrow shown in brackets) based on a resolution of 10km and 1km respectively; the population trend pre 1995 as published by Harris et al. 1995 [1], where (*) indicates no trend available and (↑), (↓) and (↔) indicate increasing, decreasing and stable population trends respectively; the population trend post 1995 taken as the most recent prediction from a selection of sources which use temporal fluctuations in species recording to infer changes in relative abundance (arrows in brackets show the published trend from each source as follows: Battersby et al. 2005 [2]; NGC GWCT 1995 - 2009 [3]; BBS 1995 - 2014 [4]; BCT 2014 [5]).

Symbols relating to abundance predictions are as follows: (γ) denotes abundance modelled by generalised linear regression model using gamma distribution; (sph) and (gau) denote abundance modelled by mixed linear regression model using spherical and Gaussian spatial autocorrelation functions respectively; (0), (1) and (2) denote the order of regression used to relate habitat suitability score and density (i.e. 0: d~1, 1: d~hs and 2: d~hs+I(hs^2)). Unless otherwise stated regression was performed using a simple linear model according to a Gaussian distribution with no spatial autocorrelation.

	**Habitat Suitability**						**Abundance**						
**Common Name**	**AUC (10km)**	**Mode (10km)**	**Extent (10km)**	**AUC (1km)**	**Mode (1km)**	**Extent (1km)**	**1995 estimate[1]**	**Predicted range (10km)**	**Min. change (10km)**	**Predicted range (1km)**	**Min. change (1km)**	**Trend pre 1995**	**Trend post 1995**
**Marsupialia**													
Red-necked wallaby	0.78	MaxEnt	126 (25)	0.85	MaxEnt	8611 (17)	29^{1}	3,590^{0} - 728,030^{0}	123 (↑)	61,019$^{\gamma,0}$ - 494,959^{gau,0}	2,103 (↑)	*	* (****)
**Insectivora**													
Hedgehog	0.86	MaxEnt	2,137 (2,347)	0.69	RF	65,647 (26,572)	1,555,000^{4}	731,546^{sph,2} - 11,979,363^{sph,2}	0 (↔)	657,712^{sph,2} - 3,222,149^{sph,1}	0 (↔)	↓	↔ (↔↔**)
Mole	0.84	MaxEnt	2,018 (2,384)	0.63	RF	94,551 (22,602)	31,000,000^{3}	126,065$^{\gamma,0}$ - 109,231,019$^{\gamma,0}$	0 (↔)	2,976,177^{0} - 61,561,156$^{\gamma,1}$	0 (↔)	*	↔ (↔***)
Common shrew	0.70	MaxEnt	1,808 (1,877)	0.64	MaxEnt	80,620 (4,942)	41,700,000^{3}	1,838,490^{sph,2} - 223,651,088^{sph,2}	0 (↔)	11,469,553^{sph,0} - 56,014,632^{sph,0}	0 (↔)	*	* (****)
Pygmy shrew	0.66	MaxEnt	1,533 (1,309)	0.65	MaxEnt	63,138 (2,122)	8,600,000^{4}	11,566^{0} - 36,213,441^{0}	0 (↔)	113,760^{0} - 23,992,074^{sph,0}	0 (↔)	*	* (****)
Water shrew	0.65	MaxEnt	1,506 (1,080)	0.67	MaxEnt	59,072 (1,716)	1,900,000^{4}	22,166^{1} - 406,189^{1}	-0.8 (↓)	82,134^{0} - 175,655^{sph,0}	-0.91 (↓)	↓	* (****)
Lesser white-toothed shrew	-	-	- (4)	-	-	- (3)	14,000^{4}	-	-	-	-	*	* (****)

**Chiroptera**												
Greater horseshoe bat	0.76	MaxEnt	355 (282)	0.66	MaxEnt	11,039 (981)	4,000^{2}	-	-	-	-	↓	↑ (↑**↑)
Lesser horseshoe bat	0.73	MaxEnt	517 (438)	0.65	RF	18,747 (2,232)	14,000^{2}	-	-	-	-	↓	↑ (↑**↑)
Whiskered bat	0.67	MaxEnt	1,046 (608)	0.64	RF	44,029 (979)	40,000^{4}	30,892$^{\gamma,0}$ - 145,757^{0}	0 (↔)	0^{sph,1} - 65,734^{sph,1}	0 (↔)	↓	↔ (↔**↔)
Brandt's bat	0.63	SVM	820 (228)	0.63	MaxEnt	50,780 (269)	30,000^{5}	17,894$^{\gamma,0}$ - 129,646$^{\gamma,0}$	0 (↔)	5^{sph,1} - 86,077^{sph,0}	0 (↔)	↓	↔ (↔**↔)
Natterer's bat	0.69	RF	1,392 (1,103)	0.65	RF	51,836 (2,334)	100,000^{4}	76,593^{0} - 1,709,679^{sph,0}	0 (↔)	77,209^{sph,2} - 670,172^{sph,1}	0 (↔)	↓	↑ (↑**↑)
Bechstein's bat	0.72	GLM	214 (100)	0.69	MaxEnt	6768 (204)	1,500^{2}	17,424^{0} - 6,590,798^{0}	10.6 (↑)	145,598^{sph,0} - 2,202,891^{gau,0}	96.1 (↑)	↓	* (****)
Greater mouse-eared bat	-	-	- (8)	0.86	MaxEnt	12,403 (12)	0^{1}	-	-	-	-	↓	* (****)
Daubenton's bat	0.69	MaxEnt	1,493 (1,375)	0.65	RF	73,026 (3,268)	150,000^{4}	39,292$^{\gamma,0}$ - 245,200^{sph,0}	0 (↔)	478^{sph,2} - 124,228^{sph,0}	-0.17 (↓)	↓	↔ (↑**↔)
Serotine	0.70	RF	715 (561)	0.64	MaxEnt	28,756 (1,656)	15,000^{4}	5,419$^{\gamma,0}$ - 20,733$^{\gamma,0}$	0 (↔)	0^{sph,0} - 7,736^{sph,0}	-0.48 (↓)	↓	↔ (***↔)
Noctule	0.69	RF	1,374 (1,126)	0.64	RF	50,932 (3,581)	50,000^{3}	26,182$^{\gamma,0}$ - 190,611^{1}	0 (↔)	0^{sph,1} - 75,953^{gau,0}	0 (↔)	↓	↔ (↔**↔)
Leisler's bat	0.63	MaxEnt	714 (193)	0.64	MaxEnt	37,705 (232)	10,000^{4}	30,319^{0} - 356,068^{0}	2 (↑)	3,566^{sph,1} - 208,016^{sph,0}	0 (↔)	↓	* (****)
Pipistrelle	0.76	MaxEnt	1,886 (1,919)	0.67	RF	73,996 (9,645)	2,000,000^{3}	454,098^{sph,2} - 1,849,199^{sph,2}	-0.1 (↓)	416,237^{sph,1} - 782,758^{sph,0}	-0.61 (↓)	↓	↑ (↑**↑)
Nathusius' pipistrelle	0.64	RF	808 (214)	0.68	GLM	25,248 (257)	-	-	-	-	-	↓	* (****)
Barbastelle	0.66	SVM	555 (281)	0.67	MaxEnt	13,981 (546)	5,000^{5}	-	-	-	-	↓	* (****)
Brown long-eared bat	0.75	MaxEnt	1,784 (1,650)	0.65	RF	66,839 (7,691)	200,000^{4}	133,497^{sph,2} - 374,147^{sph,0}	0 (↔)	20,692^{sph,1} - 149,731^{sph,0}	-0.25 (↓)	↓	↔ (↔**↔)
Grey long-eared bat	0.79	MaxEnt	119 (38)	0.72	SVM	2,587 (89)	1,000^{3}	-	-	-	-	↓	* (****)
**Lagomorpha**												
Rabbit	0.84	RF	2,144 (2,539)	0.63	MaxEnt	113,968 (21,840)	37,500,000^{3}	2,069,527^{0} - 255,508,540^{0}	0 (↔)	97,105,355^{1} - 190,325,669^{sph,0}	1.59 (↑)	↑	↓ (↓↔↓*)
Brown hare	0.82	MaxEnt	2,059 (2,151)	0.62	RF	89,415 (16,497)	817,500^{2}	118,829^{sph,2} - 3,393,442^{sph,2}	0 (↔)	205,708^{sph,1} - 1,006,524^{sph,0}	0 (↔)	↓	↔ (↔↑↔*)
Mountain hare	0.77	GLM	665 (572)	0.68	GLM	17,614 (1,509)	350,000^{3}	2,633^{0} - 1,186,763^{0}	0 (↔)	25,078^{1} - 493,613^{sph,0}	0 (↔)	↑	↔ (↓↔↔*)
**Rodentia**												
Red squirrel	0.73	MaxEnt	1,295 (1,234)	0.66	-	45,944 (13,211)	160,000^{3}	305,073^{1} - 11,237,141^{sph,2}	0.91 (↑)	338,670^{sph,2} - 3,783,303^{sph,0}	1.12 (↑)	↓	* (****)
Grey squirrel	0.81	MaxEnt	1,870 (1,954)	0.64	MaxEnt	81,703 (20,797)	2,520,000^{3}	1,545,851^{0} - 14,511,831^{sph,2}	0 (↔)	826,807^{sph,1} - 6,216,107^{sph,0}	0 (↔)	↑	↑ (↑↑↔*)
Bank vole	0.72	MaxEnt	1,582 (1,459)	0.64	MaxEnt	61,694 (3325)	23,000,000^{3}	189,622^{0} - 204,426,956^{sph,2}	0 (↔)	963,410^{sph,0} - 70,220,233^{sph,0}	0 (↔)	*	* (****)
Skomer vole	-	-	- (0)	-	-	- (0)	7,000^{1}	-	-	-	-	*	* (****)
Field vole	0.67	MaxEnt	1,682 (1,851)	0.64	RF	65,876 (5,039)	75,000,000^{4}	4,875,844^{sph,2} - 463,671,721^{sph,2}	0 (↔)	17,915,165^{sph,0} - 142,267,687^{sph,0}	0 (↔)	↓	* (****)
Orkney vole	-	-	- (1)	1.00	MaxEnt	337 (1)	1,000,000^{1}	-	-	3,260,708^{sph,1} - 3,467,584^{sph,0}	2.26 (↑)	↓	* (****)
Water vole	0.72	MaxEnt	1,446 (1,609)	0.63	RF	52,594 (9,158)	1,169,000^{3}	544,441^{sph,2} - 7,995,892,846^{sph,1}	0 (↔)	28,718,646^{sph,0} - 2,832,451,796^{sph,0}	23.6 (↑)	↓	↓ (↓***)
Wood mouse	0.72	MaxEnt	1,790 (1,831)	0.65	MaxEnt	66,882 (5,134)	38,000,000^{3}	809,118^{sph,2} - 218,026,188^{sph,2}	0 (↔)	2,456,494^{sph,0} - 61,269,545^{sph,0}	0 (↔)	↔	* (****)
Yellow-necked mouse	0.67	MaxEnt	567 (320)	0.63	SVM	21,744 (524)	750,000^{4}	55,873^{0} - 592,033^{0}	-0.21 (↓)	99,308^{sph,0} - 653,113^{sph,0}	-0.13 (↓)	↓	* (****)
Harvest mouse	0.72	RF	1,006 (843)	0.63	MaxEnt	31,194 (1,737)	1,425,000^{5}	12,941^{sph,0} - 279,861^{sph,0}	-0.8 (↓)	38,755$^{\gamma,1}$ - 93,259^{sph,0}	-0.93 (↓)	↓	* (****)
House mouse	0.67	MaxEnt	771 (461)	0.68	MaxEnt	22,362 (1,074)	5,192,000^{5}	1,449^{sph,0} - 500,536^{gau,1}	-0.9 (↓)	45,514^{gau,0} - 137,375^{gau,1}	-0.97 (↓)	↓	* (****)
Common rat	0.77	MaxEnt	1,766 (1,888)	0.66	MaxEnt	59,755 (7,831)	6,790,000^{4}	1,724,587^{1} - 31,562,005^{1}	0 (↔)	5,829,264^{1} - 7,284,898^{sph,0}	0 (↔)	↓	↑ (↑↑**)
Ship rat	0.76	MaxEnt	475 (86)	0.77	MaxEnt	15,169 (64)	1,300^{2}	4,540,169^{0} - 5,984,207^{0}	3,491 (↑)	1,885,501^{sph,1} - 2,343,009^{gau,1}	1,449 (↑)	↓	* (****)
Common dormouse	0.75	MaxEnt	723 (621)	0.66	MaxEnt	26,992 (3,425)	500,000^{3}	24,721^{0} - 15,758,420^{0}	0 (↔)	-	-	↓	↓ (↓***)
Fat dormouse	0.79	MaxEnt	124 (32)	0.71	GLM	3,724 (149)	10,000^{3}	28,630^{0} - 2,914,000^{0}	1.86 (↑)	171,964^{0} - 875,133^{0}	16.2 (↑)	↑	* (****)
Coypu	0.74	SVM	266 (193)	0.72	GLM	3,932 (83)	0^{1}	-	-	-	-	*	* (****)

**Carnivora**													
Red fox	0.81	MaxEnt	1,841 (2,334)	0.61	RF	92,360 (16,433)	240,000[4]	69,625^{sph,2} - 385,710^{sph,2}	0 (↔)	85,909^{sph,2} - 253,986^{sph,0}	0 (↔)	↑	↔ (↔↑↓*)
Pine marten	0.70	MaxEnt	703 (495)	0.69	RF	18,465 (1,522)	3,650[2]	2,019^{1} - 25,177^{sph,0}	0 (↔)	1,610^{sph,1} - 6,181^{sph,0}	0 (↔)	↓	* (****)
Stoat	0.74	RF	1,822 (1,987)	0.64	MaxEnt	81,786 (7,856)	462,000[4]	-	-	-	-	↓	↑ (↔↑**)
Weasel	0.76	MaxEnt	1,796 (1,893)	0.64	MaxEnt	78,972 (6,515)	450,000[4]	1,056,431^{sph,2} - 24,999,091^{sph,1}	1.35 (↑)	229,804^{sph,1} - 11,228,731^{sph,0}	0 (↔)	↓	↑ (↔↑**)
Polecat	0.73	RF	954 (682)	0.63	RF	58,016 (2,730)	15,000[3]	52,011^{sph,0} - 53,475^{sph,0}	2.47 (↑)	41,966^{sph,1} - 49,045^{sph,0}	1.8 (↑)	↑	↔ (↑↔**)
Feral ferret	0.65	MaxEnt	978 (353)	0.66	GLM	34,400 (295)	2,500[5]	-	-	-	-	*	* (****)
American mink	0.69	RF	1,587 (1,568)	0.67	RF	59,352 (4,293)	110,000[3]	13,714^{0} - 2,724,393^{0}	0 (↔)	24,501^{sph,1} - 1,843,372^{sph,0}	0 (↔)	↑	↓ (↔↓**)
Badger	0.79	MaxEnt	1,908 (2,149)	0.62	RF	83,121 (12,510)	250,000[1]	79,544^{sph,2} - 968,740^{sph,2}	0 (↔)	112,871^{sph,2} - 419,782^{sph,0}	0 (↔)	↑	↑ (↑***)
Otter	0.77	RF	1,877 (2,345)	0.68	RF	88,110 (14,403)	7,350[3]	155,399^{1} - 716,216^{0}	20.1 (↑)	313,333^{sph,2} - 375,780^{sph,1}	41.6 (↑)	↑	↑ (↑***)
Wildcat	0.74	MaxEnt	361 (265)	0.66	MaxEnt	12,896 (352)	3,500[3]	1,740^{0} - 16,255^{sph,0}	0 (↔)	232^{sph,1} - 6,155^{sph,0}	0 (↔)	↓	* (****)
Feral cat	0.68	MaxEnt	1,134 (794)	0.67	MaxEnt	46,383 (1,371)	813,000[4]	254,430^{sph,1} - 22,644,848^{sph,1}	0 (↔)	500,375^{sph,0} - 3,506,530^{sph,0}	0 (↔)	↑	↔ (*↔**)
**Artiodactyla**													
Red deer	0.72	RF	1,146 (1,277)	0.63	RF	62,762 (3,604)	360,000[2]	379,297^{sph,2} - 780,812^{sph,2}	0.05 (↑)	338,877^{sph,2} - 501,909^{sph,0}	0 (↔)	↑	↔ (↑↔↔*)
Sika deer	0.70	MaxEnt	748 (436)	0.64	RF	27,328 (544)	11,500[2]	21,477^{sph,2} - 357,800^{sph,2}	0.87 (↑)	12,941^{sph,1} - 71,514^{sph,0}	0.13 (↑)	↑	↑ (↑↑**)
Fallow deer	0.71	RF	1,142 (1,045)	0.65	MaxEnt	34,201 (2,134)	100,000[4]	303,314^{0} - 4,635,453^{0}	2.03 (↑)	205,263^{sph,1} - 1,451,662^{sph,2}	1.05 (↑)	↑	↔ (↑↔↔*)
Roe deer	0.76	RF	1,754 (2,047)	0.64	RF	78,398 (10,853)	500,000[3]	563,932^{0} - 4,500,284^{sph,2}	0.13 (↑)	25,296^{sph,2} - 2,185,884^{sph,0}	0 (↔)	↑	↑ (↑↑↑*)
Chinese muntjac	0.75	MaxEnt	964 (990)	0.66	GLM	33,605 (4480)	40,000[3]	1,962,152^{1} - 5,046,501^{sph,1}	48.1 (↑)	169,848^{sph,1} - 1,844,193^{sph,0}	3.25 (↑)	↑	↑ (↑↑↑*)
Chinese water deer	0.71	GLM	285 (144)	0.68	GLM	5,278 (714)	650[2]	17,781^{0} - 808,932^{0}	26.4 (↑)	53,527$^{\gamma,0}$ - 156,711^{sph,0}	81.3 (↑)	↑	↑ (↑***)
Park cattle	-	-	- (8)	0.90	MaxEnt	20,162 (7)	45[1]	-	-	179,314^{0} - 735,333$^{\gamma,0}$	3,259 (↑)	*	* (****)
Feral goat	0.66	RF	430 (137)	0.67	SVM	14,388 (191)	3,565[2]	23,871^{0} - 163,909^{sph,2}	5.7 (↑)	3,269^{sph,2} - 65,463^{sph,0}	0 (↔)	*	* (****)
Feral sheep	0.72	MaxEnt	492 (29)	0.74	BioClim	40,473 (25)	2,100[1]	447,819^{0} - 3,398,746^{sph,0}	212 (↑)	0^{sph,0} - 3,004,178^{sph,0}	0 (↔)	*	* (****)
Wild boar	0.80	MaxEnt	179 (23)	0.70	Domain	10,129 (48)	-	188^{sph,0} - 3,011^{sph,0}	-	491^{sph,0} - 1,850^{sph,0}	-	*	* (****)

[1] Harris, S., P. Morris, S. Wray and D. W. Yalden (1995). A review of British mammals: population estimates and conservation status of British mammals other than cetaceans, JNCC.

[2] Battersby, J. and Tracking Mammals Partnership (2005). UK mammals: species status and population trends. First report by the Tracking Mammals Partnership, JNCC/Tracking Mammals Partnership, Peterborough.

[3] Aebischer,N.J., P. D. Davey and N. G. Kingdon (2011). National Gamebag Census: Mammal Trends to 2009. Game & Wildlife Conservation Trust, Fordingbridge. http://www.gwct.org.uk/ngcmammals.

[4] Harris, S., D. Massimino, S. E. Newson, M. A. Eaton, D. E. Balmer, D. G. Noble, A. J. Musgrove, S. Gillings, D. Procter and J .W. Pearce-Higgins (2015). The Breeding Bird Survey 2014. BTO Research Report 673. British Trust for Ornithology, Thetford.

[5] Bat Conservation Trust (2014). Species population trends. http://www.bats.org.uk/pages/species_population_trends.html.

S2.2 Summary maps grouping species by order, origin and status

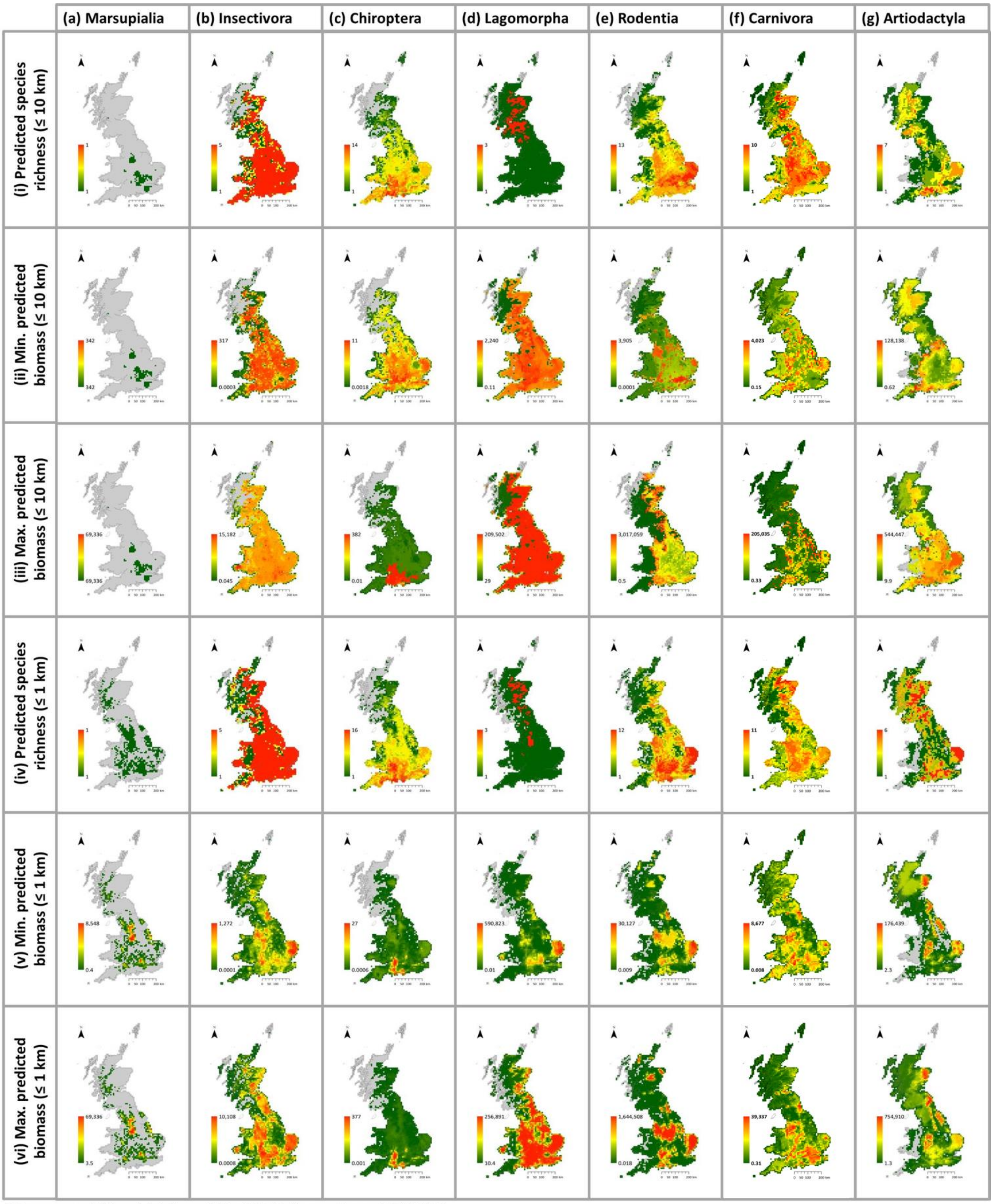

©Crown copyright and database rights 2016 Ordnance Survey 100051110.

Data courtesy of the NBN Gateway with thanks to all the data contributors. The NBN and its data contributors bear no responsibility for the further analysis or interpretation of this material, data and/or information.

Fig A. Summary maps showing predicted species richness and range of total biomass categorised by mammalian order for model outputs generated based on raster grids with a resolution of: (i)-(iii) 10 km (using all occurrence data); and (iv)-(vi) 1 km (using only occurrence data recorded at a resolution of 1 km or higher); respectively.

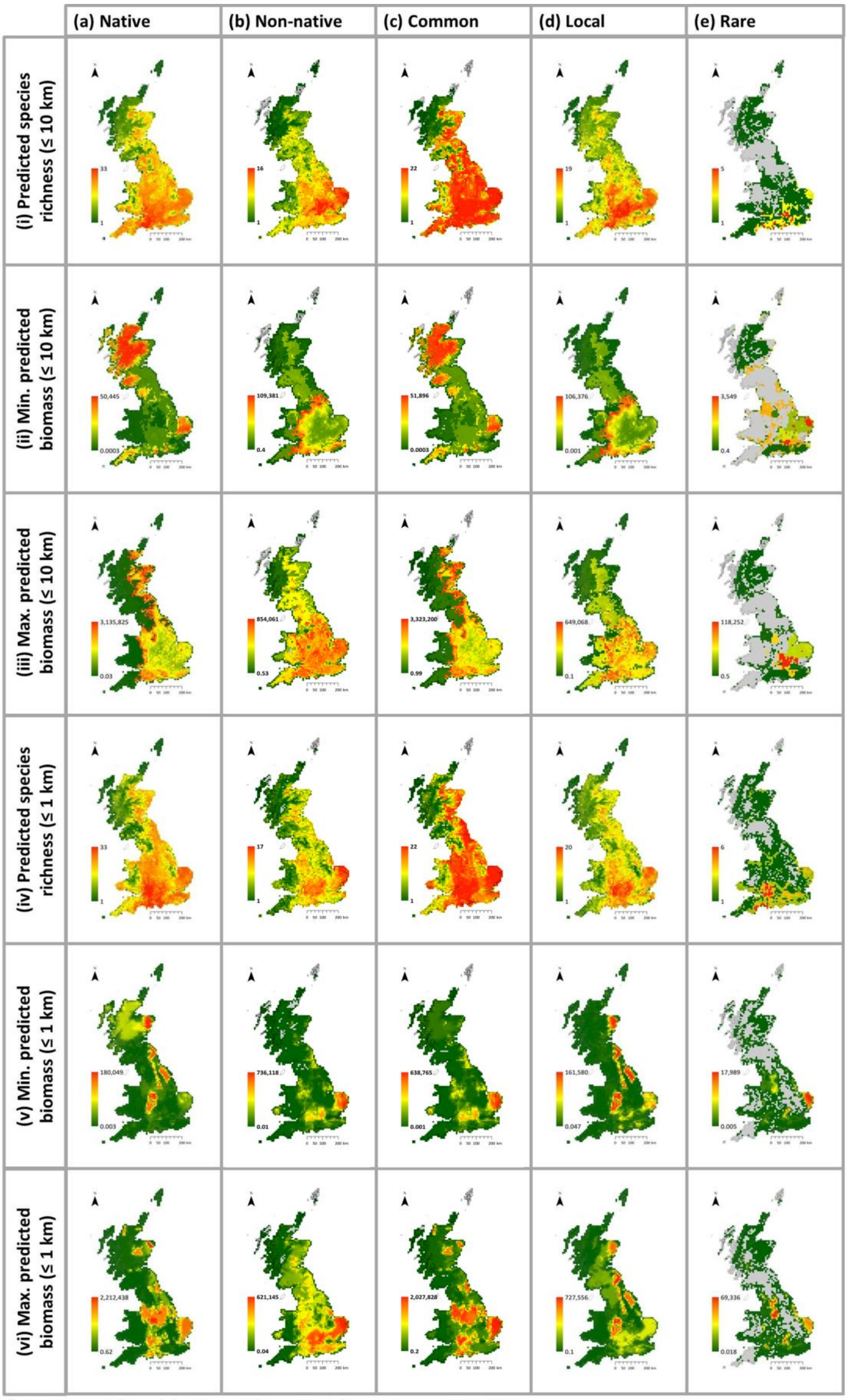

©Crown copyright and database rights 2016 Ordnance Survey 100051110.

Data courtesy of the NBN Gateway with thanks to all the data contributors. The NBN and its data contributors bear no responsibility for the further analysis or interpretation of this material, data and/or information.

Fig B. Summary maps showing predicted species richness and range of total biomass categorised by origin and status for model outputs generated based on raster grids with a resolution of: (i)-(iii) 10 km (using all occurrence data); and (iv)-(vi) 1 km (using only occurrence data recorded at a resolution of 1 km or higher); respectively.
