# Supplementary material for: A systematic approach to estimate the distribution and total abundance of British mammals
Source: PLoS One. 2017 Jun 28;12(6):e0176339. doi: 10.1371/journal.pone.0176339 (PMC5489149; doi:10.1371/journal.pone.0176339)
Supplement: S3 File — Individual reports for each of the Artiodactyla species presenting analysis of the available data and subsequent model predictions based on a 10km raster grid. Reports also include expert comment assessing the reliability (and plausibility) of results in the context of existing evidence and popular opinion. (ZIP) [file pone.0176339.s003.zip › A Chinese muntjac.pdf]

Chinese muntjac (*Muntiacus reevesi*)

Order: *Artiodactyla*

Genus: *Muntiacus*

Origin: Introduced

Status: Locally common

1995 abundance estimate: 40,000 (3)

Reported population trends: JNCC 2005, NGC 2009, BBS 2014 (↑)

Data:

The available occurrence records indicate that the distribution of Chinese muntjac is concentrated in central and south eastern regions of England with observations becoming more patchy towards the north and west (Figure 1a). These sightings were reported in various habitats (predominantly arable and improved grassland) with the majority of grid cells where occurrence was observed containing at least one record from the past decade.

From the literature review we identified several density estimates (Cooke & Farrell 2001; Hemami et al. 2005, 2007) located within south east of England spanning approximately 1% of the observed species distribution based on the available occurrence data (Figure 1b). Most of these estimates related to arable dominated land cover and consequently densities for many of the land covers where occurrence had been reported were unavailable (land class marked grey in Table 1). Estimates ranged between 45 and 64 per km^2 with the highest densities recorded in broadleaved woodland (7.82 - 54.88 per km^2 accounting for uncertainty relating to unsurveyed areas within grid cells).

Model predictions:

The habitat suitability map (Figure 2a) appears to reflect the underlying data well with the set of "best" models predicting presence (and absence) to a mean AUC of 0.77. Overall, across 100 repetitions MaxEnt proved to be the most commonly selected modelling approach displaying the highest AUC 45% of the time followed by Generalised Linear Models (23%) and Random Forest (20%). By land cover the mean habitat suitability scores suggest observation is most likely in landscapes dominated by calcareous grassland and broadleaved woodland (Table 1) but, consistent with recorded sightings, the majority of occurrence is predicted in grid cells dominated by arable and improved grassland.

Both minimum and maximum density estimates were best fitted linearly to habitat suitability with the latter accounting for spherical spatial autocorrelation. However, interestingly the relationship with minimum density shows a negative correlation predicting the highest abundance towards the edge of the distribution (this could be considered plausible for a rapidly expanding species but is unlikely in this case as muntjac are not more frequently observed at the range edge). This explains the disparity between the predictions with the gap in the central part of the minimum abundance map indicating an area where high habitat suitability lead to negative densities (these were consequently removed). In order to resolve this difference more density estimates across a greater breadth of habitat types are required.

The predicted abundance range does not contain the estimate from Harris et al. (1995). Instead, the range suggests a significant increase which could be explained by recently reported population trends (JNCC, NGC and BBS). However, the magnitude of this increase is unlikely particularly as the median year of density reporting was 2002; hence either densities would have to grow substantially over the 7 years and/or the species range would have to have expanded by at least 50 times (this is not possible within the extent of GB).

Reliability (Expert comment):

Occurrence is very unlikely in Scotland; if these observations were correct, muntjac are almost certainly absent from the wild now. The density estimates obtained from the literature represent particularly high density sites which are perhaps unrealistic over a broad spatial unit of 10km. Consequently, the range of values is unrepresentatively high compared with that which should be expected at a national level.

The predicted habitat suitability map suggests a plausible, if not a slightly conservative, distribution. The association with broadleaved woodland is consistent with expectation but there is little evidence to support any direct dependence on calcareous grassland (may simply be the coincidental consequence of occurrence in the South Downs). Despite recent trends indicating an increase in population size the predicted abundance range is unrealistically high. Clearly this is due to the unrepresentatively high density estimates which were available. In order to provide a more balanced representation of the national density range studies are required for areas of lower abundance.

References:

Cooke, A. S. and L. Farrell (2001). Impact of muntjac deer (*Muntiacus reevesi*) at Monks Wood National Nature Reserve, Cambridgeshire, eastern England. *Forestry* 74(3): 241-250.

Harris, S. J., P. Morris, S. Wray and D. Yalden (1995). A review of British mammals: population estimates and conservation status of British mammals other than cetaceans, Joint Nature Conservation Committee, Peterborough, UK.

Hemami, M. R., A. R. Watkinson and P. M. Dolman (2005). Population densities and habitat associations of introduced muntjac *Muntiacus reevesi* and native roe deer *Capreolus capreolus* in a lowland pine forest. *Forest Ecology and Management* 215(1–3): 224-238.

Hemami, M. R., A. R. Watkinson, R. M. A. Gill and P. M. Dolman (2007). Estimating abundance of introduced Chinese muntjac *Muntiacus reevesi* and native roe deer *Capreolus capreolus* using portable thermal imaging equipment. *Mammal Review* 37(3): 246-254.

Table 1: Summary of observed data and model predictions by land cover class (LCM2007 target classification). Values shown in brackets denote the spatial coverage based on a 10km resolution raster map (number of grid cells). Years represent the median of records within each land class. Ranges for density and abundance are derived using the respective minimum and maximum raster maps (lower bound is mean of values across minimum raster map with upper across the maximum) which capture the spatial uncertainty generate by projecting irregular polygons describing survey sites onto a raster grid.

**LCM2007 class**	**Observed**					**Predicted**		
	**Occurrence**		**Density**					
	**Records**	**Year**	**Estimates**	**Year**	**Range**	**Habitat suitability**	**Density**	**Abundance**
1 (Broadleaved woodland)	56 (11)	2005	0 (0)	-	-	0.87 (9)	7.8 - 54.9	7,042 - 49,389
2 (Coniferous woodland)	384 (7)	2001	1 (1)	2002	36 - 64	0.19 (2)	32.1 - 54.3	6411 - 10,864
3 (Arable and Horticultural)	13,589 (640)	2012	12 (11)	2002	6.1 - 57.1	0.76 (688)	16.6 - 52.2	1,140,579 - 3,593,653
4 (Improved grassland)	2,022 (248)	2000	0 (0)	-	-	0.52 (212)	30.9 - 52.5	655,077 - 1,113,802
5 (Rough grassland)	167 (2)	2004	2 (1)	2002	12.2 - 64	0.14 (1)	16.5 - 54.7	1,648 - 5,466
6 (Neutral grassland)	0 (0)	-	0 (0)	-	-	0.01 (0)	-	0
7 (Calcareous grassland)	7 (2)	1999	0 (0)	-	-	0.88 (2)	16.3 - 54.7	3,262 - 10,932
8 (Acid grassland)	34 (19)	2000	0 (0)	-	-	0.24 (4)	52.5 - 53.9	20,997 - 21,553
9 (Fen, Marsh, and Swamp)	0 (0)	-	0 (0)	-	-	-	-	0
10 (Heather)	2 (2)	2000	0 (0)	-	-	0.18 (0)	-	0
11 (Heather grassland)	5 (3)	2005	0 (0)	-	-	0.11 (1)	48.4 - 54	4,837 - 5,397
12 (Bog)	2 (1)	2000	0 (0)	-	-	0.1 (0)	-	0
13 (Montane habitat)	0 (0)	-	0 (0)	-	-	0.09 (0)	-	0
14 (Inland rock)	0 (0)	-	0 (0)	-	-	0.05 (0)	-	0
15 (Saltwater)	4 (1)	1969	0 (0)	-	-	0.44 (0)	-	0
16 (Freshwater)	0 (0)	-	0 (0)	-	-	0.15 (0)	-	0
17 (Supra-littoral rock)	0 (0)	-	0 (0)	-	-	0.03 (0)	-	0
18 (Supra-littoral sediment)	0 (0)	-	0 (0)	-	-	0.26 (0)	-	0
19 (Littoral rock)	0 (0)	-	0 (0)	-	-	0.21 (0)	-	0
20 (Littoral sediment)	7 (5)	1998	0 (0)	-	-	0.44 (1)	41.5 - 42.5	4,149 - 4,250
21 (Saltmarsh)	0 (0)	-	0 (0)	-	-	-	-	0
22 (Urban)	22 (4)	2003	0 (0)	-	-	0.68 (4)	27.5 - 54.4	10,979 - 21,769
23 (Suburban)	334 (45)	2010	0 (0)	-	-	0.69 (40)	26.8 - 52.4	107,172 - 209,425
Total	16,635 (990)	2010	15 (13)	2002	8.8 - 58.2	0.49 (964)	20.4 - 52.4	1,962,152 - 5,046,501

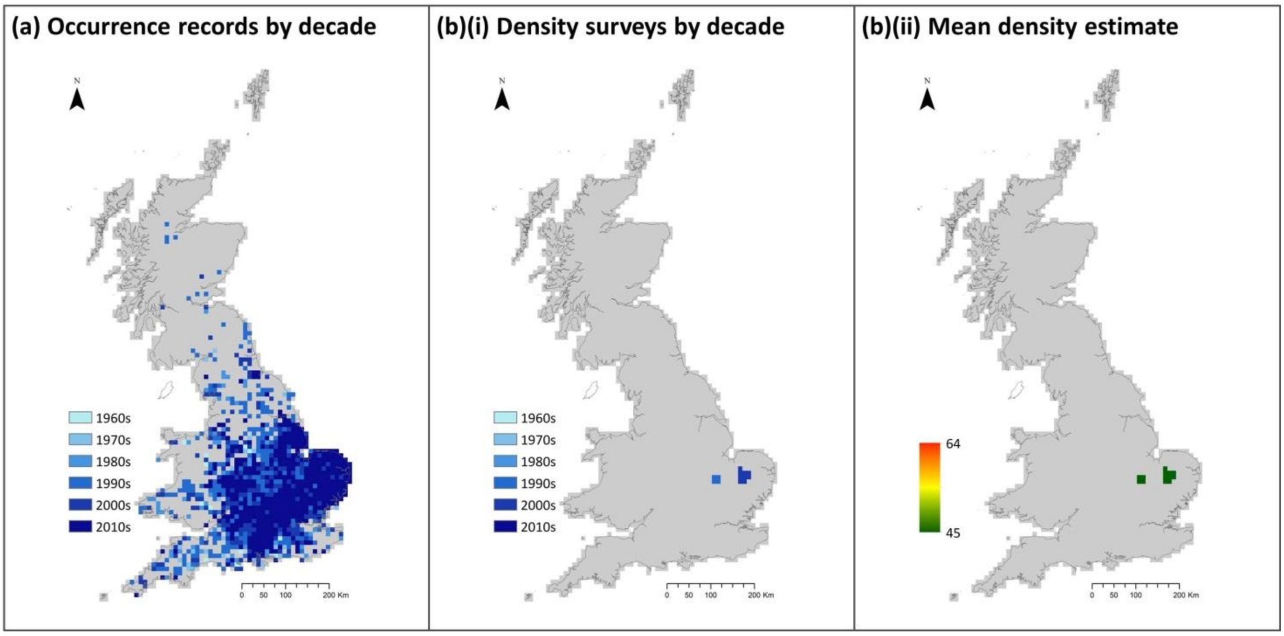

© Crown copyright and database rights 2016 Ordnance Survey 100051110. Data courtesy of the NBN Gateway with thanks to all data contributors. The NBN and its data contributors bear no responsibility for the further analysis or interpretation of this material, data and/or information.

Figure 1: 10km resolution raster maps based on BNG presenting the geographic description of available data. (a) shows the distribution of species occurrence obtained via the NBN Gateway categorised by the decade of last sighting. (b) shows information relating to density surveys identified via a search of published literature where: (i) categorises surveys by the decade of last survey; and (ii) shows the mean density estimate of surveys within grid cells (estimates assumed to be representative of entire cell, considered the upper limit of observed density).

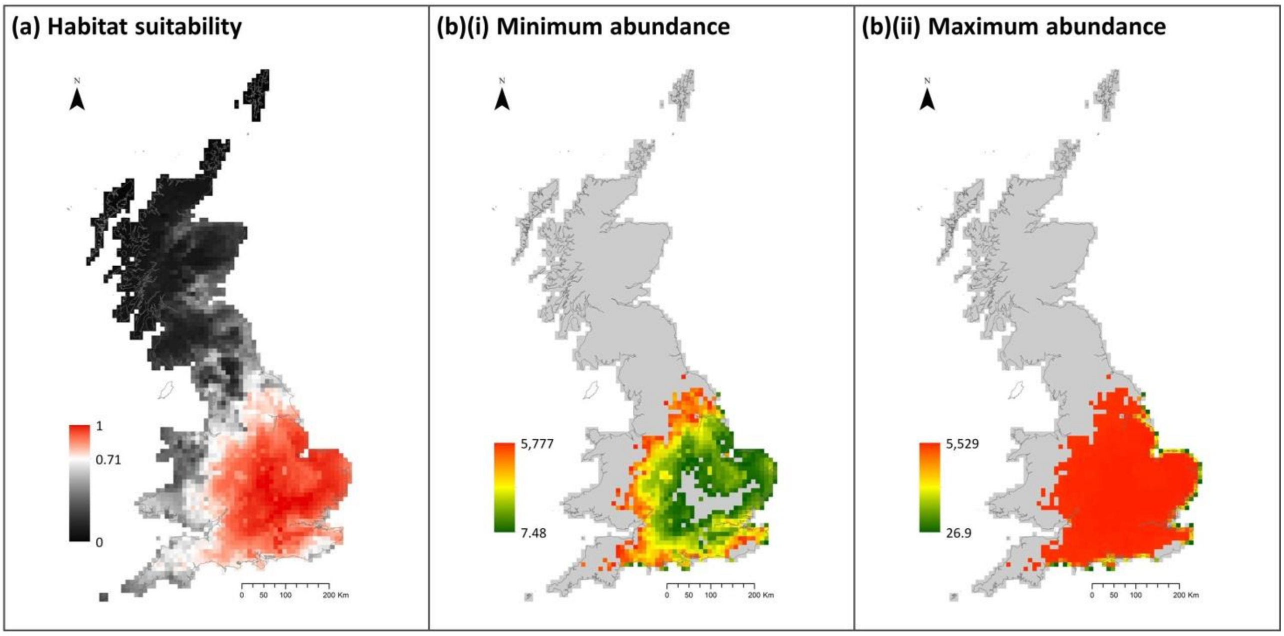

© Crown copyright and database rights 2016 Ordnance Survey 100051110. Data courtesy of the NBN Gateway with thanks to all data contributors. The NBN and its data contributors bear no responsibility for the further analysis or interpretation of this material, data and/or information.

Figure 2: Modelling predictions generated using systematic approach based on available data. (a) shows habitat suitability scores (the likelihood of observing the target species within each grid cell given variation environmental variables) determined by aggregating outputs from the "best" species distribution model (7 models compared) across 100 simulations. Here, the mid value on the scale denotes the threshold score above which occurrence is assumed. (b) shows: (i) the lower bound (Minimum); and (ii) the upper bound (Maximum); of abundance estimates determined by relating observed density (taking into account potential uncertainty) with habitat suitability scores using linear regression.
