# Supplementary material for: A systematic approach to estimate the distribution and total abundance of British mammals
Source: PLoS One. 2017 Jun 28;12(6):e0176339. doi: 10.1371/journal.pone.0176339 (PMC5489149; doi:10.1371/journal.pone.0176339)
Supplement: S3 File — Individual reports for each of the Artiodactyla species presenting analysis of the available data and subsequent model predictions based on a 10km raster grid. Reports also include expert comment assessing the reliability (and plausibility) of results in the context of existing evidence and popular opinion. (ZIP) [file pone.0176339.s003.zip › B Chinese water deer.pdf]

Chinese water deer (*Hydropotes inermis*)

Order: *Artiodactyla*

Genus: *Hydropotes*

Origin: Introduced

Status: Rare

1995 abundance estimate: 650 (2)

Reported population trends: JNCC 2005 (↑)

Data:

The available occurrence records indicate that Chinese water deer are most commonly sighted in arable dominated habitats across patches of East Anglia and the south east Midlands with some scattered sightings towards the north and south west (Figure 1a).

From the literature review we identified a single density estimate of 30 per km^2 (Harris et al. 1995) reported on arable dominated habitat in 1991 (Figure 1b). Taking to account the uncertainty relating to study area when projected onto a 10km raster grid the range of densities was large (0.66 - 30 per km^2).

Model predictions:

The habitat suitability map (Figure 2a) appears to reflect the underlying data reasonably well with the set of "best" models predicting presence (and absence) to a mean AUC of 0.71. However, the resulting distribution covers an area approximately twice that of the observations alone (Figure 1a). Overall, across 100 repetitions Generalised Linear Models proved to be the most commonly selected modelling approach displaying the highest AUC 32% of the time closely followed by MaxEnt (27%). By land cover the mean habitat suitability scores suggest observation is most likely in landscapes dominated by arable land (Table 1) and, consistent with recorded sightings, the majority of occurrence is predicted in grid cells dominated by arable and improved grassland.

Due to the limited number of density estimates it was not possible to assess any relationship with habitat suitability. Instead, a constant mean estimate was applied to all cells where occurrence was predicted and summed to derive total abundance.

The predicted abundance range did not contain the 1995 the estimate from Harris et al. (1995). Instead, our predictions suggest a significant increase which could be explained by recently reported population trends (JNCC). However, the magnitude of the increase makes this unlikely particularly as the median density estimate was reported in 1991; hence such a change could only be produced by a range expansion of approximately 25 times (this is not possible within the extent of GB).

Reliability (Expert comment):

Whilst the core distribution suggested by the observations of occurrence appears plausible the scattered outliers towards the edge of the range most likely reflect attempted introductions and not established populations. The density estimate obtained from the literature undoubtedly reflects a high density site which is unrepresentative of the national average.

The distribution suggested by the habitat suitability map is reasonable, if a little conservative, and the expanded coverage is not implausible, especially considering the time period over which analysis is conducted and the potential for under-reporting of species occurrence. The association with landscapes dominated by arable land cover is not unsurprising, particularly in the context of most likely observation.

Despite reports of an increase in population over recent years the predicted abundance range is unrealistically large and the maps lack plausible spatial variation. Both of these factors highlight limitations of the modelling process, in particular a reliance on obtaining a representative range of density estimates which are not available in this case.

References:

Harris, S. J., P. Morris, S. Wray and D. Yalden (1995). A review of British mammals: population estimates and conservation status of British mammals other than cetaceans, Joint Nature Conservation Committee, Peterborough, UK.

Table 1: Summary of observed data and model predictions by land cover class (LCM2007 target classification). Values shown in brackets denote the spatial coverage based on a 10km resolution raster map (number of grid cells). Years represent the median of records within each land class. Ranges for density and abundance are derived using the respective minimum and maximum raster maps (lower bound is mean of values across minimum raster map with upper across the maximum) which capture the spatial uncertainty generate by projecting irregular polygons describing survey sites onto a raster grid.

LCM2007 class	Observed					Predicted		
	Occurrence		Density					
	Records	Year	Estimates	Year	Range	Habitat suitability	Density	Abundance
1 (Broadleaved woodland)	2 (1)	1998	0 (0)	-	-	0.39 (1)	0.66 - 30	65.9 - 3,000
2 (Coniferous woodland)	10 (1)	2000	0 (0)	-	-	0.18 (1)	0.66 - 30	65.9 - 3,000
3 (Arable and Horticultural)	3,827 (122)	2009	1 (1)	1991	0.66 - 30	0.49 (258)	0.62 - 28.3	16,062 - 730,725
4 (Improved grassland)	105 (14)	2004	0 (0)	-	-	0.28 (20)	0.63 - 28.6	1,257 - 57,206
5 (Rough grassland)	3 (1)	1990	0 (0)	-	-	0.16 (1)	0.66 - 30	65.9 - 3,000
6 (Neutral grassland)	0 (0)	-	0 (0)	-	-	0.07 (0)	-	0
7 (Calcareous grassland)	0 (0)	-	0 (0)	-	-	0.42 (0)	-	0
8 (Acid grassland)	0 (0)	-	0 (0)	-	-	0.15 (0)	-	0
9 (Fen, Marsh, and Swamp)	0 (0)	-	0 (0)	-	-	-	-	0
10 (Heather)	0 (0)	-	0 (0)	-	-	0.21 (0)	-	0
11 (Heather grassland)	0 (0)	-	0 (0)	-	-	0.15 (0)	-	0
12 (Bog)	0 (0)	-	0 (0)	-	-	0.17 (0)	-	0
13 (Montane habitat)	0 (0)	-	0 (0)	-	-	0.14 (0)	-	0
14 (Inland rock)	0 (0)	-	0 (0)	-	-	0.11 (0)	-	0
15 (Saltwater)	0 (0)	-	0 (0)	-	-	0.28 (0)	-	0
16 (Freshwater)	0 (0)	-	0 (0)	-	-	0.11 (0)	-	0
17 (Supra-littoral rock)	0 (0)	-	0 (0)	-	-	0.15 (0)	-	0
18 (Supra-littoral sediment)	0 (0)	-	0 (0)	-	-	0.23 (0)	-	0
19 (Littoral rock)	0 (0)	-	0 (0)	-	-	0.21 (0)	-	0
20 (Littoral sediment)	0 (0)	-	0 (0)	-	-	0.32 (0)	-	0
21 (Saltmarsh)	0 (0)	-	0 (0)	-	-	-	-	0
22 (Urban)	0 (0)	-	0 (0)	-	-	0.3 (0)	-	0
23 (Suburban)	7 (5)	1972	0 (0)	-	-	0.34 (4)	0.66 - 30	264 - 12,000
Total	3,954 (144)	2008	1 (1)	1991	0.66 - 30	0.32 (285)	0.6 - 28.4	17,781 - 808,932

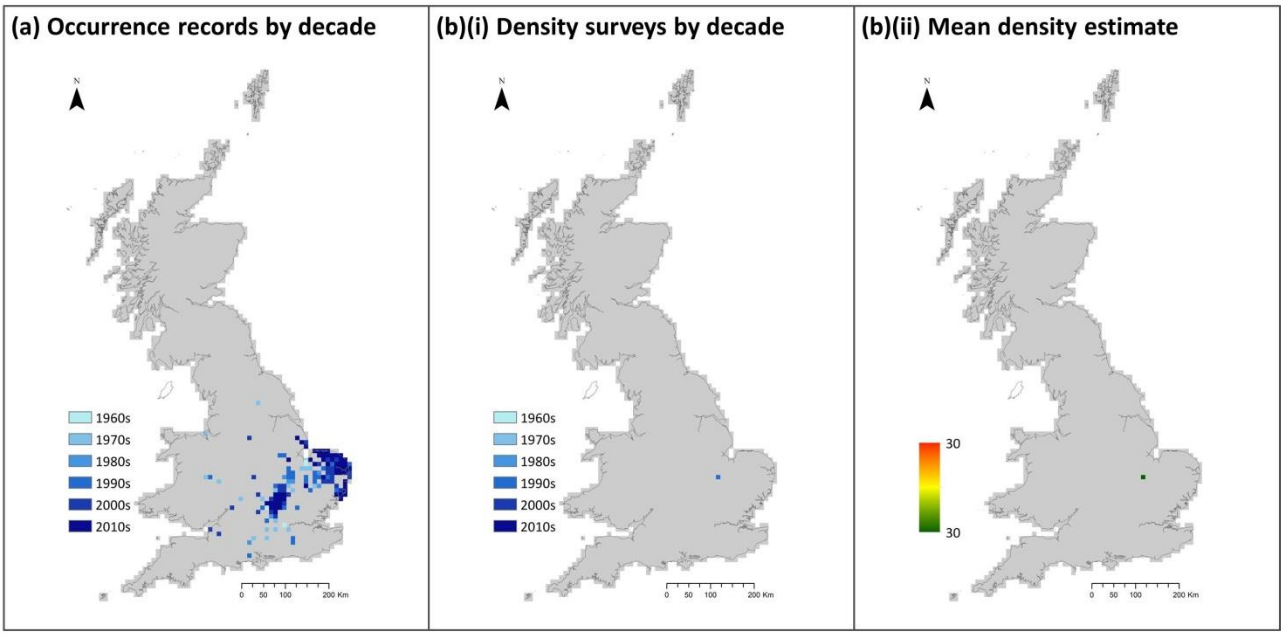

© Crown copyright and database rights 2016 Ordnance Survey 100051110. Data courtesy of the NBN Gateway with thanks to all data contributors. The NBN and its data contributors bear no responsibility for the further analysis or interpretation of this material, data and/or information.

Figure 1: 10km resolution raster maps based on BNG presenting the geographic description of available data. (a) shows the distribution of species occurrence obtained via the NBN Gateway categorised by the decade of last sighting. (b) shows information relating to density surveys identified via a search of published literature where: (i) categorises surveys by the decade of last survey; and (ii) shows the mean density estimate of surveys within grid cells (estimates assumed to be representative of entire cell, considered the upper limit of observed density).

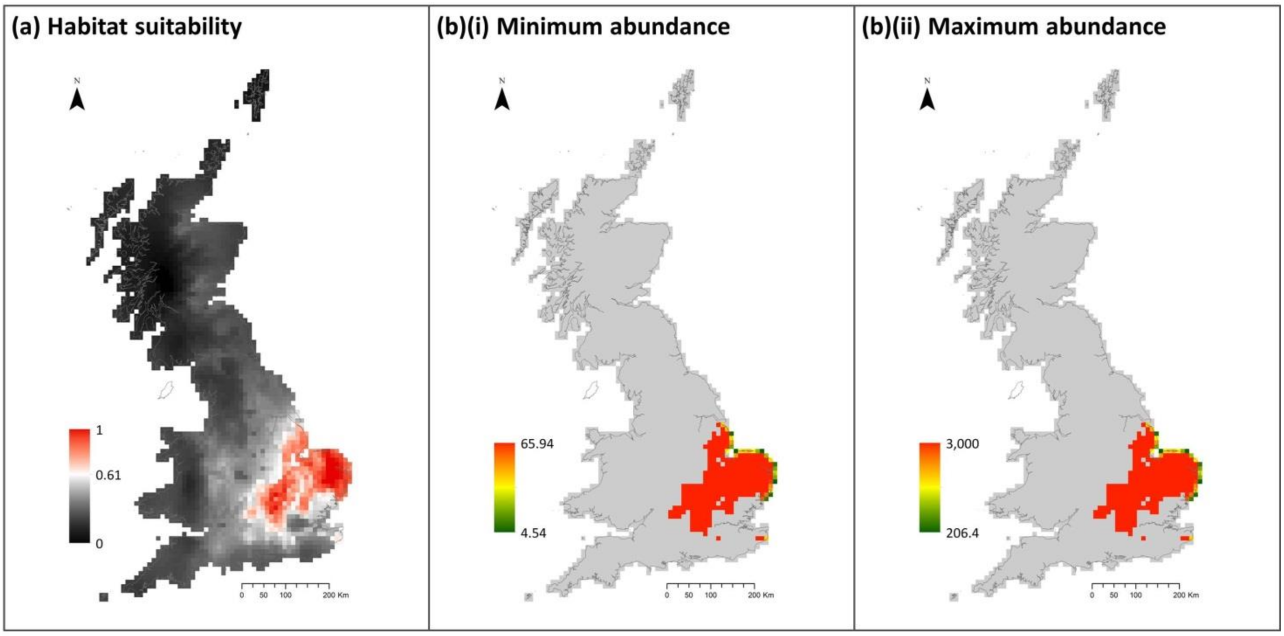

© Crown copyright and database rights 2016 Ordnance Survey 100051110. Data courtesy of the NBN Gateway with thanks to all data contributors. The NBN and its data contributors bear no responsibility for the further analysis or interpretation of this material, data and/or information.

Figure 2: Modelling predictions generated using systematic approach based on available data. (a) shows habitat suitability scores (the likelihood of observing the target species within each grid cell given variation environmental variables) determined by aggregating outputs from the "best" species distribution model (7 models compared) across 100 simulations. Here, the mid value on the scale denotes the threshold score above which occurrence is assumed. (b) shows: (i) the lower bound (Minimum); and (ii) the upper bound (Maximum); of abundance estimates determined by relating observed density (taking into account potential uncertainty) with habitat suitability scores using linear regression.
