# Supplementary material for: A systematic approach to estimate the distribution and total abundance of British mammals
Source: PLoS One. 2017 Jun 28;12(6):e0176339. doi: 10.1371/journal.pone.0176339 (PMC5489149; doi:10.1371/journal.pone.0176339)
Supplement: S3 File — Individual reports for each of the Artiodactyla species presenting analysis of the available data and subsequent model predictions based on a 10km raster grid. Reports also include expert comment assessing the reliability (and plausibility) of results in the context of existing evidence and popular opinion. (ZIP) [file pone.0176339.s003.zip › C Fallow deer.pdf]

Fallow deer (*Dama dama*)

Order: *Artiodactyla*

Genus: *Dama*

Origin: Introduced

Status: Locally common

1995 abundance estimate: 100,000 (4)

Reported population trends: JNCC 2005 (↑), NGC 2009, BBS 2014 (↔)

Data:

The available occurrence records indicate that fallow deer are most widely observed in southern regions of England and in Wales with a few isolated patches of occurrence reported through northern England and Scotland (Figure 1a). In general, sightings were reported in various habitats (predominantly arable and improved grassland) the majority of which since 1995.

From the literature review we identified a single survey (Gill et al. 1997) conducted near the Welsh border in 1994 which estimated density to be between 41.2 and 45 per km^2 (Figure 1b). This survey only sampled a small area dominated by arable and improved grassland, consequently, no estimates were available for other habitats where occurrence was observed (marked grey in Table 1).

Model predictions:

The habitat suitability map (Figure 2a) appears to reflect the underlying data well with the set of “best” models predicting presence (and absence) to a mean AUC of 0.71. However, the resulting distribution notably does not capture the more isolated patches of occurrence recorded in Scotland. Overall, across 100 repetitions Random Forest proved to be the most commonly selected modelling approach displaying the highest AUC 35% of the time closely followed by MaxEnt (33%). By land cover the mean habitat suitability scores suggest observation is most likely in landscapes dominated by broadleaved woodland (Table 1) but, consistent with recorded sightings, the majority of occurrence is predicted in grid cells dominated by arable and improved grassland.

Due to the limited number of density estimates it was not possible to assess any relationship with habitat suitability. Instead, a constant mean estimate was applied to all cells where occurrence was predicted and summed to derive total abundance.

This predicted abundance range does not contain the estimate from Harris et al. (1995). Instead, consistent with the reported trend by JNCC in 2005, our predictions suggest a significant increase in population. If large enough such an increase may explain the overestimation. However, the most recent trends from NGC and BBS do not suggest any change in population since 1995 and therefore this result is most likely due to the use of a limited number of high density estimates; particularly given the magnitude of the increase which would suggest population growth of at least 3 times over 20 years (since the median density estimate is from 1994 this could only be caused by a substantial range expansion which is unlikely).

Reliability (Expert comment):

Observations appear to be over-recorded in Scotland and northern England where only small isolated populations are present. The density estimates obtained from the literature are limited and only represent the upper end of densities across the geographic range.

The distribution suggested by the habitat suitability map is perhaps more plausible although it could be argued that this instead under-represents the occurrence in Scotland perhaps due to the dominance of records in more southerly regions. The range of predicted abundance is large highlighting the uncertainty associated with the estimates; the upper estimate is unrealistic but the lower estimate is more plausible albeit towards the upper limit of what is likely.

References:

Gill, R. M. A., M. L. Thomas and D. Stocker (1997). The use of portable thermal imaging for estimating deer population density in forest habitats. *Journal of Applied Ecology* 34(5): 1273-1286.

Harris, S. J., P. Morris, S. Wray and D. Yalden (1995). A review of British mammals: population estimates and conservation status of British mammals other than cetaceans, Joint Nature Conservation Committee, Peterborough, UK.

Table 1: Summary of observed data and model predictions by land cover class (LCM2007 target classification). Values shown in brackets denote the spatial coverage based on a 10km resolution raster map (number of grid cells). Years represent the median of records within each land class. Ranges for density and abundance are derived using the respective minimum and maximum raster maps (lower bound is mean of values across minimum raster map with upper across the maximum) which capture the spatial uncertainty generate by projecting irregular polygons describing survey sites onto a raster grid.

LCM2007 class	Observed					Predicted		
	Occurrence		Density					
	Records	Year	Estimates	Year	Range	Habitat suitability	Density	Abundance
1 (Broadleaved woodland)	164 (10)	2010	0 (0)	-	-	0.93 (11)	2.76 - 42.2	3,038 - 46,423
2 (Coniferous woodland)	368 (41)	2000	0 (0)	-	-	0.53 (13)	2.76 - 42.2	3,590 - 54,863
3 (Arable and Horticultural)	5,781 (586)	2000	6 (4)	1994	3.39 - 41.5	0.8 (712)	2.64 - 40.3	187,625 - 2,867,415
4 (Improved grassland)	1,766 (299)	2001	2 (1)	1994	0.27 - 45	0.66 (351)	2.68 - 40.9	93,925 - 1,435,428
5 (Rough grassland)	16 (6)	2000	0 (0)	-	-	0.23 (4)	2.74 - 41.8	1,094 - 16,723
6 (Neutral grassland)	0 (0)	-	0 (0)	-	-	0 (0)	-	0
7 (Calcareous grassland)	0 (0)	-	0 (0)	-	-	0.61 (0)	-	0
8 (Acid grassland)	105 (43)	2000	0 (0)	-	-	0.47 (31)	2.75 - 42	8,524 - 130,270
9 (Fen, Marsh, and Swamp)	0 (0)	-	0 (0)	-	-	-	-	0
10 (Heather)	33 (9)	1998	0 (0)	-	-	0.39 (5)	2.76 - 42.2	1,381 - 21,101
11 (Heather grassland)	22 (12)	1998	0 (0)	-	-	0.27 (1)	2.76 - 42.2	276.2 - 4,220
12 (Bog)	14 (7)	2000	0 (0)	-	-	0.19 (2)	2.76 - 42.2	552.3 - 8,440
13 (Montane habitat)	3 (1)	1965	0 (0)	-	-	0.16 (0)	-	0
14 (Inland rock)	0 (0)	-	0 (0)	-	-	0.07 (0)	-	0
15 (Saltwater)	0 (0)	-	0 (0)	-	-	0.39 (0)	-	0
16 (Freshwater)	21 (2)	2004	0 (0)	-	-	0.55 (2)	2.74 - 41.8	547.5 - 8,367
17 (Supra-littoral rock)	0 (0)	-	0 (0)	-	-	0.02 (0)	-	0
18 (Supra-littoral sediment)	0 (0)	-	0 (0)	-	-	0.18 (0)	-	0
19 (Littoral rock)	0 (0)	-	0 (0)	-	-	0.19 (0)	-	0
20 (Littoral sediment)	71 (6)	2000	0 (0)	-	-	0.47 (0)	-	0
21 (Saltmarsh)	0 (0)	-	0 (0)	-	-	-	-	0
22 (Urban)	7 (3)	1972	0 (0)	-	-	0.6 (0)	-	0
23 (Suburban)	76 (20)	2000	0 (0)	-	-	0.56 (10)	2.76 - 42.2	2,761 - 42,202
Total	8,447 (1,045)	2000	8 (5)	1994	2.76 - 42.2	0.6 (1,142)	2.66 - 40.6	303,314 - 4,635,453

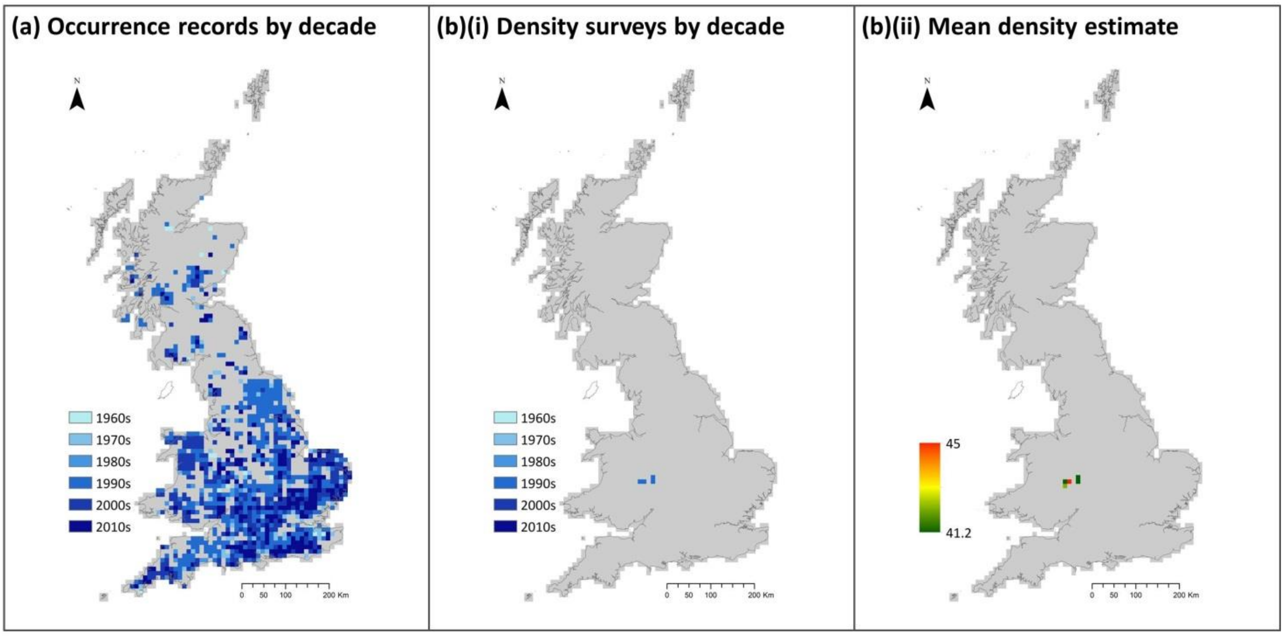

© Crown copyright and database rights 2016 Ordnance Survey 100051110. Data courtesy of the NBN Gateway with thanks to all data contributors. The NBN and its data contributors bear no responsibility for the further analysis or interpretation of this material, data and/or information.

Figure 1: 10km resolution raster maps based on BNG presenting the geographic description of available data. (a) shows the distribution of species occurrence obtained via the NBN Gateway categorised by the decade of last sighting. (b) shows information relating to density surveys identified via a search of published literature where: (i) categorises surveys by the decade of last survey; and (ii) shows the mean density estimate of surveys within grid cells (estimates assumed to be representative of entire cell, considered the upper limit of observed density).

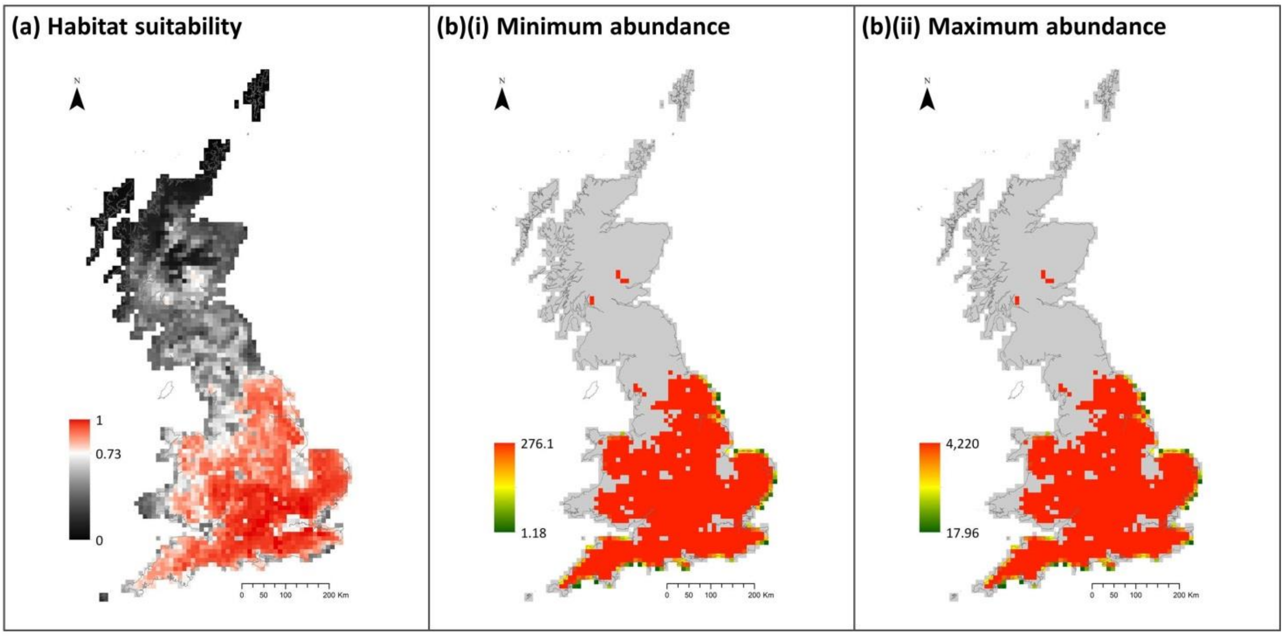

© Crown copyright and database rights 2016 Ordnance Survey 100051110. Data courtesy of the NBN Gateway with thanks to all data contributors. The NBN and its data contributors bear no responsibility for the further analysis or interpretation of this material, data and/or information.

Figure 2: Modelling predictions generated using systematic approach based on available data. (a) shows habitat suitability scores (the likelihood of observing the target species within each grid cell given variation environmental variables) determined by aggregating outputs from the "best" species distribution model (7 models compared) across 100 simulations. Here, the mid value on the scale denotes the threshold score above which occurrence is assumed. (b) shows: (i) the lower bound (Minimum); and (ii) the upper bound (Maximum); of abundance estimates determined by relating observed density (taking into account potential uncertainty) with habitat suitability scores using linear regression.
