# Supplementary material for: A systematic approach to estimate the distribution and total abundance of British mammals
Source: PLoS One. 2017 Jun 28;12(6):e0176339. doi: 10.1371/journal.pone.0176339 (PMC5489149; doi:10.1371/journal.pone.0176339)
Supplement: S3 File — Individual reports for each of the Artiodactyla species presenting analysis of the available data and subsequent model predictions based on a 10km raster grid. Reports also include expert comment assessing the reliability (and plausibility) of results in the context of existing evidence and popular opinion. (ZIP) [file pone.0176339.s003.zip › E Feral sheep.pdf]

Feral sheep (*Ovis aries*)

Order: *Artiodactyla*

Genus: *Ovis*

Origin: Introduced

Status: Locally common

1995 abundance estimate: 1,800 (1)

Reported population trends: None

Data:

The available occurrence records indicate that feral sheep are sparsely distributed throughout GB with most observations clustered in north east Scotland and the north west of England (Figure 1a). Typically, sightings were reported in landscapes dominated by improved grassland and montane land cover. The majority of these grid cells containing at least one observation since 1995.

From the literature review we identified several studies (Bullock 1985; Clutton-Brock et al. 1991; Harris et al. 1995) reporting density estimates for southern Scotland in 1979 (Figure 1b); consequently estimates may be outdated. Estimates ranged between 24.19 and 125.6 per km^2 with the highest densities recorded in habitats dominated by acid grassland (18.96 - 105.2 per km^2 accounting for uncertainty relating to unsurveyed areas within grid cells). Whilst studies provided estimates in key land covers (where much occurrence was recorded) there remained a majority of habitats where occurrence was observed for which estimates were unavailable (land class marked grey in Table 1).

Model predictions:

The habitat suitability map (Figure 2a) appears to reflect the underlying data reasonably well with the set of "best" models predicting presence (and absence) to a mean AUC of 0.72. However, the resulting distribution is substantially larger than the area described by the observations (approximately 20 times). Whilst this may be a true representation of suitable habitat it does not take into account the restrictions on movement imposed by the active management of this species and consequently it may lead to an overestimation of abundance. Overall, across 100 repetitions MaxEnt proved to be the most commonly selected modelling approach displaying the highest AUC 26% of the time followed closely by Domain (20%). By land cover the mean habitat suitability scores suggest observation is most likely in landscapes dominated by montane habitat (Table 1) but, consistent with recorded sightings, the majority of occurrence is predicted in grid cells dominated by improved grassland.

Perhaps due to the limited volume and variability of density data neither minimum nor maximum density estimates showed a correlation with habitat suitability and consequently best fit models were applied based on a constant. However, the best fit model for maximum density did require consideration for spherical spatial autocorrelation.

As anticipated the predicted abundance range does not contain the estimate from Harris et al. (1995); instead suggesting a substantial increase in the total population. As suggested above this is most likely due to the inflated distribution despite the use of a limited number of high density estimates (the same estimates were available and subsequently used to produce the 1995 abundance estimate). Since the model does not take into account any restrictions to movement, which could be argued are applicable for this species, the result may instead be interpreted as a potential carrying capacity should they be introduced across GB.

Reliability (Expert comment):

Whilst there may be a definitive characterisation separating feral animals from managed livestock visual determination is subjective and thereby unreliable. Consequently, it is difficult to comment on the distribution of observations with any degree of confidence. As is the case for feral goats, the density estimates obtained from the literature appear to be based on problem populations where numbers are too high and management is required. As a result they do not reflect the range of densities which could be expected across all areas where the species is present.

The inflated coverage predicted by the habitat suitability is plausible on the basis of habitat it does not account for human interference which limits expansion. Given the high density used in model predictions and unrealistically

inflated coverage it is inevitable that the predicted abundance range reflects a significant overestimation of the population which is completely implausible.

References:

Bullock, D. J. (1985). Annual diets of hill sheep and feral goats in southern Scotland. *Journal of Applied Ecology* 22(2): 423-433.

Clutton-Brock, T. H., O. F. Price, S. D. Albon and P. A. Jewell (1991). Persistent instability and population regulation in Soay sheep. *Journal of Animal Ecology* 60(2): 593-608.

Harris, S. J., P. Morris, S. Wray and D. Yalden (1995). A review of British mammals: population estimates and conservation status of British mammals other than cetaceans, Joint Nature Conservation Committee, Peterborough, UK.

Table 1: Summary of observed data and model predictions by land cover class (LCM2007 target classification). Values shown in brackets denote the spatial coverage based on a 10km resolution raster map (number of grid cells). Years represent the median of records within each land class. Ranges for density and abundance are derived using the respective minimum and maximum raster maps (lower bound is mean of values across minimum raster map with upper across the maximum) which capture the spatial uncertainty generate by projecting irregular polygons describing survey sites onto a raster grid.

LCM2007 class	Observed					Predicted		
	Occurrence		Density					
	Records	Year	Estimates	Year	Range	Habitat suitability	Density	Abundance
1 (Broadleaved woodland)	1 (1)	2006	0 (0)	-	-	0.45 (2)	9.82 - 74.54	1,964 - 14,909
2 (Coniferous woodland)	3 (2)	1998	6 (6)	1979	5.25 - 54.6	0.6 (81)	9.67 - 73.41	78,348 - 594,626
3 (Arable and Horticultural)	1 (1)	1995	0 (0)	-	-	0.35 (8)	7.43 - 56.41	5,946 - 45,129
4 (Improved grassland)	15 (10)	2006	0 (0)	-	-	0.49 (193)	8.92 - 67.73	172,233 - 1,307,173
5 (Rough grassland)	2 (2)	2002	0 (0)	-	-	0.42 (12)	5.2 - 39.46	6,239 - 47,349
6 (Neutral grassland)	0 (0)	-	0 (0)	-	-	0.25 (0)	-	0
7 (Calcareous grassland)	0 (0)	-	0 (0)	-	-	0.5 (0)	-	0
8 (Acid grassland)	3 (1)	1999	3 (3)	1979	18.96 - 105	0.44 (68)	9.82 - 74.54	66,788 - 506,892
9 (Fen, Marsh, and Swamp)	0 (0)	-	0 (0)	-	-	-	-	0
10 (Heather)	7 (2)	1981	0 (0)	-	-	0.61 (32)	9.82 - 74.54	31,430 - 238,537
11 (Heather grassland)	1 (1)	1999	0 (0)	-	-	0.42 (22)	9.82 - 74.54	21,608 - 163,994
12 (Bog)	3 (2)	1988	0 (0)	-	-	0.44 (23)	9.82 - 74.54	22,590 - 171,449
13 (Montane habitat)	29 (5)	1999	0 (0)	-	-	0.63 (29)	9.82 - 74.54	28,483 - 216,174
14 (Inland rock)	0 (0)	-	0 (0)	-	-	0.23 (0)	-	0
15 (Saltwater)	0 (0)	-	0 (0)	-	-	0.51 (4)	2.89 - 21.97	1,158 - 8,787
16 (Freshwater)	0 (0)	-	0 (0)	-	-	0.41 (0)	-	0
17 (Supra-littoral rock)	0 (0)	-	0 (0)	-	-	0.35 (0)	-	0
18 (Supra-littoral sediment)	0 (0)	-	0 (0)	-	-	0.43 (0)	-	0
19 (Littoral rock)	0 (0)	-	0 (0)	-	-	0.35 (0)	-	0
20 (Littoral sediment)	8 (2)	2000	0 (0)	-	-	0.57 (18)	6.13 - 46.51	11,032 - 83,726
21 (Saltmarsh)	0 (0)	-	0 (0)	-	-	-	-	0
22 (Urban)	0 (0)	-	0 (0)	-	-	0.18 (0)	-	0
23 (Suburban)	0 (0)	-	0 (0)	-	-	0.24 (0)	-	0
Total	73 (29)	1999	9 (9)	1979	9.82 - 71.46	0.43 (492)	9.1 - 69.08	447,819 - 3,398,746

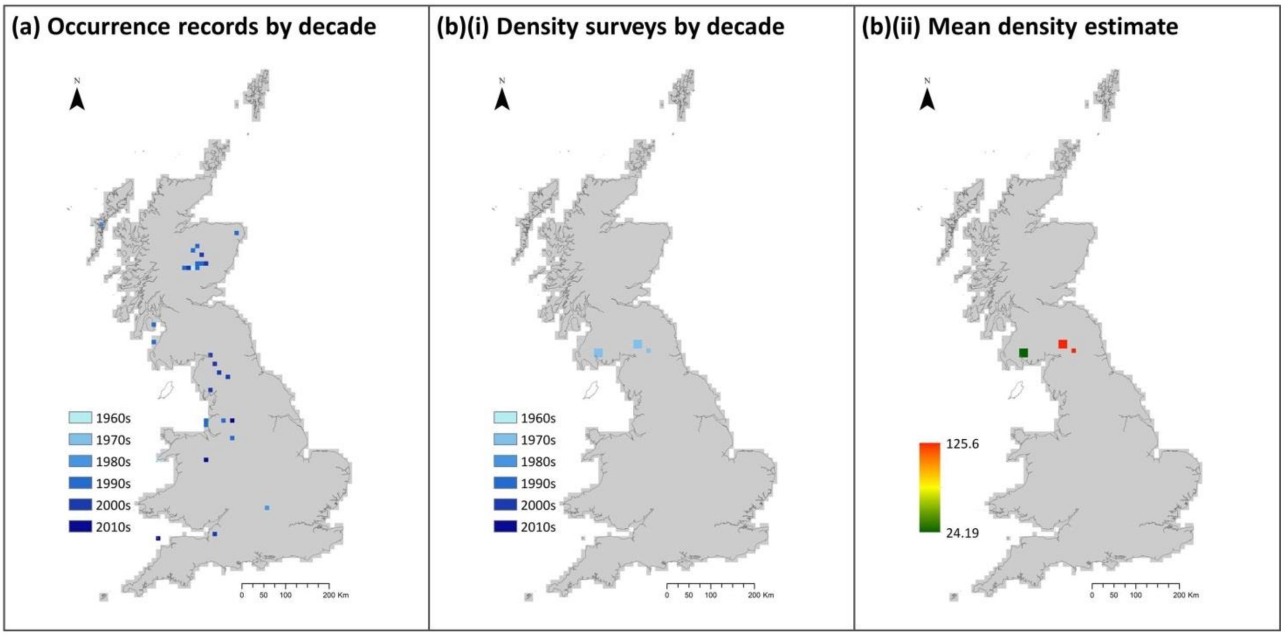

© Crown copyright and database rights 2016 Ordnance Survey 100051110. Data courtesy of the NBN Gateway with thanks to all data contributors. The NBN and its data contributors bear no responsibility for the further analysis or interpretation of this material, data and/or information.

Figure 1: 10km resolution raster maps based on BNG presenting the geographic description of available data. (a) shows the distribution of species occurrence obtained via the NBN Gateway categorised by the decade of last sighting. (b) shows information relating to density surveys identified via a search of published literature where: (i) categorises surveys by the decade of last survey; and (ii) shows the mean density estimate of surveys within grid cells (estimates assumed to be representative of entire cell, considered the upper limit of observed density).

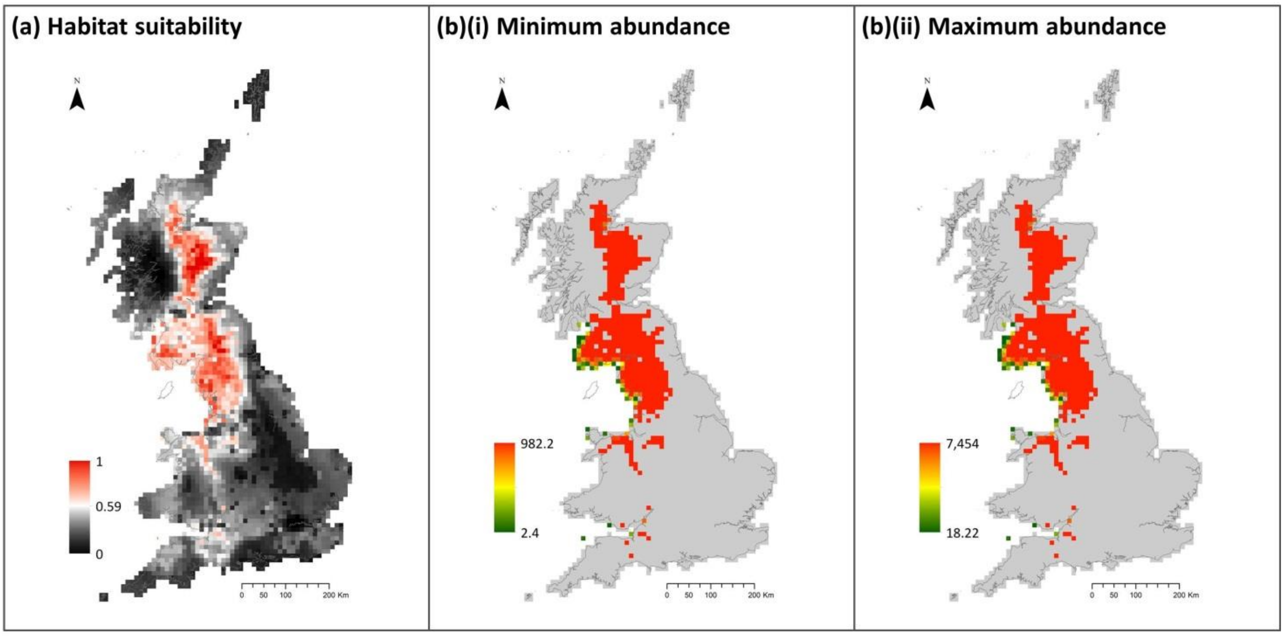

© Crown copyright and database rights 2016 Ordnance Survey 100051110. Data courtesy of the NBN Gateway with thanks to all data contributors. The NBN and its data contributors bear no responsibility for the further analysis or interpretation of this material, data and/or information.

Figure 2: Modelling predictions generated using systematic approach based on available data. (a) shows habitat suitability scores (the likelihood of observing the target species within each grid cell given variation environmental variables) determined by aggregating outputs from the "best" species distribution model (7 models compared) across 100 simulations. Here, the mid value on the scale denotes the threshold score above which occurrence is assumed. (b) shows: (i) the lower bound (Minimum); and (ii) the upper bound (Maximum); of abundance estimates determined by relating observed density (taking into account potential uncertainty) with habitat suitability scores using linear regression.
