# Supplementary material for: A systematic approach to estimate the distribution and total abundance of British mammals
Source: PLoS One. 2017 Jun 28;12(6):e0176339. doi: 10.1371/journal.pone.0176339 (PMC5489149; doi:10.1371/journal.pone.0176339)
Supplement: S3 File — Individual reports for each of the Artiodactyla species presenting analysis of the available data and subsequent model predictions based on a 10km raster grid. Reports also include expert comment assessing the reliability (and plausibility) of results in the context of existing evidence and popular opinion. (ZIP) [file pone.0176339.s003.zip › F Park cattle.pdf]

Park cattle (*Bos taurus*)

Order: *Artiodactyla* **Genus:** *Bos*

Origin: Native **Status:** Locally common

1995 abundance estimate: 45 (1) **Reported population trends:** None

Data:

The available occurrence records indicate that park cattle are sparsely distributed with localised sightings on farmland (arable and improved grassland) in northern and western England between 1998 and 2007 (Figure 1a).

From the literature review we identified a single survey (Hall 1982) conducted at Chillingham in 1981 which estimated density within the park to be approximately 37 per km^2 (Figure 1b).

Model predictions:

Unfortunately, there was insufficient data available at a 10km scale to perform any model analysis.

Reliability (Expert comment):

The British population of park cattle are fully managed in enclosed parks and therefore cannot be considered a wild species. The general modelling process does not take into account any restrictions on movement as a result of active management and therefore, even if the was sufficient data for analysis, it may be reasonable to anticipate that predictions would be implausible (a strong association with common landscapes dominated by arable and improved grassland without considerations for containment would likely lead to an overestimated distribution).

References:

Hall, S. J. G. (1982). The Chillingham herd of wild white cattle. *Applied Animal Ethology* 9(1): 96-97.

Table 1: Summary of observed data and model predictions by land cover class (LCM2007 target classification). Values shown in brackets denote the spatial coverage based on a 10km resolution raster map (number of grid cells). Years represent the median of records within each land class. Ranges for density and abundance are derived using the respective minimum and maximum raster maps (lower bound is mean of values across minimum raster map with upper across the maximum) which capture the spatial uncertainty generate by projecting irregular polygons describing survey sites onto a raster grid.

LCM2007 class	Observed					Predicted		
	Occurrence		Density					
	Records	Year	Estimates	Year	Range	Habitat suitability	Density	Abundance
1 (Broadleaved woodland)	0 (0)	-	0 (0)	-	-	-	-	-
2 (Coniferous woodland)	0 (0)	-	0 (0)	-	-	-	-	-
3 (Arable and Horticultural)	9 (3)	1998	0 (0)	-	-	-	-	-
4 (Improved grassland)	7 (5)	2007	1 (1)	1981	36.57	-	-	-
5 (Rough grassland)	0 (0)	-	0 (0)	-	-	-	-	-
6 (Neutral grassland)	0 (0)	-	0 (0)	-	-	-	-	-
7 (Calcareous grassland)	0 (0)	-	0 (0)	-	-	-	-	-
8 (Acid grassland)	0 (0)	-	0 (0)	-	-	-	-	-
9 (Fen, Marsh, and Swamp)	0 (0)	-	0 (0)	-	-	-	-	-
10 (Heather)	0 (0)	-	0 (0)	-	-	-	-	-
11 (Heather grassland)	0 (0)	-	0 (0)	-	-	-	-	-
12 (Bog)	0 (0)	-	0 (0)	-	-	-	-	-
13 (Montane habitat)	0 (0)	-	0 (0)	-	-	-	-	-
14 (Inland rock)	0 (0)	-	0 (0)	-	-	-	-	-
15 (Saltwater)	0 (0)	-	0 (0)	-	-	-	-	-
16 (Freshwater)	0 (0)	-	0 (0)	-	-	-	-	-
17 (Supra-littoral rock)	0 (0)	-	0 (0)	-	-	-	-	-
18 (Supra-littoral sediment)	0 (0)	-	0 (0)	-	-	-	-	-
19 (Littoral rock)	0 (0)	-	0 (0)	-	-	-	-	-
20 (Littoral sediment)	0 (0)	-	0 (0)	-	-	-	-	-
21 (Saltmarsh)	0 (0)	-	0 (0)	-	-	-	-	-
22 (Urban)	0 (0)	-	0 (0)	-	-	-	-	-
23 (Suburban)	0 (0)	-	0 (0)	-	-	-	-	-
Total	16 (8)	2006	1 (1)	1981	36.57	-	-	-

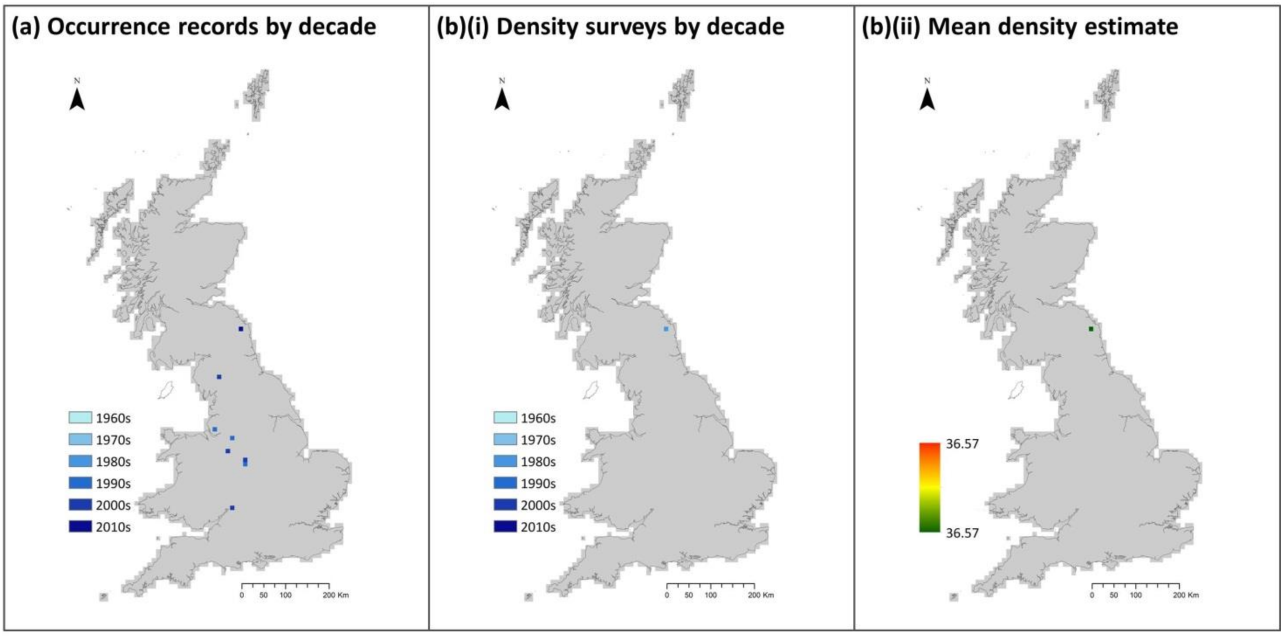

© Crown copyright and database rights 2016 Ordnance Survey 100051110. Data courtesy of the NBN Gateway with thanks to all data contributors. The NBN and its data contributors bear no responsibility for the further analysis or interpretation of this material, data and/or information.

Figure 1: 10km resolution raster maps based on BNG presenting the geographic description of available data. (a) shows the distribution of species occurrence obtained via the NBN Gateway categorised by the decade of last sighting. (b) shows information relating to density surveys identified via a search of published literature where: (i) categorises surveys by the decade of last survey; and (ii) shows the mean density estimate of surveys within grid cells (estimates assumed to be representative of entire cell, considered the upper limit of observed density).

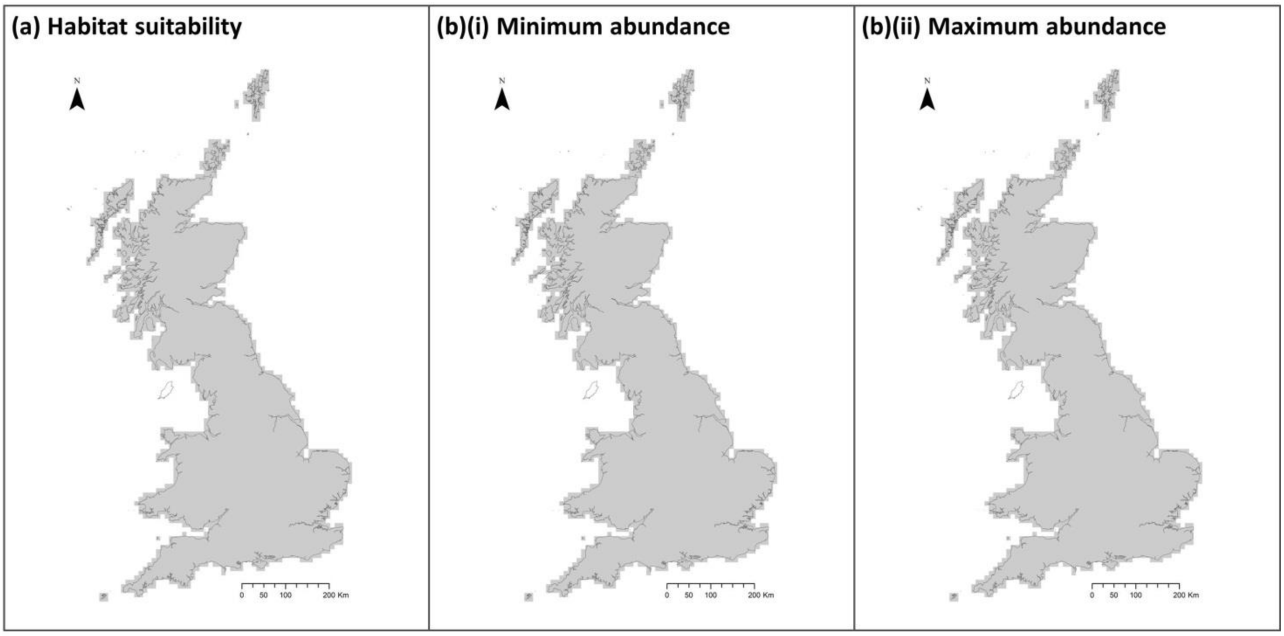

© Crown copyright and database rights 2016 Ordnance Survey 100051110. Data courtesy of the NBN Gateway with thanks to all data contributors. The NBN and its data contributors bear no responsibility for the further analysis or interpretation of this material, data and/or information.

Figure 2: Modelling predictions generated using systematic approach based on available data. (a) shows habitat suitability scores (the likelihood of observing the target species within each grid cell given variation environmental variables) determined by aggregating outputs from the "best" species distribution model (7 models compared) across 100 simulations. Here, the mid value on the scale denotes the threshold score above which occurrence is assumed. (b) shows: (i) the lower bound (Minimum); and (ii) the upper bound (Maximum); of abundance estimates determined by relating observed density (taking into account potential uncertainty) with habitat suitability scores using linear regression.
