# Supplementary material for: A systematic approach to estimate the distribution and total abundance of British mammals
Source: PLoS One. 2017 Jun 28;12(6):e0176339. doi: 10.1371/journal.pone.0176339 (PMC5489149; doi:10.1371/journal.pone.0176339)
Supplement: S3 File — Individual reports for each of the Artiodactyla species presenting analysis of the available data and subsequent model predictions based on a 10km raster grid. Reports also include expert comment assessing the reliability (and plausibility) of results in the context of existing evidence and popular opinion. (ZIP) [file pone.0176339.s003.zip › G Red deer.pdf]

Red deer (*Cervus elaphus*)

Order: *Artiodactyla* | **Genus:** *Cervus*

Origin: Native | **Status:** Common

1995 abundance estimate: 360,000 | **Reported population trends:** JNCC 2005 (↑), NGC 2009, BBS 2014 (↔)

Data:

The available occurrence records indicate that red deer are distributed throughout GB with the largest patches of coverage across Scotland, East Anglia, Cumbria and Exmoor (Figure 1a). These sightings were reported in various habitats (predominantly arable and improved grassland) the majority of which since 1995.

From the literature review we identified two publications reporting density estimates (Edwards & Kenyon 2013; Lowe 1969). The first provided the most recent density estimates from count data in each of the Deer Management Group regions across Scotland between 1992 and 2010; the second provided a historic estimate for the isolated island population on Rhum (Figure 1b). Estimates ranged between 0.69 and 27.73 per km^2 with the highest densities recorded in littoral rock dominated habitat (10.1 - 13.1per km^2 accounting for uncertainty relating to unsurveyed areas within grid cells). Despite the relatively high proportion of area surveyed estimates for several land cover classes were not available (land class marked grey in Table 1).

Model predictions:

The habitat suitability map (Figure 2a) appears to reflect the underlying data well with the set of "best" models predicting presence (and absence) to a mean AUC of 0.72. Overall, across 100 repetitions Random Forest proved to be the most commonly selected modelling approach displaying the highest AUC 46% of the time followed by MaxEnt (21%). By land cover the mean habitat suitability scores suggest observation is most likely in landscapes dominated by inland rock and montane habitats (Table 1) but, consistent with recorded sightings, the majority of occurrence is predicted in grid cells dominated by arable and improved grassland.

Both minimum and maximum density estimates were best fitted to the square of habitat suitability accounting for spherical spatial autocorrelation. Interestingly, the relationship for maximum density suggest that some of the areas of highest suitability do not contain the most abundance, this is instead associated across the midrange of suitability.

Whilst the predicted abundance range does not contain the estimate from Harris et al. (1995) our predictions suggests a significant increase in population (at least 5%) consistent with the reported trend by JNCC in 2005 which may explain the overestimation. However, the most recent trends do not agree with this assessment predicting no change in population since 1995. It is perhaps more plausible therefore that the overestimation is due to the lack of density estimates for the populations in England.

Reliability (Expert comment):

The distribution of observed occurrence appears reasonable for Scotland. However, the observations in England are slightly more widespread than expected; populations are more limited to the core areas of Norfolk, Peninsula and Cumbria. The range of density estimates also seems representative. The resulting abundance range predicted by the model seems plausible, particularly at the lower end where the corresponding distribution captures the lower abundance of many of the smaller populations in England.

References:

Edwards, T. and W. Kenyon (2013). SPICe briefing : wild deer in Scotland. Scottish Natural Heritage.

Harris, S. J., P. Morris, S. Wray and D. Yalden (1995). A review of British mammals: population estimates and conservation status of British mammals other than cetaceans, Joint Nature Conservation Committee, Peterborough, UK.

Lowe, V. P. W. (1969). Population Dynamics of the Red Deer (*Cervus elaphus*) on Rhum. *Journal of Animal Ecology* 38(2): 425-457.

Table 1: Summary of observed data and model predictions by land cover class (LCM2007 target classification). Values shown in brackets denote the spatial coverage based on a 10km resolution raster map (number of grid cells). Years represent the median of records within each land class. Ranges for density and abundance are derived using the respective minimum and maximum raster maps (lower bound is mean of values across minimum raster map with upper across the maximum) which capture the spatial uncertainty generate by projecting irregular polygons describing survey sites onto a raster grid.

LCM2007 class	Observed					Predicted		
	Occurrence		Density					
	Records	Year	Estimates	Year	Range	Habitat suitability	Density	Abundance
1 (Broadleaved woodland)	163 (10)	2000	0 (0)	-	-	0.89 (11)	4.4 - 8.1	4,810 - 8,956
2 (Coniferous woodland)	1,086 (126)	2000	95 (57)	2010	5.4 - 8.8	0.87 (128)	4.4 - 7.4	56,520 - 94,085
3 (Arable and Horticultural)	3,238 (362)	2000	8 (8)	2010	1.6 - 7.9	0.69 (266)	2.5 - 7.2	65,078 - 191,228
4 (Improved grassland)	2,321 (278)	2000	23 (21)	2009	2.3 - 8.9	0.67 (230)	2 - 6.8	46,268 - 156,678
5 (Rough grassland)	172 (28)	1996	27 (19)	2003	5.7 - 7	0.56 (29)	1.8 - 3.6	5,279 - 10,301
6 (Neutral grassland)	0 (0)	-	0 (0)	-	-	0.04 (0)	-	-
7 (Calcareous grassland)	2 (1)	2005	0 (0)	-	-	0.68 (0)	-	-
8 (Acid grassland)	2,029 (142)	2008	172 (97)	2009	8 - 8.9	0.81 (136)	4.7 - 7.8	63,828 - 105,542
9 (Fen, Marsh, and Swamp)	0 (0)	-	0 (0)	-	-	-	-	-
10 (Heather)	695 (49)	2009	60 (40)	2009	6.9 - 9.3	0.92 (56)	4.4 - 6.7	24,879 - 37,246
11 (Heather grassland)	1,168 (116)	2005	142 (106)	2008	6.1 - 8.1	0.82 (123)	3.4 - 5.4	41,583 - 66,062
12 (Bog)	608 (95)	2000	104 (87)	2007	4.7 - 5.4	0.77 (105)	3.8 - 6.4	40,018 - 66,688
13 (Montane habitat)	1,313 (51)	2004	74 (51)	2010	10.8 - 11	0.97 (53)	5.5 - 7.7	29,144 - 40,623
14 (Inland rock)	9 (1)	2003	1 (1)	2003	0.7	0.98 (1)	5.6 - 7.7	564.2 - 768.5
15 (Saltwater)	8 (2)	1992	0 (0)	-	-	0.5 (1)	0 - 6.3	-
16 (Freshwater)	21 (3)	2006	1 (1)	2000	0.9 - 1.2	0.94 (3)	4.8 - 6.5	1,311 - 1,945
17 (Supra-littoral rock)	0 (0)	-	1 (1)	2000	1.2 - 3.9	0.79 (1)	0 - 0.01	0.09 - 0.77
18 (Supra-littoral sediment)	0 (0)	-	2 (2)	2000	1.2 - 2.9	0.56 (2)	0.07 - 0.3	13.62 - 55.28
19 (Littoral rock)	0 (0)	-	3 (2)	1984	10.1 - 13.1	0.52 (1)	0.02 - 0.04	1.62 - 3.73
20 (Littoral sediment)	32 (2)	2010	0 (0)	-	-	0.35 (0)	-	-
21 (Saltmarsh)	0 (0)	-	0 (0)	-	-	-	-	-
22 (Urban)	1 (1)	2000	0 (0)	-	-	0.4 (0)	-	-
23 (Suburban)	31 (10)	2006	0 (0)	-	-	0.42 (0)	-	-
Total	12,897 (1,277)	2001	713 (493)	2009	6.4 - 8.2	0.71 (1,146)	3.3 - 6.8	379,297 - 780,812

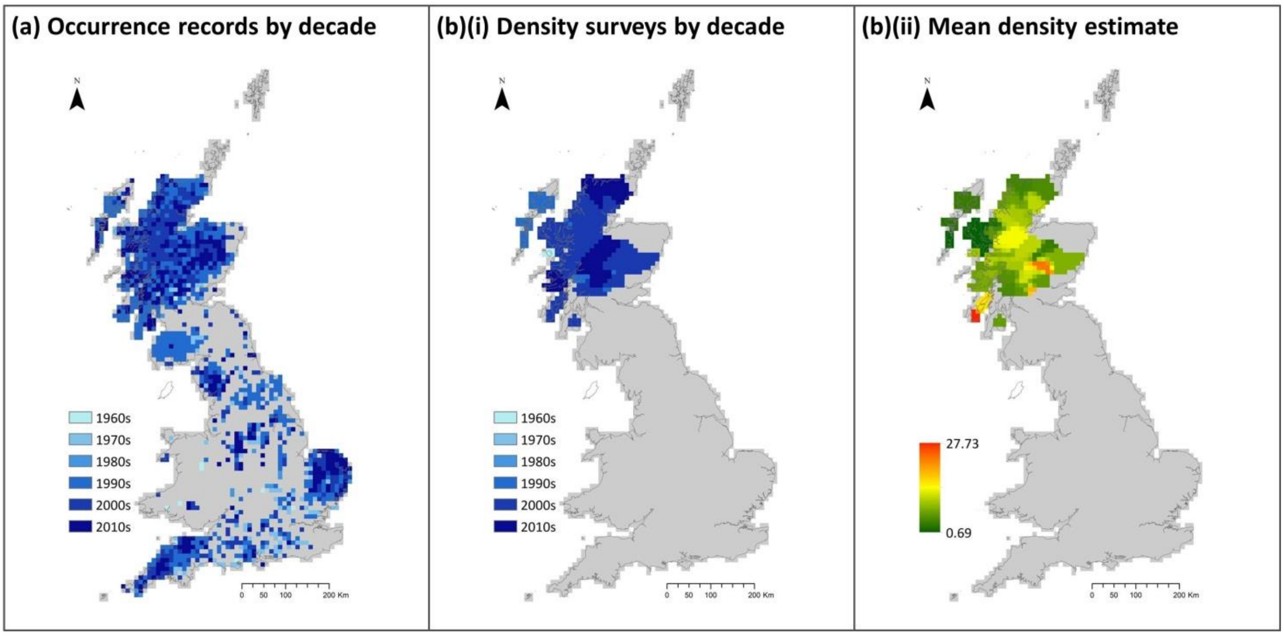

© Crown copyright and database rights 2016 Ordnance Survey 100051110. Data courtesy of the NBN Gateway with thanks to all data contributors. The NBN and its data contributors bear no responsibility for the further analysis or interpretation of this material, data and/or information.

Figure 1: 10km resolution raster maps based on BNG presenting the geographic description of available data. (a) shows the distribution of species occurrence obtained via the NBN Gateway categorised by the decade of last sighting. (b) shows information relating to density surveys identified via a search of published literature where: (i) categorises surveys by the decade of last survey; and (ii) shows the mean density estimate of surveys within grid cells (estimates assumed to be representative of entire cell, considered the upper limit of observed density).

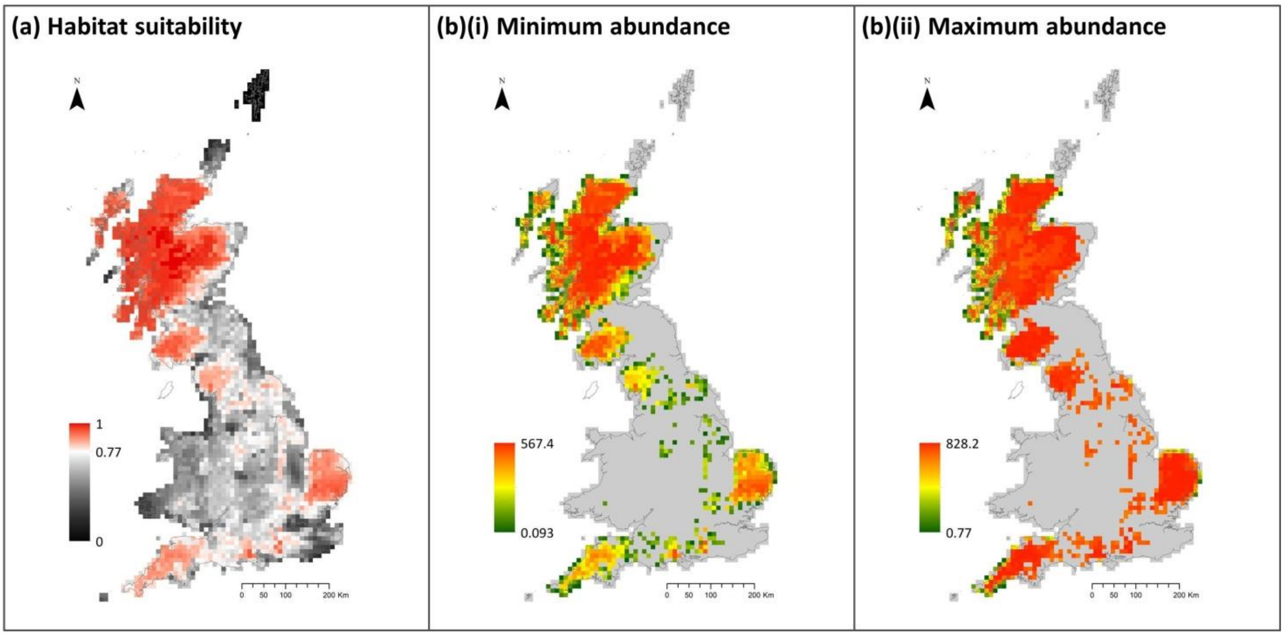

© Crown copyright and database rights 2016 Ordnance Survey 100051110. Data courtesy of the NBN Gateway with thanks to all data contributors. The NBN and its data contributors bear no responsibility for the further analysis or interpretation of this material, data and/or information.

Figure 2: Modelling predictions generated using systematic approach based on available data. (a) shows habitat suitability scores (the likelihood of observing the target species within each grid cell given variation environmental variables) determined by aggregating outputs from the "best" species distribution model (7 models compared) across 100 simulations. Here, the mid value on the scale denotes the threshold score above which occurrence is assumed. (b) shows: (i) the lower bound (Minimum); and (ii) the upper bound (Maximum); of abundance estimates determined by relating observed density (taking into account potential uncertainty) with habitat suitability scores using linear regression.
