# Supplementary material for: A systematic approach to estimate the distribution and total abundance of British mammals
Source: PLoS One. 2017 Jun 28;12(6):e0176339. doi: 10.1371/journal.pone.0176339 (PMC5489149; doi:10.1371/journal.pone.0176339)
Supplement: S3 File — Individual reports for each of the Artiodactyla species presenting analysis of the available data and subsequent model predictions based on a 10km raster grid. Reports also include expert comment assessing the reliability (and plausibility) of results in the context of existing evidence and popular opinion. (ZIP) [file pone.0176339.s003.zip › H Roe deer.pdf]

Roe deer (*Capreolus capreolus*)

Order: *Artiodactyla*

Genus: *Capreolus*

Origin: Native

Status: Common

1995 abundance estimate: 500,000 (3)

Reported population trends: JNCC 2005, NGC 2009, BBS 2014 (↑)

Data:

The available occurrence records indicate that the roe deer is widespread throughout Britain with sightings reported in most 10 km squares (approximately 73%) at least once over the past decade (Figure 1a). However, the map highlights several areas, particularly in the west of England and in Wales, where the species has never been recorded and others in the highlands of Scotland where it has not been recorded for some time.

Density estimates, primarily recorded over the past two decades, were obtained from published literature spanning less than 1% of the observed species distribution based on the available occurrence data (Gill et al. 1996, 1997; Hemami et al. 2005, 2007; Mayle 1996). Geographically, these studies were located in the south and east of England with one estimate in Scotland (Figure 1b). Estimates were limited to high density areas ranging between 22.6 and 34 per km^2 with the highest densities recorded in improved grassland (0.03 - 34 per km^2 accounting for uncertainty relating to unsurveyed areas within grid cells). Due to the relatively low proportion of area surveyed estimates for several land cover classes were not available (land class marked grey in Table 1).

Model predictions:

The habitat suitability map (Figure 2a) appears to reflect the underlying data reasonably well with the set of "best" models predicting presence (and absence) to a mean AUC of 0.76. However, the coverage does not extend fully into areas of recorded occurrence in the south west of England. Overall, across 100 repetitions Random Forest proved to be the most commonly selected modelling approach displaying the highest AUC 40% of the time followed by MaxEnt (23%). By land cover the mean habitat suitability scores suggest observation is most likely in landscapes dominated by montane habitat (Table 1) but, consistent with recorded sightings, the majority of occurrence is predicted in arable and improved grassland (the most common dominant land covers at a 10km scale).

Linear regression suggested that there was no correlation between the estimates of minimum density and habitat suitability, consequently, it was applied as a constant in cells where occurrence was predicted. However, maximum density was found to be correlated with the best fit model relating the square of habitat suitability accounting for spherical spatial autocorrelation.

The predicted abundance range does not contain the estimate from Harris et al. (1995). Instead, the range suggests a significant increase which could be explained by recently reported population trends (JNCC, NGC and BBS).

Reliability (Expert comment):

The distribution suggested by observations appears in line with expectation. However, the density range provided by published studies only represents the upper end of values which could be observed across the full distribution. Interestingly, the habitat suitability map identifies Cornwall as a region of marginal occurrence where observation is less likely despite the ubiquity of sightings. Typically, this region is associated with lower abundances. The lower limit of the abundance range is certainly plausible taking into account recent population trends (although it is worth noting that the 1995 estimate is perhaps an overestimation; Ward et al. 2005 estimated the population in 2005 to be between 275,000 and 320,000) but despite these reported increases the upper limit seems unrealistically large.

References:

Gill, R. M. A., A. L. Johnson, A. Francis, K. Hiscocks and A. J. Peace (1996). Changes in roe deer (*Capreolus capreolu*) population density in response to forest habitat succession. *Forest Ecology and Management* 88(1-2): 31-41.

Gill, R. M. A., M. L. Thomas and D. Stocker (1997). The use of portable thermal imaging for estimating deer population density in forest habitats. *Journal of Applied Ecology* 34(5): 1273-1286.

Harris, S. J., P. Morris, S. Wray and D. Yalden (1995). A review of British mammals: population estimates and conservation status of British mammals other than cetaceans, Joint Nature Conservation Committee, Peterborough, UK.

Hemami, M. R., A. R. Watkinson and P. M. Dolman (2005). Population densities and habitat associations of introduced muntjac *Muntiacus reevesi* and native roe deer *Capreolus capreolus* in a lowland pine forest. *Forest Ecology and Management* 215(1–3): 224-238.

Hemami, M. R., A. R. Watkinson, R. M. A. Gill and P. M. Dolman (2007). Estimating abundance of introduced Chinese muntjac *Muntiacus reevesi* and native roe deer *Capreolus capreolus* using portable thermal imaging equipment. *Mammal Review* 37(3): 246-254.

Mayle, B. A. (1996). Progress in predictive management of deer populations in British woodlands. *Forest Ecology and Management* 88(1–2): 187-198.

Table 1: Summary of observed data and model predictions by land cover class (LCM2007 target classification). Values shown in brackets denote the spatial coverage based on a 10km resolution raster map (number of grid cells). Years represent the median of records within each land class. Ranges for density and abundance are derived using the respective minimum and maximum raster maps (lower bound is mean of values across minimum raster map with upper across the maximum) which capture the spatial uncertainty generate by projecting irregular polygons describing survey sites onto a raster grid.

LCM2007 class	Observed					Predicted		
	Occurrence		Density					
	Records	Year	Estimates	Year	Range	Habitat suitability	Density	Abundance
1 (Broadleaved woodland)	570 (11)	2014	2 (2)	1995	0.81 - 22.6	0.93 (11)	3.45 - 27.4	3,797 - 30,175
2 (Coniferous woodland)	2,537 (143)	2008	2 (2)	1998	9.26 - 26.6	0.93 (138)	3.23 - 25.8	44,545 - 356,398
3 (Arable and Horticultural)	20,565 (800)	2012	11 (10)	2002	2.97 - 27.5	0.9 (735)	3.25 - 25.8	238,567 - 1,899,399
4 (Improved grassland)	11605 (527)	2011	1 (1)	1980	0.03 - 34	0.83 (410)	3.12 - 24.8	127,800 - 1,018,539
5 (Rough grassland)	376 (15)	2009	2 (1)	2002	5.34 - 28	0.43 (10)	3.01 - 23.9	3,008 - 23,937
6 (Neutral grassland)	0 (0)	-	0 (0)	-	-	0.06 (0)	-	-
7 (Calcareous grassland)	177 (2)	2014	0 (0)	-	-	0.96 (2)	3.45 - 27.6	690.3 - 5,523
8 (Acid grassland)	1,841 (173)	2000	0 (0)	-	-	0.9 (150)	3.42 - 27.3	51,256 - 409,866
9 (Fen, Marsh, and Swamp)	0 (0)	-	0 (0)	-	-	-	-	-
10 (Heather)	802 (50)	2009	0 (0)	-	-	0.91 (50)	3.36 - 27	16,781 - 134998
11 (Heather grassland)	946 (101)	2001	0 (0)	-	-	0.74 (84)	2.96 - 23.7	24,859 - 198,691
12 (Bog)	531 (91)	2000	0 (0)	-	-	0.64 (76)	3.26 - 26.1	24,761 - 198,285
13 (Montane habitat)	429 (53)	1998	0 (0)	-	-	0.98 (53)	3.45 - 28	18,294 - 148,215
14 (Inland rock)	1 (1)	1999	0 (0)	-	-	0.78 (0)	-	-
15 (Saltwater)	84 (6)	2013	0 (0)	-	-	0.77 (5)	1.82 - 14.4	908.1 - 7,218
16 (Freshwater)	28 (2)	2002	0 (0)	-	-	0.71 (2)	3.42 - 27.2	684.4 - 5,446
17 (Supra-littoral rock)	0 (0)	-	0 (0)	-	-	0.15 (0)	-	-
18 (Supra-littoral sediment)	25 (2)	2010	0 (0)	-	-	0.51 (2)	0.48 - 3.81	96.14 - 762.6
19 (Littoral rock)	1 (1)	2000	0 (0)	-	-	0.46 (0)	-	-
20 (Littoral sediment)	371 (18)	2012	0 (0)	-	-	0.74 (9)	2.8 - 22.3	2,517 - 20,028
21 (Saltmarsh)	0 (0)	-	0 (0)	-	-	-	-	-
22 (Urban)	80 (3)	2012	0 (0)	-	-	0.67 (0)	-	-
23 (Suburban)	1,439 (48)	2012	0 (0)	-	-	0.79 (17)	3.16 - 25.2	5,368 - 42,802
Total	42,408 (2,047)	2011	18 (16)	2002	3.45 - 27.2	0.84 (1,754)	3.22 - 25.7	563,932 - 4,500,284

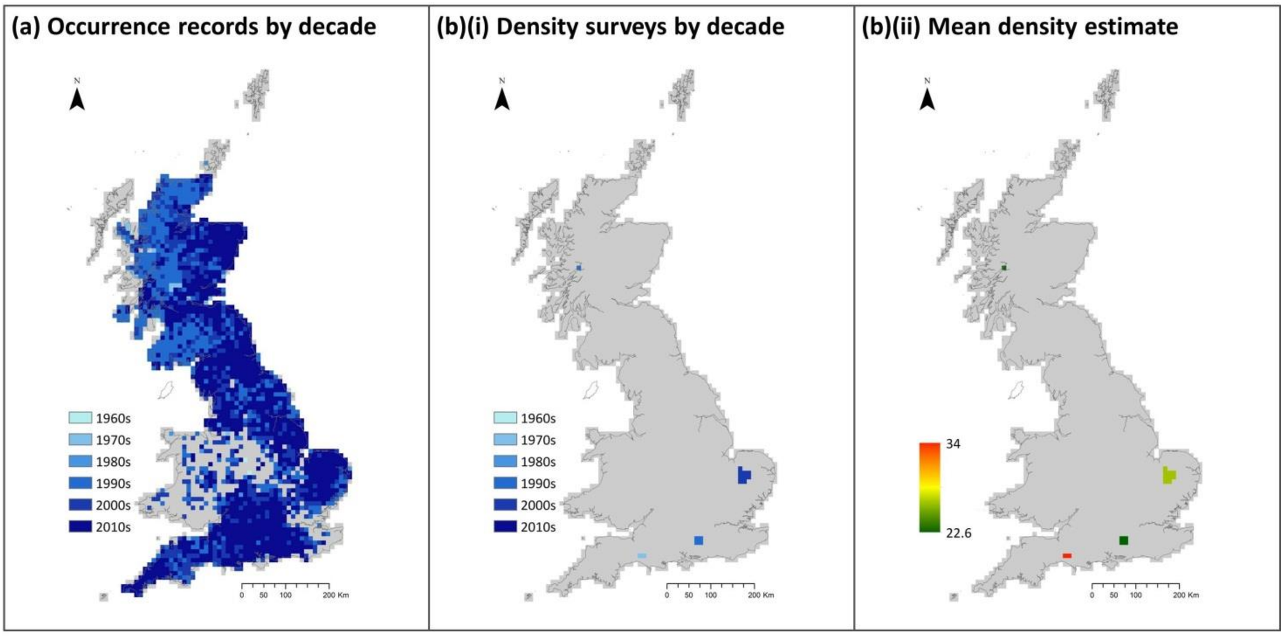

© Crown copyright and database rights 2016 Ordnance Survey 100051110. Data courtesy of the NBN Gateway with thanks to all data contributors. The NBN and its data contributors bear no responsibility for the further analysis or interpretation of this material, data and/or information.

Figure 1: 10km resolution raster maps based on BNG presenting the geographic description of available data. (a) shows the distribution of species occurrence obtained via the NBN Gateway categorised by the decade of last sighting. (b) shows information relating to density surveys identified via a search of published literature where: (i) categorises surveys by the decade of last survey; and (ii) shows the mean density estimate of surveys within grid cells (estimates assumed to be representative of entire cell, considered the upper limit of observed density).

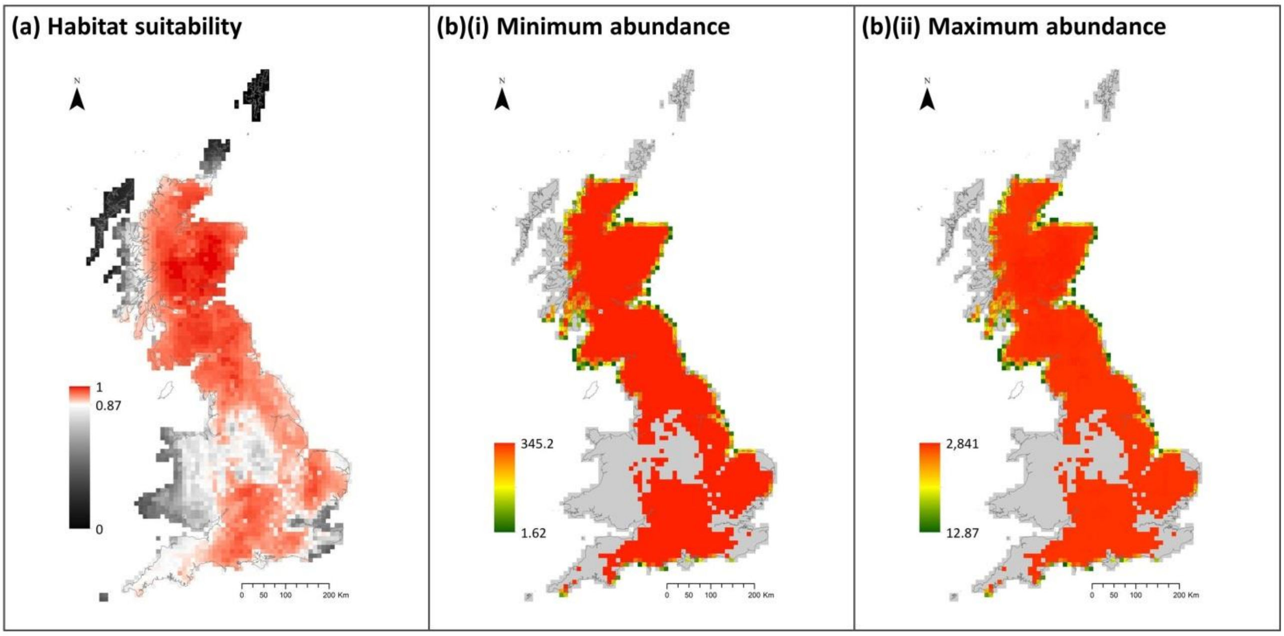

© Crown copyright and database rights 2016 Ordnance Survey 100051110. Data courtesy of the NBN Gateway with thanks to all data contributors. The NBN and its data contributors bear no responsibility for the further analysis or interpretation of this material, data and/or information.

Figure 2: Modelling predictions generated using systematic approach based on available data. (a) shows habitat suitability scores (the likelihood of observing the target species within each grid cell given variation environmental variables) determined by aggregating outputs from the "best" species distribution model (7 models compared) across 100 simulations. Here, the mid value on the scale denotes the threshold score above which occurrence is assumed. (b) shows: (i) the lower bound (Minimum); and (ii) the upper bound (Maximum); of abundance estimates determined by relating observed density (taking into account potential uncertainty) with habitat suitability scores using linear regression.
