# Supplementary material for: A systematic approach to estimate the distribution and total abundance of British mammals
Source: PLoS One. 2017 Jun 28;12(6):e0176339. doi: 10.1371/journal.pone.0176339 (PMC5489149; doi:10.1371/journal.pone.0176339)
Supplement: S3 File — Individual reports for each of the Artiodactyla species presenting analysis of the available data and subsequent model predictions based on a 10km raster grid. Reports also include expert comment assessing the reliability (and plausibility) of results in the context of existing evidence and popular opinion. (ZIP) [file pone.0176339.s003.zip › I Sika deer.pdf]

Sika deer (*Cervus nippon*)

Order: *Artiodactyla*

Genus: *Cervus*

Origin: Introduced

Status: Locally common

1995 abundance estimate: 11,500 (2)

Reported population trends: JNCC 2005, NGC 2009 (↑)

Data:

The available occurrence records indicate that sika deer are most widespread in the Highlands of Scotland with more localised coverage reported in the Borders, North Yorkshire, Lancashire and across southern parts of England, notably Dorset and the New Forest (Figure 1a). The species has not been recorded in Wales (despite some reports of localised presence) and in much of central England. In general, sightings were reported in various habitats (predominantly arable and improved grassland) the majority since 1995.

From the literature review we identified two studies (Marques et al. 2001; Putman & Clifton-Bligh 1997); one conducted in Scotland providing estimates for 1998 and the other in Dorset providing an estimate for 1993 (Figure 1b). Estimates ranged between 0 and 11.96 per km^2 with the highest densities recorded in arable dominated habitat (0.15 - 7.16 per km^2 accounting for uncertainty relating to unsurveyed areas within grid cells). Despite the relatively high proportion of area surveyed (approximately 10% of observed occurrence based on coverage) estimates for several land cover classes were not available (marked grey in Table 1).

Model predictions:

The habitat suitability map (Figure 2a) appears to reflect the underlying data reasonably well with the set of "best" models predicting presence (and absence) to a mean AUC of 0.70. However, occurrence is predicted across some areas, particularly south west Scotland and Cumbria, where few sightings have been reported. Overall, across 100 repetitions MaxEnt proved to be the most commonly selected modelling approach displaying the highest AUC 28% of the time followed by Random Forest (23%). By land cover the mean habitat suitability scores suggest observation is most likely in landscapes dominated by coniferous woodland (Table 1) and, consistent with this, the majority of occurrence is predicted in coniferous woodland, acid grassland and improved grassland dominated habitat.

Both minimum and maximum density estimates were best fitted to the square of habitat suitability accounting for spherical spatial autocorrelation suggesting a strong relationship.

Whilst the predicted abundance range does not contain the estimate from Harris et al. (1995) our prediction suggests a significant increase in total population consistent with recently reported trends (post 1995). This may explain the overestimation although it is unclear whether the reported increase is driven by a range expansion consistent with model predictions (coverage of distribution approximately 1.5 times larger compared with observed distribution).

Reliability (Expert comment):

The majority of core regions of widespread occurrence represented by the observations appear reasonable but the small outlying patches are not and are likely misidentifications. In general, the habitat suitability map is plausible but suggests a high likelihood of observation in Dumfries and Cumbria than would be expected from current knowledge. Consistent with the majority of predicted occurrence sika deer are typically associated with landscapes dominated by acid grassland and coniferous woodland. There is no specific association with calcareous grassland; this may be an incidental association driven by occurrence on the south coast rather than any meaningful ecological dependence. The abundance range is on the high side but the minimum limit is plausible. The initial figure from Harris et al. (1995) could be considered a slight underestimation and it perhaps reasonable to suggest that the true abundance lies somewhere between the two estimates.

References:

Harris, S. J., P. Morris, S. Wray and D. Yalden (1995). A review of British mammals: population estimates and conservation status of British mammals other than cetaceans, Joint Nature Conservation Committee, Peterborough, UK.

Marques, F. F. C., S. T. Buckland, D. Goffin, C. E. Dixon, D. L. Borchers, B. A. Mayle and A. J. Peace (2001). Estimating deer abundance from line transect surveys of dung: sika deer in southern Scotland. *Journal of Applied Ecology* 38(2): 349-363.

Putman, R. J. and J. R. Clifton-Bligh (1997). Age-related body weight, fecundity and population change in a south Dorset sika population (*Cervus nippon*); 1985–1993. *Journal of Natural History* 31(4): 649-660.

Table 1: Summary of observed data and model predictions by land cover class (LCM2007 target classification). Values shown in brackets denote the spatial coverage based on a 10km resolution raster map (number of grid cells). Years represent the median of records within each land class. Ranges for density and abundance are derived using the respective minimum and maximum raster maps (lower bound is mean of values across minimum raster map with upper across the maximum) which capture the spatial uncertainty generate by projecting irregular polygons describing survey sites onto a raster grid.

LCM2007 class	Observed					Predicted		
	Occurrence		Density					
	Records	Year	Estimates	Year	Range	Habitat suitability	Density	Abundance
1 (Broadleaved woodland)	50 (4)	2007	0 (0)	-	-	0.72 (6)	0.36 - 5.38	217.8 - 3,227
2 (Coniferous woodland)	436 (78)	1994	22 (9)	1997	0.14 - 0.52	0.84 (134)	0.44 - 4.99	5,867 - 66,908
3 (Arable and Horticultural)	159 (70)	2000	7 (5)	1998	0.15 - 7.16	0.44 (55)	0.12 - 4.38	636.9 - 24,088
4 (Improved grassland)	282 (77)	2000	31 (15)	1997	0.83 - 3.56	0.51 (141)	0.12 - 4.42	1,693 - 62,327
5 (Rough grassland)	18 (5)	1997	0 (0)	-	-	0.38 (12)	0.14 - 3.81	166.8 - 4,571
6 (Neutral grassland)	0 (0)	-	0 (0)	-	-	0.17 (0)	-	-
7 (Calcareous grassland)	2 (1)	2001	0 (0)	-	-	0.83 (2)	0.21 - 5.08	41.96 - 1,015
8 (Acid grassland)	513 (70)	1993	40 (14)	1998	0.56 - 2.17	0.74 (150)	0.32 - 5.14	4,853 - 77,094
9 (Fen, Marsh, and Swamp)	0 (0)	-	0 (0)	-	-	-	-	-
10 (Heather)	108 (25)	1993	3 (1)	1998	0.11 - 1.34	0.77 (48)	0.36 - 5.01	1,719 - 24,043
11 (Heather grassland)	164 (44)	1999	2 (1)	1998	0.1 - 1.84	0.64 (83)	0.34 - 4.34	2,788 - 36,032
12 (Bog)	111 (34)	1999	0 (0)	-	-	0.53 (61)	0.37 - 5.1	2,232 - 31,092
13 (Montane habitat)	104 (20)	1998	0 (0)	-	-	0.76 (39)	0.27 - 5.19	1,056 - 20,229
14 (Inland rock)	0 (0)	-	0 (0)	-	-	0.33 (0)	-	-
15 (Saltwater)	16 (3)	1995	0 (0)	-	-	0.57 (5)	0.14 - 3.02	68.46 - 1,508
16 (Freshwater)	0 (0)	-	0 (0)	-	-	0.52 (1)	0.06 - 5.02	5.94 - 502.1
17 (Supra-littoral rock)	0 (0)	-	0 (0)	-	-	0.23 (0)	-	-
18 (Supra-littoral sediment)	0 (0)	-	0 (0)	-	-	0.34 (0)	-	-
19 (Littoral rock)	0 (0)	-	0 (0)	-	-	0.29 (0)	-	-
20 (Littoral sediment)	1 (1)	1974	5 (4)	1997	0.05 - 0.25	0.49 (8)	0.14 - 4.77	112.9 - 3,817
21 (Saltmarsh)	0 (0)	-	0 (0)	-	-	-	-	-
22 (Urban)	0 (0)	-	0 (0)	-	-	0.21 (0)	-	-
23 (Suburban)	6 (4)	1994	0 (0)	-	-	0.37 (3)	0.06 - 4.49	18.87 - 1,347
Total	1,970 (436)	1999	110 (49)	1997	0.47 - 2.61	0.53 (748)	0.29 - 4.78	21,477 - 357,800

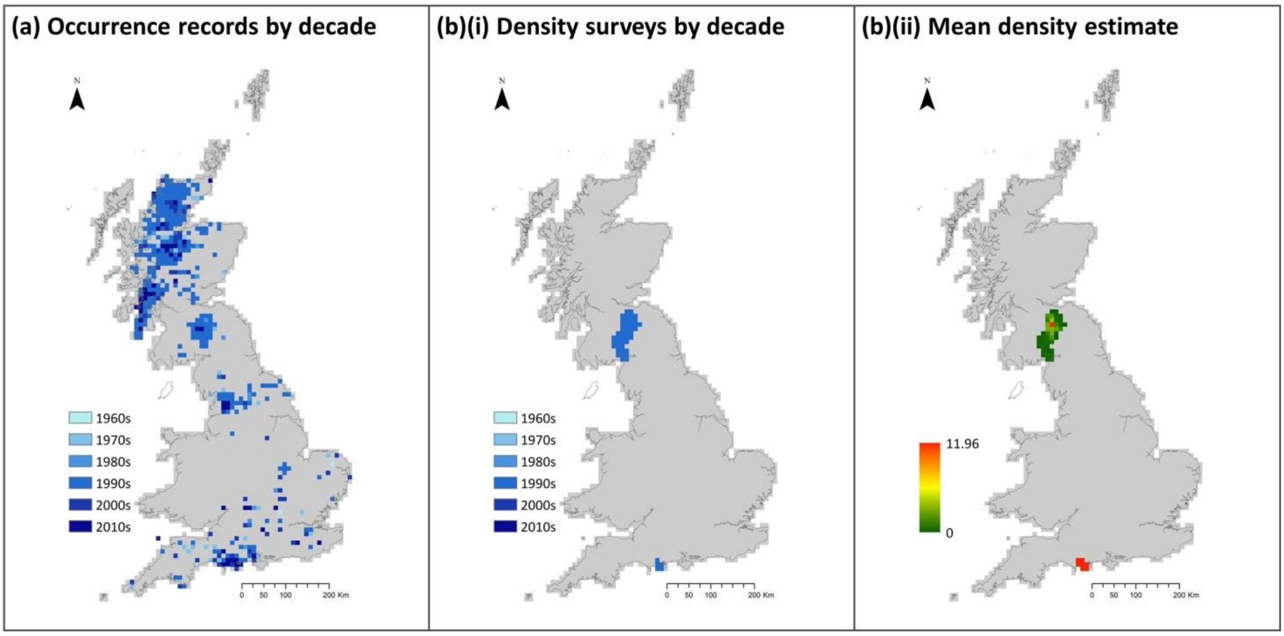

© Crown copyright and database rights 2016 Ordnance Survey 100051110. Data courtesy of the NBN Gateway with thanks to all data contributors. The NBN and its data contributors bear no responsibility for the further analysis or interpretation of this material, data and/or information.

Figure 1: 10km resolution raster maps based on BNG presenting the geographic description of available data. (a) shows the distribution of species occurrence obtained via the NBN Gateway categorised by the decade of last sighting. (b) shows information relating to density surveys identified via a search of published literature where: (i) categorises surveys by the decade of last survey; and (ii) shows the mean density estimate of surveys within grid cells (estimates assumed to be representative of entire cell, considered the upper limit of observed density).

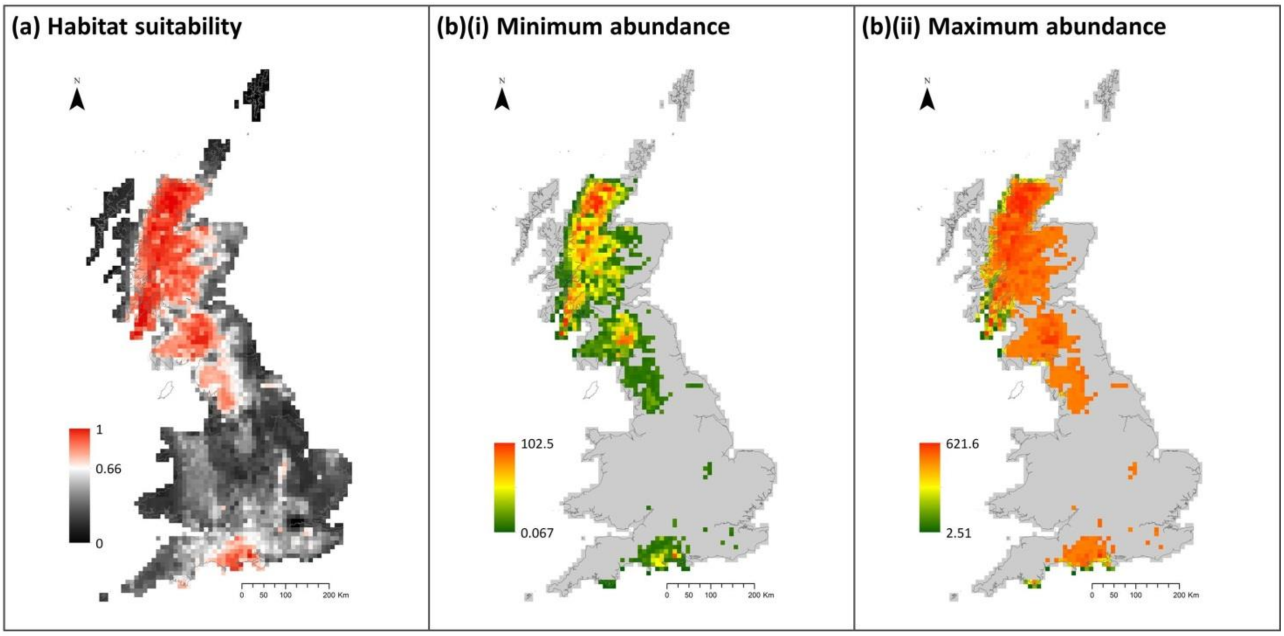

© Crown copyright and database rights 2016 Ordnance Survey 100051110. Data courtesy of the NBN Gateway with thanks to all data contributors. The NBN and its data contributors bear no responsibility for the further analysis or interpretation of this material, data and/or information.

Figure 2: Modelling predictions generated using systematic approach based on available data. (a) shows habitat suitability scores (the likelihood of observing the target species within each grid cell given variation environmental variables) determined by aggregating outputs from the "best" species distribution model (7 models compared) across 100 simulations. Here, the mid value on the scale denotes the threshold score above which occurrence is assumed. (b) shows: (i) the lower bound (Minimum); and (ii) the upper bound (Maximum); of abundance estimates determined by relating observed density (taking into account potential uncertainty) with habitat suitability scores using linear regression.
