# Supplementary material for: A systematic approach to estimate the distribution and total abundance of British mammals
Source: PLoS One. 2017 Jun 28;12(6):e0176339. doi: 10.1371/journal.pone.0176339 (PMC5489149; doi:10.1371/journal.pone.0176339)
Supplement: S3 File — Individual reports for each of the Artiodactyla species presenting analysis of the available data and subsequent model predictions based on a 10km raster grid. Reports also include expert comment assessing the reliability (and plausibility) of results in the context of existing evidence and popular opinion. (ZIP) [file pone.0176339.s003.zip › J Wild boar.pdf]

Wild boar (*Sus scrofa*)

Order: *Artiodactyla* **Genus:** *Sus*

Origin: Native **Status:** Rare

1995 abundance estimate: None **Reported population trends:** None

Data:

The available occurrence records indicate that wild boar are rare in Britain with some scattered sightings in southern parts of England and northern Scotland (Figure 1a). These reports are relatively recent (post 1995) with the majority observed in arable dominated habitats.

From the literature review we identified a single study (Wilson 2003) reporting density estimates at two locations in the south of England (one for the Weald and the other in Dorset) for 1997 (Figure 1b). Estimates ranged between 0.02 and 0.35 per km^2 with the highest densities recorded in habitats dominated by arable land cover (0.01 - 0.07 per km^2 accounting for uncertainty relating to unsurveyed areas within grid cells). Unfortunately, these surveys only sampled a limited selection of dominant land covers, consequently estimates were unavailable for some habitats where occurrence was observed notably coniferous woodland (marked grey in Table 1).

Model predictions:

The habitat suitability map (Figure 2a) appears to reflect the underlying data (combined occurrence from reported sightings and density estimates) well with the set of "best" models predicting presence (and absence) to a mean AUC of 0.80. However, some of the scattered populations in Scotland and East Anglian are not captured perhaps due to an overrepresentation of occurrence in the Weald. Overall, across 100 repetitions MaxEnt proved to be the most commonly selected modelling approach displaying the highest AUC 24% of the time followed by Support Vector Machines and Domain (17%). By land cover the mean habitat suitability scores suggest observation is most likely in landscapes dominated by broadleaved woodland (Table 1) but, consistent with recorded sightings, the majority of occurrence is predicted in arable and improved grassland (the most common dominant land covers at a 10km scale).

Neither minimum nor maximum density estimates showed a correlation with habitat suitability; perhaps due to the limited volume and variability of density data. However, maximum density did suggest a best fit using spherical spatial autocorrelation. An estimate was not provided in the 1995 report but our predictions suggest a total population between 188 and 3,011 within Britain.

Reliability (Expert comment):

The locations of reported sightings appear consistent with known populations of wild boar in Britain. Whilst the density range provided by published studies also seems reasonable the application of an estimate across such a wide geographical area as the Weald seems inappropriate given the highly localised occurrence. The use of this density estimate as an indication of occurrence introduces a clear bias in the habitat suitability map and this perhaps explains the failure to predict occurrence in Scotland. Consequently, the resulting distribution is implausible with coverage too broad in the south east of England and too restricted in the Forest of Dean (Gloucestershire). Despite this it is reasonable to suggest that the true value lies within the predicted range. However, this may simply be a fortunate coincidence and it would be difficult to support conclusions based on these figures with any degree of certainty given the distribution on which they are based. We are aware of unpublished data (e.g. Forest of Dean) that may markedly improve this analysis, but have avoided adding such data to this initial purely systematic approach.

References:

Wilson, C. J. (2003). Distribution and status of feral wild boar *Sus scrofa* in Dorset, southern England. *Mammal Review* 33(3-4): 302-307.

Table 1: Summary of observed data and model predictions by land cover class (LCM2007 target classification). Values shown in brackets denote the spatial coverage based on a 10km resolution raster map (number of grid cells). Years represent the median of records within each land class. Ranges for density and abundance are derived using the respective minimum and maximum raster maps (lower bound is mean of values across minimum raster map with upper across the maximum) which capture the spatial uncertainty generate by projecting irregular polygons describing survey sites onto a raster grid.

LCM2007 class	Observed					Predicted		
	Occurrence		Density					
	Records	Year	Estimates	Year	Range	Habitat suitability	Density	Abundance
1 (Broadleaved woodland)	0 (0)	-	5 (5)	1997	0.02	0.63 (7)	0.01 - 0.18	7.87 - 126.2
2 (Coniferous woodland)	14 (3)	2013	0 (0)	-	-	0.21 (1)	0.01 - 0.18	1.12 - 18.03
3 (Arable and Horticultural)	18 (12)	2012	31 (32)	1997	0.01 - 0.07	0.31 (83)	0.01 - 0.17	90.14 - 1,445
4 (Improved grassland)	25 (6)	2007	51 (51)	1997	0.02 - 0.03	0.29 (74)	0.01 - 0.17	79.21 - 1,270
5 (Rough grassland)	0 (0)	-	1 (1)	1997	0.02	0.14 (1)	0.01 - 0.13	0.84 - 13.45
6 (Neutral grassland)	0 (0)	-	0 (0)	-	-	0.09 (0)	-	-
7 (Calcareous grassland)	0 (0)	-	0 (0)	-	-	0.55 (2)	0.01 - 0.18	2.25 - 36.06
8 (Acid grassland)	3 (2)	2012	0 (0)	-	-	0.19 (0)	-	-
9 (Fen, Marsh, and Swamp)	0 (0)	-	0 (0)	-	-	-	-	-
10 (Heather)	0 (0)	-	0 (0)	-	-	0.17 (0)	-	-
11 (Heather grassland)	0 (0)	-	0 (0)	-	-	0.14 (0)	-	-
12 (Bog)	0 (0)	-	0 (0)	-	-	0.13 (0)	-	-
13 (Montane habitat)	0 (0)	-	0 (0)	-	-	0.16 (0)	-	-
14 (Inland rock)	0 (0)	-	0 (0)	-	-	0.14 (0)	-	-
15 (Saltwater)	0 (0)	-	0 (0)	-	-	0.26 (0)	-	-
16 (Freshwater)	0 (0)	-	0 (0)	-	-	0.19 (0)	-	-
17 (Supra-littoral rock)	0 (0)	-	0 (0)	-	-	0.12 (0)	-	-
18 (Supra-littoral sediment)	0 (0)	-	1 (1)	1997	0.02	0.24 (1)	0 - 0.05	0.34 - 5.38
19 (Littoral rock)	0 (0)	-	0 (0)	-	-	0.14 (0)	-	-
20 (Littoral sediment)	0 (0)	-	1 (1)	1997	0.02	0.25 1)	0	0.01 - 0.17
21 (Saltmarsh)	0 (0)	-	0 (0)	-	-	-	-	-
22 (Urban)	0 (0)	-	0 (0)	-	-	0.2 (0)	-	-
23 (Suburban)	0 (0)	-	7 (7)	1997	0.01 - 0.02	0.31 (9)	0.01 - 0.11	6.07 - 97.35
Total	60 (23)	2011	97 (98)	1997	0.02 - 0.04	0.26 (179)	0.01 - 0.17	187.9 - 3,011

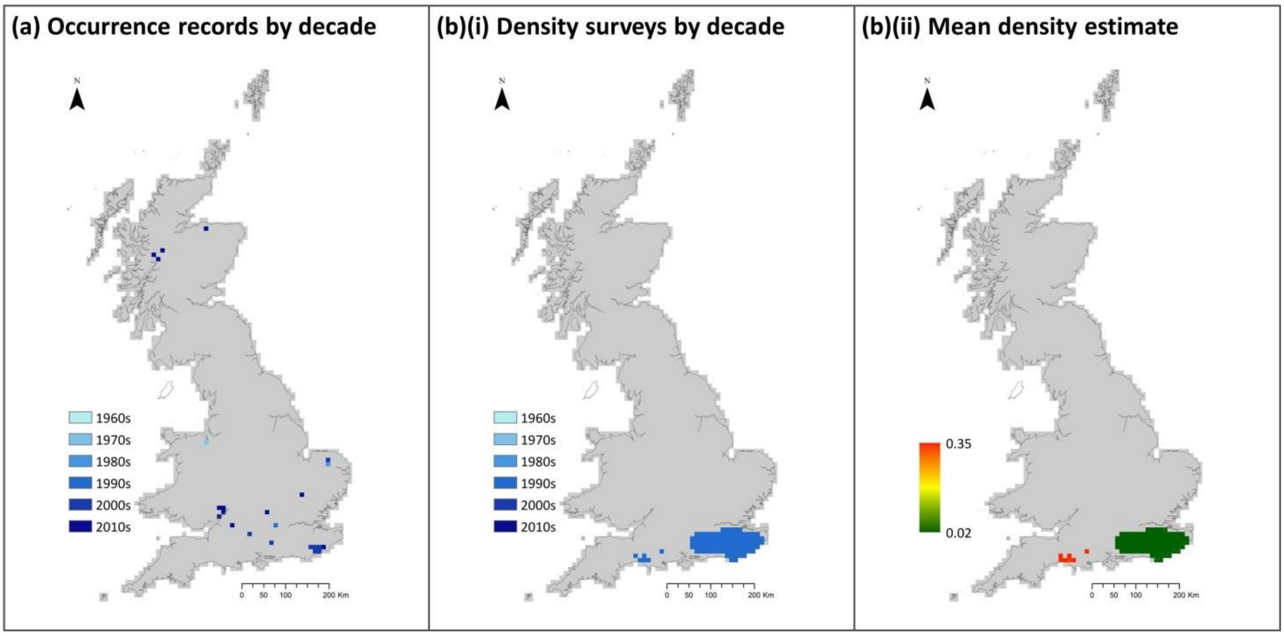

© Crown copyright and database rights 2016 Ordnance Survey 100051110. Data courtesy of the NBN Gateway with thanks to all data contributors. The NBN and its data contributors bear no responsibility for the further analysis or interpretation of this material, data and/or information.

Figure 1: 10km resolution raster maps based on BNG presenting the geographic description of available data. (a) shows the distribution of species occurrence obtained via the NBN Gateway categorised by the decade of last sighting. (b) shows information relating to density surveys identified via a search of published literature where: (i) categorises surveys by the decade of last survey; and (ii) shows the mean density estimate of surveys within grid cells (estimates assumed to be representative of entire cell, considered the upper limit of observed density).

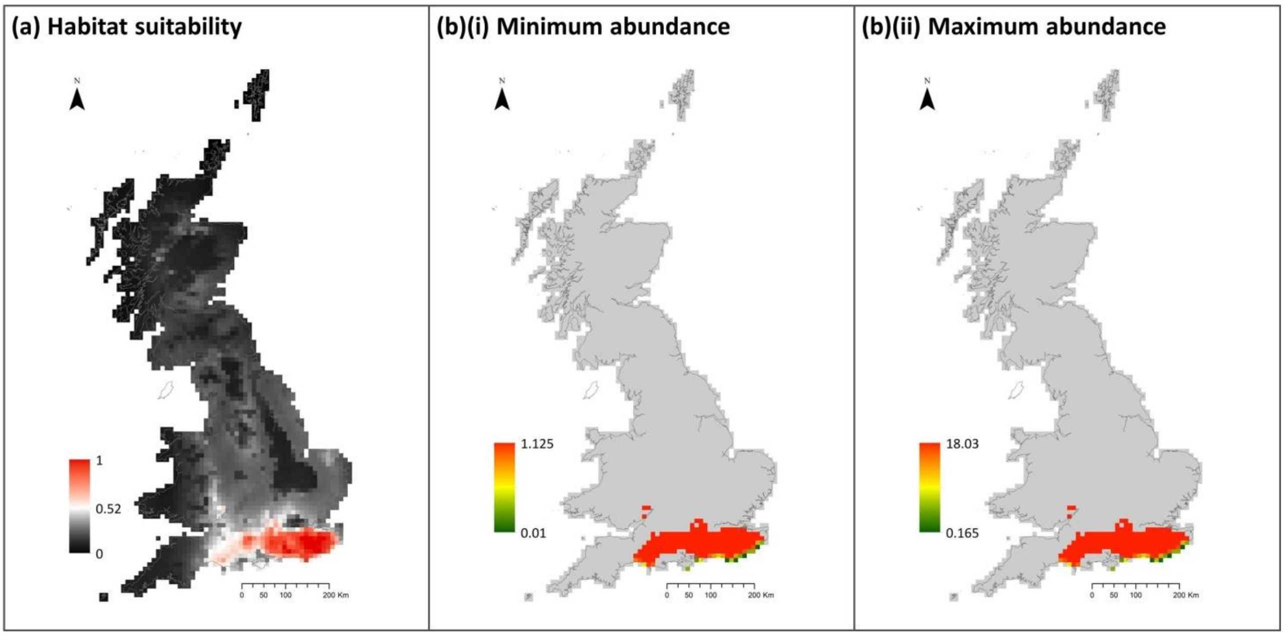

© Crown copyright and database rights 2016 Ordnance Survey 100051110. Data courtesy of the NBN Gateway with thanks to all data contributors. The NBN and its data contributors bear no responsibility for the further analysis or interpretation of this material, data and/or information.

Figure 2: Modelling predictions generated using systematic approach based on available data. (a) shows habitat suitability scores (the likelihood of observing the target species within each grid cell given variation environmental variables) determined by aggregating outputs from the "best" species distribution model (7 models compared) across 100 simulations. Here, the mid value on the scale denotes the threshold score above which occurrence is assumed. (b) shows: (i) the lower bound (Minimum); and (ii) the upper bound (Maximum); of abundance estimates determined by relating observed density (taking into account potential uncertainty) with habitat suitability scores using linear regression.
