# Supplementary material for: A systematic approach to estimate the distribution and total abundance of British mammals
Source: PLoS One. 2017 Jun 28;12(6):e0176339. doi: 10.1371/journal.pone.0176339 (PMC5489149; doi:10.1371/journal.pone.0176339)
Supplement: S4 File — Individual reports for each of the Carnivora species presenting analysis of the available data and subsequent model predictions based on a 10km raster grid. Reports also include expert comment assessing the reliability (and plausibility) of results in the context of existing evidence and popular opinion. (ZIP) [file pone.0176339.s004.zip › A American mink.pdf]

American mink (*Neovison vison*)

Order: *Carnivora*

Genus: *Mustela*

Origin: Introduced

Status: Common

1995 abundance estimate: 110,000 (3)

Reported population trends: JNCC 2005 (↔), NGC 2009 (↓)

Data:

The available occurrence records indicate that American mink is widespread throughout GB with sightings reported in more than half of 10 km squares at least once (approximately 55%), most over the past decade (Figure 1a). However, the map highlights several areas, particularly in south west Scotland and the south of England, where the species has not been recorded for some time and other areas of northern Scotland, Wales and eastern England where the species has never been reported.

Density estimates, primarily recorded over the past two decades, were obtained from published literature spanning approximately 1% of the observed species distribution based on the available occurrence data (Birks 1981; Bonesi & Macdonald 2004; Harrington & Macdonald 2008; Moore et al. 2003). Geographically, these studies were conducted on rivers in areas of central and south western England with a large scale project surveying the isolated population within the Hebrides (Figure 1b). Estimates ranged between 1.62 and 70 per km^2 with the highest densities recorded in rough grassland (0.14 - 66.53 per km^2 accounting for uncertainty relating to unsurveyed areas within grid cells). In all cases reporting of density was based on linear geographic features comprising a small proportion of cell area. The uncertainty, and therefore the range, associated with estimates was consequently large. Despite the proportion of area surveyed estimates for several dominant land covers were not available (marked grey in Table 1).

Model predictions:

The habitat suitability map (Figure 2a) appears to reflect the underlying data reasonably well with the set of "best" models predicting presence (and absence) to a mean AUC of 0.69. Overall, across 100 repetitions Random Forest proved to be the most commonly selected modelling approach displaying the highest AUC 43% of the time followed by MaxEnt (33%). By land cover the mean habitat suitability scores suggest observation is most likely in landscapes dominated by calcareous grassland, broadleaved woodland and arable (Table 1) but, consistent with recorded sightings, the majority of occurrence is predicted in grid cells dominated by arable and improved grassland.

Most likely due to the limited number of density estimates linear regression suggested no correlation with habitat suitability. Instead, a constant mean estimate was applied to all cells where occurrence was predicted and summed to derive total abundance.

Nevertheless, the predicted abundance range contains the estimate from Harris et al. (1995) suggesting no change in the total population. Whilst this result disagrees with the latest reported trends there is scope within the range to argue that populations have declined. In order to better assess potential trends and reduce the size of predicted range more density estimates are required spanning a greater proportion of the observed distribution. For this species in particular reporting of densities based on a grid rather than linear features such as rivers would also substantially reduce the uncertainty of density estimates and therefore improve the precision of predicted abundance.

Reliability (Expert comment):

These predictions are considered to be of low reliability. While a number of studies have documented mink abundance, these have presented information as linear densities along water courses. This creates difficulties when converting to areal density estimates within specific habitat types, as is required for this approach. Such estimates could be derived from the original data produced by a number of the published studies, but more information is required than is available in the published record. Additionally, the distributional data for this species is sparse in some areas, particularly offshore islands such as the Hebrides where published studies have shown this species to be more widespread than indicated by the NBN data. Improving the coverage of the NBN in such areas would increase

the spatial reliability of these predictions. As a consequence, the population estimates contain large uncertainties, but the range of estimates encompasses previous population estimates for this species.

References:

Birks, J. D. S. (1981). Home range and territorial behaviour of the feral mink (*Mustela vision*) in Devon. Ph.D. Thesis, University of Exeter.

Bonesi, L. and D. W. Macdonald (2004). Evaluation of sign surveys as a way to estimate the relative abundance of American mink (*Mustela vison*). *Journal of Zoology* 262(1): 65-72.

Harrington, L. A. and D. W. Macdonald (2008). Estimating the relative abundance of American mink *Mustela vison* on lowland rivers: evaluation and comparison of two techniques. *European Journal of Wildlife Research* 54(1): 79-87.

Harris, S. J., P. Morris, S. Wray and D. Yalden (1995). A review of British mammals: population estimates and conservation status of British mammals other than cetaceans, Joint Nature Conservation Committee, Peterborough, UK.

Moore, N. P., S. S. Roy and A. Helyar (2003). Mink (*Mustela vison*) eradication to protect ground-nesting birds in the Western Isles, Scotland, United Kingdom. *New Zealand Journal of Zoology* 30(4): 443-452.

Table 1: Summary of observed data and model predictions by land cover class (LCM2007 target classification). Values shown in brackets denote the spatial coverage based on a 10km resolution raster map (number of grid cells). Years represent the median of records within each land class. Ranges for density and abundance are derived using the respective minimum and maximum raster maps (lower bound is mean of values across minimum raster map with upper across the maximum) which capture the spatial uncertainty generate by projecting irregular polygons describing survey sites onto a raster grid.

LCM2007 class	Observed					Predicted		
	Occurrence		Density					
	Records	Year	Estimates	Year	Range	Habitat suitability	Density	Abundance
1 (Broadleaved woodland)	51 (9)	2006	0 (0)	-	-	0.92 (10)	0.09 - 18.3	92.23 - 18,323
2 (Coniferous woodland)	706 (89)	2002	0 (0)	-	-	0.81 (74)	0.09 - 17.4	650 - 129,030
3 (Arable and Horticultural)	6,906 (676)	2007	15 (9)	2005	0.1 - 19.2	0.9 (800)	0.09 - 17.1	6,895 - 1,369,658
4 (Improved grassland)	4,298 (501)	2002	3 (1)	2000	0.06 - 10.8	0.84 (560)	0.09 - 17.1	4,823 - 958,103
5 (Rough grassland)	43 (10)	2004	5 (2)	2003	0.14 - 66.5	0.38 (2)	0.09 - 18.3	18.45 - 3,665
6 (Neutral grassland)	0 (0)	-	0 (0)	-	-	0.06 (0)	0	0
7 (Calcareous grassland)	10 (2)	2002	0 (0)	-	-	0.95 (2)	0.09 - 18.3	18.45 - 3,665
8 (Acid grassland)	352 (88)	1998	0 (0)	-	-	0.72 (51)	0.09 - 17.8	457.2 - 90,825
9 (Fen, Marsh, and Swamp)	0 (0)	-	0 (0)	-	-	-	0	0
10 (Heather)	160 (27)	2007	0 (0)	-	-	0.75 (26)	0.09 - 17.6	230.6 - 45,813
11 (Heather grassland)	263 (48)	2006	6 (2)	2003	0.08 - 43.3	0.59 (25)	0.09 - 17.2	217 - 43,110
12 (Bog)	125 (37)	1994	9 (3)	2003	0.39 - 32.5	0.49 (12)	0.09 - 18.3	110.7 - 21,988
13 (Montane habitat)	46 (17)	1997	0 (0)	-	-	0.63 (1)	0.09 - 18.3	9.22 - 1,832
14 (Inland rock)	1 (1)	2006	0 (0)	-	-	0.68 (0)	0	0
15 (Saltwater)	13 (4)	2006	0 (0)	-	-	0.69 (0)	0	0
16 (Freshwater)	5 (2)	1994	0 (0)	-	-	0.69 (2)	0.09 - 18.2	18.29 - 3,633
17 (Supra-littoral rock)	0 (0)	-	0 (0)	-	-	0.25 (0)	0	0
18 (Supra-littoral sediment)	23 (2)	2010	0 (0)	-	-	0.54 (1)	0.02 - 3.5	1.74 - 346.5
19 (Littoral rock)	10 (2)	2005	0 (0)	-	-	0.46 (0)	0	0
20 (Littoral sediment)	37 (9)	2002	0 (0)	-	-	0.61 (0)	0	0
21 (Saltmarsh)	0 (0)	-	0 (0)	-	-	-	0	0
22 (Urban)	23 (4)	2012	0 (0)	-	-	0.69 (1)	0.01 - 2.64	1.33 - 263.9
23 (Suburban)	294 (40)	2011	0 (0)	-	-	0.76 (20)	0.09 - 17.1	171.9 - 34,139
Total	13,366 (1,568)	2006	38 (17)	2003	0.15 - 29.4	0.79 (1587)	0.09 - 17.2	13,714 - 2,724,393

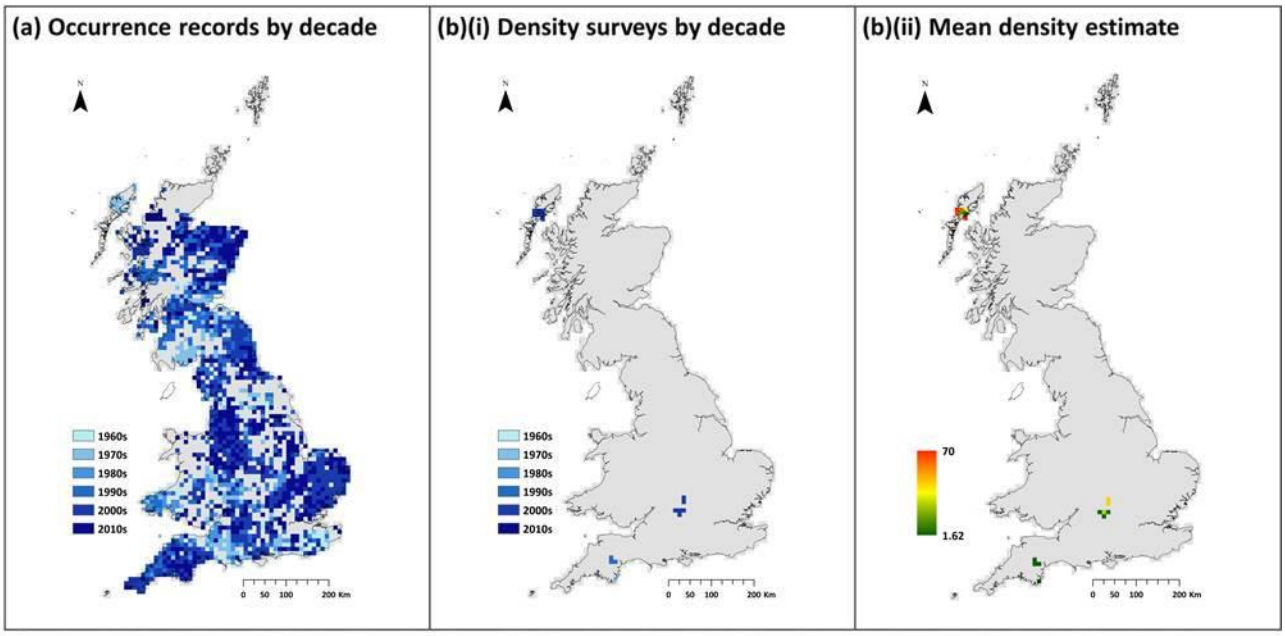

© Crown copyright and database rights 2016 Ordnance Survey 100051110. Data courtesy of the NBN Gateway with thanks to all data contributors. The NBN and its data contributors bear no responsibility for the further analysis or interpretation of this material, data and/or information.

Figure 1: 10km resolution raster maps based on BNG presenting the geographic description of available data. (a) shows the distribution of species occurrence obtained via the NBN Gateway categorised by the decade of last sighting. (b) shows information relating to density surveys identified via a search of published literature where: (i) categorises surveys by the decade of last survey; and (ii) shows the mean density estimate of surveys within grid cells (estimates assumed to be representative of entire cell, considered the upper limit of observed density).

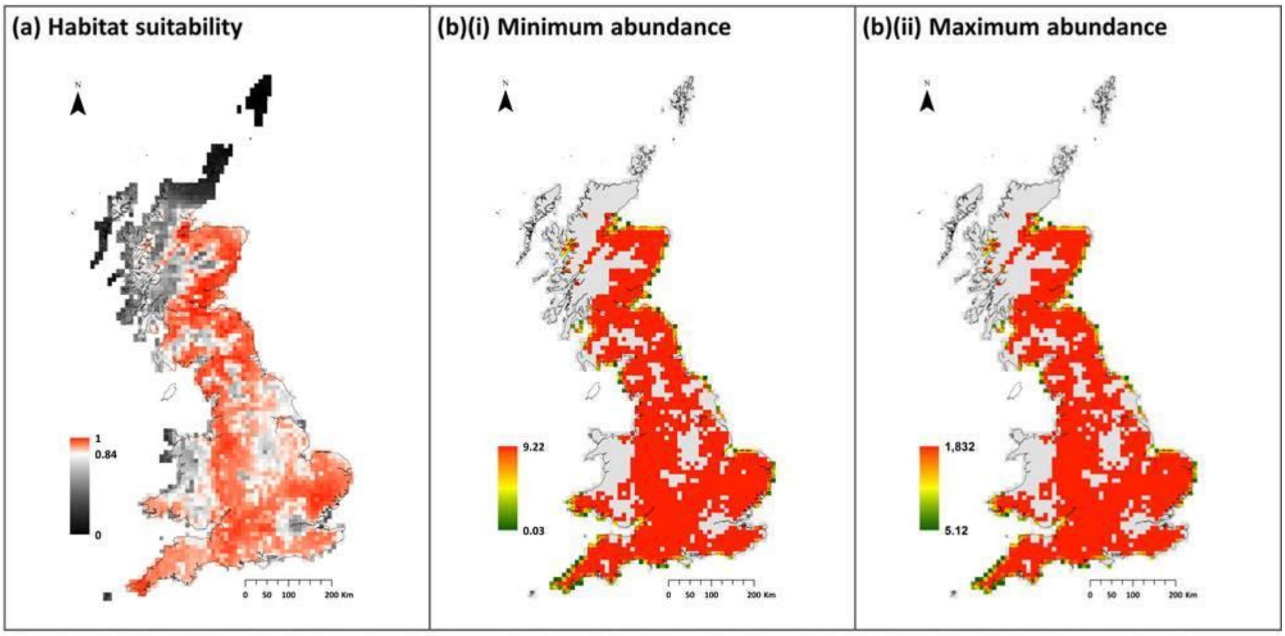

© Crown copyright and database rights 2016 Ordnance Survey 100051110. Data courtesy of the NBN Gateway with thanks to all data contributors. The NBN and its data contributors bear no responsibility for the further analysis or interpretation of this material, data and/or information.

Figure 2: Modelling predictions generated using systematic approach based on available data. (a) shows habitat suitability scores (the likelihood of observing the target species within each grid cell given variation environmental variables) determined by aggregating outputs from the "best" species distribution model (7 models compared) across 100 simulations. Here, the mid value on the scale denotes the threshold score above which occurrence is assumed. (b) shows: (i) the lower bound (Minimum); and (ii) the upper bound (Maximum); of abundance estimates determined by relating observed density (taking into account potential uncertainty) with habitat suitability scores using linear regression.
