# Supplementary material for: A systematic approach to estimate the distribution and total abundance of British mammals
Source: PLoS One. 2017 Jun 28;12(6):e0176339. doi: 10.1371/journal.pone.0176339 (PMC5489149; doi:10.1371/journal.pone.0176339)
Supplement: S4 File — Individual reports for each of the Carnivora species presenting analysis of the available data and subsequent model predictions based on a 10km raster grid. Reports also include expert comment assessing the reliability (and plausibility) of results in the context of existing evidence and popular opinion. (ZIP) [file pone.0176339.s004.zip › B Badger.pdf]

Badger (*Meles meles*)

Order: *Carnivora* **Genus:** *Meles*

Origin: Native **Status:** Common

1995 abundance estimate: 250,000 (1) **Reported population trends:** JNCC 2005 (↑)

Data:

The available occurrence records indicate that the badger is widespread throughout GB with sightings reported in most 10 km squares (approximately 77%) at least once over the past decade (Figure 1a). However, the map highlights several areas, particularly in Scotland, where the species has not been recorded for some time or not at all.

Density estimates, primarily recorded over the past two decades, were obtained from published literature spanning approximately 13% of the observed species distribution based on the available occurrence data (Delahay et al. 2006; Heydon et al. 2000; Hounsome et al. 2005; Hutchings et al. 2001, 2002; Macdonald & Newman 2002; Macdonald et al. 2009; Palphramand et al. 2007; Parrott et al. 2012; Rogers et al .1997; Roper et al. 1993). Geographically, these studies were concentrated in areas of south west England and Wales with one estimate in northern England but none in the south east or Scotland (Figure 1b). Estimates ranged between 1.16 and 36.4 per km^2 with the highest densities recorded in habitat dominated by supra-littoral sediment (3.68 - 4.84 per km^2 accounting for uncertainty relating to unsurveyed areas within grid cells). Despite the relatively high proportion of area surveyed estimates for several dominant land covers were not available (marked grey in Table 1).

Model predictions:

The habitat suitability map (Figure 2a) appears to reflect the underlying data well with the set of "best" models predicting presence (and absence) to a mean AUC of 0.79. Overall, across 100 repetitions MaxEnt proved to be the most commonly selected modelling approach displaying the highest AUC 40% of the time followed by Random Forest (29%) and Generalised Linear Models (12%). By land cover the mean habitat suitability scores suggest observation is most likely in landscapes dominated by calcareous grassland and broadleaved woodland closely followed by arable and suburban (Table 1) but, consistent with recorded sightings, the majority of occurrence is predicted in grid cells dominated by arable and improved grassland (the most common dominant land covers at a 10km scale). Occurrence is preserved in all other land covers where it is observed with the exception of littoral rock and urban dominated habitats.

Both minimum and maximum density estimates were best fitted to the square of habitat suitability accounting for spherical spatial autocorrelation with the highest abundance predicted in the south west of England.

The predicted abundance range contains the estimate from Harris et al. (1995) suggesting no significant change in the total population. Whilst this is not consistent with the latest reported trend there is scope within the range to argue a general increase is plausible (mean predicted abundance of 524,000).

Reliability (Expert comment):

The badger is a relatively common, though rarely seen, species in Britain. Published estimates to date have been a result of main sett surveys and an extrapolation based on the estimated number of badgers per main sett. These were performed in the mid-1980s, mid-1990s and early 2010s and indicated an increase in the number of badger social groups over this time period, but give much greater uncertainty in the change in population size as the number of individuals per social group may have changed. Only recently has there been a systematic estimate of social group size (not used in this modelling exercise and for England and Wales only: Judge et al. in press) to help extrapolation from the number of main setts. Based on the number of additional main setts in Scotland, this would give approximately 520,000 badgers in GB plus or minus 100,000 or so. The mean predicted abundance from the modelling is therefore almost identical to the current estimated population size and adds substantial weight to this approach.

References:

Delahay, R. J., N. Walker, G. J. Forrester, B. Harmsen, P. Riordan, D. W. Macdonald, C. Newman and C. L. Cheeseman (2006). Demographic correlates of bite wounding in European badgers (*Meles meles*) in stable and perturbed populations. *Animal Behaviour* 71(5): 1047-1055.

Harris, S. J., P. Morris, S. Wray and D. Yalden (1995). A review of British mammals: population estimates and conservation status of British mammals other than cetaceans, Joint Nature Conservation Committee, Peterborough, UK.

Heydon, M. J., J. C. Reynolds and M. J. Short (2000). Variation in abundance of foxes (*Vulpes vulpes*) between three regions of rural Britain, in relation to landscape and other variables. *Journal of Zoology* 251(2): 253-264.

Hounsome, T. D., R. P. Young, J. Davison, R. W. Yarnell, I. D. Trewby, B. T. Garnett, R. J. Delahay and G. J. Wilson (2005). An evaluation of distance sampling to estimate badger (*Meles meles*) abundance. *Journal of Zoology* 266(1): 81-87.

Hutchings, M. R., K. M. Service and S. Harris (2001). Defecation and urination patterns of badgers *Meles meles* at low density in south west England. *Acta Theriologica* 46(1): 87-96.

Hutchings, M. R., K. M. Service and S. Harris (2002). Is population density correlated with faecal and urine scent marking in European badgers (*Meles meles*) in the UK? *Mammalian Biology* 67(5): 286-293.

Judge, J., G. Wilson, R. MacArthur, R. J. Delahay (submitted). How many badgers are there in England and Wales? Estimating badger social group size and abundance from genotyping trapped hairs. *Science Reports*.

Macdonald, D. W. and C. Newman (2002). Population dynamics of badgers (*Meles meles*) in Oxfordshire, UK: numbers, density and cohort life histories, and a possible role of climate change in population growth. *Journal of Zoology* 256(1): 121-138.

Macdonald, D. W., C. Newman, P. M. Nouvellet and C. D. Buesching (2009). An analysis of Eurasian badger (*Meles meles*) population dynamics: implications for regulatory mechanisms. *Journal of Mammalogy* 90(6): 1392-1403.

Palphramand, K. L., G. Newton-Cross and P. C. L. White (2007). Spatial organization and behaviour of badgers (*Meles meles*) in a moderate-density population. *Behavioral Ecology and Sociobiology* 61(3): 401-413.

Parrott, D., A. Prickett, S. Pietravalle, T. R. Etherington and M. Fletcher (2012). Estimates of regional population densities of badger *Meles meles*, fox *Vulpes vulpes* and hare *Lepus europaeus* using walked distance sampling. *European Journal of Wildlife Research* 58(1): 23-33.

Rogers, L. M., C. L. Cheeseman, P. J. Mallinson and R. Clifton-Hadley (1997). The demography of a high-density badger (*Meles meles*) population in the west of England. *Journal of Zoology* 242(4): 705-728.

Roper, T. J., L. Conradt, J. Butler, S. E. Christian, J. Ostler and T. K. Schmid (1993). Territorial marking with faeces in badgers (*Meles meles*): a comparison of boundary and hinterland latrine use. *Behaviour* 127(3-4): 289-307.

Table 1: Summary of observed data and model predictions by land cover class (LCM2007 target classification). Values shown in brackets denote the spatial coverage based on a 10km resolution raster map (number of grid cells). Years represent the median of records within each land class. Ranges for density and abundance are derived using the respective minimum and maximum raster maps (lower bound is mean of values across minimum raster map with upper across the maximum) which capture the spatial uncertainty generate by projecting irregular polygons describing survey sites onto a raster grid.

LCM2007 class	Observed					Predicted		
	Occurrence		Density					
	Records	Year	Estimates	Year	Range	Habitat suitability	Density	Abundance
1 (Broadleaved woodland)	428 (11)	2013	0 (0)	-	-	0.99 (11)	0.69 - 6.51	764.45 - 7,161.2
2 (Coniferous woodland)	2,098 (129)	2008	4 (4)	2006	0.1 - 3.01	0.9 (102)	0.36 - 5.06	3662.7 - 51,633
3 (Arable and Horticultural)	21,181 (888)	2013	147 (128)	2006	0.47 - 3.04	0.94 (899)	0.43 - 5.13	38,849 - 461,483
4 (Improved grassland)	15,255 (669)	2012	131 (121)	2006	0.68 - 3.55	0.9 (677)	0.42 - 4.99	28,445 - 337,801
5 (Rough grassland)	89 (16)	2002	0 (0)	-	-	0.39 (2)	0.34 - 5.69	68.24 - 1,137
6 (Neutral grassland)	0 (0)	-	0 (0)	-	-	0 (0)	-	0
7 (Calcareous grassland)	430 (2)	2014	0 (0)	-	-	0.99 (2)	0.76 - 6.86	152.32 - 1,371.5
8 (Acid grassland)	1,305 (150)	1999	12 (11)	2006	0.33 - 2.66	0.86 (92)	0.35 - 5.38	3,181 - 49,478
9 (Fen, Marsh, and Swamp)	0 (0)	-	0 (0)	-	-	-	-	0
10 (Heather)	179 (39)	2000	0 (0)	-	-	0.8 (19)	0.34 - 5.11	641.54 - 9,712.5
11 (Heather grassland)	1480 (66)	2008	0 (0)	-	-	0.62 (10)	0.32 - 5.11	323.76 - 5,105.3
12 (Bog)	321 (49)	2004	2 (2)	2006	0.15 - 3.57	0.48 (14)	0.35 - 5.47	493.56 - 7,664
13 (Montane habitat)	108 (24)	1984	0 (0)	-	-	0.66 (1)	0.35 - 5.95	34.97 - 595.24
14 (Inland rock)	0 (0)	-	0 (0)	-	-	0.28 (0)	-	0
15 (Saltwater)	43 (7)	2008	0 (0)	-	-	0.82 (3)	0.07 - 1.17	21.57 - 350.49
16 (Freshwater)	9 (2)	1996	0 (0)	-	-	0.68 (2)	0.35 - 5.2	70.7 - 1,039.5
17 (Supra-littoral rock)	0 (0)	-	0 (0)	-	-	0.06 (0)	-	0
18 (Supra-littoral sediment)	29 (4)	1997	1 (1)	2006	3.68 - 4.84	0.65 (2)	0.05 - 0.69	9.34 - 138.51
19 (Littoral rock)	1 (1)	2011	0 (0)	-	-	0.4 (0)	-	0
20 (Littoral sediment)	216 (18)	2008	2 (2)	2006	0.21 - 2.82	0.8 (2)	0.25 - 4.24	50.86 - 848.09
21 (Saltmarsh)	0 (0)	-	0 (0)	-	-	-	-	0
22 (Urban)	57 (3)	2004	0 (0)	-	-	0.76 (0)	-	0
23 (Suburban)	1692 (71)	2012	1 (1)	2006	0.03 - 2.9	0.94 (70)	0.4 - 4.75	2,774.3 - 33,221
Total	44,921 (2,149)	2012	300 (270)	2006	0.56 - 3.26	0.85 (1,908)	0.42 - 5.08	79,544 - 968,740

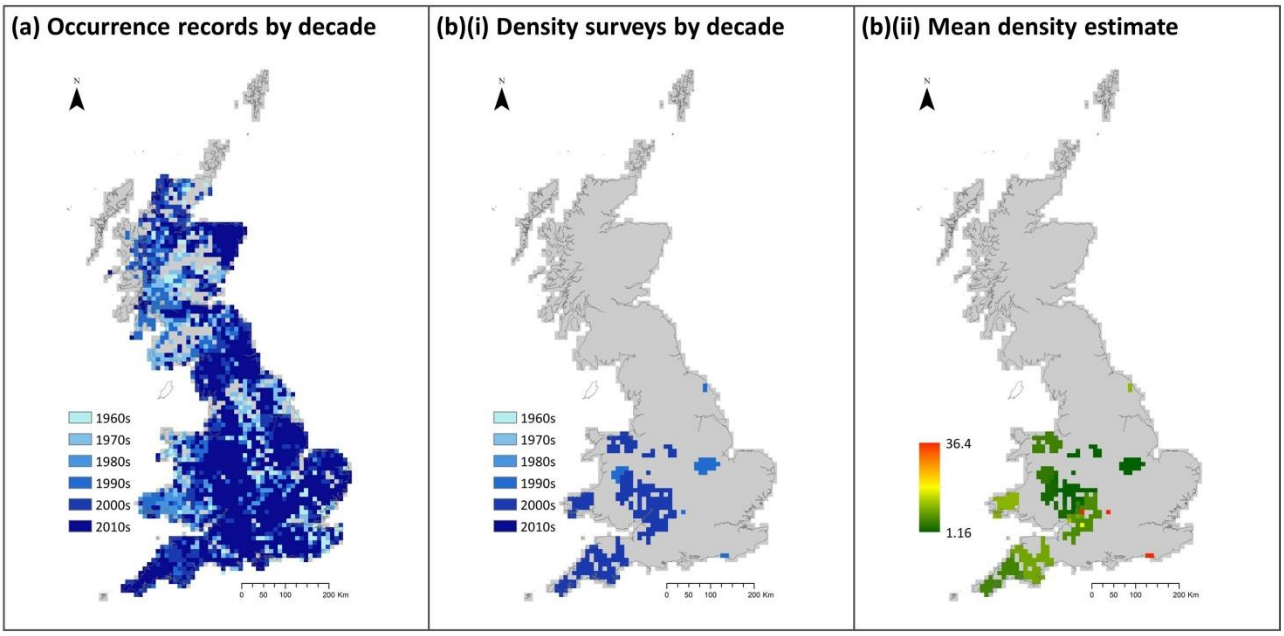

© Crown copyright and database rights 2016 Ordnance Survey 100051110. Data courtesy of the NBN Gateway with thanks to all data contributors. The NBN and its data contributors bear no responsibility for the further analysis or interpretation of this material, data and/or information.

Figure 1: 10km resolution raster maps based on BNG presenting the geographic description of available data. (a) shows the distribution of species occurrence obtained via the NBN Gateway categorised by the decade of last sighting. (b) shows information relating to density surveys identified via a search of published literature where: (i) categorises surveys by the decade of last survey; and (ii) shows the mean density estimate of surveys within grid cells (estimates assumed to be representative of entire cell, considered the upper limit of observed density).

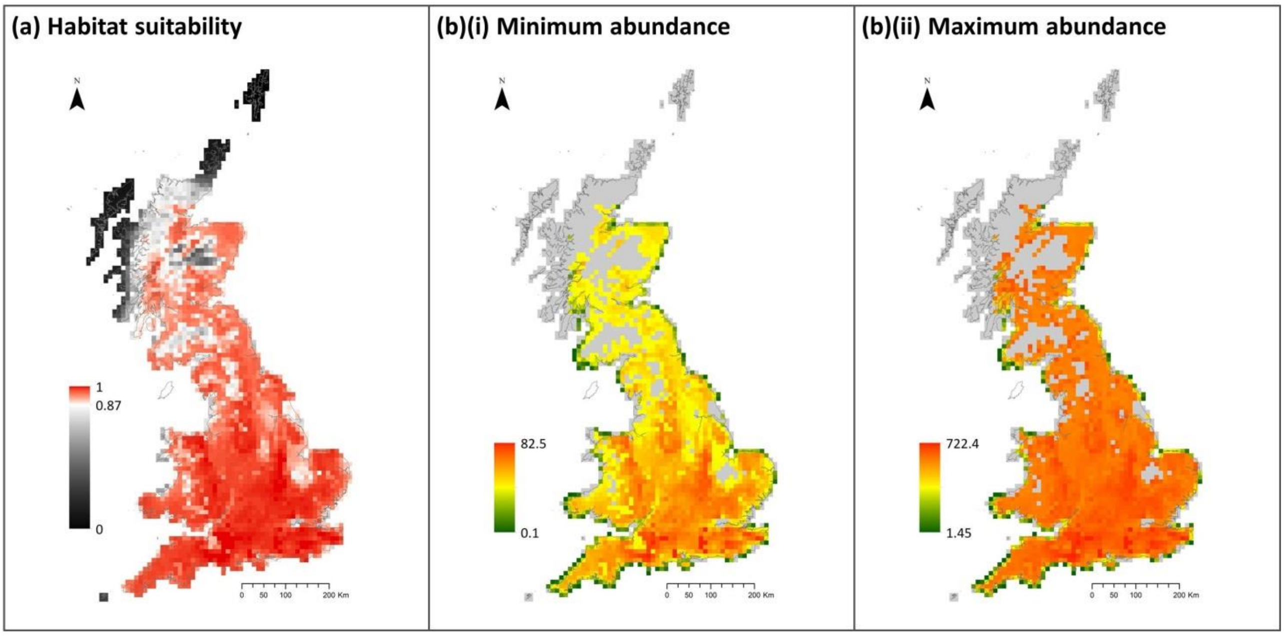

© Crown copyright and database rights 2016 Ordnance Survey 100051110. Data courtesy of the NBN Gateway with thanks to all data contributors. The NBN and its data contributors bear no responsibility for the further analysis or interpretation of this material, data and/or information.

Figure 2: Modelling predictions generated using systematic approach based on available data. (a) shows habitat suitability scores (the likelihood of observing the target species within each grid cell given variation environmental variables) determined by aggregating outputs from the "best" species distribution model (7 models compared) across 100 simulations. Here, the mid value on the scale denotes the threshold score above which occurrence is assumed. (b) shows: (i) the lower bound (Minimum); and (ii) the upper bound (Maximum); of abundance estimates determined by relating observed density (taking into account potential uncertainty) with habitat suitability scores using linear regression.
