# Supplementary material for: A systematic approach to estimate the distribution and total abundance of British mammals
Source: PLoS One. 2017 Jun 28;12(6):e0176339. doi: 10.1371/journal.pone.0176339 (PMC5489149; doi:10.1371/journal.pone.0176339)
Supplement: S4 File — Individual reports for each of the Carnivora species presenting analysis of the available data and subsequent model predictions based on a 10km raster grid. Reports also include expert comment assessing the reliability (and plausibility) of results in the context of existing evidence and popular opinion. (ZIP) [file pone.0176339.s004.zip › C Feral cat.pdf]

Feral cat (*Felis catus*)

Order: *Carnivora*

Genus: *Felis*

Origin: Introduced

Status: Locally common

1995 abundance estimate: 813,000 (4)

Reported population trends: NGC 2009 (↔)

Data:

The available occurrence records indicate that feral cats are locally distributed throughout GB with significant patches concentrated around urban areas in the south east and north west of England (Figure 1a). However, the map does highlight notable areas of absence, predominantly in Scotland. Sightings were reported in various habitats (predominantly arable and improved grassland) with the majority (of cells) where occurrence was observed containing at least one record since 1995.

From the literature review we identified two studies (Dards 1981; Page et al. 1992) conducted at dockyards in the south of England between 1977 and 1983 (Figure 1b). Estimates were relatively high, ranging between 6 and 200 per km^2 with the highest unsurprisingly recorded in habitats dominated by suburban land cover (1.21 - 106.3 per km^2). Unfortunately, due to the limited geographic area of the studies estimates were unavailable for most dominant habitats where occurrence was observed (marked grey in Table 1).

Model predictions:

The habitat suitability map (Figure 2a) appears to reflect the underlying data reasonably well with the set of "best" models predicting presence (and absence) to a mean AUC of 0.68. Overall, across 100 repetitions MaxEnt proved to be the most commonly selected modelling approach displaying the highest AUC 43% of the time followed by Generalised Linear Models (35%). By land cover the mean habitat suitability scores suggest observation is most likely in landscapes dominated by urban and suburban land cover (Table 1) but, consistent with recorded sightings, the majority of occurrence is predicted in grid cells dominated by arable and improved grassland (the most common dominant land covers at a 10km scale).

Both minimum and maximum density estimates were best fitted linearly to habitat suitability accounting for spherical spatial autocorrelation. However, these relationships suggest a negative correlation in which squares with a higher likelihood of observation contain a lower density of individuals. This trend explains the appearance of gaps in the distribution of predicted abundance around highly suitable urban areas as density becomes negative and is consequently removed. Whilst this could potentially be caused by recording bias or other factors present in these regions which are not captured by the model process it is most likely that the limited number of density estimates is responsible for this spurious result.

Despite these issues the predicted abundance range contains the estimate from Harris et al. (1995) suggesting, in agreement with recently reported trends, no significant change in the total population (this is perhaps unsurprising as some of the density estimates used here were considered in 1995; given this the result most likely indicates that the species distribution has not significantly expanded over the past 20 years). However, the predicted range is very large and given the concerns regarding the corresponding distribution additional survey efforts would be required to produce a more accurate estimate.

Reliability (Expert comment):

The population of feral cats is very hard to determine, especially since records can be confused with both the wildcat in Scotland and domestic cats. A feral cat by definition is one that is not owned, but it may still be reliant on human feeding. It is therefore impossible to determine if any sighted individual is feral or not and an individual may change status through its lifetime. Therefore the number of sightings is likely to be vastly inflated. We know that there are approximately 10 million domestic cats in the UK (Murray et al. 2010) and the model output encompasses this estimate. We also know that the areas surveyed in the 1980s had a reduced number of colonies, and cats per colony,

by the year 2000 (unpublished data). So if the total feral cat population actually halved since 1995, the model output also covers this estimate. For this species, although density estimation may be possible to accurately define, presence will always be overestimated, and the final output is some unknown combination of feral and domestic cats and therefore of little value.

References:

Dards, J. L. (1981). Habitat utilisation by feral cats in Portsmouth dockyard. *The Ecology And Control of Feral Cats*, Royal Holloway College, University of London, The Universities Federation for Animal Welfare.

Harris, S. J., P. Morris, S. Wray and D. Yalden (1995). A review of British mammals: population estimates and conservation status of British mammals other than cetaceans, Joint Nature Conservation Committee, Peterborough, UK.

Murray, J. K., W. J. Browne, M. A. Roberts, A. Whitmarsh, T. J. Gruffydd-Jones (2010). Number and ownership profiles of cats and dogs in the UK. *Veterinary Record*, 166: 163-168.

Page, R. J. C., J. Ross and D. H. Bennet (1992). A study of the home ranges, movements and behaviour of the feral cat population at Avonmouth Docks. *Wildlife Research* 19(3): 263-277.

Table 1: Summary of observed data and model predictions by land cover class (LCM2007 target classification). Values shown in brackets denote the spatial coverage based on a 10km resolution raster map (number of grid cells). Years represent the median of records within each land class. Ranges for density and abundance are derived using the respective minimum and maximum raster maps (lower bound is mean of values across minimum raster map with upper across the maximum) which capture the spatial uncertainty generate by projecting irregular polygons describing survey sites onto a raster grid.

LCM2007 class	Observed					Predicted		
	Occurrence		Density					
	Records	Year	Estimates	Year	Range	Habitat suitability	Density	Abundance
1 (Broadleaved woodland)	24 (3)	2008	0 (0)	-	-	0.65 (5)	4.1 - 363	2,052 - 181,598
2 (Coniferous woodland)	46 (12)	2007	0 (0)	-	-	0.34 (2)	5.77 - 510	1,153 - 101,955
3 (Arable and Horticultural)	2,664 (414)	2008	0 (0)	-	-	0.71 (708)	2.35 - 209	166,416 - 14,813,865
4 (Improved grassland)	1,730 (259)	2007	1 (1)	1983	0 - 12.5	0.62 (324)	2.3 - 205	74,525 - 6,631,402
5 (Rough grassland)	0 (0)	-	0 (0)	-	-	0.18 (0)	-	0
6 (Neutral grassland)	0 (0)	-	0 (0)	-	-	0.05 (0)	-	0
7 (Calcareous grassland)	0 (0)	-	0 (0)	-	-	0.47 (0)	-	0
8 (Acid grassland)	61 (18)	2004	0 (0)	-	-	0.31 (3)	4.39 - 389	1,316 - 116,655
9 (Fen, Marsh, and Swamp)	0 (0)	-	0 (0)	-	-	-	-	0
10 (Heather)	17 (3)	2000	0 (0)	-	-	0.27 (0)	-	0
11 (Heather grassland)	24 (8)	2008	0 (0)	-	-	0.28 (0)	-	0
12 (Bog)	26 (9)	2007	0 (0)	-	-	0.27 (4)	3.44 - 306	1,377 - 122,413
13 (Montane habitat)	0 (0)	-	0 (0)	-	-	0.07 (0)	-	0
14 (Inland rock)	0 (0)	-	0 (0)	-	-	0.11 (0)	-	0
15 (Saltwater)	6 (2)	2006	0 (0)	-	-	0.56 (0)	-	0
16 (Freshwater)	0 (0)	-	0 (0)	-	-	0.2 (0)	-	0
17 (Supra-littoral rock)	0 (0)	-	0 (0)	-	-	0.1 (0)	-	0
18 (Supra-littoral sediment)	0 (0)	-	1 (1)	1977	4.94 - 6	0.4 (0)	-	0
19 (Littoral rock)	2 (2)	2001	0 (0)	-	-	0.41 (2)	0.09 - 7.7	17.5 - 1,549
20 (Littoral sediment)	18 (8)	2004	0 (0)	-	-	0.57 (6)	3.08 - 273	1,847 - 163,622
21 (Saltmarsh)	0 (0)	-	0 (0)	-	-	-	-	0
22 (Urban)	126 (4)	2011	0 (0)	-	-	0.77 (4)	0	0
23 (Suburban)	1283 (52)	2011	2 (2)	1981	1.21 - 106.3	0.8 (76)	0.75 - 67.3	5,726 - 511,788
Total	6,027 (794)	2008	4 (4)	1981	1.84 - 57.8	0.55 (1,134)	2.24 - 200	254,430 - 22,644,848

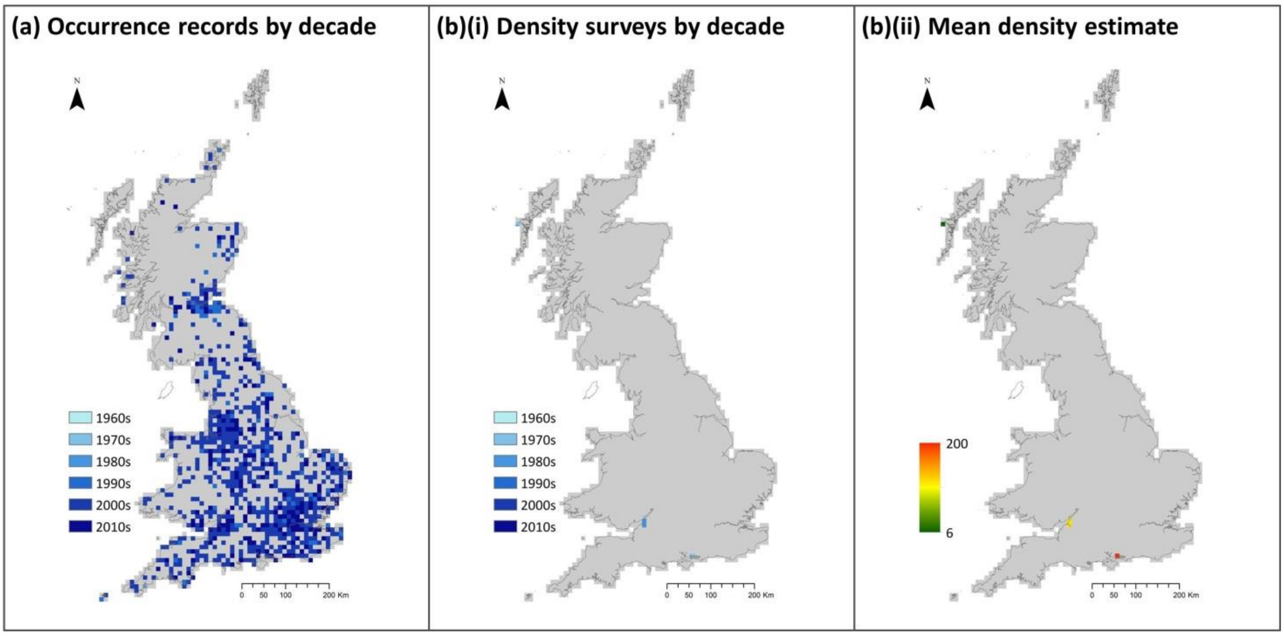

© Crown copyright and database rights 2016 Ordnance Survey 100051110. Data courtesy of the NBN Gateway with thanks to all data contributors. The NBN and its data contributors bear no responsibility for the further analysis or interpretation of this material, data and/or information.

Figure 1: 10km resolution raster maps based on BNG presenting the geographic description of available data. (a) shows the distribution of species occurrence obtained via the NBN Gateway categorised by the decade of last sighting. (b) shows information relating to density surveys identified via a search of published literature where: (i) categorises surveys by the decade of last survey; and (ii) shows the mean density estimate of surveys within grid cells (estimates assumed to be representative of entire cell, considered the upper limit of observed density).

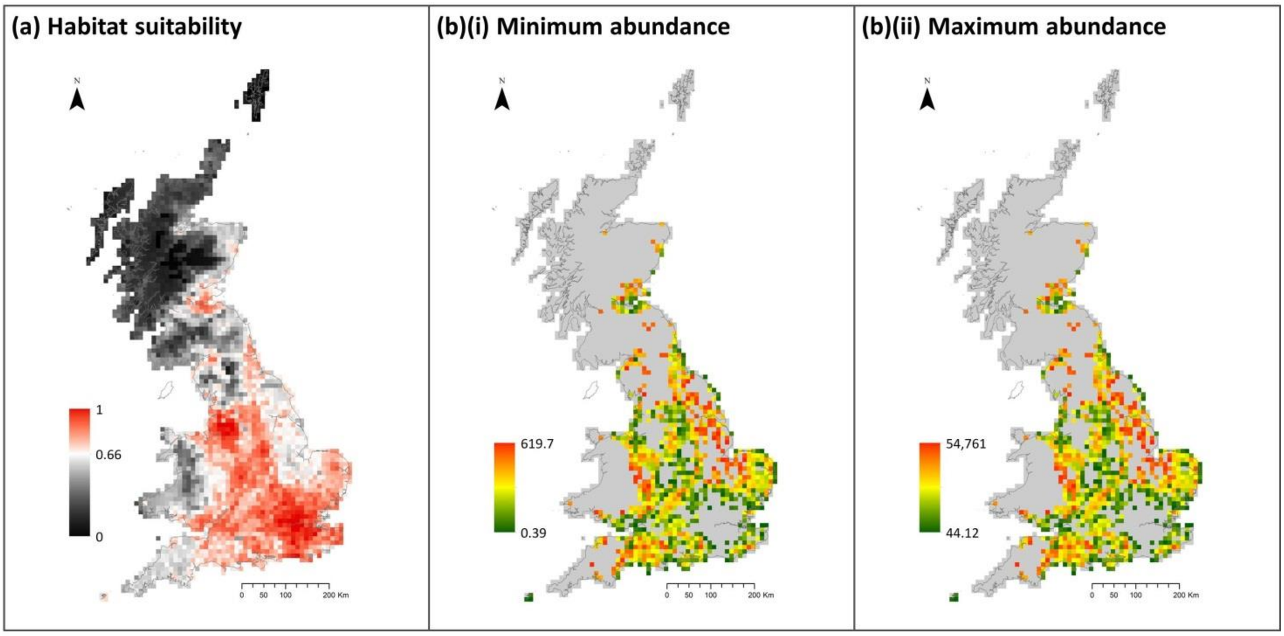

© Crown copyright and database rights 2016 Ordnance Survey 100051110. Data courtesy of the NBN Gateway with thanks to all data contributors. The NBN and its data contributors bear no responsibility for the further analysis or interpretation of this material, data and/or information.

Figure 2: Modelling predictions generated using systematic approach based on available data. (a) shows habitat suitability scores (the likelihood of observing the target species within each grid cell given variation environmental variables) determined by aggregating outputs from the "best" species distribution model (7 models compared) across 100 simulations. Here, the mid value on the scale denotes the threshold score above which occurrence is assumed. (b) shows: (i) the lower bound (Minimum); and (ii) the upper bound (Maximum); of abundance estimates determined by relating observed density (taking into account potential uncertainty) with habitat suitability scores using linear regression.
