# Supplementary material for: A systematic approach to estimate the distribution and total abundance of British mammals
Source: PLoS One. 2017 Jun 28;12(6):e0176339. doi: 10.1371/journal.pone.0176339 (PMC5489149; doi:10.1371/journal.pone.0176339)
Supplement: S4 File — Individual reports for each of the Carnivora species presenting analysis of the available data and subsequent model predictions based on a 10km raster grid. Reports also include expert comment assessing the reliability (and plausibility) of results in the context of existing evidence and popular opinion. (ZIP) [file pone.0176339.s004.zip › E Otter.pdf]

Otter (*Lutra lutra*)

Order: *Carnivora* **Genus:** *Lutra*

Origin: Native **Status:** Locally common

1995 abundance estimate: 7,350 (3) **Reported population trends:** JNCC 2005 (↑)

Data:

The available occurrence records indicate that the otter is widespread throughout GB with sightings reported in most 10 km squares (approximately 84%) at least once since 1995 (Figure 1a). However, the map highlights several areas, particularly in south east of England, where the species has not been recorded for some time or not at all.

Despite the ubiquity of the species, from the literature review we only identified a single survey (Kruuk et al. 1989) reporting an estimate of 4.42 per km^2 for the local population around the Shetland coastline in 1988 (Figure 1b). As a consequence, density estimates were not available for most dominant land covers where occurrence was observed (land class marked grey in Table 1), this included both saltwater and freshwater habitats for which the species is typically associated (although it should be noted these are poorly represented at a 10km scale).

Model predictions:

The habitat suitability map (Figure 2a) appears to reflect the underlying data reasonably well with the set of "best" models predicting presence (and absence) to a mean AUC of 0.77. However, there is a substantial contraction of the range in central and south eastern England. Overall, across 100 repetitions Random Forest proved to be the most commonly selected modelling approach displaying the highest AUC 37% of the time followed closely by MaxEnt (30%). By land cover the mean habitat suitability scores suggest observation is most likely in landscapes dominated by montane habitat, and neutral grassland (Table 1) but, consistent with recorded sightings, the majority of occurrence is predicted in grid cells dominated by arable and improved grassland (the most common dominant land covers at a 10km scale). Occurrence is preserved in all other land covers where it is observed with the exception of littoral rock and urban dominated habitats.

Linear regression suggested that there was no correlation between the estimates of maximum density and habitat suitability; consequently, it was applied as a constant in cells where occurrence is predicted. Minimum density was found to be correlated with the best fit model relating habitat suitability linearly and accounting for spherical spatial autocorrelation. However, most likely due to the limited number of density estimates this displayed a negative trend predicting the lowest density in cells of high suitability (although it may be an indication that the species is more easily observed in unsuitable habitats to which it is not adapted).

The predicted abundance range is larger than the estimate from Harris et al. (1995) suggesting a significant increase in the total population (at least 20 times). Whilst this is in agreement with recently reported trends it is unlikely that the magnitude of the increase is feasible. The overestimation is most likely due to the association of high density estimates recorded in specialist habitat (i.e. saltwater) with other dominant land covers at a 10km scale. This is a particular issue for modelling species which are best described using linear features with overestimations predicted for most species where this is the case. It is possible that this could be resolved by applying models based on a finer scale raster grid where such habitats are better represented.

Reliability (Expert comment):

As the occurrence records indicate this species has decline since the 1960s, although it is currently recovering quite well. For this species the occurrence data used should be more restricted in time. Despite this, the habitat suitability map does not include areas (e.g. Yorkshire) where they are known to occur, so it appears to underestimate the total range of this species. However, a single density estimate from prime habitat clearly has an effect on the total abundance, with the lower estimate being about an order of magnitude too high. As a riverine species, this is hard to ascribe a density per square kilometre and using that approach may well have inflated the estimate as well.

References:

Harris, S. J., P. Morris, S. Wray and D. Yalden (1995). A review of British mammals: population estimates and conservation status of British mammals other than cetaceans, Joint Nature Conservation Committee, Peterborough, UK.

Kruuk, H., A. Moorhouse, J. W. H. Conroy, L. Durbin and S. Frears (1989). An estimate of numbers and habitat preferences of otters *Lutra lutra* in Shetland, UK. *Biological Conservation* 49(4): 241-254.

Table 1: Summary of observed data and model predictions by land cover class (LCM2007 target classification). Values shown in brackets denote the spatial coverage based on a 10km resolution raster map (number of grid cells). Years represent the median of records within each land class. Ranges for density and abundance are derived using the respective minimum and maximum raster maps (lower bound is mean of values across minimum raster map with upper across the maximum) which capture the spatial uncertainty generate by projecting irregular polygons describing survey sites onto a raster grid.

LCM2007 class	Observed					Predicted		
	Occurrence		Density					
	Records	Year	Estimates	Year	Range	Habitat suitability	Density	Abundance
1 (Broadleaved woodland)	242 (6)	2002	0 (0)	-	-	0.72 (2)	1.16 - 4.42	233 - 884
2 (Coniferous woodland)	3,270 (154)	2002	0 (0)	-	-	0.95 (154)	0.67 - 4.08	10,391 - 62,844
3 (Arable and Horticultural)	22,944 (773)	2012	0 (0)	-	-	0.85 (486)	1.04 - 4.05	50,632 - 196,763
4 (Improved grassland)	20,573 (676)	2008	0 (0)	-	-	0.87 (560)	0.88 - 3.86	49,318 - 216,287
5 (Rough grassland)	748 (56)	1995	15 (15)	1988	1.48 - 4.42	0.86 (54)	0.46 - 1.94	2,505 - 10,500
6 (Neutral grassland)	3 (1)	1997	1 (1)	1988	0.73 - 4.42	0.98 (1)	0.15 - 1.19	15.13 - 119.4
7 (Calcareous grassland)	13 (2)	2010	0 (0)	-	-	0.88 (2)	1.21 - 4.42	241.1 - 884
8 (Acid grassland)	3,426 (206)	2002	0 (0)	-	-	0.93 (211)	0.8 - 4.32	16,945 - 91,141
9 (Fen, Marsh, and Swamp)	0 (0)	-	0 (0)	-	-	-	-	0
10 (Heather)	879 (54)	2006	1 (1)	1988	0.34 - 4.42	0.93 (54)	0.64 - 3.78	3,467 - 20,428
11 (Heather grassland)	3,090 (138)	2008	10 (10)	1988	1.18 - 4.42	0.92 (141)	0.56 - 2.93	7,848 - 41,307
12 (Bog)	1,612 (144)	2000	16 (16)	1988	0.51 - 4.42	0.91 (147)	0.7 - 3.31	10,250 - 48,596
13 (Montane habitat)	579 (52)	2002	0 (0)	-	-	0.98 (53)	0.54 - 4.42	2,883 - 23,427
14 (Inland rock)	19 (1)	2008	0 (0)	-	-	0.95 (1)	0.71 - 4.41	70.95 - 441.3
15 (Saltwater)	159 (5)	2012	0 (0)	-	-	0.66 (0)	-	0
16 (Freshwater)	68 (3)	2014	0 (0)	-	-	0.94 (3)	0.64 - 3.6	190.6 - 1,080
17 (Supra-littoral rock)	0 (0)	-	0 (0)	-	-	0.52 (0)	-	0
18 (Supra-littoral sediment)	27 (6)	2008	0 (0)	-	-	0.89 (5)	0.08 - 0.37	42.2 - 187.2
19 (Littoral rock)	10 (4)	1991	0 (0)	-	-	0.6 (0)	-	0
20 (Littoral sediment)	265 (20)	2006	0 (0)	-	-	0.74 (0)	-	0
21 (Saltmarsh)	0 (0)	-	0 (0)	-	-	-	-	0
22 (Urban)	31 (2)	2013	0 (0)	-	-	0.39 (0)	-	0
23 (Suburban)	911 (42)	2012	0 (0)	-	-	0.63 (3)	1.22 - 4.42	367 - 1,326
Total	58,869 (2,345)	2009	43 (43)	1988	1 - 4.42	0.87 (1,877)	0.83 - 3.82	155,399 - 716,216

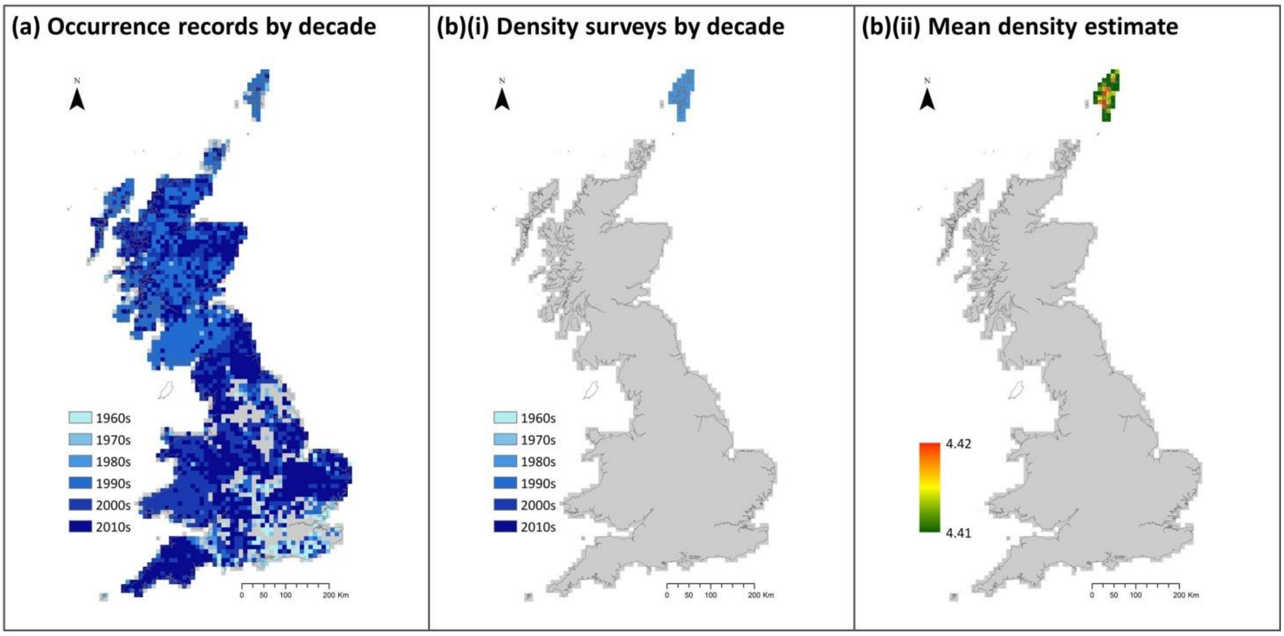

© Crown copyright and database rights 2016 Ordnance Survey 100051110. Data courtesy of the NBN Gateway with thanks to all data contributors. The NBN and its data contributors bear no responsibility for the further analysis or interpretation of this material, data and/or information.

Figure 1: 10km resolution raster maps based on BNG presenting the geographic description of available data. (a) shows the distribution of species occurrence obtained via the NBN Gateway categorised by the decade of last sighting. (b) shows information relating to density surveys identified via a search of published literature where: (i) categorises surveys by the decade of last survey; and (ii) shows the mean density estimate of surveys within grid cells (estimates assumed to be representative of entire cell, considered the upper limit of observed density).

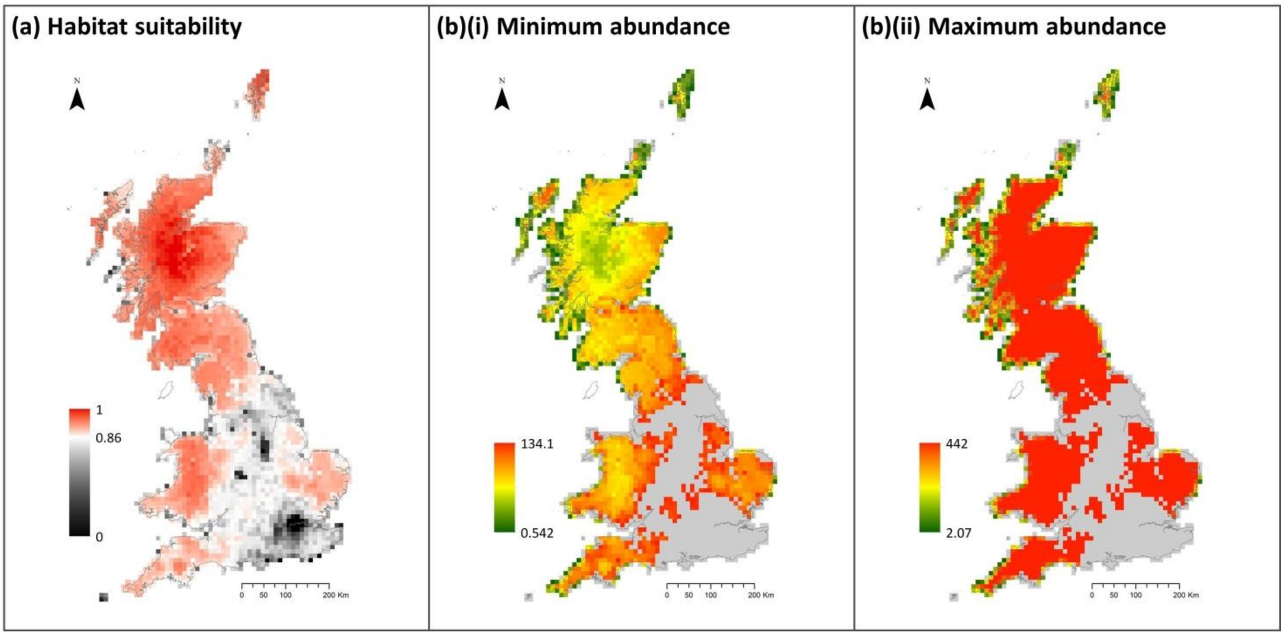

© Crown copyright and database rights 2016 Ordnance Survey 100051110. Data courtesy of the NBN Gateway with thanks to all data contributors. The NBN and its data contributors bear no responsibility for the further analysis or interpretation of this material, data and/or information.

Figure 2: Modelling predictions generated using systematic approach based on available data. (a) shows habitat suitability scores (the likelihood of observing the target species within each grid cell given variation environmental variables) determined by aggregating outputs from the "best" species distribution model (7 models compared) across 100 simulations. Here, the mid value on the scale denotes the threshold score above which occurrence is assumed. (b) shows: (i) the lower bound (Minimum); and (ii) the upper bound (Maximum); of abundance estimates determined by relating observed density (taking into account potential uncertainty) with habitat suitability scores using linear regression.
