# Supplementary material for: A systematic approach to estimate the distribution and total abundance of British mammals
Source: PLoS One. 2017 Jun 28;12(6):e0176339. doi: 10.1371/journal.pone.0176339 (PMC5489149; doi:10.1371/journal.pone.0176339)
Supplement: S4 File — Individual reports for each of the Carnivora species presenting analysis of the available data and subsequent model predictions based on a 10km raster grid. Reports also include expert comment assessing the reliability (and plausibility) of results in the context of existing evidence and popular opinion. (ZIP) [file pone.0176339.s004.zip › F Pine marten.pdf]

Pine marten (*Martes martes*)

Order: *Carnivora* **Genus:** *Martes*

Origin: Native **Status:** Locally common

1995 abundance estimate: 3,650 (2) **Reported population trends:** None

Data:

The available occurrence records indicate that pine marten are most commonly observed in northern Scotland with localised populations scattered throughout northern regions of England, particularly Cumbria, and Wales (Figure 1a). These sightings were reported in various habitats (predominantly arable and improved grassland) the majority of cells reporting at least one record since 1995. However, the maps suggests that more southerly records, particularly those in Wales, are less recent.

From the literature review we identified two studies (Bright & Halliwell 1999; Halliwell 1997) conducted in Scotland which reported densities between 1989 and 1998 (Figure 1b). Estimates ranged between 0.18 and 0.6 per km^2 with the highest densities recorded in montane habitat (0.12 - 0.6 per km^2 accounting for uncertainty relating to unsurveyed areas within grid cells). Unfortunately, due to the limited geographic range of these studies, estimates were unavailable for some habitats where occurrence was observed (marked grey in Table 1).

Model predictions:

The habitat suitability map (Figure 2a) appears to reflect the underlying data reasonably well with the set of "best" models predicting presence (and absence) to a mean AUC of 0.70. Overall, across 100 repetitions MaxEnt proved to be the most commonly selected modelling approach displaying the highest AUC 34% of the time followed by Random Forest (24%). By land cover the mean habitat suitability scores suggest observation is most likely in landscapes dominated by coniferous woodland. Consistent with this and recorded sightings, the majority of occurrence is predicted in coniferous woodland, acid grassland and improved grassland.

Linear regression suggested that there was no correlation between the estimates of maximum density and habitat suitability; consequently, it was applied as a fixed constant in cells where occurrence is predicted accounting for spherical spatial autocorrelation (which was determined to provide the best fit model). However, minimum density was found to be correlated with the best fit model relating habitat suitability linearly but without any correction for spatial autocorrelation. This result could be interpreted as an indication that assumptions associated with the minimum density are more appropriate for this species and consequently that the true estimate lies towards the lower end of the predicted range.

The predicted abundance range contains the estimate from Harris et al. (1995) suggesting no change in the total population.

Reliability (Expert comment):

The pine marten habitat suitability map reproduces the locations of known populations. The total population size should have increased since 1995, although recovery in this species appears to be slow. The true population is likely to still be in the lower quartile of the suggested range. The overall high value suggested by the model is probably due to the density estimate being made in its core habitat, and there being no density estimates available in the margins of its range, where it will be less common.

References:

Bright, P. W. and E. C. Halliwell (1999). Species recovery programme for the pine marten in England: 1996-1998. English Nature.

Halliwell, E. C. (1997). The ecology of red squirrels in Scotland in relation to pine marten predation. Ph.D. Thesis, University of Aberdeen.

Harris, S. J., P. Morris, S. Wray and D. Yalden (1995). A review of British mammals: population estimates and conservation status of British mammals other than cetaceans, Joint Nature Conservation Committee, Peterborough, UK.

Table 1: Summary of observed data and model predictions by land cover class (LCM2007 target classification). Values shown in brackets denote the spatial coverage based on a 10km resolution raster map (number of grid cells). Years represent the median of records within each land class. Ranges for density and abundance are derived using the respective minimum and maximum raster maps (lower bound is mean of values across minimum raster map with upper across the maximum) which capture the spatial uncertainty generate by projecting irregular polygons describing survey sites onto a raster grid.

LCM2007 class	Observed					Predicted		
	Occurrence		Density					
	Records	Year	Estimates	Year	Range	Habitat suitability	Density	Abundance
1 (Broadleaved woodland)	28 (2)	2008	0 (0)	-	-	0.28 (2)	0.07 - 0.39	13.11 - 77.73
2 (Coniferous woodland)	858 (87)	2011	25 (22)	1998	0.13 - 0.4	0.79 (124)	0.05 - 0.36	568.2 - 4,471
3 (Arable and Horticultural)	138 (51)	2006	1 (1)	1998	0.06 - 0.18	0.24 (51)	0.01 - 0.34	35.03 - 1,715
4 (Improved grassland)	446 (111)	2007	11 (5)	1998	0.11 - 0.3	0.41 (139)	0.01 - 0.36	195.3 - 4,988
5 (Rough grassland)	22 (9)	2007	0 (0)	-	-	0.34 (15)	0.01 - 0.3	11.84 - 453.5
6 (Neutral grassland)	0 (0)	-	0 (0)	-	-	0.09 (0)	-	0
7 (Calcareous grassland)	0 (0)	-	0 (0)	-	-	0.11 (0)	-	0
8 (Acid grassland)	387 (81)	2005	12 (12)	1998	0.07 - 0.38	0.72 (135)	0.03 - 0.38	387 - 5,120
9 (Fen, Marsh, and Swamp)	0 (0)	-	0 (0)	-	-	-	-	0
10 (Heather)	183 (36)	2012	12 (8)	1998	0.06 - 0.42	0.79 (43)	0.07 - 0.38	320.3 - 1,644
11 (Heather grassland)	655 (59)	2012	5 (5)	1989	0.07 - 0.42	0.63 (92)	0.04 - 0.31	328.92 - 2,849
12 (Bog)	55 (30)	2007	0 (0)	-	-	0.46 (53)	0.02 - 0.37	90.8 - 1,957
13 (Montane habitat)	183 (24)	2012	1 (1)	1998	0.12 - 0.6	0.78 (46)	0.01 - 0.39	59.88 - 1,788
14 (Inland rock)	0 (0)	-	0 (0)	-	-	0.32 (0)	-	0
15 (Saltwater)	23 (2)	2011	1 (1)	1998	0.14 - 0.53	0.35 (1)	0.09 - 0.36	8.67 - 35.89
16 (Freshwater)	1 (1)	2010	0 (0)	-	-	0.58 (2)	0 - 0.39	0 - 77.06
17 (Supra-littoral rock)	0 (0)	-	0 (0)	-	-	0.1 (0)	-	0
18 (Supra-littoral sediment)	0 (0)	-	0 (0)	-	-	0.22 (0)	-	0
19 (Littoral rock)	0 (0)	-	0 (0)	-	-	0.18 (0)	-	0
20 (Littoral sediment)	8 (1)	2009	0 (0)	-	-	0.26 (0)	-	0
21 (Saltmarsh)	0 (0)	-	0 (0)	-	-	-	-	0
22 (Urban)	0 (0)	-	0 (0)	-	-	0.12 (0)	-	0
23 (Suburban)	3 (1)	2014	0 (0)	-	-	0.15 (0)	-	0
Total	2,990 (495)	2009	68 (55)	1998	0.1 - 0.39	0.41 (703)	0.03 - 0.36	2,019 - 25,177

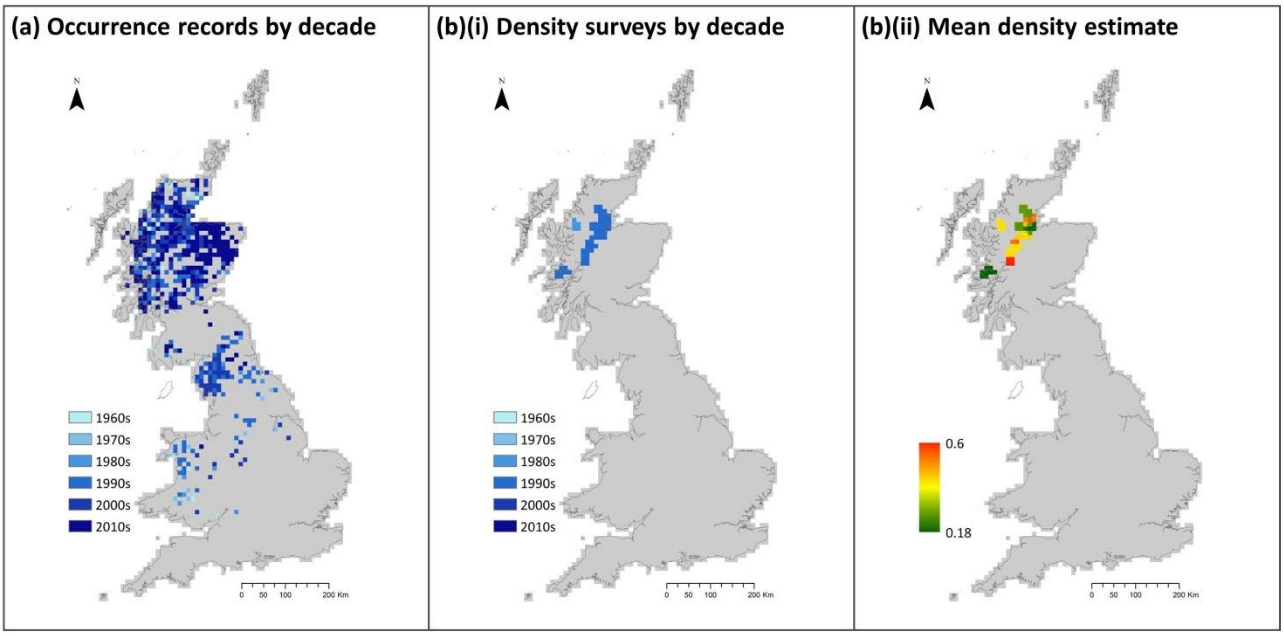

© Crown copyright and database rights 2016 Ordnance Survey 100051110. Data courtesy of the NBN Gateway with thanks to all data contributors. The NBN and its data contributors bear no responsibility for the further analysis or interpretation of this material, data and/or information.

Figure 1: 10km resolution raster maps based on BNG presenting the geographic description of available data. (a) shows the distribution of species occurrence obtained via the NBN Gateway categorised by the decade of last sighting. (b) shows information relating to density surveys identified via a search of published literature where: (i) categorises surveys by the decade of last survey; and (ii) shows the mean density estimate of surveys within grid cells (estimates assumed to be representative of entire cell, considered the upper limit of observed density).

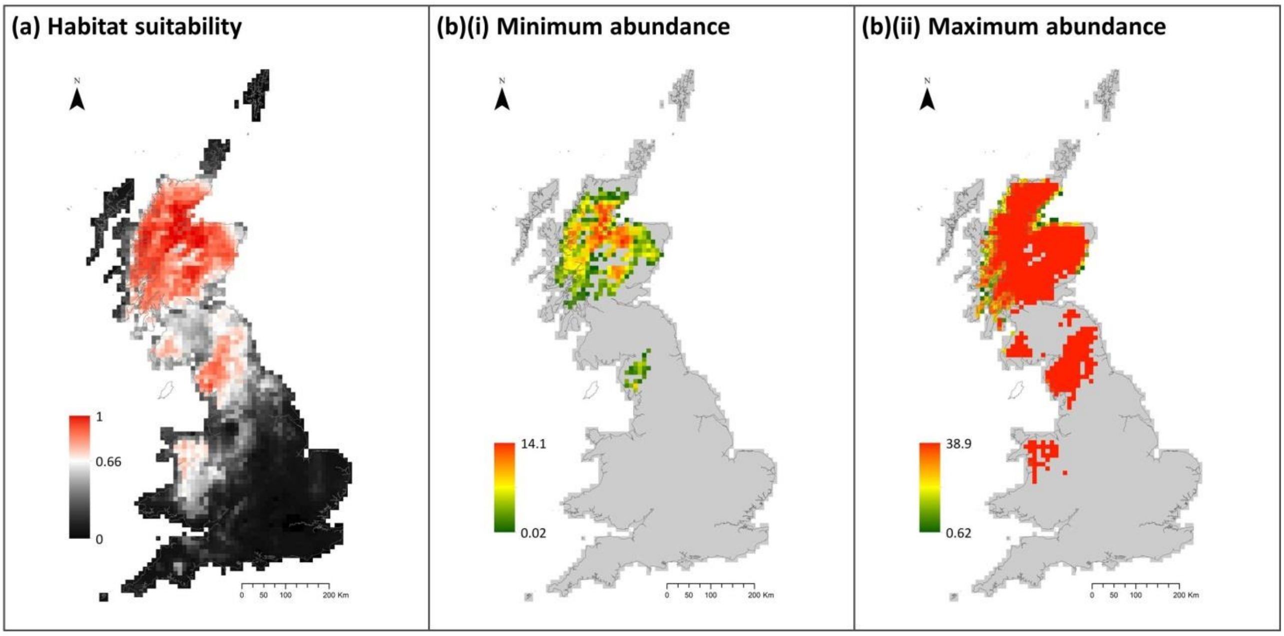

© Crown copyright and database rights 2016 Ordnance Survey 100051110. Data courtesy of the NBN Gateway with thanks to all data contributors. The NBN and its data contributors bear no responsibility for the further analysis or interpretation of this material, data and/or information.

Figure 2: Modelling predictions generated using systematic approach based on available data. (a) shows habitat suitability scores (the likelihood of observing the target species within each grid cell given variation environmental variables) determined by aggregating outputs from the "best" species distribution model (7 models compared) across 100 simulations. Here, the mid value on the scale denotes the threshold score above which occurrence is assumed. (b) shows: (i) the lower bound (Minimum); and (ii) the upper bound (Maximum); of abundance estimates determined by relating observed density (taking into account potential uncertainty) with habitat suitability scores using linear regression.
