# Supplementary material for: A systematic approach to estimate the distribution and total abundance of British mammals
Source: PLoS One. 2017 Jun 28;12(6):e0176339. doi: 10.1371/journal.pone.0176339 (PMC5489149; doi:10.1371/journal.pone.0176339)
Supplement: S4 File — Individual reports for each of the Carnivora species presenting analysis of the available data and subsequent model predictions based on a 10km raster grid. Reports also include expert comment assessing the reliability (and plausibility) of results in the context of existing evidence and popular opinion. (ZIP) [file pone.0176339.s004.zip › G Polecat.pdf]

Polecat (*Mustela putorius*)

Order: *Carnivora*

Genus: *Mustela*

Origin: Native

Status: Locally common

1995 abundance estimate: 15,000 (3)

Reported population trends: JNCC 2005 (↑), NGC 2009 (↔)

Data:

The available occurrence records indicate that polecats are widely distributed across Wales, the south east of England and Cumbria with some more localised populations scattered through the rest of GB (Figure 1a). These sightings were reported in various habitats (predominantly arable and improved grassland) with the majority (of cells) where occurrence was observed containing at least one record since 1995. However, the map highlights several areas, particularly in Wales, were the records have not been updated for some time.

From the literature review we identified a single publication (Birks 1997) reporting estimates for 1996 across large regions of Wales and Cumbria (Figure 1b). Whilst this provided data for a substantial proportion of the observed distribution there was a lack of distinction between habitats and consequently most assumed similar narrow ranges of density. Estimates ranged between 0 and 1.86 with the highest densities recorded in habitats dominated by bog (0.58 - 1.44 per km^2 accounting for uncertainty relating to unsurveyed areas within grid cells). Due to the coverage of reported estimates density data was available in all dominant land covers with the exception of calcareous grassland.

Model predictions:

The habitat suitability map (Figure 2a) appears to reflect the underlying data well with the set of "best" models predicting presence (and absence) to a mean AUC of 0.73. Overall, across 100 repetitions Random Forest proved to be the most commonly selected modelling approach displaying the highest AUC 31% of the time closely followed by MaxEnt (30%). By land cover the mean habitat suitability scores suggest observation is most likely in landscapes dominated by calcareous grassland (despite few observations) but, consistent with recorded sightings, the majority of occurrence is predicted in arable and improved grassland (the most common dominant land covers at a 10km scale).

Neither minimum nor maximum density estimates showed a correlation with habitat suitability. Both were best fitted using density as a fixed constant in cells where occurrence was predicted with accounting for spherical spatial autocorrelation. This is most likely a consequence of the lack of variation across habitats offered by the available density data.

The predicted abundance range does not contain the Harris et al. (1995) estimate; instead suggesting a significant increase in total population. Although this is in agreement with some reported trends the magnitude of the overestimation is again most likely due to a lack of variation in density across habitats. Whilst the estimates could be considered reasonable for the geographic areas over which they are reported the broad application mean that any association with habitat is lost and consequently higher densities could be incorrectly applied to common habitat compounding an overestimation resulting from the over-inflation of predicted range.

Reliability (Expert comment):

The polecat population has been recovering from past persecution. A single density estimate from close to 1995 suggests the predicted estimate should be similar to the 1995 estimate, although the additional spread since 1995 would inflate this. A recent survey that looked at verifiable true polecat records indicates a wider distribution than suggested by the NBN records. The overall uncertainty is clearly too narrow, but this was due to the large areas over which density was estimated and therefore no variance was introduced. Some of the NBN sighting data could be feral ferrets and thus the distribution could be inflated, along with the population estimate. However, although the final value may by slightly high, it is reasonable.

References:

Birks, J. D. S. (1997). A volunteer-based system for sampling variations in the abundance of polecats (*Mustela putorius*). *Journal of Zoology* 243(4): 857-863.

Harris, S. J., P. Morris, S. Wray and D. Yalden (1995). A review of British mammals: population estimates and conservation status of British mammals other than cetaceans, Joint Nature Conservation Committee, Peterborough, UK.

Table 1: Summary of observed data and model predictions by land cover class (LCM2007 target classification). Values shown in brackets denote the spatial coverage based on a 10km resolution raster map (number of grid cells). Years represent the median of records within each land class. Ranges for density and abundance are derived using the respective minimum and maximum raster maps (lower bound is mean of values across minimum raster map with upper across the maximum) which capture the spatial uncertainty generate by projecting irregular polygons describing survey sites onto a raster grid.

LCM2007 class	Observed					Predicted		
	Occurrence		Density					
	Records	Year	Estimates	Year	Range	Habitat suitability	Density	Abundance
1 (Broadleaved woodland)	58 (3)	2009	2 (2)	1996	1.33 - 1.33	0.48 (2)	0.58 - 0.6	116.4 - 119.6
2 (Coniferous woodland)	67 (17)	1998	15 (11)	1996	0.8 - 1.21	0.32 (13)	0.58 - 0.6	753.8 - 775
3 (Arable and Horticultural)	2,160 (278)	2009	257 (193)	1996	0.4 - 0.46	0.54 (376)	0.57 - 0.59	21,519 - 22,124
4 (Improved grassland)	2,194 (290)	2001	310 (243)	1996	0.97 - 1.08	0.59 (414)	0.53 - 0.54	21,827 - 22,441
5 (Rough grassland)	34 (7)	1996	5 (5)	1996	0.98 - 1.35	0.23 (8)	0.37 - 0.38	298.9 - 307.4
6 (Neutral grassland)	0 (0)	-	0 (0)	-	-	0 (0)	-	0
7 (Calcareous grassland)	2 (1)	2005	0 (0)	-	-	0.77 (2)	0.58 - 0.6	116.4 - 119.6
8 (Acid grassland)	191 (50)	1999	77 (53)	1996	1.25 - 1.4	0.39 (72)	0.58 - 0.6	4,181 - 4,299
9 (Fen, Marsh, and Swamp)	0 (0)	-	0 (0)	-	-	-	-	0
10 (Heather)	0 (0)	-	0 (0)	-	-	0.18 (0)	-	0
11 (Heather grassland)	11 (4)	2000	5 (4)	1996	0.52 - 1.41	0.14 (4)	0.58 - 0.6	232.7 - 239.3
12 (Bog)	13 (10)	2010	9 (9)	1996	0.58 - 1.44	0.21 (20)	0.58 - 0.6	1,164 - 1,196
13 (Montane habitat)	8 (3)	1997	2 (2)	1996	1.22 - 1.33	0.15 (1)	0.58 - 0.6	58.18 - 59.81
14 (Inland rock)	0 (0)	-	0 (0)	-	-	0.03 (0)	-	0
15 (Saltwater)	1 (1)	2012	4 (4)	1996	0.96 - 1.34	0.54 (3)	0.04 - 0.04	10.73 - 11.03
16 (Freshwater)	0 (0)	-	0 (0)	-	-	0.18 (0)	-	0
17 (Supra-littoral rock)	0 (0)	-	0 (0)	-	-	0.04 (0)	-	0
18 (Supra-littoral sediment)	0 (0)	-	1 (1)	1996	0.86 - 0.86	0.27 (1)	0.04 - 0.04	4.2 - 4.32
19 (Littoral rock)	0 (0)	-	0 (0)	-	-	0.12 (0)	-	0
20 (Littoral sediment)	33 (8)	2000	19 (16)	1996	0.88 - 1.19	0.56 (19)	0.35 - 0.36	674 - 692.9
21 (Saltmarsh)	0 (0)	-	0 (0)	-	-	-	-	0
22 (Urban)	1 (1)	2006	1 (0)	1996	0	0.25 (0)	-	0
23 (Suburban)	21 (9)	2008	18 (8)	1996	0.23 - 0.25	0.41 (19)	0.56 - 0.57	1,056 - 1,085
Total	4,794 (682)	2006	725 (551)	1996	0.77 - 0.9	0.46 (954)	0.55 - 0.56	52,011 - 53,475

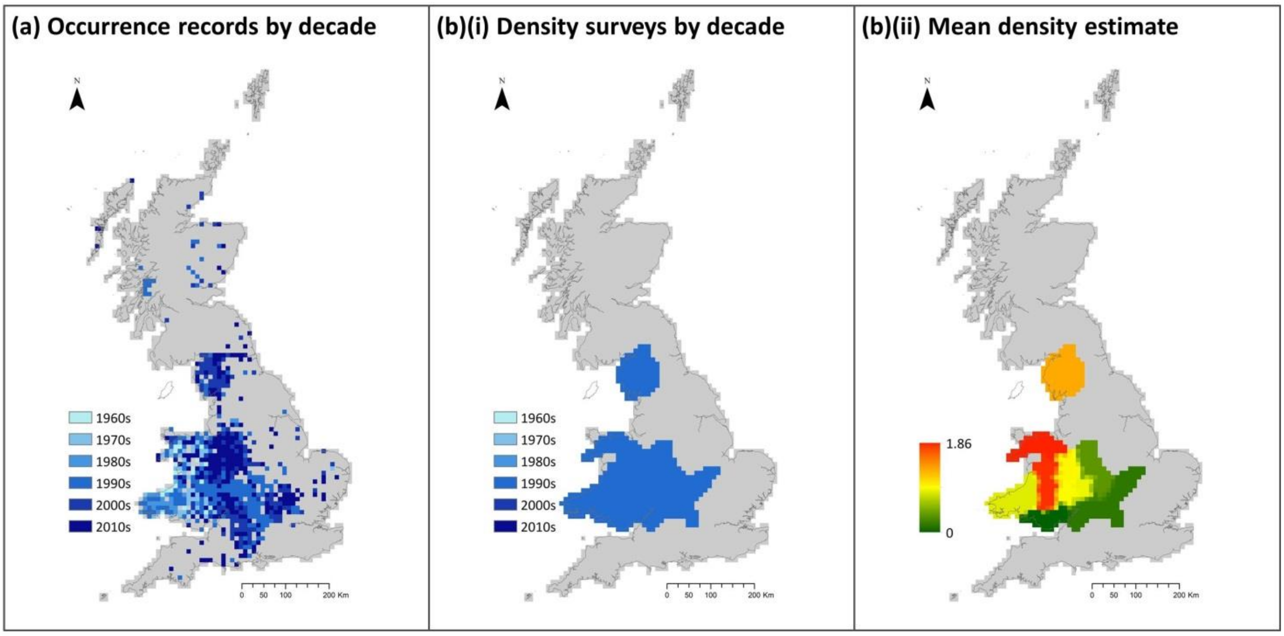

© Crown copyright and database rights 2016 Ordnance Survey 100051110. Data courtesy of the NBN Gateway with thanks to all data contributors. The NBN and its data contributors bear no responsibility for the further analysis or interpretation of this material, data and/or information.

Figure 1: 10km resolution raster maps based on BNG presenting the geographic description of available data. (a) shows the distribution of species occurrence obtained via the NBN Gateway categorised by the decade of last sighting. (b) shows information relating to density surveys identified via a search of published literature where: (i) categorises surveys by the decade of last survey; and (ii) shows the mean density estimate of surveys within grid cells (estimates assumed to be representative of entire cell, considered the upper limit of observed density).

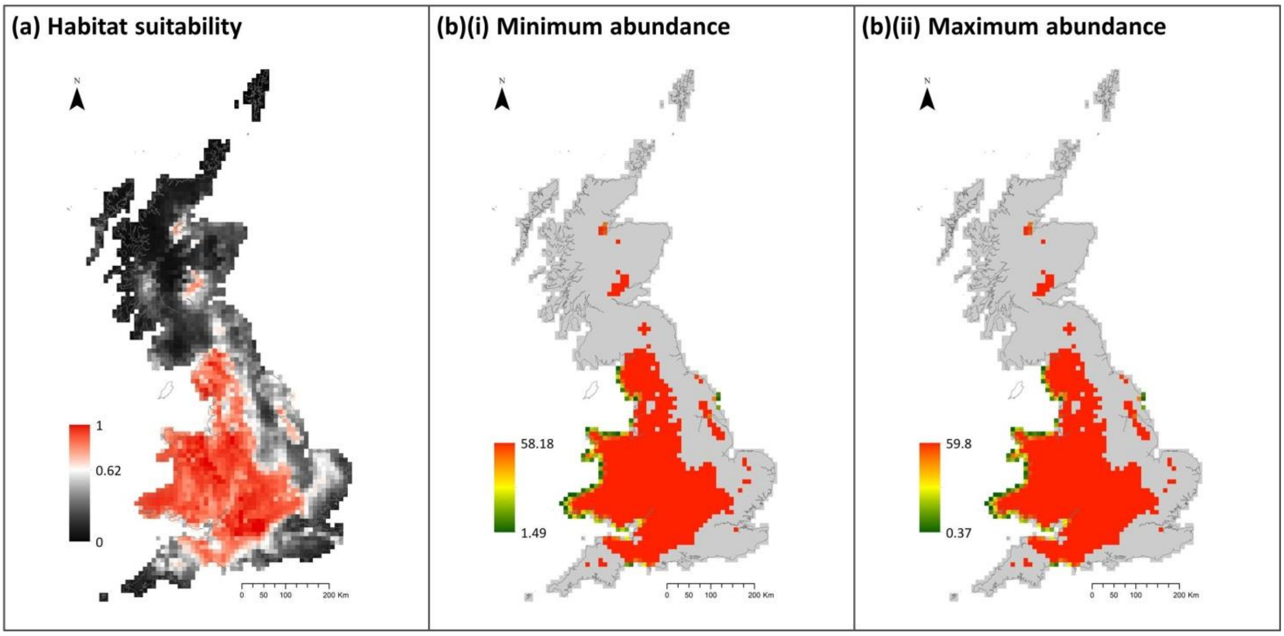

© Crown copyright and database rights 2016 Ordnance Survey 100051110. Data courtesy of the NBN Gateway with thanks to all data contributors. The NBN and its data contributors bear no responsibility for the further analysis or interpretation of this material, data and/or information.

Figure 2: Modelling predictions generated using systematic approach based on available data. (a) shows habitat suitability scores (the likelihood of observing the target species within each grid cell given variation environmental variables) determined by aggregating outputs from the "best" species distribution model (7 models compared) across 100 simulations. Here, the mid value on the scale denotes the threshold score above which occurrence is assumed. (b) shows: (i) the lower bound (Minimum); and (ii) the upper bound (Maximum); of abundance estimates determined by relating observed density (taking into account potential uncertainty) with habitat suitability scores using linear regression.
