# Supplementary material for: A systematic approach to estimate the distribution and total abundance of British mammals
Source: PLoS One. 2017 Jun 28;12(6):e0176339. doi: 10.1371/journal.pone.0176339 (PMC5489149; doi:10.1371/journal.pone.0176339)
Supplement: S4 File — Individual reports for each of the Carnivora species presenting analysis of the available data and subsequent model predictions based on a 10km raster grid. Reports also include expert comment assessing the reliability (and plausibility) of results in the context of existing evidence and popular opinion. (ZIP) [file pone.0176339.s004.zip › H Red fox.pdf]

Red fox (*Vulpes vulpes*)

Order: *Carnivora* **Genus:** *Vulpes*

Origin: Native **Status:** Common

1995 abundance estimate: 240,000 (4) **Reported population trends:** JNCC 2005 (↔), NGC 2009 (↑), BBS 2014 (↓)

Data:

The available occurrence records indicate that the red fox is widespread throughout GB with sightings reported in most 10 km squares (approximately 84%) at least once over the past decade (Figure 1a). However, the map highlights several areas, particularly in Scotland and Wales, where the species has not been recorded for some time or not at all.

Density estimates, primarily recorded over the past two decades, were obtained from published literature spanning approximately 10% of the observed species distribution based on the available occurrence data (Baker et al. 2004; Heydon et al. 2000; Lambin et al. 2000; Parrott et al. 2012). Geographically, these studies were concentrated in areas of south west England and Wales with one estimate in northern England and none in the south east or Scotland (Figure 1b). Estimates ranged between 0.18 and 27.6 per km^2 with the highest densities recorded in suburban habitat (0.77 - 15.58 per km^2 accounting for uncertainty relating to unsurveyed areas within grid cells). Despite the relatively high proportion of area surveyed estimates for several land cover classes were not available (land class marked grey in Table 1).

Model predictions:

The habitat suitability map (Figure 2a) appears to reflect the underlying data well with the set of “best” models predicting presence (and absence) to a mean AUC of 0.81. However, the distribution is slightly contracted towards the outer edges, particularly in the north west of Scotland where occurrence is observed. Overall, across 100 repetitions MaxEnt proved to be the most commonly selected modelling approach displaying the highest AUC 51% of the time followed by Random Forest (20%) and Generalised Linear Models (13%). By land cover the mean habitat suitability scores suggest observation is most likely in landscapes dominated by calcareous grassland, broadleaved woodland and arable land cover but, consistent with recorded sightings, the majority of occurrence is predicted in arable and improved grassland (the most common dominant land covers at a 10km scale).

Both minimum and maximum density estimates were best fitted to the square of habitat suitability accounting for spherical spatial autocorrelation. Interestingly, these relationships suggest a negative correlation in which squares with a higher likelihood of observation contain a lower density of individuals. This may be due to recording bias in these areas or the influence of other factors which are not fully captured by the modelling process.

The predicted abundance range contains the estimate from Harris et al. (1995) suggesting no significant change in the total population. Whilst this is not consistent with the latest reported trend there is scope within the range to suggest a general decrease is plausible. However, given the home range and broad use of habitat displayed by foxes it may be more reasonable to suggest that densities provided for partially surveyed squares are representative of the whole and therefore the true population estimate lies towards the top end of the predicted range.

Reliability (Expert comment):

For the red fox the most systematic assessment of its range was completed by Webbon et al. (2004), although they could not determine variation within a land class. Whilst there is a good similarity between these maps, the habitat suitability predicted here does not include some areas (e.g. Cumbria and south-west Scotland) where they are known to occur. This modelling also does not take account of localised fox control and so may be expected to overestimate the total population size. This approach (particular at a 10km scale) may be expected to miss any urban populations which can exist at substantially higher densities, albeit locally, and we know that urban populations have expanded in the number of locations, but not the local density, since 1995 (Scott et al. 2014). The true population is however likely to be in the upper half of the predicted range.

References:

Baker, P. J., S. M. Funk, M. W. Bruford and S. Harris (2004). Polygynandry in a red fox population: implications for the evolution of group living in canids? *Behavioral Ecology* 15(5): 766-778.

Harris, S. J., P. Morris, S. Wray and D. Yalden (1995). A review of British mammals: population estimates and conservation status of British mammals other than cetaceans, Joint Nature Conservation Committee, Peterborough, UK.

Heydon, M. J., J. C. Reynolds and M. J. Short (2000). Variation in abundance of foxes (*Vulpes vulpes*) between three regions of rural Britain, in relation to landscape and other variables. *Journal of Zoology* 251(2): 253-264.

Lambin, X., S. J. Petty and J. L. Mackinnon (2000). Cyclic dynamics in field vole populations and generalist predation. *Journal of Animal Ecology* 69(1): 106-119.

Parrott, D., A. Prickett, S. Pietravalle, T. R. Etherington and M. Fletcher (2012). Estimates of regional population densities of badger *Meles meles*, fox *Vulpes vulpes* and hare *Lepus europaeus* using walked distance sampling. *European Journal of Wildlife Research* 58(1): 23-33.

Scott, D. M., M. J. Berg, B. A. Tolhurst, A. L. M. Chauvenet, G. C. Smith, K. Neaves, J. Lochhead and P. J. Baker (2014). Changes in the distribution of red foxes (*Vulpes vulpes*) in urban areas in Great Britain: findings and limitations of a media-driven nationwide survey. PLoS ONE, 9, e99059.

Webbon, C.C., P. J. Baker and S. Harris (2004). Faecal density counts for monitoring changes in red fox numbers in rural Britain. Journal of Applied Ecology, 41, 768-779.

Table 1: Summary of observed data and model predictions by land cover class (LCM2007 target classification). Values shown in brackets denote the spatial coverage based on a 10km resolution raster map (number of grid cells). Years represent the median of records within each land class. Ranges for density and abundance are derived using the respective minimum and maximum raster maps (lower bound is mean of values across minimum raster map with upper across the maximum) which capture the spatial uncertainty generate by projecting irregular polygons describing survey sites onto a raster grid.

LCM2007 class	Observed					Predicted		
	Occurrence		Density					
	Records	Year	Estimates	Year	Range	Habitat suitability	Density	Abundance
1 (Broadleaved woodland)	717 (11)	2013	0 (0)	-	-	0.95 (11)	0.4 - 2.17	435.5 - 2,387
2 (Coniferous woodland)	1,213 (144)	2006	9 (10)	1998	0.86 - 3.84	0.9 (75)	0.4 - 2.19	2,969 - 16,389
3 (Arable and Horticultural)	25,478 (934)	2013	157 (151)	2006	0.4 - 2.25	0.95 (938)	0.35 - 1.89	33,024 - 177,346
4 (Improved grassland)	15,559 (687)	2012	125 (118)	2006	0.47 - 2.54	0.9 (619)	0.39 - 2.18	24,059 - 134,884
5 (Rough grassland)	141 (21)	2003	0 (0)	-	-	0.42 (0)	-	-
6 (Neutral grassland)	0 (0)	-	0 (0)	-	-	0 (0)	-	-
7 (Calcareous grassland)	66 (2)	2008	0 (0)	-	-	0.97 (2)	0.32 - 1.51	63.02 - 302.9
8 (Acid grassland)	1,129 (170)	2002	12 (11)	2006	0.14 - 1.53	0.86 (69)	0.51 - 3.07	3,507 - 21,176
9 (Fen, Marsh, and Swamp)	0 (0)	-	0 (0)	-	-	-	-	-
10 (Heather)	188 (45)	2007	0 (0)	-	-	0.84 (22)	0.54 - 3.34	1,185 - 7,344
11 (Heather grassland)	760 (83)	2006	0 (0)	-	-	0.66 (15)	0.56 - 3.46	835.5 - 5,195
12 (Bog)	331 (72)	2003	2 (2)	2006	0.12 - 2.51	0.56 (9)	0.48 - 2.82	429.1 - 2,536
13 (Montane habitat)	140 (39)	1998	0 (0)	-	-	0.84 (2)	0.62 - 3.92	123 - 783.3
14 (Inland rock)	4 (1)	2002	0 (0)	-	-	0.65 (0)	-	-
15 (Saltwater)	75 (8)	2011	0 (0)	-	-	0.84 (1)	0.28 - 1.83	28.48 - 182.9
16 (Freshwater)	4 (2)	1994	0 (0)	-	-	0.69 (0)	-	-
17 (Supra-littoral rock)	0 (0)	-	0 (0)	-	-	0.08 (0)	-	-
18 (Supra-littoral sediment)	30 (3)	2011	1 (1)	2006	2.7 - 3.55	0.64 (0)	-	-
19 (Littoral rock)	2 (1)	2006	0 (0)	-	-	0.43 (0)	-	-
20 (Littoral sediment)	453 (27)	2012	2 (2)	2006	0.07 - 0.98	0.84 (4)	0.46 - 2.81	183.4 - 1,122
21 (Saltmarsh)	0 (0)	-	0 (0)	-	-	-	-	-
22 (Urban)	830 (8)	2014	0 (0)	-	-	0.93 (7)	0.3 - 1.71	213.2 - 1,198
23 (Suburban)	9,431 (76)	2014	2 (2)	2004	0.77 - 15.58	0.93 (67)	0.38 - 2.22	2,570 - 14,863
Total	56,551 (2,334)	2011	310 (297)	2006	0.44 - 2.48	0.86 (1,841)	0.38 - 2.1	69,626 - 385,710

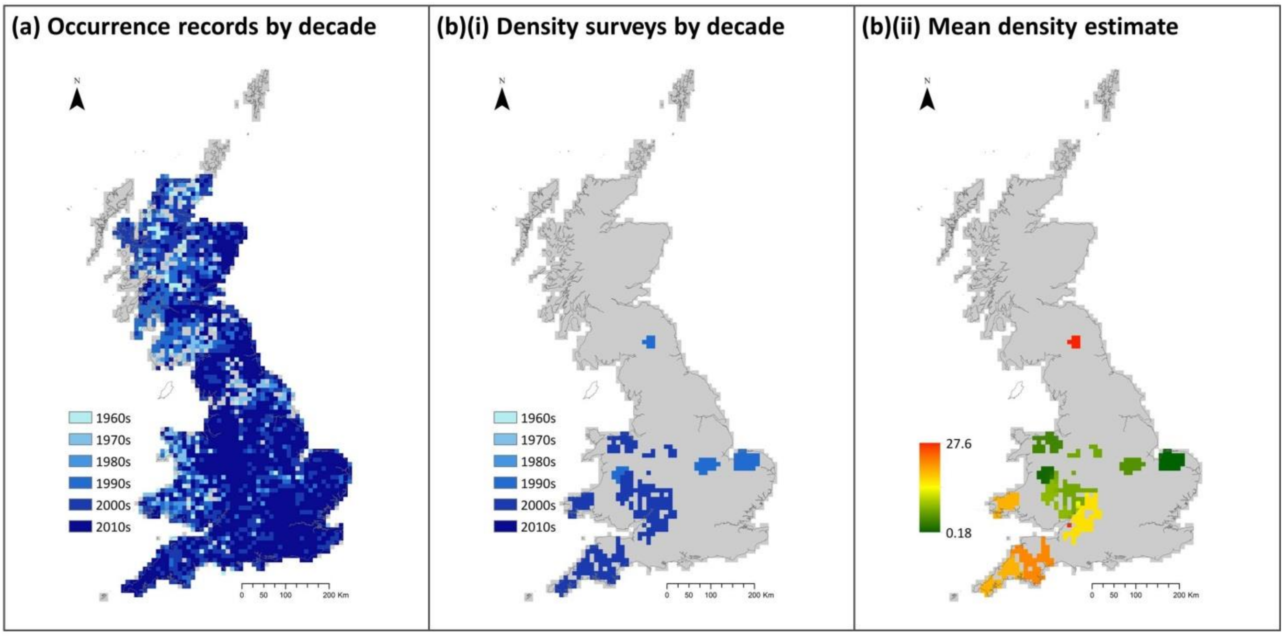

© Crown copyright and database rights 2016 Ordnance Survey 100051110. Data courtesy of the NBN Gateway with thanks to all data contributors. The NBN and its data contributors bear no responsibility for the further analysis or interpretation of this material, data and/or information.

Figure 1: 10km resolution raster maps based on BNG presenting the geographic description of available data. (a) shows the distribution of species occurrence obtained via the NBN Gateway categorised by the decade of last sighting. (b) shows information relating to density surveys identified via a search of published literature where: (i) categorises surveys by the decade of last survey; and (ii) shows the mean density estimate of surveys within grid cells (estimates assumed to be representative of entire cell, considered the upper limit of observed density).

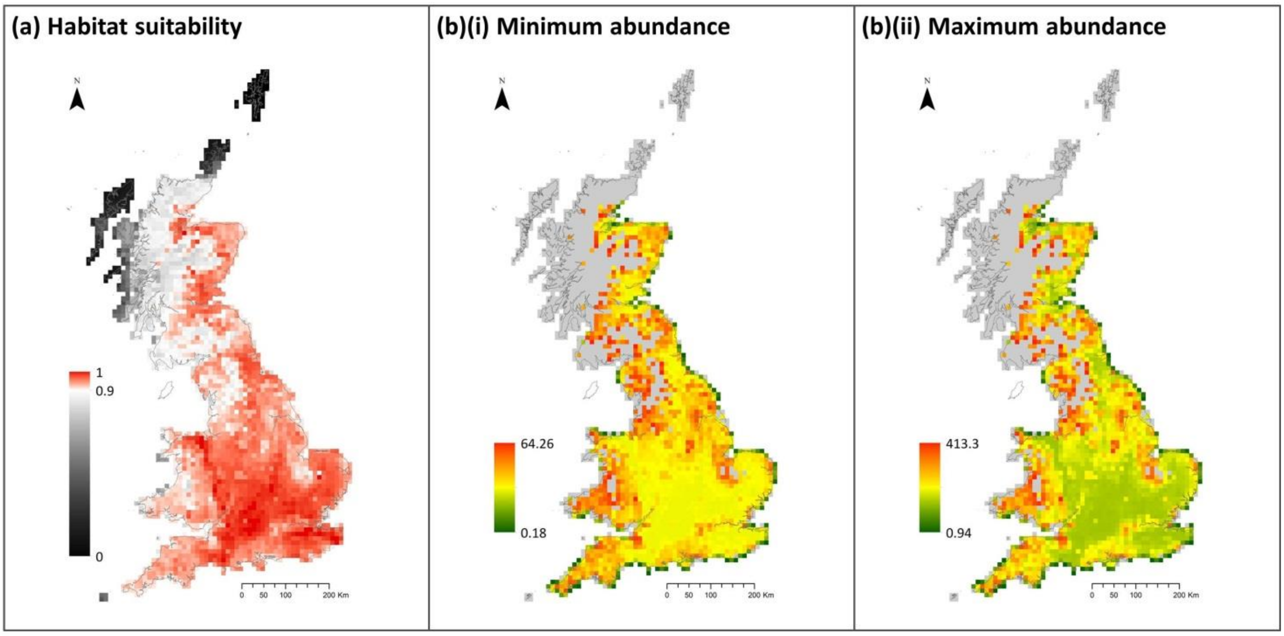

© Crown copyright and database rights 2016 Ordnance Survey 100051110. Data courtesy of the NBN Gateway with thanks to all data contributors. The NBN and its data contributors bear no responsibility for the further analysis or interpretation of this material, data and/or information.

Figure 2: Modelling predictions generated using systematic approach based on available data. (a) shows habitat suitability scores (the likelihood of observing the target species within each grid cell given variation environmental variables) determined by aggregating outputs from the "best" species distribution model (7 models compared) across 100 simulations. Here, the mid value on the scale denotes the threshold score above which occurrence is assumed. (b) shows: (i) the lower bound (Minimum); and (ii) the upper bound (Maximum); of abundance estimates determined by relating observed density (taking into account potential uncertainty) with habitat suitability scores using linear regression.
