# Supplementary material for: A systematic approach to estimate the distribution and total abundance of British mammals
Source: PLoS One. 2017 Jun 28;12(6):e0176339. doi: 10.1371/journal.pone.0176339 (PMC5489149; doi:10.1371/journal.pone.0176339)
Supplement: S4 File — Individual reports for each of the Carnivora species presenting analysis of the available data and subsequent model predictions based on a 10km raster grid. Reports also include expert comment assessing the reliability (and plausibility) of results in the context of existing evidence and popular opinion. (ZIP) [file pone.0176339.s004.zip › I Stoat.pdf]

Stoat (*Mustela erminea*)

Order: *Carnivora*

Genus: *Mustela*

Origin: Native

Status: Common

1995 abundance estimate: 462,000 (4)

Reported population trends: JNCC 2005 (↔), NGC 2009 (↑)

Data:

The available occurrence records indicate that the stoat is widespread throughout GB with sightings reported at least once in approximately 72% of 10km cells (Figure 1a). However, the map highlights some areas, predominantly in Scotland and Wales, where the species has never been recorded and other areas in England where it has not been recorded for some time.

From the literature review we were unable to identify any publications reporting an estimate of density.

Model predictions:

The habitat suitability map (Figure 2a) appears to reflect the underlying data well with the set of "best" models predicting presence (and absence) to a mean AUC of 0.74. Interestingly, London appears as an isolated patch of extremely unsuitable habitat within the landscape. Overall, across 100 repetitions Random Forest proved to be the most commonly selected modelling approach displaying the highest AUC 42% of the time closely followed by MaxEnt (38%). By land cover the mean habitat suitability scores suggest observation is most likely in landscapes dominated by calcareous grassland, arable and broadleaved woodland but, consistent with recorded sightings, the majority of occurrence is predicted in grid cells dominated by arable and improved grassland (the most common dominant land covers at a 10km scale). Occurrence is preserved in all other land covers where it is observed with the exception of inland and littoral rock dominated landscapes. The relative composition of habitats in the observed data is not reproduced.

Unfortunately, due to the lack of density estimates model analysis to predict abundance could not be performed.

Reliability (Expert comment):

The predicted range correctly identifies northern Scotland as habitat where the stoat is mostly absent, however, the occurrence data suggests areas of absence across Wales and Devon, with mostly only older records in Kent. While the model does predict Kent is suitable, it suggests areas of Wales and Devon are not very suitable. This is one species where further work on presence as well as density would be very helpful.

References:

None

Table 1: Summary of observed data and model predictions by land cover class (LCM2007 target classification). Values shown in brackets denote the spatial coverage based on a 10km resolution raster map (number of grid cells). Years represent the median of records within each land class. Ranges for density and abundance are derived using the respective minimum and maximum raster maps (lower bound is mean of values across minimum raster map with upper across the maximum) which capture the spatial uncertainty generate by projecting irregular polygons describing survey sites onto a raster grid.

LCM2007 class	Observed					Predicted		
	Occurrence		Density					
	Records	Year	Estimates	Year	Range	Habitat suitability	Density	Abundance
1 (Broadleaved woodland)	50 (9)	1985	0 (0)	-	-	0.92 (11)	-	-
2 (Coniferous woodland)	506 (99)	1996	0 (0)	-	-	0.79 (55)	-	-
3 (Arable and Horticultural)	11,020 (868)	2010	0 (0)	-	-	0.94 (949)	-	-
4 (Improved grassland)	4,055 (604)	2005	0 (0)	-	-	0.87 (600)	-	-
5 (Rough grassland)	153 (22)	1983	0 (0)	-	-	0.43 (9)	-	-
6 (Neutral grassland)	0 (0)	-	0 (0)	-	-	0 (0)	-	-
7 (Calcareous grassland)	13 (2)	2010	0 (0)	-	-	0.96 (2)	-	-
8 (Acid grassland)	446 (100)	2002	0 (0)	-	-	0.66 (47)	-	-
9 (Fen, Marsh, and Swamp)	0 (0)	-	0 (0)	-	-	-	-	-
10 (Heather)	193 (37)	2004	0 (0)	-	-	0.76 (26)	-	-
11 (Heather grassland)	564 (66)	2003	0 (0)	-	-	0.58 (26)	-	-
12 (Bog)	212 (62)	2002	0 (0)	-	-	0.52 (30)	-	-
13 (Montane habitat)	76 (20)	2004	0 (0)	-	-	0.54 (4)	-	-
14 (Inland rock)	1 (1)	2005	0 (0)	-	-	0.54 (0)	-	-
15 (Saltwater)	32 (7)	2006	0 (0)	-	-	0.78 (5)	-	-
16 (Freshwater)	8 (2)	2004	0 (0)	-	-	0.64 (1)	-	-
17 (Supra-littoral rock)	0 (0)	-	0 (0)	-	-	0 (0)	-	-
18 (Supra-littoral sediment)	22 (3)	2010	0 (0)	-	-	0.55 (2)	-	-
19 (Littoral rock)	2 (1)	2011	0 (0)	-	-	0.33 (0)	-	-
20 (Littoral sediment)	166 (26)	2006	0 (0)	-	-	0.82 (21)	-	-
21 (Saltmarsh)	0 (0)	-	0 (0)	-	-	-	-	-
22 (Urban)	24 (4)	2012	0 (0)	-	-	0.53 (1)	-	-
23 (Suburban)	289 (54)	2002	0 (0)	-	-	0.79 (33)	-	-
Total	17,832 (1,987)	2006	0 (0)	-	-	0.81 (1,822)	-	-

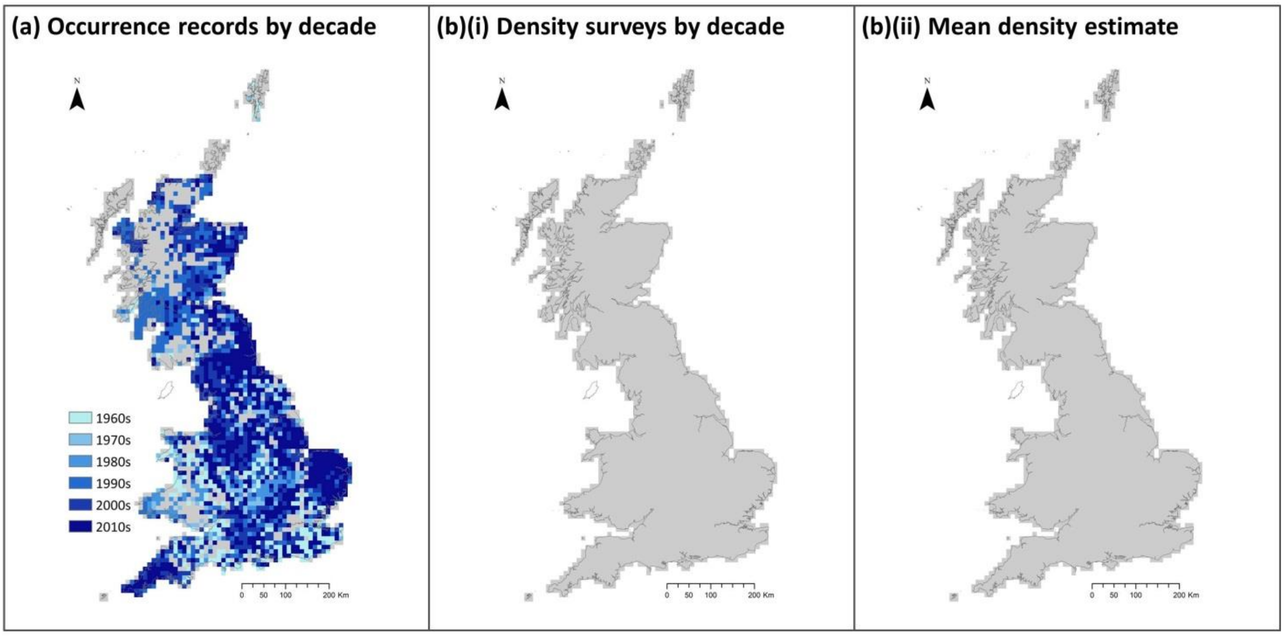

© Crown copyright and database rights 2016 Ordnance Survey 100051110. Data courtesy of the NBN Gateway with thanks to all data contributors. The NBN and its data contributors bear no responsibility for the further analysis or interpretation of this material, data and/or information.

Figure 1: 10km resolution raster maps based on BNG presenting the geographic description of available data. (a) shows the distribution of species occurrence obtained via the NBN Gateway categorised by the decade of last sighting. (b) shows information relating to density surveys identified via a search of published literature where: (i) categorises surveys by the decade of last survey; and (ii) shows the mean density estimate of surveys within grid cells (estimates assumed to be representative of entire cell, considered the upper limit of observed density).

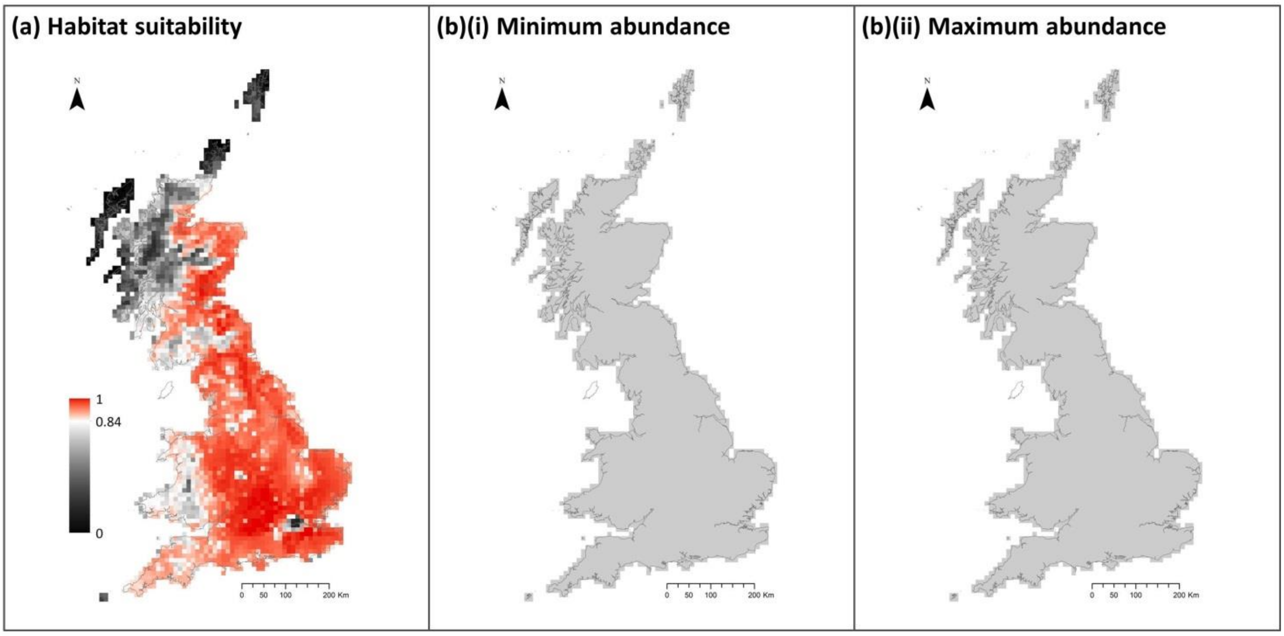

© Crown copyright and database rights 2016 Ordnance Survey 100051110. Data courtesy of the NBN Gateway with thanks to all data contributors. The NBN and its data contributors bear no responsibility for the further analysis or interpretation of this material, data and/or information.

Figure 2: Modelling predictions generated using systematic approach based on available data. (a) shows habitat suitability scores (the likelihood of observing the target species within each grid cell given variation environmental variables) determined by aggregating outputs from the "best" species distribution model (7 models compared) across 100 simulations. Here, the mid value on the scale denotes the threshold score above which occurrence is assumed. (b) shows: (i) the lower bound (Minimum); and (ii) the upper bound (Maximum); of abundance estimates determined by relating observed density (taking into account potential uncertainty) with habitat suitability scores using linear regression.
