# Supplementary material for: A systematic approach to estimate the distribution and total abundance of British mammals
Source: PLoS One. 2017 Jun 28;12(6):e0176339. doi: 10.1371/journal.pone.0176339 (PMC5489149; doi:10.1371/journal.pone.0176339)
Supplement: S4 File — Individual reports for each of the Carnivora species presenting analysis of the available data and subsequent model predictions based on a 10km raster grid. Reports also include expert comment assessing the reliability (and plausibility) of results in the context of existing evidence and popular opinion. (ZIP) [file pone.0176339.s004.zip › J Weasel.pdf]

Weasel (*Mustela nivalis*)

Order: *Carnivora*

Genus: *Mustela*

Origin: Native

Status: Common

1995 abundance estimate: 450,000 (4)

Reported population trends: JNCC 2005 (↔), NGC 2009 (↑)

Data:

The available occurrence records indicate that the weasel is widespread throughout GB (Figure 1a). However, the map highlights several areas, particularly in Wales, Scotland and the south east of England where the species has not been recorded for some time, or not at all.

Density estimates, recorded between 1973 and 2000, were obtained from published literature (Graham 2002; Harris et al. 1995; Moors 1975). Geographically, surveys were reasonably well spread across the distribution of observed occurrence (Figure 1b). Estimates ranged between 12.99 and 275 per km^2 with the highest densities recorded in habitat dominated by arable land cover (6.4 - 144 per km^2 accounting for uncertainty relating to unsurveyed areas within grid cells). Due to the limited coverage of the available surveys estimates were unavailable for a majority of dominant land covers where occurrence was reported (marked grey in Table 1).

Model predictions:

The habitat suitability map (Figure 2a) appears to reflect the underlying data reasonably well with the set of "best" models predicting presence (and absence) to a mean AUC of 0.76. Overall, across 100 repetitions MaxEnt proved to be the most commonly selected modelling approach displaying the highest AUC 47% of the time followed by Random Forest (27%) and Generalised Linear Models (21%). By land cover the mean habitat suitability scores suggest observation is most likely in landscapes dominated by arable and broadleaved woodland. Consistent with this and recorded sightings, the majority of occurrence is predicted in arable and improved grassland (the most common dominant land covers at a 10km scale).

Both minimum and maximum density estimates were best fitted linearly to habitat suitability accounting for spherical spatial autocorrelation.

Whilst the predicted abundance range does not contain the estimate from Harris et al. (1995) our prediction suggests a significant increase in total population consistent with the most recently reported trend (post 1995). This may explain the overestimation although perhaps the most likely cause is the use of high density estimates recorded across limited, potentially specialist, survey sites leading to inappropriate associations with more common dominant land covers at a 10km scale. It is possible that this could be resolved by applying models based on a finer scale raster grid where such habitats are better represented.

Reliability (Expert comment):

The predicted suitability map is very similar to that of the stoat. The weasel is harder to sight and the sparse records in Scotland and Wales may identify areas of non-suitable habitat or areas of low density (both weasel and human). The limited density estimates for the weasel, with one of these being rather old, appears to over-predict total abundance. Although there is limited evidence of a population increase, the original 1995 estimate was highly uncertain so it is possible that the true population is close to the lower estimate predicted here.

References:

Graham, I. M. (2002). Estimating weasel *Mustela nivalis* abundance from tunnel tracking indices at fluctuating field vole *Microtus agrestis* density. *Wildlife Biology* 8(4): 279-287.

Harris, S. J., P. Morris, S. Wray and D. Yalden (1995). A review of British mammals: population estimates and conservation status of British mammals other than cetaceans, Joint Nature Conservation Committee, Peterborough, UK.

Moors, P. J. (1975). The annual energy budget of a weasel (*Mustela nivalis*) population in farmland. Ph.D. Thesis, University of Aberdeen.

Table 1: Summary of observed data and model predictions by land cover class (LCM2007 target classification). Values shown in brackets denote the spatial coverage based on a 10km resolution raster map (number of grid cells). Years represent the median of records within each land class. Ranges for density and abundance are derived using the respective minimum and maximum raster maps (lower bound is mean of values across minimum raster map with upper across the maximum) which capture the spatial uncertainty generate by projecting irregular polygons describing survey sites onto a raster grid.

LCM2007 class	Observed					Predicted		
	Occurrence		Density					
	Records	Year	Estimates	Year	Range	Habitat suitability	Density	Abundance
1 (Broadleaved woodland)	34 (10)	1974	0 (0)	-	-	0.93 (11)	7.61 - 176.6	8,373 - 194,266
2 (Coniferous woodland)	303 (95)	1994	7 (7)	2000	3.99 - 75	0.77 (50)	3.15 - 86.44	15,751 - 432,189
3 (Arable and Horticultural)	8,418 (862)	2007	2 (2)	1977	6.4 - 144	0.93 (951)	7.5 - 171.8	713,079 - 16,339,584
4 (Improved grassland)	2,853 (583)	2001	0 (0)	-	-	0.84 (606)	4.57 - 113.3	277,007 - 6,866,027
5 (Rough grassland)	77 (17)	1993	0 (0)	-	-	0.37 (2)	3.88 - 100.1	776 - 20,013
6 (Neutral grassland)	0 (0)	-	0 (0)	-	-	0.01 (0)	-	-
7 (Calcareous grassland)	24 (2)	2014	0 (0)	-	-	0.93 (2)	7.47 - 173.8	1,494 - 34,764
8 (Acid grassland)	185 (77)	1996	0 (0)	-	-	0.6 (8)	0.21 - 17.28	165.5 - 13,820
9 (Fen, Marsh, and Swamp)	0 (0)	-	0 (0)	-	-	-	-	-
10 (Heather)	74 (28)	1996	0 (0)	-	-	0.68 (13)	0.55 - 28.79	713.9 - 37,428
11 (Heather grassland)	246 (49)	2003	0 (0)	-	-	0.51 (14)	0.38 - 26.16	531.6 - 36,624
12 (Bog)	139 (47)	2001	0 (0)	-	-	0.44 (18)	2.2 - 67.45	3,969 - 121,418
13 (Montane habitat)	47 (17)	1998	0 (0)	-	-	0.54 (2)	0 - 14.35	-
14 (Inland rock)	0 (0)	-	0 (0)	-	-	0.22 (0)	-	-
15 (Saltwater)	46 (7)	2007	0 (0)	-	-	0.79 (6)	1.14 - 35.12	685 - 21,071
16 (Freshwater)	7 (2)	1994	0 (0)	-	-	0.63 (1)	0 - 24.79	-
17 (Supra-littoral rock)	0 (0)	-	0 (0)	-	-	0.08 (0)	-	-
18 (Supra-littoral sediment)	20 (2)	1990	0 (0)	-	-	0.58 (2)	0.62 - 17.17	123.7 - 3,435
19 (Littoral rock)	2 (1)	2013	0 (0)	-	-	0.4 (3)	0.01 - 0.46	2.5 - 137.5
20 (Littoral sediment)	186 (27)	1998	0 (0)	-	-	0.84 (29)	2.21 - 58.06	6,408 - 168,380
21 (Saltmarsh)	0 (0)	-	0 (0)	-	-	-	-	-
22 (Urban)	94 (7)	2010	0 (0)	-	-	0.86 (8)	1.15 - 36.49	916.8 - 29,196
23 (Suburban)	564 (60)	2006	0 (0)	-	-	0.88 (70)	3.78 - 96.48	26,434 - 675,390
Total	13,319 (1,893)	2004	9 (9)	2000	4.52 - 90	0.78 (1,796)	5.88 - 139.2	1,056,431 - 24,999,091

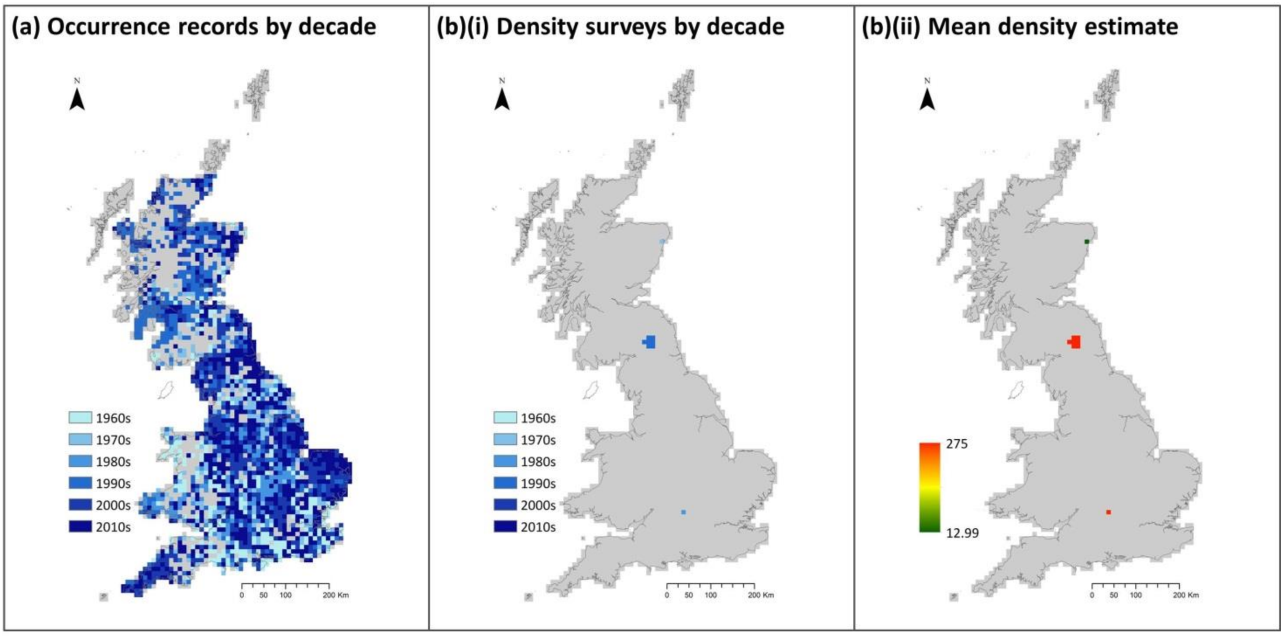

© Crown copyright and database rights 2016 Ordnance Survey 100051110. Data courtesy of the NBN Gateway with thanks to all data contributors. The NBN and its data contributors bear no responsibility for the further analysis or interpretation of this material, data and/or information.

Figure 1: 10km resolution raster maps based on BNG presenting the geographic description of available data. (a) shows the distribution of species occurrence obtained via the NBN Gateway categorised by the decade of last sighting. (b) shows information relating to density surveys identified via a search of published literature where: (i) categorises surveys by the decade of last survey; and (ii) shows the mean density estimate of surveys within grid cells (estimates assumed to be representative of entire cell, considered the upper limit of observed density).

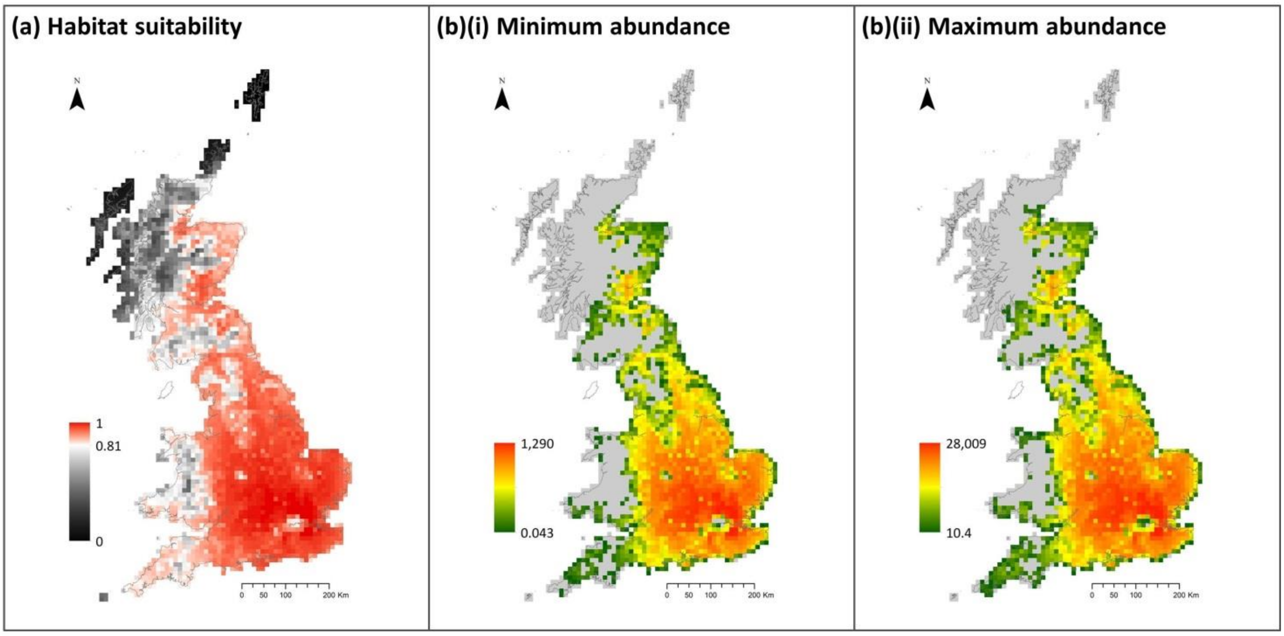

© Crown copyright and database rights 2016 Ordnance Survey 100051110. Data courtesy of the NBN Gateway with thanks to all data contributors. The NBN and its data contributors bear no responsibility for the further analysis or interpretation of this material, data and/or information.

Figure 2: Modelling predictions generated using systematic approach based on available data. (a) shows habitat suitability scores (the likelihood of observing the target species within each grid cell given variation environmental variables) determined by aggregating outputs from the "best" species distribution model (7 models compared) across 100 simulations. Here, the mid value on the scale denotes the threshold score above which occurrence is assumed. (b) shows: (i) the lower bound (Minimum); and (ii) the upper bound (Maximum); of abundance estimates determined by relating observed density (taking into account potential uncertainty) with habitat suitability scores using linear regression.
