# Supplementary material for: A systematic approach to estimate the distribution and total abundance of British mammals
Source: PLoS One. 2017 Jun 28;12(6):e0176339. doi: 10.1371/journal.pone.0176339 (PMC5489149; doi:10.1371/journal.pone.0176339)
Supplement: S4 File — Individual reports for each of the Carnivora species presenting analysis of the available data and subsequent model predictions based on a 10km raster grid. Reports also include expert comment assessing the reliability (and plausibility) of results in the context of existing evidence and popular opinion. (ZIP) [file pone.0176339.s004.zip › K Wildcat.pdf]

Wildcat (*Felis silvestris*)

Order: *Carnivora*

Genus: *Felis*

Origin: Native

Status: Rare

1995 abundance estimate: 3,500 (3)

Reported population trends: None

Data:

The available occurrence records indicate that the wildcat is limited to Scotland with the majority located across northern regions in habitat dominated by coniferous woodland (Figure 1a). Whilst some records are recent the map highlights several areas where the species has not been reported for some time.

From the literature review we identified two studies (Corbett 1979; Kilshaw et al. 2014) conducted in the north east of Scotland (Figure 1b) with one reporting densities from 1978 and the other from 2010. Estimates ranged between 0.3 and 0.68 per km^2 with the highest densities reported in coniferous woodland and heather grassland. Due to the limited coverage of these surveys estimates were unavailable for several dominant land covers where occurrence was reported (marked grey in Table 1).

Model predictions:

The habitat suitability map (Figure 2a) appears to reflect the underlying data well with the set of "best" models predicting presence (and absence) to a mean AUC of 0.73. Overall, across 100 repetitions MaxEnt proved to be the most commonly selected modelling approach displaying the highest AUC 31% of the time followed by Support Machine Vectors and Random Forest (18%). By land cover the mean habitat suitability scores suggest observation is most likely in landscapes dominated by heather but, consistent with recorded sightings, the majority of occurrence is predicted in coniferous woodland and heather grassland.

Neither minimum nor maximum density estimates showed a correlation with habitat suitability. Both were best fitted using density as a fixed constant in cells where occurrence was predicted with accounting for spherical spatial autocorrelation. This is most likely a consequence of the limited number of density estimates available.

Despite this the predicted abundance range contains the Harris et al. (1995) estimate suggesting no change in total population (this is unsurprising as both predictions use similar density studies; it perhaps indicates that any changes in distribution over the past 20 years are not significant). In order to produce more spatially informative results a greater number of density studies would be required. Given the rarity of the species it may also be advisable to consider analysis based on a finer scale raster grid in which meaningful associations with specialist habitat can be identified.

Reliability (Expert comment):

The wildcat in Britain (*Felis sylvestris sylvestris*) is limited to more remote parts of Scotland and without doubt many of the sightings here will be of hybrid animals since it can be impossible to differentiate between them by sight alone. Despite the potential for the sighting data to include many domesticated cats with little or no true wildcat genetic make-up, the model output is thankfully low. Given this likely over-reporting, the true population is likely to be closer to the lower end of the estimated range. However, given the difficulties of identifying this sub-species we cannot determine with any accuracy whether the population has changed since the 1995 was made.

References:

Corbett, L. K. (1979). Feeding ecology and social organization of wildcats. Ph.D. Thesis, University of Aberdeen.

Harris, S. J., P. Morris, S. Wray and D. Yalden (1995). A review of British mammals: population estimates and conservation status of British mammals other than cetaceans, Joint Nature Conservation Committee, Peterborough.

Kilshaw, K., P. J. Johnson, A. C. Kitchener and D. W. Macdonald (2014). Detecting the elusive Scottish wildcat *Felis silvestris* using camera trapping. *Oryx* 49(2): 207-215.

Table 1: Summary of observed data and model predictions by land cover class (LCM2007 target classification). Values shown in brackets denote the spatial coverage based on a 10km resolution raster map (number of grid cells). Years represent the median of records within each land class. Ranges for density and abundance are derived using the respective minimum and maximum raster maps (lower bound is mean of values across minimum raster map with upper across the maximum) which capture the spatial uncertainty generate by projecting irregular polygons describing survey sites onto a raster grid.

LCM2007 class	Observed					Predicted		
	Occurrence		Density					
	Records	Year	Estimates	Year	Range	Habitat suitability	Density	Abundance
1 (Broadleaved woodland)	0 (0)	-	0 (0)	-	-	0.09 (0)	-	-
2 (Coniferous woodland)	200 (58)	1994	3 (3)	2010	0.01 - 0.55	0.58 (78)	0.05 - 0.45	377.7 - 3,528
3 (Arable and Horticultural)	30 (13)	1995	0 (0)	-	-	0.1 (7)	0.05 - 0.49	36.54 - 341.3
4 (Improved grassland)	118 (33)	1993	0 (0)	-	-	0.16 (28)	0.04 - 0.41	121.5 - 1,135
5 (Rough grassland)	24 (6)	1996	0 (0)	-	-	0.22 (6)	0.04 - 0.36	22.94 - 214.3
6 (Neutral grassland)	0 (0)	-	0 (0)	-	-	0.09 (0)	-	-
7 (Calcareous grassland)	0 (0)	-	0 (0)	-	-	0.08 (0)	-	-
8 (Acid grassland)	103 (38)	1978	0 (0)	-	-	0.43 (68)	0.05 - 0.47	342.3 - 3,197
9 (Fen, Marsh, and Swamp)	0 (0)	-	0 (0)	-	-	-	-	-
10 (Heather)	103 (29)	1997	1 (1)	1978	0.24 - 0.3	0.67 (43)	0.05 - 0.47	217.2 - 2,029
11 (Heather grassland)	182 (47)	1998	3 (3)	2010	0.04 - 0.55	0.5 (61)	0.04 - 0.41	270.1 - 2,523
12 (Bog)	30 (20)	1985	0 (0)	-	-	0.34 (42)	0.05 - 0.46	206.2 - 1,926
13 (Montane habitat)	46 (18)	1973	2 (2)	1978	0.04 - 0.3	0.62 (26)	0.05 - 0.49	135.7 - 1,268
14 (Inland rock)	0 (0)	-	0 (0)	-	-	0.22 (0)	-	-
15 (Saltwater)	0 (0)	-	0 (0)	-	-	0.14 (1)	0.05 - 0.45	4.82 - 45.03
16 (Freshwater)	2 (2)	1970	0 (0)	-	-	0.53 (1)	0.05 - 0.49	5.22 - 48.75
17 (Supra-littoral rock)	0 (0)	-	0 (0)	-	-	0.1 (0)	-	-
18 (Supra-littoral sediment)	0 (0)	-	0 (0)	-	-	0.13 (0)	-	-
19 (Littoral rock)	0 (0)	-	0 (0)	-	-	0.12 (0)	-	-
20 (Littoral sediment)	0 (0)	-	0 (0)	-	-	0.07 (0)	-	-
21 (Saltmarsh)	0 (0)	-	0 (0)	-	-	-	-	-
22 (Urban)	0 (0)	-	0 (0)	-	-	0.06 (0)	-	-
23 (Suburban)	1 (1)	1960	0 (0)	-	-	0.08 (0)	-	-
Total	839 (265)	1994	9 (9)	1978	0.05 - 0.47	0.23 (361)	0.05 - 0.45	1,740 - 16,255

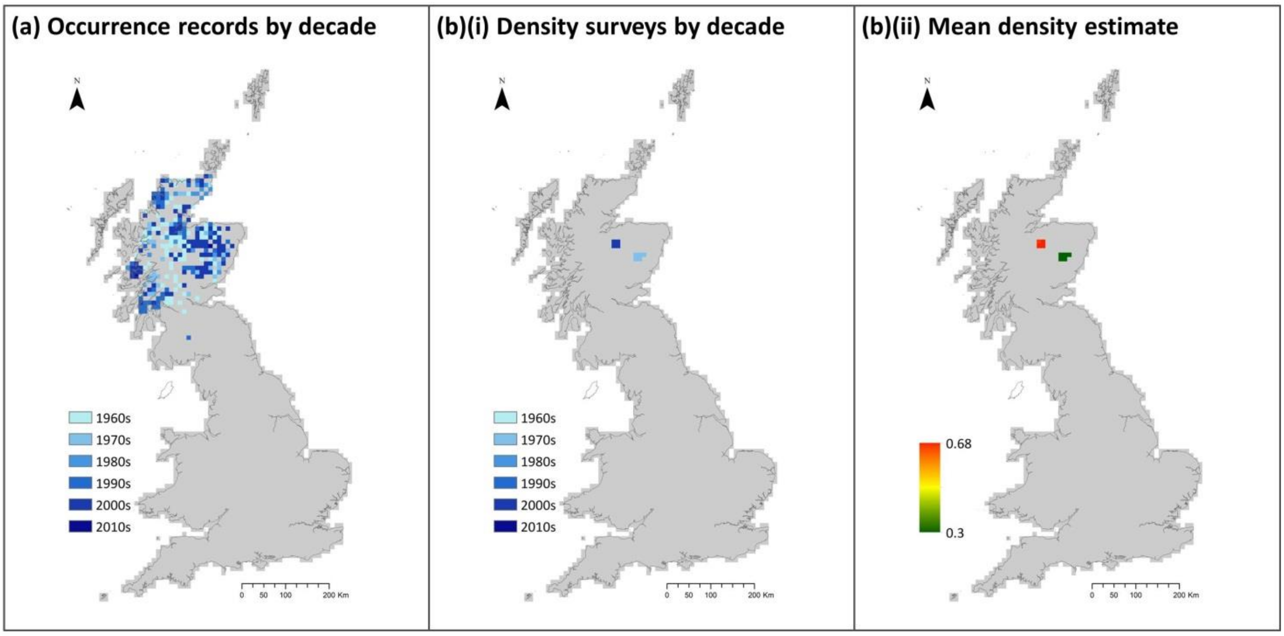

© Crown copyright and database rights 2016 Ordnance Survey 100051110. Data courtesy of the NBN Gateway with thanks to all data contributors. The NBN and its data contributors bear no responsibility for the further analysis or interpretation of this material, data and/or information.

Figure 1: 10km resolution raster maps based on BNG presenting the geographic description of available data. (a) shows the distribution of species occurrence obtained via the NBN Gateway categorised by the decade of last sighting. (b) shows information relating to density surveys identified via a search of published literature where: (i) categorises surveys by the decade of last survey; and (ii) shows the mean density estimate of surveys within grid cells (estimates assumed to be representative of entire cell, considered the upper limit of observed density).

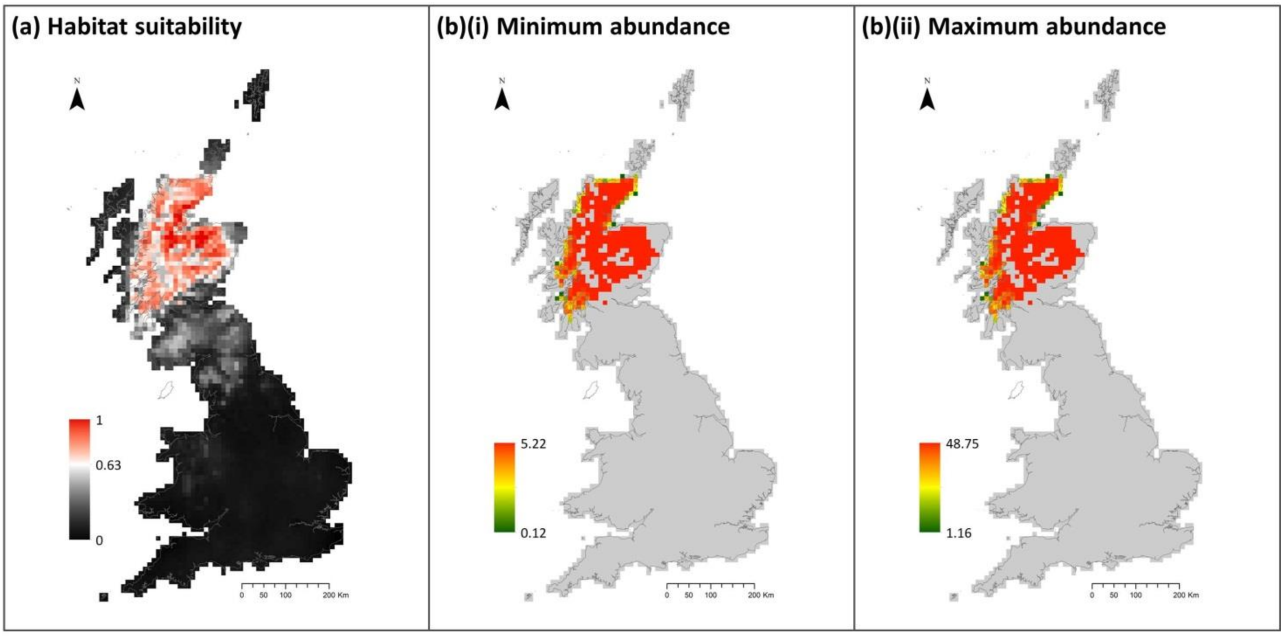

© Crown copyright and database rights 2016 Ordnance Survey 100051110. Data courtesy of the NBN Gateway with thanks to all data contributors. The NBN and its data contributors bear no responsibility for the further analysis or interpretation of this material, data and/or information.

Figure 2: Modelling predictions generated using systematic approach based on available data. (a) shows habitat suitability scores (the likelihood of observing the target species within each grid cell given variation environmental variables) determined by aggregating outputs from the "best" species distribution model (7 models compared) across 100 simulations. Here, the mid value on the scale denotes the threshold score above which occurrence is assumed. (b) shows: (i) the lower bound (Minimum); and (ii) the upper bound (Maximum); of abundance estimates determined by relating observed density (taking into account potential uncertainty) with habitat suitability scores using linear regression.
