# Supplementary material for: A systematic approach to estimate the distribution and total abundance of British mammals
Source: PLoS One. 2017 Jun 28;12(6):e0176339. doi: 10.1371/journal.pone.0176339 (PMC5489149; doi:10.1371/journal.pone.0176339)
Supplement: S5 File — Individual reports for each of the Chiroptera species presenting analysis of the available data and subsequent model predictions based on a 10km raster grid. Reports also include expert comment assessing the reliability (and plausibility) of results in the context of existing evidence and popular opinion. (ZIP) [file pone.0176339.s005.zip › B Bechsteins bat.pdf]

Bechstein's bat (*Myotis bechsteinii*)

Order: *Chiroptera*

Genus: *Myotis*

Origin: Native

Status: Very rare

1995 abundance estimate: 1,500 (2)

Reported population trends: None

Data:

The available occurrence records indicate that the distribution of Bechstein's bat is limited to southern England (Figure 1a). Observations were made in a variety of habitats (predominantly arable and improved grassland) with the majority recorded over the last decade.

From the literature review we identified a single survey conducted at Brackett's copse (Dorset) in 2011 which estimated density within this isolated patch of woodland to be approximately 329 per km^2 (Fairless 2013). At a 10km scale this study site is represented by a single grid cell dominated by improved grassland (Figure 1b). Due to the relative size of area surveyed the potential range of density accounting for uncertainty is very large (0.87 - 329 per km^2).

Model predictions:

The habitat suitability map (Figure 2a) appears to reflect the underlying data well with the set of "best" models predicting presence (and absence) to a mean AUC of 0.72. Overall, across 100 repetitions Generalised Linear Models proved to be the most commonly selected modelling approach displaying the highest AUC 32% of the time followed by BioClim (18%). By land cover the mean habitat suitability scores suggest observation is most likely in landscapes dominated by calcareous grassland (Table 1) but, consistent with recorded sightings, the majority of occurrence is predicted in grid cells dominated by arable and improved grassland. The analysis shows that occurrence is preserved in all land covers where it is observed with the exception of acid grassland dominated habitats.

Due to the limited number of density estimates it was not possible to assess any relationship with habitat suitability. Instead, a constant mean estimate was applied to all cells where occurrence was predicted and summed to derive total abundance.

The predicted abundance range does not contain the estimate from Harris et al. (1995) instead suggesting a significant increase in the total population (this result could be explained by an increase in density between 1995 and the median year of estimates, which is 2011, and/or a significant expansion of species range over the past 20 years). Additionally, the range is very large due to the uncertainty caused by small survey sites relative to the 10km scale at which modelling is performed.

Reliability (Expert comment):

The distribution of available occurrence records is not completely implausible but possibly does not account for the known behaviour of males; which can be found at some distance from breeding communities. Consequently, the core distribution of established populations within the landscape may be smaller than suggested by these observations. The density estimate obtained from the published literature is very high and is therefore not representative of a national average.

The distribution presented in the habitat suitability map is plausible given our current knowledge of the species. However, there is evidence to suggest that the species may also be present in other areas, such as Oxfordshire, which are not predicted. The habitat associations highlighted by the land cover analysis are reasonable given the location and known ecology of the species (typically found in broadleaved woodland).

Despite the confidence with which the 1995 estimate was reported (reliability score of 2 out of 5 indicating a good estimate) a value of 1,500 could be considered low. A mean abundance between 10,000 and 20,000, closer to the lower bound of the predicted range, would be more reasonable. Spatially, the maps of predicted abundance show a

lack of variability across the distribution (Figure 2b); applying a high density to most areas where occurrence is predicted. In reality, such high densities would be restricted to small localised patches within the landscape. The broad use of an estimate for a very high density site would likely explain the overestimation of predicted abundance.

In order to produce more meaningful model predictions in a spatial context additional density estimates are required. It may also be possible to improve model accuracy (narrowing the range of predictions) by performing model analysis based on a finer scale raster grid which would better represents the variation in habitat, especially important for a species such as Bechstein's bat which appears to uniquely demonstrate a specialist requirement for old deciduous woodland. Unfortunately, fine scale analysis using data from the NBN is too unreliable due to access restrictions imposed on available occurrence data.

References:

Fairless, L. (2013). Ecology and ecophysiology of social structure and population dynamics in bats (*Vespertilionidae*). Ph.D. Thesis, University of Southampton.

Harris, S. J., P. Morris, S. Wray and D. Yalden (1995). A review of British mammals: population estimates and conservation status of British mammals other than cetaceans, Joint Nature Conservation Committee, Peterborough, UK.

Table 1: Summary of observed data and model predictions by land cover class (LCM2007 target classification). Values shown in brackets denote the spatial coverage based on a 10km resolution raster map (number of grid cells). Years represent the median of records within each land class. Ranges for density and abundance are derived using the respective minimum and maximum raster maps (lower bound is mean of values across minimum raster map with upper across the maximum) which capture the spatial uncertainty generate by projecting irregular polygons describing survey sites onto a raster grid.

LCM2007 class	Observed					Predicted		
	Occurrence		Density					
	Records	Year	Estimates	Year	Range	Habitat suitability	Density	Abundance
1 (Broadleaved woodland)	2 (2)	1998	0 (0)	-	-	0.53 (6)	0.87 - 329	521.8 - 197,371
2 (Coniferous woodland)	108 (1)	2011	0 (0)	-	-	0.15 (1)	0.87 - 329	86.97 - 32,895
3 (Arable and Horticultural)	320 (47)	2010	0 (0)	-	-	0.32 (123)	0.83 - 315	10,236 - 3,871,908
4 (Improved grassland)	502 (42)	2010	1 (1)	2011	0.87 - 329	0.28 (76)	0.79 - 300	6037 - 2,283,565
5 (Rough grassland)	0 (0)	-	0 (0)	-	-	0.11 (0)	-	0
6 (Neutral grassland)	0 (0)	-	0 (0)	-	-	0.01 (0)	-	0
7 (Calcareous grassland)	4 (1)	1985	0 (0)	-	-	0.74 (2)	0.87 - 329	173.9 - 65,790
8 (Acid grassland)	11 (1)	2002	0 (0)	-	-	0.14 (0)	-	0
9 (Fen, Marsh, and Swamp)	0 (0)	-	0 (0)	-	-	-	-	0
10 (Heather)	0 (0)	-	0 (0)	-	-	0.17 (0)	-	0
11 (Heather grassland)	0 (0)	-	0 (0)	-	-	0.13 (0)	-	0
12 (Bog)	0 (0)	-	0 (0)	-	-	0.14 (0)	-	0
13 (Montane habitat)	0 (0)	-	0 (0)	-	-	0.16 (0)	-	0
14 (Inland rock)	0 (0)	-	0 (0)	-	-	0.06 (0)	-	0
15 (Saltwater)	2 (1)	2012	0 (0)	-	-	0.29 (1)	0.39 - 147	38.82 - 14,684
16 (Freshwater)	0 (0)	-	0 (0)	-	-	0.09 (0)	-	0
17 (Supra-littoral rock)	0 (0)	-	0 (0)	-	-	0.05 (0)	-	0
18 (Supra-littoral sediment)	0 (0)	-	0 (0)	-	-	0.15 (0)	-	0
19 (Littoral rock)	0 (0)	-	0 (0)	-	-	0.15 (0)	-	0
20 (Littoral sediment)	0 (0)	-	0 (0)	-	-	0.21 (0)	-	0
21 (Saltmarsh)	0 (0)	-	0 (0)	-	-	-	-	0
22 (Urban)	0 (0)	-	0 (0)	-	-	0.22 (0)	-	0
23 (Suburban)	7 (5)	1994	0 (0)	-	-	0.32 (5)	0.66 - 249	329.4 - 124,585
Total	956 (100)	2009	1 (1)	2011	0.87 - 329	0.25 (214)	0.81 - 308	17,424 - 6,590,798

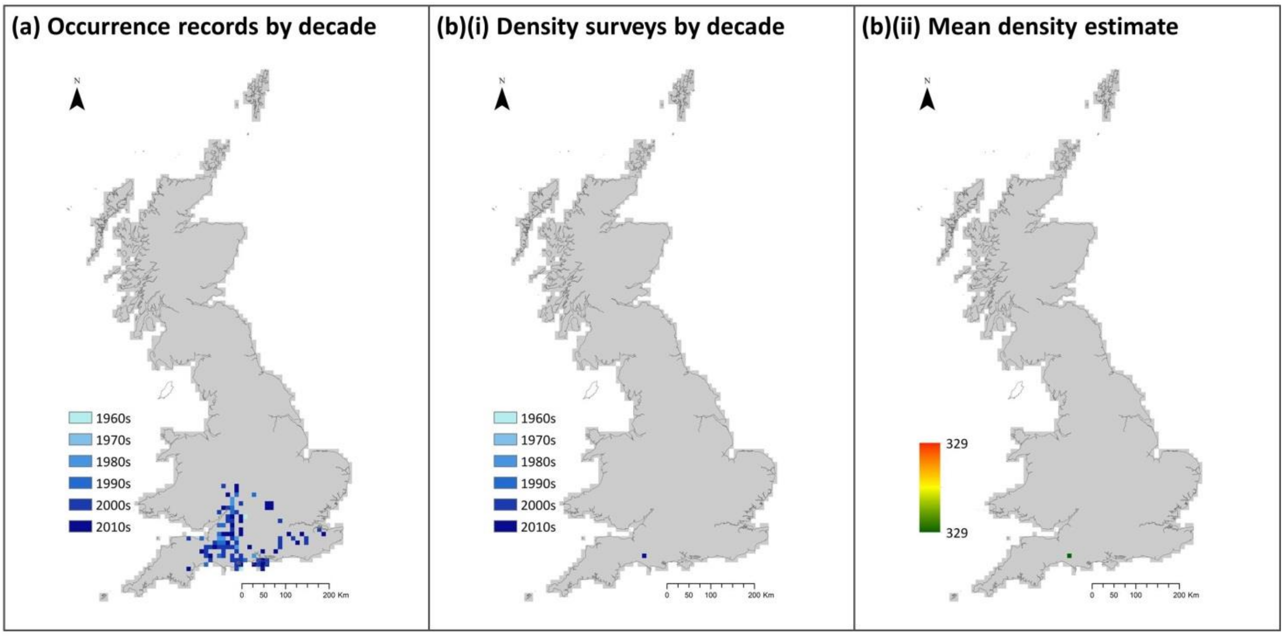

© Crown copyright and database rights 2016 Ordnance Survey 100051110. Data courtesy of the NBN Gateway with thanks to all data contributors. The NBN and its data contributors bear no responsibility for the further analysis or interpretation of this material, data and/or information.

Figure 1: 10km resolution raster maps based on BNG presenting the geographic description of available data. (a) shows the distribution of species occurrence obtained via the NBN Gateway categorised by the decade of last sighting. (b) shows information relating to density surveys identified via a search of published literature where: (i) categorises surveys by the decade of last survey; and (ii) shows the mean density estimate of surveys within grid cells (estimates assumed to be representative of entire cell, considered the upper limit of observed density).

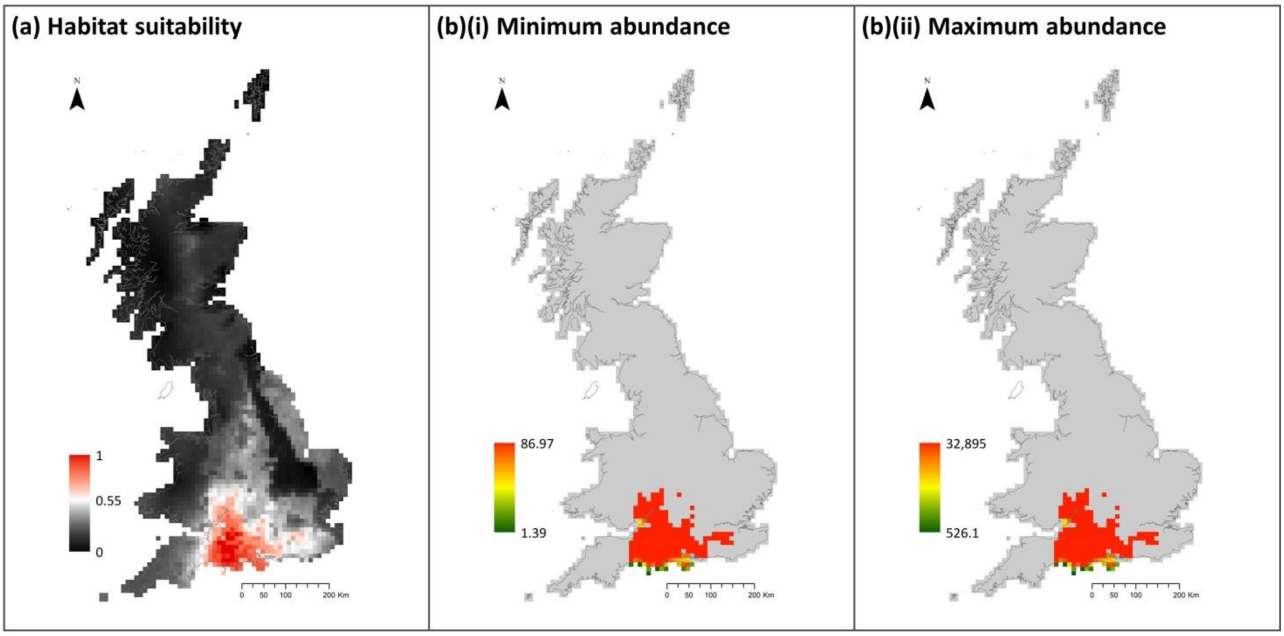

© Crown copyright and database rights 2016 Ordnance Survey 100051110. Data courtesy of the NBN Gateway with thanks to all data contributors. The NBN and its data contributors bear no responsibility for the further analysis or interpretation of this material, data and/or information.

Figure 2: Modelling predictions generated using systematic approach based on available data. (a) shows habitat suitability scores (the likelihood of observing the target species within each grid cell given variation environmental variables) determined by aggregating outputs from the "best" species distribution model (7 models compared) across 100 simulations. Here, the mid value on the scale denotes the threshold score above which occurrence is assumed. (b) shows: (i) the lower bound (Minimum); and (ii) the upper bound (Maximum); of abundance estimates determined by relating observed density (taking into account potential uncertainty) with habitat suitability scores using linear regression.
