# Supplementary material for: A systematic approach to estimate the distribution and total abundance of British mammals
Source: PLoS One. 2017 Jun 28;12(6):e0176339. doi: 10.1371/journal.pone.0176339 (PMC5489149; doi:10.1371/journal.pone.0176339)
Supplement: S5 File — Individual reports for each of the Chiroptera species presenting analysis of the available data and subsequent model predictions based on a 10km raster grid. Reports also include expert comment assessing the reliability (and plausibility) of results in the context of existing evidence and popular opinion. (ZIP) [file pone.0176339.s005.zip › C Brandts bat.pdf]

Brandt's bat (*Myotis brandtii*)

Order: *Chiroptera*

Genus: *Myotis*

Origin: Native

Status: Locally common

1995 abundance estimate: 30,000 (5)

Reported population trends: JNCC 2005, BCT 2014 (↔)

Data:

The available occurrence records indicate that Brandt's bat are locally distributed throughout England and Wales (Figure 1a). These observations were reported in various habitats (predominantly arable and improved grassland) the majority of which since 1995.

From the literature review we identified a single survey (Jones et al. 1996) conducted in northern England in 1990 which estimated density to be approximately 1.7 per km^2 (Figure 1b). This survey only sampled habitats dominated by arable and improved grassland, consequently no estimates were available for other habitats where occurrence was observed (marked grey in Table 1).

Model predictions:

The habitat suitability map (Figure 2a) appears to reflect the underlying data reasonably well with the set of "best" models predicting presence (and absence) to a mean AUC of 0.63. However, the resulting distribution is substantially larger than the area described by the observations (approximately 4 times) and includes some presence predicted in Scotland where no sightings were available. Overall, across 100 repetitions Support Vector Machine proved to be the most commonly selected modelling approach displaying the highest AUC 34% of the time followed by Mahalanobis distance (22%) and BioClim (16%). By land cover the mean habitat suitability scores suggest observation is most likely in landscapes dominated by calcareous grassland (Table 1) but, consistent with recorded sightings, the majority of occurrence is predicted in grid cells dominated by improved grassland.

Due to the limited number and variability of density estimates it was not possible to assess any relationship with habitat suitability. Instead, a constant mean estimate was applied to all cells where occurrence was predicted and summed to derive total abundance.

In agreement with recently reported trends (JNCC, BCT) the predicted abundance range contains the estimate from Harris et al. (1995) suggesting no significant change in the total population (as the 1995 estimate was based on the same density survey this is perhaps unsurprising; this is instead an indication that there have been no significant changes in the species distribution since 1995, despite the apparent expansion of predictions in relation that of observations which is highlighted above). However, the range is large due to the uncertainty caused by the conversion of irregular survey sites to the 10km raster grid upon which modelling is performed. Based on the total area reported in the published study (which shows large areas of absence in the landscape) it may be reasonable to argue that the true abundance lies towards the lower end of the predicted range.

Reliability (Expert comment):

The distribution of available occurrence records is plausible although it does not include recent reports of the species in southern Scotland. In this context it is interesting that this species is predicted to occur across the Scottish borders and the central lowlands of Scotland. Given the general lack of information on this species it is difficult to comment on the relative size of the density estimate in the context of a national range. However, it is likely that the value is an underestimation of the population in this geographical region.

Arguably, the distribution suggested by the habitat suitability map remains consistent with that observed and the increased coverage is not unreasonable. The association with habitats typical of British lowland agricultural landscapes is unsurprising given the location and known ecology of the species though the predicted constraint to the wetter and

warmer areas of Great Britain is in contracts to similar species (e.g. *M. mystacinus*) might be worthy of future targeted research.

The estimate made by Harris et al. (1995) presents a reasonable figure for total abundance and is comparable to the lower bound of the predicted range. However, the upper bound is unrealistically high. In addition to this the maps of predicted abundance show less variability than expected (Figure 2b). As explained previously this due to the constraints imposed on the modelling process by a lack of georeferenced density estimates.

References:

Harris, S. J., P. Morris, S. Wray and D. Yalden (1995). A review of British mammals: population estimates and conservation status of British mammals other than cetaceans, Joint Nature Conservation Committee, Peterborough, UK.

Jones, K. E., J. D. Altringham and R. Deaton (1996). Distribution and population densities of seven species of bat in northern England. *Journal of Zoology* 240(4): 788-798.

Table 1: Summary of observed data and model predictions by land cover class (LCM2007 target classification). Values shown in brackets denote the spatial coverage based on a 10km resolution raster map (number of grid cells). Years represent the median of records within each land class. Ranges for density and abundance are derived using the respective minimum and maximum raster maps (lower bound is mean of values across minimum raster map with upper across the maximum) which capture the spatial uncertainty generate by projecting irregular polygons describing survey sites onto a raster grid.

LCM2007 class	Observed					Predicted		
	Occurrence		Density					
	Records	Year	Estimates	Year	Range	Habitat suitability	Density	Abundance
1 (Broadleaved woodland)	2 (1)	1993	0 (0)	-	-	0.46 (2)	0.23 - 1.7	46.93 - 340
2 (Coniferous woodland)	16 (4)	1988	0 (0)	-	-	0.25 (6)	0.23 - 1.7	140.8 - 1,020
3 (Arable and Horticultural)	265 (79)	2004	3 (3)	1990	0.16 - 1.7	0.44 (232)	0.22 - 1.58	5,070 - 36,733
4 (Improved grassland)	455 (117)	2001	5 (5)	1990	0.27 - 1.7	0.57 (508)	0.22 - 1.57	11,033 - 79,937
5 (Rough grassland)	2 (1)	1981	0 (0)	-	-	0.19 (4)	0.18 - 1.29	71.35 - 516.9
6 (Neutral grassland)	0 (0)	-	0 (0)	-	-	0.08 (0)	-	0
7 (Calcareous grassland)	1 (1)	1985	0 (0)	-	-	0.68 (2)	0.23 - 1.7	46.93 - 340
8 (Acid grassland)	79 (11)	2000	0 (0)	-	-	0.3 (39)	0.23 - 1.69	912 - 6,607
9 (Fen, Marsh, and Swamp)	0 (0)	-	0 (0)	-	-	-	-	0
10 (Heather)	0 (0)	-	0 (0)	-	-	0.25 (6)	0.2 - 1.42	117.4 - 850.5
11 (Heather grassland)	8 (4)	1994	0 (0)	-	-	0.19 (5)	0.23 - 1.7	117.3 - 850
12 (Bog)	19 (1)	2014	0 (0)	-	-	0.2 (11)	0.23 - 1.7	258.1 - 1,870
13 (Montane habitat)	1 (1)	1978	0 (0)	-	-	0.2 (0)	-	0
14 (Inland rock)	0 (0)	-	0 (0)	-	-	0.14 (0)	-	0
15 (Saltwater)	3 (1)	2006	0 (0)	-	-	0.41 (1)	0.09 - 0.68	9.39 - 68.05
16 (Freshwater)	0 (0)	-	0 (0)	-	-	0.2 (0)	-	0
17 (Supra-littoral rock)	0 (0)	-	0 (0)	-	-	0.15 (0)	-	0
18 (Supra-littoral sediment)	0 (0)	-	0 (0)	-	-	0.26 (0)	-	0
19 (Littoral rock)	0 (0)	-	0 (0)	-	-	0.2 (0)	-	0
20 (Littoral sediment)	3 (2)	2004	0 (0)	-	-	0.38 (4)	0.18 - 1.28	70.68 - 512.1
21 (Saltmarsh)	0 (0)	-	0 (0)	-	-	-	-	0
22 (Urban)	0 (0)	-	0 (0)	-	-	0.24 (0)	-	0
23 (Suburban)	12 (5)	1987	0 (0)	-	-	0.33 (0)	-	0
Total	866 (228)	2001	8 (8)	1990	0.23 - 1.7	0.41 (820)	0.22 - 1.58	17,894 - 1,296,456

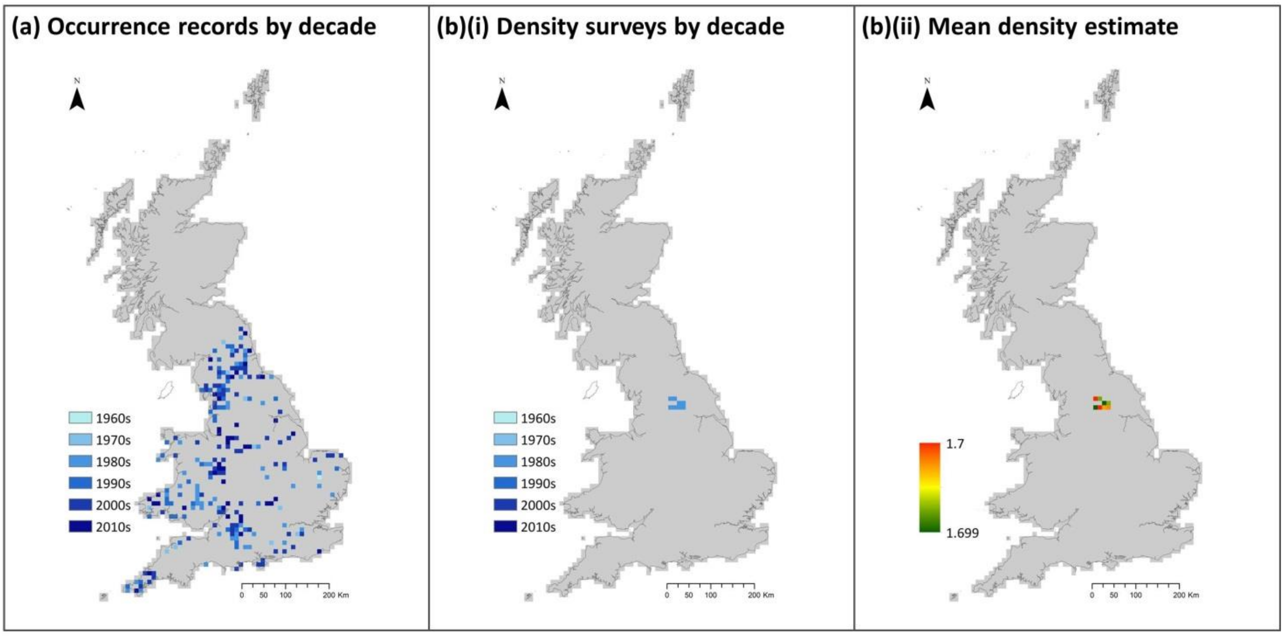

© Crown copyright and database rights 2016 Ordnance Survey 100051110. Data courtesy of the NBN Gateway with thanks to all data contributors. The NBN and its data contributors bear no responsibility for the further analysis or interpretation of this material, data and/or information.

Figure 1: 10km resolution raster maps based on BNG presenting the geographic description of available data. (a) shows the distribution of species occurrence obtained via the NBN Gateway categorised by the decade of last sighting. (b) shows information relating to density surveys identified via a search of published literature where: (i) categorises surveys by the decade of last survey; and (ii) shows the mean density estimate of surveys within grid cells (estimates assumed to be representative of entire cell, considered the upper limit of observed density).

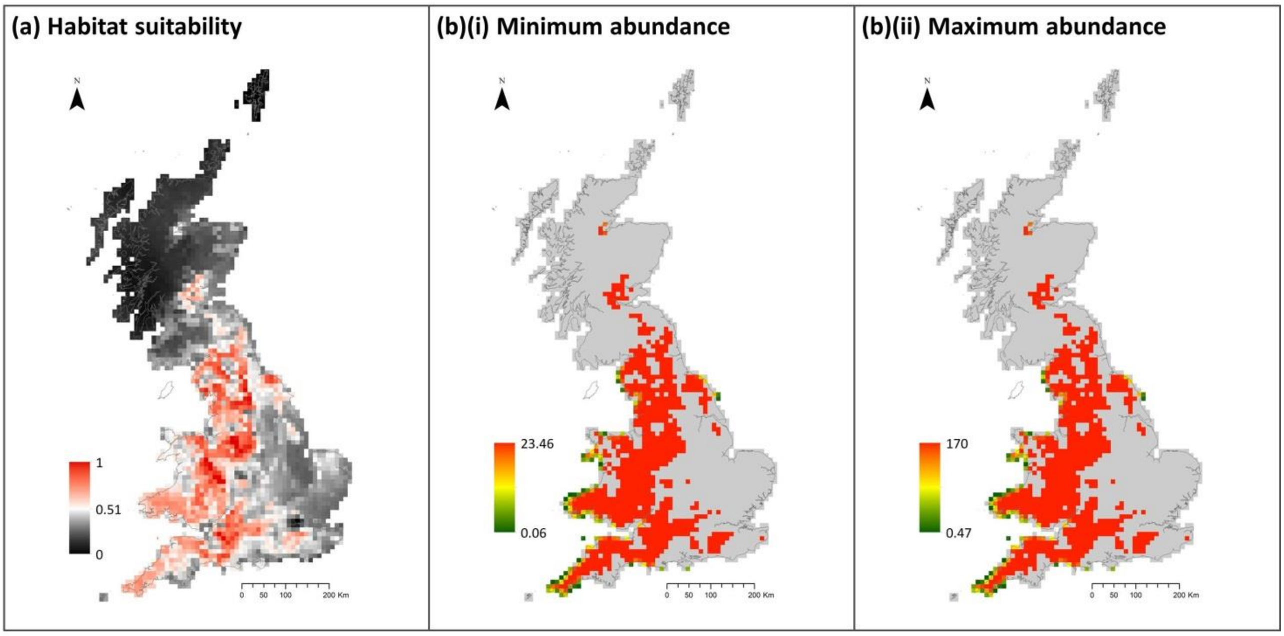

© Crown copyright and database rights 2016 Ordnance Survey 100051110. Data courtesy of the NBN Gateway with thanks to all data contributors. The NBN and its data contributors bear no responsibility for the further analysis or interpretation of this material, data and/or information.

Figure 2: Modelling predictions generated using systematic approach based on available data. (a) shows habitat suitability scores (the likelihood of observing the target species within each grid cell given variation environmental variables) determined by aggregating outputs from the "best" species distribution model (7 models compared) across 100 simulations. Here, the mid value on the scale denotes the threshold score above which occurrence is assumed. (b) shows: (i) the lower bound (Minimum); and (ii) the upper bound (Maximum); of abundance estimates determined by relating observed density (taking into account potential uncertainty) with habitat suitability scores using linear regression.
