# Supplementary material for: A systematic approach to estimate the distribution and total abundance of British mammals
Source: PLoS One. 2017 Jun 28;12(6):e0176339. doi: 10.1371/journal.pone.0176339 (PMC5489149; doi:10.1371/journal.pone.0176339)
Supplement: S5 File — Individual reports for each of the Chiroptera species presenting analysis of the available data and subsequent model predictions based on a 10km raster grid. Reports also include expert comment assessing the reliability (and plausibility) of results in the context of existing evidence and popular opinion. (ZIP) [file pone.0176339.s005.zip › D Brown long-eared bat.pdf]

Brown long-eared bat (*Plecotus auritus*)

Order: *Chiroptera* **Genus:** *Plecotus*

Origin: Native **Status:** Common

1995 abundance estimate: 200,000 (4) **Reported population trends:** JNCC 2005, BCT 2014 (↔)

Data:

The available occurrence records indicate that the brown long-eared bat is widespread throughout Britain with sightings reported in more than half of 10 km squares at least once (approximately 59%), most over the past decade (Figure 1a). However, the map highlights several areas, particularly in the west of Scotland and in Yorkshire, where the species has never been reported.

Density estimates, recorded over the past three decades, were obtained from published literature (Fairless 2013; Jones et al. 1996; Speakman et al. 1991) spanning approximately 3% of the observed species distribution based on the available occurrence data (Figure 1b). Estimates ranged between 1.4 and 2.89 per km^2 with the highest densities recorded in landscapes dominated by improved grassland (0.37 - 1.73 per km^2 accounting for uncertainty relating to unsurveyed areas within grid cells). Due to the limited coverage of the surveys estimates were unavailable for several dominant land covers where occurrence was reported (marked grey in Table 1).

Model predictions:

The habitat suitability map (Figure 2a) appears to reflect the underlying data well with the set of "best" models predicting presence (and absence) to a mean AUC of 0.75. Overall, across 100 repetitions MaxEnt proved to be the most commonly selected modelling approach displaying the highest AUC 43% of the time followed by Generalised Linear Models (23%). By land cover the mean habitat suitability scores suggest observation is most likely in landscapes dominated by calcareous grassland and broadleaved woodland (Table 1) but, consistent with recorded sightings, the majority of occurrence is predicted in grid cells dominated by arable and improved grassland. The analysis shows that occurrence is preserved in all land covers where it is observed with the exception of freshwater and saltwater dominated habitat.

Linear regression suggested that there was no correlation between the estimates of maximum density and habitat suitability; consequently, it was applied as a constant in cells where occurrence was predicted. Minimum density was found to be correlated with the best fit model relating the square of habitat suitability accounting for spherical spatial autocorrelation.

The predicted abundance range contains the estimate from Harris et al. (1995) suggesting no significant change in the total population, and this trend is in agreement with other reports (as the 1995 estimate was based on the same density survey this is perhaps unsurprising; this could instead be considered an indication that there have been no significant changes in the species distribution since 1995). The lack of correlation with maximum density perhaps indicates that the assumption applied to minimum density are more appropriate and therefore the true estimate of abundance should lies towards the lower end of the predicted range.

Reliability (Expert comment):

The brown long-eared bat is ubiquitous in Britain. Whilst the available occurrence records reflect this there are notable gaps in reports from Scotland and northern England . These are due to under reporting in these areas and should not be considered as an indication of absence. The density estimates obtained from the published literature provide a believable set of values around a national mean but may not fully represent the range which may include some areas with higher densities. Geographically, the appearance of higher densities recorded in some of the southern counties of England is not unexpected (Figure 1b).

The predicted habitat suitability map is plausible with the thresholded area presenting a reasonable reflection of regions likely to support higher densities (e.g. more marginal areas such as western Scotland are excluded). The association with habitats representing agricultural landscapes typical of the British lowlands (including broadleaved woodland, isolated standards as well as trees in hedgerows and shelterbelts) is not uncharacteristic for this species.

The estimate made by Harris et al. (1995) may still represent a plausible reflection of the total population for this species as it is in agreement with the predicted abundance range and its ubiquity suggests no reason to anticipate any significant bias in our estimates of abundance.

References:

Fairless, L. (2013). Ecology and ecophysiology of social structure and population dynamics in bats (*Vespertilionidae*). Ph.D. Thesis, University of Southampton.

Harris, S. J., P. Morris, S. Wray and D. Yalden (1995). A review of British mammals: population estimates and conservation status of British mammals other than cetaceans, Joint Nature Conservation Committee, Peterborough, UK.

Jones, K. E., J. D. Altringham and R. Deaton (1996). Distribution and population densities of seven species of bat in northern England. *Journal of Zoology* 240(4): 788-798.

Speakman, J. R., P. A. Racey, C. M. C. Catto, P. I. Webb, S. M. Swift and A. M. Burnett (1991). Minimum summer populations and densities of bats in NE Scotland, near the northern borders of their distributions. *Journal of Zoology* 225(2): 327-345.

Table 1: Summary of observed data and model predictions by land cover class (LCM2007 target classification). Values shown in brackets denote the spatial coverage based on a 10km resolution raster map (number of grid cells). Years represent the median of records within each land class. Ranges for density and abundance are derived using the respective minimum and maximum raster maps (lower bound is mean of values across minimum raster map with upper across the maximum) which capture the spatial uncertainty generate by projecting irregular polygons describing survey sites onto a raster grid.

LCM2007 class	Observed					Predicted		
	Occurrence		Density					
	Records	Year	Estimates	Year	Range	Habitat suitability	Density	Abundance
1 (Broadleaved woodland)	228 (10)	2013	0 (0)	-	-	0.91 (11)	1.86 - 2.27	2,050 - 2,495
2 (Coniferous woodland)	707 (70)	2004	4 (4)	1987	0.75 - 1.66	0.63 (49)	0.73 - 2.26	3,588 - 11,074
3 (Arable and Horticultural)	15,458 (759)	2012	15 (15)	1990	0.34 - 1.5	0.84 (909)	0.76 - 2.08	69,105 - 189,350
4 (Improved grassland)	8,207 (560)	2010	20 (20)	1990	0.37 - 1.73	0.79 (637)	0.82 - 2.09	51,934 - 133,051
5 (Rough grassland)	105 (10)	2003	0 (0)	-	-	0.31 (6)	0.31 - 1.89	185.5 - 1,135
6 (Neutral grassland)	0 (0)	-	0 (0)	-	-	0 (0)	-	0
7 (Calcareous grassland)	32 (2)	2012	0 (0)	-	-	0.94 (2)	2.37 - 2.27	474.3 - 453.6
8 (Acid grassland)	548 (84)	2002	0 (0)	-	-	0.57 (74)	0.41 - 2.23	3,049 - 16,531
9 (Fen, Marsh, and Swamp)	0 (0)	-	0 (0)	-	-	-	-	0
10 (Heather)	156 (27)	2004	7 (7)	1987	0.28 - 1.62	0.67 (25)	0.5 - 2.18	1,255 - 5,444
11 (Heather grassland)	198 (30)	2006	1 (1)	1987	0.25 - 1.66	0.39 (13)	0.4 - 2.27	519.2 - 2,948
12 (Bog)	69 (17)	2008	0 (0)	-	-	0.3 (5)	0.26 - 2.27	130.6 - 1,134
13 (Montane habitat)	56 (9)	2001	0 (0)	-	-	0.41 (1)	0.19 - 2.27	19.02 - 226.8
14 (Inland rock)	0 (0)	-	0 (0)	-	-	0.09 (0)	-	0
15 (Saltwater)	29 (3)	2012	0 (0)	-	-	0.62 (0)	-	0
16 (Freshwater)	2 (1)	1994	0 (0)	-	-	0.46 (0)	-	0
17 (Supra-littoral rock)	0 (0)	-	0 (0)	-	-	0.02 (0)	-	0
18 (Supra-littoral sediment)	0 (0)	-	0 (0)	-	-	0.3 (0)	-	0
19 (Littoral rock)	0 (0)	-	0 (0)	-	-	0.22 (0)	-	0
20 (Littoral sediment)	436 (14)	2002	0 (0)	-	-	0.65 (2)	0.17 - 1.69	33.9 - 339
21 (Saltmarsh)	0 (0)	-	0 (0)	-	-	-	-	0
22 (Urban)	20 (3)	2010	0 (0)	-	-	0.68 (1)	0.06 - 0.74	6.06 - 73.75
23 (Suburban)	507 (51)	2012	0 (0)	-	-	0.76 (49)	0.23 - 2.02	1,147 - 9,893
Total	26,758 (1,650)	2011	47 (47)	1990	0.38 - 1.63	0.7 (1,784)	0.75 - 2.1	133,497 - 374,147

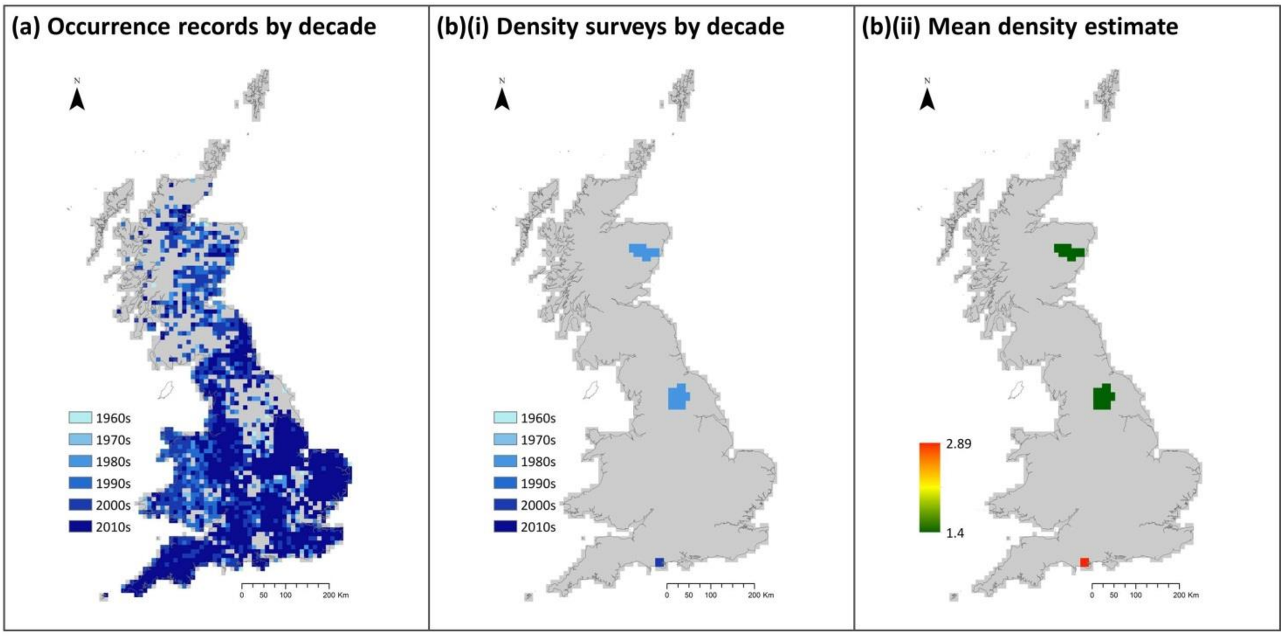

© Crown copyright and database rights 2016 Ordnance Survey 100051110. Data courtesy of the NBN Gateway with thanks to all data contributors. The NBN and its data contributors bear no responsibility for the further analysis or interpretation of this material, data and/or information.

Figure 1: 10km resolution raster maps based on BNG presenting the geographic description of available data. (a) shows the distribution of species occurrence obtained via the NBN Gateway categorised by the decade of last sighting. (b) shows information relating to density surveys identified via a search of published literature where: (i) categorises surveys by the decade of last survey; and (ii) shows the mean density estimate of surveys within grid cells (estimates assumed to be representative of entire cell, considered the upper limit of observed density).

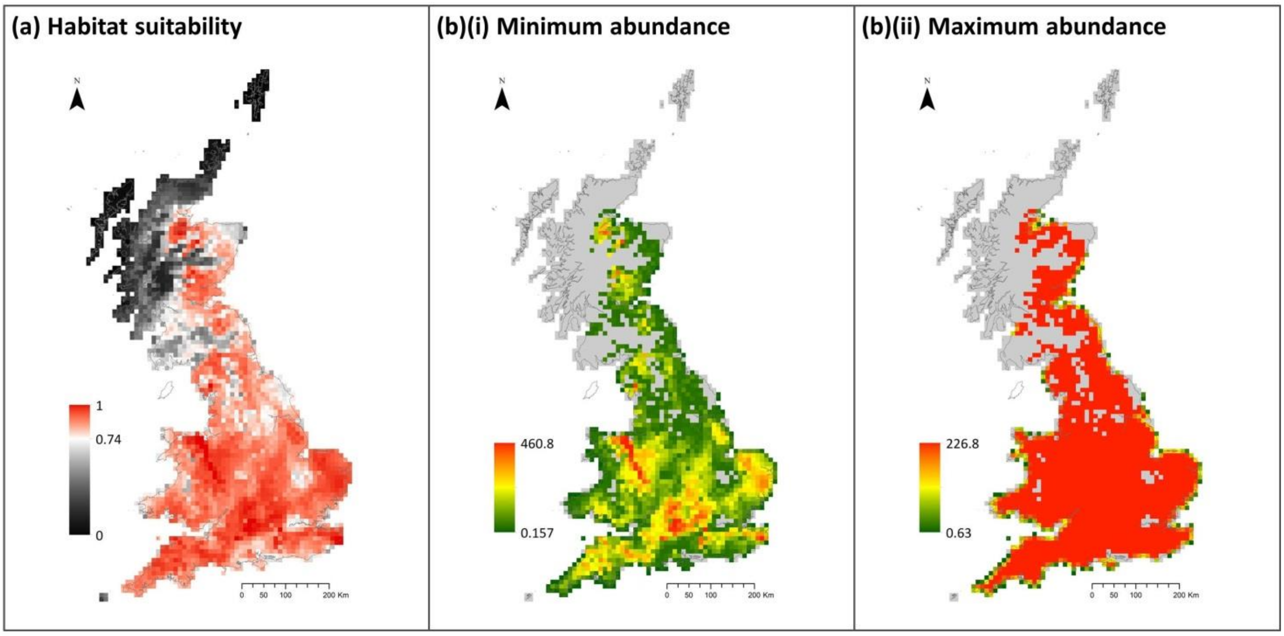

© Crown copyright and database rights 2016 Ordnance Survey 100051110. Data courtesy of the NBN Gateway with thanks to all data contributors. The NBN and its data contributors bear no responsibility for the further analysis or interpretation of this material, data and/or information.

Figure 2: Modelling predictions generated using systematic approach based on available data. (a) shows habitat suitability scores (the likelihood of observing the target species within each grid cell given variation environmental variables) determined by aggregating outputs from the "best" species distribution model (7 models compared) across 100 simulations. Here, the mid value on the scale denotes the threshold score above which occurrence is assumed. (b) shows: (i) the lower bound (Minimum); and (ii) the upper bound (Maximum); of abundance estimates determined by relating observed density (taking into account potential uncertainty) with habitat suitability scores using linear regression.
