# Supplementary material for: A systematic approach to estimate the distribution and total abundance of British mammals
Source: PLoS One. 2017 Jun 28;12(6):e0176339. doi: 10.1371/journal.pone.0176339 (PMC5489149; doi:10.1371/journal.pone.0176339)
Supplement: S5 File — Individual reports for each of the Chiroptera species presenting analysis of the available data and subsequent model predictions based on a 10km raster grid. Reports also include expert comment assessing the reliability (and plausibility) of results in the context of existing evidence and popular opinion. (ZIP) [file pone.0176339.s005.zip › E Daubentons bat.pdf]

Daubenton's bat (*Myotis daubentonii*)

Order: *Chiroptera*

Genus: *Myotis*

Origin: Native

Status: Common

1995 abundance estimate: 150,000 (4)

Reported population trends: JNCC 2005 (↑), BCT 2014 (↔)

Data:

The available occurrence records indicate that Daubenton's bat are widely distributed throughout England and Wales with a notable absence of records in the north west of Scotland (Figure 1a). Observations were reported in a variety of landscape types (predominantly those dominated by arable and improved grassland) the majority (of cells) reporting at least one record since 1995.

Two surveys conducted in northern England and north east Scotland between 1987 and 1990 (Figure 1b) yielded estimates of density (Jones et al. 1996; Speakman et al. 1991). Estimates ranged between 1 and 2.4 per km^2 with the highest densities recorded in habitats dominated by broadleaved woodland, heather and heather grassland. Unfortunately, these surveys only sampled a limited selection of dominant land covers, consequently estimates were unavailable for some habitats where occurrence was observed (marked grey in Table 1).

Model predictions:

The habitat suitability map (Figure 2a) appears to reflect the underlying data reasonably well with the set of "best" models predicting presence (and absence) to a mean AUC of 0.69. Overall, across 100 repetitions MaxEnt proved to be the most commonly selected modelling approach displaying the highest AUC 30% of the time followed by Random Forest (24%) and Generalised Linear Models (23%). By land cover the mean habitat suitability scores suggest observation is most likely in landscapes dominated by arable and broadleaved woodland (Table 1) but, consistent with recorded sightings, the majority of occurrence is predicted in grid cells dominated by arable and improved grassland.

Neither minimum nor maximum density estimates showed a correlation with habitat suitability. Perhaps due to the limited volume and variability of density data.. Minimum density was best fitted using a GLM with a gamma distribution. Whereas, maximum density was normally distributed and suggested a best fit using spherical spatial autocorrelation.

Despite the difficulties in fitting density to habitat suitability the predicted abundance range contains the estimate from Harris et al. (1995) which in agreement with recent trend analysis and suggests no significant change in the total population (as the 1995 estimate was based on the same density survey this is perhaps unsurprising; this could instead be considered an indication that there have been no significant changes in the species distribution since 1995). Unlike many of the estimates presented the predicted range is quite narrow with an even distribution around the 1995 estimate of 150,000.

Reliability (Expert comment):

Daubenton's bat is ubiquitous in Britain. Whilst the available occurrence records reflect this there are notable gaps in the recorded distribution. These gaps are unlikely to be a true indication of absence but could be linked to the predominant records of this species on the NBN being made at accessible waterways and water bodies. The density estimates obtained from the published literature are low compared to the likely national range with much higher densities possible in suitable landscapes. Although, it should be noted that it is difficult to gauge two dimensional densities for a species with one dimensional behaviour. Given the information presented it is not possible to comment on the spatial positioning of estimates as in this case local contexts (roost and foraging) are more important than climate and geography and all are confounded by the use of grids and the spatial scale of study.

The habitat suitability map is plausible given the observed records and the main associations with particular dominant land covers are as expected.

Both the Harris et al. (1995) estimate and the range of predicted abundance are low. The upper end of the predicted range could be plausible for a mean number. Given the generalist nature of the species it may be reasonable to argue that this upper bound is more likely given the assumptions made during the modelling process.

References:

Harris, S. J., P. Morris, S. Wray and D. Yalden (1995). A review of British mammals: population estimates and conservation status of British mammals other than cetaceans, Joint Nature Conservation Committee, Peterborough, UK.

Jones, K. E., J. D. Altringham and R. Deaton (1996). Distribution and population densities of seven species of bat in northern England. *Journal of Zoology* 240(4): 788-798.

Speakman, J. R., P. A. Racey, C. M. C. Catto, P. I. Webb, S. M. Swift and A. M. Burnett (1991). Minimum summer populations and densities of bats in NE Scotland, near the northern borders of their distributions. *Journal of Zoology* 225(2): 327-345.

Table 1: Summary of observed data and model predictions by land cover class (LCM2007 target classification). Values shown in brackets denote the spatial coverage based on a 10km resolution raster map (number of grid cells). Years represent the median of records within each land class. Ranges for density and abundance are derived using the respective minimum and maximum raster maps (lower bound is mean of values across minimum raster map with upper across the maximum) which capture the spatial uncertainty generate by projecting irregular polygons describing survey sites onto a raster grid.

LCM2007 class	Observed					Predicted		
	Occurrence		Density					
	Records	Year	Estimates	Year	Range	Habitat suitability	Density	Abundance
1 (Broadleaved woodland)	86 (8)	2011	0 (0)	-	-	0.86 (8)	0.27 - 1.71	219.5 - 1,370
2 (Coniferous woodland)	639 (58)	2006	2 (2)	1987	0.55 - 2.4	0.65 (42)	0.27 - 1.71	1148 - 7,166
3 (Arable and Horticultural)	9913 (612)	2011	6 (6)	1990	0.23 - 1	0.86 (754)	0.26 - 1.64	19,772 - 123,383
4 (Improved grassland)	6902 (480)	2010	6 (6)	1990	0.26 - 1.23	0.82 (582)	0.26 - 1.63	15,222 - 94,991
5 (Rough grassland)	97 (10)	2008	0 (0)	-	-	0.32 (7)	0.27 - 1.7	191.1 - 1,192
6 (Neutral grassland)	0 (0)	-	0 (0)	-	-	0 (0)	-	0
7 (Calcareous grassland)	0 (0)	-	0 (0)	-	-	0.57 (0)	-	0
8 (Acid grassland)	472 (64)	2006	0 (0)	-	-	0.58 (42)	0.27 - 1.71	1,149 - 7,170
9 (Fen, Marsh, and Swamp)	0 (0)	-	0 (0)	-	-	-	-	0
10 (Heather)	66 (13)	2000	5 (5)	1987	0.26 - 2.4	0.62 (0)	-	0
11 (Heather grassland)	119 (25)	2006	1 (1)	1987	0.11 - 2.4	0.4 (10)	0.27 - 1.71	274.4 - 1,712
12 (Bog)	73 (19)	2006	0 (0)	-	-	0.36 (8)	0.27 - 1.71	219.5 - 1,370
13 (Montane habitat)	42 (11)	1997	0 (0)	-	-	0.48 (1)	0.27 - 1.71	27.44 - 171.3
14 (Inland rock)	0 (0)	-	0 (0)	-	-	0.08 (0)	-	0
15 (Saltwater)	3 (2)	2009	0 (0)	-	-	0.6 (0)	-	0
16 (Freshwater)	10 (2)	2012	0 (0)	-	-	0.63 (1)	0.27 - 1.68	26.97 - 168.3
17 (Supra-littoral rock)	0 (0)	-	0 (0)	-	-	0.03 (0)	-	0
18 (Supra-littoral sediment)	2 (1)	1993	0 (0)	-	-	0.41 (0)	-	0
19 (Littoral rock)	0 (0)	-	0 (0)	-	-	0.2 (0)	-	0
20 (Littoral sediment)	56 (12)	2008	0 (0)	-	-	0.65 (0)	-	0
21 (Saltmarsh)	0 (0)	-	0 (0)	-	-	-	-	0
22 (Urban)	50 (4)	2013	0 (0)	-	-	0.74 (3)	0.27 - 1.71	82.33 - 513.8
23 (Suburban)	1,082 (54)	2012	0 (0)	-	-	0.8 (35)	0.27 - 1.71	960.3 - 5,992
Total	19,612 (1,375)	2010	20 (20)	1990	0.27 - 1.63	0.73 (1,493)	0.26 - 1.64	39,292 - 245,200

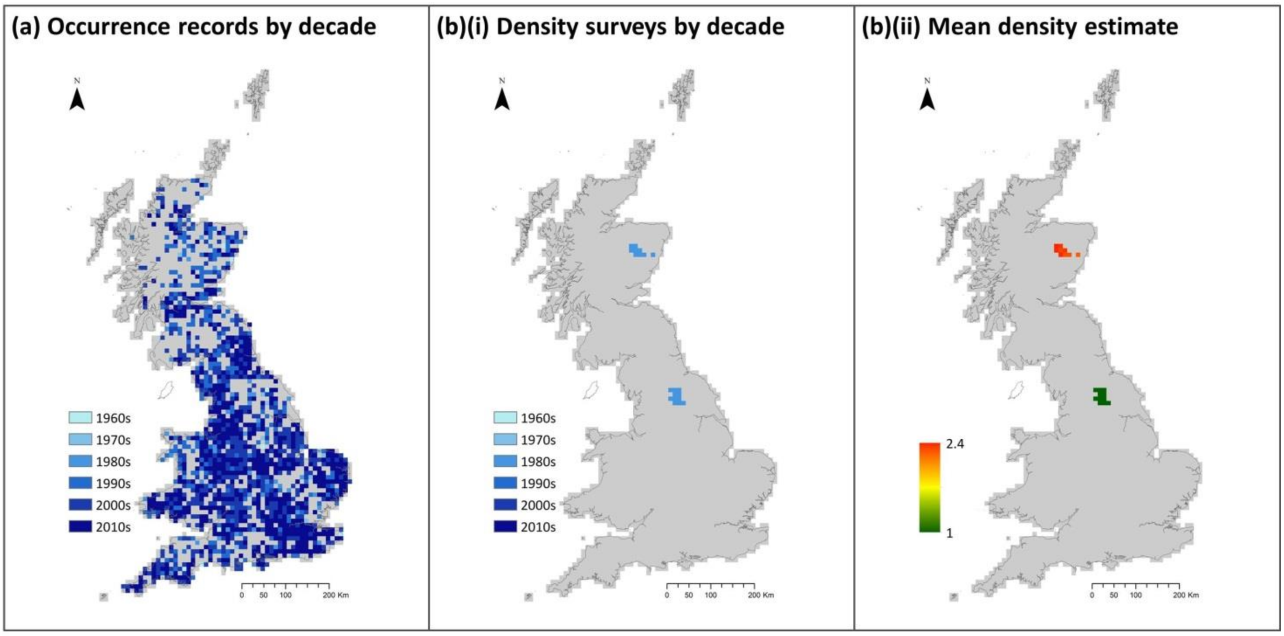

© Crown copyright and database rights 2016 Ordnance Survey 100051110. Data courtesy of the NBN Gateway with thanks to all data contributors. The NBN and its data contributors bear no responsibility for the further analysis or interpretation of this material, data and/or information.

Figure 1: 10km resolution raster maps based on BNG presenting the geographic description of available data. (a) shows the distribution of species occurrence obtained via the NBN Gateway categorised by the decade of last sighting. (b) shows information relating to density surveys identified via a search of published literature where: (i) categorises surveys by the decade of last survey; and (ii) shows the mean density estimate of surveys within grid cells (estimates assumed to be representative of entire cell, considered the upper limit of observed density).

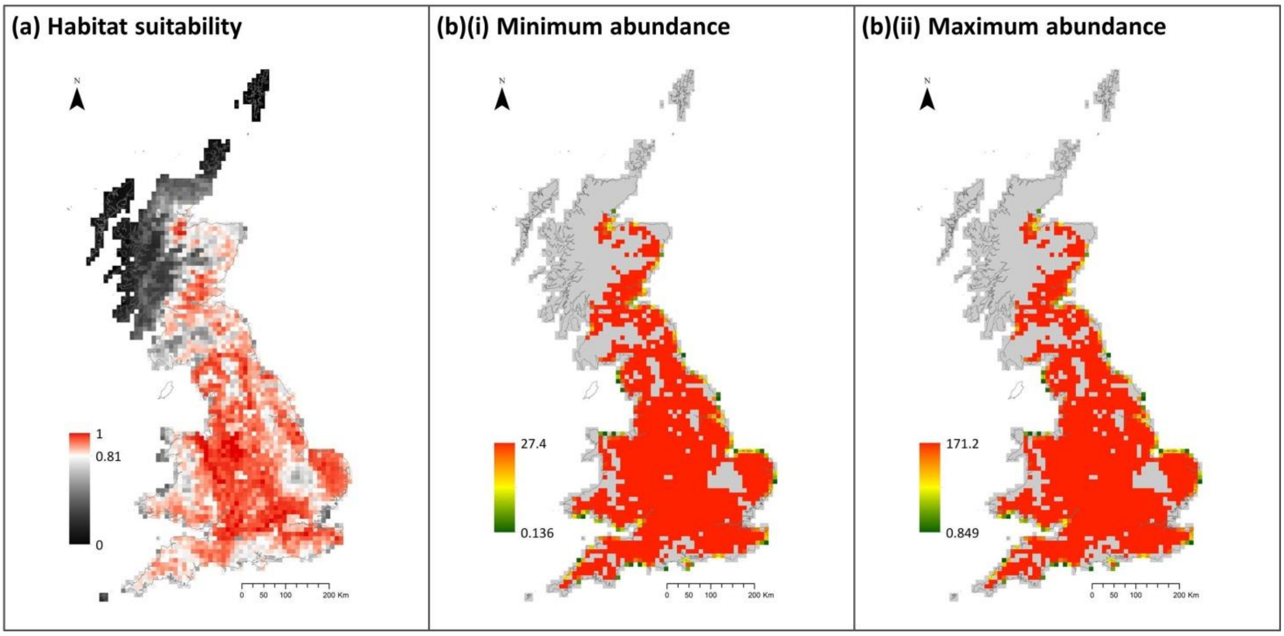

© Crown copyright and database rights 2016 Ordnance Survey 100051110. Data courtesy of the NBN Gateway with thanks to all data contributors. The NBN and its data contributors bear no responsibility for the further analysis or interpretation of this material, data and/or information.

Figure 2: Modelling predictions generated using systematic approach based on available data. (a) shows habitat suitability scores (the likelihood of observing the target species within each grid cell given variation environmental variables) determined by aggregating outputs from the "best" species distribution model (7 models compared) across 100 simulations. Here, the mid value on the scale denotes the threshold score above which occurrence is assumed. (b) shows: (i) the lower bound (Minimum); and (ii) the upper bound (Maximum); of abundance estimates determined by relating observed density (taking into account potential uncertainty) with habitat suitability scores using linear regression.
