# Supplementary material for: A systematic approach to estimate the distribution and total abundance of British mammals
Source: PLoS One. 2017 Jun 28;12(6):e0176339. doi: 10.1371/journal.pone.0176339 (PMC5489149; doi:10.1371/journal.pone.0176339)
Supplement: S5 File — Individual reports for each of the Chiroptera species presenting analysis of the available data and subsequent model predictions based on a 10km raster grid. Reports also include expert comment assessing the reliability (and plausibility) of results in the context of existing evidence and popular opinion. (ZIP) [file pone.0176339.s005.zip › G Greater mouse-eared bat.pdf]

Greater mouse-eared bat (*Myotis myotis*)

Order: *Chiroptera* **Genus:** *Myotis*

Origin: Native **Status:** Extinct

1995 abundance estimate: 0 (1) **Reported population trends:** None

Data:

Only a handful of records describing observations of this species are available (Figure 1a) suggesting the presence of bats in Merseyside and Hampshire; historic sightings (1970s) were recorded on the Dorset and Kent coastlines. All sightings were reported in grid cells dominated by arable and improved grassland dominated habitats.

From the literature review we were unable to identify any publications reporting an estimate of density.

Model predictions:

Unfortunately, there was insufficient data available at a 10km scale to perform any model analysis.

Reliability (Expert comment):

It is widely accepted that a breeding population of this species is not present in Britain. There has been a single recurring sighting verified in Hampshire but it is likely that other records are either old or misidentifications.

References:

None

Table 1: Summary of observed data and model predictions by land cover class (LCM2007 target classification). Values shown in brackets denote the spatial coverage based on a 10km resolution raster map (number of grid cells). Years represent the median of records within each land class. Ranges for density and abundance are derived using the respective minimum and maximum raster maps (lower bound is mean of values across minimum raster map with upper across the maximum) which capture the spatial uncertainty generate by projecting irregular polygons describing survey sites onto a raster grid.

LCM2007 class	Observed					Predicted		
	Occurrence		Density					
	Records	Year	Estimates	Year	Range	Habitat suitability	Density	Abundance
1 (Broadleaved woodland)	0 (0)	-	0 (0)	-	-	-	-	-
2 (Coniferous woodland)	0 (0)	-	0 (0)	-	-	-	-	-
3 (Arable and Horticultural)	49 (4)	2009	0 (0)	-	-	-	-	-
4 (Improved grassland)	12 (4)	1978	0 (0)	-	-	-	-	-
5 (Rough grassland)	0 (0)	-	0 (0)	-	-	-	-	-
6 (Neutral grassland)	0 (0)	-	0 (0)	-	-	-	-	-
7 (Calcareous grassland)	0 (0)	-	0 (0)	-	-	-	-	-
8 (Acid grassland)	0 (0)	-	0 (0)	-	-	-	-	-
9 (Fen, Marsh, and Swamp)	0 (0)	-	0 (0)	-	-	-	-	-
10 (Heather)	0 (0)	-	0 (0)	-	-	-	-	-
11 (Heather grassland)	0 (0)	-	0 (0)	-	-	-	-	-
12 (Bog)	0 (0)	-	0 (0)	-	-	-	-	-
13 (Montane habitat)	0 (0)	-	0 (0)	-	-	-	-	-
14 (Inland rock)	0 (0)	-	0 (0)	-	-	-	-	-
15 (Saltwater)	0 (0)	-	0 (0)	-	-	-	-	-
16 (Freshwater)	0 (0)	-	0 (0)	-	-	-	-	-
17 (Supra-littoral rock)	0 (0)	-	0 (0)	-	-	-	-	-
18 (Supra-littoral sediment)	0 (0)	-	0 (0)	-	-	-	-	-
19 (Littoral rock)	0 (0)	-	0 (0)	-	-	-	-	-
20 (Littoral sediment)	0 (0)	-	0 (0)	-	-	-	-	-
21 (Saltmarsh)	0 (0)	-	0 (0)	-	-	-	-	-
22 (Urban)	0 (0)	-	0 (0)	-	-	-	-	-
23 (Suburban)	0 (0)	-	0 (0)	-	-	-	-	-
Total	61 (8)	1996	0 (0)	-	-	-	-	-

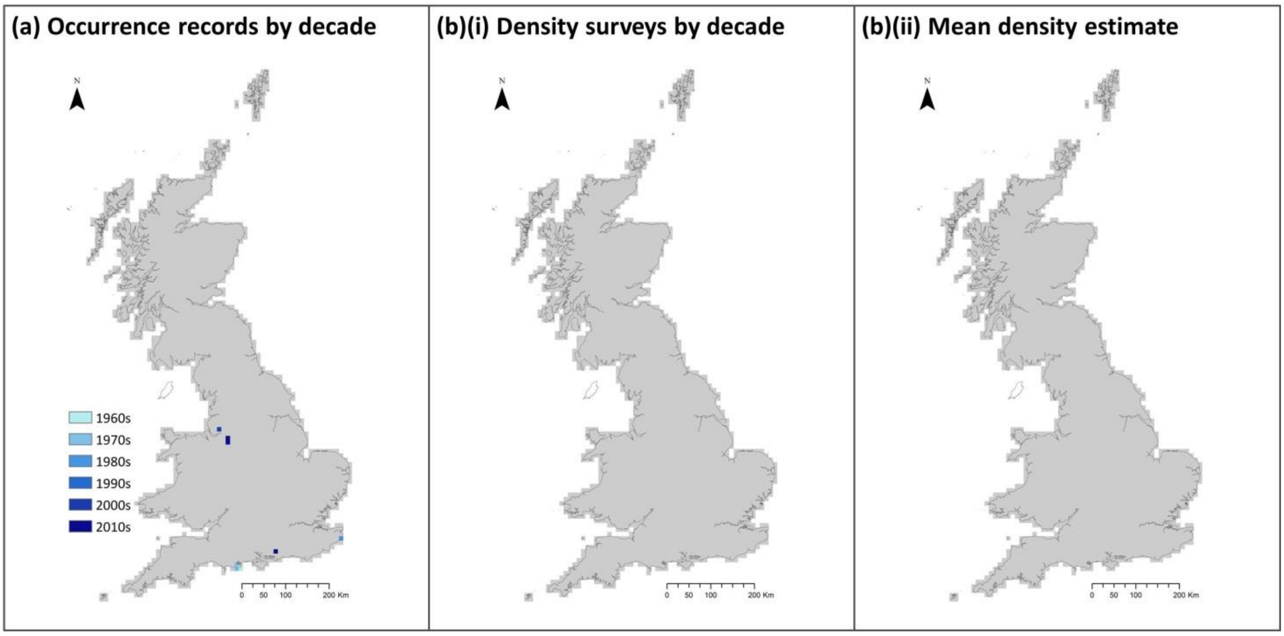

© Crown copyright and database rights 2016 Ordnance Survey 100051110. Data courtesy of the NBN Gateway with thanks to all data contributors. The NBN and its data contributors bear no responsibility for the further analysis or interpretation of this material, data and/or information.

Figure 1: 10km resolution raster maps based on BNG presenting the geographic description of available data. (a) shows the distribution of species occurrence obtained via the NBN Gateway categorised by the decade of last sighting. (b) shows information relating to density surveys identified via a search of published literature where: (i) categorises surveys by the decade of last survey; and (ii) shows the mean density estimate of surveys within grid cells (estimates assumed to be representative of entire cell, considered the upper limit of observed density).

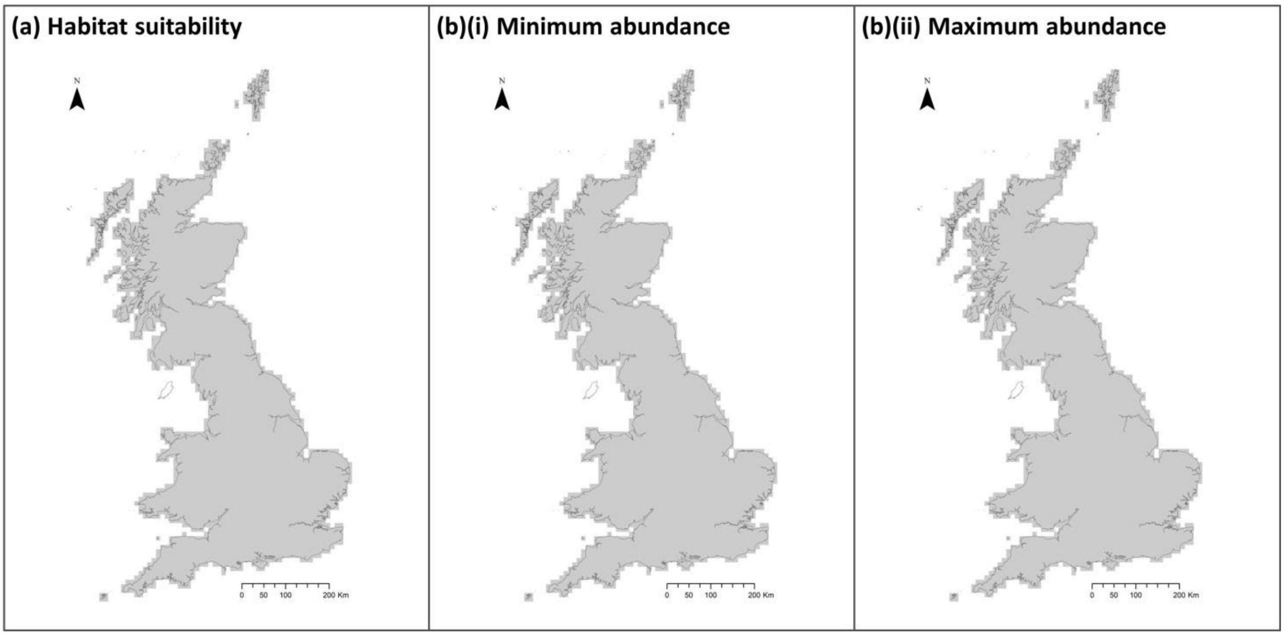

© Crown copyright and database rights 2016 Ordnance Survey 100051110. Data courtesy of the NBN Gateway with thanks to all data contributors. The NBN and its data contributors bear no responsibility for the further analysis or interpretation of this material, data and/or information.

Figure 2: Modelling predictions generated using systematic approach based on available data. (a) shows habitat suitability scores (the likelihood of observing the target species within each grid cell given variation environmental variables) determined by aggregating outputs from the "best" species distribution model (7 models compared) across 100 simulations. Here, the mid value on the scale denotes the threshold score above which occurrence is assumed. (b) shows: (i) the lower bound (Minimum); and (ii) the upper bound (Maximum); of abundance estimates determined by relating observed density (taking into account potential uncertainty) with habitat suitability scores using linear regression.
