# Supplementary material for: A systematic approach to estimate the distribution and total abundance of British mammals
Source: PLoS One. 2017 Jun 28;12(6):e0176339. doi: 10.1371/journal.pone.0176339 (PMC5489149; doi:10.1371/journal.pone.0176339)
Supplement: S5 File — Individual reports for each of the Chiroptera species presenting analysis of the available data and subsequent model predictions based on a 10km raster grid. Reports also include expert comment assessing the reliability (and plausibility) of results in the context of existing evidence and popular opinion. (ZIP) [file pone.0176339.s005.zip › H Grey long-eared bat.pdf]

Grey long-eared bat (*Plecotus austriacus*)

Order: *Chiroptera*

Genus: *Plecotus*

Origin: Native

Status: Very rare

1995 abundance estimate: 1,000 (3)

Reported population trends: None

Data:

The available occurrence records indicate that grey long-eared bats are restricted to distributed small area in southern England (mainly Dorset, Hampshire and the Isle of Wight) with a single sighting in the Midlands (Figure 1a). These observations were predominantly reported in grid cells dominated by arable and improved grassland between 1993 and 2003.

From the literature review we were unable to identify any publications reporting an estimate of density.

Model predictions:

The habitat suitability map (Figure 2a) appears to reflect the underlying data well with the set of "best" models predicting presence (and absence) to a mean AUC of 0.79. Overall, across 100 repetitions MaxEnt proved to be the most commonly selected modelling approach displaying the highest AUC 28% of the time followed by Support Machine Vector (18%). By land cover the mean habitat suitability scores suggest, despite no confirmed sightings, observation is most likely in landscapes dominated by broadleaved woodland (Table 1) perhaps indicating an alternative environmental factor such as climate may be driving this dependence. However, consistent with available records, the majority of occurrence is predicted in arable, improved grassland and suburban habitats.

Unfortunately, due to the lack of density estimates model analysis to predict abundance could not be performed.

Reliability (Expert comment):

The observed occurrence records appear plausible although there are some sightings in Wales than do not appear on the NBN and would add important information to the model. It should be noted that this species is easily confused with the brown long-eared bat and consequently older data could be considered more uncertain.

The resulting habitat suitability map reflects the recorded observations without artefact but disappointingly also does not infer any occurrence in Wales. The association with wooded pastoral landscapes (broadleaved woodland and calcareous grassland) is consistent with current knowledge.

References:

None

Table 1: Summary of observed data and model predictions by land cover class (LCM2007 target classification). Values shown in brackets denote the spatial coverage based on a 10km resolution raster map (number of grid cells). Years represent the median of records within each land class. Ranges for density and abundance are derived using the respective minimum and maximum raster maps (lower bound is mean of values across minimum raster map with upper across the maximum) which capture the spatial uncertainty generate by projecting irregular polygons describing survey sites onto a raster grid.

LCM2007 class	Observed					Predicted		
	Occurrence		Density					
	Records	Year	Estimates	Year	Range	Habitat suitability	Density	Abundance
1 (Broadleaved woodland)	0 (0)	-	0 (0)	-	-	0.41 (4)	-	-
2 (Coniferous woodland)	0 (0)	-	0 (0)	-	-	0.11 (0)	-	-
3 (Arable and Horticultural)	65 (13)	2003	0 (0)	-	-	0.19 (60)	-	-
4 (Improved grassland)	84 (19)	1993	0 (0)	-	-	0.19 (43)	-	-
5 (Rough grassland)	0 (0)	-	0 (0)	-	-	0.11 (0)	-	-
6 (Neutral grassland)	0 (0)	-	0 (0)	-	-	0.09 (0)	-	-
7 (Calcareous grassland)	0 (0)	-	0 (0)	-	-	0.4 (0)	-	-
8 (Acid grassland)	0 (0)	-	0 (0)	-	-	0.12 (0)	-	-
9 (Fen, Marsh, and Swamp)	0 (0)	-	0 (0)	-	-	-	-	-
10 (Heather)	0 (0)	-	0 (0)	-	-	0.1 (0)	-	-
11 (Heather grassland)	0 (0)	-	0 (0)	-	-	0.11 (0)	-	-
12 (Bog)	0 (0)	-	0 (0)	-	-	0.1 (0)	-	-
13 (Montane habitat)	0 (0)	-	0 (0)	-	-	0.1 (0)	-	-
14 (Inland rock)	0 (0)	-	0 (0)	-	-	0.11 (0)	-	-
15 (Saltwater)	6 (1)	2005	0 (0)	-	-	0.26 (2)	-	-
16 (Freshwater)	0 (0)	-	0 (0)	-	-	0.11 (0)	-	-
17 (Supra-littoral rock)	0 (0)	-	0 (0)	-	-	0.08 (0)	-	-
18 (Supra-littoral sediment)	0 (0)	-	0 (0)	-	-	0.13 (0)	-	-
19 (Littoral rock)	0 (0)	-	0 (0)	-	-	0.12 (0)	-	-
20 (Littoral sediment)	0 (0)	-	0 (0)	-	-	0.13 (0)	-	-
21 (Saltmarsh)	0 (0)	-	0 (0)	-	-	-	-	-
22 (Urban)	0 (0)	-	0 (0)	-	-	0.18 (0)	-	-
23 (Suburban)	11 (5)	1992	0 (0)	-	-	0.29 (10)	-	-
Total	166 (38)	1994	0 (0)	-	-	0.17 (119)	-	-

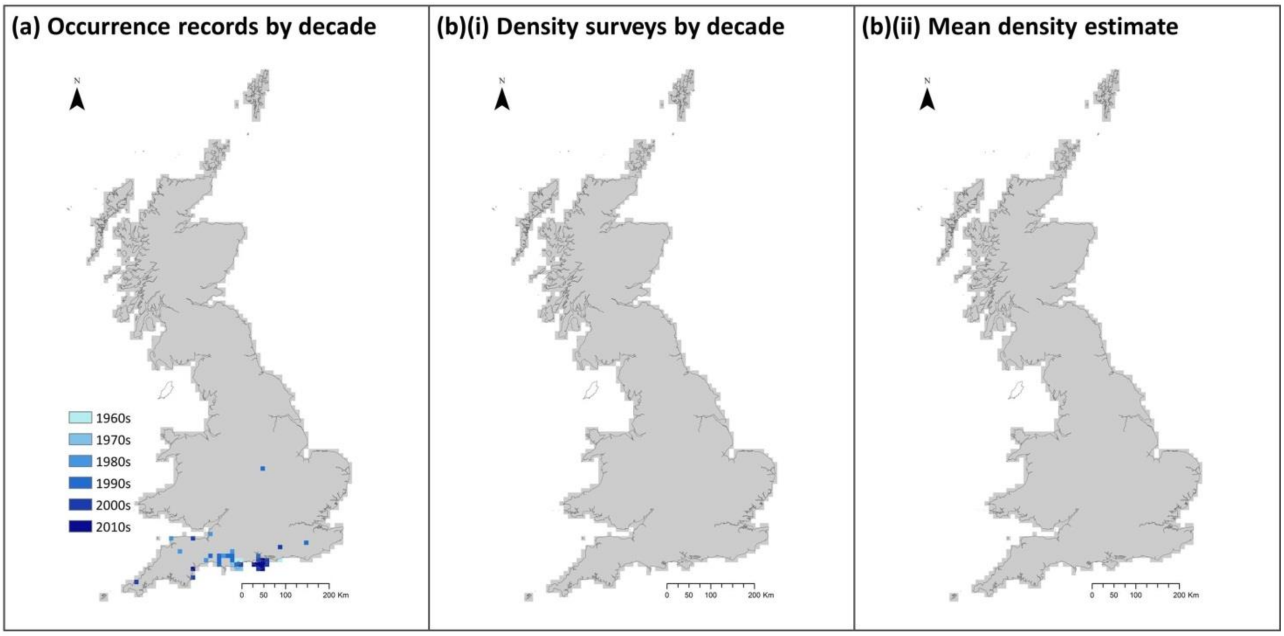

© Crown copyright and database rights 2016 Ordnance Survey 100051110. Data courtesy of the NBN Gateway with thanks to all data contributors. The NBN and its data contributors bear no responsibility for the further analysis or interpretation of this material, data and/or information.

Figure 1: 10km resolution raster maps based on BNG presenting the geographic description of available data. (a) shows the distribution of species occurrence obtained via the NBN Gateway categorised by the decade of last sighting. (b) shows information relating to density surveys identified via a search of published literature where: (i) categorises surveys by the decade of last survey; and (ii) shows the mean density estimate of surveys within grid cells (estimates assumed to be representative of entire cell, considered the upper limit of observed density).

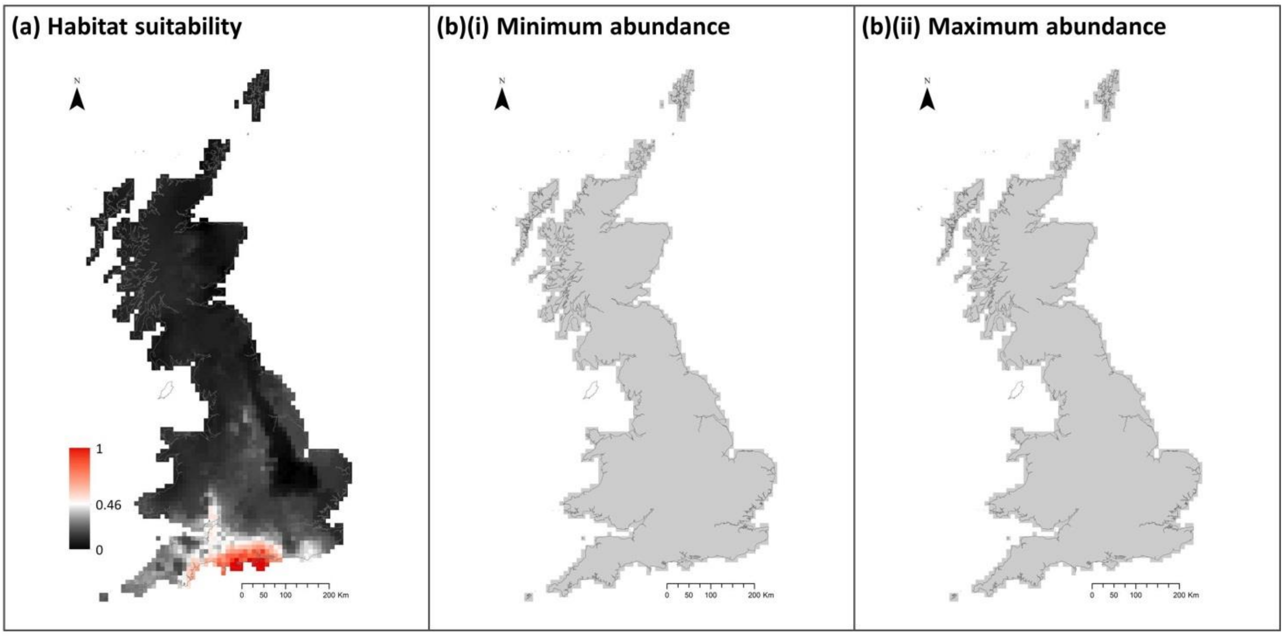

© Crown copyright and database rights 2016 Ordnance Survey 100051110. Data courtesy of the NBN Gateway with thanks to all data contributors. The NBN and its data contributors bear no responsibility for the further analysis or interpretation of this material, data and/or information.

Figure 2: Modelling predictions generated using systematic approach based on available data. (a) shows habitat suitability scores (the likelihood of observing the target species within each grid cell given variation environmental variables) determined by aggregating outputs from the "best" species distribution model (7 models compared) across 100 simulations. Here, the mid value on the scale denotes the threshold score above which occurrence is assumed. (b) shows: (i) the lower bound (Minimum); and (ii) the upper bound (Maximum); of abundance estimates determined by relating observed density (taking into account potential uncertainty) with habitat suitability scores using linear regression.
