# Supplementary material for: A systematic approach to estimate the distribution and total abundance of British mammals
Source: PLoS One. 2017 Jun 28;12(6):e0176339. doi: 10.1371/journal.pone.0176339 (PMC5489149; doi:10.1371/journal.pone.0176339)
Supplement: S5 File — Individual reports for each of the Chiroptera species presenting analysis of the available data and subsequent model predictions based on a 10km raster grid. Reports also include expert comment assessing the reliability (and plausibility) of results in the context of existing evidence and popular opinion. (ZIP) [file pone.0176339.s005.zip › I Leislers bat.pdf]

Leisler's bat (*Nyctalus leisleri*)

Order: *Chiroptera*

Genus: *Nyctalus*

Origin: Native

Status: Locally common

1995 abundance estimate: 10,000 (4)

Reported population trends: None

Data:

The available occurrence records indicate that Leisler's bat are distributed locally throughout England, notably in south eastern and central regions, with additional observations in the south west of Scotland and Wales (Figure 1a). These sightings were reported in a variety of landscape contexts (predominantly arable and improved grassland) with the majority (of cells) recorded at least once since 1995.

We identified a single study (Waters et al. 1999) conducted in 1994 at two locations in the south of England (Figure 1b) which report densities of Leisler's bats. Estimates ranged between 4.37 and 6.67 per km^2 with the highest densities recorded in habitats dominated by improved grassland (0.43 - 6.67 per km^2). Unfortunately, these sites only comprised of a limited selection of dominant land covers, consequently estimates were unavailable for some where occurrence was observed (marked grey in Table 1).

Model predictions:

The habitat suitability map (Figure 2a) appears to reflect the underlying data reasonably well with the set of "best" models predicting presence (and absence) to a mean AUC of 0.63. However, the resulting distribution is substantially larger than the area described by the observations (between 3 and 4 times). Overall, across 100 repetitions MaxEnt proved to be the most commonly selected modelling approach displaying the highest AUC 26% of the time followed closely by Domain (20%). By land cover the mean habitat suitability scores suggest observation is most likely in landscapes dominated by arable habitat (Table 1) and, consistent with this, the majority of occurrence is predicted in grid cells dominated by arable and improved grassland.

There was no relationship with habitat suitability, potentially due to the limited range of density estimates. Constant density values were therefore applied across all cells where occurrence was predicted. For maximum density spherical autocorrelation provided the best fit and was therefore accounted for in the modelling process.

As anticipated the predicted abundance range does not contain the estimate from Harris et al. (1995) instead suggesting a significant increase in the total population. This in part is due to the inflated distribution but may be exacerbated by the use of a limited number of high density estimates.

Reliability (Expert comment):

The geographic extent indicated by the observed occurrence records is plausible, as is the reported density estimate which was considered representative of the locations surveyed. However, it would be fair to suggest that they only capture the higher end of the possible range across a national scale.

The predicted occurrence appears representative of core distribution given the available records. It is difficult to further comment on the accuracy of the distribution as a definitive range for the species has not been established (perhaps due to its large home range). Despite this lack of knowledge it is probably reasonable to suggest that the maps of abundance display less variability than would be expected (Figure 2b).

The prediction of minimum abundance seems reasonable and it could be argued that biotic interactions with other bat species would produce a scenario favouring this lower bound of the range. Although little is known about the species it is likely that the true estimate of total abundance lies between this lower bound and the original estimate by Harris et al. (1995); the latter reflecting a slight underestimation.

References:

Harris, S. J., P. Morris, S. Wray and D. Yalden (1995). A review of British mammals: population estimates and conservation status of British mammals other than cetaceans, Joint Nature Conservation Committee, Peterborough, UK.

Waters, D., G. Jones and M. Furlong (1999). Foraging ecology of Leisler's bat (*Nyctalus leisleri*) at two sites in southern Britain. *Journal of Zoology* 249(2): 173-180.

Table 1: Summary of observed data and model predictions by land cover class (LCM2007 target classification). Values shown in brackets denote the spatial coverage based on a 10km resolution raster map (number of grid cells). Years represent the median of records within each land class. Ranges for density and abundance are derived using the respective minimum and maximum raster maps (lower bound is mean of values across minimum raster map with upper across the maximum) which capture the spatial uncertainty generate by projecting irregular polygons describing survey sites onto a raster grid.

LCM2007 class	Observed					Predicted		
	Occurrence		Density					
	Records	Year	Estimates	Year	Range	Habitat suitability	Density	Abundance
1 (Broadleaved woodland)	2 (1)	2006	0 (0)	-	-	0.36 (1)	0.44 - 5.14	43.74 - 513.7
2 (Coniferous woodland)	31 (9)	2010	0 (0)	-	-	0.22 (11)	0.42 - 4.94	462.6 - 5,433
3 (Arable and Horticultural)	274 (111)	2007	3 (3)	1994	0.18 - 4.37	0.47 (480)	0.43 - 5.02	20,515 - 240,920
4 (Improved grassland)	128 (57)	2007	1 (1)	1994	0.43 - 6.67	0.35 (189)	0.42 - 4.89	7,862 - 92,332
5 (Rough grassland)	0 (0)	-	0 (0)	-	-	0.1 (1)	0.44 - 5.14	43.74 - 513.7
6 (Neutral grassland)	0 (0)	-	0 (0)	-	-	0.05 (0)	-	0
7 (Calcareous grassland)	0 (0)	-	0 (0)	-	-	0.36 (0)	-	0
8 (Acid grassland)	3 (3)	2007	0 (0)	-	-	0.1 (0)	-	0
9 (Fen, Marsh, and Swamp)	0 (0)	-	0 (0)	-	-	-	-	0
10 (Heather)	1 (1)	1968	0 (0)	-	-	0.11 (0)	-	0
11 (Heather grassland)	1 (1)	2010	0 (0)	-	-	0.08 (0)	-	0
12 (Bog)	1 (1)	2010	0 (0)	-	-	0.08 (0)	-	0
13 (Montane habitat)	0 (0)	-	0 (0)	-	-	0.03 (0)	-	0
14 (Inland rock)	0 (0)	-	0 (0)	-	-	0.02 (0)	-	0
15 (Saltwater)	0 (0)	-	0 (0)	-	-	0.24 (0)	-	0
16 (Freshwater)	0 (0)	-	0 (0)	-	-	0.19 (0)	-	0
17 (Supra - littoral rock)	0 (0)	-	0 (0)	-	-	0.07 (0)	-	0
18 (Supra - littoral sediment)	0 (0)	-	0 (0)	-	-	0.11 (0)	-	0
19 (Littoral rock)	0 (0)	-	0 (0)	-	-	0.12 (0)	-	0
20 (Littoral sediment)	1 (1)	1961	0 (0)	-	-	0.22 (0)	-	0
21 (Saltmarsh)	0 (0)	-	0 (0)	-	-	-	-	0
22 (Urban)	2 (1)	2012	0 (0)	-	-	0.38 (3)	0.44 - 5.14	131.2 - 1,541
23 (Suburban)	23 (7)	1994	2 (2)	1994	0.83 - 5.52	0.41 (29)	0.43 - 5.11	1,261 - 14,814
Total	467 (193)	2007	6 (6)	1994	0.44 - 5.14	0.32 (714)	0.42 - 4.99	30,319 - 356,068

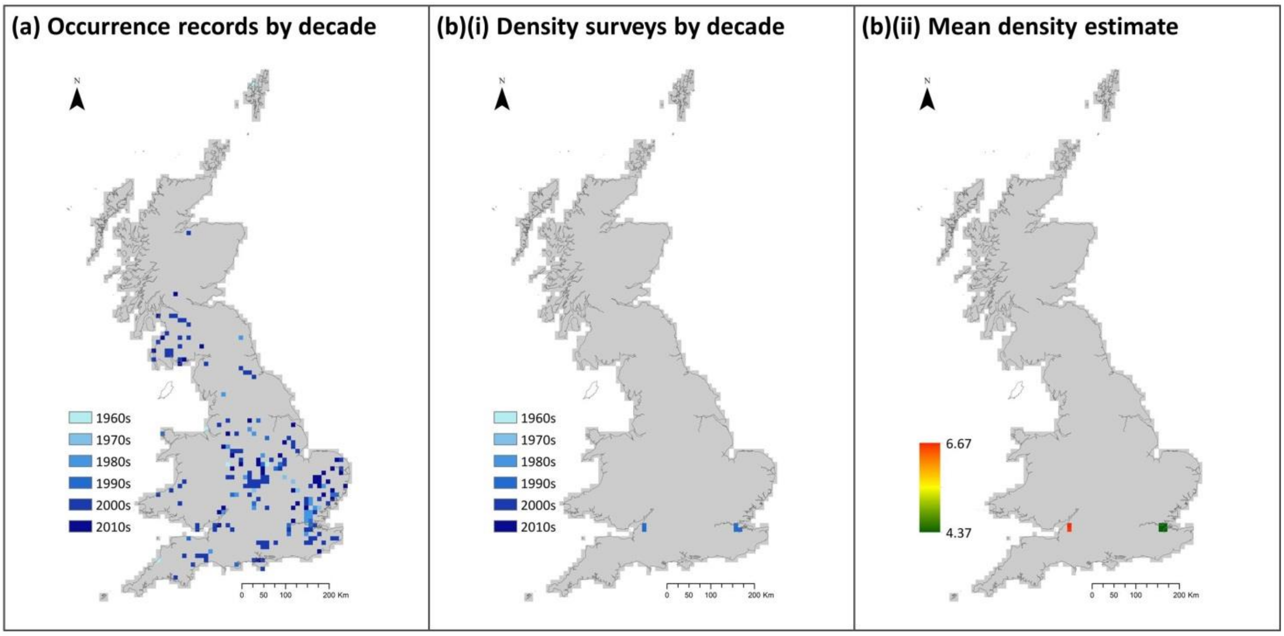

© Crown copyright and database rights 2016 Ordnance Survey 100051110. Data courtesy of the NBN Gateway with thanks to all data contributors. The NBN and its data contributors bear no responsibility for the further analysis or interpretation of this material, data and/or information.

Figure 1: 10km resolution raster maps based on BNG presenting the geographic description of available data. (a) shows the distribution of species occurrence obtained via the NBN Gateway categorised by the decade of last sighting. (b) shows information relating to density surveys identified via a search of published literature where: (i) categorises surveys by the decade of last survey; and (ii) shows the mean density estimate of surveys within grid cells (estimates assumed to be representative of entire cell, considered the upper limit of observed density).

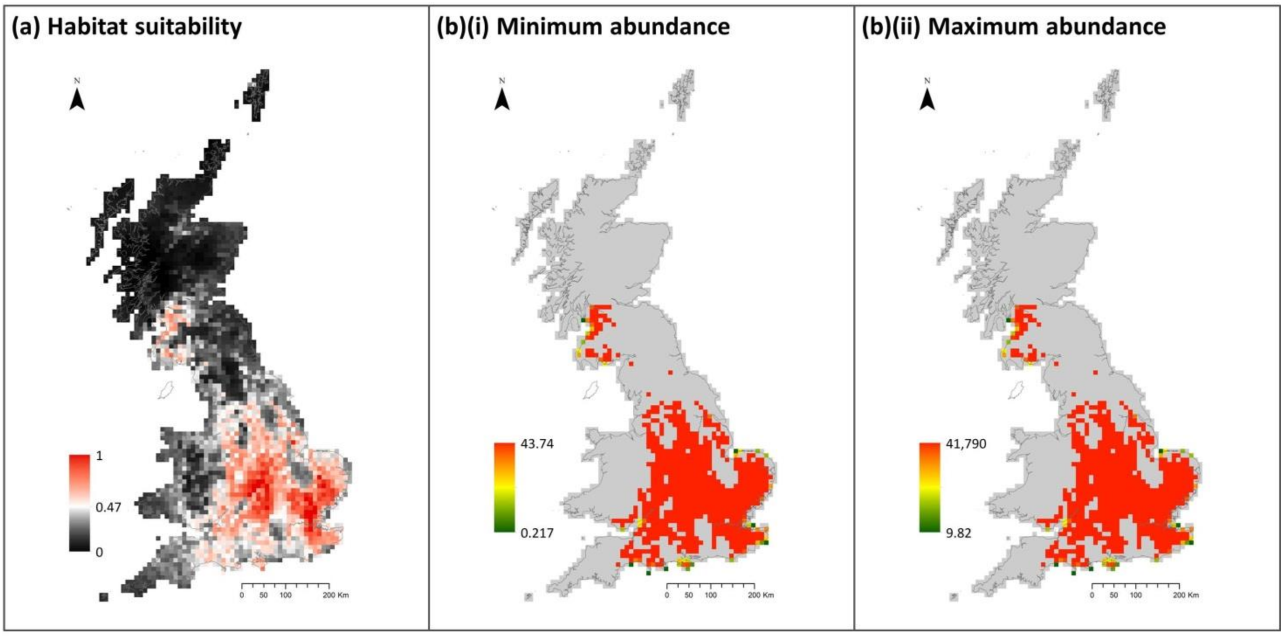

© Crown copyright and database rights 2016 Ordnance Survey 100051110. Data courtesy of the NBN Gateway with thanks to all data contributors. The NBN and its data contributors bear no responsibility for the further analysis or interpretation of this material, data and/or information.

Figure 2: Modelling predictions generated using systematic approach based on available data. (a) shows habitat suitability scores (the likelihood of observing the target species within each grid cell given variation environmental variables) determined by aggregating outputs from the "best" species distribution model (7 models compared) across 100 simulations. Here, the mid value on the scale denotes the threshold score above which occurrence is assumed. (b) shows: (i) the lower bound (Minimum); and (ii) the upper bound (Maximum); of abundance estimates determined by relating observed density (taking into account potential uncertainty) with habitat suitability scores using linear regression.
