# Supplementary material for: A systematic approach to estimate the distribution and total abundance of British mammals
Source: PLoS One. 2017 Jun 28;12(6):e0176339. doi: 10.1371/journal.pone.0176339 (PMC5489149; doi:10.1371/journal.pone.0176339)
Supplement: S5 File — Individual reports for each of the Chiroptera species presenting analysis of the available data and subsequent model predictions based on a 10km raster grid. Reports also include expert comment assessing the reliability (and plausibility) of results in the context of existing evidence and popular opinion. (ZIP) [file pone.0176339.s005.zip › K Nathusius pipistrelle.pdf]

Nathusius’ pipistrelle (*Pipistrellus nathusii*)

Order: *Chiroptera* **Genus:** *Pipistrellus*

Origin: Migrant **Status:** Rare

1995 abundance estimate: None (-) **Reported population trends:** None

Data:

The available occurrence records indicate that Nathusius’ pipistrelle are sparsely distributed throughout Britain with a trend for records towards the south east of England (Figure 1a). These sightings were reported in grid cells dominated by various habitats (predominantly arable); the majority within the last decade (2010 - 2013).

From the literature review we were unable to identify any publications reporting an estimate of density.

Model predictions:

The habitat suitability map (Figure 2a) appears to reflect the underlying data reasonably well with the set of “best” models predicting presence (and absence) to a mean AUC of 0.64. However, the resulting distribution is substantially larger than the area described by the observations (approximately 4 times). Overall, across 100 repetitions Random Forest proved to be the most commonly selected modelling approach displaying the highest AUC 30% of the time followed by Generalised Linear Models (24%), Support Vector Machine (20%) and MaxEnt (17%). By land cover the mean habitat suitability scores suggest observation is most likely in landscapes dominated by urban and suburban habitat (Table 1) but, consistent with recorded sightings, the majority of occurrence is predicted in grid cells dominated by arable and improved grassland. The analysis shows that occurrence is preserved in all habitats where it is observed with the exception of acid grassland where there are few records. Despite the expanded coverage of the predicted distribution the composition of habitats remain similar to that of the observed data increasing in relative proportion with the exception of heather and coniferous woodland which show a decrease.

Unfortunately, due to the lack of density estimates model analysis to predict abundance could not be performed.

Reliability (Expert comment):

The observed occurrence records appear to include a lot of auditory detections. The number of known maternity roosts is much smaller which makes inference about where breeding occurs and density of resident bats much harder, and in addition, as this species may be migratory, it is possible that the predicted distribution is biased by records of Scandinavian bats in transit along the eastern seaboard.

The predicted habitat associations for this species are unproven but are potentially influenced by the locations of bat recoveries (grounded bats); which are more likely to be found near human populations and may be confounded by the considerable distances this species is known to move during their nightly foraging.

References:

None

Table 1: Summary of observed data and model predictions by land cover class (LCM2007 target classification). Values shown in brackets denote the spatial coverage based on a 10km resolution raster map (number of grid cells). Years represent the median of records within each land class. Ranges for density and abundance are derived using the respective minimum and maximum raster maps (lower bound is mean of values across minimum raster map with upper across the maximum) which capture the spatial uncertainty generate by projecting irregular polygons describing survey sites onto a raster grid.

LCM2007 class	Observed					Predicted		
	Occurrence		Density					
	Records	Year	Estimates	Year	Range	Habitat suitability	Density	Abundance
1 (Broadleaved woodland)	2 (1)	2013	0 (0)	-	-	0.48 (5)	-	-
2 (Coniferous woodland)	4 (2)	2010	0 (0)	-	-	0.17 (1)	-	-
3 (Arable and Horticultural)	416 (121)	2011	0 (0)	-	-	0.52 (514)	-	-
4 (Improved grassland)	153 (53)	2010	0 (0)	-	-	0.37 (157)	-	-
5 (Rough grassland)	11 (5)	1998	0 (0)	-	-	0.27 (14)	-	-
6 (Neutral grassland)	0 (0)	-	0 (0)	-	-	0.36 (0)	-	-
7 (Calcareous grassland)	0 (0)	-	0 (0)	-	-	0.31 (0)	-	-
8 (Acid grassland)	9 (2)	1999	0 (0)	-	-	0.12 (0)	-	-
9 (Fen, Marsh, and Swamp)	0 (0)	-	0 (0)	-	-	-	-	-
10 (Heather)	2 (2)	2000	0 (0)	-	-	0.2 (1)	-	-
11 (Heather grassland)	4 (3)	1996	0 (0)	-	-	0.15 (9)	-	-
12 (Bog)	4 (4)	2004	0 (0)	-	-	0.19 (11)	-	-
13 (Montane habitat)	0 (0)	-	0 (0)	-	-	0.08 (0)	-	-
14 (Inland rock)	0 (0)	-	0 (0)	-	-	0.03 (0)	-	-
15 (Saltwater)	0 (0)	-	0 (0)	-	-	0.42 (4)	-	-
16 (Freshwater)	0 (0)	-	0 (0)	-	-	0.21 (0)	-	-
17 (Supra-littoral rock)	0 (0)	-	0 (0)	-	-	0.13 (0)	-	-
18 (Supra-littoral sediment)	0 (0)	-	0 (0)	-	-	0.29 (1)	-	-
19 (Littoral rock)	0 (0)	-	0 (0)	-	-	0.26 (3)	-	-
20 (Littoral sediment)	2 (2)	2004	0 (0)	-	-	0.41 (8)	-	-
21 (Saltmarsh)	0 (0)	-	0 (0)	-	-	-	-	-
22 (Urban)	15 (3)	2012	0 (0)	-	-	0.74 (6)	-	-
23 (Suburban)	36 (16)	2011	0 (0)	-	-	0.71 (74)	-	-
Total	658 (214)	2010	0 (0)	-	-	0.37 (808)	-	-

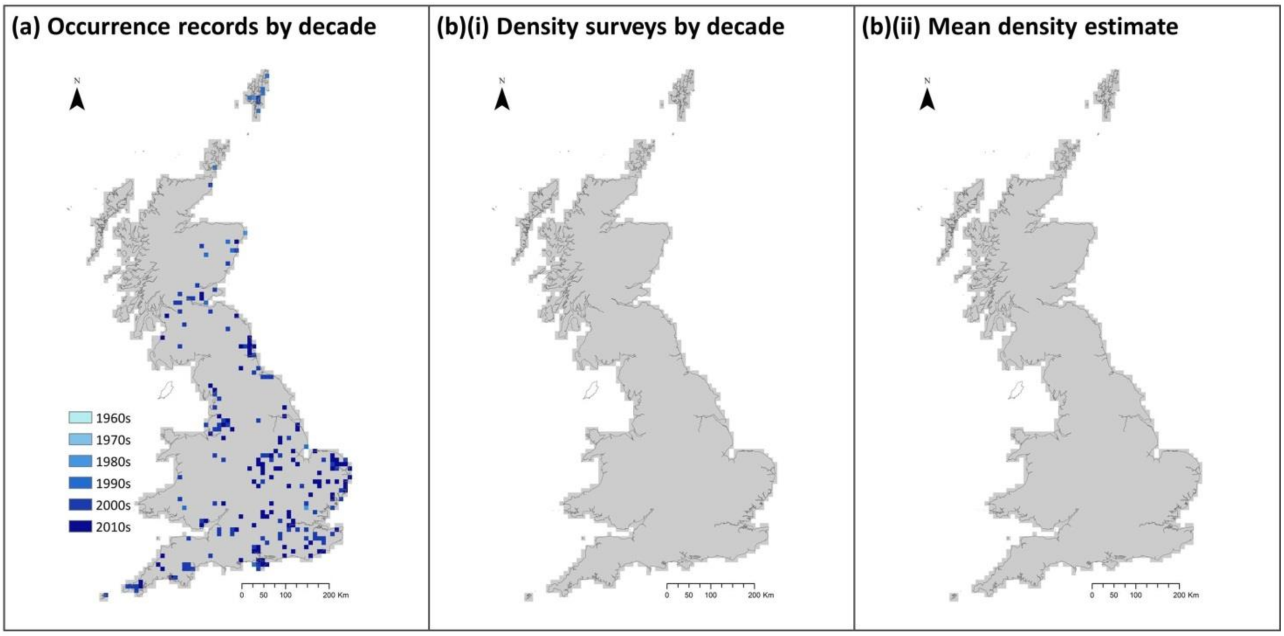

© Crown copyright and database rights 2016 Ordnance Survey 100051110. Data courtesy of the NBN Gateway with thanks to all data contributors. The NBN and its data contributors bear no responsibility for the further analysis or interpretation of this material, data and/or information.

Figure 1: 10km resolution raster maps based on BNG presenting the geographic description of available data. (a) shows the distribution of species occurrence obtained via the NBN Gateway categorised by the decade of last sighting. (b) shows information relating to density surveys identified via a search of published literature where: (i) categorises surveys by the decade of last survey; and (ii) shows the mean density estimate of surveys within grid cells (estimates assumed to be representative of entire cell, considered the upper limit of observed density).

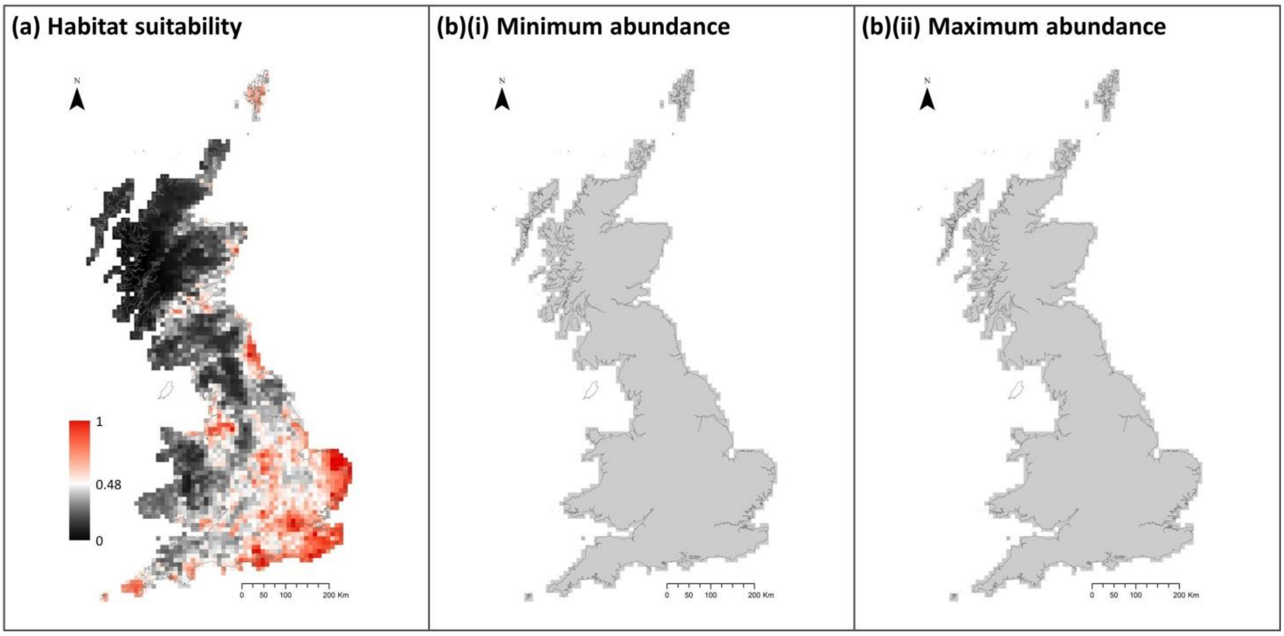

© Crown copyright and database rights 2016 Ordnance Survey 100051110. Data courtesy of the NBN Gateway with thanks to all data contributors. The NBN and its data contributors bear no responsibility for the further analysis or interpretation of this material, data and/or information.

Figure 2: Modelling predictions generated using systematic approach based on available data. (a) shows habitat suitability scores (the likelihood of observing the target species within each grid cell given variation environmental variables) determined by aggregating outputs from the "best" species distribution model (7 models compared) across 100 simulations. Here, the mid value on the scale denotes the threshold score above which occurrence is assumed. (b) shows: (i) the lower bound (Minimum); and (ii) the upper bound (Maximum); of abundance estimates determined by relating observed density (taking into account potential uncertainty) with habitat suitability scores using linear regression.
