# Supplementary material for: A systematic approach to estimate the distribution and total abundance of British mammals
Source: PLoS One. 2017 Jun 28;12(6):e0176339. doi: 10.1371/journal.pone.0176339 (PMC5489149; doi:10.1371/journal.pone.0176339)
Supplement: S5 File — Individual reports for each of the Chiroptera species presenting analysis of the available data and subsequent model predictions based on a 10km raster grid. Reports also include expert comment assessing the reliability (and plausibility) of results in the context of existing evidence and popular opinion. (ZIP) [file pone.0176339.s005.zip › L Natterers bat.pdf]

Natterer's bat (*Myotis nattereri*)

Order: *Chiroptera*

Genus: *Myotis*

Origin: Native

Status: Common

1995 abundance estimate: 100,000 (4)

Reported population trends: JNCC 2005, BCT 2014 (↑)

Data:

Natterer's bat are widely distributed throughout England and Wales with a notable absence in the north west of Scotland (Figure 1a). It is not clear if areas of poor reporting indicate a lack of observations and records, or a genuine absence of the species. Observations were reported in grid cells dominated by various habitats (predominantly arable and improved grassland) with the occupancy of the majority of cells validated by at least one record since 1995.

From the literature review we identified two surveys (Fairless 2013; Jones et al. 1996) conducted in northern England and north east Scotland between 1987 and 1990 (Figure 1b). Estimates ranged between 1.8 and 24.3 per km^2 with the highest densities recorded in habitats dominated by improved grassland (0.48 - 10.23 per km^2 accounting for uncertainty relating to unsurveyed areas within grid cells). Unfortunately, these surveys only sampled a limited selection of dominant land covers (arable and improved grassland), consequently estimates were unavailable for some habitats where occurrence was observed (marked grey in Table 1).

Model predictions:

The habitat suitability map (Figure 2a) appears to reflect the underlying data reasonably well with the set of "best" models predicting presence (and absence) to a mean AUC of 0.69. Overall, across 100 repetitions Random Forest proved to be the most commonly selected modelling approach displaying the highest AUC 36% of the time closely followed by MaxEnt (35%). By land cover the mean habitat suitability scores suggest observation is most likely in landscapes dominated by arable and broadleaved woodland (Table 1) but, consistent with recorded sightings, the majority of occurrence is predicted in grid cells dominated by arable and improved grassland.

Neither minimum nor maximum density estimates showed a correlation with predicted habitat suitability, possibly due to the limited number of density estimates. However, maximum density suggested a best fit using spherical spatial autocorrelation.

Despite this the predicted abundance range contains the estimate from Harris et al. (1995) suggesting no significant change in the total population (as the 1995 estimate was based on similar density surveys this is perhaps unsurprising; the result may instead indicate that there have been no significant changes in the species distribution over the past 20 years). There is scope within the range to accommodate an increase in population, as suggested by recent trends, but this cannot be considered statistically significant (i.e. taking into uncertainties relating to the available data and modelling process).

Reliability (Expert comment):

The observed occurrence records do not show any obvious biases including the reduced number of sightings in Scotland which is consistent with this species' reduced range (relative to *M. daubentonii*) elsewhere in Europe. As for density, the upper estimate obtained from the literature is implausible on a 10km scale but may be true across smaller areas.

The map of habitat suitability (Figure 2a) reflects the observed data but the maps of predicted abundance demonstrate an inappropriate lack of variability (Figure 2b). The Harris estimate from 1995 represents a reasonable ballpark figure for the time and there is no evidence to suggest a significant change in population. In keeping with this the lower bound of the range is a more appropriate estimate compared with the upper end which is unrealistic. This is perhaps a reflection of the previous comment regarding the applicability of the upper density estimate.

References:

Fairless, L. (2013). Ecology and ecophysiology of social structure and population dynamics in bats (*Vespertilionidae*). Ph.D. Thesis, University of Southampton.

Harris, S. J., P. Morris, S. Wray and D. Yalden (1995). A review of British mammals: population estimates and conservation status of British mammals other than cetaceans, Joint Nature Conservation Committee, Peterborough, UK.

Jones, K. E., J. D. Altringham and R. Deaton (1996). Distribution and population densities of seven species of bat in northern England. *Journal of Zoology* 240(4): 788-798.

Table 1: Summary of observed data and model predictions by land cover class (LCM2007 target classification). Values shown in brackets denote the spatial coverage based on a 10km resolution raster map (number of grid cells). Years represent the median of records within each land class. Ranges for density and abundance are derived using the respective minimum and maximum raster maps (lower bound is mean of values across minimum raster map with upper across the maximum) which capture the spatial uncertainty generate by projecting irregular polygons describing survey sites onto a raster grid.

LCM2007 class	Observed					Predicted		
	Occurrence		Density					
	Records	Year	Estimates	Year	Range	Habitat suitability	Density	Abundance
1 (Broadleaved woodland)	75 (7)	2010	0 (0)	-	-	0.85 (8)	0.58 - 13.0	466.7 - 10,416
2 (Coniferous woodland)	413 (39)	2001	0 (0)	-	-	0.54 (24)	0.58 - 13.0	1,395 - 31,136
3 (Arable and Horticultural)	7,319 (532)	2010	4 (4)	1990	0.79 - 7.42	0.83 (736)	0.55 - 12.2	40,229 - 897,978
4 (Improved grassland)	4,945 (397)	2008	8 (8)	1990	0.48 - 10.2	0.77 (537)	0.55 - 12.2	29,451 - 657,393
5 (Rough grassland)	46 (6)	2006	0 (0)	-	-	0.23 (5)	0.58 - 12.9	289.5 - 6,461
6 (Neutral grassland)	0 (0)	-	0 (0)	-	-	0 (0)	-	0
7 (Calcareous grassland)	1 (1)	1987	0 (0)	-	-	0.77 (1)	0.58 - 13.0	58.33 - 1,302
8 (Acid grassland)	403 (52)	2003	0 (0)	-	-	0.49 (51)	0.58 - 13.0	2,967 - 66,232
9 (Fen, Marsh, and Swamp)	0 (0)	-	0 (0)	-	-	-	-	0
10 (Heather)	6 (4)	1993	0 (0)	-	-	0.37 (0)	-	0
11 (Heather grassland)	21 (12)	1996	0 (0)	-	-	0.25 (6)	0.58 - 13.0	350 - 7,812
12 (Bog)	41 (12)	2005	0 (0)	-	-	0.22 (11)	0.58 - 13.0	641.6 - 14,322
13 (Montane habitat)	23 (6)	2002	0 (0)	-	-	0.39 (0)	-	0
14 (Inland rock)	0 (0)	-	0 (0)	-	-	0.07 (0)	-	0
15 (Saltwater)	10 (2)	2004	0 (0)	-	-	0.56 (0)	-	0
16 (Freshwater)	1 (1)	2012	0 (0)	-	-	0.46 (0)	-	0
17 (Supra - littoral rock)	0 (0)	-	0 (0)	-	-	0.03 (0)	-	0
18 (Supra - littoral sediment)	0 (0)	-	0 (0)	-	-	0.26 (0)	-	0
19 (Littoral rock)	0 (0)	-	0 (0)	-	-	0.21 (0)	-	0
20 (Littoral sediment)	68 (7)	2010	0 (0)	-	-	0.55 (1)	0.45 - 10.0	44.92 - 1,003
21 (Saltmarsh)	0 (0)	-	0 (0)	-	-	-	-	0
22 (Urban)	26 (1)	2013	0 (0)	-	-	0.46 (0)	-	0
23 (Suburban)	205 (24)	2006	0 (0)	-	-	0.64 (12)	0.58 - 13.0	700 - 15,624
Total	13,603 (1,103)	2009	12 (12)	1990	0.58 - 9.3	0.66 (1,392)	0.55 - 12.3	76,593 - 1,709,679

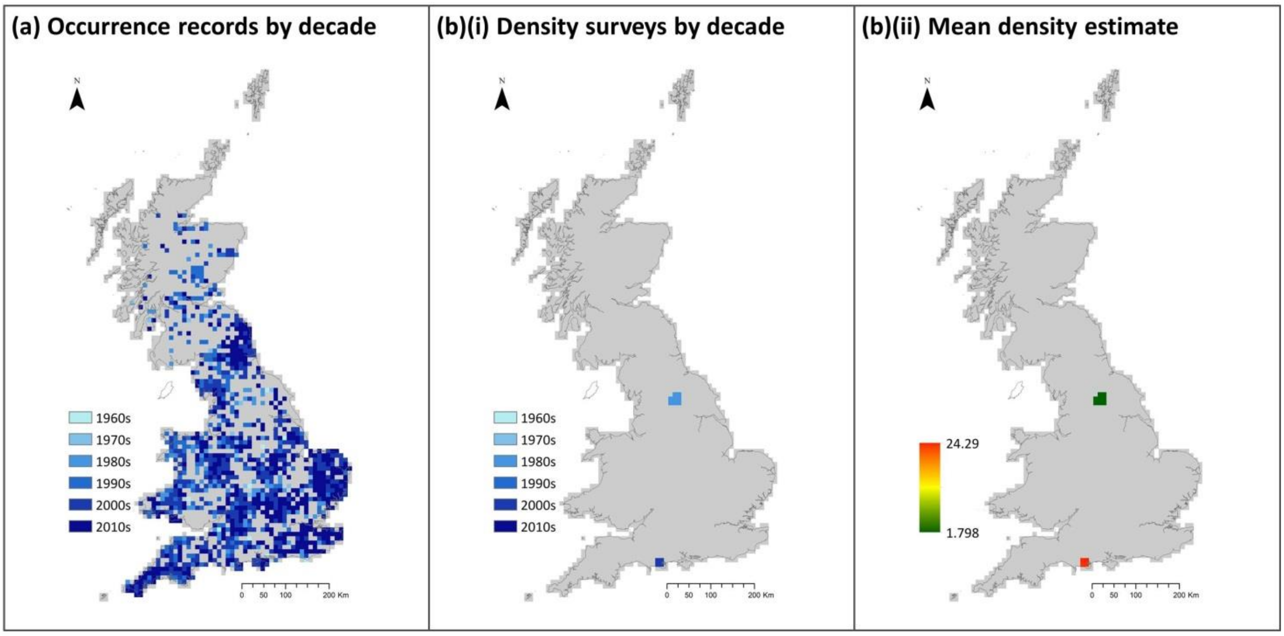

© Crown copyright and database rights 2016 Ordnance Survey 100051110. Data courtesy of the NBN Gateway with thanks to all data contributors. The NBN and its data contributors bear no responsibility for the further analysis or interpretation of this material, data and/or information.

Figure 1: 10km resolution raster maps based on BNG presenting the geographic description of available data. (a) shows the distribution of species occurrence obtained via the NBN Gateway categorised by the decade of last sighting. (b) shows information relating to density surveys identified via a search of published literature where: (i) categorises surveys by the decade of last survey; and (ii) shows the mean density estimate of surveys within grid cells (estimates assumed to be representative of entire cell, considered the upper limit of observed density).

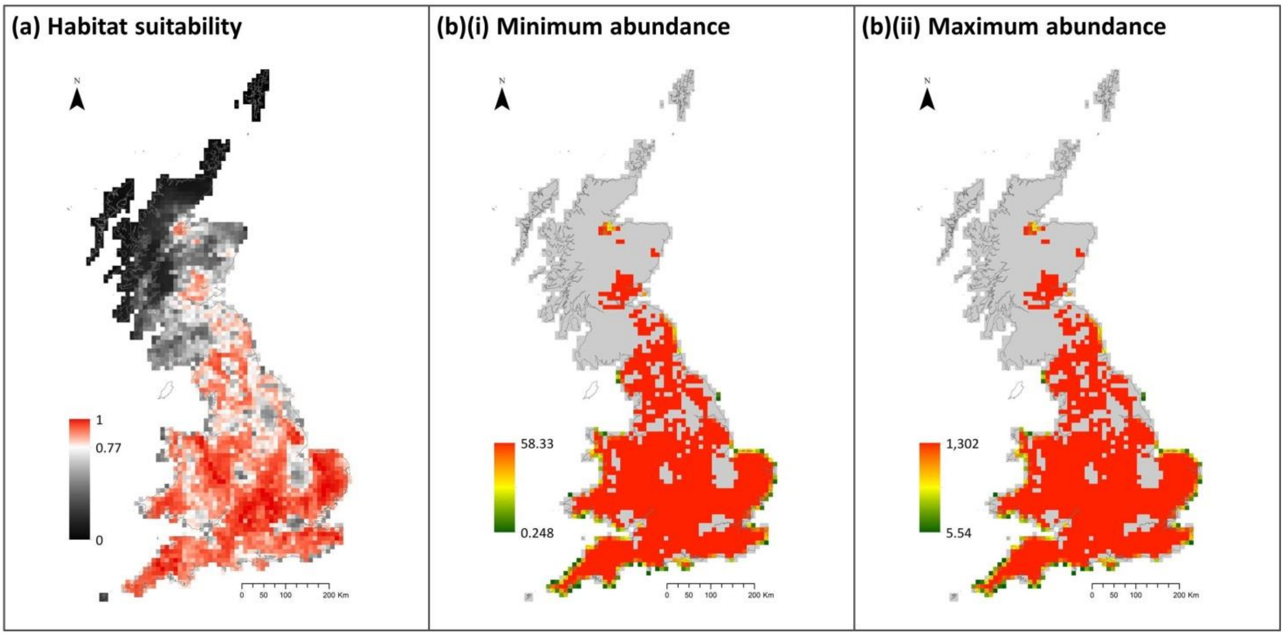

© Crown copyright and database rights 2016 Ordnance Survey 100051110. Data courtesy of the NBN Gateway with thanks to all data contributors. The NBN and its data contributors bear no responsibility for the further analysis or interpretation of this material, data and/or information.

Figure 2: Modelling predictions generated using systematic approach based on available data. (a) shows habitat suitability scores (the likelihood of observing the target species within each grid cell given variation environmental variables) determined by aggregating outputs from the "best" species distribution model (7 models compared) across 100 simulations. Here, the mid value on the scale denotes the threshold score above which occurrence is assumed. (b) shows: (i) the lower bound (Minimum); and (ii) the upper bound (Maximum); of abundance estimates determined by relating observed density (taking into account potential uncertainty) with habitat suitability scores using linear regression.
