# Supplementary material for: A systematic approach to estimate the distribution and total abundance of British mammals
Source: PLoS One. 2017 Jun 28;12(6):e0176339. doi: 10.1371/journal.pone.0176339 (PMC5489149; doi:10.1371/journal.pone.0176339)
Supplement: S5 File — Individual reports for each of the Chiroptera species presenting analysis of the available data and subsequent model predictions based on a 10km raster grid. Reports also include expert comment assessing the reliability (and plausibility) of results in the context of existing evidence and popular opinion. (ZIP) [file pone.0176339.s005.zip › M Noctule.pdf]

Noctule (*Nyctalus noctula*)

Order: *Chiroptera*

Genus: *Nyctalus*

Origin: Native

Status: Common

1995 abundance estimate: 50,000 (3)

Reported population trends: JNCC 2005, BCT 2014 (↔)

Data:

The noctule is widely distributed throughout England and Wales with few records in Scotland (Figure 1a). These sightings were reported in various habitats (predominantly arable and improved grassland) with the majority of cells containing at least one record since 1995.

From the literature review we identified a single survey (Jones et al. 1996) conducted in northern England in 1990 which estimated density to be approximately 1.5 per km^2 (Figure 1b). This survey only sampled habitats dominated by arable and improved grassland, consequently no estimates were available for other habitats where occurrence was observed (marked grey in Table 1).

Model predictions:

The habitat suitability map (Figure 2a) appears to reflect the underlying data reasonably well with the set of "best" models predicting presence (and absence) to a mean AUC of 0.69. Overall, across 100 repetitions Random Forest proved to be the most commonly selected modelling approach displaying the highest AUC 25% of the time followed by MaxEnt (23%) and Generalised Linear Models (23%). By land cover the mean habitat suitability scores suggest observation is most likely in landscapes dominated by urban and broadleaved woodland (Table 1) but, consistent with recorded sightings, the majority of occurrence is predicted in grid cells dominated by arable and improved grassland. The analysis shows that occurrence is preserved in all land covers where it is observed with the exception of heather, montane and saltwater dominated habitat.

Linear regression suggested that there was no correlation between the estimates of maximum density and habitat suitability; consequently, it was applied as a constant in cells where occurrence is predicted. Minimum density was found to be correlated with the best fit model relating habitat suitability linearly using a GLM with a gamma distribution.

The predicted abundance range contains the estimate from Harris et al. (1995) suggesting, in agreement with recent trend analysis, no significant change in the total population (as both estimates are based on the similar density surveys this is perhaps unsurprising; the result could instead be considered an indication that there have been no significant changes in the species distribution over the past 20 years).

Reliability (Expert comment):

The observed sightings records underrepresent occurrence in Scotland; as therefore does the predicted habitat suitability map (Figure 2a). The density estimate obtained from the literature is suspected to be plausible for the species and may represent the mid-range of likely estimates. It should be noted however that gauging density for such a wide ranging species even at a 10km scale is complicated, particularly, rationalising the differences between roosting and foraging.

The predicted range of abundances is plausible with scope to suggest a larger population than that of the original Harris estimate; which could be considered an underestimation (the estimate is only given a moderate reliability of 3 out of 5). The location of high abundance areas are interesting perhaps indicating a strong association with the warmer (July average) arable landscapes which suggests further investigation.

References:

Harris, S. J., P. Morris, S. Wray and D. Yalden (1995). A review of British mammals: population estimates and conservation status of British mammals other than cetaceans, Joint Nature Conservation Committee, Peterborough, UK.

Jones, K. E., J. D. Altringham and R. Deaton (1996). Distribution and population densities of seven species of bat in northern England. *Journal of Zoology* 240(4): 788-798.

Table 1: Summary of observed data and model predictions by land cover class (LCM2007 target classification). Values shown in brackets denote the spatial coverage based on a 10km resolution raster map (number of grid cells). Years represent the median of records within each land class. Ranges for density and abundance are derived using the respective minimum and maximum raster maps (lower bound is mean of values across minimum raster map with upper across the maximum) which capture the spatial uncertainty generate by projecting irregular polygons describing survey sites onto a raster grid.

LCM2007 class	Observed					Predicted		
	Occurrence		Density					
	Records	Year	Estimates	Year	Range	Habitat suitability	Density	Abundance
1 (Broadleaved woodland)	57 (8)	2012	0 (0)	-	-	0.88 (10)	0.14 - 1.5	140.6 - 1,500
2 (Coniferous woodland)	106 (9)	2006	0 (0)	-	-	0.26 (3)	0.06 - 1.5	16.65 - 450
3 (Arable and Horticultural)	6,055 (589)	2011	5 (5)	1990	0.2 - 1.5	0.86 (790)	0.21 - 1.39	16,691 - 109,879
4 (Improved grassland)	3,241 (402)	2011	3 (3)	1990	0.42 - 1.5	0.77 (482)	0.18 - 1.39	8,844 - 66,992
5 (Rough grassland)	25 (8)	1999	0 (0)	-	-	0.26 (3)	0.1 - 1.5	29.61 - 449.8
6 (Neutral grassland)	0 (0)	-	0 (0)	-	-	0 (0)	-	0
7 (Calcareous grassland)	5 (1)	2014	0 (0)	-	-	0.84 (2)	0.02 - 1.5	4.11 - 300
8 (Acid grassland)	101 (24)	2008	0 (0)	-	-	0.3 (9)	0.06 - 1.48	52.02 - 1,330
9 (Fen, Marsh, and Swamp)	0 (0)	-	0 (0)	-	-	-	-	0
10 (Heather)	1 (1)	2014	0 (0)	-	-	0.21 (0)	-	0
11 (Heather grassland)	58 (3)	2014	0 (0)	-	-	0.15 (1)	0 - 1.5	0 - 150
12 (Bog)	28 (6)	2008	0 (0)	-	-	0.2 (4)	0.01 - 1.5	5.98 - 600
13 (Montane habitat)	2 (1)	1998	0 (0)	-	-	0.09 (0)	-	0
14 (Inland rock)	0 (0)	-	0 (0)	-	-	0.01 (0)	-	0
15 (Saltwater)	3 (1)	2012	0 (0)	-	-	0.56 (0)	-	0
16 (Freshwater)	0 (0)	-	0 (0)	-	-	0.14 (0)	-	0
17 (Supra - littoral rock)	0 (0)	-	0 (0)	-	-	0.07 (0)	-	0
18 (Supra - littoral sediment)	0 (0)	-	0 (0)	-	-	0.34 (0)	-	0
19 (Littoral rock)	0 (0)	-	0 (0)	-	-	0.23 (0)	-	0
20 (Littoral sediment)	50 (11)	2005	0 (0)	-	-	0.69 (1)	0 - 0.47	0 - 46.83
21 (Saltmarsh)	0 (0)	-	0 (0)	-	-	-	-	0
22 (Urban)	44 (7)	2012	0 (0)	-	-	0.9 (8)	0.14 - 1.01	113.7 - 810.6
23 (Suburban)	785 (55)	2012	0 (0)	-	-	0.83 (61)	0.05 - 1.33	284.2 - 8,102
Total	10,561 (1,126)	2011	8 (8)	1990	0.28 - 1.5	0.63 (1,374)	0.19 - 1.39	26,182 - 190,611

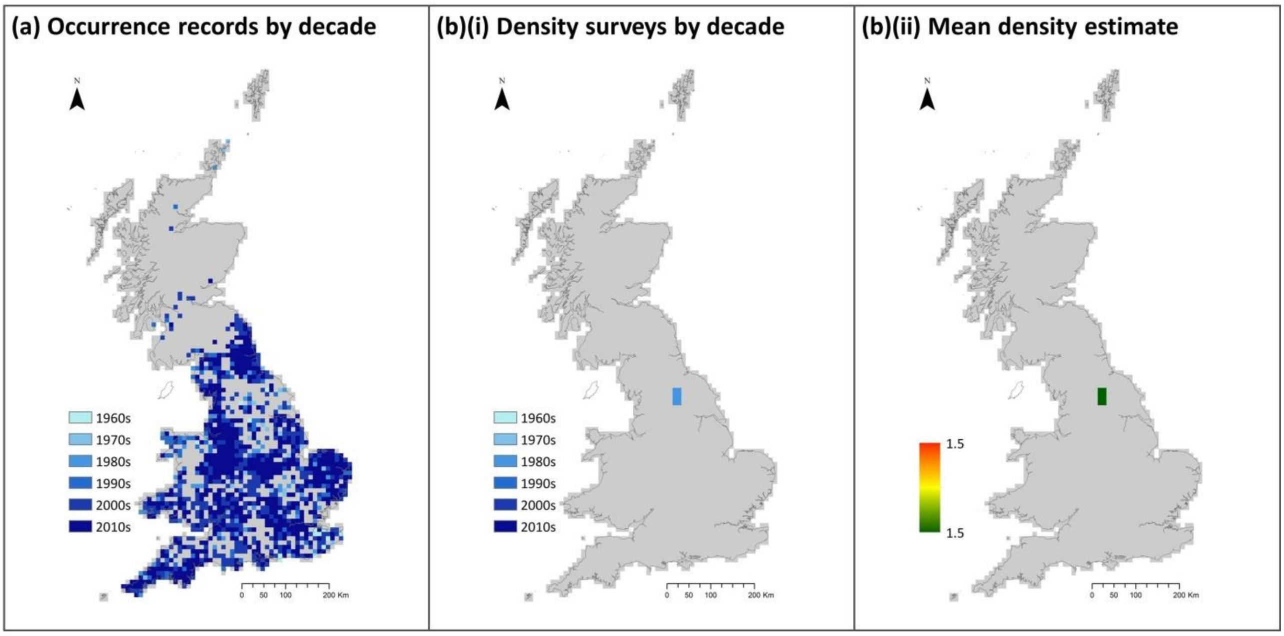

© Crown copyright and database rights 2016 Ordnance Survey 100051110. Data courtesy of the NBN Gateway with thanks to all data contributors. The NBN and its data contributors bear no responsibility for the further analysis or interpretation of this material, data and/or information.

Figure 1: 10km resolution raster maps based on BNG presenting the geographic description of available data. (a) shows the distribution of species occurrence obtained via the NBN Gateway categorised by the decade of last sighting. (b) shows information relating to density surveys identified via a search of published literature where: (i) categorises surveys by the decade of last survey; and (ii) shows the mean density estimate of surveys within grid cells (estimates assumed to be representative of entire cell, considered the upper limit of observed density).

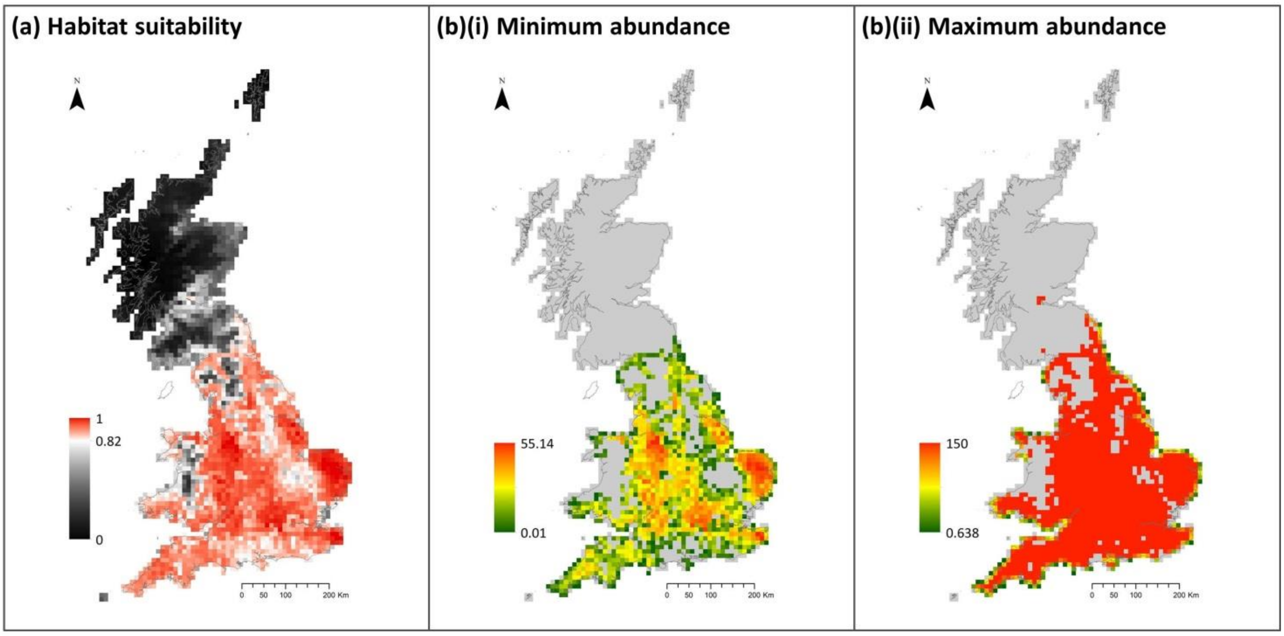

© Crown copyright and database rights 2016 Ordnance Survey 100051110. Data courtesy of the NBN Gateway with thanks to all data contributors. The NBN and its data contributors bear no responsibility for the further analysis or interpretation of this material, data and/or information.

Figure 2: Modelling predictions generated using systematic approach based on available data. (a) shows habitat suitability scores (the likelihood of observing the target species within each grid cell given variation environmental variables) determined by aggregating outputs from the "best" species distribution model (7 models compared) across 100 simulations. Here, the mid value on the scale denotes the threshold score above which occurrence is assumed. (b) shows: (i) the lower bound (Minimum); and (ii) the upper bound (Maximum); of abundance estimates determined by relating observed density (taking into account potential uncertainty) with habitat suitability scores using linear regression.
