# Supplementary material for: A systematic approach to estimate the distribution and total abundance of British mammals
Source: PLoS One. 2017 Jun 28;12(6):e0176339. doi: 10.1371/journal.pone.0176339 (PMC5489149; doi:10.1371/journal.pone.0176339)
Supplement: S5 File — Individual reports for each of the Chiroptera species presenting analysis of the available data and subsequent model predictions based on a 10km raster grid. Reports also include expert comment assessing the reliability (and plausibility) of results in the context of existing evidence and popular opinion. (ZIP) [file pone.0176339.s005.zip › N Pipistrelle.pdf]

Pipistrelle (*Pipistrellus pipistrellus sensu lato / Pipistrellus pygmaeus*)

Order: *Chiroptera*

Genus: *Pipistrellus*

Origin: Native

Status: Common

1995 abundance estimate: 2,000,000 (3)

Reported population trends: JNCC 2005, BCT 2014 (↑)

Data:

Together, common and soprano pipistrelle are ubiquitous across Britain with sightings reported in more than half of 10 km squares at least once (approximately 69%), most over the past decade (Figure 1a). However, the map highlights several areas, particularly in Scotland, where the species has not been reported for some time, or not at all.

Density estimates, recorded over the past three decades, were obtained from published literature (Fairless 2013; Jones et al. 1996; Speakman et al. 1991) spanning approximately 3% of the observed species distribution based on the available occurrence data (Figure 1b). Estimates ranged between 2.89 and 18.23 per km^2 with the highest densities recorded in habitat dominated by coniferous woodland (8.87 - 18.21 per km^2 accounting for uncertainty relating to unsurveyed areas within grid cells). In many of the land covers where occurrence was observed a suitable density estimate was not available (land class marked grey in Table 1).

Model predictions:

Whilst the habitat suitability map predicts that the core range of the two small pipistrelle species is less extensive than the observations suggest (Figure 2a) it appears a robust and plausible description of the underlying data with the set of "best" models predicting presence (and absence) to a mean AUC of 0.76. Overall, across 100 repetitions MaxEnt proved to be the most commonly selected modelling approach displaying the highest AUC 46% of the time followed by Random Forest (26%) and Generalised Linear Models (20%). By land cover the mean habitat suitability scores suggest observation is most likely in landscapes dominated by urban and calcareous grassland (Table 1) but, consistent with recorded sightings, the majority of occurrence is predicted in grid cells dominated by arable and improved grassland. Interestingly, for a significant number of land covers where it is observed occurrence is not preserved in predictions.

Both minimum and maximum density estimates were best fitted to the square of habitat suitability accounting for spherical spatial autocorrelation. However, particularly in urban areas, the relationship with minimum density appears to shows some indications of a negative correlation (this may occur as the values attributed to urban areas lie at a functional minimum with the values either side predicting higher densities) .

The predicted abundance range does not contain the estimate from Harris et al. (1995) and, contrary to recent trends, suggests a decrease in total population. Whilst this result is difficult to explain the underestimation may be due to a slight contraction of the distribution (2%) coupled with the limited availability of density estimates across land covers (although the estimates themselves cover a reasonable temporal period both pre and post 1995). Given the ubiquity of the species it may be reasonable to argue that densities provided for partially surveyed squares are representative of the whole and therefore that the true population estimate lies towards the top end of the predicted range. This would at least limit the decrease to approximately 8% since 1995.

Reliability (Expert comment):

Together the pipistrelle species are ubiquitous species in Britain. Whilst the observed sightings are consistent with this records from the NBN appear to contain bias reflecting human density and activity. This is most apparent in Scotland where occurrence is probably substantially underrepresented. The density estimates obtained from the literature appear to capture some of the range of expected densities but may be conservative at both low and high ends. Given the information presented it is not possible to comment on the spatial positioning of estimates as in this case local contexts (roost and foraging) are more important than climate and geography.

Before commenting on the model prediction it is important to highlight that several factors confound simple quantitative processes and inference for combined pipistrelle species. This complex includes two species with regional variations in dominance and the potential for grossly different estimates of density (soprano are considered more dominant in Scotland and show substantially larger roost sizes which may affect density estimates). It is unlikely that the emerging understanding of the subtleties in habitat preference of each species would be captured at 10km scale.

That said the predicted abundance range is not implausible but may be slightly low; perhaps due to the low maximum estimate of density. The lack of spatial variation of abundance across the predicted distribution (Figure 2b) may be acceptable for such a generalist species.

References:

Fairless, L. (2013). Ecology and ecophysiology of social structure and population dynamics in bats (*Vespertilionidae*). Ph.D. Thesis, University of Southampton.

Harris, S. J., P. Morris, S. Wray and D. Yalden (1995). A review of British mammals: population estimates and conservation status of British mammals other than cetaceans, Joint Nature Conservation Committee, Peterborough, UK.

Jones, K. E., J. D. Altringham and R. Deaton (1996). Distribution and population densities of seven species of bat in northern England. *Journal of Zoology* 240(4): 788-798.

Speakman, J. R., P. A. Racey, C. M. C. Catto, P. I. Webb, S. M. Swift and A. M. Burnett (1991). Minimum summer populations and densities of bats in NE Scotland, near the northern borders of their distributions. *Journal of Zoology* 225(2): 327-345.

Table 1: Summary of observed data and model predictions by land cover class (LCM2007 target classification). Values shown in brackets denote the spatial coverage based on a 10km resolution raster map (number of grid cells). Years represent the median of records within each land class. Ranges for density and abundance are derived using the respective minimum and maximum raster maps (lower bound is mean of values across minimum raster map with upper across the maximum) which capture the spatial uncertainty generate by projecting irregular polygons describing survey sites onto a raster grid.

LCM2007 class	Observed					Predicted		
	Occurrence		Density					
	Records	Year	Estimates	Year	Range	Habitat suitability	Density	Abundance
1 (Broadleaved woodland)	144 (8)	2014	0 (0)	-	-	0.9 (11)	3 - 10.6	3,303 - 11,678
2 (Coniferous woodland)	413 (95)	1994	6 (6)	1987	8.87 - 18.2	0.74 (76)	1.47 - 9.55	11,189 - 72,614
3 (Arable and Horticultural)	15,429 (875)	2013	15 (15)	1990	4.46 - 13.1	0.9 (945)	2.66 - 9.78	251,526 - 924,308
4 (Improved grassland)	9825 (617)	2011	25 (25)	1990	5.11 - 14.1	0.83 (643)	2.39 - 9.89	153,508 - 635,827
5 (Rough grassland)	88 (15)	2000	0 (0)	-	-	0.36 (8)	0.58 - 8.69	466.1 - 6,949
6 (Neutral grassland)	0 (0)	-	0 (0)	-	-	0 (0)	-	0
7 (Calcareous grassland)	12 (2)	1996	0 (0)	-	-	0.94 (2)	3.08 - 10.7	616.6 - 2,135
8 (Acid grassland)	455 (102)	1998	0 (0)	-	-	0.64 (76)	1.28 - 10.6	9,756 - 80,382
9 (Fen, Marsh, and Swamp)	0 (0)	-	0 (0)	-	-	-	-	0
10 (Heather)	43 (22)	1994	6 (6)	1987	3.62 - 18.2	0.62 (12)	0.74 - 10.6	882.8 - 12,771
11 (Heather grassland)	120 (41)	1994	1 (1)	1987	2.73 - 18.2	0.46 (12)	0.61 - 9.96	730.9 - 11,957
12 (Bog)	135 (33)	1994	0 (0)	-	-	0.38 (14)	1.46 - 10.7	2,044 - 14,904
13 (Montane habitat)	54 (9)	1996	0 (0)	-	-	0.42 (0)	-	0
14 (Inland rock)	1 (1)	1994	0 (0)	-	-	0.58 (0)	-	0
15 (Saltwater)	12 (3)	2006	0 (0)	-	-	0.6 (0)	-	0
16 (Freshwater)	18 (2)	2001	0 (0)	-	-	0.64 (2)	1.57 - 10.5	314.8 - 2,107
17 (Supra - littoral rock)	0 (0)	-	0 (0)	-	-	0.01 (0)	-	0
18 (Supra - littoral sediment)	1 (1)	1993	0 (0)	-	-	0.46 (0)	-	0
19 (Littoral rock)	2 (1)	1964	0 (0)	-	-	0.39 (0)	-	0
20 (Littoral sediment)	302 (14)	2012	0 (0)	-	-	0.66 (0)	-	0
21 (Saltmarsh)	0 (0)	-	0 (0)	-	-	-	-	0
22 (Urban)	117 (7)	2013	0 (0)	-	-	0.93 (8)	1.42 - 7.3	1,133 - 5,818
23 (Suburban)	1,956 (71)	2013	1 (1)	1987	9.33 - 18.2	0.91 (77)	2.42 - 8.8	18,629 - 67,749
Total	29,127 (1,919)	2011	54 (54)	1987	5.22 - 14.9	0.76 (1,886)	2.41 - 9.8	454,098 - 1,849,199

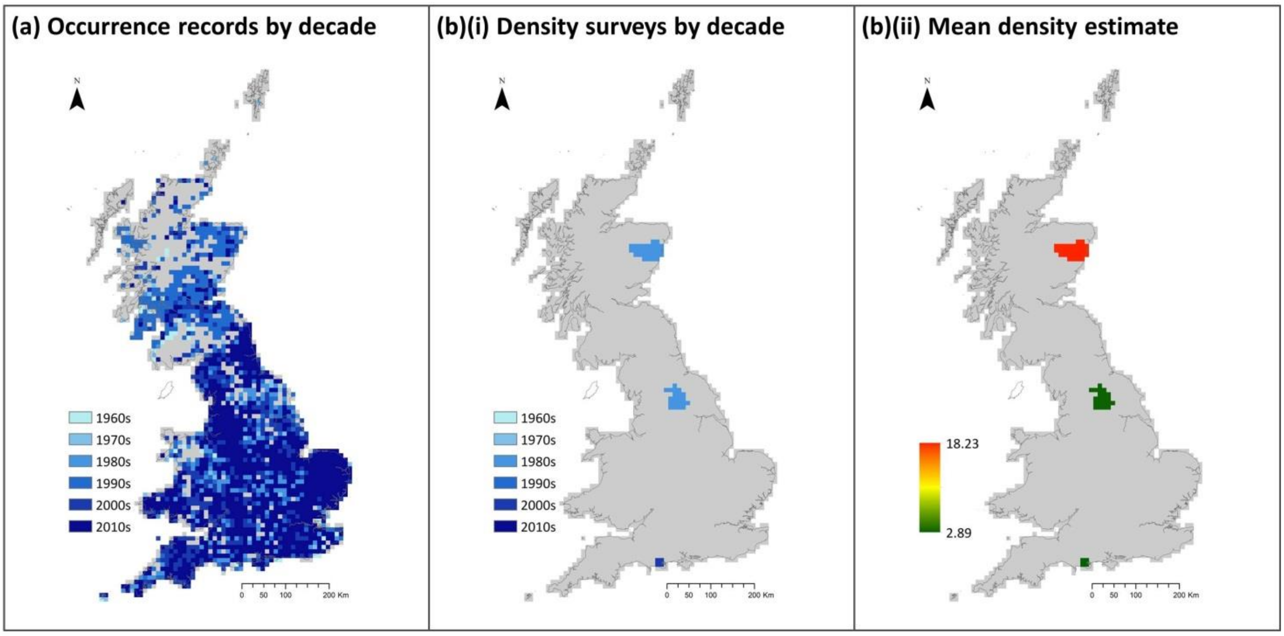

© Crown copyright and database rights 2016 Ordnance Survey 100051110. Data courtesy of the NBN Gateway with thanks to all data contributors. The NBN and its data contributors bear no responsibility for the further analysis or interpretation of this material, data and/or information.

Figure 1: 10km resolution raster maps based on BNG presenting the geographic description of available data. (a) shows the distribution of species occurrence obtained via the NBN Gateway categorised by the decade of last sighting. (b) shows information relating to density surveys identified via a search of published literature where: (i) categorises surveys by the decade of last survey; and (ii) shows the mean density estimate of surveys within grid cells (estimates assumed to be representative of entire cell, considered the upper limit of observed density).

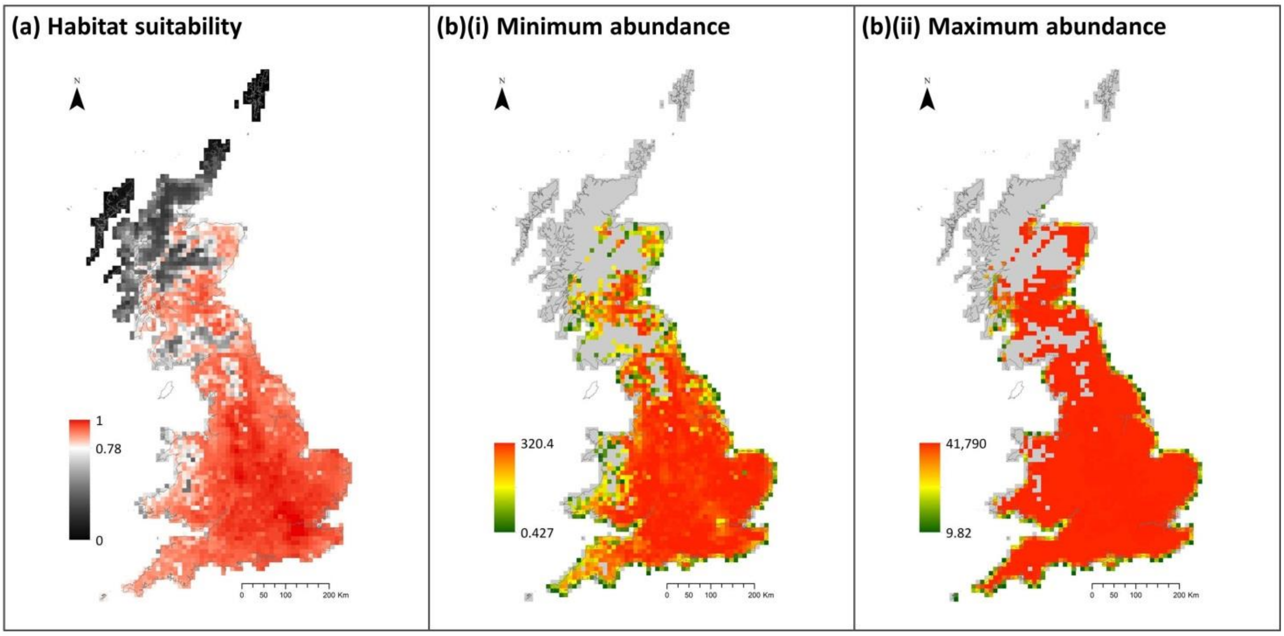

© Crown copyright and database rights 2016 Ordnance Survey 100051110. Data courtesy of the NBN Gateway with thanks to all data contributors. The NBN and its data contributors bear no responsibility for the further analysis or interpretation of this material, data and/or information.

Figure 2: Modelling predictions generated using systematic approach based on available data. (a) shows habitat suitability scores (the likelihood of observing the target species within each grid cell given variation environmental variables) determined by aggregating outputs from the “best” species distribution model (7 models compared) across 100 simulations. Here, the mid value on the scale denotes the threshold score above which occurrence is assumed. (b) shows: (i) the lower bound (Minimum); and (ii) the upper bound (Maximum); of abundance estimates determined by relating observed density (taking into account potential uncertainty) with habitat suitability scores using linear regression.
