# Supplementary material for: A systematic approach to estimate the distribution and total abundance of British mammals
Source: PLoS One. 2017 Jun 28;12(6):e0176339. doi: 10.1371/journal.pone.0176339 (PMC5489149; doi:10.1371/journal.pone.0176339)
Supplement: S5 File — Individual reports for each of the Chiroptera species presenting analysis of the available data and subsequent model predictions based on a 10km raster grid. Reports also include expert comment assessing the reliability (and plausibility) of results in the context of existing evidence and popular opinion. (ZIP) [file pone.0176339.s005.zip › O Serotine.pdf]

Serotine (*Eptesicus serotinus*)

Order: *Chiroptera*

Genus: *Eptesicus*

Origin: Native

Status: Locally common

1995 abundance estimate: 15,000 (4)

Reported population trends: BCT 2014 (↔)

Data:

The available occurrence records indicate that the serotine bat is widespread across the south of England and East Anglia with scattered patches in central regions of England and in Wales (Figure 1a). The species is notably absent in the north of England and in Scotland. Sightings were reported in various habitats (predominantly arable and improved grassland) with the majority of cells where occurrence was observed containing at least one record since 1995.

From the literature review we identified a single survey (Robinson & Stebbings 1997) conducted in East Anglia in 1991 (Figure 1b). Estimates ranged between 0.2 and 0.46 per km^2 with densities recorded in landscapes dominated by arable land cover. Unfortunately, due to the limited geographic area of this study estimates were unavailable for other habitats where occurrence was observed (marked grey in Table 1).

Model predictions:

The habitat suitability map (Figure 2a) appears to reflect the underlying data well with the set of "best" models predicting presence (and absence) to a mean AUC of 0.70. Overall, across 100 repetitions Random Forest proved to be the most commonly selected modelling approach displaying the highest AUC 26% of the time closely followed by Generalised Linear Models (24%) and MaxEnt (20%). By land cover the mean habitat suitability scores suggest observation is most likely in landscapes dominated by broadleaved woodland (Table 1) but, consistent with recorded sightings, the majority of occurrence is predicted in grid cells dominated by arable and improved grassland.

Neither minimum nor maximum density estimates showed a correlation with habitat suitability. Both were best fitted using a GLM with a gamma distribution applying density as a fixed constant in cells where occurrence was predicted.

The predicted abundance range contains the estimate from Harris et al. (1995) which, in agreement with recent trend analysis, suggests no significant change in the total population.

Reliability (Expert comment):

The observed occurrence records appear plausible. However, sightings towards the edge of the suggested range may reflect nomadic or dispersing individuals and not established populations as the distribution of known nursery roosts is more restricted. Consequently, the omission of such occurrence by the predicted habitat suitability map (Figure 2a) could be argued a better representation of the population. The density estimates obtained from the literature are likely an underestimation of the local populations measured. These populations may also represent populations towards the cold edge of the European range and probably represent a low estimate when considered at the national scale.

The predicted abundance range is plausible and may well capture the possibility that the estimate reported in Harris et al. (1995) was a slight overestimate. Typically, this species is quite patchy across the landscape which may support an argument to favours an estimate towards the lower end of the range.

References:

Harris, S. J., P. Morris, S. Wray and D. Yalden (1995). A review of British mammals: population estimates and conservation status of British mammals other than cetaceans, Joint Nature Conservation Committee, Peterborough, UK.

Robinson, M. F. and R. E. Stebbings (1997). Home range and habitat use by the serotine bat, *Eptesicus serotinus*, in England. *Journal of Zoology* 243(1): 117-136.

Table 1: Summary of observed data and model predictions by land cover class (LCM2007 target classification). Values shown in brackets denote the spatial coverage based on a 10km resolution raster map (number of grid cells). Years represent the median of records within each land class. Ranges for density and abundance are derived using the respective minimum and maximum raster maps (lower bound is mean of values across minimum raster map with upper across the maximum) which capture the spatial uncertainty generate by projecting irregular polygons describing survey sites onto a raster grid.

LCM2007 class	Observed					Predicted		
	Occurrence		Density					
	Records	Year	Estimates	Year	Range	Habitat suitability	Density	Abundance
1 (Broadleaved woodland)	60 (9)	2010	0 (0)	-	-	0.78 (9)	0.08 - 0.32	74.18 - 283.8
2 (Coniferous woodland)	38 (2)	2012	0 (0)	-	-	0.15 (2)	0.08 - 0.32	16.48 - 63.07
3 (Arable and Horticultural)	2,822 (326)	2011	12 (5)	1991	0.08 - 0.32	0.61 (444)	0.08 - 0.29	3,393 - 12,982
4 (Improved grassland)	1,396 (181)	2011	0 (0)	-	-	0.44 (220)	0.08 - 0.29	1,659 - 6,347
5 (Rough grassland)	12 (2)	2014	0 (0)	-	-	0.15 (2)	0.07 - 0.28	14.39 - 55.06
6 (Neutral grassland)	0 (0)	-	0 (0)	-	-	0.02 (0)	-	-
7 (Calcareous grassland)	22 (2)	2013	0 (0)	-	-	0.93 (2)	0.08 - 0.32	16.48 - 63.07
8 (Acid grassland)	30 (4)	2005	0 (0)	-	-	0.16 (0)	-	-
9 (Fen, Marsh, and Swamp)	0 (0)	-	0 (0)	-	-	-	-	-
10 (Heather)	0 (0)	-	0 (0)	-	-	0.14 (0)	-	-
11 (Heather grassland)	0 (0)	-	0 (0)	-	-	0.11 (0)	-	-
12 (Bog)	1 (1)	2012	0 (0)	-	-	0.1 (0)	-	-
13 (Montane habitat)	0 (0)	-	0 (0)	-	-	0.12 (0)	-	-
14 (Inland rock)	0 (0)	-	0 (0)	-	-	0.1 (0)	-	-
15 (Saltwater)	5 (1)	2007	0 (0)	-	-	0.4 (2)	0.02 - 0.07	3.88 - 14.85
16 (Freshwater)	0 (0)	-	0 (0)	-	-	0.12 (0)	-	-
17 (Supra - littoral rock)	0 (0)	-	0 (0)	-	-	0.05 (0)	-	-
18 (Supra - littoral sediment)	0 (0)	-	0 (0)	-	-	0.27 (0)	-	-
19 (Littoral rock)	0 (0)	-	0 (0)	-	-	0.2 (0)	-	-
20 (Littoral sediment)	3 (3)	2007	0 (0)	-	-	0.38 (0)	-	-
21 (Saltmarsh)	0 (0)	-	0 (0)	-	-	-	-	-
22 (Urban)	4 (2)	2010	0 (0)	-	-	0.5 (2)	0.08 - 0.32	16.48 - 63.07
23 (Suburban)	338 (28)	2011	0 (0)	-	-	0.6 (32)	0.07 - 0.27	224.9 - 860.6
Total	4,731 (561)	2011	12 (5)	1991	0.08 - 0.32	0.41 (715)	0.08 - 0.29	5,419 - 20,733

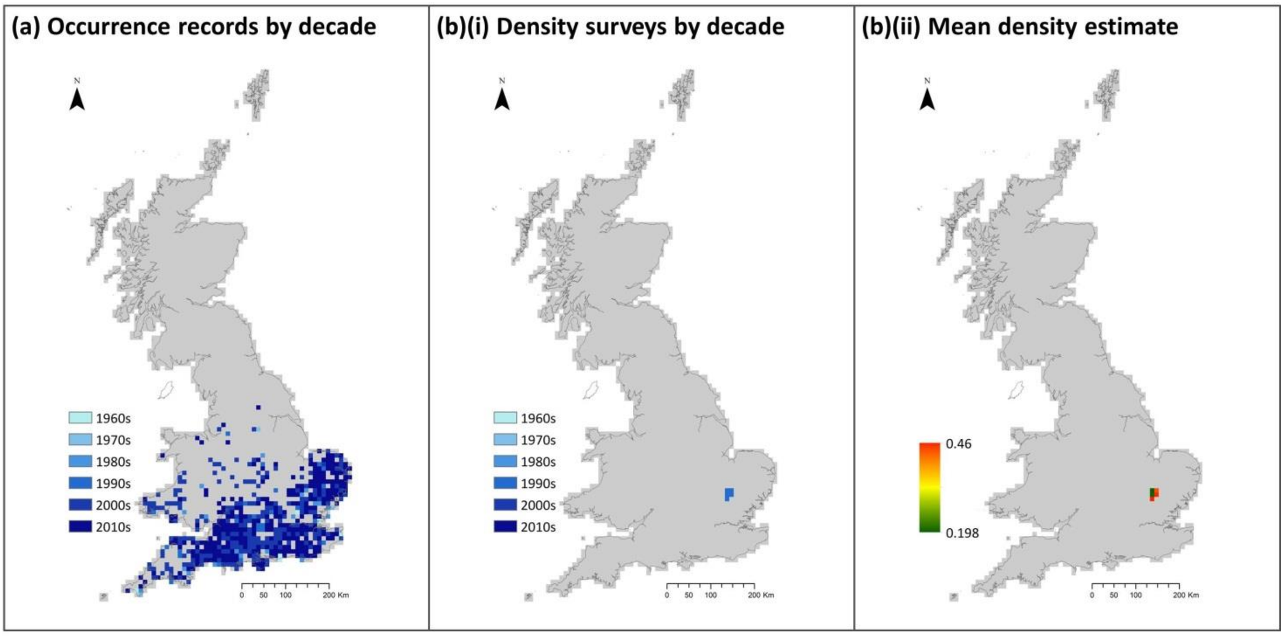

© Crown copyright and database rights 2016 Ordnance Survey 100051110. Data courtesy of the NBN Gateway with thanks to all data contributors. The NBN and its data contributors bear no responsibility for the further analysis or interpretation of this material, data and/or information.

Figure 1: 10km resolution raster maps based on BNG presenting the geographic description of available data. (a) shows the distribution of species occurrence obtained via the NBN Gateway categorised by the decade of last sighting. (b) shows information relating to density surveys identified via a search of published literature where: (i) categorises surveys by the decade of last survey; and (ii) shows the mean density estimate of surveys within grid cells (estimates assumed to be representative of entire cell, considered the upper limit of observed density).

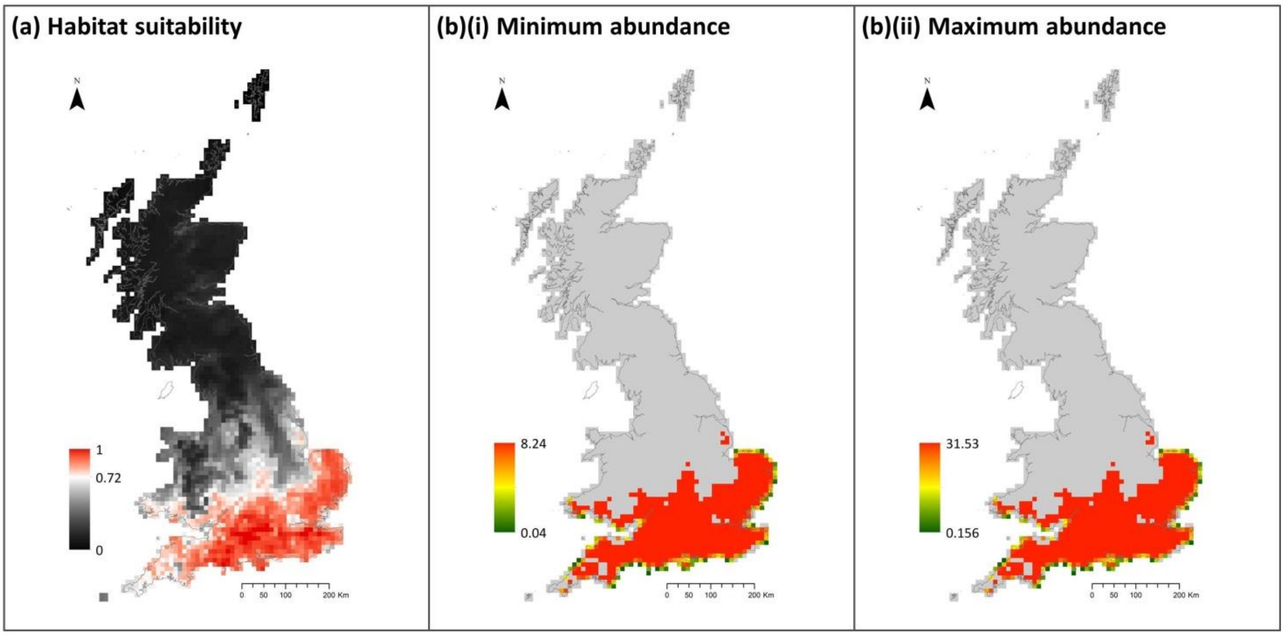

© Crown copyright and database rights 2016 Ordnance Survey 100051110. Data courtesy of the NBN Gateway with thanks to all data contributors. The NBN and its data contributors bear no responsibility for the further analysis or interpretation of this material, data and/or information.

Figure 2: Modelling predictions generated using systematic approach based on available data. (a) shows habitat suitability scores (the likelihood of observing the target species within each grid cell given variation environmental variables) determined by aggregating outputs from the "best" species distribution model (7 models compared) across 100 simulations. Here, the mid value on the scale denotes the threshold score above which occurrence is assumed. (b) shows: (i) the lower bound (Minimum); and (ii) the upper bound (Maximum); of abundance estimates determined by relating observed density (taking into account potential uncertainty) with habitat suitability scores using linear regression.
