# Supplementary material for: A systematic approach to estimate the distribution and total abundance of British mammals
Source: PLoS One. 2017 Jun 28;12(6):e0176339. doi: 10.1371/journal.pone.0176339 (PMC5489149; doi:10.1371/journal.pone.0176339)
Supplement: S5 File — Individual reports for each of the Chiroptera species presenting analysis of the available data and subsequent model predictions based on a 10km raster grid. Reports also include expert comment assessing the reliability (and plausibility) of results in the context of existing evidence and popular opinion. (ZIP) [file pone.0176339.s005.zip › P Whiskered bat.pdf]

Whiskered bat (*Myotis mystacinus*)

Order: *Chiroptera*

Genus: *Myotis*

Origin: Native

Status: Locally common

1995 abundance estimate: 40,000 (4)

Reported population trends: JNCC 2005, BCT 2014 (↔)

Data:

The available occurrence records indicate that whiskered bats are locally distributed throughout England and Wales with the largest patches of coverage in the north west of England and in Cornwall (Figure 1a). There is a notable absence across much of Scotland and the south east of England. In general, sightings were reported in various habitats (predominantly grid cells dominated by arable and improved grassland) with the majority of cells where occurrence was observed containing at least one record since 1995.

From the literature review we identified a single survey (Jones et al. 1996) conducted in northern England in 1990 which estimated density to be approximately 1.5 per km^2 (Figure1b). This survey only sampled habitats dominated by arable and improved grassland, consequently no estimates were available for other habitats where occurrence was observed (marked grey in Table 1).

Model predictions:

The habitat suitability map (Figure 2a) appears to reflect the underlying data reasonably well with the set of "best" models predicting presence (and absence) to a mean AUC of 0.67. Overall, across 100 repetitions MaxEnt proved to be the most commonly selected modelling approach displaying the highest AUC 39% of the time followed by Random Forest (24%) and Support Vector Machines (17%). By land cover the mean habitat suitability scores suggest observation is most likely in landscapes dominated by calcareous grassland (Table 1) but, consistent with recorded sightings, the majority of occurrence is predicted in grid cells dominated by arable and improved grassland.

Neither minimum nor maximum density estimates showed a correlation with habitat suitability, possibly due to the limited number of density estimates. Minimum density was best fitted using a GLM with a gamma distribution. Whereas, maximum density was normally distributed and suggested a best fit without any consideration for spatial autocorrelation. In both cases density was applied as a fixed constant in all cells where occurrence was predicted.

The predicted abundance range contains the estimate from Harris et al. (1995) suggesting no significant change in the total population, and is in agreement with recent trend analysis.

Reliability (Expert comment):

The distribution suggested by the observed occurrence records is plausible for such an apparently widespread species. However, there may be gaps in reporting, particularly in Yorkshire and East Anglia as it is also commonly found in these regions. A number of factors may combine to make the estimate of abundance poor. The reported density estimate may underestimate the measured population (its extrapolation assumed a large foraging radius) and there is a high degree of spatial variation of roost density within the surveyed area. In addition, the survey location may represent a low density population in a national context.

The predicted habitat suitability map suggests a distribution covering a larger area than records suggest. The poor association with south eastern regions of England, where occurrence would be expected, is perhaps artefact of limited records. The predicted abundance range could be considered more plausible than that reported by Harris et al. (1995), which was not considered particularly reliable (score of 4), in suggesting the possibility of a larger total population for such a wide spread species.

References:

Harris, S. J., P. Morris, S. Wray and D. Yalden (1995). A review of British mammals: population estimates and conservation status of British mammals other than cetaceans, Joint Nature Conservation Committee, Peterborough, UK.

Jones, K. E., J. D. Altringham and R. Deaton (1996). Distribution and population densities of seven species of bat in northern England. *Journal of Zoology* 240(4): 788-798.

Table 1: Summary of observed data and model predictions by land cover class (LCM2007 target classification). Values shown in brackets denote the spatial coverage based on a 10km resolution raster map (number of grid cells). Years represent the median of records within each land class. Ranges for density and abundance are derived using the respective minimum and maximum raster maps (lower bound is mean of values across minimum raster map with upper across the maximum) which capture the spatial uncertainty generate by projecting irregular polygons describing survey sites onto a raster grid.

LCM2007 class	Observed					Predicted		
	Occurrence		Density					
	Records	Year	Estimates	Year	Range	Habitat suitability	Density	Abundance
1 (Broadleaved woodland)	17 (4)	2011	0 (0)	-	-	0.63 (5)	0.32 - 1.5	159 - 750
2 (Coniferous woodland)	35 (7)	1997	0 (0)	-	-	0.2 (5)	0.32 - 1.5	159 - 750
3 (Arable and Horticultural)	1,090 (274)	2003	10 (10)	1990	0.3 - 1.5	0.61 (517)	0.3 - 1.41	15,455 - 72,923
4 (Improved grassland)	1,209 (266)	2002	3 (3)	1990	0.37 - 1.5	0.63 (458)	0.29 - 1.38	13,381 - 63,138
5 (Rough grassland)	1 (1)	2004	0 (0)	-	-	0.14 (2)	0.32 - 1.5	63.54 - 299.8
6 (Neutral grassland)	0 (0)	-	0 (0)	-	-	0.02 (0)	-	-
7 (Calcareous grassland)	1 (1)	1985	0 (0)	-	-	0.73 (2)	0.32 - 1.5	63.58 - 300
8 (Acid grassland)	100 (24)	1998	0 (0)	-	-	0.28 (31)	0.32 - 1.49	981.3 - 4,630
9 (Fen, Marsh, and Swamp)	0 (0)	-	0 (0)	-	-	-	-	-
10 (Heather)	1 (1)	1998	0 (0)	-	-	0.16 (1)	0 - 0	0.09 - 0.42
11 (Heather grassland)	6 (2)	1996	0 (0)	-	-	0.1 (1)	0.32 - 1.5	31.79 - 150
12 (Bog)	9 (4)	2008	0 (0)	-	-	0.12 (6)	0.32 - 1.5	190.8 - 900
13 (Montane habitat)	1 (1)	1978	0 (0)	-	-	0.09 (0)	-	-
14 (Inland rock)	0 (0)	-	0 (0)	-	-	0.05 (0)	-	-
15 (Saltwater)	6 (1)	2006	0 (0)	-	-	0.46 (0)	-	-
16 (Freshwater)	0 (0)	-	0 (0)	-	-	0.14 (0)	-	-
17 (Supra - littoral rock)	0 (0)	-	0 (0)	-	-	0.05 (0)	-	-
18 (Supra - littoral sediment)	0 (0)	-	0 (0)	-	-	0.18 (0)	-	-
19 (Littoral rock)	0 (0)	-	0 (0)	-	-	0.16 (0)	-	-
20 (Littoral sediment)	24 (7)	2002	0 (0)	-	-	0.49 (12)	0.2 - 0.94	238.6 - 1,126
21 (Saltmarsh)	0 (0)	-	0 (0)	-	-	-	-	-
22 (Urban)	0 (0)	-	0 (0)	-	-	0.32 (0)	-	-
23 (Suburban)	42 (15)	1996	0 (0)	-	-	0.45 (6)	0.28 - 1.32	167.4 - 789.8
Total	2,542 (608)	2002	13 (13)	1990	0.32 - 1.5	0.47 (1,046)	0.3 - 1.39	30,892 - 145,757

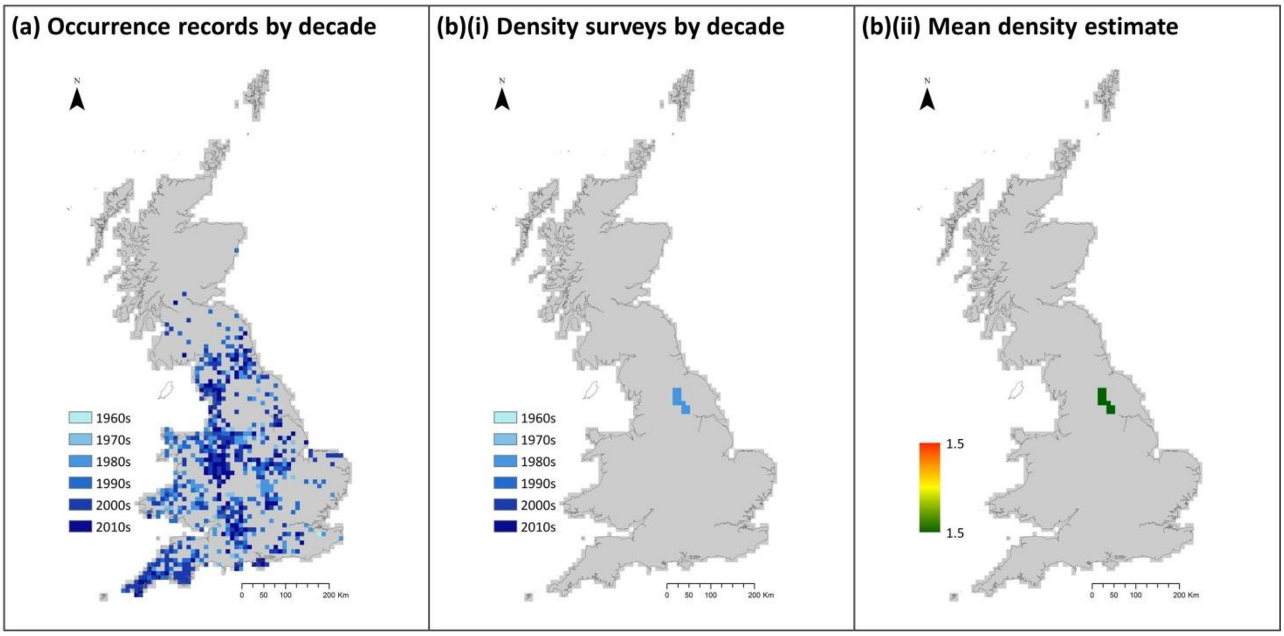

© Crown copyright and database rights 2016 Ordnance Survey 100051110. Data courtesy of the NBN Gateway with thanks to all data contributors. The NBN and its data contributors bear no responsibility for the further analysis or interpretation of this material, data and/or information.

Figure 1: 10km resolution raster maps based on BNG presenting the geographic description of available data. (a) shows the distribution of species occurrence obtained via the NBN Gateway categorised by the decade of last sighting. (b) shows information relating to density surveys identified via a search of published literature where: (i) categorises surveys by the decade of last survey; and (ii) shows the mean density estimate of surveys within grid cells (estimates assumed to be representative of entire cell, considered the upper limit of observed density).

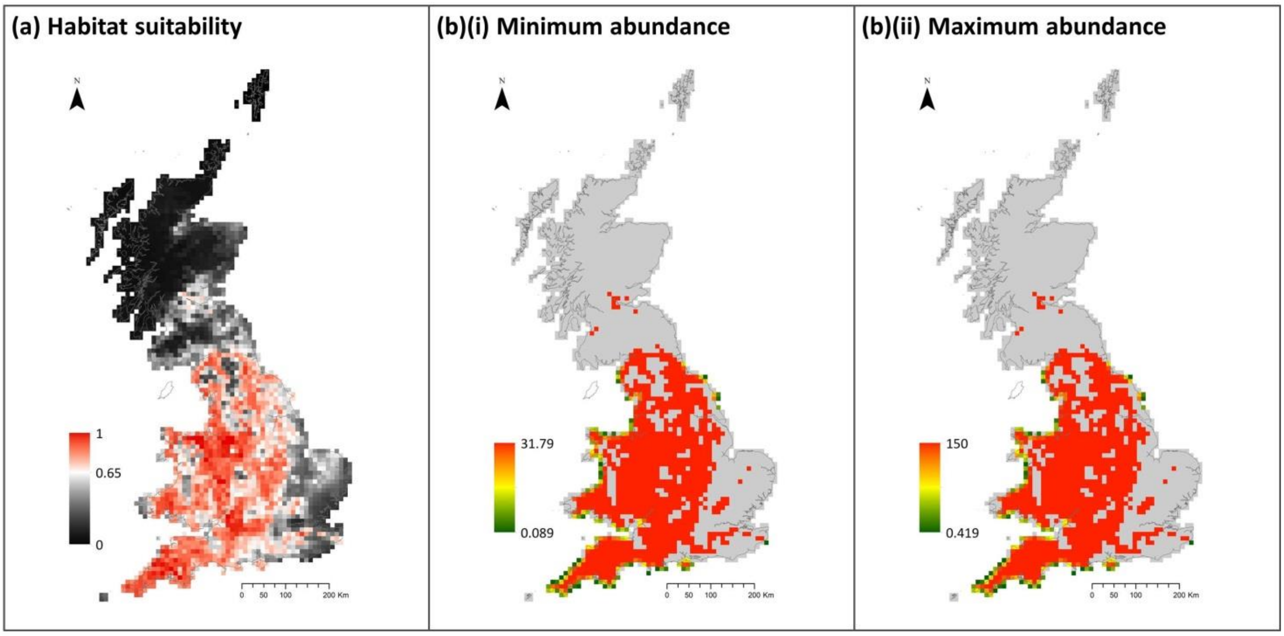

© Crown copyright and database rights 2016 Ordnance Survey 100051110. Data courtesy of the NBN Gateway with thanks to all data contributors. The NBN and its data contributors bear no responsibility for the further analysis or interpretation of this material, data and/or information.

Figure 2: Modelling predictions generated using systematic approach based on available data. (a) shows habitat suitability scores (the likelihood of observing the target species within each grid cell given variation environmental variables) determined by aggregating outputs from the "best" species distribution model (7 models compared) across 100 simulations. Here, the mid value on the scale denotes the threshold score above which occurrence is assumed. (b) shows: (i) the lower bound (Minimum); and (ii) the upper bound (Maximum); of abundance estimates determined by relating observed density (taking into account potential uncertainty) with habitat suitability scores using linear regression.
