# Supplementary material for: A systematic approach to estimate the distribution and total abundance of British mammals
Source: PLoS One. 2017 Jun 28;12(6):e0176339. doi: 10.1371/journal.pone.0176339 (PMC5489149; doi:10.1371/journal.pone.0176339)
Supplement: S6 File — Individual reports for each of the Insectivora species presenting analysis of the available data and subsequent model predictions based on a 10km raster grid. Reports also include expert comment assessing the reliability (and plausibility) of results in the context of existing evidence and popular opinion. (ZIP) [file pone.0176339.s006.zip › A Common shrew.pdf]

## Common shrew (*Sorex araneus*)

**Order:** *Insectivora* **Genus:** *Sorex*

**Origin:** Native **Status:** Common

**1995 abundance estimate:** 41,700,000 (3) **Reported population trends:** None

**Data:**

The available occurrence records indicate that the common shrew is widespread throughout GB (Figure 1a) with observation in various habitats (primarily those dominated by arable and improved grassland). However, the map highlights patches where the species has not been recorded for some time (south east England and Wales), or not at all (Scotland).

From the literature review we identified several studies (Baker et al. 2005; Churchfield & Brown 1987; Gelling et al. 2007; Kotzageorgis & Mason 1997; Pernetta 1977; Shore et al. 2005; Tattersall et al. 2002; White & Searle 2007) located across the range of observed occurrence (Figure 1b). Estimates ranged between 0 and 3,267 per km$^2$ with the highest densities reported in suburban dominated land cover (75.13 - 1,382 per km$^2$ accounting for uncertainty relating to unsurveyed areas within grid cells). Due to the limited coverage of density surveys estimates were unavailable for several dominant land covers where occurrence was reported (marked grey in Table 1) and where estimates were available the relative uncertainty within cells was large.

**Model predictions:**

The habitat suitability map (Figure 2a) appears to reflect the underlying data reasonably well with the set of "best" models predicting presence (and absence) to a mean AUC of 0.70. Overall, across 100 repetitions MaxEnt proved to be the most commonly selected modelling approach displaying the highest AUC 44% of the time followed by Random Forest (28%). By land cover the mean habitat suitability scores suggest observation is most likely in landscapes dominated by calcareous grassland (Table 1) but, consistent with recorded sightings, the majority of occurrence is predicted in arable habitat (the most common dominant land covers at a 10km scale). Occurrence is preserved in all land covers where it is observed with the exception of inland rock.

Both minimum and maximum density estimates were best fitted linearly to habitat suitability accounting for spherical spatial autocorrelation. However, interestingly the relationship appear to shows a negative correlation predicting lower abundance in cells of high suitability (this pattern is less consistent across the distribution of maximum abundance only seeming to affect the areas around London and the across the Midlands).

Despite this, the predicted abundance range contains the estimate from Harris et al. (1995) suggesting no change in the total population. Although, the range is very large due to the uncertainty caused by small survey sites relative to the 10km scale at which modelling is performed. Given the home range of the species it is perhaps reasonable to suggest that the true estimate lies towards the lower end of the range where small isolated populations within cells are assumed unrepresentative of the wider landscape. In order to provide more accurate predictions future model analysis could be based on a finer scale raster grid which would better represent the variations in habitat for smaller mammals. Unfortunately, at present this is too unreliable due to access restrictions imposed on occurrence data.

**Reliability (Expert comment):**

Records suggest continued widespread distribution in Britain, although there appear to be some regional changes. Compared to data reported by Arnold (1993), recent sightings appear to have increased in East Anglia, south-west England and northern England (Cumbria, Northumberland, Durham) and declined in south east England (Kent, Sussex). It is unclear whether these represent real changes or recording bias. In Britain (and elsewhere) preferred habitats of common shrews include grassland, woodland, arable land and hedgerows. The data presented here include a relatively low number of observations for woodland, although high levels of habitat suitability were correctly predicted for coniferous and broadleaved woodland. There were no observations for neutral grassland and hence a

low habitat suitability index and zero population were predicted for this habitat category. It is unclear how much influence this had on the predicted total abundance as the proportion of land cover for each habitat type is not given here.

**References:**


Arnold H. R. (1993). Atlas of mammals in Britain: HMSO.

Baker, P. J., A. J. Bentley, R. J. Ansell and S. Harris (2005). Impact of predation by domestic cats *Felis catus* in an urban area. *Mammal Review* 35(3-4): 302-312.

Churchfield, S. and V. K. Brown (1987). The trophic impact of small mammals in successional grasslands. *Biological Journal of the Linnean Society* 31(3): 273-290.

Gelling, M., D. W. Macdonald and F. Mathews (2007). Are hedgerows the route to increased farmland small mammal density? Use of hedgerows in British pastoral habitats. *Landscape Ecology* 22(7): 1019-1032.

Harris, S. J., P. Morris, S. Wray and D. Yalden (1995). A review of British mammals: population estimates and conservation status of British mammals other than cetaceans, Joint Nature Conservation Committee, Peterborough, UK.

Kotzageorgis, G. C. and C. F. Mason (1997). Small mammal populations in relation to hedgerow structure in an arable landscape. *Journal of Zoology* 242(3): 425-434.

Pernetta, J. C. (1977). Population ecology of British shrews in grassland. *Acta Theriologica* 22(20): 279-296.

Shore, R. F., W. R. Meek, T. H. Sparks, R. F. Pywell and M. Nowakowski (2005). Will environmental stewardship enhance small mammal abundance on intensively managed farmland? *Mammal Review* 35(3-4): 277-284.

Tattersall, F. H., D. W. Macdonald, B. J. Hart, P. Johnson, W. Manley and R. Feber (2002). Is habitat linearity important for small mammal communities on farmland? *Journal of Applied Ecology* 39(4): 643-652.

White, T. A. and J. B. Searle (2007). Genetic diversity and population size: island populations of the common shrew, *Sorex araneus*. *Molecular Ecology* 16(10): 2005-2016.

**Table 1:** Summary of observed data and model predictions by land cover class (LCM2007 target classification). Values shown in brackets denote the spatial coverage based on a 10km resolution raster map (number of grid cells). Years represent the median of records within each land class. Ranges for density and abundance are derived using the respective minimum and maximum raster maps (lower bound is mean of values across minimum raster map with upper across the maximum) which capture the spatial uncertainty generate by projecting irregular polygons describing survey sites onto a raster grid.

| LCM2007 class | Observed | | | | | Predicted | | |
|---|---|---|---|---|---|---|---|---|
| | Occurrence | | Density | | | | | |
| | Records | Year | Estimates | Year | Range | Habitat suitability | Density | Abundance |
| 1 (Broadleaved woodland) | 75 (9) | 1969 | 1 (1) | 1986 | 0.1 - 1,309 | 0.91 (11) | 10.6 - 1,430 | 11,605 - 1,573,365 |
| 2 (Coniferous woodland) | 369 (109) | 1994 | 0 (0) | - | - | 0.83 (77) | 14.7 - 1,132 | 113,265 - 8,716,417 |
| 3 (Arable and Horticultural) | 6,882 (793) | 2003 | 9 (6) | 1997 | 0.1 - 3.7 | 0.91 (925) | 8.5 - 1,277 | 782,101 - 118,095,324 |
| 4 (Improved grassland) | 3,485 (565) | 1997 | 26 (17) | 2003 | 16.3 - 570 | 0.86 (600) | 11.3 - 1,236 | 675,777 - 74,173,203 |
| 5 (Rough grassland) | 59 (18) | 1984 | 0 (0) | - | - | 0.42 (4) | 12.5 - 759 | 4,983 - 303,407 |
| 6 (Neutral grassland) | 0 (0) | - | 0 (0) | - | - | 0.01 (0) | - | 0 |
| 7 (Calcareous grassland) | 14 (2) | 2013 | 0 (0) | - | - | 0.94 (2) | 5.33 - 1,462 | 1,065 - 292,362 |
| 8 (Acid grassland) | 263 (103) | 1992 | 4 (3) | 2005 | 3.3 - 1,067 | 0.73 (40) | 24.2 - 1,123 | 96,587 - 4,491,645 |
| 9 (Fen, Marsh, and Swamp) | 0 (0) | - | 0 (0) | - | - | - | - | 0 |
| 10 (Heather) | 63 (33) | 1990 | 0 (0) | - | - | 0.75 (11) | 22.6 - 1,169 | 24,820 - 1,286,234 |
| 11 (Heather grassland) | 274 (64) | 1999 | 7 (3) | 2005 | 9.9 - 509 | 0.62 (12) | 20.8 - 1,118 | 24,915 - 1,341,594 |
| 12 (Bog) | 117 (48) | 1995 | 0 (0) | - | - | 0.5 (11) | 16.5 - 1,282 | 18,175 - 1,410,261 |
| 13 (Montane habitat) | 53 (21) | 1994 | 0 (0) | - | - | 0.64 (1) | 17.1 - 1,320 | 1,714 - 131,950 |
| 14 (Inland rock) | 2 (1) | 2005 | 0 (0) | - | - | 0.71 (0) | - | 0 |
| 15 (Saltwater) | 85 (6) | 1996 | 0 (0) | - | - | 0.78 (1) | 8.9 - 557 | 891 - 55,663 |
| 16 (Freshwater) | 5 (2) | 1994 | 0 (0) | - | - | 0.7 (1) | 15.8 - 1,322 | 1,578 - 132,238 |
| 17 (Supra-littoral rock) | 0 (0) | - | 0 (0) | - | - | 0.09 (0) | - | 0 |
| 18 (Supra-littoral sediment) | 14 (4) | 1981 | 0 (0) | - | - | 0.64 (3) | 4 - 225 | 1,186 - 67,485 |
| 19 (Littoral rock) | 0 (0) | - | 0 (0) | - | - | 0.49 (0) | - | 0 |
| 20 (Littoral sediment) | 167 (25) | 2000 | 0 (0) | - | - | 0.84 (25) | 9.8 - 801 | 24,497 - 2,001,420 |
| 21 (Saltmarsh) | 0 (0) | - | 0 (0) | - | - | - | - | 0 |
| 22 (Urban) | 36 (7) | 2006 | 0 (0) | - | - | 0.91 (8) | 6.7 - 923 | 53,294 - 737,968 |
| 23 (Suburban) | 521 (67) | 1999 | 1 (1) | 2002 | 75.1 - 1,382 | 0.92 (76) | 6.6 - 1,163 | 500,001 - 8,840,553 |
| Total | 12,484 (1,877) | 1999 | 48 (31) | 2003 | 12.3 - 535 | 0.81 (1,808) | 10.2 - 1,237 | 1,838,490 - 223,651,088 |

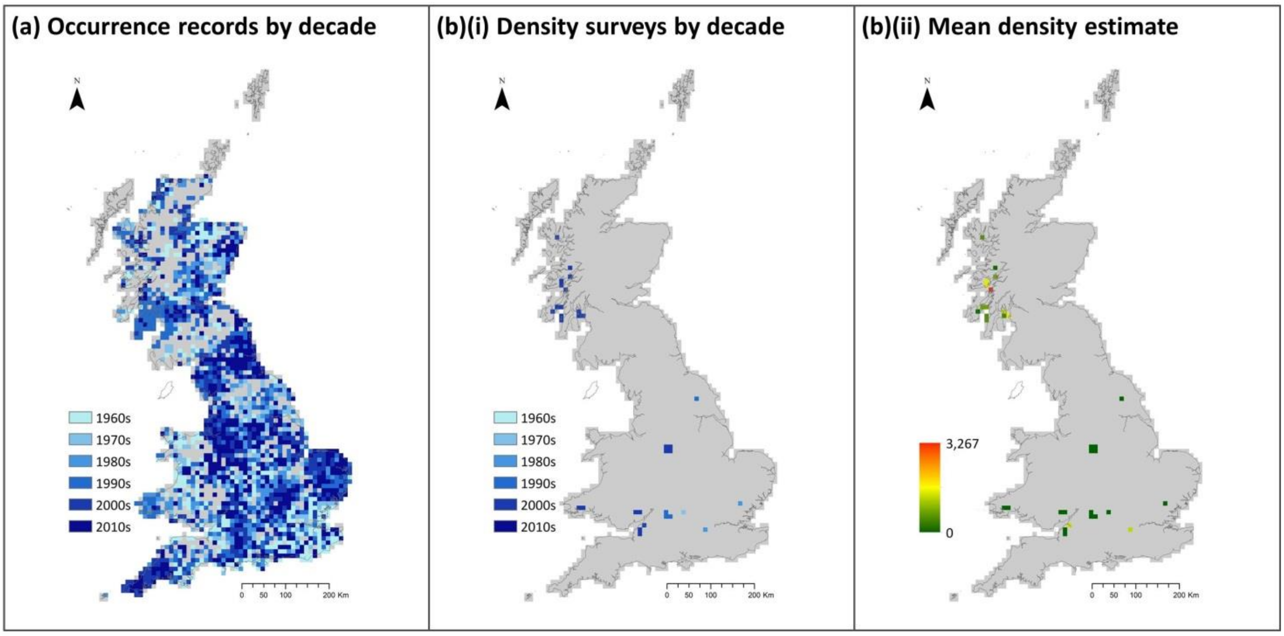


© Crown copyright and database rights 2016 Ordnance Survey 100051110. Data courtesy of the NBN Gateway with thanks to all data contributors. The NBN and its data contributors bear no responsibility for the further analysis or interpretation of this material, data and/or information.

**Figure 1:** 10km resolution raster maps based on BNG presenting the geographic description of available data. (a) shows the distribution of species occurrence obtained via the NBN Gateway categorised by the decade of last sighting. (b) shows information relating to density surveys identified via a search of published literature where: (i) categorises surveys by the decade of last survey; and (ii) shows the mean density estimate of surveys within grid cells (estimates assumed to be representative of entire cell, considered the upper limit of observed density).

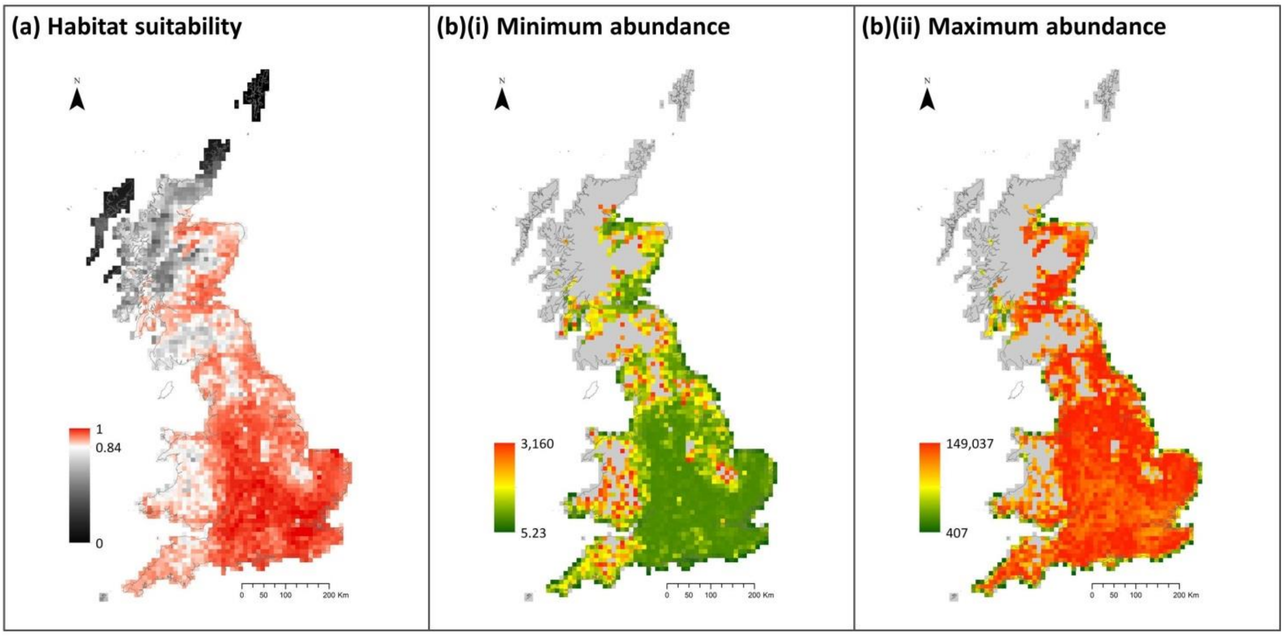


© Crown copyright and database rights 2016 Ordnance Survey 100051110. Data courtesy of the NBN Gateway with thanks to all data contributors. The NBN and its data contributors bear no responsibility for the further analysis or interpretation of this material, data and/or information.

**Figure 2:** Modelling predictions generated using systematic approach based on available data. (a) shows habitat suitability scores (the likelihood of observing the target species within each grid cell given variation environmental variables) determined by aggregating outputs from the "best" species distribution model (7 models compared) across 100 simulations. Here, the mid value on the scale denotes the threshold score above which occurrence is assumed. (b) shows: (i) the lower bound (Minimum); and (ii) the upper bound (Maximum); of abundance estimates determined by relating observed density (taking into account potential uncertainty) with habitat suitability scores using linear regression.
