# Supplementary material for: A systematic approach to estimate the distribution and total abundance of British mammals
Source: PLoS One. 2017 Jun 28;12(6):e0176339. doi: 10.1371/journal.pone.0176339 (PMC5489149; doi:10.1371/journal.pone.0176339)
Supplement: S6 File — Individual reports for each of the Insectivora species presenting analysis of the available data and subsequent model predictions based on a 10km raster grid. Reports also include expert comment assessing the reliability (and plausibility) of results in the context of existing evidence and popular opinion. (ZIP) [file pone.0176339.s006.zip › B Hedgehog.pdf]

Hedgehog (*Erinaceus europaeus*)

Order: *Insectivora*

Genus: *Erinaceus*

Origin: Native

Status: Locally common

1995 abundance estimate: 1,555,000 (4)

Reported population trends: JNCC 2005, NGC 2009 (↔)

Data:

The available occurrence records indicate that the hedgehog is widespread throughout GB with sightings reported in most 10 km squares (approximately 84%) at least once over the past decade (Figure 1a). However, the map highlights several areas, particularly in Scotland, where the species has not been recorded for some time or not at all.

Density estimates, some relative historic recorded up to three decades, were obtained from published literature spanning approximately 2% of the observed species distribution based on the available occurrence data (Doncaster 1994; Morris et al. 1992; Reeve 1981; Young et al. 2006). Geographically, these studies were concentrated in areas of south west England and Wales with one estimate in central England and one in the north. No estimates were available for the south east, Wales or Scotland (Figure 1b). Estimates ranged between 20.83 and 178.6 per km^2 with the highest densities recorded in suburban habitat (0.45 - 100 per km^2 accounting for uncertainty relating to unsurveyed areas within grid cells). Unfortunately, due to the relatively small proportion of area surveyed estimates for several land cover classes were not available (land class marked grey in Table 1).

Model predictions:

The habitat suitability map (Figure 2a) appears to reflect the underlying data well with the set of "best" models predicting presence (and absence) to a mean AUC of 0.86. Overall, across 100 repetitions MaxEnt proved to be the most commonly selected modelling approach displaying the highest AUC 57% of the time followed by Random Forest (24%). By land cover the mean habitat suitability scores suggest observation is most likely in landscapes dominated by urban and suburban land cover (Table 1) but, consistent with recorded sightings, the majority of occurrence is predicted in grid cells dominated by arable and improved grassland (the most common dominant land covers at a 10km scale).

Both minimum and maximum density estimates were best fitted linearly to square of habitat suitability accounting for spherical spatial autocorrelation. Interestingly, the relationship for minimum density suggests a negative correlation in which squares with a higher likelihood of observation contain a lower density of individuals. This inconsistency is most likely caused by scaling bias effecting the relative magnitude of minimum density.

The predicted abundance range contains the estimate from Harris et al. (1995) suggesting, in agreement with recent trends, no change in the total population. However, the range is very large due to the uncertainty caused by small survey sites relative to the 10km scale at which modelling is performed. In order to provide more accurate predictions future model analysis could be based on a finer scale raster grid which would better represent the variations in habitat for smaller mammals. Unfortunately, at present this is too unreliable due to access restrictions imposed on occurrence data.

Reliability (Expert comment):

Although the hedgehog is very widespread across Britain, local density can vary a lot. The model suggests a relatively high density in arable landscapes, but in many intensive arable areas the local density may be close to zero. Additionally, we know that hedgehog density can double when badgers are culled in the landscape (Trewby et al. 2014) and badger numbers appear to have increased over the last few decades. This may suggest that hedgehog number have declined, but the model does not include any potential impact from predation. Overall, this would suggest that the true population estimate should be closer to the lower bound and this would suggest that it relatively close to the 1995 estimate.

References:

Doncaster, C. P. (1994). Factors regulating local variations in abundance: field tests on hedgehogs, *Erinaceus europaeus*. *Oikos* 69(2): 182-192.

Harris, S. J., P. Morris, S. Wray and D. Yalden (1995). A review of British mammals: population estimates and conservation status of British mammals other than cetaceans, Joint Nature Conservation Committee, Peterborough, UK.

Morris, P. A., S. Munn and S. Craig-Wood (1992). The effects of releasing captive hedgehogs (*Erinaceus europaeus*) into the wild. *Field Studies* 8: 89-99.

Reeve, N. J. (1981). A field study of the hedgehog (*Erinaceus europaeus*) with particular reference to movements and behaviour. Ph.D. Thesis, University of London.

Trewby, I.D., R. Young, R. A. McDonald, G. J. Wilson, J. Davison, N. Walker, A. Robertson, C. P. Doncaster and R. J. Delahay (2014). Impacts of Removing Badgers on Localised Counts of Hedgehogs. *PLoS ONE*, 9: e95477.

Young, R. P., J. Davison, I. D. Trewby, G. J. Wilson, R. J. Delahay and C. P. Doncaster (2006). Abundance of hedgehogs (*Erinaceus europaeus*) in relation to the density and distribution of badgers (*Meles meles*). *Journal of Zoology* 269(3): 349-356.

Table 1: Summary of observed data and model predictions by land cover class (LCM2007 target classification). Values shown in brackets denote the spatial coverage based on a 10km resolution raster map (number of grid cells). Years represent the median of records within each land class. Ranges for density and abundance are derived using the respective minimum and maximum raster maps (lower bound is mean of values across minimum raster map with upper across the maximum) which capture the spatial uncertainty generate by projecting irregular polygons describing survey sites onto a raster grid.

LCM2007 class	Observed					Predicted		
	Occurrence		Density					
	Records	Year	Estimates	Year	Range	Habitat suitability	Density	Abundance
1 (Broadleaved woodland)	357 (11)	2013	0 (0)	-	-	0.89 (11)	3.48 - 74.56	3,829 - 82,013
2 (Coniferous woodland)	1,116 (134)	2006	0 (0)	-	-	0.76 (109)	2.22 - 69.66	24,221 - 759,296
3 (Arable and Horticultural)	47,564 (944)	2013	23 (23)	2002	5.27 - 66.19	0.86 (961)	4.25 - 51.66	408,673 - 4,964,297
4 (Improved grassland)	18,215 (728)	2013	27 (27)	2002	6.66 - 29.44	0.83 (727)	3.35 - 54.73	243,759 - 3,978,713
5 (Rough grassland)	206 (34)	2006	0 (0)	-	-	0.49 (9)	0 - 102	0 - 91,765
6 (Neutral grassland)	2 (1)	1971	0 (0)	-	-	0.61 (0)	-	0
7 (Calcareous grassland)	43 (2)	2014	0 (0)	-	-	0.88 (2)	5.38 - 60.48	1,076 - 12,097
8 (Acid grassland)	893 (141)	2006	1 (1)	1989	0.16 - 66.67	0.63 (99)	1.42 - 69.01	14,037 - 683,216
9 (Fen, Marsh, and Swamp)	0 (0)	-	0 (0)	-	-	-	-	0
10 (Heather)	271 (42)	2006	0 (0)	-	-	0.67 (32)	0.95 - 74.09	3,045 - 237,099
11 (Heather grassland)	685 (83)	2006	0 (0)	-	-	0.55 (29)	0.74 - 79.02	2,147 - 229,169
12 (Bog)	516 (80)	2006	0 (0)	-	-	0.53 (24)	1.52 - 65.79	3,654 - 157,900
13 (Montane habitat)	67 (18)	2006	0 (0)	-	-	0.39 (2)	0 - 107.2	0 - 21,433
14 (Inland rock)	2 (1)	1978	0 (0)	-	-	0.5 (0)	-	0
15 (Saltwater)	60 (8)	2012	0 (0)	-	-	0.73 (9)	0 - 39.38	0 - 35,446
16 (Freshwater)	47 (3)	2013	0 (0)	-	-	0.72 (2)	2.66 - 95.18	531.5 - 19,037
17 (Supra-littoral rock)	0 (0)	-	0 (0)	-	-	0.06 (0)	-	0
18 (Supra-littoral sediment)	12 (3)	2006	0 (0)	-	-	0.56 (3)	0 - 18.96	0 - 5,688
19 (Littoral rock)	5 (1)	2009	0 (0)	-	-	0.26 (0)	-	0
20 (Littoral sediment)	440 (29)	2013	0 (0)	-	-	0.8 (32)	1.24 - 36.92	3,969 - 118,150
21 (Saltmarsh)	0 (0)	-	0 (0)	-	-	-	-	0
22 (Urban)	365 (8)	2014	0 (0)	-	-	0.92 (8)	0.9 - 111.2	718.2 - 88,955
23 (Suburban)	6,851 (76)	2014	1 (1)	1979	0.45 - 100	0.89 (78)	2.81 - 63.47	21,885 - 495,090
Total	77,717 (2,347)	2013	52 (52)	2002	5.8 - 47.77	0.76 (2,137)	3.42 - 56.06	731,546 - 11,979,363

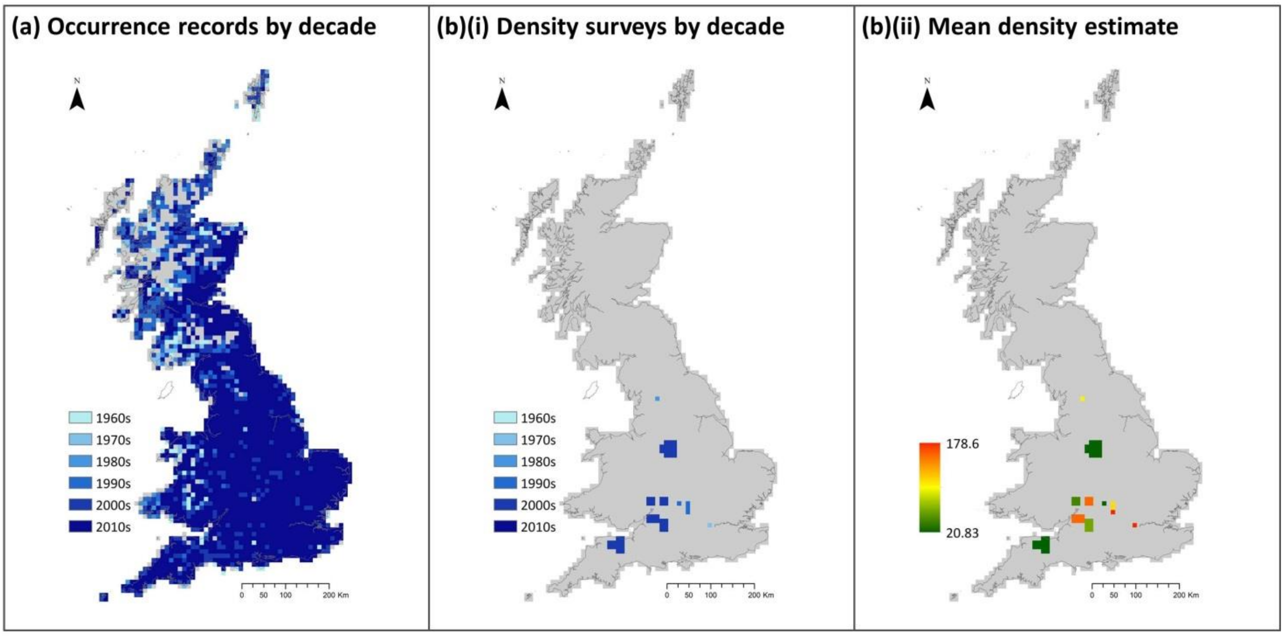

© Crown copyright and database rights 2016 Ordnance Survey 100051110. Data courtesy of the NBN Gateway with thanks to all data contributors. The NBN and its data contributors bear no responsibility for the further analysis or interpretation of this material, data and/or information.

Figure 1: 10km resolution raster maps based on BNG presenting the geographic description of available data. (a) shows the distribution of species occurrence obtained via the NBN Gateway categorised by the decade of last sighting. (b) shows information relating to density surveys identified via a search of published literature where: (i) categorises surveys by the decade of last survey; and (ii) shows the mean density estimate of surveys within grid cells (estimates assumed to be representative of entire cell, considered the upper limit of observed density).

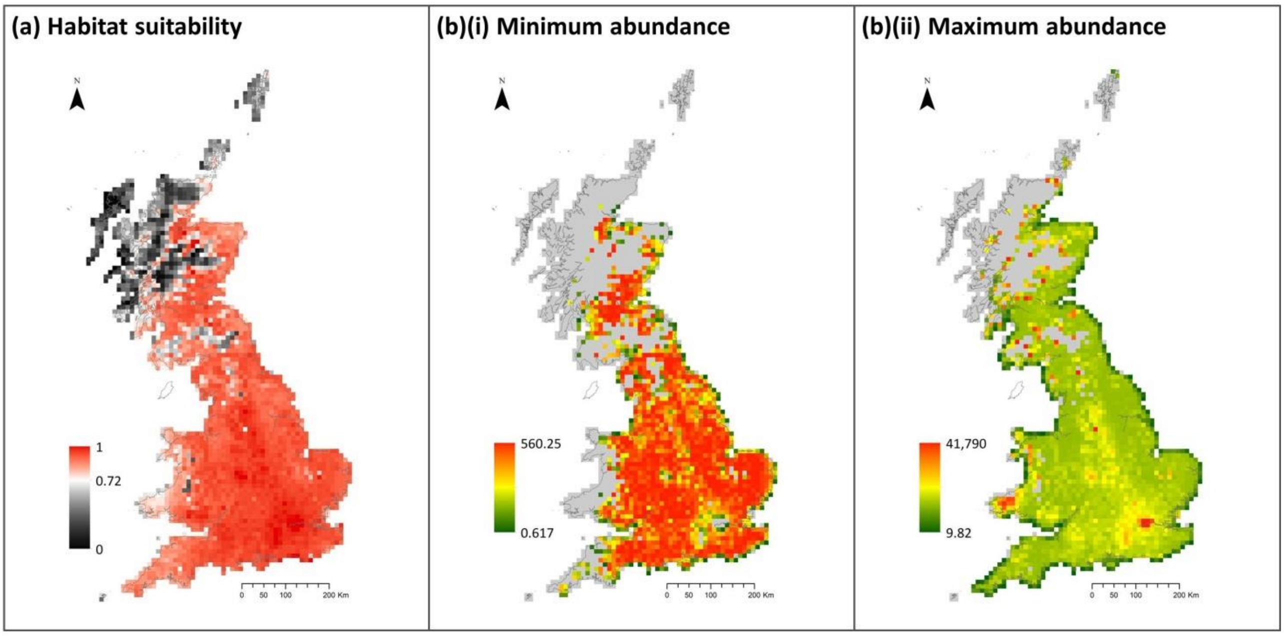

© Crown copyright and database rights 2016 Ordnance Survey 100051110. Data courtesy of the NBN Gateway with thanks to all data contributors. The NBN and its data contributors bear no responsibility for the further analysis or interpretation of this material, data and/or information.

Figure 2: Modelling predictions generated using systematic approach based on available data. (a) shows habitat suitability scores (the likelihood of observing the target species within each grid cell given variation environmental variables) determined by aggregating outputs from the "best" species distribution model (7 models compared) across 100 simulations. Here, the mid value on the scale denotes the threshold score above which occurrence is assumed. (b) shows: (i) the lower bound (Minimum); and (ii) the upper bound (Maximum); of abundance estimates determined by relating observed density (taking into account potential uncertainty) with habitat suitability scores using linear regression.
