# Supplementary material for: A systematic approach to estimate the distribution and total abundance of British mammals
Source: PLoS One. 2017 Jun 28;12(6):e0176339. doi: 10.1371/journal.pone.0176339 (PMC5489149; doi:10.1371/journal.pone.0176339)
Supplement: S6 File — Individual reports for each of the Insectivora species presenting analysis of the available data and subsequent model predictions based on a 10km raster grid. Reports also include expert comment assessing the reliability (and plausibility) of results in the context of existing evidence and popular opinion. (ZIP) [file pone.0176339.s006.zip › D Mole.pdf]

Mole (*Talpa europaea*)

Order: *Insectivora*

Genus: *Talpa*

Origin: Native

Status: Common

1995 abundance estimate: 31,000,000 (3)

Reported population trends: JNCC 2005 (↔)

Data:

The available occurrence records indicate that the mole is widespread throughout GB with sightings reported in most 10 km squares (approximately 84%) at least once over the past decade (Figure 1a). However, the map highlights several areas, particularly in Wales, where the species has not been recorded for some time.

A limited number of density estimates, recorded over the past three decades, were obtained from published literature (Macdonald et al. 1996; Stone 1986). However, the surveyed area described by these studies spanned less than 1% of the observed species distribution based on the available occurrence data) and was limited exclusively to arable dominated habitats with one survey conducted in Scotland and the other in Oxfordshire (Figure 1b). Estimates ranged between 480 and 691.3 per km^2 although due to the limited coverage of the survey within this cell the density range accounting for uncertainty was much larger (0.67 - 584.8 per km^2).

Model predictions:

The habitat suitability map (Figure 2a) appears to reflect the underlying data well with the set of "best" models predicting presence (and absence) to a mean AUC of 0.84. However, the distribution is slightly contracted towards the outer edges, particularly in the north west of Scotland where occurrence is observed. Overall, across 100 repetitions MaxEnt proved to be the most commonly selected modelling approach displaying the highest AUC 46% of the time followed by Random Forest (26%). By land cover the mean habitat suitability scores suggest observation is most likely in landscapes dominated by calcareous grassland and broadleaved woodland closely followed by arable and suburban (Table 1) but, consistent with recorded sightings, the majority of occurrence is predicted in grid cells dominated by arable and improved grassland (the most common dominant land covers at a 10km scale).

Due to the limited volume and variability of density data neither minimum nor maximum density estimates showed a correlation with habitat suitability. Both were best fitted using a "glm" with a gamma distribution applying density as a fixed constant in cells where occurrence was predicted. In agreement with recently reported trends the predicted abundance range contains the estimate from Harris et al. (1995) suggesting no significant change in the total population (since both estimates reference similar density studies this is perhaps unsurprising; the result may indicate no significant change in species distribution over the past 20 years despite the concerns highlighted above). However, as previously suggested the range is large due to the uncertainty caused by the conversion of irregular survey sites to the 10km raster grid upon which modelling is performed.

Reliability (Expert comment):

The mole is a common British species, one of the easiest to record and unlike many British mammals the density does not vary a lot from year to year. We would therefore expect the full extent of its distribution to be recorded. The lack of recent records in many areas (Figure 1a) therefore suggests a population retraction or a degree of under-reporting. Mole density can vary locally quite substantially depending on land use and it is disappointing that the available density estimates are not recent and more widespread. We would therefore expect the true population to be toward the lower end of the model output. However, the predicted habitat suitability appears to be restricted with the true range of the mole more widespread than the model suggests. The extremely large range in the model estimate is disappointing and suggests that even rough density estimation approaches could markedly improve the model output.

References:

Harris, S. J., P. Morris, S. Wray and D. Yalden (1995). A review of British mammals: population estimates and conservation status of British mammals other than cetaceans, Joint Nature Conservation Committee, Peterborough, UK.

Macdonald, D. W., R. P. D. Atkinson and G. Blanchard (1996). Spatial and temporal patterns in the activity of European moles. *Oecologia* 109(1): 88-97.

Stone, R. D. (1986). The social ecology of the European mole, *Talpa europaea* and the Pyrenean desman, *Galemys pyrenaicus*: a comparative study. Ph.D. Thesis, University of Aberdeen.

Table 1: Summary of observed data and model predictions by land cover class (LCM2007 target classification). Values shown in brackets denote the spatial coverage based on a 10km resolution raster map (number of grid cells). Years represent the median of records within each land class. Ranges for density and abundance are derived using the respective minimum and maximum raster maps (lower bound is mean of values across minimum raster map with upper across the maximum) which capture the spatial uncertainty generate by projecting irregular polygons describing survey sites onto a raster grid.

LCM2007 class	Observed					Predicted		
	Occurrence		Density					
	Records	Year	Estimates	Year	Range	Habitat suitability	Density	Abundance
1 (Broadleaved woodland)	304 (11)	2013	0 (0)	-	-	0.95 (11)	0.67 - 584.8	742.4 - 643,230
2 (Coniferous woodland)	2,335 (142)	2010	0 (0)	-	-	0.89 (87)	0.67 - 578.4	5,808 - 5,032,204
3 (Arable and Horticultural)	26,291 (937)	2013	7 (4)	1987	0.67 - 584.8	0.95 (941)	0.62 - 539.8	58,624 - 50,795,818
4 (Improved grassland)	20,081 (719)	2012	0 (0)	-	-	0.9 (679)	0.62 - 533.7	41,824 - 36,239,375
5 (Rough grassland)	260 (28)	2003	0 (0)	-	-	0.46 (12)	0.55 - 477.9	661.8 - 573,438
6 (Neutral grassland)	0 (0)	-	0 (0)	-	-	0.01 (0)	-	0
7 (Calcareous grassland)	56 (2)	2014	0 (0)	-	-	0.97 (2)	0.67 - 584.8	135 - 116,951
8 (Acid grassland)	2,284 (176)	2000	0 (0)	-	-	0.86 (106)	0.67 - 584	7,145 - 6,190,672
9 (Fen, Marsh, and Swamp)	0 (0)	-	0 (0)	-	-	-	-	0
10 (Heather)	545 (49)	2007	0 (0)	-	-	0.86 (38)	0.66 - 574.7	2,521 - 2,183,934
11 (Heather grassland)	1208 (78)	2009	0 (0)	-	-	0.64 (27)	0.67 - 583.2	1,817 - 1,574,560
12 (Bog)	922 (77)	2006	0 (0)	-	-	0.56 (32)	0.66 - 573.3	2,117 - 1,834,499
13 (Montane habitat)	334 (42)	2006	0 (0)	-	-	0.84 (10)	0.67 - 584.8	674.9 - 584,754
14 (Inland rock)	0 (0)	-	0 (0)	-	-	0.36 (0)	-	0
15 (Saltwater)	86 (11)	2008	0 (0)	-	-	0.88 (8)	0.25 - 219.9	203.1 - 175,935
16 (Freshwater)	16 (2)	2006	0 (0)	-	-	0.67 (0)	-	0
17 (Supra-littoral rock)	0 (0)	-	0 (0)	-	-	0.13 (0)	-	0
18 (Supra-littoral sediment)	38 (4)	2012	0 (0)	-	-	0.65 (2)	0.16 - 142.6	32.92 - 28,524
19 (Littoral rock)	8 (2)	2012	0 (0)	-	-	0.46 (1)	0.02 - 14.11	1.63 - 1,411
20 (Littoral sediment)	381 (29)	2007	0 (0)	-	-	0.85 (13)	0.44 - 378.6	568.1 - 492,193
21 (Saltmarsh)	0 (0)	-	0 (0)	-	-	-	-	0
22 (Urban)	116 (6)	1982	0 (0)	-	-	0.85 (1)	0.55 - 473.5	54.65 - 47,352
23 (Suburban)	944 (69)	2009	0 (0)	-	-	0.89 (48)	0.65 - 565.8	3,135 - 2,716,170
Total	56,209 (2,384)	2012	7 (4)	1987	0.67 - 584.8	0.86 (2,018)	0.62 - 541.3	126,065 - 109,231,019

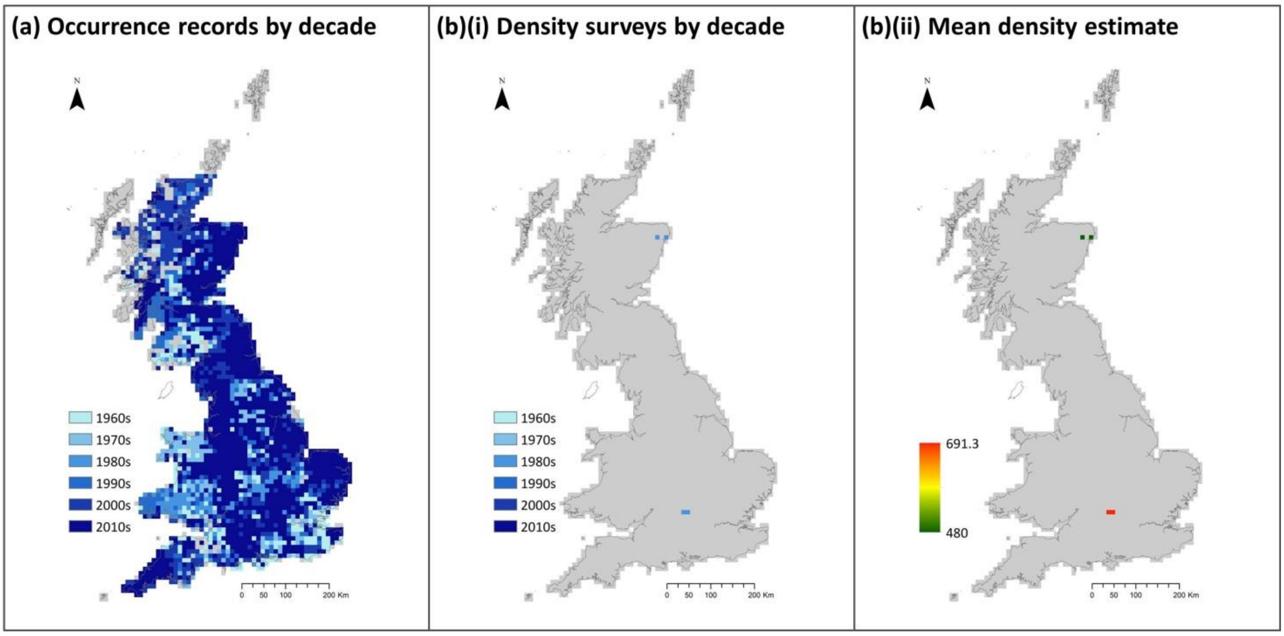

© Crown copyright and database rights 2016 Ordnance Survey 100051110. Data courtesy of the NBN Gateway with thanks to all data contributors. The NBN and its data contributors bear no responsibility for the further analysis or interpretation of this material, data and/or information.

Figure 1: 10km resolution raster maps based on BNG presenting the geographic description of available data. (a) shows the distribution of species occurrence obtained via the NBN Gateway categorised by the decade of last sighting. (b) shows information relating to density surveys identified via a search of published literature where: (i) categorises surveys by the decade of last survey; and (ii) shows the mean density estimate of surveys within grid cells (estimates assumed to be representative of entire cell, considered the upper limit of observed density).

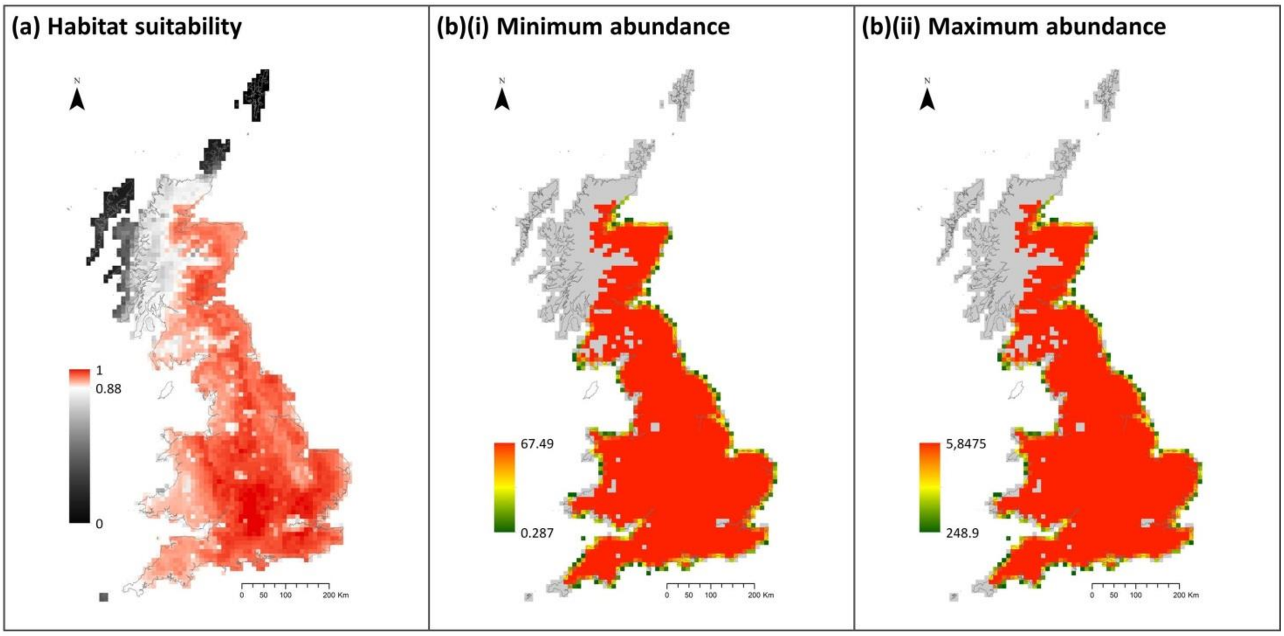

© Crown copyright and database rights 2016 Ordnance Survey 100051110. Data courtesy of the NBN Gateway with thanks to all data contributors. The NBN and its data contributors bear no responsibility for the further analysis or interpretation of this material, data and/or information.

Figure 2: Modelling predictions generated using systematic approach based on available data. (a) shows habitat suitability scores (the likelihood of observing the target species within each grid cell given variation environmental variables) determined by aggregating outputs from the "best" species distribution model (7 models compared) across 100 simulations. Here, the mid value on the scale denotes the threshold score above which occurrence is assumed. (b) shows: (i) the lower bound (Minimum); and (ii) the upper bound (Maximum); of abundance estimates determined by relating observed density (taking into account potential uncertainty) with habitat suitability scores using linear regression.
