# Supplementary material for: A systematic approach to estimate the distribution and total abundance of British mammals
Source: PLoS One. 2017 Jun 28;12(6):e0176339. doi: 10.1371/journal.pone.0176339 (PMC5489149; doi:10.1371/journal.pone.0176339)
Supplement: S6 File — Individual reports for each of the Insectivora species presenting analysis of the available data and subsequent model predictions based on a 10km raster grid. Reports also include expert comment assessing the reliability (and plausibility) of results in the context of existing evidence and popular opinion. (ZIP) [file pone.0176339.s006.zip › E Pygmy shrew.pdf]

Pygmy shrew (*Sorex minutus*)

Order: *Insectivora*

Genus: *Sorex*

Origin: Native

Status: Common

1995 abundance estimate: 8,600,000 (4)

Reported population trends: None

Data:

The available occurrence records indicate that the pygmy shrew is locally distributed throughout GB (Figure 1a). However, the map highlights several regions, particularly in Scotland, where the species has never been reported and other areas, such as in the south east of England, where it has not been reported for some time.

From the literature review we identified several studies (Churchfield & Brown 1987; Kotzageorgis & Mason 1997; Pernetta 1997; Shore et al. 2005; Tattersall et al. 2002) conducted across England between 1972 and 2000 (Figure 1b). Estimates ranged between 0 and 1,309 per km^2 with the highest densities reported in broadleaved woodland dominated land cover (0.12 - 1,309 per km^2 accounting for uncertainty relating to unsurveyed areas within grid cells). Due to the limited coverage of these surveys estimates were unavailable for several dominant land covers where occurrence was reported (marked grey in Table 1) and where estimates were available the relative uncertainty within cells was large.

Model predictions:

The habitat suitability map (Figure 2a) appears to reflect the underlying data reasonably well with the set of "best" models predicting presence (and absence) to a mean AUC of 0.66. Overall, across 100 repetitions MaxEnt proved to be the most commonly selected modelling approach displaying the highest AUC 25% of the time followed by Random Forest (30%). By land cover the mean habitat suitability scores suggest observation is most likely in landscapes dominated by urban and broadleaved woodland land cover but, consistent with recorded sightings, the majority of occurrence is predicted in arable and improved grassland (the most common dominant land covers at a 10km scale).

Perhaps due to the limited number of density estimates linear regression suggested that there was no correlation with habitat suitability. Instead, a constant mean estimate was applied to all cells where occurrence was predicted and summed to derive total abundance.

The predicted abundance range contains the estimate from Harris et al. (1995) suggesting no change in total population (this is perhaps unsurprising as the mean year for density estimation is 1996; the result may instead indicate that any changes in distribution over the past 20 years have not been significant). However, the range is very large due to the uncertainty caused by small survey sites relative to the 10km scale at which modelling is performed. Given the home range of the species it is perhaps reasonable to suggest that the true estimate lies towards the lower end of the range where small isolated populations within cells are assumed unrepresentative of the wider landscape. In order to provide more accurate predictions future model analysis could be based on a finer scale raster grid which would better represent the variations in habitat for smaller mammals. Unfortunately, at present this is too unreliable due to access restrictions imposed on occurrence data.

Reliability (Expert comment):

In a similar pattern to that seen for common shrews, occurrence records appear to suggest an increase in East Anglia, south west England and northern England with a decrease in south east England. Again this could be due to changes in recording effort between regions over time, or a genuine effect. That the same pattern was seen for both species could be a result of effects acting equally on both species. Habitat preferences of pygmy shrews largely overlap with those of the common shrew, which is reflected in the observations of occurrence and model predictions which were similar for the two species; again there were no observations for neutral grassland. Upper limits for predicted density in arable and horticultural habitats and improved grassland were substantially higher than those reported from

published data, but well within the published range for broadleaved woodland (hence the range for predicted total density was close to the range for published data).

References:

Churchfield, S. and V. K. Brown (1987). The trophic impact of small mammals in successional grasslands. *Biological Journal of the Linnean Society* 31(3): 273-290.

Harris, S. J., P. Morris, S. Wray and D. Yalden (1995). A review of British mammals: population estimates and conservation status of British mammals other than cetaceans, Joint Nature Conservation Committee, Peterborough, UK.

Kotzageorgis, G. C. and C. F. Mason (1997). Small mammal populations in relation to hedgerow structure in an arable landscape. *Journal of Zoology* 242(3): 425-434.

Pernetta, J. C. (1977). Population ecology of British shrews in grassland. *Acta Theriologica* 22(20): 279-296.

Shore, R. F., W. R. Meek, T. H. Sparks, R. F. Pywell and M. Nowakowski (2005). Will environmental stewardship enhance small mammal abundance on intensively managed farmland? *Mammal Review* 35(3-4): 277-284.

Tattersall, F. H., D. W. Macdonald, B. J. Hart, P. Johnson, W. Manley and R. Feber (2002). Is habitat linearity important for small mammal communities on farmland? *Journal of Applied Ecology* 39(4): 643-652.

Table 1: Summary of observed data and model predictions by land cover class (LCM2007 target classification). Values shown in brackets denote the spatial coverage based on a 10km resolution raster map (number of grid cells). Years represent the median of records within each land class. Ranges for density and abundance are derived using the respective minimum and maximum raster maps (lower bound is mean of values across minimum raster map with upper across the maximum) which capture the spatial uncertainty generate by projecting irregular polygons describing survey sites onto a raster grid.

LCM2007 class	Observed					Predicted		
	Occurrence		Density					
	Records	Year	Estimates	Year	Range	Habitat suitability	Density	Abundance
1 (Broadleaved woodland)	56 (7)	1968	1 (1)	1986	0.12 - 1,309	0.88 (11)	0.08 - 265.3	93.2 - 291,807
2 (Coniferous woodland)	115 (61)	1994	0 (0)	-	-	0.7 (40)	0.08 - 236.9	302.7 - 947,643
3 (Arable and Horticultural)	2,810 (572)	1991	7 (4)	1996	0.07 - 3.07	0.87 (821)	0.08 - 243	6,373 - 19,952,102
4 (Improved grassland)	1,301 (410)	1986	2 (1)	1996	0.02 - 5.49	0.82 (514)	0.08 - 238.2	3,911 - 12,244,460
5 (Rough grassland)	40 (17)	1994	0 (0)	-	-	0.49 (8)	0.04 - 137.7	35.19 - 110,165
6 (Neutral grassland)	0 (0)	-	0 (0)	-	-	0.02 (0)	-	0
7 (Calcareous grassland)	8 (2)	2013	0 (0)	-	-	0.92 (2)	0.08 - 265.3	16.95 - 53,056
8 (Acid grassland)	76 (41)	1988	0 (0)	-	-	0.5 (1)	0.08 - 265.3	8.47 - 26,528
9 (Fen, Marsh, and Swamp)	0 (0)	-	0 (0)	-	-	-	-	0
10 (Heather)	46 (25)	1988	0 (0)	-	-	0.7 (14)	0.07 - 216.8	96.92 - 303,453
11 (Heather grassland)	123 (40)	1994	0 (0)	-	-	0.58 (9)	0.05 - 171.1	49.19 - 154,015
12 (Bog)	65 (35)	1985	0 (0)	-	-	0.51 (5)	0.08 - 262.1	41.86 - 131,069
13 (Montane habitat)	23 (11)	1994	0 (0)	-	-	0.46 (0)	-	0
14 (Inland rock)	2 (1)	1975	0 (0)	-	-	0.74 (0)	-	0
15 (Saltwater)	27 (4)	1985	0 (0)	-	-	0.73 (2)	0.06 - 181.7	11.61 - 36,341
16 (Freshwater)	3 (2)	1996	0 (0)	-	-	0.75 (0)	-	0
17 (Supra-littoral rock)	0 (0)	-	0 (0)	-	-	0.21 (0)	-	0
18 (Supra-littoral sediment)	13 (3)	1973	0 (0)	-	-	0.71 (2)	0.02 - 64.7	4.13 - 12,940
19 (Littoral rock)	0 (0)	-	0 (0)	-	-	0.55 (1)	0 - 0.84	0.03 - 83.73
20 (Littoral sediment)	79 (22)	1991	0 (0)	-	-	0.84 (28)	0.04 - 138.9	124.2 - 388,867
21 (Saltmarsh)	0 (0)	-	0 (0)	-	-	-	-	0
22 (Urban)	17 (6)	1976	0 (0)	-	-	0.88 (7)	0.05 - 166.9	37.31 - 116,825
23 (Suburban)	221 (50)	1972	0 (0)	-	-	0.87 (68)	0.07 - 212.4	461.2 - 1,444,086
Total	5,025 (1,309)	1988	10 (6)	1996	0.07 - 189.9	0.76 (1,533)	0.08 - 236.2	11,566 - 36,213,441

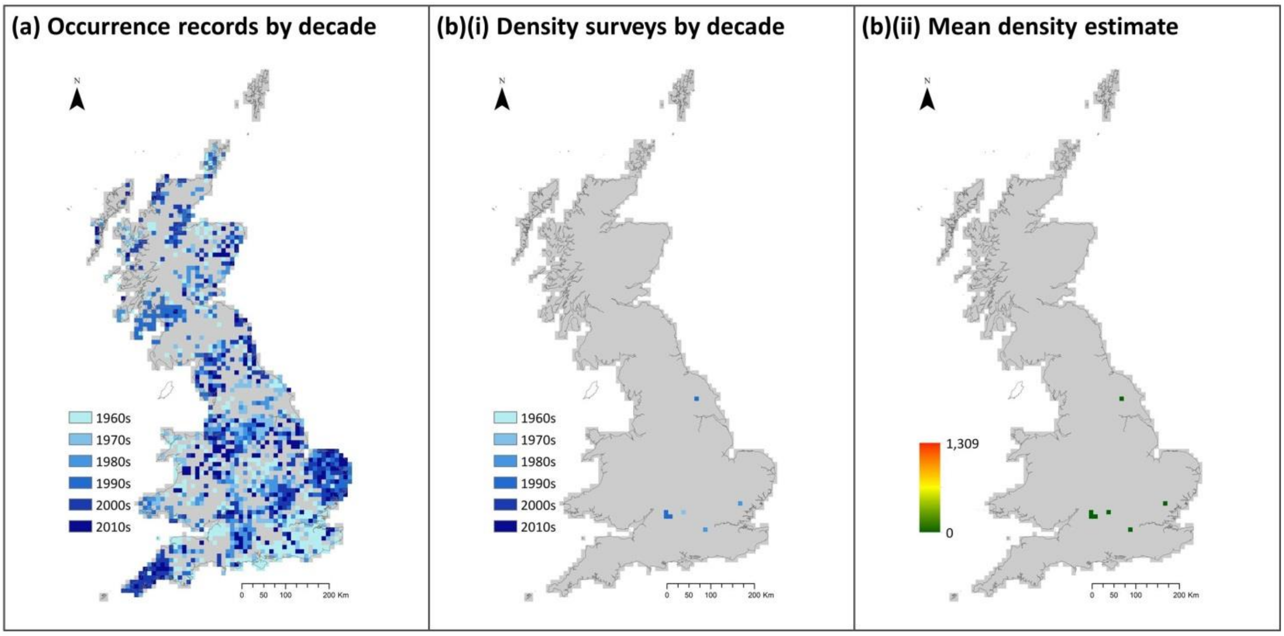

© Crown copyright and database rights 2016 Ordnance Survey 100051110. Data courtesy of the NBN Gateway with thanks to all data contributors. The NBN and its data contributors bear no responsibility for the further analysis or interpretation of this material, data and/or information.

Figure 1: 10km resolution raster maps based on BNG presenting the geographic description of available data. (a) shows the distribution of species occurrence obtained via the NBN Gateway categorised by the decade of last sighting. (b) shows information relating to density surveys identified via a search of published literature where: (i) categorises surveys by the decade of last survey; and (ii) shows the mean density estimate of surveys within grid cells (estimates assumed to be representative of entire cell, considered the upper limit of observed density).

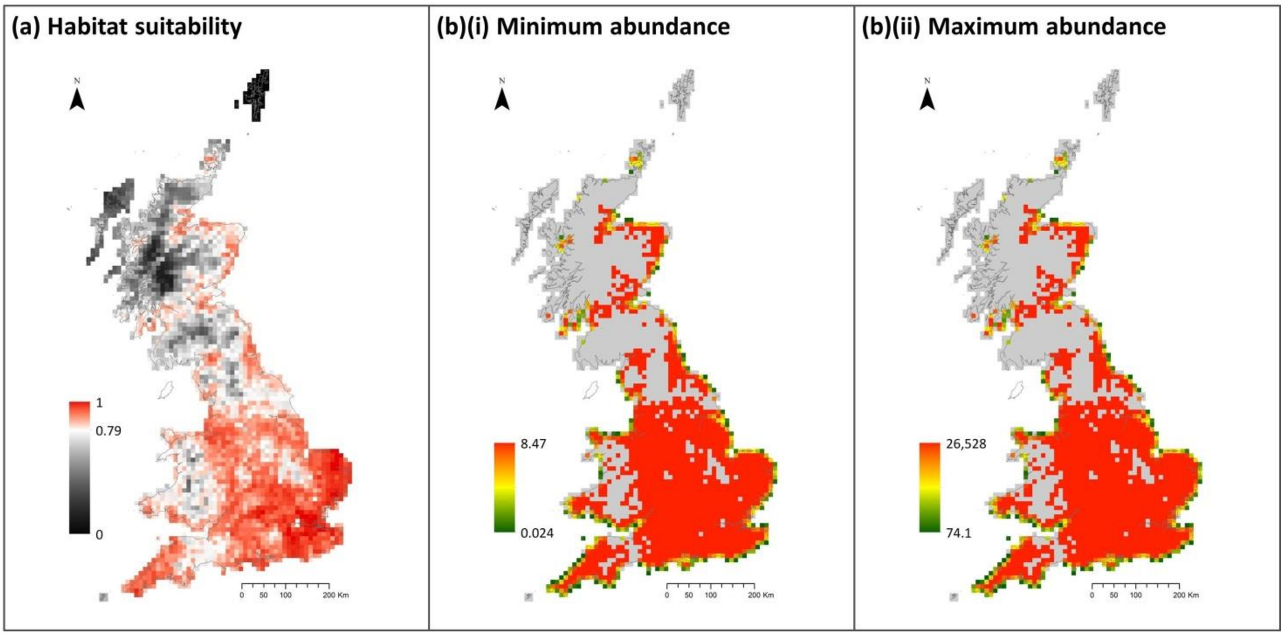

© Crown copyright and database rights 2016 Ordnance Survey 100051110. Data courtesy of the NBN Gateway with thanks to all data contributors. The NBN and its data contributors bear no responsibility for the further analysis or interpretation of this material, data and/or information.

Figure 2: Modelling predictions generated using systematic approach based on available data. (a) shows habitat suitability scores (the likelihood of observing the target species within each grid cell given variation environmental variables) determined by aggregating outputs from the "best" species distribution model (7 models compared) across 100 simulations. Here, the mid value on the scale denotes the threshold score above which occurrence is assumed. (b) shows: (i) the lower bound (Minimum); and (ii) the upper bound (Maximum); of abundance estimates determined by relating observed density (taking into account potential uncertainty) with habitat suitability scores using linear regression.
