# Supplementary material for: A systematic approach to estimate the distribution and total abundance of British mammals
Source: PLoS One. 2017 Jun 28;12(6):e0176339. doi: 10.1371/journal.pone.0176339 (PMC5489149; doi:10.1371/journal.pone.0176339)
Supplement: S6 File — Individual reports for each of the Insectivora species presenting analysis of the available data and subsequent model predictions based on a 10km raster grid. Reports also include expert comment assessing the reliability (and plausibility) of results in the context of existing evidence and popular opinion. (ZIP) [file pone.0176339.s006.zip › F Water shrew.pdf]

Water shrew (*Neomys fodiens*)

Order: *Insectivora*

Genus: *Neomys*

Origin: Native

Status: Locally common

1995 abundance estimate: 1,900,000 (4)

Reported population trends: None

Data:

The available occurrence records indicate that the water shrew is locally distributed throughout GB with patches of more widespread occurrence in East Anglia, Cornwall and the north west of England (Figure 1a). However, the map does highlight several areas, primarily the south east of England, where the species has not be reported for some time, and other areas, mainly in Scotland and Wales, where the it has never been reported.

From the literature review we identified a limited number of studies (Kotzageorgis & Mason 1997; Shore et al. 2005) conducted on farmland in England (one in Yorkshire and the other in East Anglia) between 1990 and 2000 (Figure 1b). Estimates ranged between 2.91 and 3.14 per km^2. Due to the limited coverage of these surveys estimates were unavailable for all dominant land covers where occurrence was reported (marked grey in Table 1) with the exception of arable.

Model predictions:

The habitat suitability map (Figure 2a) appears to reflect the underlying data reasonably well with the set of "best" models predicting presence (and absence) to a mean AUC of 0.74. However, the resulting distribution is substantially larger than the area described by the observations (approximately 1.5 times). Overall, across 100 repetitions MaxEnt proved to be the most commonly selected modelling approach displaying the highest AUC 49% of the time followed by Random Forest (26%) and Generalised Linear Models (12%). By land cover the mean habitat suitability scores suggest observation is most likely in landscapes dominated by calcareous grassland (despite few observations; Table 1) but, consistent with recorded sightings, the majority of occurrence is predicted in arable and improved grassland (the most common dominant land covers at a 10km scale).

Both minimum and maximum density estimates were best fitted linearly to habitat suitability accounting for spherical spatial autocorrelation. However, the relationship with maximum density appear to be negative relating the highest densities to cells where suitability was low. This result may perhaps explain the rather small estimate for the upper bound of total abundance.

As a consequence the predicted range of abundance does not contain the estimate from Harris et al. (1995) instead suggesting a significant decrease in the total population. In order to resolve the differences between model outputs and provide more accurate predictions future model analysis could be based on a finer scale raster grid which would better represent the variations in habitat for smaller mammals. Unfortunately, at present this is too unreliable due to access restrictions imposed on occurrence data.

Reliability (Expert comment):

Apart from a greater affinity for wetland habitats, it has been reported that habitat preferences of water shrews largely overlap with those of common and pygmy shrews (Rychlik 2000). This could explain why recent changes in the distribution of occurrence records are similar for all three of these species; increases in East Anglia, south west England and northern England and a decrease in south east England could reflect genuine increases in those regions due to underlying factors acting equally on all three species. Again, an alternative explanation is changes in recording effort between regions over time; however it is important to note that this would be unlikely to affect the model predictions as habitat preference of individual species is unlikely to differ between regions. It is slightly surprising that the model predicted lower suitability of bog and freshwater habitats for water shrews compared to common and pygmy shrews; there were no observed records for fen, marsh and swamp habitats for any of the shrew species considered here.

References:

Harris, S. J., P. Morris, S. Wray and D. Yalden (1995). A review of British mammals: population estimates and conservation status of British mammals other than cetaceans, Joint Nature Conservation Committee, Peterborough, UK.

Kotzageorgis, G. C. and C. F. Mason (1997). Small mammal populations in relation to hedgerow structure in an arable landscape. *Journal of Zoology* 242(3): 425-434.

Rychlik L. (2000). Habitat preferences of four sympatric species of shrews. *Acta Theriologica* 45: 173-190.

Shore, R. F., W. R. Meek, T. H. Sparks, R. F. Pywell and M. Nowakowski (2005). Will environmental stewardship enhance small mammal abundance on intensively managed farmland? *Mammal Review* 35(3-4): 277-284.

Table 1: Summary of observed data and model predictions by land cover class (LCM2007 target classification). Values shown in brackets denote the spatial coverage based on a 10km resolution raster map (number of grid cells). Years represent the median of records within each land class. Ranges for density and abundance are derived using the respective minimum and maximum raster maps (lower bound is mean of values across minimum raster map with upper across the maximum) which capture the spatial uncertainty generate by projecting irregular polygons describing survey sites onto a raster grid.

LCM2007 class	Observed					Predicted		
	Occurrence		Density					
	Records	Year	Estimates	Year	Range	Habitat suitability	Density	Abundance
1 (Broadleaved woodland)	44 (6)	1971	0 (0)	-	-	0.79 (9)	0.1 - 3.07	91.57 - 2,767
2 (Coniferous woodland)	94 (53)	1994	0 (0)	-	-	0.64 (48)	0.11 - 2.85	512.5 - 13,662
3 (Arable and Horticultural)	2,143 (497)	2000	2 (2)	1995	0.14 - 3.03	0.83 (820)	0.16 - 2.72	13,319 - 223,116
4 (Improved grassland)	1,049 (335)	1993	0 (0)	-	-	0.76 (499)	0.14 - 2.72	6,836 - 135,911
5 (Rough grassland)	27 (14)	1990	0 (0)	-	-	0.37 (12)	0.07 - 2.01	84.95 - 2,413
6 (Neutral grassland)	0 (0)	-	0 (0)	-	-	0.01 (0)	-	-
7 (Calcareous grassland)	2 (2)	1998	0 (0)	-	-	0.95 (2)	0.28 - 2.83	56.38 - 565.9
8 (Acid grassland)	71 (37)	1997	0 (0)	-	-	0.45 (5)	0.05 - 3.14	25.93 - 1,571
9 (Fen, Marsh, and Swamp)	0 (0)	-	0 (0)	-	-	-	-	-
10 (Heather)	11 (10)	1996	0 (0)	-	-	0.49 (1)	0 - 0.01	0.03 - 0.86
11 (Heather grassland)	95 (38)	2004	0 (0)	-	-	0.49 (25)	0.06 - 2.32	159.6 - 5,788
12 (Bog)	32 (19)	1994	0 (0)	-	-	0.33 (6)	0.13 - 3.04	75.94 - 1,825
13 (Montane habitat)	5 (4)	1982	0 (0)	-	-	0.25 (0)	-	-
14 (Inland rock)	0 (0)	-	0 (0)	-	-	0.21 (0)	-	-
15 (Saltwater)	17 (4)	2008	0 (0)	-	-	0.69 (3)	0.04 - 1.47	10.84 - 440.4
16 (Freshwater)	1 (1)	1981	0 (0)	-	-	0.46 (1)	0.1 - 3.03	9.5 - 302.8
17 (Supra-littoral rock)	0 (0)	-	0 (0)	-	-	0.05 (0)	-	-
18 (Supra-littoral sediment)	5 (2)	1988	0 (0)	-	-	0.5 (2)	0.01 - 0.41	1.73 - 81.6
19 (Littoral rock)	0 (0)	-	0 (0)	-	-	0.32 (2)	0 - 0.04	0.09 - 8.64
20 (Littoral sediment)	56 (11)	1996	0 (0)	-	-	0.67 (11)	0.05 - 2.34	54.24 - 2,575
21 (Saltmarsh)	0 (0)	-	0 (0)	-	-	-	-	-
22 (Urban)	20 (3)	2004	0 (0)	-	-	0.66 (3)	0.02 - 1.34	7.04 - 401.4
23 (Suburban)	174 (44)	1984	0 (0)	-	-	0.81 (57)	0.16 - 2.59	920.5 - 14,759
Total	3,846 (1,080)	1996	2 (2)	1995	0.14 - 3.03	0.68 (1,506)	0.15 - 2.7	22,166 - 406,189

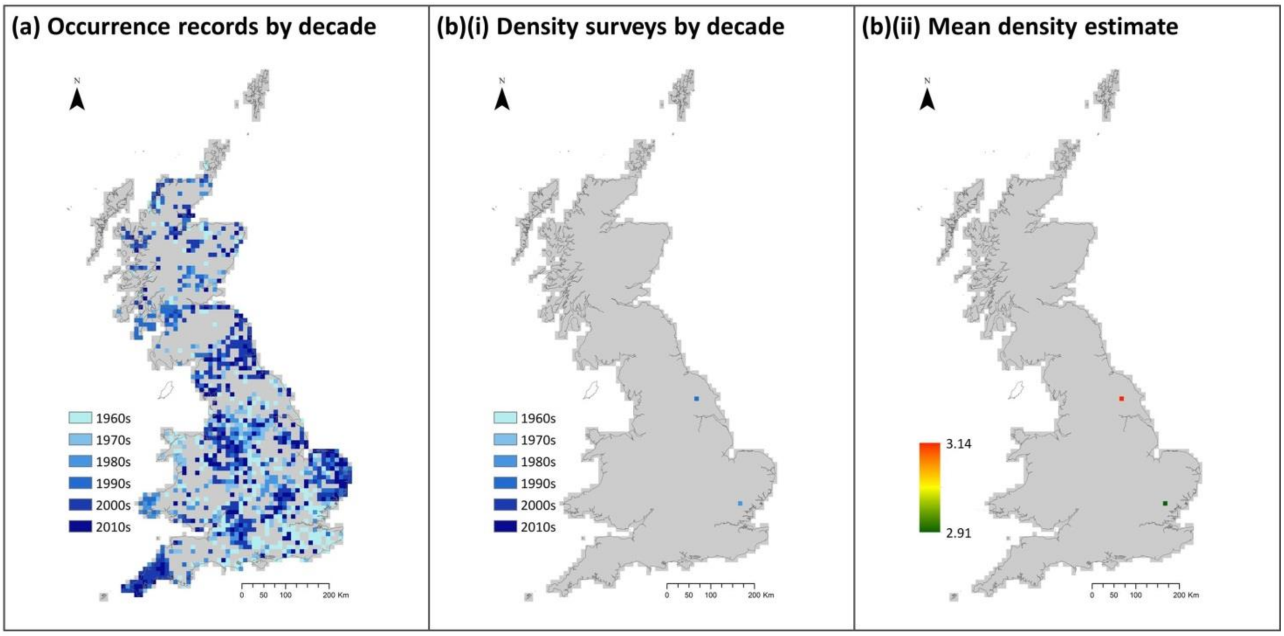

© Crown copyright and database rights 2016 Ordnance Survey 100051110. Data courtesy of the NBN Gateway with thanks to all data contributors. The NBN and its data contributors bear no responsibility for the further analysis or interpretation of this material, data and/or information.

Figure 1: 10km resolution raster maps based on BNG presenting the geographic description of available data. (a) shows the distribution of species occurrence obtained via the NBN Gateway categorised by the decade of last sighting. (b) shows information relating to density surveys identified via a search of published literature where: (i) categorises surveys by the decade of last survey; and (ii) shows the mean density estimate of surveys within grid cells (estimates assumed to be representative of entire cell, considered the upper limit of observed density).

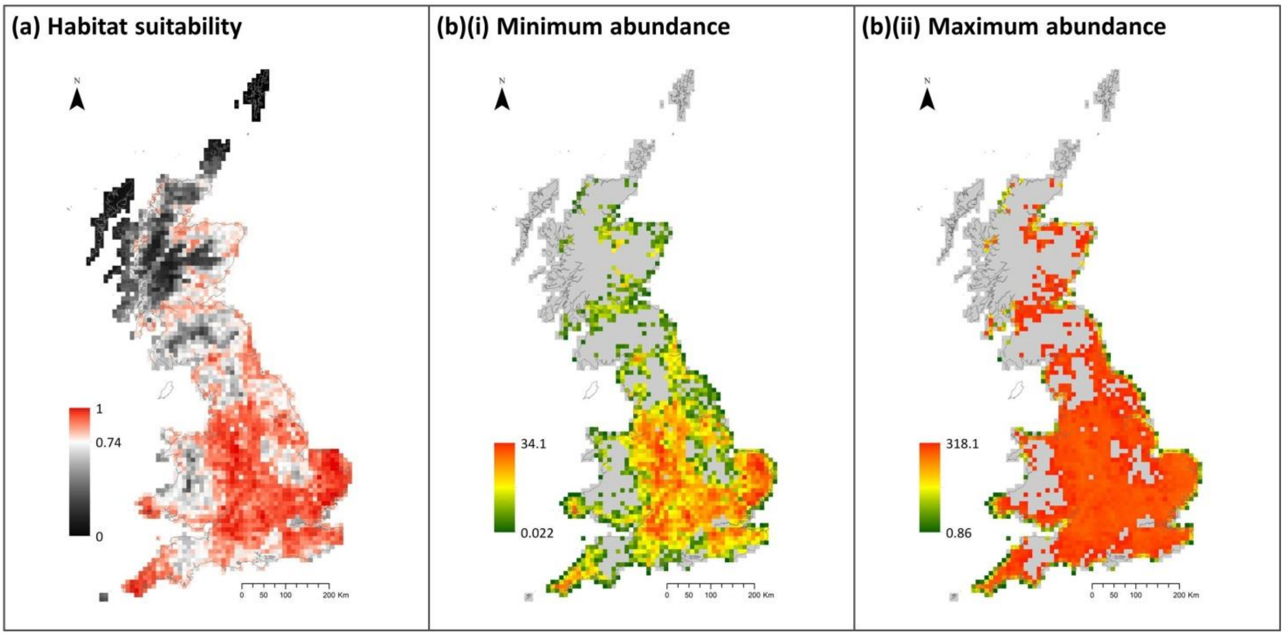

© Crown copyright and database rights 2016 Ordnance Survey 100051110. Data courtesy of the NBN Gateway with thanks to all data contributors. The NBN and its data contributors bear no responsibility for the further analysis or interpretation of this material, data and/or information.

Figure 2: Modelling predictions generated using systematic approach based on available data. (a) shows habitat suitability scores (the likelihood of observing the target species within each grid cell given variation environmental variables) determined by aggregating outputs from the "best" species distribution model (7 models compared) across 100 simulations. Here, the mid value on the scale denotes the threshold score above which occurrence is assumed. (b) shows: (i) the lower bound (Minimum); and (ii) the upper bound (Maximum); of abundance estimates determined by relating observed density (taking into account potential uncertainty) with habitat suitability scores using linear regression.
