# Supplementary material for: A systematic approach to estimate the distribution and total abundance of British mammals
Source: PLoS One. 2017 Jun 28;12(6):e0176339. doi: 10.1371/journal.pone.0176339 (PMC5489149; doi:10.1371/journal.pone.0176339)
Supplement: S7 File — Individual reports for each of the Lagomorpha species presenting analysis of the available data and subsequent model predictions based on a 10km raster grid. Reports also include expert comment assessing the reliability (and plausibility) of results in the context of existing evidence and popular opinion. (ZIP) [file pone.0176339.s007.zip › A Brown hare.pdf]

Brown hare (*Lepus europaeus*)

Order: *Lagomorpha* **Genus:** *Lepus*

Origin: Introduced **Status:** Common

1995 abundance estimate: 817,500 (2) **Reported population trends:** JNCC 2005 (↔), NGC 2009 (↑), BBS 2014 (↔)

Data:

The available occurrence records indicate that the brown hare is widespread throughout GB with sightings reported in most 10 km squares (approximately 77%) at least once over the past decade (Figure 1a). However, the map highlights several areas, particularly in Scotland and around London, where the species has not been recorded for some time or not at all.

Density estimates, primarily recorded over the past two decades, were obtained from published literature spanning approximately 16% of the observed species distribution based on the available occurrence data (Forman 2005; Heydon et al. 2000; Langbein et al. 1999; Parrott et al. 2012; Reynolds et al. 2010; Smith et al. 2004; Tapper & Barnes 1986). Geographically, these studies were well spread across the distribution of observed occurrence with the notable omission of south east England (Figure 1b). Estimates ranged between 0 and 77.3 per km^2 with the highest densities recorded in habitats dominated by calcareous grassland (0.08 - 15.8 per km^2 accounting for uncertainty relating to unsurveyed areas within grid cells). Despite the relatively high proportion of area surveyed estimates for several dominant land covers were not available (land class marked grey in Table 1).

Model predictions:

The habitat suitability map (Figure 2a) appears to reflect the underlying data well with the set of "best" models predicting presence (and absence) to a mean AUC of 0.82. Overall, across 100 repetitions MaxEnt proved to be the most commonly selected modelling approach displaying the highest AUC 36% of the time followed by Random Forest (27%) and Generalised Linear Models (13%). By land cover the mean habitat suitability scores suggest observation is most likely in landscapes dominated by calcareous grassland (Table 1) but, consistent with recorded sightings, the majority of occurrence is predicted in grid cells dominated by arable and improved grassland (the most common dominant land covers at a 10km scale).

Both minimum and maximum density estimates were best fitted to the square of habitat suitability accounting for spherical spatial autocorrelation. However, interestingly the relationship for maximum density shows a negative correlation, predicting higher densities in cells with low suitability. This inconsistency could perhaps be due to the broad scale reporting of density for the species which lacks the spatial variability required to determine meaningful associations with habitat (although the mean statistics for each land cover do not support).

Nevertheless, the predicted abundance range contains the estimate from Harris et al. (1995) which in agreement with recent trends (JNCC, BBS) suggests no significant change in the total population. As is the case for most species the range is large due to the disparity between survey areas and the 10km raster grid upon which models are based.

Reliability (Expert comment):

Tapper & Stoate (1992) estimated that the pre-breeding adult brown hare population was approximately 1 million based on an estimated annual game bag of 390,000 hares with this representing, on average, 40% of the total population. Hutchings & Harris (1996) suggested that, using these data but making alternative assumptions regarding the proportion of hares killed and density estimates, the February population in the early 1990s was approximately 425,000. Robertson et al. (1989) derived estimates based on hare density, using Barnes & Tapper's (1985) night count methodology, in areas with and without gamekeepers. This suggested estimates of between 1,250,000 and 1,900,000 hares overwinter in Britain, the range reflecting assumptions about the impact of game keeping on hare densities. Hutchings & Harris (1996) point out that such estimates are based on a small number of sampled sites, all in England and areas with low hare densities probably being under represented. The 1991-1993 National Hare Survey was thus

undertaken using the line transect approach described by Langbein et al. (1996) with a stratified random sampling process in relation to the ITE land classification scheme. This generated a broadly accepted estimate of 817,520 (+95% confidence interval of 137,251) hares present overwinter (Hutchings & Harris 1996). The survey was repeated during the winters of 1997-1999. This was briefly reported on by White et al. (2000) who quoted a national estimate of 752,608±37,697 hares. There thus appeared to have been no statistically significant overall change in hare numbers during the 1990s although White et al. (2000) state that there were significant reductions in arable areas in southern England and northeast England and eastern Scotland. The NGC however showed a significant increase in brown hare abundance between 1995 and 2009. The BBS suggested no significant change in abundance between 1995 and 2014. The model's predictions are thus consistent with no recent significant changes in brown hare abundance.

The model's predictions thus fall within the range indicated by the surveys and NGC bags with no significant recent changes in the total UK population. It is perhaps surprising that there is apparently no suitable habitat at all in northwest Scotland. Brown hares should be more than capable of surviving in the Machair, at least. It might be that the model is artificially truncating the niche, which can easily happen with insular species given the general consistency of the landscape. The minimum abundance map appears realistic. While there are potential issues with the inclusion of some land class data due to ecological relevance (e.g. saltwater), it is appreciated that the cells are quite large and it would be undesirable for cross-species consistency to remove any land classes. Otherwise the land class values describe a generalist species, which the brown hare is.

References:

Forman, D. W. (2005). An assessment of the local impact of native predators on an established population of British water voles (*Arvicola terrestris*). *Journal of Zoology* 266(3): 221-226.

Harris, S. J., P. Morris, S. Wray and D. Yalden (1995). A review of British mammals: population estimates and conservation status of British mammals other than cetaceans, Joint Nature Conservation Committee, Peterborough.

Heydon, M. J., J. C. Reynolds and M. J. Short (2000). Variation in abundance of foxes (*Vulpes vulpes*) between three regions of rural Britain, in relation to landscape and other variables. *Journal of Zoology* 251(2): 253-264.

Hutchings, M. R. and S. Harris (1996) The current status of the brown hare (Lepus europaeus) in Britain. Joint Nature Conservation Committee, Peterborough, UK.

Langbein, J., M. R. Hutchings, S. Harris, C. Stoate, S. C. Tapper and S. Wray (1999). Techniques for assessing the abundance of brown hares *Lepus europaeus*. *Mammal Review* 29(2): 93-116.

Parrott, D., A. Prickett, S. Pietravalle, T. R. Etherington and M. Fletcher (2012). Estimates of regional population densities of badger *Meles meles*, fox *Vulpes vulpes* and hare *Lepus europaeus* using walked distance sampling. *European Journal of Wildlife Research* 58(1): 23-33.

Reynolds, J. C., C. Stoate, M. H. Brockless, N. J. Aebischer and S. C. Tapper (2010). The consequences of predator control for brown hares (*Lepus europaeus*) on UK farmland. *European Journal of Wildlife Research* 56(4): 541-549.

Robertson P. A., M. I. A. Woodburn, S. C. Tapper and C. Stoate (1989) Estimating game densities in Britain from land-use maps. Institute of Terrestrial Ecology, Grange-over-Sands, UK.

Smith, R. K., N. V. Jennings, A. Robinson and S. Harris (2004). Conservation of European hares *Lepus europaeus* in Britain: is increasing habitat heterogeneity in farmland the answer? *Journal of Applied Ecology* 41(6): 1092-1102.

Tapper, S. C. and R. F. W. Barnes (1986). Influence of Farming Practice on the Ecology of the brown hare (*Lepus europaeus*). *Journal of Applied Ecology* 23(1): 39-52.

Tapper S. and C. Stoate (1994) Hares - the game management connection. *Game Conservancy Review* 25, 63 64.

White P., P. Baker, G. Newton Cross, J. Smart, R. Moberly, G. McLaren, R. Ansell and S. Harris (2000) Report on Contract 5: Management of the population of foxes, deer, hares and mink and the impact of hunting with dogs and Report on Contract 6: Methods of controlling foxes, deer, hare and mink for Lord Burns' committee of inquiry into hunting with dogs. Report of the Committee of Inquiry into Hunting with Dogs (by T. Burns, V. Edwards, J. Marsh, L. Soulsby and M. Winter). The Stationary Office, Norwich, UK.

Table 1: Summary of observed data and model predictions by land cover class (LCM2007 target classification). Values shown in brackets denote the spatial coverage based on a 10km resolution raster map (number of grid cells). Years represent the median of records within each land class. Ranges for density and abundance are derived using the respective minimum and maximum raster maps (lower bound is mean of values across minimum raster map with upper across the maximum) which capture the spatial uncertainty generate by projecting irregular polygons describing survey sites onto a raster grid.

LCM2007 class	Observed					Predicted		
	Occurrence		Density					
	Records	Year	Estimates	Year	Range	Habitat suitability	Density	Abundance
1 (Broadleaved woodland)	119 (8)	2008	0 (0)	-	-	0.86 (11)	0.13 - 18.75	139.6 - 20,629
2 (Coniferous woodland)	868 (117)	2006	3 (3)	2006	0.17 - 3.93	0.82 (92)	0.37 - 17.08	3,446 - 157,126
3 (Arable and Horticultural)	34,688 (927)	2013	196 (179)	2006	3.69 - 9.67	0.93 (958)	0.67 - 16.47	64,121 - 1,578,115
4 (Improved grassland)	14,552 (686)	2011	147 (134)	2006	0.36 - 2.73	0.91 (707)	0.62 - 16.14	43,721 - 1,140,844
5 (Rough grassland)	301 (22)	1996	0 (0)	-	-	0.48 (13)	0.13 - 14.12	172.1 - 18,356
6 (Neutral grassland)	0 (0)	-	0 (0)	-	-	0 (0)	-	0
7 (Calcareous grassland)	165 (2)	2014	1 (1)	1990	0.08 - 15.8	0.99 (2)	1.61 - 17.35	322.3 - 3,471
8 (Acid grassland)	890 (117)	2007	14 (12)	2006	0.22 - 2.08	0.71 (100)	0.3 - 18.22	2,955 - 182,248
9 (Fen, Marsh, and Swamp)	0 (0)	-	0 (0)	-	-	-	-	0
10 (Heather)	253 (43)	2006	0 (0)	-	-	0.79 (30)	0.63 - 18.12	1,892 - 54,355
11 (Heather grassland)	347 (42)	2001	0 (0)	-	-	0.52 (17)	0.09 - 18.76	152.2 - 31,891
12 (Bog)	605 (55)	2003	2 (2)	2006	0.13 - 3.66	0.53 (27)	0.45 - 18.25	1,219 - 49,275
13 (Montane habitat)	137 (23)	2004	0 (0)	-	-	0.65 (6)	0.16 - 18.67	93.02 - 11,204
14 (Inland rock)	0 (0)	-	0 (0)	-	-	0.27 (0)	-	0
15 (Saltwater)	83 (5)	2012	1 (1)	1990	0.16 - 3.7	0.73 (0)	-	0
16 (Freshwater)	4 (2)	1997	0 (0)	-	-	0.69 (2)	0.13 - 18.57	25.48 - 3,713
17 (Supra-littoral rock)	0 (0)	-	0 (0)	-	-	0.13 (0)	-	0
18 (Supra-littoral sediment)	25 (3)	2013	1 (1)	2006	0.39 - 0.51	0.64 (3)	0.02 - 3.61	5.52 - 1,082
19 (Littoral rock)	0 (0)	-	0 (0)	-	-	0.46 (0)	-	0
20 (Littoral sediment)	496 (28)	2008	2 (2)	2006	0.33 - 4.58	0.84 (30)	0.05 - 12	152.8 - 35,999
21 (Saltmarsh)	0 (0)	-	0 (0)	-	-	-	-	0
22 (Urban)	242 (6)	1986	0 (0)	-	-	0.82 (5)	0.04 - 9.06	21.27 - 4,528
23 (Suburban)	1,117 (65)	2006	1 (1)	2006	0 - 0.43	0.83 (56)	0.07 - 17.97	390.8 - 100,608
Total	54,892 (2,151)	2011	368 (336)	2006	2.12 - 6.44	0.83 (2,059)	0.58 - 16.48	118,829 - 3,393,442

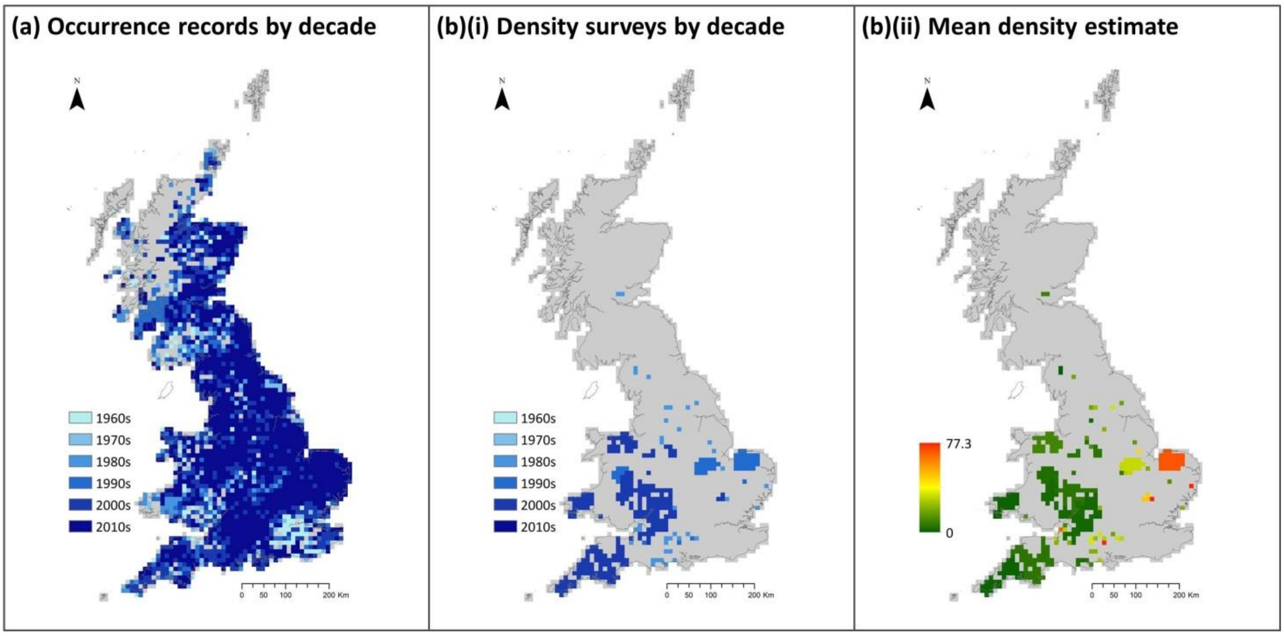

© Crown copyright and database rights 2016 Ordnance Survey 100051110. Data courtesy of the NBN Gateway with thanks to all data contributors. The NBN and its data contributors bear no responsibility for the further analysis or interpretation of this material, data and/or information.

Figure 1: 10km resolution raster maps based on BNG presenting the geographic description of available data. (a) shows the distribution of species occurrence obtained via the NBN Gateway categorised by the decade of last sighting. (b) shows information relating to density surveys identified via a search of published literature where: (i) categorises surveys by the decade of last survey; and (ii) shows the mean density estimate of surveys within grid cells (estimates assumed to be representative of entire cell, considered the upper limit of observed density).

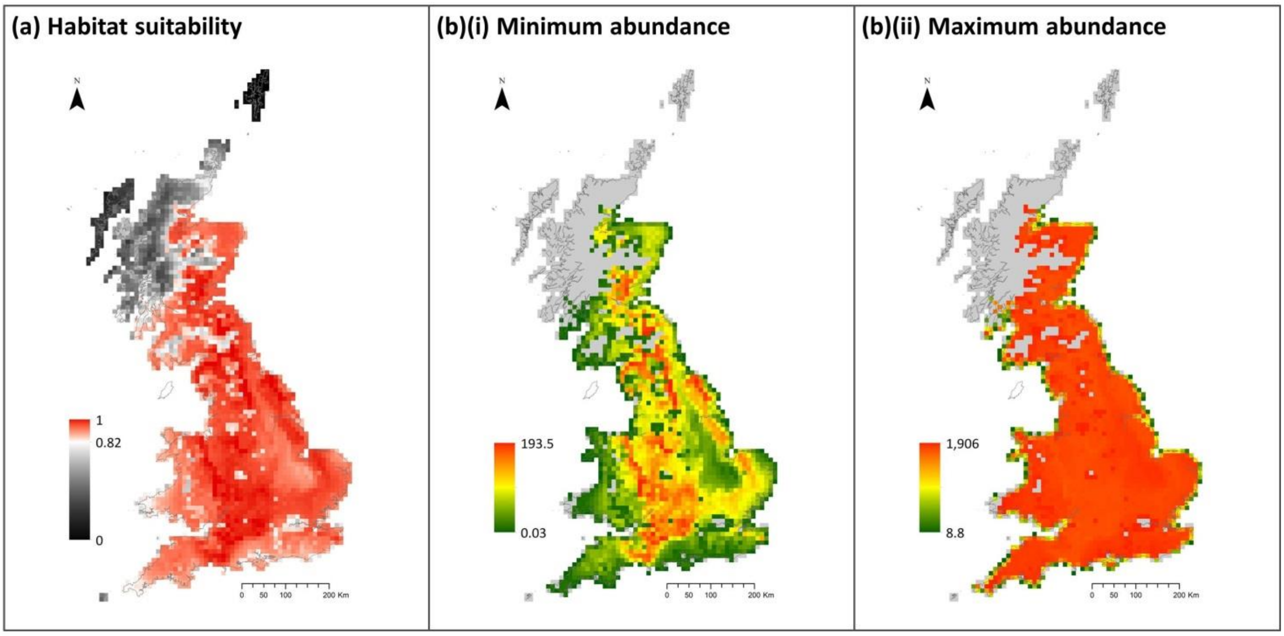

© Crown copyright and database rights 2016 Ordnance Survey 100051110. Data courtesy of the NBN Gateway with thanks to all data contributors. The NBN and its data contributors bear no responsibility for the further analysis or interpretation of this material, data and/or information.

Figure 2: Modelling predictions generated using systematic approach based on available data. (a) shows habitat suitability scores (the likelihood of observing the target species within each grid cell given variation environmental variables) determined by aggregating outputs from the "best" species distribution model (7 models compared) across 100 simulations. Here, the mid value on the scale denotes the threshold score above which occurrence is assumed. (b) shows: (i) the lower bound (Minimum); and (ii) the upper bound (Maximum); of abundance estimates determined by relating observed density (taking into account potential uncertainty) with habitat suitability scores using linear regression.
