# Supplementary material for: A systematic approach to estimate the distribution and total abundance of British mammals
Source: PLoS One. 2017 Jun 28;12(6):e0176339. doi: 10.1371/journal.pone.0176339 (PMC5489149; doi:10.1371/journal.pone.0176339)
Supplement: S7 File — Individual reports for each of the Lagomorpha species presenting analysis of the available data and subsequent model predictions based on a 10km raster grid. Reports also include expert comment assessing the reliability (and plausibility) of results in the context of existing evidence and popular opinion. (ZIP) [file pone.0176339.s007.zip › B Mountain hare.pdf]

Mountain hare (*Lepus timidus*)

Order: *Lagomorpha*

Genus: *Lepus*

Origin: Native

Status: Locally common

1995 abundance estimate: 350,000 (3)

Reported population trends: JNCC 2005 (↓), NGC 2009, BBS 2014 (↔)

Data:

The available occurrence indicate that mountain hare are most commonly sighted in Scotland with some scattered patches in England and Wales, the most notable of which is located in the Pennies (Figure 1a). Sightings were reported in various habitats (predominantly coniferous woodland and acid grassland) with the majority of cells where occurrence was observed containing at least one record since 1995.

From the literature review we identified a single study (Watson et al. 1973) conducted on Scottish moorland between 1961 and 1971 (Figure 1b). Estimates were variable ranging between 6.25 and 57.62 per km^2 with the highest densities recorded in habitats dominated by heather (0 - 57.62 per km^2 accounting for uncertainty relating to unsurveyed areas within grid cells). Unfortunately, these surveys only sampled a limited selection of dominant land covers, consequently estimates were unavailable for some habitats where occurrence was observed notably coniferous woodland (marked grey in Table 1).

Model predictions:

The habitat suitability map (Figure 2a) appears to reflect the underlying data well with the set of "best" models predicting presence (and absence) to a mean AUC of 0.77. However, the patchy occurrence observed in England is not well captured. Overall, across 100 repetitions Generalised Linear Models proved to be the most commonly selected modelling approach displaying the highest AUC 28% of the time closely followed by Random Forest (25%). By land cover the mean habitat suitability scores suggest observation is most likely in landscapes dominated by montane habitat (Table 1) but, consistent with recorded sightings, the majority of occurrence is predicted in grid cells dominated by acid grassland and coniferous woodland. Occurrence is preserved in all land covers where it is observed with the exception of saltwater and suburban dominated habitats.

Most likely due to the limited number of density estimates linear regression suggested no correlation with habitat suitability. Instead, best fit models were based on a constant which was applied to all cells where occurrence was predicted and summed to derive total abundance.

Nevertheless, the predicted abundance range contains the estimate from Harris et al. (1995) suggesting, in agreement with recent trend analysis, no significant change in the total population (since both estimates reference similar density studies this is perhaps unsurprising; the result may indicate that any change in species distribution over the past 20 years is not significant).

Reliability (Expert comment):

All English points outside the Peak District can be justifiably removed as they are almost certainly incorrectly identified brown hares. This will not make much difference to the models, but retaining them will introduce a little skew. The habitat suitability map is contracted in the northwest and on islands when compared to the species ecology and known occurrences. It is surprising that no areas of Wales, particularly the Snowdonia national park, are highlighted in the habitat suitability map. That is not to say that the map is necessarily wrong just that one would have expected a little projection. The land class affinities are as expected. The BBS has indicated a decline 26% decline in abundance between 1995 and 2014 although this was not statistically significant and needs to be treated with caution given the relatively small number of survey squares with sightings. The NGC also recorded a decline between 1998 and 2009 when the relatively low bag index was described as the trough of cycle of abundance. There are thus considerable uncertainties associated with these apparent trends and, hence, the model's predicted abundance range seems reasonable given that it contains the estimate from Harris et al. (1995).

References:

Harris, S. J., P. Morris, S. Wray and D. Yalden (1995). A review of British mammals: population estimates and conservation status of British mammals other than cetaceans, Joint Nature Conservation Committee, Peterborough, UK.

Watson, A., R. Hewson, D. Jenkins and R. Parr (1973). Population densities of mountain hares compared with red grouse on Scottish moors. *Oikos* 24(2): 225-230.

Table 1: Summary of observed data and model predictions by land cover class (LCM2007 target classification). Values shown in brackets denote the spatial coverage based on a 10km resolution raster map (number of grid cells). Years represent the median of records within each land class. Ranges for density and abundance are derived using the respective minimum and maximum raster maps (lower bound is mean of values across minimum raster map with upper across the maximum) which capture the spatial uncertainty generate by projecting irregular polygons describing survey sites onto a raster grid.

LCM2007 class	Observed					Predicted		
	Occurrence		Density					
	Records	Year	Estimates	Year	Range	Habitat suitability	Density	Abundance
1 (Broadleaved woodland)	0 (0)	-	0 (0)	-	-	0.2 (0)	-	0
2 (Coniferous woodland)	304 (100)	2007	4 (4)	1971	0.09 - 6.25	0.8 (129)	0.04 - 18.02	515.7 - 232,426
3 (Arable and Horticultural)	55 (28)	2007	0 (0)	-	-	0.17 (18)	0.04 - 19.72	78.76 - 35,498
4 (Improved grassland)	479 (77)	2003	0 (0)	-	-	0.3 (69)	0.04 - 18.36	281.1 - 126,705
5 (Rough grassland)	27 (15)	2007	0 (0)	-	-	0.46 (14)	0.03 - 14.23	44.19 - 19,918
6 (Neutral grassland)	0 (0)	-	0 (0)	-	-	0.32 (0)	-	0
7 (Calcareous grassland)	0 (0)	-	0 (0)	-	-	0.16 (0)	-	0
8 (Acid grassland)	467 (108)	2007	0 (0)	-	-	0.71 (147)	0.04 - 19.16	624.9 - 281,638
9 (Fen, Marsh, and Swamp)	0 (0)	-	0 (0)	-	-	-	-	0
10 (Heather)	646 (41)	2008	1 (1)	1971	0 - 57.62	0.82 (48)	0.04 - 18.23	194.2 - 87,526
11 (Heather grassland)	659 (76)	2007	2 (2)	1961	0 - 30.67	0.72 (95)	0.03 - 15.35	323.6 - 145,822
12 (Bog)	619 (71)	2007	0 (0)	-	-	0.69 (89)	0.04 - 16.5	325.8 - 146,829
13 (Montane habitat)	1,075 (50)	2010	3 (1)	1961	0 - 13.81	0.96 (53)	0.04 - 19.72	231.9 - 104,522
14 (Inland rock)	2 (1)	1993	0 (0)	-	-	0.83 (1)	0.04 - 19.69	4.37 - 1,969
15 (Saltwater)	1 (1)	1964	0 (0)	-	-	0.19 (0)	-	0
16 (Freshwater)	4 (2)	1994	0 (0)	-	-	0.63 (2)	0.04 - 19.55	8.68 - 3,910
17 (Supra-littoral rock)	0 (0)	-	0 (0)	-	-	0.42 (0)	-	0
18 (Supra-littoral sediment)	0 (0)	-	0 (0)	-	-	0.19 (0)	-	0
19 (Littoral rock)	0 (0)	-	0 (0)	-	-	0.23 (0)	-	0
20 (Littoral sediment)	0 (0)	-	0 (0)	-	-	0.13 (0)	-	0
21 (Saltmarsh)	0 (0)	-	0 (0)	-	-	-	-	0
22 (Urban)	0 (0)	-	0 (0)	-	-	0.13 (0)	-	0
23 (Suburban)	9 (2)	2007	0 (0)	-	-	0.17 (0)	-	0
Total	4,347 (572)	2007	10 (8)	1971	0.04 - 19.72	0.38 (665)	0.04 - 17.85	2,633 - 1,186,763

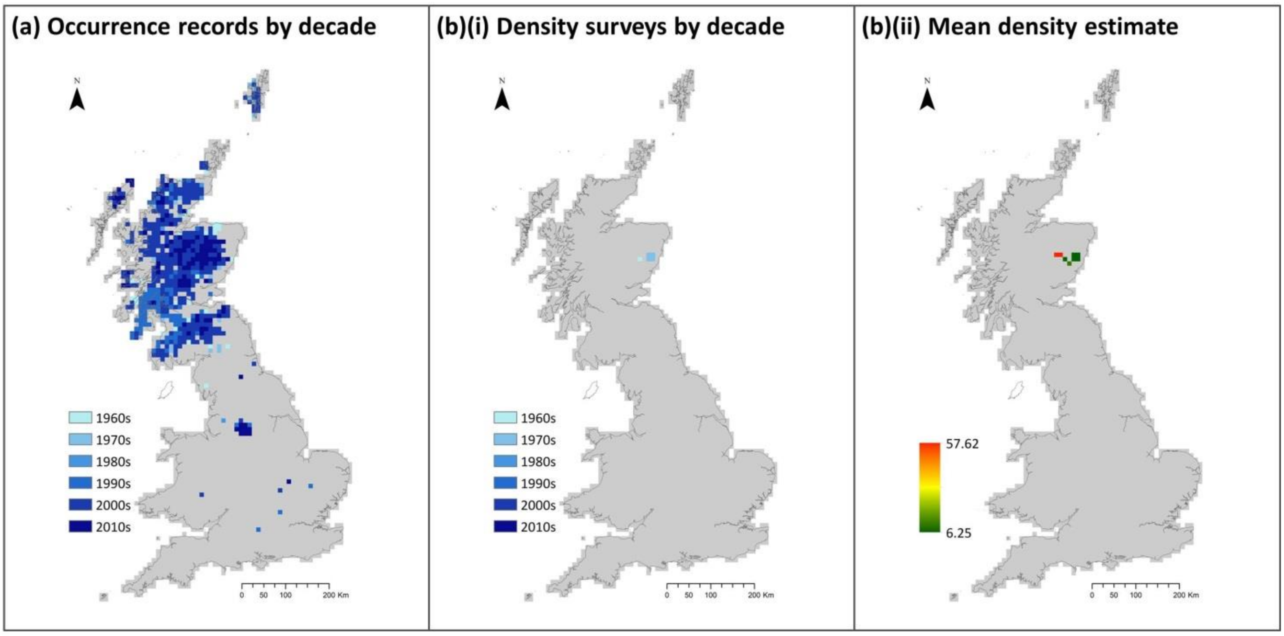

© Crown copyright and database rights 2016 Ordnance Survey 100051110. Data courtesy of the NBN Gateway with thanks to all data contributors. The NBN and its data contributors bear no responsibility for the further analysis or interpretation of this material, data and/or information.

Figure 1: 10km resolution raster maps based on BNG presenting the geographic description of available data. (a) shows the distribution of species occurrence obtained via the NBN Gateway categorised by the decade of last sighting. (b) shows information relating to density surveys identified via a search of published literature where: (i) categorises surveys by the decade of last survey; and (ii) shows the mean density estimate of surveys within grid cells (estimates assumed to be representative of entire cell, considered the upper limit of observed density).

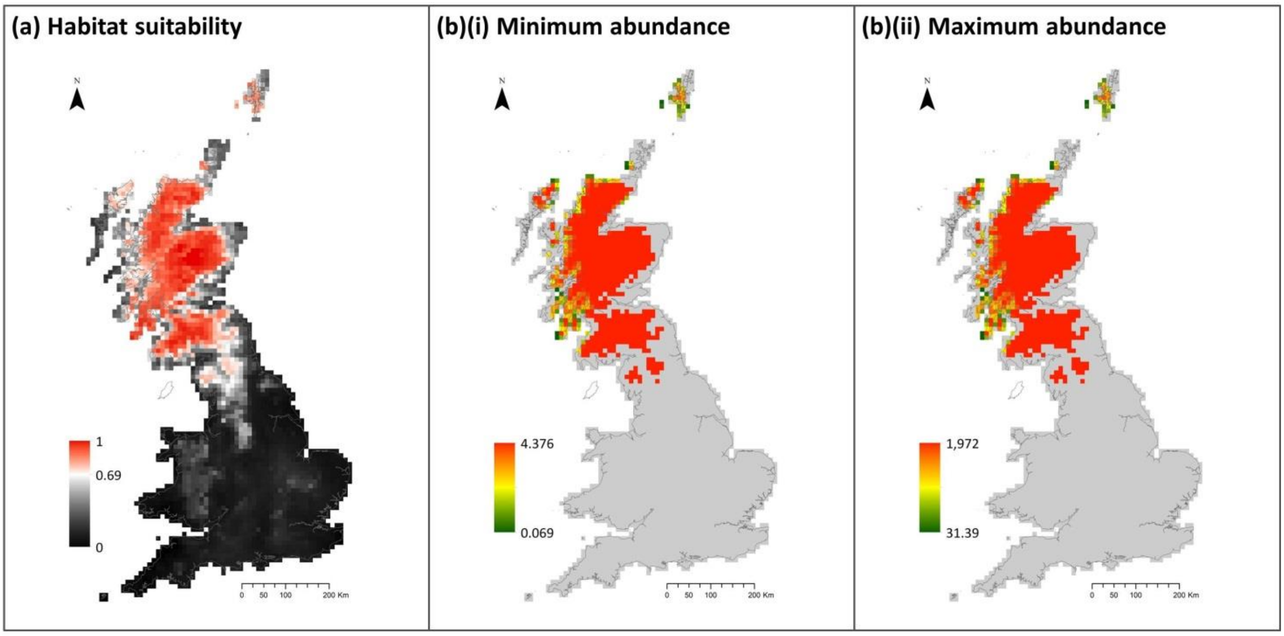

© Crown copyright and database rights 2016 Ordnance Survey 100051110. Data courtesy of the NBN Gateway with thanks to all data contributors. The NBN and its data contributors bear no responsibility for the further analysis or interpretation of this material, data and/or information.

Figure 2: Modelling predictions generated using systematic approach based on available data. (a) shows habitat suitability scores (the likelihood of observing the target species within each grid cell given variation environmental variables) determined by aggregating outputs from the "best" species distribution model (7 models compared) across 100 simulations. Here, the mid value on the scale denotes the threshold score above which occurrence is assumed. (b) shows: (i) the lower bound (Minimum); and (ii) the upper bound (Maximum); of abundance estimates determined by relating observed density (taking into account potential uncertainty) with habitat suitability scores using linear regression.
