# Supplementary material for: A systematic approach to estimate the distribution and total abundance of British mammals
Source: PLoS One. 2017 Jun 28;12(6):e0176339. doi: 10.1371/journal.pone.0176339 (PMC5489149; doi:10.1371/journal.pone.0176339)
Supplement: S7 File — Individual reports for each of the Lagomorpha species presenting analysis of the available data and subsequent model predictions based on a 10km raster grid. Reports also include expert comment assessing the reliability (and plausibility) of results in the context of existing evidence and popular opinion. (ZIP) [file pone.0176339.s007.zip › C Rabbit.pdf]

Rabbit (*Oryctolagus cuniculus*)

Order: *Lagomorpha*

Genus: *Oryctolagus*

Origin: Introduced

Status: Common

1995 abundance estimate: 37,500,000 (3)

Reported population trends: NGC 2009 (↔), JNCC 2005, BBS 2014 (↓)

Data:

The available occurrence records indicate that the rabbit is widespread throughout GB with sightings reported in most 10 km squares (approximately 91%) at least once over the past decade (Figure 1a). However, the map highlights several areas, particularly in Scotland, where the species has not been recorded for some time or not at all.

Despite the coverage and apparent ease with which rabbits can be observed we identified relative few studies reporting estimates of density (Flux & Fullagar 1992; Forman 2005; Kolb 1991a, 1991b). In total, only four surveys were identified spanning a very limited area; less than 1% of the observed species distribution based on the available occurrence data. Geographically, these studies were clustered in Scotland and north east England (historic estimates for 1986 - 1989) with a recent estimate (2004) in Wales (Figure 1b). Estimates ranged between 19.83 and 5,000 per km^2 with the highest densities recorded on Lindisfarne which appears as saltwater dominated habitat (1,463 - 5,000 per km^2 accounting for uncertainty relating to unsurveyed areas within grid cells). Due to the relatively low proportion of area surveyed estimates for many land cover classes were not available (land class marked grey in Table 1).

Model predictions:

The habitat suitability map (Figure 2a) appears to reflect the underlying data well with the set of "best" models predicting presence (and absence) to a mean AUC of 0.84. However, the distribution is slightly contracted towards the outer edges, particularly in the north west of Scotland where occurrence is observed. Overall, across 100 repetitions Random Forest proved to be the most commonly selected modelling approach displaying the highest AUC 44% of the time followed by Generalised Linear Models (22%). MaxEnt could not be assessed as there were insufficient cells without occurrence to perform model analysis. By land cover the mean habitat suitability scores suggest observation is most likely in landscapes dominated by calcareous grassland but, consistent with recorded sightings, the majority of occurrence is predicted in arable and improved grassland (the most common dominant land covers at a 10km scale).

Most likely due to the limited number of density estimates linear regression suggested no correlation with habitat suitability. Instead, a constant mean estimate was applied to all cells where occurrence was predicted and summed to derive total abundance.

Nevertheless, the predicted abundance range contains the estimate from Harris et al. (1995) suggesting no change in the total population (this is perhaps unsurprising as both estimates are based on similar density surveys; the result may instead indicate that any changes in distribution over the past 20 years have not been significant, although the model is unlikely to capture any contractions in range which could result in a population decrease as there is no consideration for recentness of sightings). Whilst the suggestion of stability disagrees with recently reported trends there is scope within the range to argue that populations have declined. In order to better assess potential trends and reduce the size of predicted range more density estimates are required spanning a greater proportion of the observed distribution.

Reliability (Expert comment):

The contraction of the habitat suitability map does not appear to match the known species ecology nor known occurrences. Nevertheless, rabbits are essentially ubiquitous in their distribution.

There are two sources of variation that make estimating rabbit population densities problematic. Firstly, substantial seasonal variation in abundance from pre-breeding lows through to peaks in the latter stages of the breeding season thus density estimates need to be defined with respect to these fluctuations.

Secondly, there have been substantial long-term changes in abundance primarily driven by two diseases. The NGC gamebag data suggest that the population recovered from its collapse, following the arrival myxomatosis in 1953, to approximately 70% of the pre-myxomatosis level by the mid-1990s. There was then a decline around the turn of the century, perhaps driven by the rabbit haemorrhagic disease (RHD), with the trend then levelling off at approximately 50% of pre-myxomatosis numbers by 2010. The BTO Breeding Bird Survey (BBS) trends match those of the NGC, at least until 2009, with a 36% decline in both sets of data from 1995 (although the decline in the NGC data was not statistically significant). However, the BBS has subsequently reported a 57% decline from 1995 to 2014 which suggests that there has been a further decline in rabbit abundance since 2009. Nevertheless, it seems likely that despite these trends the actual current abundance would fall within the range predicted by the model.

References:

Flux, J. E. C. and P. J. Fullagar (1992). World distribution of the rabbit Oryctolagus funiculus on islands. *Mammal Review* 22(3-4): 151-205.

Forman, D. W. (2005). An assessment of the local impact of native predators on an established population of British water voles (Arvicola terrestris). *Journal of Zoology* 266(3): 221-226.

Harris, S. J., P. Morris, S. Wray and D. Yalden (1995). A review of British mammals: population estimates and conservation status of British mammals other than cetaceans, Joint Nature Conservation Committee, Peterborough, UK.

Kolb, H. H. (1991a). Use of burrows and movements by wild rabbits (Oryctolagus cuniculus) on an area of sand dunes. *Journal of Applied Ecology* 28(3): 879-891.

Kolb, H. H. (1991b). Use of burrows and movements of wild rabbits (Oryctolagus cuniculus) in an area of hill grazing and forestry. *Journal of Applied Ecology* 28(3): 892-905.

Table 1: Summary of observed data and model predictions by land cover class (LCM2007 target classification). Values shown in brackets denote the spatial coverage based on a 10km resolution raster map (number of grid cells). Years represent the median of records within each land class. Ranges for density and abundance are derived using the respective minimum and maximum raster maps (lower bound is mean of values across minimum raster map with upper across the maximum) which capture the spatial uncertainty generate by projecting irregular polygons describing survey sites onto a raster grid.

LCM2007 class	Observed					Predicted		
	Occurrence		Density					
	Records	Year	Estimates	Year	Range	Habitat suitability	Density	Abundance
1 (Broadleaved woodland)	546 (11)	2013	0 (0)	-	-	0.89 (11)	10.7 - 1,318	11,743 - 1,449,763
2 (Coniferous woodland)	1,559 (148)	2004	1 (1)	1989	0.38 - 170	0.8 (99)	10.5 - 1,294	103,793 - 12,814,479
3 (Arable and Horticultural)	58,606 (948)	2013	2 (2)	1988	26.2 - 3,115	0.89 (958)	9.7 - 1,203	933,289 - 115,226,023
4 (Improved grassland)	30,075 (741)	2011	1 (1)	2004	0.25 - 19.83	0.87 (719)	9.5 - 1,174	683,658 - 84,405,916
5 (Rough grassland)	453 (55)	1994	0 (0)	-	-	0.71 (20)	7.3 - 904.3	14,649 - 1,808,589
6 (Neutral grassland)	22 (1)	2002	0 (0)	-	-	0.76 (0)	-	-
7 (Calcareous grassland)	86 (2)	2012	0 (0)	-	-	0.97 (2)	10.7 - 1,318	2,135 - 263,593
8 (Acid grassland)	1,926 (162)	2004	1 (1)	1989	0.29 - 170	0.74 (104)	10.6 - 1,304	109,835 - 13,560,535
9 (Fen, Marsh, and Swamp)	0 (0)	-	0 (0)	-	-	-	-	-
10 (Heather)	653 (55)	2006	0 (0)	-	-	0.83 (45)	9.9 - 1221	44,491 - 5,492,940
11 (Heather grassland)	1,458 (121)	2004	0 (0)	-	-	0.74 (58)	8.7 - 1,070	50,287 - 6,208,504
12 (Bog)	1,646 (126)	2003	0 (0)	-	-	0.74 (71)	9.5 - 1,174	67,519 - 8,336,042
13 (Montane habitat)	476 (37)	2003	0 (0)	-	-	0.65 (5)	10.7 - 1,318	5,338 - 658,983
14 (Inland rock)	12 (1)	2013	0 (0)	-	-	0.71 (0)	-	-
15 (Saltwater)	132 (10)	2012	1 (1)	1986	1463 - 5000	0.78 (8)	4.4 - 547.7	3,549 - 438,158
16 (Freshwater)	20 (3)	2004	0 (0)	-	-	0.8 (2)	10.6 - 1,307	2,117 - 261,313
17 (Supra-littoral rock)	0 (0)	-	0 (0)	-	-	0 (0)	-	-
18 (Supra-littoral sediment)	206 (5)	2011	0 (0)	-	-	0.74 (4)	1.73 - 214	693.2 - 85,585
19 (Littoral rock)	65 (3)	2012	0 (0)	-	-	0.41 (0)	-	-
20 (Littoral sediment)	627 (28)	2010	0 (0)	-	-	0.73 (4)	6 - 744.4	2,412 - 297,761
21 (Saltmarsh)	0 (0)	-	0 (0)	-	-	-	-	-
22 (Urban)	239 (8)	2010	0 (0)	-	-	0.78 (5)	8.8 - 1,090	4,414 - 544,975
23 (Suburban)	3,225 (74)	2013	0 (0)	-	-	0.76 (29)	10.2 - 1260	29,607 - 3,655,383
Total	102,032 (2,539)	2011	6 (6)	1989	253 - 1,932	0.83 (2,144)	9.7 - 1,192	2,069,527 - 255,508,540

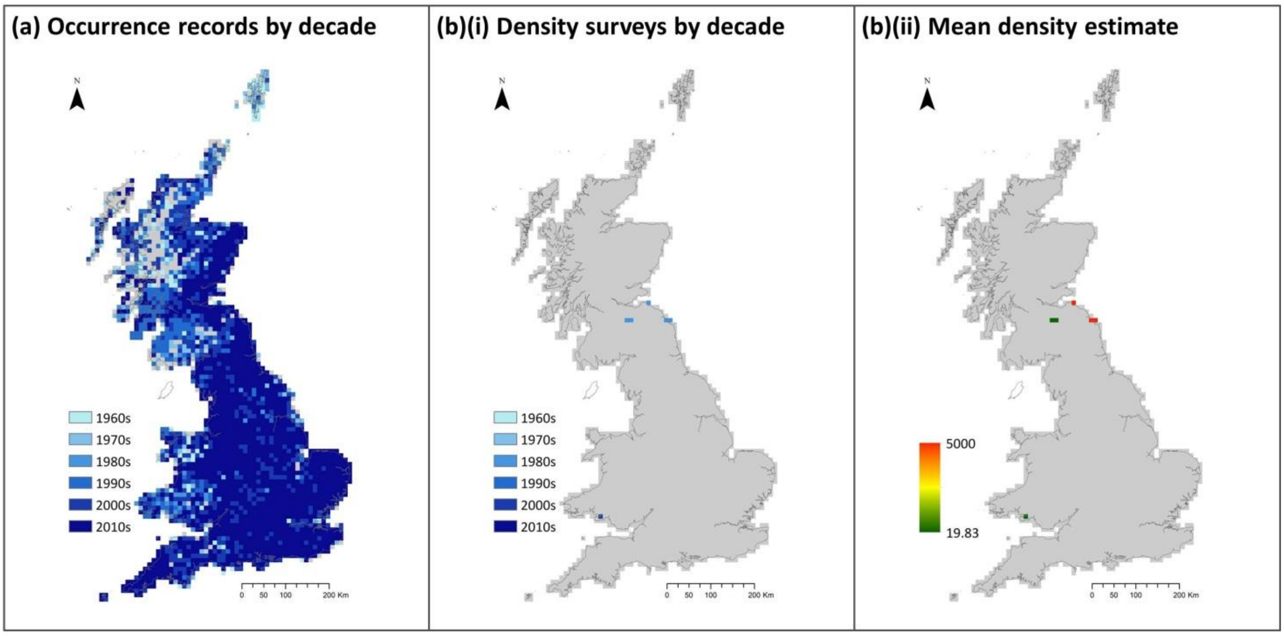

© Crown copyright and database rights 2016 Ordnance Survey 100051110. Data courtesy of the NBN Gateway with thanks to all data contributors. The NBN and its data contributors bear no responsibility for the further analysis or interpretation of this material, data and/or information.

Figure 1: 10km resolution raster maps based on BNG presenting the geographic description of available data. (a) shows the distribution of species occurrence obtained via the NBN Gateway categorised by the decade of last sighting. (b) shows information relating to density surveys identified via a search of published literature where: (i) categorises surveys by the decade of last survey; and (ii) shows the mean density estimate of surveys within grid cells (estimates assumed to be representative of entire cell, considered the upper limit of observed density).

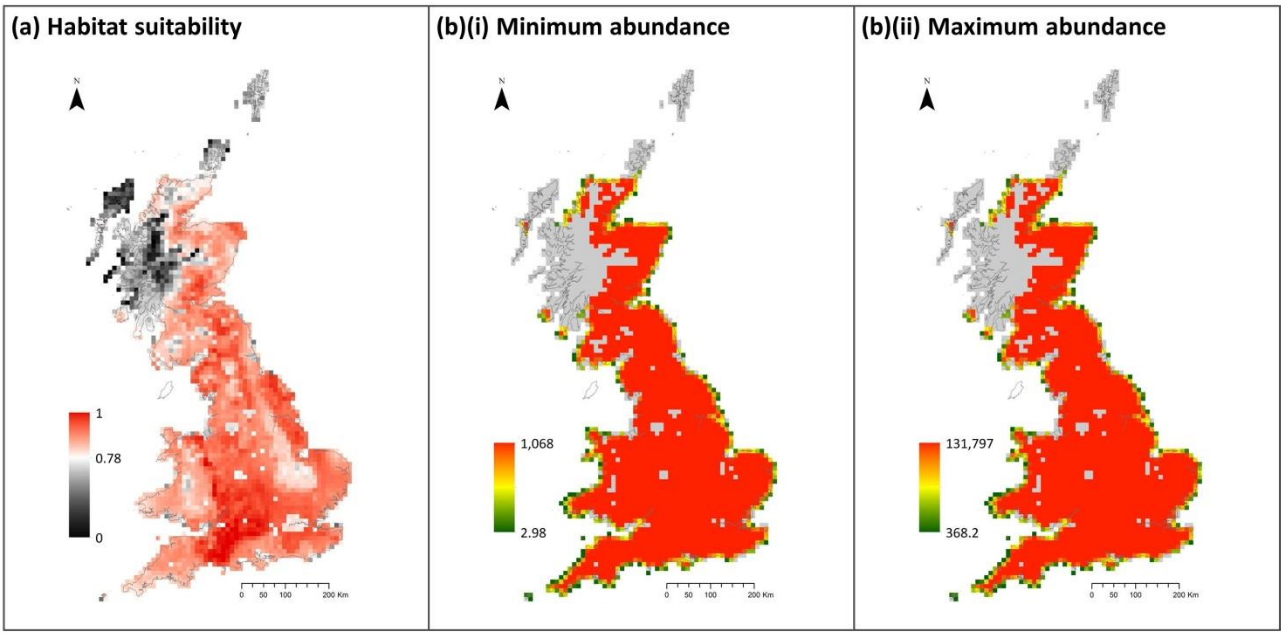

© Crown copyright and database rights 2016 Ordnance Survey 100051110. Data courtesy of the NBN Gateway with thanks to all data contributors. The NBN and its data contributors bear no responsibility for the further analysis or interpretation of this material, data and/or information.

Figure 2: Modelling predictions generated using systematic approach based on available data. (a) shows habitat suitability scores (the likelihood of observing the target species within each grid cell given variation environmental variables) determined by aggregating outputs from the "best" species distribution model (7 models compared) across 100 simulations. Here, the mid value on the scale denotes the threshold score above which occurrence is assumed. (b) shows: (i) the lower bound (Minimum); and (ii) the upper bound (Maximum); of abundance estimates determined by relating observed density (taking into account potential uncertainty) with habitat suitability scores using linear regression.
