# Supplementary material for: A systematic approach to estimate the distribution and total abundance of British mammals
Source: PLoS One. 2017 Jun 28;12(6):e0176339. doi: 10.1371/journal.pone.0176339 (PMC5489149; doi:10.1371/journal.pone.0176339)
Supplement: S8 File — Individual reports for each of the Marsupialia species presenting analysis of the available data and subsequent model predictions based on a 10km raster grid. Reports also include expert comment assessing the reliability (and plausibility) of results in the context of existing evidence and popular opinion. (ZIP) [file pone.0176339.s008.zip › A Red-necked wallaby.pdf]

Red-necked wallaby (*Macropus rufogriseus*)

Order: *Marsupialia*

Genus: *Macropus*

Origin: Introduced

Status: Rare

1995 abundance estimate: 29 (1)

Reported population trends: None

Data:

The available occurrence records indicate that the red-necked wallaby is rare with localised patches scattered across south eastern and central part of England (Figure 1a). These sightings were reported in various habitats (predominantly arable and improved grassland) with the majority (of cells) where occurrence was observed containing at least one record since 1995. However, the map highlights several cells where the species has not been reported for some time.

From the literature review we identified a single survey (Weir et al. 1995) conducted in western Scotland in 1992 (Figure 1b). At a 10km scale the study comprised a single grid cell dominated by freshwater habitat estimating density to be 57.78 per km^2. Consequently, estimates in other land covers where occurrence had been observed were not available (marked grey in Table 1). Due to the relative size of area surveyed within the cell the potential range of density, accounting for uncertainty, was very large (0.28 - 57.78 per km^2).

Model predictions:

The habitat suitability map (Figure 2a) appears to reflect the underlying data reasonably well with the set of "best" models predicting presence (and absence) to a mean AUC of 0.78. However, the resulting distribution is substantially larger than the area described by the observations (approximately 5 times) which is unlikely given the rarity of the species and recentness of records. Overall, across 100 repetitions MaxEnt proved to be the most commonly selected modelling approach displaying the highest AUC 27% of the time followed by BioClim (24%). By land cover the mean habitat suitability scores suggest observation is most likely in landscapes dominated by freshwater but, consistent with recorded sightings, the majority of occurrence is predicted in arable and improved grassland (the most common dominant land covers at a 10km scale).

Due to the limited number of density estimates it was not possible to assess any relationship with habitat suitability. Instead, a constant mean estimate was applied to all cells where occurrence was predicted and summed to derive total abundance.

Perhaps unsurprisingly given the inflated distribution and high density estimates (although it should be noted these are the same as were used by Harris et al.), the predicted abundance range does not contain the estimate from Harris et al. (1995) suggesting a significant increase in the total population. This overestimation highlights the difficulty of modelling very rare species at such broad scales. Predictions may be more plausible by applying models based on finer scale raster data.

Reliability (Expert comment):

The reliability of these estimates is considered to be low. This is a scarce non-native species with only limited data on which to base density estimates and habitat associations. While a breeding population has been established over many years, including a reported association with heather moorland and grassland, recent anecdotal evidence suggests this species may no longer be breeding in the wild. Alongside this, sporadic releases and sightings of escapes from captivity continue to provide distributional records that may reflect the location of captive animals more than the species' habitat requirements. Consequently, it is likely that the distributional extent as a breeding species is over-estimated due to sporadic releases, while the density estimates and habitat associations, particularly with freshwater, should be treated with caution.

References:

Harris, S. J., P. Morris, S. Wray and D. Yalden (1995). A review of British mammals: population estimates and conservation status of British mammals other than cetaceans, Joint Nature Conservation Committee, Peterborough, UK.

Weir, A., J. McLeod and C. E. Adams (1995). The winter diet and parasitic fauna of a population of red-necked wallabies *Macropus rufogriseus* recently introduced to Scotland. *Mammal Review* 25(3): 111-116.

Table 1: Summary of observed data and model predictions by land cover class (LCM2007 target classification). Values shown in brackets denote the spatial coverage based on a 10km resolution raster map (number of grid cells). Years represent the median of records within each land class. Ranges for density and abundance are derived using the respective minimum and maximum raster maps (lower bound is mean of values across minimum raster map with upper across the maximum) which capture the spatial uncertainty generate by projecting irregular polygons describing survey sites onto a raster grid.

LCM2007 class	Observed					Predicted		
	Occurrence		Density					
	Records	Year	Estimates	Year	Range	Habitat suitability	Density	Abundance
1 (Broadleaved woodland)	0 (0)	-	0 (0)	-	-	0.27 (0)	-	-
2 (Coniferous woodland)	1 (1)	2005	0 (0)	-	-	0.17 (1)	0.28 - 57.78	28.49 - 5,778
3 (Arable and Horticultural)	15 (10)	1998	0 (0)	-	-	0.32 (59)	0.28 - 57.78	1,681 - 340,903
4 (Improved grassland)	99 (14)	1970	0 (0)	-	-	0.28 (65)	0.28 - 57.78	1,852 - 375,571
5 (Rough grassland)	0 (0)	-	0 (0)	-	-	0.11 (0)	-	-
6 (Neutral grassland)	0 (0)	-	0 (0)	-	-	0.04 (0)	-	-
7 (Calcareous grassland)	0 (0)	-	0 (0)	-	-	0.38 (0)	-	-
8 (Acid grassland)	0 (0)	-	0 (0)	-	-	0.16 (0)	-	-
9 (Fen, Marsh, and Swamp)	0 (0)	-	0 (0)	-	-	-	-	-
10 (Heather)	0 (0)	-	0 (0)	-	-	0.14 (0)	-	-
11 (Heather grassland)	0 (0)	-	0 (0)	-	-	0.11 (0)	-	-
12 (Bog)	0 (0)	-	0 (0)	-	-	0.11 (0)	-	-
13 (Montane habitat)	0 (0)	-	0 (0)	-	-	0.11 (0)	-	-
14 (Inland rock)	0 (0)	-	0 (0)	-	-	0.14 (0)	-	-
15 (Saltwater)	0 (0)	-	0 (0)	-	-	0.17 (0)	-	-
16 (Freshwater)	0 (0)	-	1 (1)	1992	0.28 - 57.78	0.45 (1)	0.28 - 57.78	28.49 - 5,778
17 (Supra-littoral rock)	0 (0)	-	0 (0)	-	-	0.1 (0)	-	-
18 (Supra-littoral sediment)	0 (0)	-	0 (0)	-	-	0.11 (0)	-	-
19 (Littoral rock)	0 (0)	-	0 (0)	-	-	0.13 (0)	-	-
20 (Littoral sediment)	0 (0)	-	0 (0)	-	-	0.16 (0)	-	-
21 (Saltmarsh)	0 (0)	-	0 (0)	-	-	-	-	-
22 (Urban)	0 (0)	-	0 (0)	-	-	0.16 (0)	-	-
23 (Suburban)	0 (0)	-	0 (0)	-	-	0.19 (0)	-	-
Total	115 (25)	1993	1 (1)	1992	0.28 - 57.78	0.24 (126)	0.28 - 57.78	3,590 - 728,030

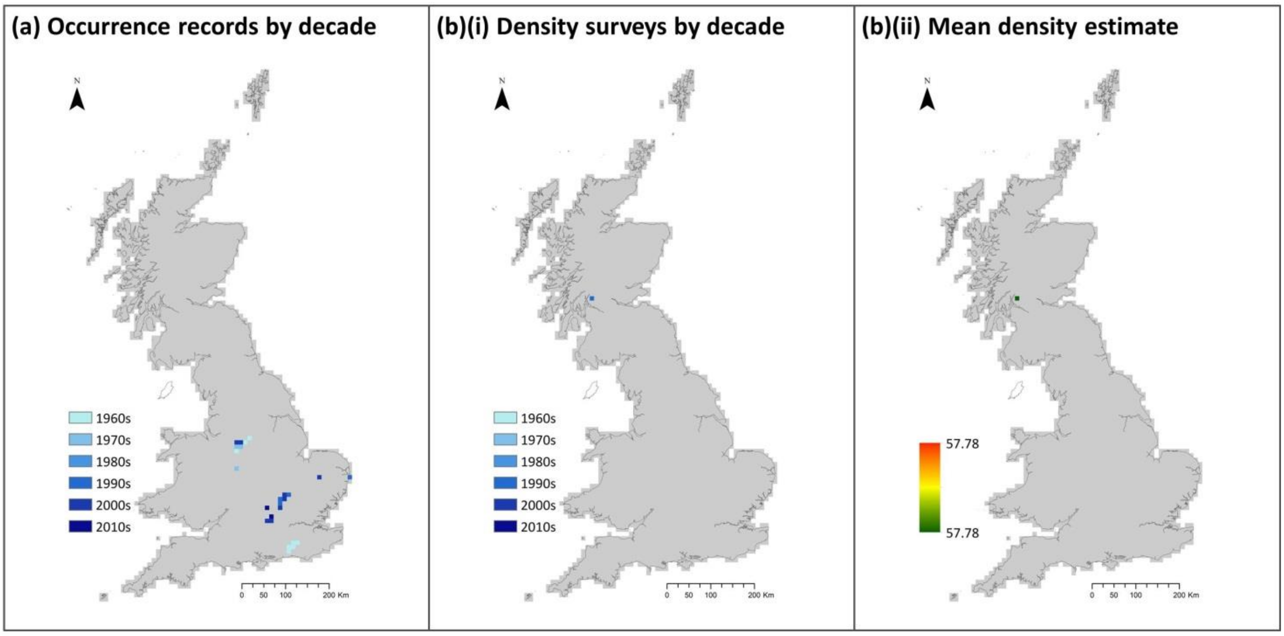

© Crown copyright and database rights 2016 Ordnance Survey 100051110. Data courtesy of the NBN Gateway with thanks to all data contributors. The NBN and its data contributors bear no responsibility for the further analysis or interpretation of this material, data and/or information.

Figure 1: 10km resolution raster maps based on BNG presenting the geographic description of available data. (a) shows the distribution of species occurrence obtained via the NBN Gateway categorised by the decade of last sighting. (b) shows information relating to density surveys identified via a search of published literature where: (i) categorises surveys by the decade of last survey; and (ii) shows the mean density estimate of surveys within grid cells (estimates assumed to be representative of entire cell, considered the upper limit of observed density).

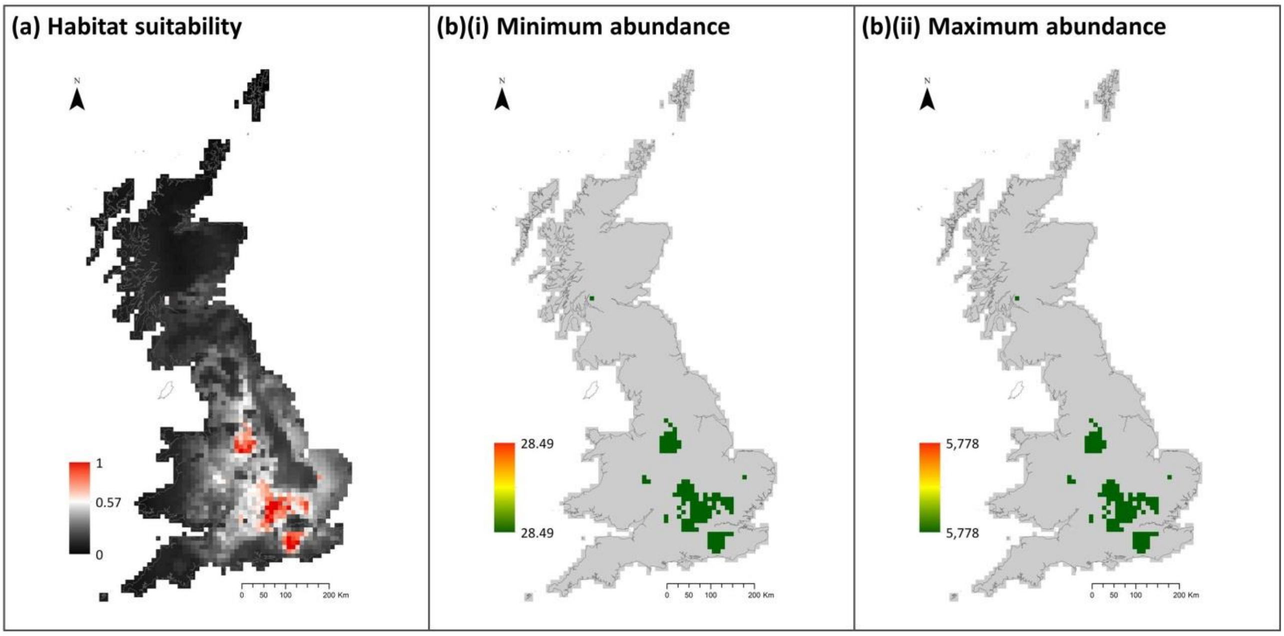

© Crown copyright and database rights 2016 Ordnance Survey 100051110. Data courtesy of the NBN Gateway with thanks to all data contributors. The NBN and its data contributors bear no responsibility for the further analysis or interpretation of this material, data and/or information.

Figure 2: Modelling predictions generated using systematic approach based on available data. (a) shows habitat suitability scores (the likelihood of observing the target species within each grid cell given variation environmental variables) determined by aggregating outputs from the “best” species distribution model (7 models compared) across 100 simulations. Here, the mid value on the scale denotes the threshold score above which occurrence is assumed. (b) shows: (i) the lower bound (Minimum); and (ii) the upper bound (Maximum); of abundance estimates determined by relating observed density (taking into account potential uncertainty) with habitat suitability scores using linear regression.
