# Supplementary material for: A systematic approach to estimate the distribution and total abundance of British mammals
Source: PLoS One. 2017 Jun 28;12(6):e0176339. doi: 10.1371/journal.pone.0176339 (PMC5489149; doi:10.1371/journal.pone.0176339)
Supplement: S9 File — Individual reports for each of the Rodentia species presenting analysis of the available data and subsequent model predictions based on a 10km raster grid. Reports also include expert comment assessing the reliability (and plausibility) of results in the context of existing evidence and popular opinion. (ZIP) [file pone.0176339.s009.zip › A Bank vole.pdf]

Bank vole (*Myodes glareolus*)

Order: *Rodentia*

Genus: *Myodes*

Origin: Native

Status: Common

1995 abundance estimate: 23,000,000 (3)

Reported population trends: None

Data:

The available occurrence records indicate that the bank vole is widespread throughout England with sightings becoming more sporadic in Wales and Scotland (Figure 1a). The map highlights several areas, particularly in the south east of England, where the species has not been recorded for some time.

Density estimates, primarily recorded over the past two decades, were obtained from published literature spanning approximately 2% of the observed species distribution based on the available occurrence data (Baker et al. 2005; Bush et al. 2012; Flowerdew & Ellwood 2001; Flowerdew et al. 2004; Forman 2005, Gelling et al. 2007; Kotzageorgis & Mason 1997; Mallorie & Flowerdew 1994; Shore et al. 2005; Tattersall et al. 2002; Telfer et al . 2007). Geographically, these studies were well spread throughout the observed occurrence distribution (Figure 1b); although most were conducted in habitats dominated by arable and improved grassland land cover with estimates unavailable for some other less common land covers (land class marked grey in Table 1). Estimates ranged between 0 and 9,753 per km^2 with the highest densities recorded in suburban habitat (6.85 - 2,011 per km^2 accounting for uncertainty relating to unsurveyed areas within grid cells). In common with reporting of density for most small mammals survey sites comprised only a fraction of 10km cells. The uncertainty, and therefore the range, associated with density estimates was consequently very large.

Model predictions:

The habitat suitability map (Figure 2a) appears to reflect the underlying data well with the set of "best" models predicting presence (and absence) to a mean AUC of 0.72. Overall, across 100 repetitions MaxEnt proved to be the most commonly selected modelling approach displaying the highest AUC 36% of the time followed closely by Random Forest (30%). By land cover the mean habitat suitability scores suggest observation is most likely in landscapes dominated by calcareous grassland, broadleaved woodland, suburban and arable land (Table 1); the latter two perhaps indicates use of man-made drainage channels. However, consistent with recorded sightings, the majority of occurrence is predicted in grid cells dominated by arable and improved grassland. The analysis shows that occurrence is preserved in all land covers where it is observed with the exception of heather and montane dominated habitats.

Linear regression suggested that there was no correlation between the estimates of minimum density and habitat suitability; consequently it was applied as a constant across all cells where occurrence was predicted. This result was most likely due to differences in survey areas causing relative scaling issues (i.e. studies with smaller areas will be subject to a greater reduction when accounting for uncertainty; due to the variety of different study sizes estimates will not be scaled equally disrupting any associations with habitat). Maximum density was found to be correlated with the best fit model relating the square of habitat suitability accounting for spherical spatial autocorrelation.

The predicted abundance range contains the estimate from Harris et al. (1995) suggesting no change in the total population (since the median year of observed density estimates is 1995 then this result is likely to indicate no significant change in spatial distribution over the past 20 years). However, the range is very large again due to the uncertainty caused by small survey sites relative to the 10km scale at which modelling is performed. In order to provide more accurate predictions future model analysis could be based on a finer scale raster grid which would better represent the variations in habitat for smaller mammals. Unfortunately, at present this is too unreliable due to access restrictions imposed on occurrence data.

Reliability (Expert comment):

Bank voles were most frequently recorded in arable and horticultural habitats. Relatively high densities were recorded for coniferous woodland, arable and horticulture, improved grassland, rough grassland and suburban habitats, i.e. in all but one land class (freshwater) for which data were available. This reflects the ubiquitous nature of bank voles in Britain, although their density in grassland (and other land classes including bog and freshwater) is likely to depend on availability of preferred habitats within this, such as woodland edges and hedgerows. The range for predicted total abundance is very large, and it is possible that the upper limit is inflated by extrapolating densities recorded in preferred (linear) habitats (e.g. hedgerows) within grid cells to the dominant land class for those grid cells.

References:

Baker, P. J., A. J. Bentley, R. J. Ansell and S. Harris (2005). Impact of predation by domestic cats *Felis catus* in an urban area. *Mammal Review* 35(3-4): 302-312.

Bush, E. R., C. D. Buesching, E. M. Slade and D. W. Macdonald (2012). Woodland recovery after suppression of deer: cascade effects for small mammals, wood mice (*Apodemus sylvaticus*) and bank voles (*Myodes glareolus*). *PLOS ONE* 7(2): e31404.

Flowerdew, J. R. and S. A. Ellwood (2001). Impacts of woodland deer on small mammal ecology. *Forestry* 74(3): 277-287.

Flowerdew, J. R., R. F. Shore, S. M. C. Poulton and T. H. Sparks (2004). Live trapping to monitor small mammals in Britain. *Mammal Review* 34(1-2): 31-50.

Forman, D. W. (2005). An assessment of the local impact of native predators on an established population of British water voles (*Arvicola terrestris*). *Journal of Zoology* 266(3): 221-226.

Gelling, M., D. W. Macdonald and F. Mathews (2007). Are hedgerows the route to increased farmland small mammal density? Use of hedgerows in British pastoral habitats. *Landscape Ecology* 22(7): 1019-1032.

Harris, S. J., P. Morris, S. Wray and D. Yalden (1995). A review of British mammals: population estimates and conservation status of British mammals other than cetaceans, Joint Nature Conservation Committee, Peterborough, UK.

Kotzageorgis, G. C. and C. F. Mason (1997). Small mammal populations in relation to hedgerow structure in an arable landscape. *Journal of Zoology* 242(3): 425-434.

Mallorie, H. C. and J. R. Flowerdew (1994). Woodland small mammal population ecology in Britain: a preliminary review of the Mammal Society survey of Wood Mice *Apodemus sylvaticus* and Bank Voles *Clethrionomys glareolus*, 1982–87. *Mammal Review* 24(1): 1-15.

Shore, R. F., W. R. Meek, T. H. Sparks, R. F. Pywell and M. Nowakowski (2005). Will environmental stewardship enhance small mammal abundance on intensively managed farmland? *Mammal Review* 35(3-4): 277-284.

Tattersall, F. H., D. W. Macdonald, B. J. Hart, P. Johnson, W. Manley and R. Feber (2002). Is habitat linearity important for small mammal communities on farmland? *Journal of Applied Ecology* 39(4): 643-652.

Telfer, S., H. E. Clough, R. J. Birtles, M. Bennett, D. Carslake, S. Helyar and M. Begon (2007). Ecological differences and coexistence in a guild of micro-parasites: *Bartonella* in wild rodents. *Ecology* 88(7): 1841-1849.

Table 1: Summary of observed data and model predictions by land cover class (LCM2007 target classification). Values shown in brackets denote the spatial coverage based on a 10km resolution raster map (number of grid cells). Years represent the median of records within each land class. Ranges for density and abundance are derived using the respective minimum and maximum raster maps (lower bound is mean of values across minimum raster map with upper across the maximum) which capture the spatial uncertainty generate by projecting irregular polygons describing survey sites onto a raster grid.

LCM2007 class	Observed					Predicted		
	Occurrence		Density					
	Records	Year	Estimates	Year	Range	Habitat suitability	Density	Abundance
1 (Broadleaved woodland)	91 (8)	1994	0 (0)	-	-	0.89 (11)	1.3 - 1,449	1,443- 1,594,189
2 (Coniferous woodland)	159 (60)	1994	1 (1)	1987	0.3 - 1,605	0.66 (28)	1.3 - 1,180	3,655 - 3,303,408
3 (Arable and Horticultural)	5,529 (699)	2003	22 (15)	1994	0.6 - 1,069	0.89 (880)	1.2 - 1,338	106,598 - 117,715,939
4 (Improved grassland)	2,738 (473)	1997	20 (16)	2003	1.3 - 1,367	0.81 (538)	1.2 - 1,277	65,030 - 68,688,025
5 (Rough grassland)	40 (10)	1999	1 (1)	1994	0.2 - 1,235	0.31 (8)	0.8 - 676	643 - 540,856
6 (Neutral grassland)	0 (0)	-	0 (0)	-	-	0.03 (0)	-	0
7 (Calcareous grassland)	7 (2)	1976	0 (0)	-	-	0.92 (2)	1.3 - 1,548	262.3 - 309,642
8 (Acid grassland)	117 (45)	1993	0 (0)	-	-	0.51 (4)	1.3 - 954.7	524.7 - 381,874
9 (Fen, Marsh, and Swamp)	0 (0)	-	0 (0)	-	-	-	-	0
10 (Heather)	26 (15)	1997	0 (0)	-	-	0.56 (3)	0.9 - 627	262.7 - 188,108
11 (Heather grassland)	92 (29)	1999	0 (0)	-	-	0.42 (0)	-	0
12 (Bog)	37 (17)	1985	0 (0)	-	-	0.29 (7)	1.3 - 1,214	918.2 - 849,516
13 (Montane habitat)	22 (7)	1979	0 (0)	-	-	0.35 (0)	-	0
14 (Inland rock)	0 (0)	-	0 (0)	-	-	0.2 (0)	-	0
15 (Saltwater)	12 (4)	1986	0 (0)	-	-	0.71 (1)	0.6 - 529	58.55 - 52,897
16 (Freshwater)	21 (2)	2010	1 (1)	1986	0.06 - 370	0.57 (1)	1.3 - 1,186	128.9 - 118,586
17 (Supra-littoral rock)	0 (0)	-	0 (0)	-	-	0.1 (0)	-	0
18 (Supra-littoral sediment)	7 (2)	1988	0 (0)	-	-	0.52 (1)	0.4 - 321	39.17 - 32,144
19 (Littoral rock)	0 (0)	-	0 (0)	-	-	0.33 (1)	0 - 4	0.41 - 401.6
20 (Littoral sediment)	153 (20)	1995	0 (0)	-	-	0.79 (20)	0.8 - 797	1,665 - 1,594,715
21 (Saltmarsh)	0 (0)	-	0 (0)	-	-	-	-	0
22 (Urban)	24 (4)	1972	0 (0)	-	-	0.78 (3)	1 - 808	305 - 242,313
23 (Suburban)	497 (62)	1987	2 (2)	2003	6.9 - 2,011	0.89 (74)	1.1 - 1,191	8,088 - 8,814,344
Total	9,572 (1,459)	2000	47 (36)	1995	1.2 - 1,252	0.73 (1,582)	1.2 - 1,292	189,622 - 204,426,956

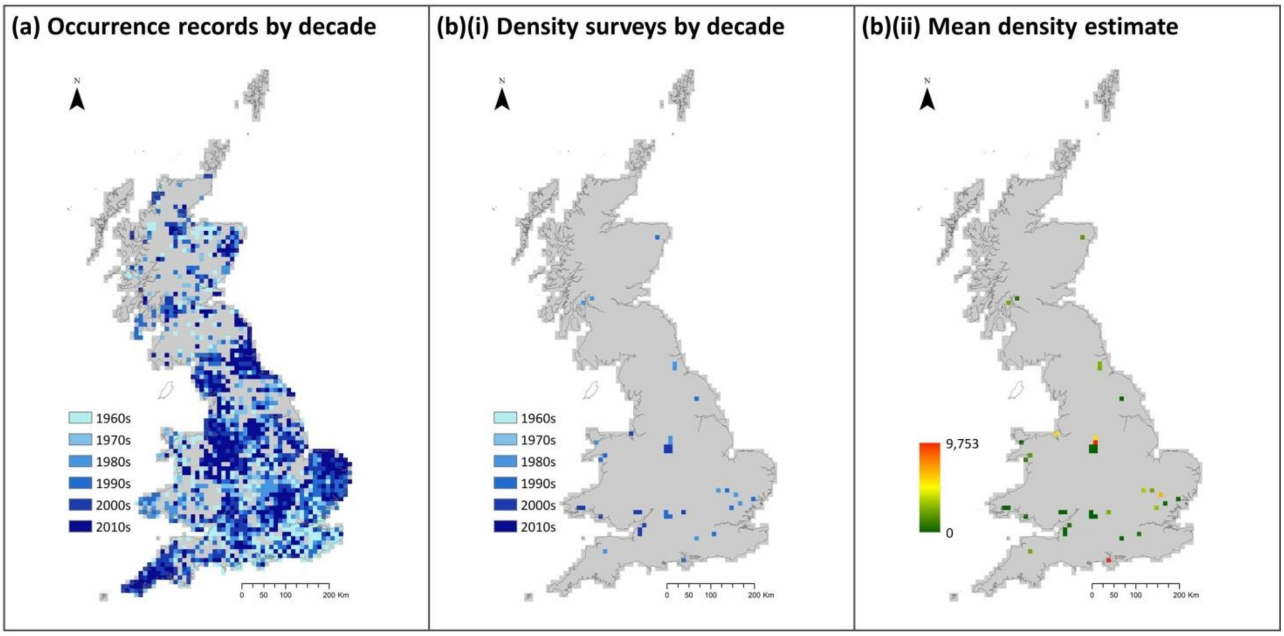

© Crown copyright and database rights 2016 Ordnance Survey 100051110. Data courtesy of the NBN Gateway with thanks to all data contributors. The NBN and its data contributors bear no responsibility for the further analysis or interpretation of this material, data and/or information.

Figure 1: 10km resolution raster maps based on BNG presenting the geographic description of available data. (a) shows the distribution of species occurrence obtained via the NBN Gateway categorised by the decade of last sighting. (b) shows information relating to density surveys identified via a search of published literature where: (i) categorises surveys by the decade of last survey; and (ii) shows the mean density estimate of surveys within grid cells (estimates assumed to be representative of entire cell, considered the upper limit of observed density).

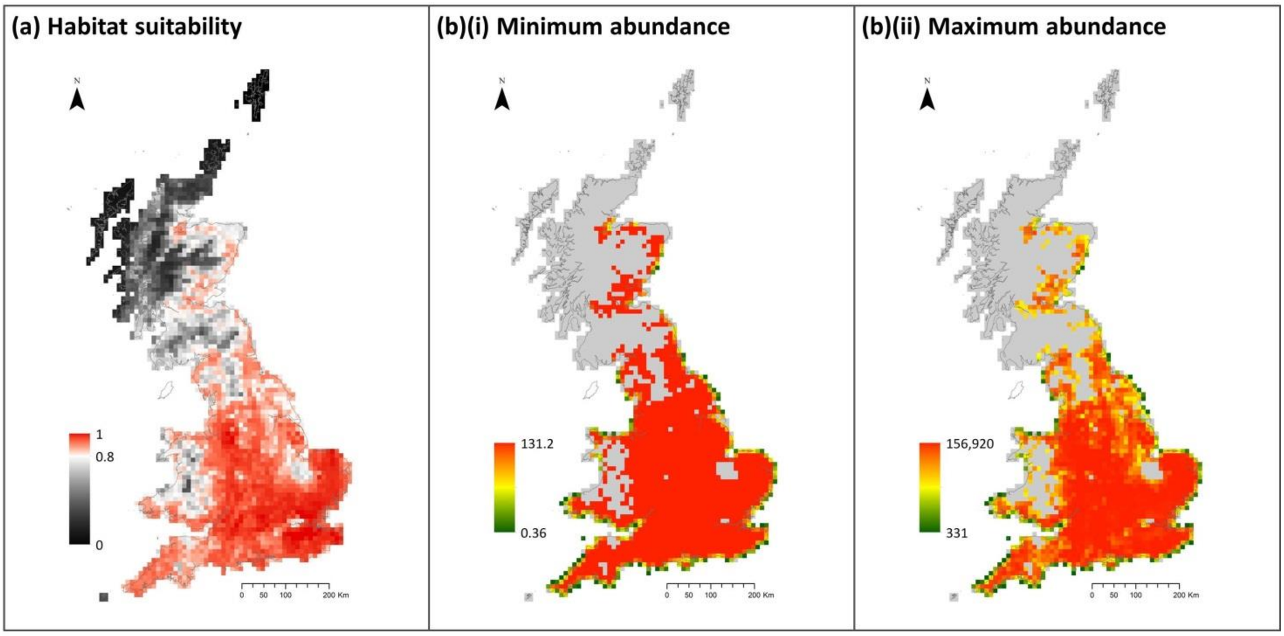

© Crown copyright and database rights 2016 Ordnance Survey 100051110. Data courtesy of the NBN Gateway with thanks to all data contributors. The NBN and its data contributors bear no responsibility for the further analysis or interpretation of this material, data and/or information.

Figure 2: Modelling predictions generated using systematic approach based on available data. (a) shows habitat suitability scores (the likelihood of observing the target species within each grid cell given variation environmental variables) determined by aggregating outputs from the “best” species distribution model (7 models compared) across 100 simulations. Here, the mid value on the scale denotes the threshold score above which occurrence is assumed. (b) shows: (i) the lower bound (Minimum); and (ii) the upper bound (Maximum); of abundance estimates determined by relating observed density (taking into account potential uncertainty) with habitat suitability scores using linear regression.
