# Supplementary material for: A systematic approach to estimate the distribution and total abundance of British mammals
Source: PLoS One. 2017 Jun 28;12(6):e0176339. doi: 10.1371/journal.pone.0176339 (PMC5489149; doi:10.1371/journal.pone.0176339)
Supplement: S9 File — Individual reports for each of the Rodentia species presenting analysis of the available data and subsequent model predictions based on a 10km raster grid. Reports also include expert comment assessing the reliability (and plausibility) of results in the context of existing evidence and popular opinion. (ZIP) [file pone.0176339.s009.zip › B Common dormouse.pdf]

Common dormouse (*Muscardinus avellanarius*)

Order: *Rodentia*

Genus: *Muscardinus*

Origin: Native

Status: Locally common

1995 abundance estimate: 500,000 (3)

Reported population trends: JNCC 2005 (↓)

Data:

The available occurrence records indicate that the common dormouse is most often observed in the south of England spreading into Wales with a few localised populations scattered throughout northern regions (Figure 1a). However, the map highlights that the species is almost completely absent in Scotland with only a single cell recording any sightings. Overall, sightings were reported in various habitats (predominantly arable and improved grassland) with the majority of cells where occurrence was observed containing at least one record since 1995.

From the literature review we identified a single density estimate of 235 per km^2 (Trout et al. 2012) reported on arable dominated habitat in 2007 (Figure 1b). Consequently, no estimates were available for other dominant land covers (marked grey in Table 1). Taking to account the uncertainty relating to study area when projected onto a 10km raster grid the range of densities was very large (0.37 - 235 per km^2).

Model predictions:

The habitat suitability map (Figure 2a) appears to reflect the underlying data well with the set of "best" models predicting presence (and absence) to a mean AUC of 0.75. Overall, across 100 repetitions MaxEnt proved to be the most commonly selected modelling approach displaying the highest AUC 42% of the time followed by Support Vector Machines (15%). By land cover the mean habitat suitability scores suggest observation is most likely in landscapes dominated by broadleaved woodland (Table 1) but, consistent with recorded sightings, the majority of occurrence is predicted in grid cells dominated by arable and improved grassland.

Due to the limited number of density estimates it was not possible to assess any relationship with habitat suitability. Instead, a constant mean estimate was applied across all cells where occurrence was predicted.

The predicted abundance range contains the estimate from Harris et al. (1995) suggesting no change in the total population. Whilst this result disagrees with recently reported trends there is scope within the range to argue that populations have declined (if the decline is due to a contraction of the species distribution rather than density then this is unlikely to be detected by the model as there is no consideration for the age of sightings). The range itself is very large due to the uncertainty caused by small survey sites relative to the 10km scale at which modelling is performed. In order to provide more accurate predictions future model analysis could be based on a finer scale raster grid which would better represent the variations in habitat for smaller mammals. Unfortunately, at present this is too unreliable due to access restrictions imposed on occurrence data.

Reliability (Expert comment):

Surveys of potential common dormouse habitats are commonly undertaken in Britain by developers to ensure compliance with European legislation (which gives legal protection to common dormice and their breeding sites); the paucity of records for broadleaved woodland is therefore unexpected, although the model correctly predicts the highest habitat suitability for this land class. The large number of records for improved grassland and arable and horticultural land classes probably reflects hedgerow populations. The upper limit for predicted total abundance may be inflated by an assumption that densities recorded in preferred habitats (e.g. hedgerows) within improved grassland, arable and horticultural land classes are representative of the density for the land class as a whole.

References:

Harris, S. J., P. Morris, S. Wray and D. Yalden (1995). A review of British mammals: population estimates and conservation status of British mammals other than cetaceans, Joint Nature Conservation Committee, Peterborough, UK.

Trout, R. C., S. E. Brooks, P. Rudlin and J. Neil (2012). The effects of restoring a conifer Plantation on an Ancient Woodland Site (PAWS) in the UK on the habitat and local population of the Hazel Dormouse (*Muscardinus avellanarius*). *European Journal of Wildlife Research* 58(4): 635-643.

Table 1: Summary of observed data and model predictions by land cover class (LCM2007 target classification). Values shown in brackets denote the spatial coverage based on a 10km resolution raster map (number of grid cells). Years represent the median of records within each land class. Ranges for density and abundance are derived using the respective minimum and maximum raster maps (lower bound is mean of values across minimum raster map with upper across the maximum) which capture the spatial uncertainty generate by projecting irregular polygons describing survey sites onto a raster grid.

LCM2007 class	Observed					Predicted		
	Occurrence		Density					
	Records	Year	Estimates	Year	Range	Habitat suitability	Density	Abundance
1 (Broadleaved woodland)	126 (8)	2013	0 (0)	-	-	0.81 (9)	0.37 - 235	331.8 - 211,500
2 (Coniferous woodland)	88 (4)	2013	0 (0)	-	-	0.17 (2)	0.37 - 235	73.73 - 47,000
3 (Arable and Horticultural)	6,823 (306)	2011	1 (1)	2007	0.37 - 235	0.54 (379)	0.34 - 217.2	12,912 - 8,230,944
4 (Improved grassland)	4,236 (257)	2012	0 (0)	-	-	0.5 (288)	0.35 - 221.8	10,021 - 6,388,152
5 (Rough grassland)	0 (0)	-	0 (0)	-	-	0.11 (0)	-	0
6 (Neutral grassland)	0 (0)	-	0 (0)	-	-	0.04 (0)	-	0
7 (Calcareous grassland)	0 (0)	-	0 (0)	-	-	0.71 (2)	0.37 - 235	73.73 - 47,000
8 (Acid grassland)	140 (17)	2010	0 (0)	-	-	0.22 (15)	0.34 - 217.6	512.1 - 326,412
9 (Fen, Marsh, and Swamp)	0 (0)	-	0 (0)	-	-	-	-	0
10 (Heather)	0 (0)	-	0 (0)	-	-	0.11 (0)	-	0
11 (Heather grassland)	0 (0)	-	0 (0)	-	-	0.08 (0)	-	0
12 (Bog)	23 (1)	2011	0 (0)	-	-	0.11 (1)	0.37 - 235	36.87 - 23,500
13 (Montane habitat)	0 (0)	-	0 (0)	-	-	0.07 (0)	-	0
14 (Inland rock)	0 (0)	-	0 (0)	-	-	0.06 (0)	-	0
15 (Saltwater)	0 (0)	-	0 (0)	-	-	0.32 (1)	0.01 - 5.77	0.91 - 577.1
16 (Freshwater)	0 (0)	-	0 (0)	-	-	0.1 (0)	-	0
17 (Supra-littoral rock)	0 (0)	-	0 (0)	-	-	0.06 (0)	-	0
18 (Supra-littoral sediment)	0 (0)	-	0 (0)	-	-	0.16 (0)	-	0
19 (Littoral rock)	0 (0)	-	0 (0)	-	-	0.13 (0)	-	0
20 (Littoral sediment)	7 (3)	1998	0 (0)	-	-	0.31 (0)	-	0
21 (Saltmarsh)	0 (0)	-	0 (0)	-	-	-	-	0
22 (Urban)	0 (0)	-	0 (0)	-	-	0.25 (1)	0.12 - 76.42	11.99 - 7,642
23 (Suburban)	94 (25)	2012	0 (0)	-	-	0.5 (25)	0.3 - 190.3	746.2 - 475,694
Total	11,537 (621)	2011	1 (1)	2007	0.37 - 235	0.4 (723)	0.34 - 218	24,721 - 15,758,420

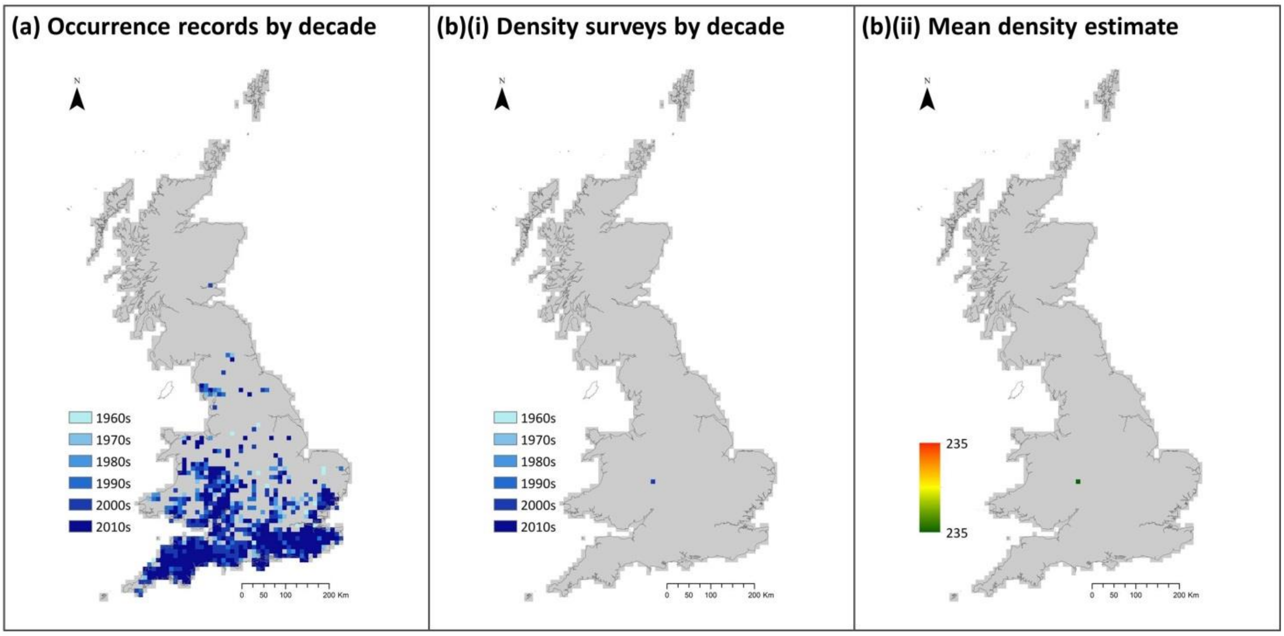

© Crown copyright and database rights 2016 Ordnance Survey 100051110. Data courtesy of the NBN Gateway with thanks to all data contributors. The NBN and its data contributors bear no responsibility for the further analysis or interpretation of this material, data and/or information.

Figure 1: 10km resolution raster maps based on BNG presenting the geographic description of available data. (a) shows the distribution of species occurrence obtained via the NBN Gateway categorised by the decade of last sighting. (b) shows information relating to density surveys identified via a search of published literature where: (i) categorises surveys by the decade of last survey; and (ii) shows the mean density estimate of surveys within grid cells (estimates assumed to be representative of entire cell, considered the upper limit of observed density).

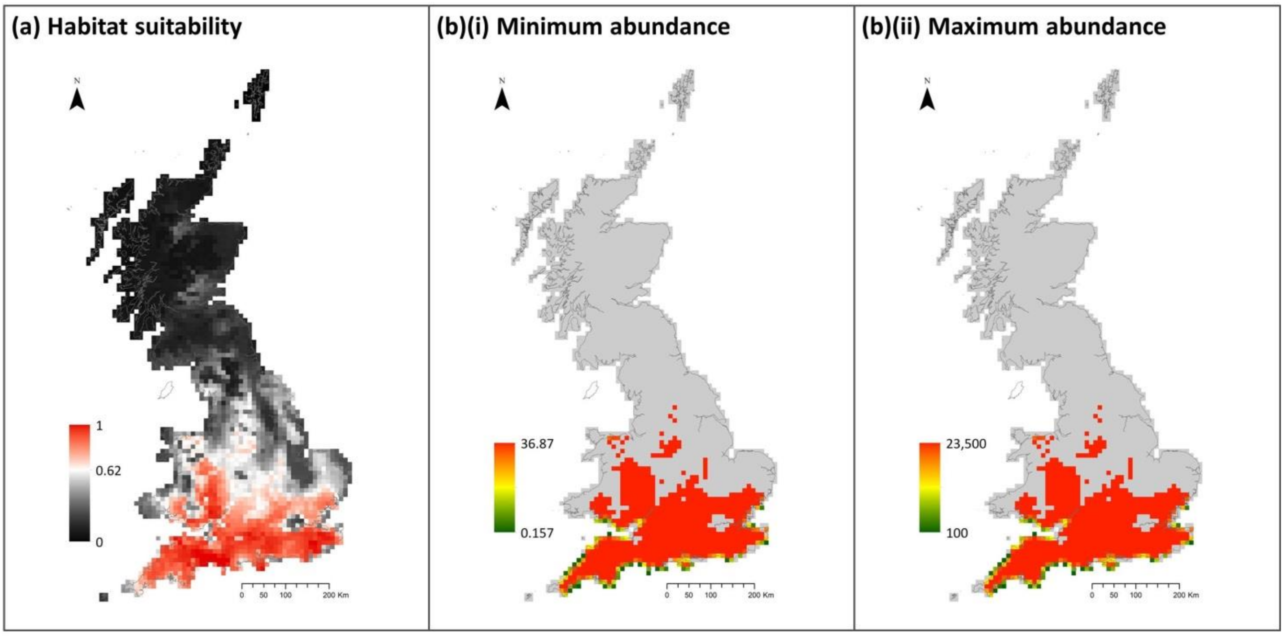

© Crown copyright and database rights 2016 Ordnance Survey 100051110. Data courtesy of the NBN Gateway with thanks to all data contributors. The NBN and its data contributors bear no responsibility for the further analysis or interpretation of this material, data and/or information.

Figure 2: Modelling predictions generated using systematic approach based on available data. (a) shows habitat suitability scores (the likelihood of observing the target species within each grid cell given variation environmental variables) determined by aggregating outputs from the "best" species distribution model (7 models compared) across 100 simulations. Here, the mid value on the scale denotes the threshold score above which occurrence is assumed. (b) shows: (i) the lower bound (Minimum); and (ii) the upper bound (Maximum); of abundance estimates determined by relating observed density (taking into account potential uncertainty) with habitat suitability scores using linear regression.
