# Supplementary material for: A systematic approach to estimate the distribution and total abundance of British mammals
Source: PLoS One. 2017 Jun 28;12(6):e0176339. doi: 10.1371/journal.pone.0176339 (PMC5489149; doi:10.1371/journal.pone.0176339)
Supplement: S9 File — Individual reports for each of the Rodentia species presenting analysis of the available data and subsequent model predictions based on a 10km raster grid. Reports also include expert comment assessing the reliability (and plausibility) of results in the context of existing evidence and popular opinion. (ZIP) [file pone.0176339.s009.zip › C Common rat.pdf]

Common rat (*Rattus norvegicus*)

Order: *Rodentia*

Genus: *Rattus*

Origin: Introduced

Status: Common

1995 abundance estimate: 6,790,000 (4)

Reported population trends: JNCC 2005, NGC 2009 (↑)

Data:

The available occurrence records indicate that the common rat is widespread throughout England with more localised coverage in Wales and Scotland (Figure 1a). These sightings were reported in various habitats (predominantly arable and improved grassland) with the majority of cells where occurrence was observed containing at least one record since 1995. However, the map highlights some areas, particularly in Wales and Scotland, were records are more historic.

From the literature review we identified two studies (Baker et al. 2004; Forman 2005); one conducted in Bristol in 2002; the other in south Wales in 2004 (Figure 1b). Estimates ranged between 6.61 and 238 per km^2 with the highest densities reported in habitat dominated by suburban land cover (12.94 - 238 per km^2 accounting for uncertainty relating to unsurveyed areas within grid cells). Due to the limited coverage of these surveys estimates were unavailable for the majority of dominant land covers where occurrence was reported (marked grey in Table 1) and where estimates were available the relative uncertainty within cells was large.

Model predictions:

The habitat suitability map (Figure 2a) appears to reflect the underlying data well with the set of "best" models predicting presence (and absence) to a mean AUC of 0.77. Overall, across 100 repetitions MaxEnt proved to be the most commonly selected modelling approach displaying the highest AUC 49% of the time followed by Random Forest (28%). By land cover the mean habitat suitability scores suggest observation is most likely in landscapes dominated by urban and suburban habitat (Table 1) but, consistent with recorded sightings, the majority of occurrence is predicted in grid cells dominated by arable and improved grassland (the most common dominant land covers at a 10km scale).

Both minimum and maximum density estimates were best fitted linearly to habitat suitability with no consideration for spatial autocorrelation.

The predicted abundance range contains the estimate from Harris et al. (1995) suggesting no change in the total population. Whilst this result disagrees with recently reported trends there is scope within the range to argue that populations have increased. The range itself is very large due to the uncertainty caused by small survey sites relative to the 10km scale at which modelling is performed. In order to provide more accurate predictions future model analysis could be based on a finer scale raster grid which would better represent the variations in habitat for smaller mammals. Unfortunately, at present this is too unreliable due to access restrictions imposed on occurrence data.

Reliability (Expert comment):

Although they can survive independently of human activities, for example in coastal habitats with sufficient food resources, common rats in Britain are largely commensal and they frequently occupy urban habitats (domestic dwellings, gardens, commercial properties and sewers). The number of observations from urban habitats reported here is low compared to suburban, and this may reflect a greater tendency to record suburban sightings. Common rats also occupy rural habitats, particularly grain storage areas, arable hedgerows and pheasant feeders, and this is consistent with the large number of records for the arable and horticultural land cover class. The upper limit for the range of densities in broadleaved woodland in Britain is unlikely to exceed that for arable and horticultural habitats except where supplementary feeding of pheasants or other game occurs. The large number of records for improved grassland is unexpected, and this almost certainly reflects populations associated with livestock farms (particularly feed storage areas and outbuildings) rather than open pasture land. By combining data from surveys of domestic dwellings, commercial properties, sewers and farm premises occupied by rats, the Food and Environment Research

Agency recently estimated that there are 10.5 million common rats in Britain, which is within the range of estimates presented here.

References:

Baker, P. J., S. M. Funk, M. W. Bruford and S. Harris (2004). Polygynandry in a red fox population: implications for the evolution of group living in canids? *Behavioral Ecology* 15(5): 766 - 778.

Forman, D. W. (2005). An assessment of the local impact of native predators on an established population of British water voles (*Arvicola terrestris*). *Journal of Zoology* 266(3): 221 - 226.

Harris, S. J., P. Morris, S. Wray and D. Yalden (1995). A review of British mammals: population estimates and conservation status of British mammals other than cetaceans, Joint Nature Conservation Committee, Peterborough, UK.

Table 1: Summary of observed data and model predictions by land cover class (LCM2007 target classification). Values shown in brackets denote the spatial coverage based on a 10km resolution raster map (number of grid cells). Years represent the median of records within each land class. Ranges for density and abundance are derived using the respective minimum and maximum raster maps (lower bound is mean of values across minimum raster map with upper across the maximum) which capture the spatial uncertainty generate by projecting irregular polygons describing survey sites onto a raster grid.

LCM2007 class	Observed					Predicted		
	Occurrence		Density					
	Records	Year	Estimates	Year	Range	Habitat suitability	Density	Abundance
1 (Broadleaved woodland)	132 (11)	2008	0 (0)	-	-	0.93 (11)	18.03 - 329	19,829 - 362,010
2 (Coniferous woodland)	232 (87)	1994	0 (0)	-	-	0.67 (47)	1.12 - 20.79	5,271 - 97,722
3 (Arable and Horticultural)	17,335 (869)	2008	0 (0)	-	-	0.9 (953)	13.03 - 238	1,241,885 - 22,717,824
4 (Improved grassland)	7,010 (593)	2006	1 (1)	2004	0.08 - 6.61	0.82 (603)	4.98 - 91.46	300,171 - 5,514,952
5 (Rough grassland)	58 (27)	1994	0 (0)	-	-	0.47 (10)	0.44 - 8.77	437.4 - 8,767
6 (Neutral grassland)	0 (0)	-	0 (0)	-	-	0 (0)	-	0
7 (Calcareous grassland)	15 (2)	2009	0 (0)	-	-	0.89 (2)	6.31 - 119	1,262 - 23,740
8 (Acid grassland)	169 (60)	1994	0 (0)	-	-	0.42 (2)	0	0
9 (Fen, Marsh, and Swamp)	0 (0)	-	0 (0)	-	-	-	-	0
10 (Heather)	34 (14)	1978	0 (0)	-	-	0.44 (0)	-	0
11 (Heather grassland)	208 (52)	1999	0 (0)	-	-	0.47 (4)	0	0
12 (Bog)	97 (42)	1983	0 (0)	-	-	0.4 (5)	0	0
13 (Montane habitat)	18 (11)	1996	0 (0)	-	-	0.32 (0)	-	0
14 (Inland rock)	0 (0)	-	0 (0)	-	-	0.22 (0)	-	0
15 (Saltwater)	20 (8)	1974	0 (0)	-	-	0.8 (10)	0	0
16 (Freshwater)	13 (3)	1994	0 (0)	-	-	0.78 (2)	0	0
17 (Supra - littoral rock)	0 (0)	-	0 (0)	-	-	0.09 (0)	-	0
18 (Supra - littoral sediment)	8 (2)	1990	0 (0)	-	-	0.64 (1)	0	0
19 (Littoral rock)	14 (2)	2010	0 (0)	-	-	0.47 (1)	0	0
20 (Littoral sediment)	158 (24)	2005	0 (0)	-	-	0.8 (29)	0.66 - 12.39	1,921 - 35,943
21 (Saltmarsh)	0 (0)	-	0 (0)	-	-	-	-	0
22 (Urban)	129 (8)	2012	0 (0)	-	-	0.95 (8)	20.8 - 378	16,636 - 302,191
23 (Suburban)	1,958 (73)	2012	1 (1)	2002	12.94 - 238	0.93 (78)	17.59 - 320	137,174 - 2,498,856
Total	27,608 (1,888)	2006	2 (2)	2003	6.51 - 122.3	0.74 (1,766)	9.77 - 179	1,724,587 - 31,562,005

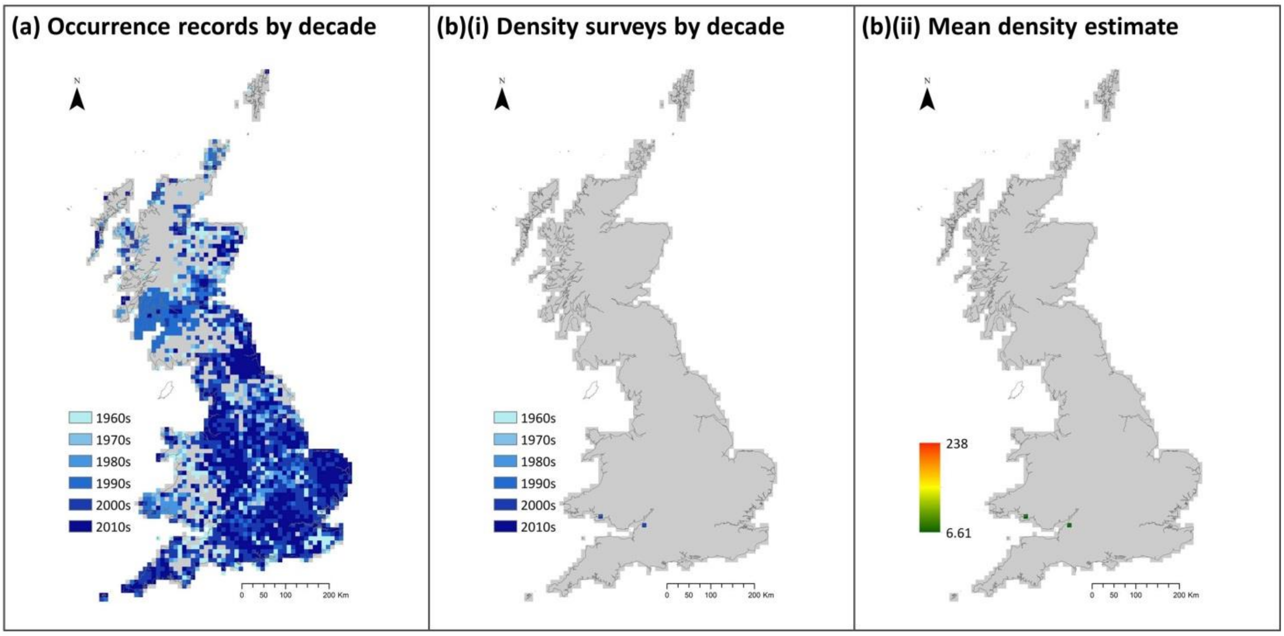

© Crown copyright and database rights 2016 Ordnance Survey 100051110. Data courtesy of the NBN Gateway with thanks to all data contributors. The NBN and its data contributors bear no responsibility for the further analysis or interpretation of this material, data and/or information.

Figure 1: 10km resolution raster maps based on BNG presenting the geographic description of available data. (a) shows the distribution of species occurrence obtained via the NBN Gateway categorised by the decade of last sighting. (b) shows information relating to density surveys identified via a search of published literature where: (i) categorises surveys by the decade of last survey; and (ii) shows the mean density estimate of surveys within grid cells (estimates assumed to be representative of entire cell, considered the upper limit of observed density).

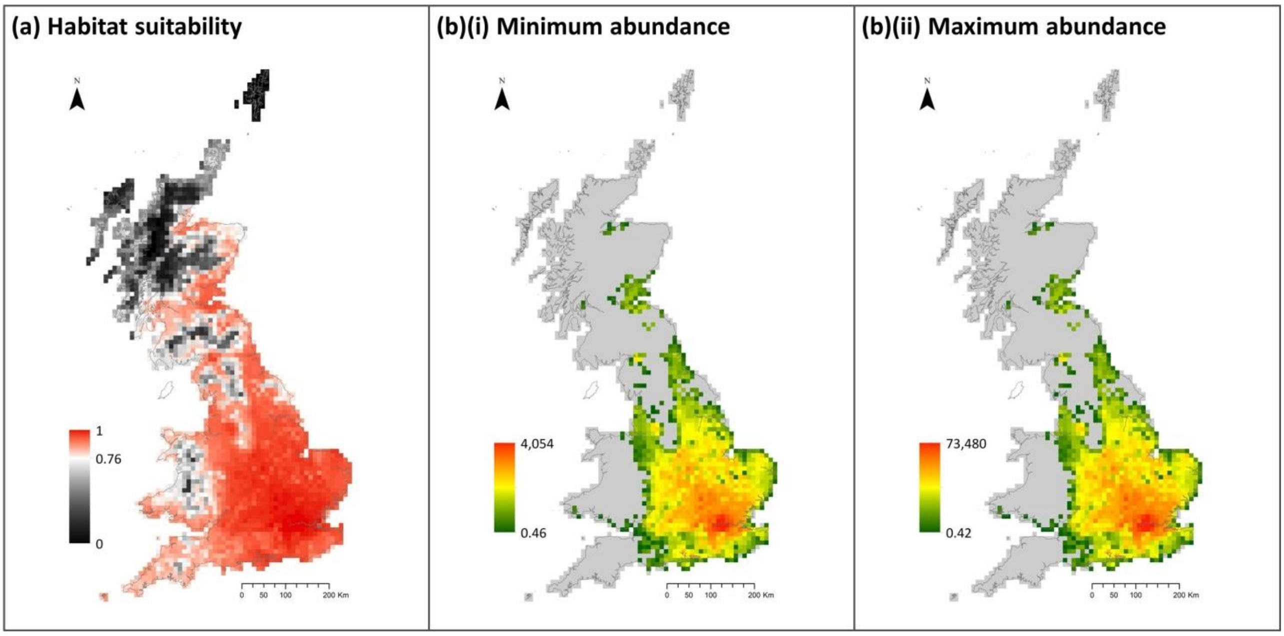

© Crown copyright and database rights 2016 Ordnance Survey 100051110. Data courtesy of the NBN Gateway with thanks to all data contributors. The NBN and its data contributors bear no responsibility for the further analysis or interpretation of this material, data and/or information.

Figure 2: Modelling predictions generated using systematic approach based on available data. (a) shows habitat suitability scores (the likelihood of observing the target species within each grid cell given variation environmental variables) determined by aggregating outputs from the "best" species distribution model (7 models compared) across 100 simulations. Here, the mid value on the scale denotes the threshold score above which occurrence is assumed. (b) shows: (i) the lower bound (Minimum); and (ii) the upper bound (Maximum); of abundance estimates determined by relating observed density (taking into account potential uncertainty) with habitat suitability scores using linear regression.
