# Supplementary material for: A systematic approach to estimate the distribution and total abundance of British mammals
Source: PLoS One. 2017 Jun 28;12(6):e0176339. doi: 10.1371/journal.pone.0176339 (PMC5489149; doi:10.1371/journal.pone.0176339)
Supplement: S9 File — Individual reports for each of the Rodentia species presenting analysis of the available data and subsequent model predictions based on a 10km raster grid. Reports also include expert comment assessing the reliability (and plausibility) of results in the context of existing evidence and popular opinion. (ZIP) [file pone.0176339.s009.zip › D Coypu.pdf]

Coypu (*Myocastor coypus*)

Order: *Rodentia*

Genus: *Myocastor*

Origin: Introduced

Status: Extinct

1995 abundance estimate: 0 (1)

Reported population trends: None

Data:

The available occurrence records indicate that coypu are likely extinct with only two sightings recorded since 1995 (1997 and 2012). Prior to this the distribution of coypu was concentrated in East Anglia with a few isolated populations dotted throughout England (Figure 1a). Sightings were predominantly reported on farmland (arable and improved grassland land cover).

From the literature review we were unable to identify any publications reporting an estimate of density.

Model predictions:

The habitat suitability map (Figure 2a) appears to reflect the underlying data well with the set of "best" models predicting presence (and absence) to a mean AUC of 0.74. Overall, across 100 repetitions Support Vector Machine proved to be the most commonly selected modelling approach displaying the highest AUC 24% of the time closely followed by Generalised Linear Models (23%). By land cover the mean habitat suitability scores suggest observation is most likely in landscapes dominated by arable land (Table 1). Consistent with this, the majority of occurrence is also predicted in arable dominated habitats with the coverage of occurrence in other land covers largely maintained relative to the observed data.

Unfortunately, due to the lack of density estimates model analysis to predict abundance could not be performed.

Reliability (Expert comment):

The coypu is a semi-aquatic rodent native to South America. Feral populations established in Britain from commercial fur farms in the early 20th century; damage to crops, native flora and water management systems prompted two major systematic removal campaigns in Britain, the second of which ended in 1992. The two reported sightings since 1995 confirm that the species is now largely absent from Britain, although one of these sightings was relatively recent (in 2012). The high predicted habitat suitability for the arable and horticultural land class confirms the potential for crop damage, although this is likely to be heavily dependent on access to nearby aquatic habitats, a combination typically found in East Anglia as shown by the habitat suitability map. The species was not recorded in cells dominated by the freshwater land class, which may reflect a preference for small rivers, streams and ponds rather than large expanses of open water.

References:

None

Table 1: Summary of observed data and model predictions by land cover class (LCM2007 target classification). Values shown in brackets denote the spatial coverage based on a 10km resolution raster map (number of grid cells). Years represent the median of records within each land class. Ranges for density and abundance are derived using the respective minimum and maximum raster maps (lower bound is mean of values across minimum raster map with upper across the maximum) which capture the spatial uncertainty generate by projecting irregular polygons describing survey sites onto a raster grid.

LCM2007 class	Observed					Predicted		
	Occurrence		Density					
	Records	Year	Estimates	Year	Range	Habitat suitability	Density	Abundance
1 (Broadleaved woodland)	2 (1)	1961	0 (0)	-	-	0.39 (0)	-	-
2 (Coniferous woodland)	20 (1)	1984	0 (0)	-	-	0.25 (1)	-	-
3 (Arable and Horticultural)	2,165 (168)	1984	0 (0)	-	-	0.52 (251)	-	-
4 (Improved grassland)	79 (14)	1965	0 (0)	-	-	0.32 (8)	-	-
5 (Rough grassland)	35 (1)	1985	0 (0)	-	-	0.2 (1)	-	-
6 (Neutral grassland)	0 (0)	-	0 (0)	-	-	0.14 (0)	-	-
7 (Calcareous grassland)	0 (0)	-	0 (0)	-	-	0.41 (0)	-	-
8 (Acid grassland)	0 (0)	-	0 (0)	-	-	0.23 (0)	-	-
9 (Fen, Marsh, and Swamp)	0 (0)	-	0 (0)	-	-	-	-	-
10 (Heather)	0 (0)	-	0 (0)	-	-	0.24 (0)	-	-
11 (Heather grassland)	0 (0)	-	0 (0)	-	-	0.2 (0)	-	-
12 (Bog)	0 (0)	-	0 (0)	-	-	0.2 (0)	-	-
13 (Montane habitat)	0 (0)	-	0 (0)	-	-	0.22 (0)	-	-
14 (Inland rock)	0 (0)	-	0 (0)	-	-	0.17 (0)	-	-
15 (Saltwater)	1 (1)	1965	0 (0)	-	-	0.37 (0)	-	-
16 (Freshwater)	0 (0)	-	0 (0)	-	-	0.22 (0)	-	-
17 (Supra-littoral rock)	0 (0)	-	0 (0)	-	-	0.15 (0)	-	-
18 (Supra-littoral sediment)	0 (0)	-	0 (0)	-	-	0.28 (0)	-	-
19 (Littoral rock)	0 (0)	-	0 (0)	-	-	0.23 (1)	-	-
20 (Littoral sediment)	8 (5)	1964	0 (0)	-	-	0.43 (4)	-	-
21 (Saltmarsh)	0 (0)	-	0 (0)	-	-	-	-	-
22 (Urban)	0 (0)	-	0 (0)	-	-	0.29 (0)	-	-
23 (Suburban)	2 (2)	1972	0 (0)	-	-	0.32 (0)	-	-
Total	2,312 (193)	1984	0 (0)	-	-	0.36 (266)	-	-

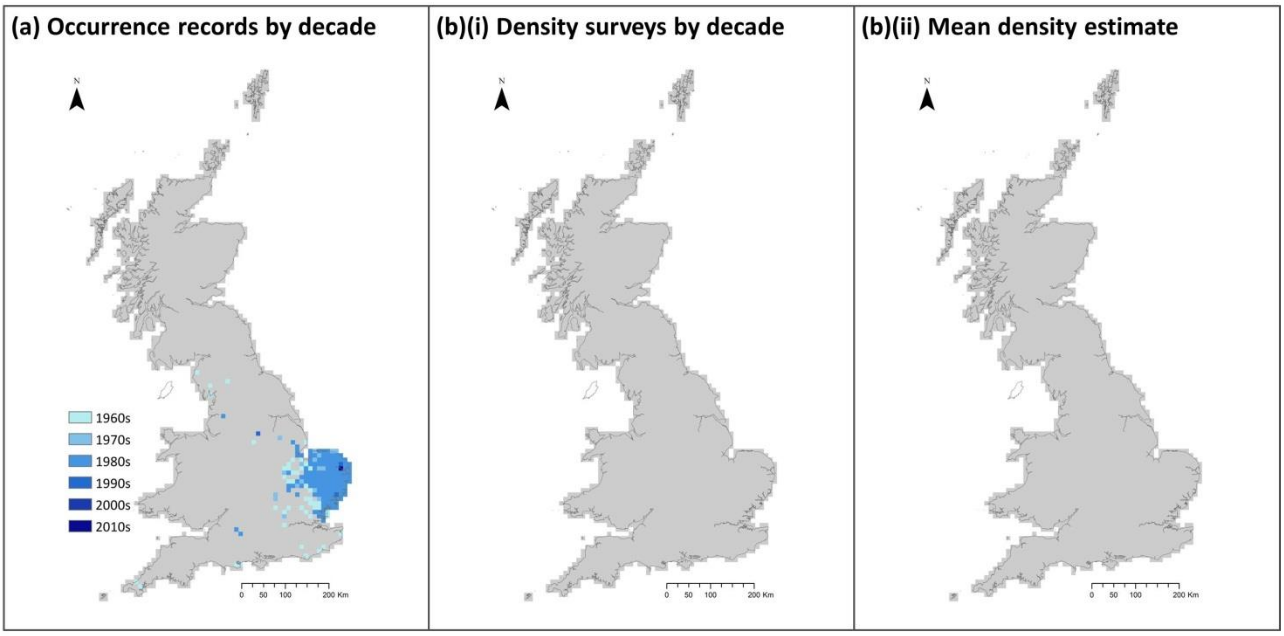

© Crown copyright and database rights 2016 Ordnance Survey 100051110. Data courtesy of the NBN Gateway with thanks to all data contributors. The NBN and its data contributors bear no responsibility for the further analysis or interpretation of this material, data and/or information.

Figure 1: 10km resolution raster maps based on BNG presenting the geographic description of available data. (a) shows the distribution of species occurrence obtained via the NBN Gateway categorised by the decade of last sighting. (b) shows information relating to density surveys identified via a search of published literature where: (i) categorises surveys by the decade of last survey; and (ii) shows the mean density estimate of surveys within grid cells (estimates assumed to be representative of entire cell, considered the upper limit of observed density).

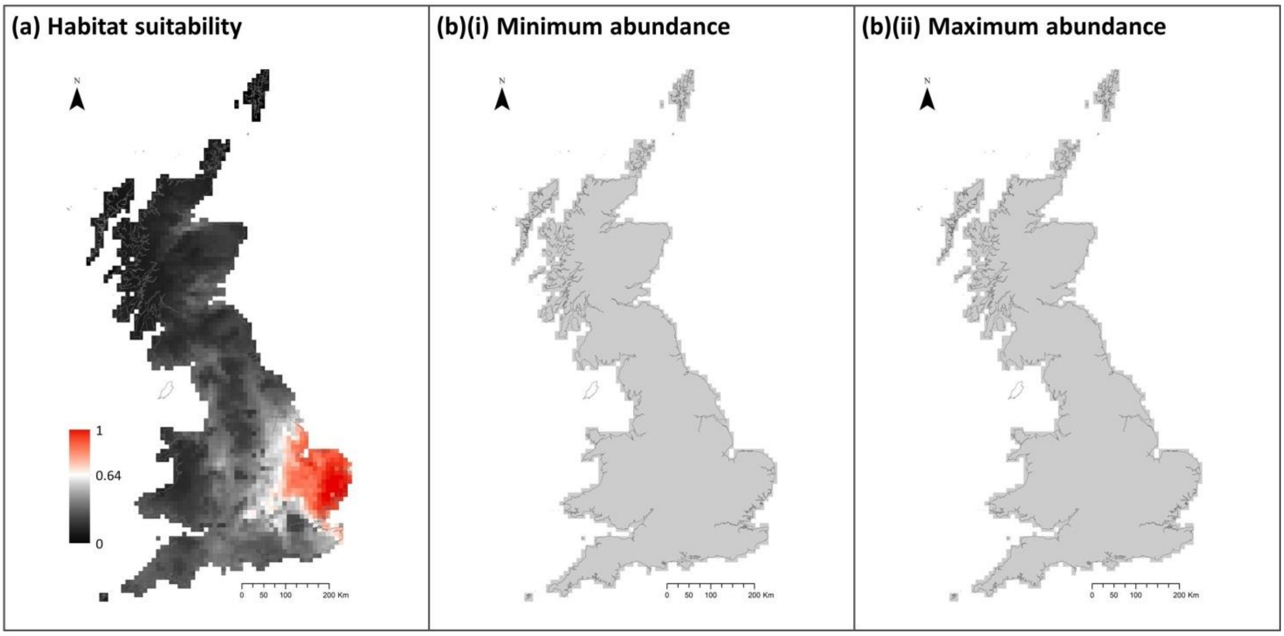

© Crown copyright and database rights 2016 Ordnance Survey 100051110. Data courtesy of the NBN Gateway with thanks to all data contributors. The NBN and its data contributors bear no responsibility for the further analysis or interpretation of this material, data and/or information.

Figure 2: Modelling predictions generated using systematic approach based on available data. (a) shows habitat suitability scores (the likelihood of observing the target species within each grid cell given variation environmental variables) determined by aggregating outputs from the "best" species distribution model (7 models compared) across 100 simulations. Here, the mid value on the scale denotes the threshold score above which occurrence is assumed. (b) shows: (i) the lower bound (Minimum); and (ii) the upper bound (Maximum); of abundance estimates determined by relating observed density (taking into account potential uncertainty) with habitat suitability scores using linear regression.
