# Supplementary material for: A systematic approach to estimate the distribution and total abundance of British mammals
Source: PLoS One. 2017 Jun 28;12(6):e0176339. doi: 10.1371/journal.pone.0176339 (PMC5489149; doi:10.1371/journal.pone.0176339)
Supplement: S9 File — Individual reports for each of the Rodentia species presenting analysis of the available data and subsequent model predictions based on a 10km raster grid. Reports also include expert comment assessing the reliability (and plausibility) of results in the context of existing evidence and popular opinion. (ZIP) [file pone.0176339.s009.zip › E Fat dormouse.pdf]

Fat dormouse (*Glis glis*)

Order: *Rodentia*

Genus: *Glis*

Origin: Introduced

Status: Locally common

1995 abundance estimate: 10,000 (3)

Reported population trends: None

Data:

The available occurrence records indicate that the fat dormouse is locally distributed across the south of England with a single isolated sighting in the north and in Scotland respectively; none were recorded in Wales (Figure 1a). Sightings were only recorded in habitats dominated by arable, improved grassland and suburban land cover with the majority of cells where occurrence has been observed containing at least one record since 1995.

From the literature review we only identified a single study (Burgess et al. 2003) conducted at a high density site in the south east of England in 2001 (Figure 1b). This study reported a density estimate of 235 per km^2 in a single cell dominated by arable land. Due to the limited coverage of the survey within this cell the density range, taking into account the relative uncertainty, was large (2.31 - 235 per km^2).

Model predictions:

The habitat suitability map (Figure 2a) appears to reflect the underlying data well with the set of "best" models predicting presence (and absence) to a mean AUC of 0.79. However, the resulting distribution is substantially larger than the area described by the observations alone (approximately 4 times). Overall, across 100 repetitions MaxEnt proved to be the most commonly selected modelling approach displaying the highest AUC 24% of the time followed by Support Vector Machines (20%). By land cover the mean habitat suitability scores suggest observation is most likely in landscapes dominated by calcareous grassland (Table 1) but, consistent with recorded sightings, the majority of occurrence is predicted in grid cells dominated by arable and improved grassland.

Due to the limited number of density estimates it was not possible to assess any relationship with habitat suitability. Instead, a constant mean estimate was applied across all cells where occurrence was predicted and summed to derive total abundance.

Perhaps unsurprisingly given the inflated distribution and high density estimate, the predicted abundance range does not contain the estimate from Harris et al. (1995); instead suggesting a significant increase in the total population. This overestimation highlights the difficulty of modelling very rare species at such broad scales. Predictions may be more plausible by apply models based on finer resolution raster data where the habitats of small mammal such as this species can be better represented.

Reliability (Expert comment):

The core range for the species does not appear to have expanded substantially over the last 20 years; several recent sightings in the south of England well away from the core range are likely to be releases (translocations) of animals trapped in households where they can cause damage and disturbance. Householders are often reluctant or unable to kill fat dormice caught in live traps, although most translocated animals tend to have low chances of survival and it is unlikely that these sporadic releases will establish new populations outside the core range. Under the Wildlife and Countryside Act 1981 it is illegal to release or permit the release of fat dormice in England Wales and Scotland. Fat dormice are occasionally sold as pets, and recent sightings in northern England and Scotland could be accidental or deliberate releases of pet animals. The lower estimate for total abundance presented here is close to an estimate of 30,000 recently reported in the media, although it is unclear where that figure came from. Despite the largely unchanged range, the population appears to have increased three-fold since 1995.

References:

Burgess, M., P. Morris and P. Bright (2003). Population dynamics of the edible dormouse (*Glis glis*) in England*. Acta Zoologica Academiae Scientarum Hungaricae* 49(1): 27-31.

Harris, S. J., P. Morris, S. Wray and D. Yalden (1995). A review of British mammals: population estimates and conservation status of British mammals other than cetaceans, Joint Nature Conservation Committee, Peterborough, UK.

Table 1: Summary of observed data and model predictions by land cover class (LCM2007 target classification). Values shown in brackets denote the spatial coverage based on a 10km resolution raster map (number of grid cells). Years represent the median of records within each land class. Ranges for density and abundance are derived using the respective minimum and maximum raster maps (lower bound is mean of values across minimum raster map with upper across the maximum) which capture the spatial uncertainty generate by projecting irregular polygons describing survey sites onto a raster grid.

LCM2007 class	Observed					Predicted		
	Occurrence		Density					
	Records	Year	Estimates	Year	Range	Habitat suitability	Density	Abundance
1 (Broadleaved woodland)	0 (0)	-	0 (0)	-	-	0.3 (1)	2.31 - 235	230.9 - 23,500
2 (Coniferous woodland)	0 (0)	-	0 (0)	-	-	0.11 (0)	-	0
3 (Arable and Horticultural)	172 (15)	2008	1 (1)	2001	2.31 - 235	0.31 (71)	2.31 - 235	16,393 - 1,668,500
4 (Improved grassland)	123 (14)	2009	0 (0)	-	-	0.21 (41)	2.31 - 235	9,466 - 963,500
5 (Rough grassland)	0 (0)	-	0 (0)	-	-	0.08 (0)	-	0
6 (Neutral grassland)	0 (0)	-	0 (0)	-	-	0.07 (0)	-	0
7 (Calcareous grassland)	0 (0)	-	0 (0)	-	-	0.34 (0)	-	0
8 (Acid grassland)	0 (0)	-	0 (0)	-	-	0.08 (0)	-	0
9 (Fen, Marsh, and Swamp)	0 (0)	-	0 (0)	-	-	-	-	0
10 (Heather)	0 (0)	-	0 (0)	-	-	0.11 (0)	-	0
11 (Heather grassland)	0 (0)	-	0 (0)	-	-	0.08 (0)	-	0
12 (Bog)	0 (0)	-	0 (0)	-	-	0.09 (0)	-	0
13 (Montane habitat)	0 (0)	-	0 (0)	-	-	0.09 (0)	-	0
14 (Inland rock)	0 (0)	-	0 (0)	-	-	0.04 (0)	-	0
15 (Saltwater)	0 (0)	-	0 (0)	-	-	0.19 (0)	-	0
16 (Freshwater)	0 (0)	-	0 (0)	-	-	0.07 (0)	-	0
17 (Supra-littoral rock)	0 (0)	-	0 (0)	-	-	0.06 (0)	-	0
18 (Supra-littoral sediment)	0 (0)	-	0 (0)	-	-	0.11 (0)	-	0
19 (Littoral rock)	0 (0)	-	0 (0)	-	-	0.12 (0)	-	0
20 (Littoral sediment)	0 (0)	-	0 (0)	-	-	0.21 (0)	-	0
21 (Saltmarsh)	0 (0)	-	0 (0)	-	-	-	-	0
22 (Urban)	0 (0)	-	0 (0)	-	-	0.29 (0)	-	0
23 (Suburban)	14 (3)	2006	0 (0)	-	-	0.31 (11)	2.31 - 235	2,540 - 258,500
Total	309 (32)	2008	1 (1)	2001	2.31 - 235	0.21 (124)	2.31 - 235	28,630 - 2,914,000

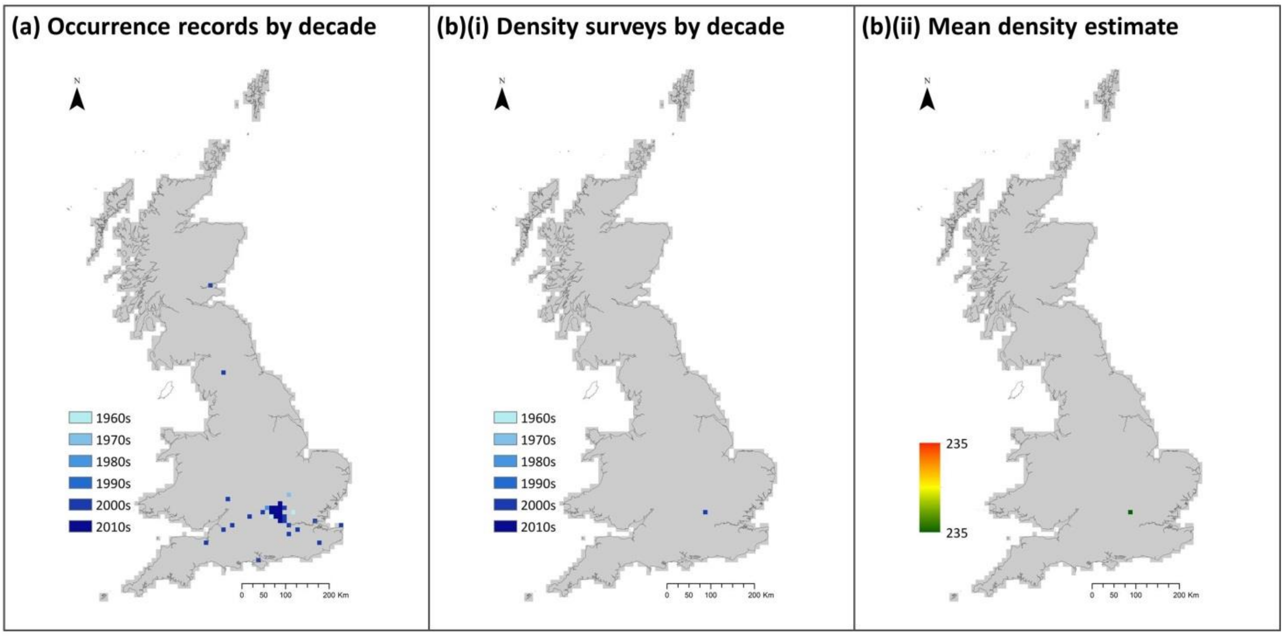

© Crown copyright and database rights 2016 Ordnance Survey 100051110. Data courtesy of the NBN Gateway with thanks to all data contributors. The NBN and its data contributors bear no responsibility for the further analysis or interpretation of this material, data and/or information.

Figure 1: 10km resolution raster maps based on BNG presenting the geographic description of available data. (a) shows the distribution of species occurrence obtained via the NBN Gateway categorised by the decade of last sighting. (b) shows information relating to density surveys identified via a search of published literature where: (i) categorises surveys by the decade of last survey; and (ii) shows the mean density estimate of surveys within grid cells (estimates assumed to be representative of entire cell, considered the upper limit of observed density).

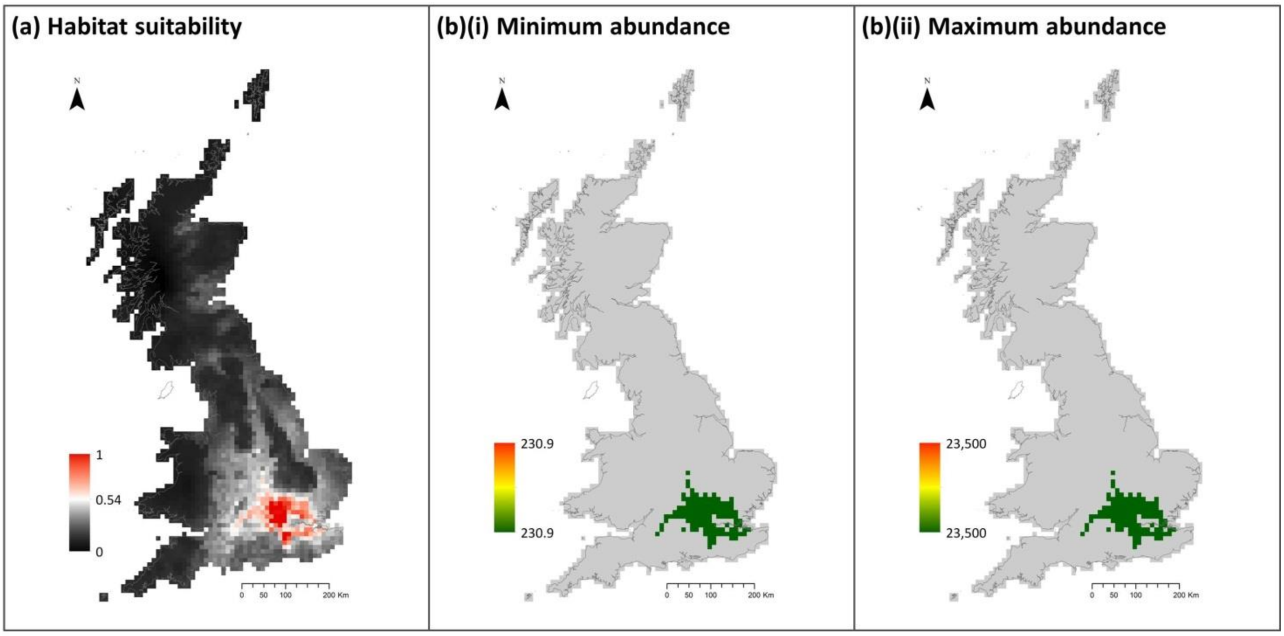

© Crown copyright and database rights 2016 Ordnance Survey 100051110. Data courtesy of the NBN Gateway with thanks to all data contributors. The NBN and its data contributors bear no responsibility for the further analysis or interpretation of this material, data and/or information.

Figure 2: Modelling predictions generated using systematic approach based on available data. (a) shows habitat suitability scores (the likelihood of observing the target species within each grid cell given variation environmental variables) determined by aggregating outputs from the "best" species distribution model (7 models compared) across 100 simulations. Here, the mid value on the scale denotes the threshold score above which occurrence is assumed. (b) shows: (i) the lower bound (Minimum); and (ii) the upper bound (Maximum); of abundance estimates determined by relating observed density (taking into account potential uncertainty) with habitat suitability scores using linear regression.
