# Supplementary material for: A systematic approach to estimate the distribution and total abundance of British mammals
Source: PLoS One. 2017 Jun 28;12(6):e0176339. doi: 10.1371/journal.pone.0176339 (PMC5489149; doi:10.1371/journal.pone.0176339)
Supplement: S9 File — Individual reports for each of the Rodentia species presenting analysis of the available data and subsequent model predictions based on a 10km raster grid. Reports also include expert comment assessing the reliability (and plausibility) of results in the context of existing evidence and popular opinion. (ZIP) [file pone.0176339.s009.zip › F Field vole.pdf]

Field vole (*Microtus agrestis*)

Order: *Rodentia*

Genus: *Microtus*

Origin: Native

Status: Locally common

1995 abundance estimate: 75,000,000 (4)

Reported population trends: None

Data:

The available occurrence records (Figure 1a) indicate that the field vole is widespread throughout GB with observation in various habitats (primarily those dominated by arable and improved grassland). However, the map highlights localised patches where the species has not been recorded for some time (south east England and Wales) or not at all (Scotland).

From the literature review we identified several studies (Burthe et al. 2006; Forman 2005; Gelling et al. 2007; Kotzageorgis & Mason 1997; Lambin et al. 2000; O'Mahony et al. 1999; Shore et al. 2005; Tattersall et al. 2002) conducted over the last two decades and located across the range of observed occurrence (Figure 1b). Estimates ranged between 2.02 and 22,976 per km^2 with the highest densities reported in coniferous woodland dominated land cover (187.3 - 7,476 per km^2 accounting for uncertainty relating to unsurveyed areas within grid cells). Due to the limited coverage of these surveys estimates were unavailable for several dominant land covers where occurrence was reported (marked grey in Table 1) and where estimates were available the relative uncertainty within cells was large.

Model predictions:

The habitat suitability map (Figure 2a) appears to reflect the underlying data reasonably well with the set of "best" models predicting presence (and absence) to a mean AUC of 0.67. However, the patches of occurrence in northern England and Scotland are less well captured. Overall, across 100 repetitions MaxEnt proved to be the most commonly selected modelling approach displaying the highest AUC 36% of the time closely followed by Random Forest (33%). By land cover the mean habitat suitability scores suggest observation is most likely in landscapes dominated by suburban land (Table 1) but, consistent with recorded sightings, the majority of occurrence is predicted in grid cells dominated by arable and improved grassland.

Both minimum and maximum density estimates were best fitted to the square of habitat suitability accounting for spherical spatial autocorrelation. Interestingly, these relationships appear to suggest a negative correlation in which squares with a higher likelihood of observation contain a lower density of individuals. This explains the inconsistency between the distributions of abundance as where predictions of density in cells of high suitability became negative they were removed (set to zero abundance). Although the best fit models have the same structure the specific parameterisation is not and hence negative cells appear in different locations. It is most likely that the quality and quantity of density estimates are responsible for this result.

Nevertheless, the predicted abundance range contain the estimate from Harris et al. (1995) suggesting no change in the total population (since the median year of observed density estimates is around 1995 then this result is likely to indicate no significant change in spatial distribution over the past 20 years). However, the range is very large due to the uncertainty caused by small survey sites relative to the 10km scale at which modelling is performed. In order to provide more accurate results additional model analysis would be required based on a finer scale raster grid which better represents the variations in habitat for smaller mammals. Unfortunately, at present this is too unreliable due to restrictions imposed on occurrence data.

Reliability (Expert comment):

Field voles are herbivorous and graze primarily on leaves and stems of grasses, the diet may also contain herbs, grass seeds and plant storage organs. The majority of recorded observations were from arable, horticultural and improved grassland. Population densities reported for these habitats was low; crop production and livestock grazing usually produce unfavourable habitats for field voles (although two field vole 'plagues' were reported across large areas of

upland sheep grazing in Scotland in the late 19th century). A higher density was reported for coniferous woodland and it is known that the diet of field voles includes tree bark, sometimes to the extent that in Scandinavia (and also some parts of central Europe) field voles are regarded as forestry pests of major economic importance. In Britain, young forestry plantations with dense ground cover provide suitable field vole habitats, although vole populations decline as the maturing tree canopy shades out the understory plant species, and their impact on forestry in Britain is not thought to be significant. Surprisingly few observations were reported for rough grassland, although, as would be expected, predicted density for rough grassland was relatively high. Predicted density was also high for other grassland categories and typical upland habitats, which seems reasonable, although an unexpectedly high upper limit for field vole density in freshwater habitats was predicted.

References:

Burthe, S., S. Telfer, X. Lambin, M. Bennett, D. Carslake, A. Smith and M. Begon (2006). Cowpox virus infection in natural field vole *Microtus agrestis* populations: delayed density dependence and individual risk. *Journal of Animal Ecology* 75(6): 1416-1425.

Forman, D. W. (2005). An assessment of the local impact of native predators on an established population of British water voles (*Arvicola terrestris*). *Journal of Zoology* 266(3): 221-226.

Gelling, M., D. W. Macdonald and F. Mathews (2007). Are hedgerows the route to increased farmland small mammal density? Use of hedgerows in British pastoral habitats. *Landscape Ecology* 22(7): 1019-1032.

Harris, S. J., P. Morris, S. Wray and D. Yalden (1995). A review of British mammals: population estimates and conservation status of British mammals other than cetaceans, Joint Nature Conservation Committee, Peterborough, UK.

Kotzageorgis, G. C. and C. F. Mason (1997). Small mammal populations in relation to hedgerow structure in an arable landscape. *Journal of Zoology* 242(3): 425-434.

Lambin, X., S. J. Petty and J. L. Mackinnon (2000). Cyclic dynamics in field vole populations and generalist predation. *Journal of Animal Ecology* 69(1): 106-119.

O'Mahony, D., X. Lambin, J. L. MacKinnon and C. Coles (1999). Fox predation on cyclic field vole populations in Britain. *Ecography* 22(5): 575-581.

Shore, R. F., W. R. Meek, T. H. Sparks, R. F. Pywell and M. Nowakowski (2005). Will environmental stewardship enhance small mammal abundance on intensively managed farmland? *Mammal Review* 35(3-4): 277-284.

Tattersall, F. H., D. W. Macdonald, B. J. Hart, P. Johnson, W. Manley and R. Feber (2002). Is habitat linearity important for small mammal communities on farmland? *Journal of Applied Ecology* 39(4): 643-652.

Table 1: Summary of observed data and model predictions by land cover class (LCM2007 target classification). Values shown in brackets denote the spatial coverage based on a 10km resolution raster map (number of grid cells). Years represent the median of records within each land class. Ranges for density and abundance are derived using the respective minimum and maximum raster maps (lower bound is mean of values across minimum raster map with upper across the maximum) which capture the spatial uncertainty generate by projecting irregular polygons describing survey sites onto a raster grid.

LCM2007 class	Observed					Predicted		
	Occurrence		Density					
	Records	Year	Estimates	Year	Range	Habitat suitability	Density	Abundance
1 (Broadleaved woodland)	75 (9)	1974	0 (0)	-	-	0.86 (10)	40.4 - 3,614	40,395 - 3,614,343
2 (Coniferous woodland)	564 (118)	1994	23 (9)	1997	187 - 7,476	0.83 (97)	26.8 - 3,874	259,981 - 37,581,982
3 (Arable and Horticultural)	6,579 (746)	2005	8 (6)	1999	0.22 - 8.49	0.87 (830)	34.27 - 1,887	2,844,727 - 156,630,096
4 (Improved grassland)	3,130 (537)	1999	11 (10)	2003	0.45 - 14.1	0.82 (530)	26.85 - 3,402	1,422,897 - 180,292,669
5 (Rough grassland)	53 (21)	1994	0 (0)	-	-	0.45 (6)	16.82 - 6,189	10,091 - 3,713,303
6 (Neutral grassland)	0 (0)	-	0 (0)	-	-	0.02 (0)	-	0
7 (Calcareous grassland)	28 (2)	2013	0 (0)	-	-	0.87 (2)	51.02 - 2,685	10,204 - 536,996
8 (Acid grassland)	352 (111)	1999	0 (0)	-	-	0.75 (27)	1.5 - 8,389	4,043 - 22,650,558
9 (Fen, Marsh, and Swamp)	0 (0)	-	0 (0)	-	-	-	-	0
10 (Heather)	68 (34)	2003	0 (0)	-	-	0.76 (15)	5.73 - 6,856	8,590 - 1,0284,173
11 (Heather grassland)	730 (73)	2007	0 (0)	-	-	0.65 (24)	8.33 - 6,375	19,990 - 15,300,658
12 (Bog)	205 (69)	2005	0 (0)	-	-	0.56 (28)	25.67 - 4,339	71,871 - 12,150,202
13 (Montane habitat)	49 (21)	2002	0 (0)	-	-	0.65 (4)	5.41 - 7,428	2,163 - 2,971,234
14 (Inland rock)	2 (1)	1974	0 (0)	-	-	0.72 (0)	-	0
15 (Saltwater)	40 (6)	1996	0 (0)	-	-	0.76 (0)	-	0
16 (Freshwater)	4 (2)	1987	0 (0)	-	-	0.74 (1)	0 - 8,283	0 - 828,326
17 (Supra-littoral rock)	0 (0)	-	0 (0)	-	-	0.13 (0)	-	0
18 (Supra-littoral sediment)	13 (2)	1988	0 (0)	-	-	0.62 (1)	0 - 1,937	0 - 193,691
19 (Littoral rock)	2 (1)	2012	0 (0)	-	-	0.48 (0)	-	0
20 (Littoral sediment)	150 (24)	1996	0 (0)	-	-	0.81 (23)	18.22 - 2,333	41,914 - 5,366,804
21 (Saltmarsh)	0 (0)	-	0 (0)	-	-	-	-	0
22 (Urban)	61 (8)	1980	0 (0)	-	-	0.93 (8)	7.97 - 3,254	6,373 - 2,603,016
23 (Suburban)	513 (66)	1976	0 (0)	-	-	0.91 (76)	17.45 - 1,178	132,605 - 8,953,670
Total	12,618 (1,851)	2002	42 (25)	1997	68 - 2,699	0.79 (1,682)	28.99 - 2,757	4,875,844 - 463,671,721

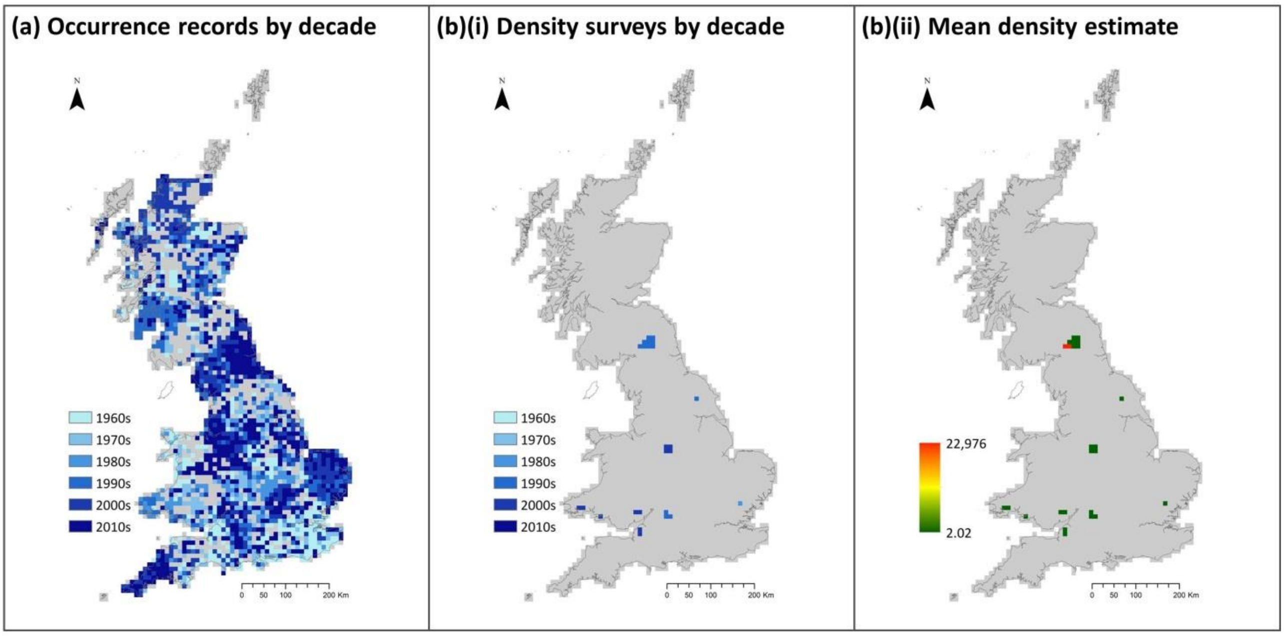

© Crown copyright and database rights 2016 Ordnance Survey 100051110. Data courtesy of the NBN Gateway with thanks to all data contributors. The NBN and its data contributors bear no responsibility for the further analysis or interpretation of this material, data and/or information.

Figure 1: 10km resolution raster maps based on BNG presenting the geographic description of available data. (a) shows the distribution of species occurrence obtained via the NBN Gateway categorised by the decade of last sighting. (b) shows information relating to density surveys identified via a search of published literature where: (i) categorises surveys by the decade of last survey; and (ii) shows the mean density estimate of surveys within grid cells (estimates assumed to be representative of entire cell, considered the upper limit of observed density).

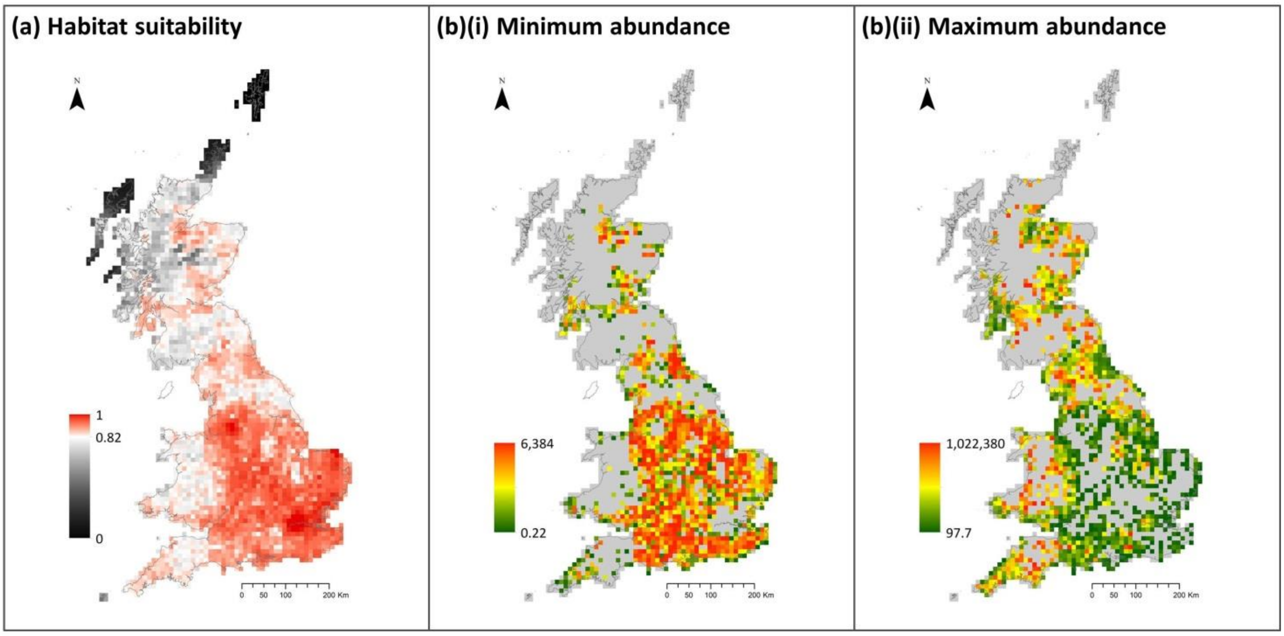

© Crown copyright and database rights 2016 Ordnance Survey 100051110. Data courtesy of the NBN Gateway with thanks to all data contributors. The NBN and its data contributors bear no responsibility for the further analysis or interpretation of this material, data and/or information.

Figure 2: Modelling predictions generated using systematic approach based on available data. (a) shows habitat suitability scores (the likelihood of observing the target species within each grid cell given variation environmental variables) determined by aggregating outputs from the "best" species distribution model (7 models compared) across 100 simulations. Here, the mid value on the scale denotes the threshold score above which occurrence is assumed. (b) shows: (i) the lower bound (Minimum); and (ii) the upper bound (Maximum); of abundance estimates determined by relating observed density (taking into account potential uncertainty) with habitat suitability scores using linear regression.
