# Supplementary material for: A systematic approach to estimate the distribution and total abundance of British mammals
Source: PLoS One. 2017 Jun 28;12(6):e0176339. doi: 10.1371/journal.pone.0176339 (PMC5489149; doi:10.1371/journal.pone.0176339)
Supplement: S9 File — Individual reports for each of the Rodentia species presenting analysis of the available data and subsequent model predictions based on a 10km raster grid. Reports also include expert comment assessing the reliability (and plausibility) of results in the context of existing evidence and popular opinion. (ZIP) [file pone.0176339.s009.zip › G Grey squirrel.pdf]

Grey squirrel (*Sciurus carolinensis*)

Order: *Rodentia*

Genus: *Sciurus*

Origin: Introduced

Status: Common

1995 abundance estimate: 2,520,000 (3)

Reported population trends: JNCC 2005, NGC 2009 (↑), BBS 2014 (↔)

Data:

The available occurrence records indicate that the grey squirrel is widespread throughout Britain (Figure 1a). However, the map highlights a notable region of absence in the north of Scotland. Sightings were reported in various habitats (predominantly arable and improved grassland) with the majority of cells where occurrence was observed containing at least one record since 1995.

From the literature review we identified several studies (Bryce et al. 2002; Gurnell et al 2004a, 2004b; Kenward et al. 1998; Wauters et al. 2000) conducted at different locations across the range of observed occurrence between 1987 and 2000 (Figure 1b). Estimates ranged between 8 and 169.3 per km^2 with the highest densities reported in arable dominated land cover (9.07 - 99.46 per km^2 accounting for uncertainty relating to unsurveyed areas within grid cells). Due to the limited coverage of these surveys estimates were unavailable for several dominant land covers where occurrence was reported (marked grey in Table 1) and where estimates were available the relative uncertainty within cells was large.

Model predictions:

The habitat suitability map (Figure 2a) appears to reflect the underlying data very well with the set of "best" models predicting presence (and absence) to a mean AUC of 0.81. Overall, across 100 repetitions MaxEnt and Random Forest proved to be the most commonly selected modelling approach displaying the highest AUC 29% of the time. By land cover the mean habitat suitability scores suggest observation is most likely in landscapes dominated by calcareous grassland (Table 1) but, consistent with recorded sightings, the majority of occurrence is predicted in grid cells dominated by improved grassland.

Linear regression suggested that there was no correlation between the estimates of minimum density and habitat suitability; consequently density was applied as a constant in cells where occurrence is predicted. Maximum density was found to be correlated with the best fit model relating the square of habitat suitability accounting for spherical spatial autocorrelation. This inconsistency may be caused by scaling bias effecting the relative magnitude of minimum density.

The predicted abundance range contains the estimate from Harris et al. (1995) suggesting, in agreement with the most recent reported trend, no change in the total population (since the median year of observed density estimates is around 1995 then this result may suggest no significant change in spatial distribution over the past 20 years). However, as is the case for all small mammal predictions the range is very large due to the uncertainty caused by small survey sites relative to the 10km scale at which modelling is performed.

Reliability (Expert comment):

Over the last 20 years the range of the grey squirrel has extended northwards in Britain, and south from central Scotland, filling in the previous gap either side of the England-Scotland borders reported by Arnold (1993). The populations in central Scotland have not greatly expanded their range northwards and grey squirrels are still largely absent from the Scottish Highlands. High upper limits for densities in arable, horticultural and improved grassland account for the possibility that 10km squares dominated by these land classes may contain up to 50% of favourable habitat (e.g. woodland), leading to a relatively wide range of abundance estimates.

References:

Arnold, H. R. (1993). Atlas of mammals in Britain: HMSO.

Bryce, J., P. J. Johnson and D. W. Macdonald (2002). Can niche use in red and grey squirrels offer clues for their apparent coexistence*? Journal of Applied Ecology* 39(6): 875-887.

Gurnell, J., P. W. W. Lurz, M. D. F. Shirley, S. Cartmel, P. J. Garson, L. Magris and J. Steele (2004a). Monitoring red squirrels *Sciurus vulgaris* and grey squirrels *Sciurus carolinensis* in Britain. *Mammal Review* 34(1-2): 51-74.

Gurnell, J., L. A. Wauters, P. W. W. Lurz and G. Tosi (2004b). Alien species and interspecific competition: effects of introduced eastern grey squirrels on red squirrel population dynamics. *Journal of Animal Ecology* 73(1): 26-35.

Harris, S. J., P. Morris, S. Wray and D. Yalden (1995). A review of British mammals: population estimates and conservation status of British mammals other than cetaceans, Joint Nature Conservation Committee, Peterborough, UK.

Kenward, R. E., K. H. Hodder, R. J. Rose, C. A. Walls, T. Parish, J. L. Holm, P. A. Morris, S. S. Walls and F. I. Doyle (1998). Comparative demography of red squirrels (*Sciurus vulgaris*) and grey squirrels (*Sciurus carolinensis*) in deciduous and conifer woodland. *Journal of Zoology* 244(1): 7-21.

Wauters, L. A., P. W. W. Lurz and J. Gurnell (2000). Interspecific effects of grey squirrels (*Sciurus carolinensis*) on the space use and population demography of red squirrels (*Sciurus vulgaris*) in conifer plantations. *Ecological Research* 15(3): 271-284.

Table 1: Summary of observed data and model predictions by land cover class (LCM2007 target classification). Values shown in brackets denote the spatial coverage based on a 10km resolution raster map (number of grid cells). Years represent the median of records within each land class. Ranges for density and abundance are derived using the respective minimum and maximum raster maps (lower bound is mean of values across minimum raster map with upper across the maximum) which capture the spatial uncertainty generate by projecting irregular polygons describing survey sites onto a raster grid.

LCM2007 class	Observed					Predicted		
	Occurrence		Density					
	Records	Year	Estimates	Year	Range	Habitat suitability	Density	Abundance
1 (Broadleaved woodland)	1,898 (11)	2013	0 (0)	-	-	0.92 (11)	8.89 - 86.47	9,778 - 95,118
2 (Coniferous woodland)	3,097 (78)	2012	2 (2)	1999	21.7 - 45	0.69 (58)	8.88 - 81.15	51,493 - 470,660
3 (Arable and Horticultural)	61,363 (901)	2013	5 (5)	1997	9.1 - 99.5	0.92 (914)	8.22 - 77.57	751,252 - 7,089,934
4 (Improved grassland)	52,909 (653)	2013	9 (5)	1998	3.1 - 81	0.86 (646)	8.27 - 77.97	534,371 - 5,036,545
5 (Rough grassland)	440 (12)	2014	1 (1)	1997	11.3 - 82	0.28 (7)	8.84 - 84.31	6,189 - 59,016
6 (Neutral grassland)	0 (0)	-	0 (0)	-	-	0 (0)	-	0
7 (Calcareous grassland)	40 (2)	2010	0 (0)	-	-	0.96 (2)	8.89 - 83.33	1,778 - 16,666
8 (Acid grassland)	4,988 (116)	2011	0 (0)	-	-	0.64 (100)	8.79 - 82.14	87,905 - 821,358
9 (Fen, Marsh, and Swamp)	0 (0)	-	0 (0)	-	-	-	-	0
10 (Heather)	195 (18)	2006	0 (0)	-	-	0.54 (9)	8.89 - 82.81	8,000 - 74,530
11 (Heather grassland)	638 (20)	2010	0 (0)	-	-	0.29 (13)	8.89 - 76.57	11,556 - 99,535
12 (Bog)	756 (23)	2012	0 (0)	-	-	0.27 (17)	8.89 - 81.87	15,112 - 139,178
13 (Montane habitat)	22 (4)	2008	0 (0)	-	-	0.26 (0)	-	0
14 (Inland rock)	0 (0)	-	0 (0)	-	-	0.11 (0)	-	0
15 (Saltwater)	152 (5)	2013	0 (0)	-	-	0.73 (0)	-	0
16 (Freshwater)	276 (2)	2012	0 (0)	-	-	0.59 (1)	8.74 - 77.6	873.5 - 7,760
17 (Supra-littoral rock)	0 (0)	-	0 (0)	-	-	0.07 (0)	-	0
18 (Supra-littoral sediment)	0 (0)	-	0 (0)	-	-	0.39 (0)	-	0
19 (Littoral rock)	1 (1)	2011	0 (0)	-	-	0.31 (0)	-	0
20 (Littoral sediment)	1,232 (25)	2011	0 (0)	-	-	0.78 (12)	6.19 - 49.37	7,431 - 59,242
21 (Saltmarsh)	0 (0)	-	0 (0)	-	-	-	-	0
22 (Urban)	1,126 (7)	2014	0 (0)	-	-	0.86 (6)	7.31 - 67.81	4,386 - 40,684
23 (Suburban)	20,150 (76)	2014	0 (0)	-	-	0.87 (74)	7.53 - 67.78	55,726 - 501,605
Total	149,283 (1,954)	2013	17 (13)	1997	8.9 - 82.6	0.75 (1,870)	8.27 - 77.6	1,545,851 - 14,511,831

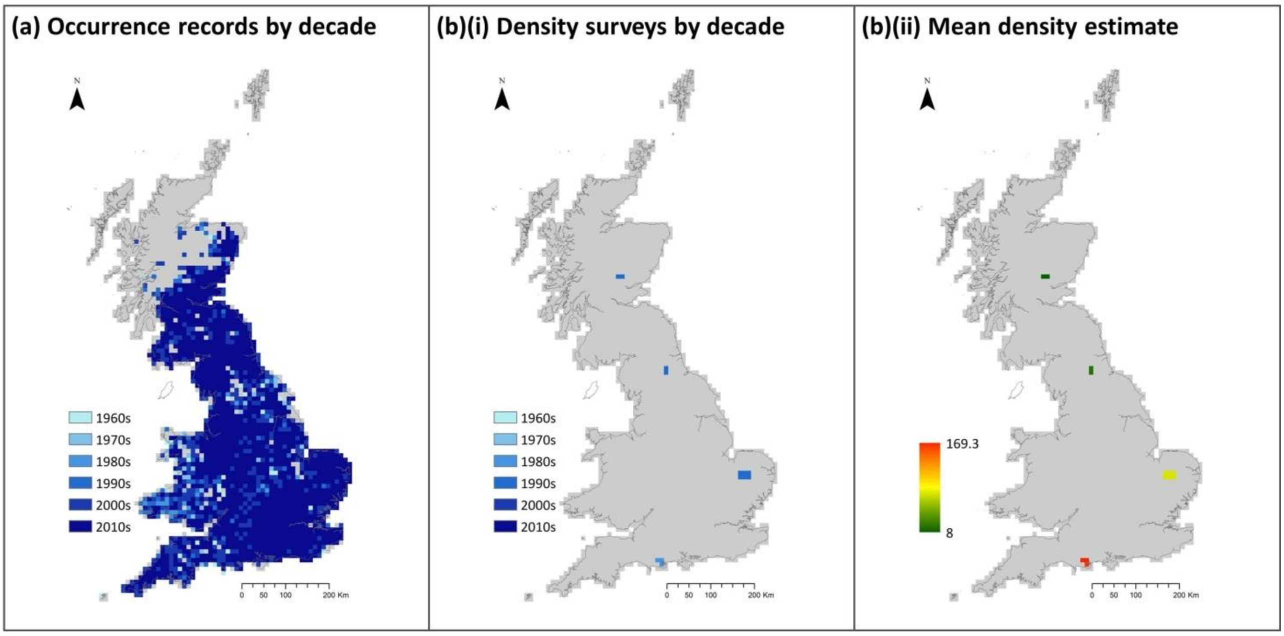

© Crown copyright and database rights 2016 Ordnance Survey 100051110. Data courtesy of the NBN Gateway with thanks to all data contributors. The NBN and its data contributors bear no responsibility for the further analysis or interpretation of this material, data and/or information.

Figure 1: 10km resolution raster maps based on BNG presenting the geographic description of available data. (a) shows the distribution of species occurrence obtained via the NBN Gateway categorised by the decade of last sighting. (b) shows information relating to density surveys identified via a search of published literature where: (i) categorises surveys by the decade of last survey; and (ii) shows the mean density estimate of surveys within grid cells (estimates assumed to be representative of entire cell, considered the upper limit of observed density).

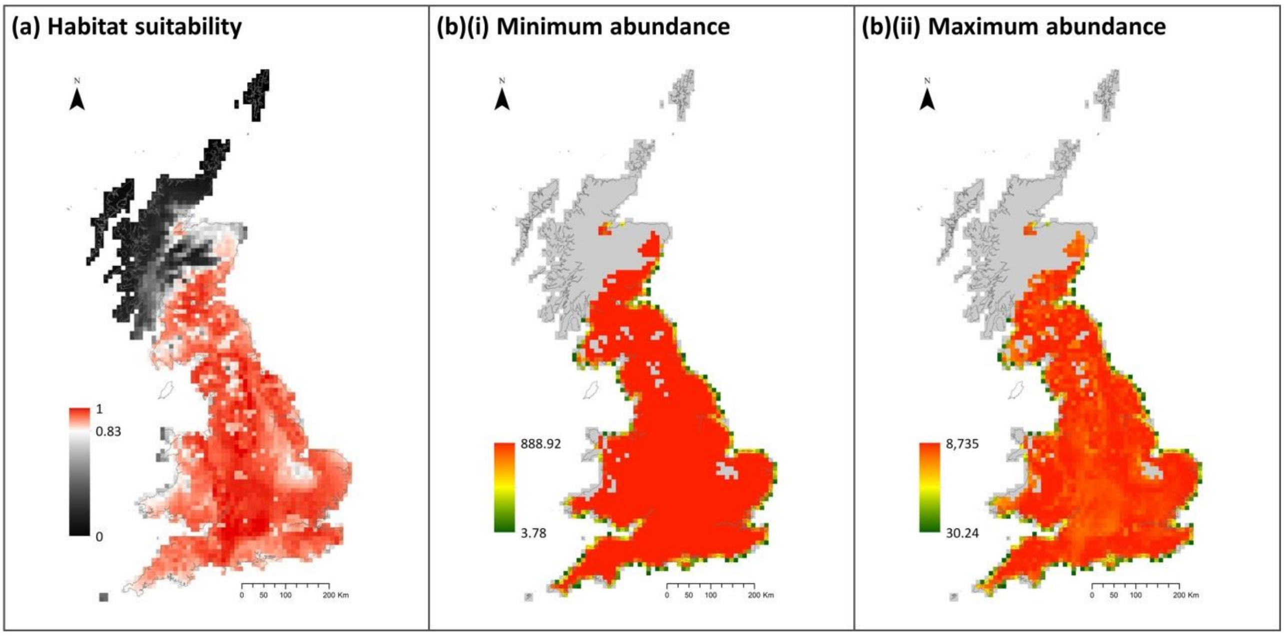

© Crown copyright and database rights 2016 Ordnance Survey 100051110. Data courtesy of the NBN Gateway with thanks to all data contributors. The NBN and its data contributors bear no responsibility for the further analysis or interpretation of this material, data and/or information.

Figure 2: Modelling predictions generated using systematic approach based on available data. (a) shows habitat suitability scores (the likelihood of observing the target species within each grid cell given variation environmental variables) determined by aggregating outputs from the "best" species distribution model (7 models compared) across 100 simulations. Here, the mid value on the scale denotes the threshold score above which occurrence is assumed. (b) shows: (i) the lower bound (Minimum); and (ii) the upper bound (Maximum); of abundance estimates determined by relating observed density (taking into account potential uncertainty) with habitat suitability scores using linear regression.
