# Supplementary material for: A systematic approach to estimate the distribution and total abundance of British mammals
Source: PLoS One. 2017 Jun 28;12(6):e0176339. doi: 10.1371/journal.pone.0176339 (PMC5489149; doi:10.1371/journal.pone.0176339)
Supplement: S9 File — Individual reports for each of the Rodentia species presenting analysis of the available data and subsequent model predictions based on a 10km raster grid. Reports also include expert comment assessing the reliability (and plausibility) of results in the context of existing evidence and popular opinion. (ZIP) [file pone.0176339.s009.zip › H Harvest mouse.pdf]

Harvest mouse (*Micromys minutus*)

Order: *Rodentia*

Genus: *Micromys*

Origin: Introduced

Status: Locally common

1995 abundance estimate: 1,425,000 (5)

Reported population trends: None

Data:

The available occurrence records indicate that the harvest mouse is widespread throughout England with a few localised sightings observed along the Welsh coastline (Figure 1a). However, the maps highlights several patches where the species has not been reported for some time. The majority of sightings were reported in habitat dominated by arable, improved grassland and suburban land cover.

From the literature review we identified two studies (Kotzageorgis & Mason 1997; Tattersall et al. 2002) conducted in the south of England between 1990 and 1997 (Figure 1b). Estimates ranged between 2.45 and 3.49 per km^2 with the highest densities reported in arable dominated land cover (0.1 - 2.82 per km^2 accounting for uncertainty relating to unsurveyed areas within grid cells). Due to the limited coverage of these surveys estimates were unavailable for several dominant land covers where occurrence was reported (marked grey in Table 1) and where estimates were available the relative uncertainty within cells was large.

Model predictions:

The habitat suitability map (Figure 2a) appears to reflect the underlying data well with the set of "best" models predicting presence (and absence) to a mean AUC of 0.72. Overall, across 100 repetitions Random Forest proved to be the most commonly selected modelling approach displaying the highest AUC 25% of the time closely followed by Generalised Linear Models (24%). By land cover the mean habitat suitability scores suggest observation is most likely in landscapes dominated by calcareous grassland (Table 1) but, consistent with recorded sightings, the majority of occurrence is predicted in grid cells dominated by arable and improved grassland.

Due to the limited number of density estimates neither minimum nor maximum density estimates showed a correlation with habitat suitability. Both were best fitted using density as a fixed constant in cells where occurrence was predicted with accounting for spherical spatial autocorrelation.

Interestingly though, the predicted abundance range does not contain the 1995 estimate; instead suggesting a significant decrease in total population. In the absence of recent trend analysis the most likely explanation for this underestimation, given that the predicted distribution shows no evidence of a range contraction, is that the assumed density estimates are relatively low compared with the mean or perhaps more likely that the population was overestimated by Harris et al. (1995); the estimate is assigned the lowest reliability score (5 out of 5).

Reliability (Expert comment):

Whilst the overall range of harvest mice in Britain does not appear to have changed greatly over the last 20 years, the species appears to have become much more patchily distributed, and there are few recent records for central southern England and the south east. This merits further investigation to determine whether recording bias, genuine localised population declines or other factors are responsible. A notable addition to the known range of harvest mice in Britain is the Isle of Wight, where (suspected) residency appears to have been confirmed by a small number of records from 2012. However, the total abundance predicted here suggests a dramatic and worrying population decline in Britain of at least 80% relative to estimates published in 1995.

References:

Harris, S. J., P. Morris, S. Wray and D. Yalden (1995). A review of British mammals: population estimates and conservation status of British mammals other than cetaceans, Joint Nature Conservation Committee, Peterborough, UK.

Kotzageorgis, G. C. and C. F. Mason (1997). Small mammal populations in relation to hedgerow structure in an arable landscape. *Journal of Zoology* 242(3): 425-434.

Tattersall, F. H., D. W. Macdonald, B. J. Hart, P. Johnson, W. Manley and R. Feber (2002). Is habitat linearity important for small mammal communities on farmland? *Journal of Applied Ecology* 39(4): 643-652.

Table 1: Summary of observed data and model predictions by land cover class (LCM2007 target classification). Values shown in brackets denote the spatial coverage based on a 10km resolution raster map (number of grid cells). Years represent the median of records within each land class. Ranges for density and abundance are derived using the respective minimum and maximum raster maps (lower bound is mean of values across minimum raster map with upper across the maximum) which capture the spatial uncertainty generate by projecting irregular polygons describing survey sites onto a raster grid.

LCM2007 class	Observed					Predicted		
	Occurrence		Density					
	Records	Year	Estimates	Year	Range	Habitat suitability	Density	Abundance
1 (Broadleaved woodland)	16 (9)	1974	0 (0)	-	-	0.76 (9)	0.14 - 2.97	123.6 - 2,673
2 (Coniferous woodland)	2 (2)	1986	0 (0)	-	-	0.13 (2)	0.14 - 2.97	27.47 - 594
3 (Arable and Horticultural)	3,009 (585)	1987	5 (3)	1996	0.1 - 2.82	0.78 (768)	0.13 - 2.78	9,871 - 213,474
4 (Improved grassland)	564 (195)	1979	2 (1)	1996	0.01 - 2.45	0.49 (210)	0.13 - 2.77	2,690 - 58,171
5 (Rough grassland)	14 (2)	1993	0 (0)	-	-	0.16 (1)	0.14 - 2.97	13.73 - 297
6 (Neutral grassland)	0 (0)	-	0 (0)	-	-	0.02 (0)	-	0
7 (Calcareous grassland)	22 (2)	1993	0 (0)	-	-	0.9 (2)	0.14 - 2.97	27.47 - 594
8 (Acid grassland)	0 (0)	-	0 (0)	-	-	0.08 (0)	-	0
9 (Fen, Marsh, and Swamp)	0 (0)	-	0 (0)	-	-	-	-	0
10 (Heather)	0 (0)	-	0 (0)	-	-	0.09 (0)	-	0
11 (Heather grassland)	0 (0)	-	0 (0)	-	-	0.07 (0)	-	0
12 (Bog)	1 (1)	1966	0 (0)	-	-	0.09 (0)	-	0
13 (Montane habitat)	0 (0)	-	0 (0)	-	-	0.03 (0)	-	0
14 (Inland rock)	0 (0)	-	0 (0)	-	-	0.03 (0)	-	0
15 (Saltwater)	18 (1)	1974	0 (0)	-	-	0.49 (0)	-	0
16 (Freshwater)	0 (0)	-	0 (0)	-	-	0.07 (0)	-	0
17 (Supra-littoral rock)	0 (0)	-	0 (0)	-	-	0.05 (0)	-	0
18 (Supra-littoral sediment)	1 (1)	1965	0 (0)	-	-	0.31 (0)	-	0
19 (Littoral rock)	0 (0)	-	0 (0)	-	-	0.23 (0)	-	0
20 (Littoral sediment)	12 (5)	1975	0 (0)	-	-	0.48 (0)	-	0
21 (Saltmarsh)	0 (0)	-	0 (0)	-	-	-	-	0
22 (Urban)	25 (3)	2010	0 (0)	-	-	0.54 (0)	-	0
23 (Suburban)	91 (37)	1974	0 (0)	-	-	0.64 (14)	0.13 - 2.9	187.6 - 4,057
Total	3,775 (843)	1983	7 (4)	1996	0.08 - 2.73	0.47 (1,006)	0.13 - 2.78	12,941 - 279,861

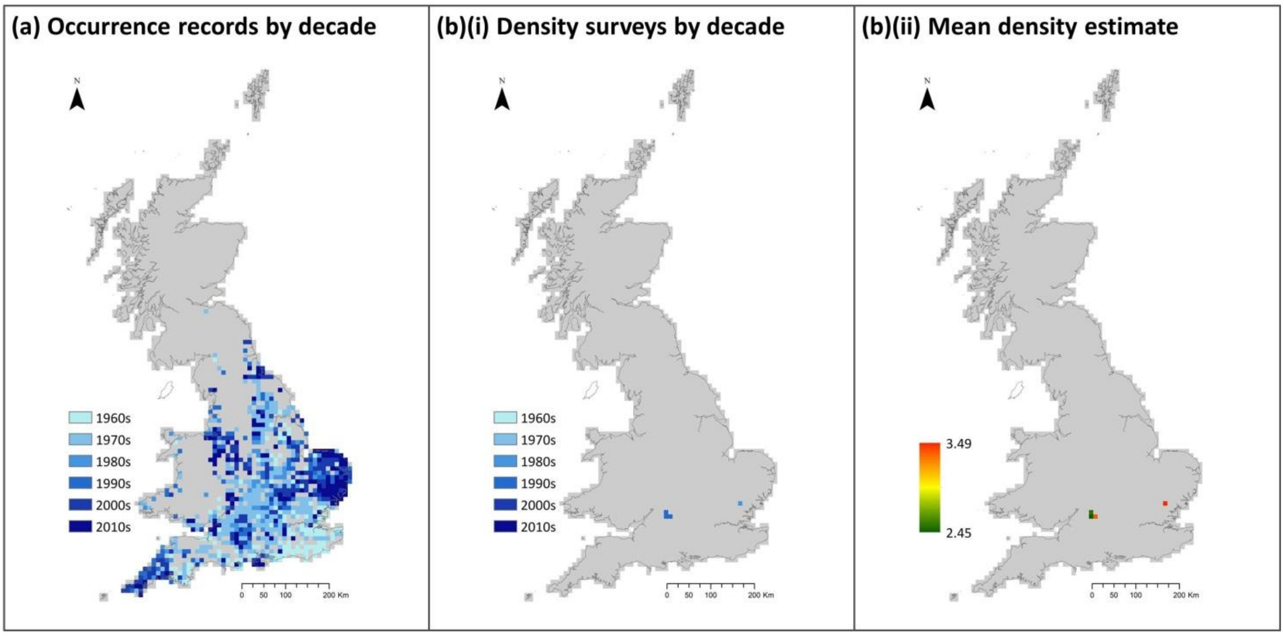

© Crown copyright and database rights 2016 Ordnance Survey 100051110. Data courtesy of the NBN Gateway with thanks to all data contributors. The NBN and its data contributors bear no responsibility for the further analysis or interpretation of this material, data and/or information.

Figure 1: 10km resolution raster maps based on BNG presenting the geographic description of available data. (a) shows the distribution of species occurrence obtained via the NBN Gateway categorised by the decade of last sighting. (b) shows information relating to density surveys identified via a search of published literature where: (i) categorises surveys by the decade of last survey; and (ii) shows the mean density estimate of surveys within grid cells (estimates assumed to be representative of entire cell, considered the upper limit of observed density).

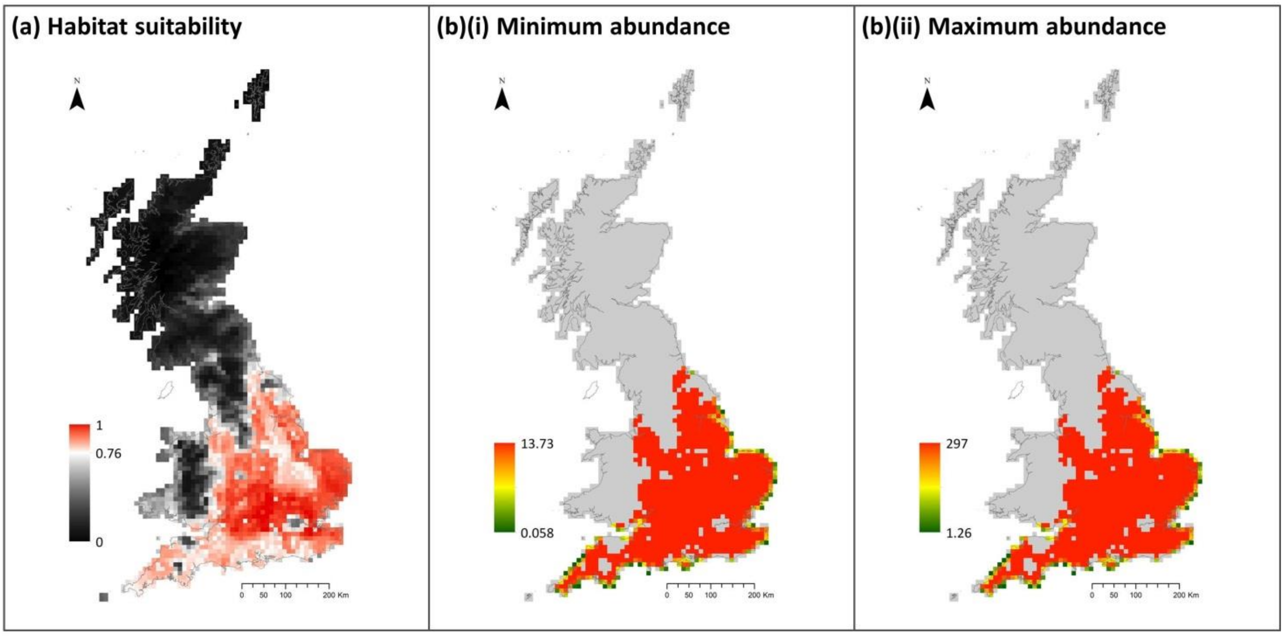

© Crown copyright and database rights 2016 Ordnance Survey 100051110. Data courtesy of the NBN Gateway with thanks to all data contributors. The NBN and its data contributors bear no responsibility for the further analysis or interpretation of this material, data and/or information.

Figure 2: Modelling predictions generated using systematic approach based on available data. (a) shows habitat suitability scores (the likelihood of observing the target species within each grid cell given variation environmental variables) determined by aggregating outputs from the "best" species distribution model (7 models compared) across 100 simulations. Here, the mid value on the scale denotes the threshold score above which occurrence is assumed. (b) shows: (i) the lower bound (Minimum); and (ii) the upper bound (Maximum); of abundance estimates determined by relating observed density (taking into account potential uncertainty) with habitat suitability scores using linear regression.
