# Supplementary material for: A systematic approach to estimate the distribution and total abundance of British mammals
Source: PLoS One. 2017 Jun 28;12(6):e0176339. doi: 10.1371/journal.pone.0176339 (PMC5489149; doi:10.1371/journal.pone.0176339)
Supplement: S9 File — Individual reports for each of the Rodentia species presenting analysis of the available data and subsequent model predictions based on a 10km raster grid. Reports also include expert comment assessing the reliability (and plausibility) of results in the context of existing evidence and popular opinion. (ZIP) [file pone.0176339.s009.zip › I House mouse.pdf]

House mouse (*Mus musculus*)

Order: *Rodentia*

Genus: *Mus*

Origin: Introduced

Status: Locally common

1995 abundance estimate: 5,192,000 (5)

Reported population trends: None

Data:

The available occurrence records indicate the house mouse is locally distributed throughout Britain with a few patches of widespread occurrence, most notably in East Anglia and Cornwall (Figure 1a). However, the map highlights several large areas, predominantly in Wales and Scotland, where the species is absent. Overall, sightings were reported in various habitats (predominantly arable and improved grassland) with the majority of grid cells where occurrence was observed containing at least one record since 1995.

From the literature review we identified two studies (Berry 1968; Tattersall et al. 2002); one reporting an estimate for a single cell dominated by rough grassland in Wales in 1967, and the other reporting an estimate across farmland (arable and improved grassland) in the south east of England between 1996 and 1997 (Figure 1b). Estimates ranged between 3.25 and 700 per km^2 with the highest density in the cell dominated by rough grassland (43.52 - 700 per km^2 accounting for uncertainty relating to unsurveyed areas within grid cells). Due to the limited coverage of these surveys estimates were unavailable for several dominant land covers where occurrence was reported (marked grey in Table 1) and where estimates were available the relative uncertainty within cells was large.

Model predictions:

The habitat suitability map (Figure 2a) appears to reflect the underlying data reasonably well with the set of "best" models predicting presence (and absence) to a mean AUC of 0.67. However, the resulting distribution covers an area approximately twice that of the observations alone (Figure 1a). Overall, across 100 repetitions MaxEnt proved to be the most commonly selected modelling approach displaying the highest AUC 32% of the time followed by Random Forest (24%). By land cover the mean habitat suitability scores suggest observation is most likely in landscapes dominated by calcareous grassland (despite only one observation) but, consistent with recorded sightings, the majority of occurrence is predicted in arable and improved grassland (the most common dominant land covers at a 10km scale).

Linear regression suggested that there was no correlation between the estimates of minimum density and habitat suitability; consequently, it was applied as a fixed constant in cells where occurrence is predicted accounting for spherical spatial autocorrelation (which was determined to provide the best fit model). Maximum density was found to be correlated with the best fit model relating habitat suitability linearly and accounting for Gaussian spatial autocorrelation. However, the relationship was determined to be negative with the highest densities applied to cells with a low suitability. This explains the inconsistency between the distributions of abundance as where predictions of density in cells of high suitability became negative they were removed (set to zero abundance). Most likely as a result of this the upper bound of the predicted range appear to be low and consequently the range does not contain the estimate from Harris et al. (1995); instead suggesting a substantial decrease in the total population. Whilst the is no evidence to contradict this trend a decrease of the magnitude suggested by the prediction seems unlikely. In order to provide more accurate predictions future model analysis could be based on a finer scale raster grid which would better represent the variations in habitat for smaller mammals. Unfortunately, at present this is too unreliable due to access restrictions imposed on occurrence data.

Reliability (Expert comment):

House mice are almost entirely commensal in Britain, and they are rarely encountered outside buildings; it is unlikely that house mouse populations in Britain would be near the upper limits for the range of densities suggested for broadleaved woodland, calcareous grassland and bog, although predicted abundance for these categories was low,

and hence they made little contribution to predicted total abundance. The large number of records for arable and horticultural habitats almost certainly reflects occupancy of agricultural buildings such as grain and animal feed stores. The most striking feature of the occurrence data is the complete absence of records for urban habitats, and the relatively large number of records for improved grassland. The latter may represent populations associated with livestock farms, while the absence of urban records clearly reflects sampling bias. Large-scale surveys have reported that house mice were present in 2-3% of domestic dwellings in England; assuming that the same pattern exists in Scotland and Wales, one house mouse in each of 2% of the 24,340,100 GB households in 2004 represents a population of 486,802, although the figure is likely to be higher than this as house mice are rarely solitary. The upper estimate of 500,536 house mice for GB is therefore probably too low; urban populations alone are likely to exceed this figure, even without the substantial populations associated with agricultural holdings.

References:

Berry, R. J. (1968). The ecology of an island population of the house mouse. *Journal of Animal Ecology* 37(2): 445-470.

Harris, S. J., P. Morris, S. Wray and D. Yalden (1995). A review of British mammals: population estimates and conservation status of British mammals other than cetaceans, Joint Nature Conservation Committee, Peterborough, UK.

Tattersall, F. H., D. W. Macdonald, B. J. Hart, P. Johnson, W. Manley and R. Feber (2002). Is habitat linearity important for small mammal communities on farmland? *Journal of Applied Ecology* 39(4): 643-652.

Table 1: Summary of observed data and model predictions by land cover class (LCM2007 target classification). Values shown in brackets denote the spatial coverage based on a 10km resolution raster map (number of grid cells). Years represent the median of records within each land class. Ranges for density and abundance are derived using the respective minimum and maximum raster maps (lower bound is mean of values across minimum raster map with upper across the maximum) which capture the spatial uncertainty generate by projecting irregular polygons describing survey sites onto a raster grid.

LCM2007 class	Observed					Predicted		
	Occurrence		Density					
	Records	Year	Estimates	Year	Range	Habitat suitability	Density	Abundance
1 (Broadleaved woodland)	2 (1)	2003	0 (0)	-	-	0.4 (1)	0.02 - 16.15	2.05 - 1,615
2 (Coniferous woodland)	17 (8)	2004	0 (0)	-	-	0.27 (6)	0.02 - 9.95	12.15 - 5,969
3 (Arable and Horticultural)	1,760 (294)	2004	4 (2)	1997	0.02 - 3.54	0.65 (533)	0.02 - 5.4	1,007 - 288,057
4 (Improved grassland)	520 (96)	2006	2 (1)	1996	0.02 - 4.71	0.43 (152)	0.02 - 9.66	299.1 - 146,820
5 (Rough grassland)	2 (1)	2001	1 (1)	1967	43.52 - 700	0.18 (2)	0.02 - 9.56	4.1 - 1,912
6 (Neutral grassland)	0 (0)	-	0 (0)	-	-	0.08 (0)	-	0
7 (Calcareous grassland)	1 (1)	1968	0 (0)	-	-	0.67 (2)	0.02 - 12.33	4.1 - 2,466
8 (Acid grassland)	16 (13)	2004	0 (0)	-	-	0.24 (9)	0.02 - 9.05	18.45 - 8,144
9 (Fen, Marsh, and Swamp)	0 (0)	-	0 (0)	-	-	-	-	0
10 (Heather)	5 (1)	2005	0 (0)	-	-	0.23 (1)	0 - 0.02	0.01 - 2.03
11 (Heather grassland)	8 (6)	2004	0 (0)	-	-	0.2 (2)	0.01 - 5.99	2.21 - 1,199
12 (Bog)	10 (7)	1997	0 (0)	-	-	0.19 (2)	0.02 - 14.08	4.1 - 2,816
13 (Montane habitat)	2 (1)	1960	0 (0)	-	-	0.16 (0)	-	0
14 (Inland rock)	0 (0)	-	0 (0)	-	-	0.12 (0)	-	0
15 (Saltwater)	1 (1)	2004	0 (0)	-	-	0.46 (2)	0 - 2.72	0.92 - 545
16 (Freshwater)	0 (0)	-	0 (0)	-	-	0.17 (0)	-	0
17 (Supra - littoral rock)	0 (0)	-	0 (0)	-	-	0.1 (0)	-	0
18 (Supra - littoral sediment)	0 (0)	-	0 (0)	-	-	0.24 (0)	-	0
19 (Littoral rock)	2 (1)	1962	0 (0)	-	-	0.31 (2)	0 - 0.03	0.07 - 5.07
20 (Littoral sediment)	30 (10)	1996	0 (0)	-	-	0.61 (20)	0.01 - 4.78	23.23 - 9,556
21 (Saltmarsh)	0 (0)	-	0 (0)	-	-	-	-	0
22 (Urban)	0 (0)	-	0 (0)	-	-	0.47 (0)	-	0
23 (Suburban)	157 (20)	2010	0 (0)	-	-	0.58 (37)	0.02 - 8.49	71.2 - 31,431
Total	2,533 (461)	2004	7 (4)	1996	10.89 - 178	0.44 (771)	0.02 - 6.49	1,449 - 500,536

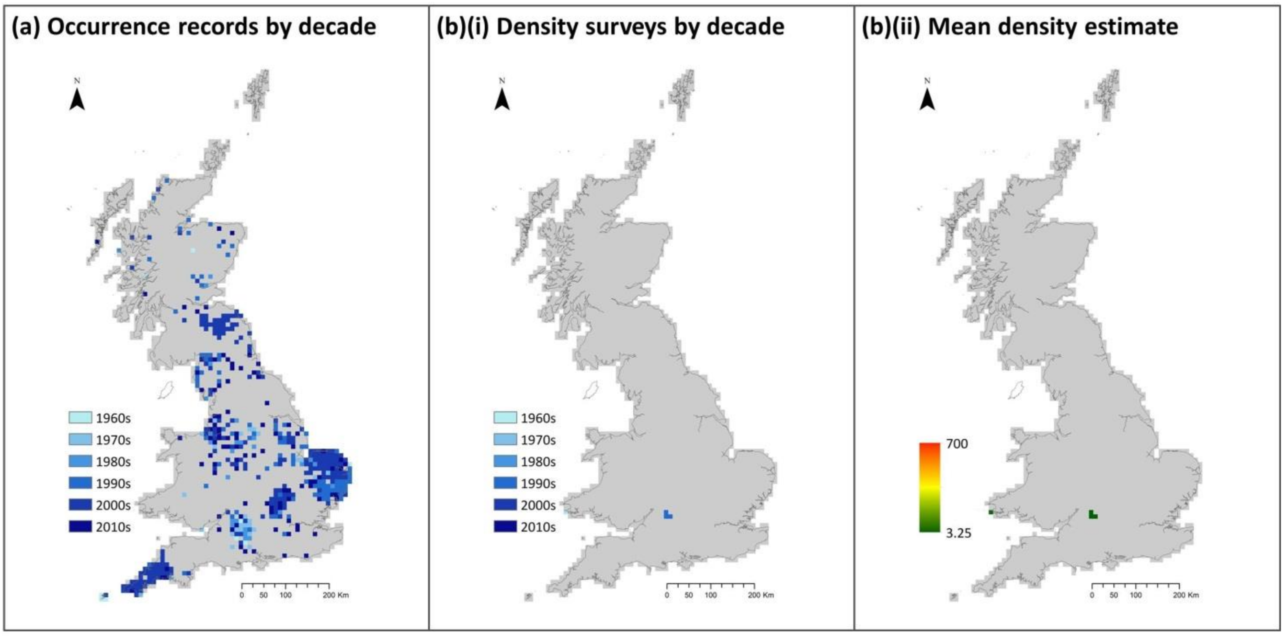

© Crown copyright and database rights 2016 Ordnance Survey 100051110. Data courtesy of the NBN Gateway with thanks to all data contributors. The NBN and its data contributors bear no responsibility for the further analysis or interpretation of this material, data and/or information.

Figure 1: 10km resolution raster maps based on BNG presenting the geographic description of available data. (a) shows the distribution of species occurrence obtained via the NBN Gateway categorised by the decade of last sighting. (b) shows information relating to density surveys identified via a search of published literature where: (i) categorises surveys by the decade of last survey; and (ii) shows the mean density estimate of surveys within grid cells (estimates assumed to be representative of entire cell, considered the upper limit of observed density).

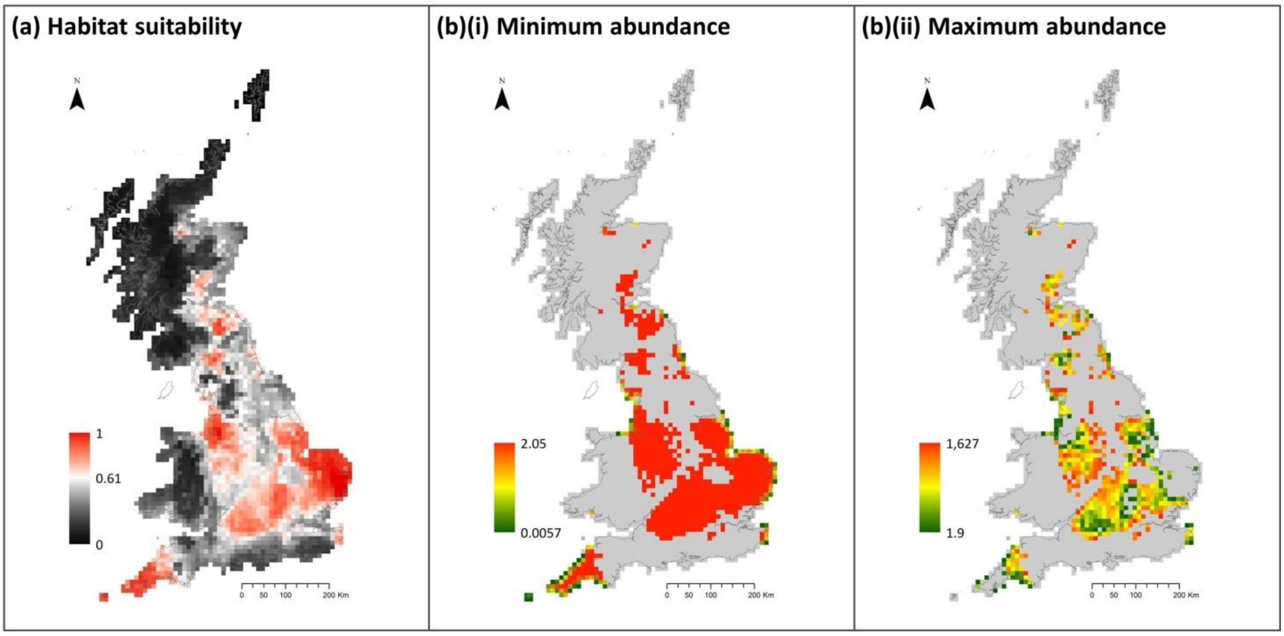

© Crown copyright and database rights 2016 Ordnance Survey 100051110. Data courtesy of the NBN Gateway with thanks to all data contributors. The NBN and its data contributors bear no responsibility for the further analysis or interpretation of this material, data and/or information.

Figure 2: Modelling predictions generated using systematic approach based on available data. (a) shows habitat suitability scores (the likelihood of observing the target species within each grid cell given variation environmental variables) determined by aggregating outputs from the "best" species distribution model (7 models compared) across 100 simulations. Here, the mid value on the scale denotes the threshold score above which occurrence is assumed. (b) shows: (i) the lower bound (Minimum); and (ii) the upper bound (Maximum); of abundance estimates determined by relating observed density (taking into account potential uncertainty) with habitat suitability scores using linear regression.
