# Supplementary material for: A systematic approach to estimate the distribution and total abundance of British mammals
Source: PLoS One. 2017 Jun 28;12(6):e0176339. doi: 10.1371/journal.pone.0176339 (PMC5489149; doi:10.1371/journal.pone.0176339)
Supplement: S9 File — Individual reports for each of the Rodentia species presenting analysis of the available data and subsequent model predictions based on a 10km raster grid. Reports also include expert comment assessing the reliability (and plausibility) of results in the context of existing evidence and popular opinion. (ZIP) [file pone.0176339.s009.zip › J Orkney vole.pdf]

Orkney vole (*Microtus arvalis orcadensis*)

Order: *Rodentia*

Genus: *Microtus*

Origin: Introduced

Status: Locally common

1995 abundance estimate: 1,000,000 (1)

Reported population trends: None

Data:

The available occurrence records indicate that the distribution of Orkney vole is limited to a single cell of improved grassland located on the Orkney Isles with the latest sighting in 2002 (Figure 1a).

From the literature review we identified a single survey (Gorman & Reynolds 1993) conducted in 1990 which estimated density in various habitats (improved, rough and heather grassland; bog) on Orkney reporting values between 9,890 (in bog) and 11,349 (in rough grassland) per km^2 (Figure 1b).

Model predictions:

Unfortunately, there was insufficient data available at a 10km scale to perform any model analysis.

Reliability (Expert comment):

Observed occurrence suggests a possible reduction in range; Arnold (1993) reported records from the Orkney islands of South Ronaldsay, Mainland, Rousay, Westray, Stronsay, Sanday, while here records are reported for Orkney Mainland only. However, it is likely that the species persists more widely in Britain despite the absence of recording on the NBN; Cucchi et al. (2014) reported analysis of 131 modern *Microtus arvalis orcadensis* samples from seven Orkney islands, as well as modern samples from Guernsey, although it is not clear how recently these were collected.

References:

Arnold, H. R. (1993). Atlas of mammals in Britain: HMSO.

Cucchi, T., R. Barnett, N. Martínková, S. Renaud, E. Renvoisé, A. Evin, A. Sheridan, I. Mainland, C. Wickham-Jones and C. Tougard (2014). The changing pace of insular life: 5000 years of microevolution in the Orkney vole (*Microtus arvalis orcadensis*). *Evolution* 68: 2804-2820.

Gorman, M. L. and P. Reynolds (1993). The impact of land-use change on voles and raptors. *Mammal Review* 23(3-4): 121-126.

Table 1: Summary of observed data and model predictions by land cover class (LCM2007 target classification). Values shown in brackets denote the spatial coverage based on a 10km resolution raster map (number of grid cells). Years represent the median of records within each land class. Ranges for density and abundance are derived using the respective minimum and maximum raster maps (lower bound is mean of values across minimum raster map with upper across the maximum) which capture the spatial uncertainty generate by projecting irregular polygons describing survey sites onto a raster grid.

LCM2007 class	Observed					Predicted		
	Occurrence		Density					
	Records	Year	Estimates	Year	Range	Habitat suitability	Density	Abundance
1 (Broadleaved woodland)	0 (0)	-	0 (0)	-	-	-	-	-
2 (Coniferous woodland)	0 (0)	-	0 (0)	-	-	-	-	-
3 (Arable and Horticultural)	0 (0)	-	0 (0)	-	-	-	-	-
4 (Improved grassland)	8 (1)	2002	10 (10)	1990	10,070	-	-	-
5 (Rough grassland)	0 (0)	-	2 (2)	1990	11,349	-	-	-
6 (Neutral grassland)	0 (0)	-	0 (0)	-	-	-	-	-
7 (Calcareous grassland)	0 (0)	-	0 (0)	-	-	-	-	-
8 (Acid grassland)	0 (0)	-	0 (0)	-	-	-	-	-
9 (Fen, Marsh, and Swamp)	0 (0)	-	0 (0)	-	-	-	-	-
10 (Heather)	0 (0)	-	0 (0)	-	-	-	-	-
11 (Heather grassland)	0 (0)	-	1 (1)	1990	11,119	-	-	-
12 (Bog)	0 (0)	-	1 (1)	1990	9,890	-	-	-
13 (Montane habitat)	0 (0)	-	0 (0)	-	-	-	-	-
14 (Inland rock)	0 (0)	-	0 (0)	-	-	-	-	-
15 (Saltwater)	0 (0)	-	0 (0)	-	-	-	-	-
16 (Freshwater)	0 (0)	-	0 (0)	-	-	-	-	-
17 (Supra-littoral rock)	0 (0)	-	0 (0)	-	-	-	-	-
18 (Supra-littoral sediment)	0 (0)	-	0 (0)	-	-	-	-	-
19 (Littoral rock)	0 (0)	-	0 (0)	-	-	-	-	-
20 (Littoral sediment)	0 (0)	-	0 (0)	-	-	-	-	-
21 (Saltmarsh)	0 (0)	-	0 (0)	-	-	-	-	-
22 (Urban)	0 (0)	-	0 (0)	-	-	-	-	-
23 (Suburban)	0 (0)	-	0 (0)	-	-	-	-	-
Total	8 (1)	2002	14 (14)	1990	10,315	-	-	-

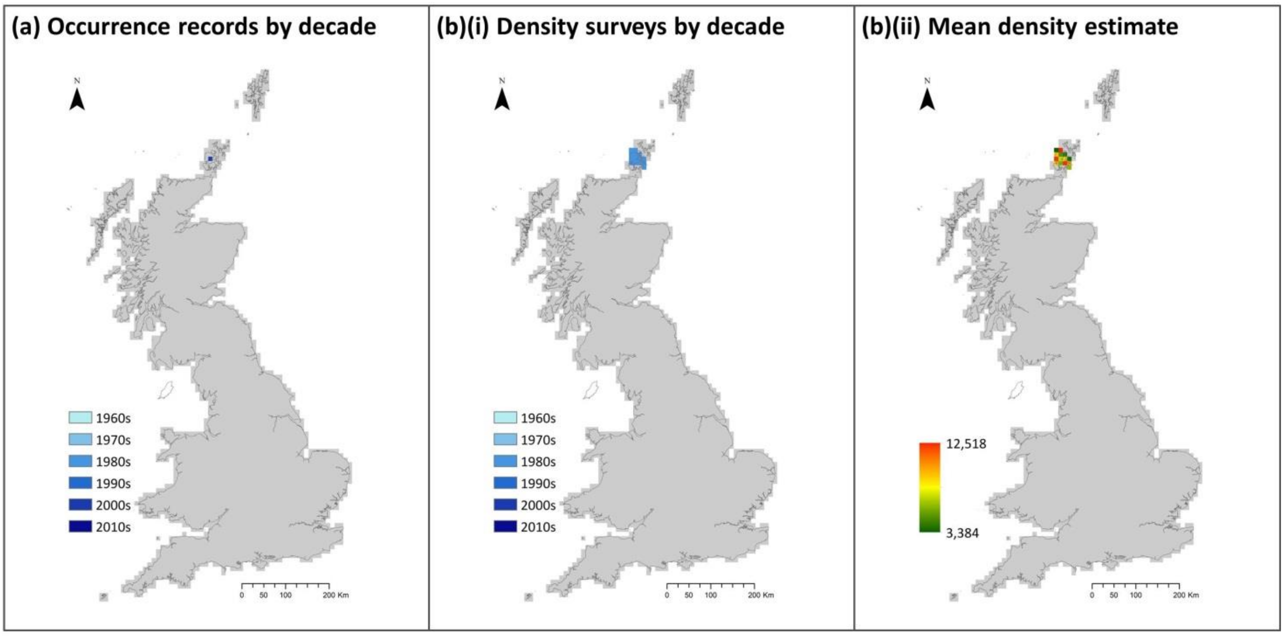

© Crown copyright and database rights 2016 Ordnance Survey 100051110. Data courtesy of the NBN Gateway with thanks to all data contributors. The NBN and its data contributors bear no responsibility for the further analysis or interpretation of this material, data and/or information.

Figure 1: 10km resolution raster maps based on BNG presenting the geographic description of available data. (a) shows the distribution of species occurrence obtained via the NBN Gateway categorised by the decade of last sighting. (b) shows information relating to density surveys identified via a search of published literature where: (i) categorises surveys by the decade of last survey; and (ii) shows the mean density estimate of surveys within grid cells (estimates assumed to be representative of entire cell, considered the upper limit of observed density).

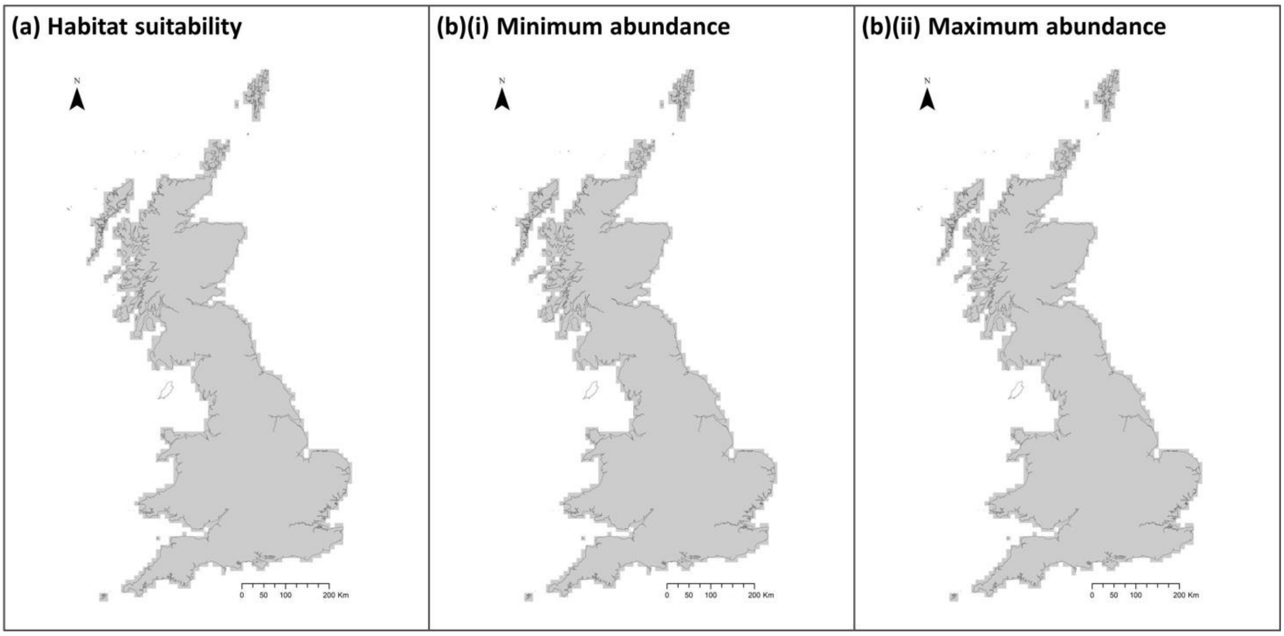

© Crown copyright and database rights 2016 Ordnance Survey 100051110. Data courtesy of the NBN Gateway with thanks to all data contributors. The NBN and its data contributors bear no responsibility for the further analysis or interpretation of this material, data and/or information.

Figure 2: Modelling predictions generated using systematic approach based on available data. (a) shows habitat suitability scores (the likelihood of observing the target species within each grid cell given variation environmental variables) determined by aggregating outputs from the "best" species distribution model (7 models compared) across 100 simulations. Here, the mid value on the scale denotes the threshold score above which occurrence is assumed. (b) shows: (i) the lower bound (Minimum); and (ii) the upper bound (Maximum); of abundance estimates determined by relating observed density (taking into account potential uncertainty) with habitat suitability scores using linear regression.
