# Supplementary material for: A systematic approach to estimate the distribution and total abundance of British mammals
Source: PLoS One. 2017 Jun 28;12(6):e0176339. doi: 10.1371/journal.pone.0176339 (PMC5489149; doi:10.1371/journal.pone.0176339)
Supplement: S9 File — Individual reports for each of the Rodentia species presenting analysis of the available data and subsequent model predictions based on a 10km raster grid. Reports also include expert comment assessing the reliability (and plausibility) of results in the context of existing evidence and popular opinion. (ZIP) [file pone.0176339.s009.zip › K Red squirrel.pdf]

Red squirrel (*Sciurus vulgaris*)

Order: *Rodentia*

Genus: *Sciurus*

Origin: Native

Status: Locally common

1995 abundance estimate: 160,000 (3)

Reported population trends: None

Data:

The available occurrence records indicate that the red squirrel is most widespread in Scotland and northern part of England with an isolated community on the Isle of Wight (Figure 1a). Whilst the map also highlights occurrence in other areas throughout GB, primarily Wales and East Anglia, the species has not been recorded for some time.

From the literature review we identified several studies (Bryce et al. 2002; Gurnell et al. 2004; Halliwell 1997; Holm 1990; Kenward et al. 1998; Shuttleworth 1996; Wauters et al. 2000) primarily conducted in areas of high density within the observed distribution between 1987 and 2000 (Figure 1b). Estimates ranged between 3.2 and 230 per km^2 with the highest densities reported on the Isle of Wight in habitat dominated by littoral sediment (4.12 - 230 per km^2 accounting for uncertainty relating to unsurveyed areas within grid cells). Due to the limited coverage of these surveys estimates were unavailable for several dominant land covers where occurrence was reported (marked grey in Table 1) and where estimates were available the relative uncertainty within cells was large.

Model predictions:

The habitat suitability map (Figure 2a) appears to reflect the underlying data reasonably well with the set of "best" models predicting presence (and absence) to a mean AUC of 0.73. However, the population on the Isle of Wight is not captured. Overall, across 100 repetitions MaxEnt proved to be the most commonly selected modelling approach displaying the highest AUC 46% of the time followed by Support Vector Machines (24%). By land cover the mean habitat suitability scores suggest observation is most likely in landscapes dominated by coniferous woodland (Table 1) but, consistent with recorded sightings, the majority of occurrence is predicted in arable and improved grassland (the most common dominant land covers at a 10km scale).

Both minimum and maximum density estimates were found to be correlated with habitat suitability, best fitted linearly and to the square applying spherical spatial autocorrelation respectively. However, both relationships suggested a negative association predicting the highest densities in cells of lower suitability.

The predicted abundance range does not contain the estimate from Harris et al. (1995) and instead suggests a significant increase in total population. Whilst the negative correlation between density and habitat suitability may explain this overestimation it is perhaps more likely that the use of high density estimates and the limited spatial extent of these surveys is responsible. In order to provide more accurate predictions future model analysis could be based on a finer scale raster grid which would better represent the variations in habitat for smaller mammals. Unfortunately, at present this is too unreliable due to access restrictions imposed on occurrence data.

Reliability (Expert comment):

According to occurrence records, the range of the red squirrel is continuing to contract in most areas of England and Wales; distribution in northern Britain appears to have changed very little over the last 20 years and red squirrel conservation groups in northern England have indicated that populations remained stable during 2012 - 2014, despite advances in grey squirrel range into this region. Additionally, compared to data reported by Arnold (1993), occurrence of red (and grey) squirrels has increased in southern Scotland. Red squirrels continue to do well in the southern Highlands, into which grey squirrels appear to have made little, if any, advance from central Scotland over the last 20 years. The lower bound for the total abundance estimate reported here is nearly twice the population estimate reported by Harris et al. (1995), which also gives some cause for optimism, although the method for deriving these two estimates differs considerably hence comparisons must be made with caution.

References:

Arnold, H. R. (1993). Atlas of mammals in Britain: HMSO.

Bryce, J., P. J. Johnson and D. W. Macdonald (2002). Can niche use in red and grey squirrels offer clues for their apparent coexistence*? Journal of Applied Ecology* 39(6): 875-887.

Gurnell, J., L. A. Wauters, P. W. W. Lurz and G. Tosi (2004). Alien species and interspecific competition: effects of introduced eastern grey squirrels on red squirrel population dynamics. *Journal of Animal Ecology* 73(1): 26-35.

Halliwell, E. C. (1997). The ecology of red squirrels in Scotland in relation to pine marten predation. Ph.D. Thesis, University of Aberdeen.

Harris, S. J., P. Morris, S. Wray and D. Yalden (1995). A review of British mammals: population estimates and conservation status of British mammals other than cetaceans, Joint Nature Conservation Committee, Peterborough, UK.

Holm, J. L. (1990). The ecology of red squirrels (*Sciurus vulgaris*) in deciduous woodlands. Ph.D. Thesis, Royal Holloway, University of London.

Kenward, R. E., K. H. Hodder, R. J. Rose, C. A. Walls, T. Parish, J. L. Holm, P. A. Morris, S. S. Walls and F. I. Doyle (1998). Comparative demography of red squirrels (*Sciurus vulgaris*) and grey squirrels (*Sciurus carolinensis*) in deciduous and conifer woodland. *Journal of Zoology* 244(1): 7-21.

Shuttleworth, C. M. (1996). The effect of supplemental feeding on the red squirrel (*Sciurus vulgaris*). Ph.D. Thesis, University of London.

Wauters, L. A., P. W. W. Lurz and J. Gurnell (2000). Interspecific effects of grey squirrels (*Sciurus carolinensis*) on the space use and population demography of red squirrels (*Sciurus vulgaris*) in conifer plantations. *Ecological Research* 15(3): 271-284.

Table 1: Summary of observed data and model predictions by land cover class (LCM2007 target classification). Values shown in brackets denote the spatial coverage based on a 10km resolution raster map (number of grid cells). Years represent the median of records within each land class. Ranges for density and abundance are derived using the respective minimum and maximum raster maps (lower bound is mean of values across minimum raster map with upper across the maximum) which capture the spatial uncertainty generate by projecting irregular polygons describing survey sites onto a raster grid.

LCM2007 class	Observed					Predicted		
	Occurrence		Density					
	Records	Year	Estimates	Year	Range	Habitat suitability	Density	Abundance
1 (Broadleaved woodland)	639 (3)	2012	0 (0)	-	-	0.42 (2)	2.3 - 83	451 - 16,648
2 (Coniferous woodland)	13,419 (142)	2013	4 (3)	1998	1.6 - 64	0.88 (149)	1.5 - 77	21,972 - 1,141,378
3 (Arable and Horticultural)	35,846 (361)	1995	1 (1)	1987	0.1 - 86	0.58 (343)	2.6 - 91	90,203 - 3,102,351
4 (Improved grassland)	39,280 (370)	2010	13 (8)	1997	1.3 - 49	0.64 (402)	2.4 - 85	94,315 - 3,418,242
5 (Rough grassland)	373 (13)	2012	2 (1)	1998	0.2 - 26	0.3 (13)	2.3 - 76	3,015 - 98,898
6 (Neutral grassland)	0 (0)	-	0 (0)	-	-	0.03 (0)	-	-
7 (Calcareous grassland)	0 (0)	-	0 (0)	-	-	0.16 (0)	-	-
8 (Acid grassland)	8,373 (143)	2012	0 (0)	-	-	0.78 (176)	2.7 - 95	47,077 - 1,676,814
9 (Fen, Marsh, and Swamp)	0 (0)	-	0 (0)	-	-	-	-	-
10 (Heather)	1,254 (37)	2013	0 (0)	-	-	0.74 (44)	2.4 - 92	10,469 - 402,435
11 (Heather grassland)	1,466 (42)	2013	0 (0)	-	-	0.42 (39)	2.2 - 81	8,585 - 316,659
12 (Bog)	980 (29)	2006	0 (0)	-	-	0.32 (31)	2.9 - 98	9,051 - 303,657
13 (Montane habitat)	971 (43)	2012	0 (0)	-	-	0.85 (48)	2.1 - 86	10,194 - 413,667
14 (Inland rock)	0 (0)	-	0 (0)	-	-	0.09 (0)	-	-
15 (Saltwater)	373 (5)	2007	2 (1)	1995	7.4 - 220	0.62 (6)	1.1 - 42	667.9 - 25,200
16 (Freshwater)	71 (2)	2014	0 (0)	-	-	0.64 (2)	1.8 - 78	350.3 - 15,615
17 (Supra-littoral rock)	0 (0)	-	0 (0)	-	-	0.01 (0)	-	-
18 (Supra-littoral sediment)	0 (0)	-	0 (0)	-	-	0.29 (0)	-	-
19 (Littoral rock)	0 (0)	-	0 (0)	-	-	0.2 (0)	-	-
20 (Littoral sediment)	2,363 (17)	2013	2 (2)	1996	4.1 - 230	0.65 (20)	2 - 65	3,945 - 130,460
21 (Saltmarsh)	0 (0)	-	0 (0)	-	-	-	-	-
22 (Urban)	50 (1)	2012	0 (0)	-	-	0.51 (1)	0.5 - 15	50.81 - 1,536
23 (Suburban)	1,268 (26)	2006	0 (0)	-	-	0.55 (19)	2.5 - 91	4,727 - 173,580
Total	106,726 (1,234)	2011	24 (16)	1996	1.9 - 86	0.6 (1,295)	2.4 - 87	305,073 - 11,237,141

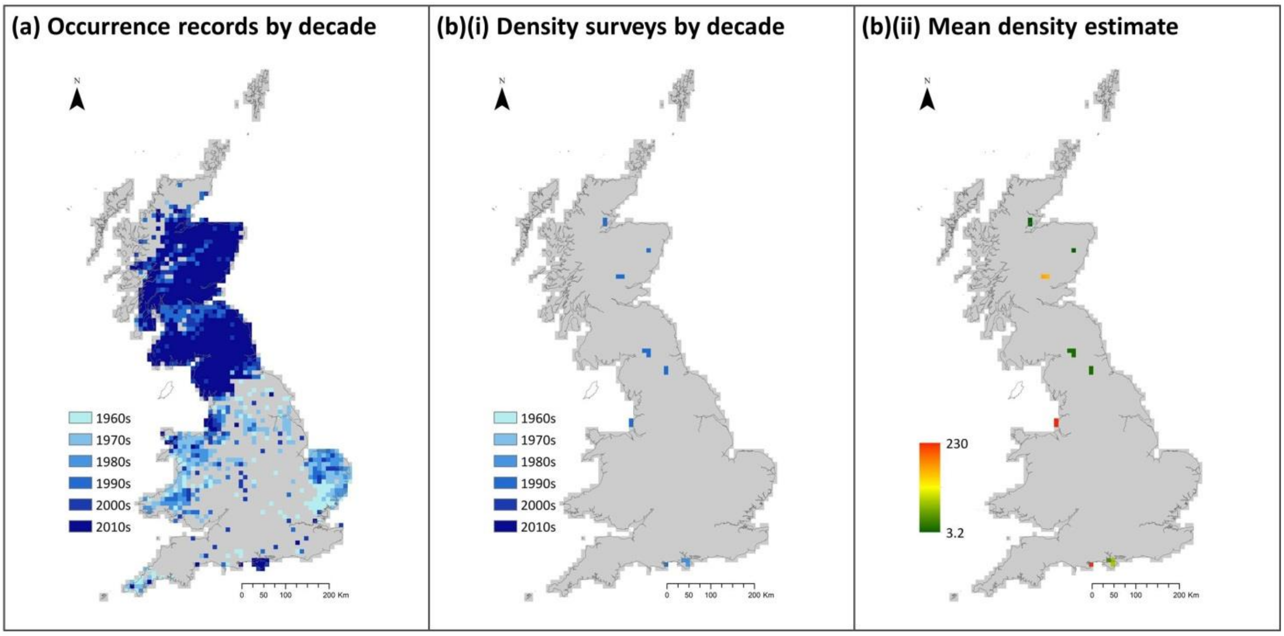

© Crown copyright and database rights 2016 Ordnance Survey 100051110. Data courtesy of the NBN Gateway with thanks to all data contributors. The NBN and its data contributors bear no responsibility for the further analysis or interpretation of this material, data and/or information.

Figure 1: 10km resolution raster maps based on BNG presenting the geographic description of available data. (a) shows the distribution of species occurrence obtained via the NBN Gateway categorised by the decade of last sighting. (b) shows information relating to density surveys identified via a search of published literature where: (i) categorises surveys by the decade of last survey; and (ii) shows the mean density estimate of surveys within grid cells (estimates assumed to be representative of entire cell, considered the upper limit of observed density).

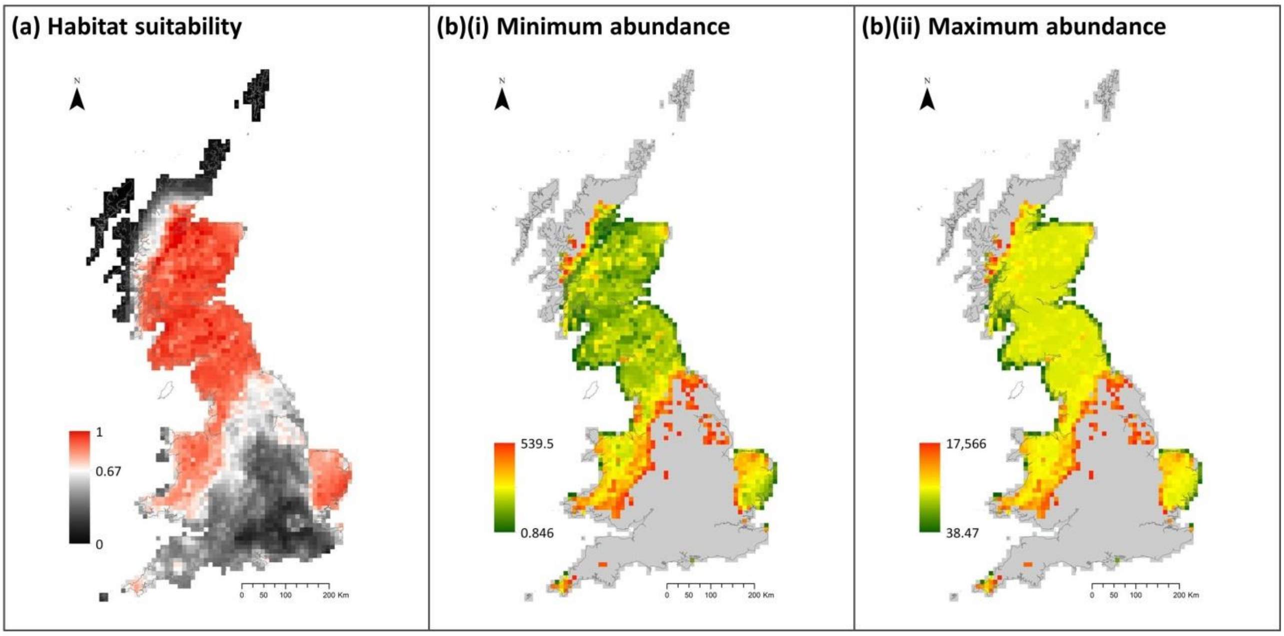

© Crown copyright and database rights 2016 Ordnance Survey 100051110. Data courtesy of the NBN Gateway with thanks to all data contributors. The NBN and its data contributors bear no responsibility for the further analysis or interpretation of this material, data and/or information.

Figure 2: Modelling predictions generated using systematic approach based on available data. (a) shows habitat suitability scores (the likelihood of observing the target species within each grid cell given variation environmental variables) determined by aggregating outputs from the "best" species distribution model (7 models compared) across 100 simulations. Here, the mid value on the scale denotes the threshold score above which occurrence is assumed. (b) shows: (i) the lower bound (Minimum); and (ii) the upper bound (Maximum); of abundance estimates determined by relating observed density (taking into account potential uncertainty) with habitat suitability scores using linear regression.
