# Supplementary material for: A systematic approach to estimate the distribution and total abundance of British mammals
Source: PLoS One. 2017 Jun 28;12(6):e0176339. doi: 10.1371/journal.pone.0176339 (PMC5489149; doi:10.1371/journal.pone.0176339)
Supplement: S9 File — Individual reports for each of the Rodentia species presenting analysis of the available data and subsequent model predictions based on a 10km raster grid. Reports also include expert comment assessing the reliability (and plausibility) of results in the context of existing evidence and popular opinion. (ZIP) [file pone.0176339.s009.zip › L Ship rat.pdf]

Ship rat (*Rattus rattus*)

Order: *Rodentia*

Genus: *Rattus*

Origin: Introduced

Status: Rare

1995 abundance estimate: 1,300 (2)

Reported population trends: None

Data:

The available occurrence records indicate that the ship rat is rare with scattered sightings reported throughout Britain (Figure 1a). However, the records in many cells are historic and have not been updated for some time.

From the literature review we identified a single high density estimate of 161 per km^2 for the littoral rock dominated habitat of the Shiant Islands (McDonald et al. 1997)), surveyed in 1996 (Figure 1b). Due to the limited coverage of this study estimates were not available in the majority of dominant land covers where occurrence has been recorded (land cover marked grey in Table 1). Unlike for most small mammals the density range assumed from the study accounting for uncertainty was narrow (122.9 - 161 per km^2).

Model predictions:

The habitat suitability map (Figure 2a) appears to reflect the underlying data reasonably well with the set of "best" models predicting presence (and absence) to a mean AUC of 0.79. However, the resulting distribution is substantially larger than the area described by the observations (approximately 5 times) which is perhaps unlikely. Overall, across 100 repetitions MaxEnt proved to be the most commonly selected modelling approach displaying the highest AUC 51% of the time followed by Generalised Linear Models (23%). By land cover the mean habitat suitability scores suggest observation is most likely in landscapes dominated by urban and suburban habitat (Table 1) but, consistent with recorded sightings, the majority of occurrence is predicted in grid cells dominated by arable land.

Due to the limited number of density estimates it was not possible to assess any relationship with habitat suitability. Instead, a constant mean estimate was applied to all cells where occurrence was predicted and summed to derive total abundance.

Perhaps unsurprisingly given the inflated distribution and high density estimates, the predicted abundance range does not contain the estimate from Harris et al. (1995); instead suggesting a significant increase in the total population. This overestimation highlights the difficulty of modelling very rare species at such broad scales. Predictions may be made more plausible using models based on finer resolution raster data where the habitats of small mammal such as this species can be better represented.

Reliability (Expert comment):

Once much more common in Britain, historical accounts indicate that ship rat populations declined following the introduction of common rats in the early 18th century. The low number of recorded observations is therefore unsurprising and their distribution suggests an association with coastal areas and a possible link with commercial shipping activities. Observations indicate that several isolated offshore populations remain, although some of these (e.g. Lundy in the Bristol channel) have since been removed through seabird conservation projects. The median year of recorded observations for all land classes is more than 20 years ago, which suggests observations (and potentially populations) are continuing to decline, and the range of estimates presented here may be too high. The habitat suitability maps should be interpreted with caution; given that ship rats were once widespread in Britain, the overriding factor that currently characterises habitat suitability is probably the presence or absence of common rats.

References:

Harris, S. J., P. Morris, S. Wray and D. Yalden (1995). A review of British mammals: population estimates and conservation status of British mammals other than cetaceans, Joint Nature Conservation Committee, Peterborough, UK.

McDonald, R. A., M. R. Hutchings and J. G. M. Keeling (1997). The status of ship rats *Rattus rattus* on the Shiant Islands, Outer Hebrides, Scotland. *Biological Conservation* 82(1): 113-117.

Table 1: Summary of observed data and model predictions by land cover class (LCM2007 target classification). Values shown in brackets denote the spatial coverage based on a 10km resolution raster map (number of grid cells). Years represent the median of records within each land class. Ranges for density and abundance are derived using the respective minimum and maximum raster maps (lower bound is mean of values across minimum raster map with upper across the maximum) which capture the spatial uncertainty generate by projecting irregular polygons describing survey sites onto a raster grid.

LCM2007 class	Observed					Predicted		
	Occurrence		Density					
	Records	Year	Estimates	Year	Range	Habitat suitability	Density	Abundance
1 (Broadleaved woodland)	0 (0)	-	0 (0)	-	-	0.15 (1)	122.9 - 162	12,288 - 16,197
2 (Coniferous woodland)	0 (0)	-	0 (0)	-	-	0.1 (0)	-	-
3 (Arable and Horticultural)	58 (32)	1985	0 (0)	-	-	0.23 (201)	104.1 - 137.2	2,092,608 - 2,758,179
4 (Improved grassland)	27 (22)	1972	0 (0)	-	-	0.2 (147)	96.3 - 126.9	1,415,653 - 1,865,913
5 (Rough grassland)	9 (2)	1978	0 (0)	-	-	0.2 (9)	28.64 - 37.75	25,778 - 33,976
6 (Neutral grassland)	0 (0)	-	0 (0)	-	-	0.22 (0)	-	-
7 (Calcareous grassland)	0 (0)	-	0 (0)	-	-	0.09 (0)	-	-
8 (Acid grassland)	0 (0)	-	0 (0)	-	-	0.07 (0)	-	-
9 (Fen, Marsh, and Swamp)	0 (0)	-	0 (0)	-	-	-	-	-
10 (Heather)	0 (0)	-	0 (0)	-	-	0.07 (0)	-	-
11 (Heather grassland)	1 (1)	1994	0 (0)	-	-	0.12 (2)	54.54 - 71.89	10,909 - 14,378
12 (Bog)	1 (1)	1961	0 (0)	-	-	0.12 (3)	67.94 - 89.55	20,382 - 26,865
13 (Montane habitat)	0 (0)	-	0 (0)	-	-	0.03 (0)	-	-
14 (Inland rock)	0 (0)	-	0 (0)	-	-	0.08 (0)	-	-
15 (Saltwater)	0 (0)	-	0 (0)	-	-	0.3 (6)	38.48 - 50.72	23,087 - 30,430
16 (Freshwater)	0 (0)	-	0 (0)	-	-	0.24 (1)	120.8 - 159.2	12,076 - 15,917
17 (Supra - littoral rock)	0 (0)	-	0 (0)	-	-	0.13 (0)	-	-
18 (Supra - littoral sediment)	0 (0)	-	0 (0)	-	-	0.16 (0)	-	-
19 (Littoral rock)	1 (1)	1973	1 (1)	1996	123 - 162	0.33 (5)	1.56 - 2.05	777.9 - 1,025
20 (Littoral sediment)	8 (3)	1971	0 (0)	-	-	0.33 (14)	48.84 - 64.38	68,382 - 90,131
21 (Saltmarsh)	0 (0)	-	0 (0)	-	-	-	-	-
22 (Urban)	15 (3)	1979	0 (0)	-	-	0.74 (8)	83.01 - 109.4	66,405 - 87,526
23 (Suburban)	65 (21)	1979	0 (0)	-	-	0.64 (78)	101.5 - 133.8	791,824 - 1,043,670
Total	185 (86)	1979	1 (1)	1996	123 - 162	0.2 (475)	95.58 - 126	4,540,169 - 5,984,207

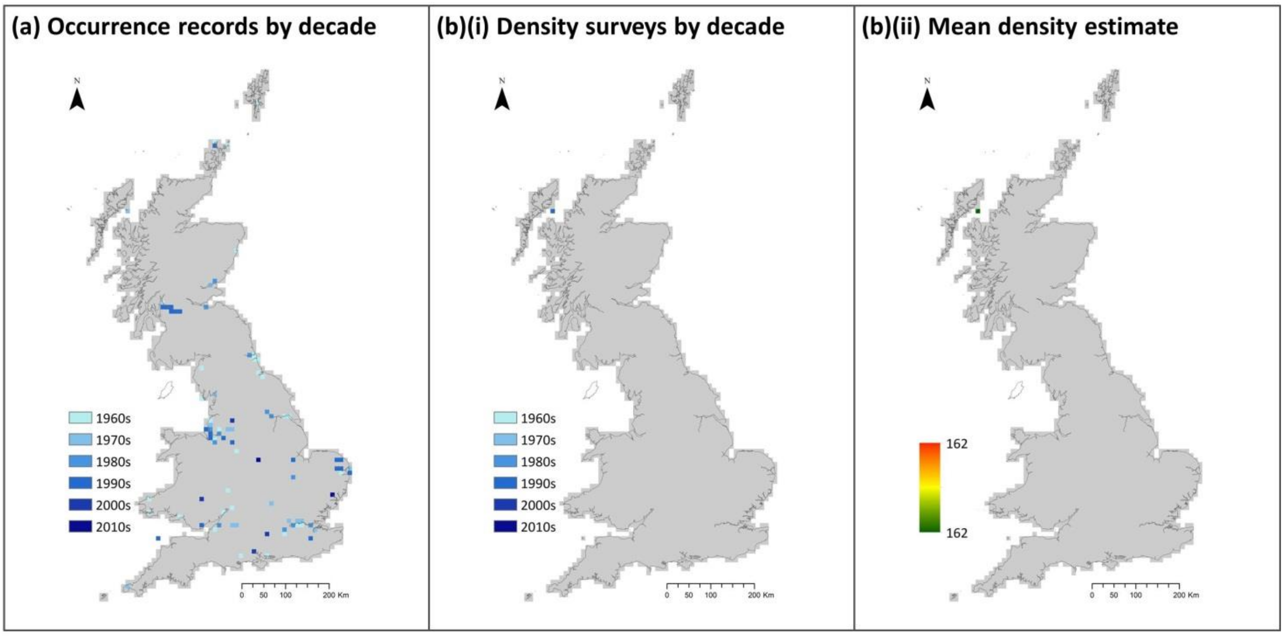

© Crown copyright and database rights 2016 Ordnance Survey 100051110. Data courtesy of the NBN Gateway with thanks to all data contributors. The NBN and its data contributors bear no responsibility for the further analysis or interpretation of this material, data and/or information.

Figure 1: 10km resolution raster maps based on BNG presenting the geographic description of available data. (a) shows the distribution of species occurrence obtained via the NBN Gateway categorised by the decade of last sighting. (b) shows information relating to density surveys identified via a search of published literature where: (i) categorises surveys by the decade of last survey; and (ii) shows the mean density estimate of surveys within grid cells (estimates assumed to be representative of entire cell, considered the upper limit of observed density).

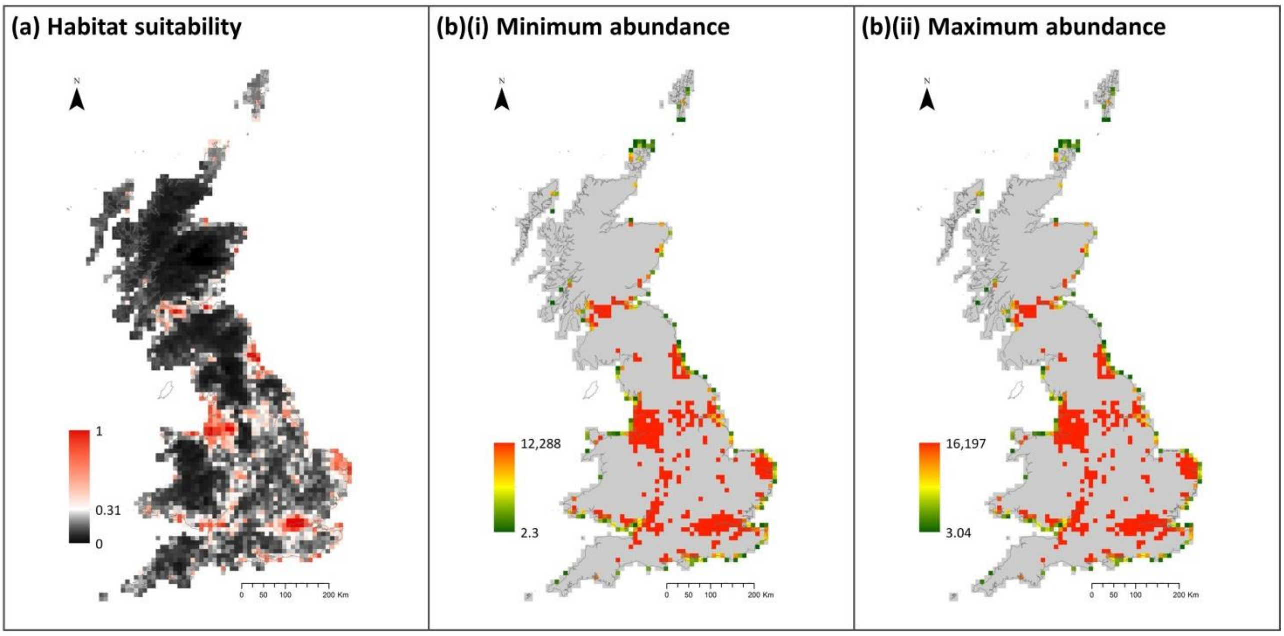

© Crown copyright and database rights 2016 Ordnance Survey 100051110. Data courtesy of the NBN Gateway with thanks to all data contributors. The NBN and its data contributors bear no responsibility for the further analysis or interpretation of this material, data and/or information.

Figure 2: Modelling predictions generated using systematic approach based on available data. (a) shows habitat suitability scores (the likelihood of observing the target species within each grid cell given variation environmental variables) determined by aggregating outputs from the "best" species distribution model (7 models compared) across 100 simulations. Here, the mid value on the scale denotes the threshold score above which occurrence is assumed. (b) shows: (i) the lower bound (Minimum); and (ii) the upper bound (Maximum); of abundance estimates determined by relating observed density (taking into account potential uncertainty) with habitat suitability scores using linear regression.
