# Supplementary material for: A systematic approach to estimate the distribution and total abundance of British mammals
Source: PLoS One. 2017 Jun 28;12(6):e0176339. doi: 10.1371/journal.pone.0176339 (PMC5489149; doi:10.1371/journal.pone.0176339)
Supplement: S9 File — Individual reports for each of the Rodentia species presenting analysis of the available data and subsequent model predictions based on a 10km raster grid. Reports also include expert comment assessing the reliability (and plausibility) of results in the context of existing evidence and popular opinion. (ZIP) [file pone.0176339.s009.zip › M Skomer vole.pdf]

Skomer vole (*Myodes glareolus skomerensis*)

Order: *Rodentia*

Genus: *Myodes*

Origin: Native

Status: Locally common

1995 abundance estimate: 7,000 (1)

Reported population trends: None

Data:

No occurrence records were available.

From the literature review we identified a single survey (Harris et al. 1995) conducted in 1992 which estimated density on Skomer Island to be approximately 2,414 per km^2 (Figure 1b); this did not specify any variation based on habitat or specific geographic locations.

Model predictions:

Unfortunately, there was insufficient data available at a 10km scale to perform any model analysis.

Reliability (Expert comment):

Loughran recently reported that despite declines since 2001, Skomer voles (which still share many similarities with ancestral mainland populations of bank voles) were still present in 2013. It is likely that populations continue to persist despite the lack of available records on the NBN.

References:

Harris, S. J., P. Morris, S. Wray and D. Yalden (1995). A review of British mammals: population estimates and conservation status of British mammals other than cetaceans, Joint Nature Conservation Committee, Peterborough, UK.

Loughran, M., Report on the work carried out on Skomer voles between 2001 - 2013. Unpublished report to The Wildlife Trust of South & West Wales.

Table 1: Summary of observed data and model predictions by land cover class (LCM2007 target classification). Values shown in brackets denote the spatial coverage based on a 10km resolution raster map (number of grid cells). Years represent the median of records within each land class. Ranges for density and abundance are derived using the respective minimum and maximum raster maps (lower bound is mean of values across minimum raster map with upper across the maximum) which capture the spatial uncertainty generate by projecting irregular polygons describing survey sites onto a raster grid.

LCM2007 class	Observed					Predicted		
	Occurrence		Density					
	Records	Year	Estimates	Year	Range	Habitat suitability	Density	Abundance
1 (Broadleaved woodland)	0 (0)	-	0 (0)	-	-	-	-	-
2 (Coniferous woodland)	0 (0)	-	0 (0)	-	-	-	-	-
3 (Arable and Horticultural)	0 (0)	-	0 (0)	-	-	-	-	-
4 (Improved grassland)	0 (0)	-	1 (1)	1992	2,414	-	-	-
5 (Rough grassland)	0 (0)	-	1 (1)	1992	2,414	-	-	-
6 (Neutral grassland)	0 (0)	-	0 (0)	-	-	-	-	-
7 (Calcareous grassland)	0 (0)	-	0 (0)	-	-	-	-	-
8 (Acid grassland)	0 (0)	-	0 (0)	-	-	-	-	-
9 (Fen, Marsh, and Swamp)	0 (0)	-	0 (0)	-	-	-	-	-
10 (Heather)	0 (0)	-	0 (0)	-	-	-	-	-
11 (Heather grassland)	0 (0)	-	0 (0)	-	-	-	-	-
12 (Bog)	0 (0)	-	0 (0)	-	-	-	-	-
13 (Montane habitat)	0 (0)	-	0 (0)	-	-	-	-	-
14 (Inland rock)	0 (0)	-	0 (0)	-	-	-	-	-
15 (Saltwater)	0 (0)	-	0 (0)	-	-	-	-	-
16 (Freshwater)	0 (0)	-	0 (0)	-	-	-	-	-
17 (Supra-littoral rock)	0 (0)	-	0 (0)	-	-	-	-	-
18 (Supra-littoral sediment)	0 (0)	-	0 (0)	-	-	-	-	-
19 (Littoral rock)	0 (0)	-	0 (0)	-	-	-	-	-
20 (Littoral sediment)	0 (0)	-	0 (0)	-	-	-	-	-
21 (Saltmarsh)	0 (0)	-	0 (0)	-	-	-	-	-
22 (Urban)	0 (0)	-	0 (0)	-	-	-	-	-
23 (Suburban)	0 (0)	-	0 (0)	-	-	-	-	-
Total	0 (0)	-	2 (2)	1992	2,414	-	-	-

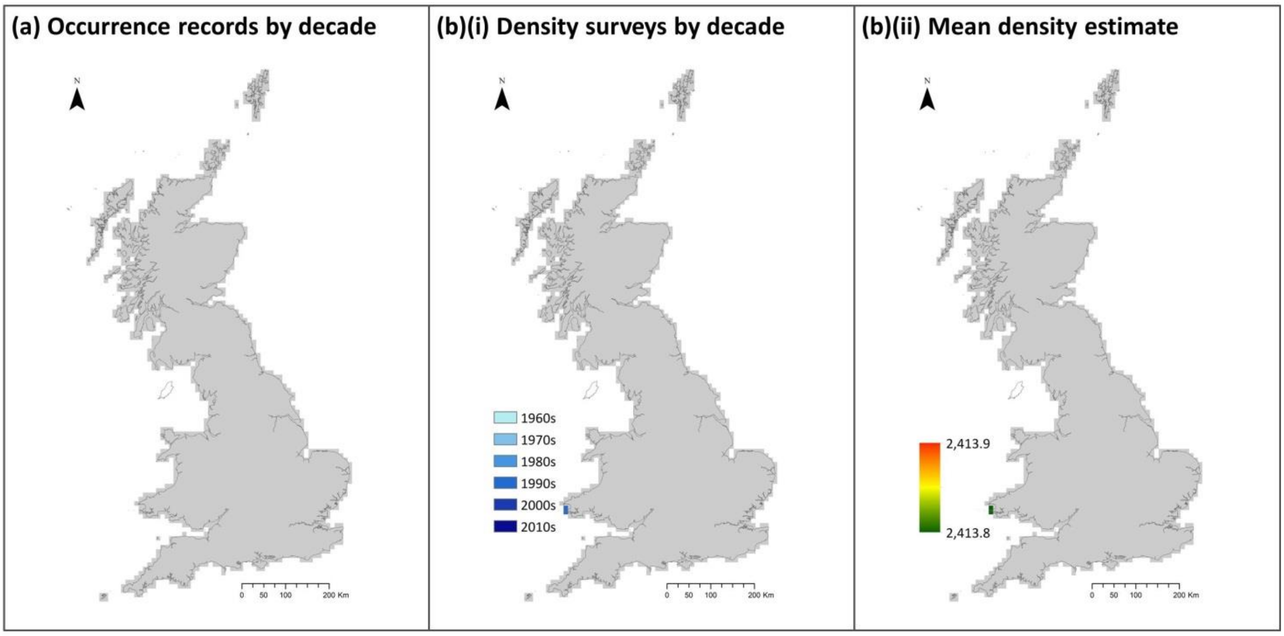

© Crown copyright and database rights 2016 Ordnance Survey 100051110. Data courtesy of the NBN Gateway with thanks to all data contributors. The NBN and its data contributors bear no responsibility for the further analysis or interpretation of this material, data and/or information.

Figure 1: 10km resolution raster maps based on BNG presenting the geographic description of available data. (a) shows the distribution of species occurrence obtained via the NBN Gateway categorised by the decade of last sighting. (b) shows information relating to density surveys identified via a search of published literature where: (i) categorises surveys by the decade of last survey; and (ii) shows the mean density estimate of surveys within grid cells (estimates assumed to be representative of entire cell, considered the upper limit of observed density).

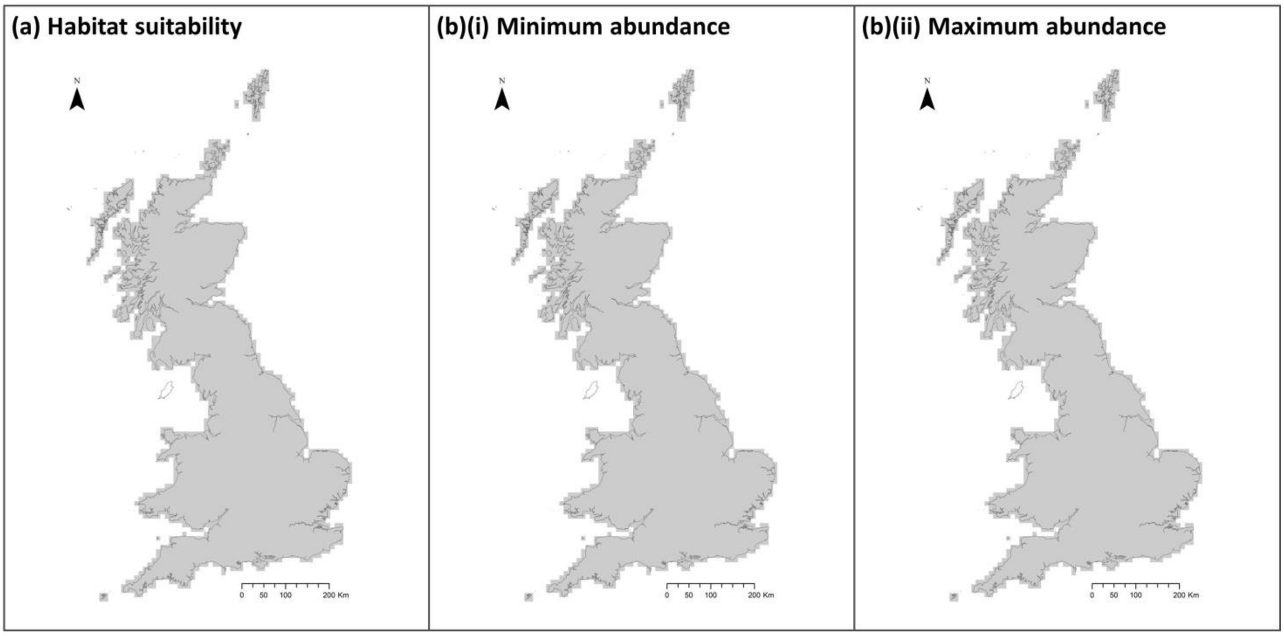

© Crown copyright and database rights 2016 Ordnance Survey 100051110. Data courtesy of the NBN Gateway with thanks to all data contributors. The NBN and its data contributors bear no responsibility for the further analysis or interpretation of this material, data and/or information.

Figure 2: Modelling predictions generated using systematic approach based on available data. (a) shows habitat suitability scores (the likelihood of observing the target species within each grid cell given variation environmental variables) determined by aggregating outputs from the "best" species distribution model (7 models compared) across 100 simulations. Here, the mid value on the scale denotes the threshold score above which occurrence is assumed. (b) shows: (i) the lower bound (Minimum); and (ii) the upper bound (Maximum); of abundance estimates determined by relating observed density (taking into account potential uncertainty) with habitat suitability scores using linear regression.
