# Supplementary material for: A systematic approach to estimate the distribution and total abundance of British mammals
Source: PLoS One. 2017 Jun 28;12(6):e0176339. doi: 10.1371/journal.pone.0176339 (PMC5489149; doi:10.1371/journal.pone.0176339)
Supplement: S9 File — Individual reports for each of the Rodentia species presenting analysis of the available data and subsequent model predictions based on a 10km raster grid. Reports also include expert comment assessing the reliability (and plausibility) of results in the context of existing evidence and popular opinion. (ZIP) [file pone.0176339.s009.zip › N Water vole.pdf]

Water vole (*Arvicola terrestris*)

Order: *Rodentia*

Genus: *Arvicola*

Origin: Native

Status: Common

1995 abundance estimate: 1,169,000 (3)

Reported population trends: JNCC 2005 (↓)

Data:

The available occurrence records indicate that the water vole is widespread throughout the majority of England with sightings becoming more patchy in areas of Cornwall, Wales and Scotland (Figure 1a). In general sightings are recent, however, the map highlights some areas where records have not been updated for some time.

From the literature review we identified several studies (Aars et al. 2001; Benge 2004; Forman 2005; Telfer et al. 2003) conducted across different geographic areas of the observed distribution between 1998 and 2004 (Figure 1b). Estimates ranged between 0 and 167,686 per km^2 with the highest densities reported in arable dominated land cover (17 - 167,686 per km^2 accounting for uncertainty relating to unsurveyed areas within grid cells). Due to the limited coverage of these surveys estimates were unavailable for several dominant land covers where occurrence was reported (marked grey in Table 1) and where estimates were available the relative uncertainty within cells was very large.

Model predictions:

The habitat suitability map (Figure 2a) appears to reflect the underlying data reasonably well with the set of "best" models predicting presence (and absence) to a mean AUC of 0.72. However, the distribution of predicted occurrence appears to show some contraction along the western edge and in the northern regions of Scotland. Overall, across 100 repetitions MaxEnt proved to be the most commonly selected modelling approach displaying the highest AUC 48% of the time followed by Generalised Linear Models (28%). By land cover the mean habitat suitability scores suggest observation is most likely in landscapes dominated by urban and suburban land cover (Table 1); perhaps indicating the use of man-made drainage channels. However, consistent with recorded sightings, the majority of occurrence is predicted in arable and improved grassland.

Both minimum and maximum density estimates were found to be correlated with habitat suitability, best fitted to a quadratic and linear function respectively and accounting for spherical spatial autocorrelation. Although, interestingly both relationships suggested a negative association predicting the highest densities in cells of lower suitability with the minimum density displaying the more extreme trend (the gaps in distribution are caused by the prediction of negative densities in cells where suitability is high).

Despite this the resulting predicted abundance range contains the estimate from Harris et al. (1995) suggesting no change in the total population. Whilst this disagrees with recently reported trends there is scope within the range to argue that populations have decreased. The range itself is very large due to the uncertainty caused by small survey sites relative to the 10km scale at which modelling is performed. This is a particular issue for modelling species which are best described using linear features with most studies only surveying specialist habitat compromising a small proportion of cells. Standardised practice for density collection and reporting based on a raster grid accounting for all habitat within a given cell would potentially alleviate the problem. Given this specialist nature it is perhaps reasonable to suggest that the true estimate lies towards the lower end of the range where small isolated populations within cells are assumed unrepresentative of the wider landscape. In order to provide more accurate predictions future model analysis could be based on a finer scale raster grid which would better represent the variations in habitat for smaller mammals. Unfortunately, at present this is too unreliable due to access restrictions imposed on occurrence data.

Reliability (Expert comment):

The majority of recorded observations were from arable, horticulture and improved grassland, along with relatively high upper limits for density; this appears to have led to high predicted densities for these two land classes. There

were also a large number of observations from upland habitats including bog and heather grassland, and while observed densities for these were low, upper limits for predicted densities were high. A surprisingly high total abundance of up to 8 billion water voles was predicted, despite there being no recorded observations from freshwater-dominated grid-squares.

References:

Aars, J., X. Lambin, R. Denny and A. C. Griffin (2001). Water vole in the Scottish uplands: distribution patterns of disturbed and pristine populations ahead and behind the American mink invasion front. *Animal Conservation* 4(3): 187-194.

Benge, J. M. (2004). The ecology of the water vole (*Arvicola terrertris*) in Southern England. Ph.D. Thesis, University of Hertfordshire.

Forman, D. W. (2005). An assessment of the local impact of native predators on an established population of British water voles (*Arvicola terrestris*). *Journal of Zoology* 266(3): 221-226.

Harris, S. J., P. Morris, S. Wray and D. Yalden (1995). A review of British mammals: population estimates and conservation status of British mammals other than cetaceans, Joint Nature Conservation Committee, Peterborough, UK.

Telfer, S., J. F. Dallas, J. Aars, S. B. Piertney, W. A. Stewart and X. Lambin (2003). Demographic and genetic structure of fossorial water voles (*Arvicola terrestris*) on Scottish islands. *Journal of Zoology* 259(1): 23-29.

Table 1: Summary of observed data and model predictions by land cover class (LCM2007 target classification). Values shown in brackets denote the spatial coverage based on a 10km resolution raster map (number of grid cells). Years represent the median of records within each land class. Ranges for density and abundance are derived using the respective minimum and maximum raster maps (lower bound is mean of values across minimum raster map with upper across the maximum) which capture the spatial uncertainty generate by projecting irregular polygons describing survey sites onto a raster grid.

LCM2007 class	Observed					Predicted		
	Occurrence		Density					
	Records	Year	Estimates	Year	Range	Habitat suitability	Density	Abundance
1 (Broadleaved woodland)	12 (7)	1973	0 (0)	-	-	0.83 (9)	4.9 - 71,713	4,386 - 64,541,362
2 (Coniferous woodland)	190 (47)	2002	2 (1)	1998	25.9 - 2,613	0.58 (3)	2.7 - 83,877	815.9 - 25,163,007
3 (Arable and Horticultural)	27,786 (790)	2008	1 (1)	-	17 - 167,686	0.89 (867)	3.7 - 49,667	321,362 - 4,306,130,985
4 (Improved grassland)	10,945 (424)	1999	5 (2)	1999	7.4 - 885.6	0.72 (333)	4 - 65,128	131,429 - 2,168,774,703
5 (Rough grassland)	87 (8)	2008	0 (0)	-	-	0.27 (2)	0 - 84,241	-
6 (Neutral grassland)	0 (0)	-	0 (0)	-	-	0 (0)	-	-
7 (Calcareous grassland)	233 (2)	2012	0 (0)	-	-	0.88 (2)	8.3 - 63,887	1,660 - 12,777,497
8 (Acid grassland)	1206 (66)	2002	0 (0)	-	-	0.58 (1)	0 - 96,735	-
9 (Fen, Marsh, and Swamp)	0 (0)	-	0 (0)	-	-	-	-	-
10 (Heather)	294 (30)	2005	0 (0)	-	-	0.69 (20)	2.1 - 87,038	4,137 - 174,076,447
11 (Heather grassland)	846 (45)	2007	7 (6)	1999	0.01 - 1.9	0.46 (14)	3.1 - 83,342	4,372 - 116,678,253
12 (Bog)	2810 (56)	2008	1 (1)	1999	0.03 - 2.75	0.45 (55)	4.1 - 77,377	22,453 - 425,575,210
13 (Montane habitat)	1207 (41)	2009	11 (7)	1999	0.02 - 2.75	0.84 (43)	5.4 - 70,548	23,121 - 303,354,276
14 (Inland rock)	0 (0)	-	0 (0)	-	-	0.06 (0)	-	-
15 (Saltwater)	22 (2)	1988	0 (0)	-	-	0.53 (0)	-	-
16 (Freshwater)	0 (0)	-	0 (0)	-	-	0.36 (0)	-	-
17 (Supra-littoral rock)	0 (0)	-	0 (0)	-	-	0.03 (0)	-	-
18 (Supra-littoral sediment)	28 (2)	1982	0 (0)	-	-	0.48 (1)	0 - 28722	-
19 (Littoral rock)	0 (0)	-	0 (0)	-	-	0.24 (1)	0 - 307.6	-
20 (Littoral sediment)	140 (19)	1997	0 (0)	-	-	0.74 (16)	2.9 - 34300	4,609 - 54,879,995
21 (Saltmarsh)	0 (0)	-	0 (0)	-	-	-	-	-
22 (Urban)	115 (6)	1988	0 (0)	-	-	0.9 (7)	4.2 - 36,861	2,905 - 25,802,601
23 (Suburban)	1,829 (64)	2006	2 (2)	-	1.4 - 15,160	0.91 (72)	3.2 - 40,099	23,192 - 288,713,831
Total	47,750 (1,609)	2005	29 (20)	1999	3.2 - 9,681	0.72 (1446)	3.8 - 55,297	544,441 - 7,995,892,846

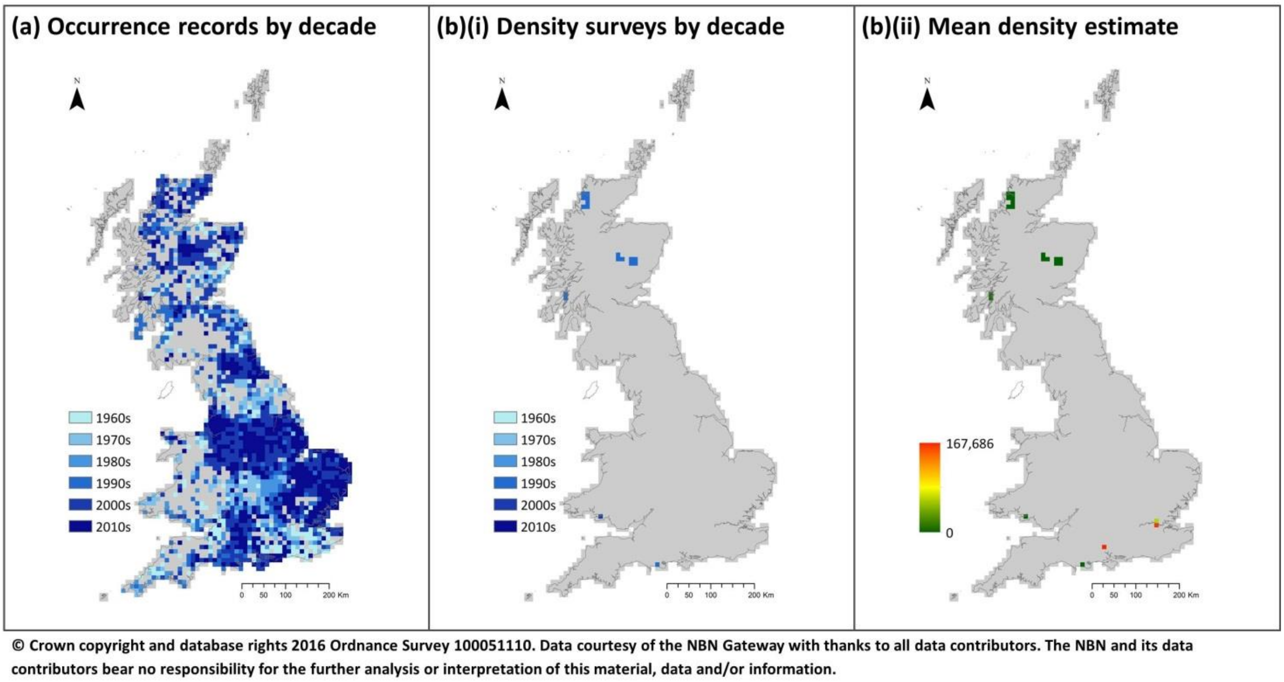

Figure 1: 10km resolution raster maps based on BNG presenting the geographic description of available data. (a) shows the distribution of species occurrence obtained via the NBN Gateway categorised by the decade of last sighting. (b) shows information relating to density surveys identified via a search of published literature where: (i) categorises surveys by the decade of last survey; and (ii) shows the mean density estimate of surveys within grid cells (estimates assumed to be representative of entire cell, considered the upper limit of observed density).

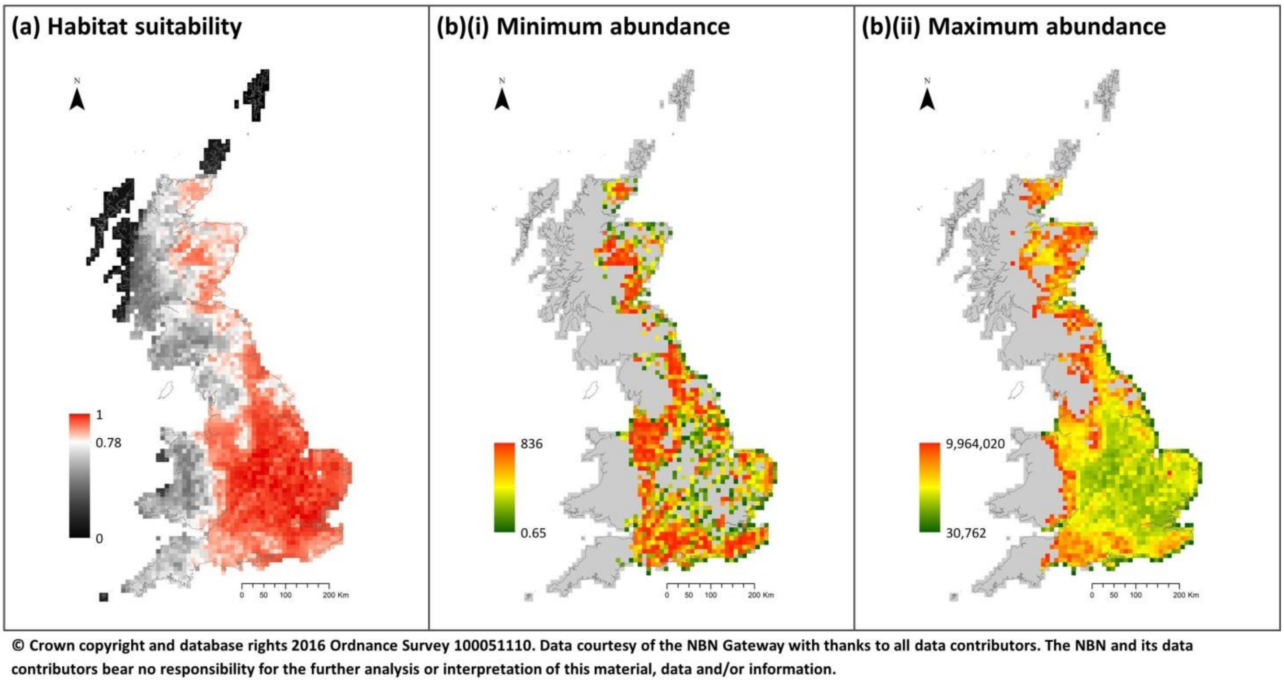

Figure 2: Modelling predictions generated using systematic approach based on available data. (a) shows habitat suitability scores (the likelihood of observing the target species within each grid cell given variation environmental variables) determined by aggregating outputs from the "best" species distribution model (7 models compared) across 100 simulations. Here, the mid value on the scale denotes the threshold score above which occurrence is assumed. (b) shows: (i) the lower bound (Minimum); and (ii) the upper bound (Maximum); of abundance estimates determined by relating observed density (taking into account potential uncertainty) with habitat suitability scores using linear regression.
