# Supplementary material for: A systematic approach to estimate the distribution and total abundance of British mammals
Source: PLoS One. 2017 Jun 28;12(6):e0176339. doi: 10.1371/journal.pone.0176339 (PMC5489149; doi:10.1371/journal.pone.0176339)
Supplement: S9 File — Individual reports for each of the Rodentia species presenting analysis of the available data and subsequent model predictions based on a 10km raster grid. Reports also include expert comment assessing the reliability (and plausibility) of results in the context of existing evidence and popular opinion. (ZIP) [file pone.0176339.s009.zip › O Wood mouse.pdf]

Wood mouse (*Apodemus sylvaticus*)

Order: *Rodentia* | **Genus:** *Apodemus*

Origin: Native | **Status:** Common

1995 abundance estimate: 38,000,000 (3) | **Reported population trends:** None

Data:

The available occurrence records indicate that the wood mouse is widespread throughout GB (Figure 1a). However, the map highlights several areas, particularly in Wales, Scotland and the south east of England where the species has not been recorded for some time, or not at all.

Density estimates, primarily recorded over the past two decades, were obtained from published literature (Baker et al. 2005; Bush et al. 2012; Flowerdew & Ellwood 2001; Flowerdew et al. 2004; Forman 2005; Gelling et al. 2007; Kotzageorgis & Mason 1997; Mallorie & Flowerdew 1994; Shore et al. 2005; Tattersall et al. 2002; Telfer et al. 2007). These estimates were reported in various habitats and were well spread across the distribution of observed occurrence (Figure 1b). However, due to the limited coverage of these surveys estimates were unavailable for several dominant land covers where occurrence was reported (marked grey in Table 1) and where estimates were available the relative uncertainty within cells was large.

Model predictions:

The habitat suitability map (Figure 2a) appears to reflect the underlying data well with the set of "best" models predicting presence (and absence) to a mean AUC of 0.72. Overall, across 100 repetitions MaxEnt proved to be the most commonly selected modelling approach displaying the highest AUC 48% of the time followed by Random Forest (28%). By land cover the mean habitat suitability scores suggest observation is most likely in landscapes dominated by urban and broadleaved woodland (Table 1) but, consistent with recorded sightings, the majority of occurrence is predicted in arable and improved grassland (the most common dominant land covers at a 10km scale).

Both minimum and maximum density estimates were best fitted to the square of habitat suitability accounting for spherical spatial autocorrelation.

The resulting predicted abundance range contains the estimate from Harris et al. (1995) suggests no change in total population (this is unsurprising given the similar temporal range of density surveys used to produce both predictions; the result indicates that any changes to the distribution over the past 20 years have not been significant). However, this range is very large due to the uncertainty caused by small survey sites relative to the 10km scale at which modelling is performed. In order to provide more accurate predictions future model analysis could be based on a finer scale raster grid which would better represent the variations in habitat for smaller mammals. Unfortunately, at present this is too unreliable due to access restrictions imposed on occurrence data.

Reliability (Expert comment):

The distribution of recorded sightings does not appear to have changed greatly over the last 20 years and is similar to that reported by Arnold (1993), except for an apparent increase in East Anglia. Although wood mice forage in cereal crops during summer, their abundance in arable, horticultural and improved grassland is probably dependent on the presence of hedgerows and woodland edges; the upper limits for abundance predictions may be too high if they assume an unrealistically high proportion of favourable habitats within less-favourable dominant land classes. Wood mice frequently enter houses and outbuildings in autumn and winter looking for food and harbourage, and the estimates for suburban habitats appear reasonable. No published density estimates were found for 13 of the 23 land classes and apart from broadleaved woodland and urban habitats these were predicted to make a relatively small contribution to total abundance. These predictions seem reasonable, given the known habitat preferences for the species. However, populations in broadleaved woodland may be even higher than predicted, peak densities of 225 wood mice per hectare (22,500 per km^2) have been reported for oak woodland in Devon (Gurnell 1978).

References:

Arnold, H. R. (1993). Atlas of mammals in Britain: HMSO.

Baker, P. J., A. J. Bentley, R. J. Ansell and S. Harris (2005). Impact of predation by domestic cats *Felis catus* in an urban area. *Mammal Review* 35(3-4): 302-312.

Bush, E. R., C. D. Buesching, E. M. Slade and D. W. Macdonald (2012). Woodland recovery after suppression of deer: cascade effects for small mammals, wood mice (*Apodemus sylvaticus*) and bank voles (*Myodes glareolus*). *PLOS ONE* 7(2): e31404.

Flowerdew, J. R. and S. A. Ellwood (2001). Impacts of woodland deer on small mammal ecology. *Forestry* 74(3): 277-287.

Flowerdew, J. R., R. F. Shore, S. M. C. Poulton and T. H. Sparks (2004). Live trapping to monitor small mammals in Britain. *Mammal Review* 34(1-2): 31-50.

Forman, D. W. (2005). An assessment of the local impact of native predators on an established population of British water voles (*Arvicola terrestris*). *Journal of Zoology* 266(3): 221-226.

Gelling, M., D. W. Macdonald and F. Mathews (2007). Are hedgerows the route to increased farmland small mammal density? Use of hedgerows in British pastoral habitats. *Landscape Ecology* 22(7): 1019-1032.

Gurnell, J. (1978). Seasonal changes in numbers and male behavioural interaction in a population of wood mice, *Apodemus sylvaticus*. *Journal of Animal Ecology* 47(3): 741-755.

Harris, S. J., P. Morris, S. Wray and D. Yalden (1995). A review of British mammals: population estimates and conservation status of British mammals other than cetaceans, Joint Nature Conservation Committee, Peterborough, UK.

Kotzageorgis, G. C. and C. F. Mason (1997). Small mammal populations in relation to hedgerow structure in an arable landscape. *Journal of Zoology* 242(3): 425-434.

Mallorie, H. C. and J. R. Flowerdew (1994). Woodland small mammal population ecology in Britain: a preliminary review of the Mammal Society survey of Wood Mice *Apodemus sylvaticus* and Bank Voles *Clethrionomys glareolus*, 1982–87. *Mammal Review* 24(1): 1-15.

Shore, R. F., W. R. Meek, T. H. Sparks, R. F. Pywell and M. Nowakowski (2005). Will environmental stewardship enhance small mammal abundance on intensively managed farmland? *Mammal Review* 35(3-4): 277-284.

Tattersall, F. H., D. W. Macdonald, B. J. Hart, P. Johnson, W. Manley and R. Feber (2002). Is habitat linearity important for small mammal communities on farmland? *Journal of Applied Ecology* 39(4): 643-652.

Telfer, S., H. E. Clough, R. J. Birtles, M. Bennett, D. Carslake, S. Helyar and M. Begon (2007). Ecological differences and coexistence in a guild of micro-parasites: *Bartonella* in wild rodents. *Ecology* 88(7): 1841-1849.

Table 1: Summary of observed data and model predictions by land cover class (LCM2007 target classification). Values shown in brackets denote the spatial coverage based on a 10km resolution raster map (number of grid cells). Years represent the median of records within each land class. Ranges for density and abundance are derived using the respective minimum and maximum raster maps (lower bound is mean of values across minimum raster map with upper across the maximum) which capture the spatial uncertainty generate by projecting irregular polygons describing survey sites onto a raster grid.

LCM2007 class	Observed					Predicted		
	Occurrence		Density					
	Records	Year	Estimates	Year	Range	Habitat suitability	Density	Abundance
1 (Broadleaved woodland)	129 (11)	2005	0 (0)	-	-	0.91 (11)	13.4 - 3,032	14,755 - 3,335,492
2 (Coniferous woodland)	414 (107)	1994	1 (1)	1987	0.14 - 864.2	0.73 (92)	2.1 - 988	18,860 - 9,091,660
3 (Arable and Horticultural)	9,017 (781)	2005	22 (16)	1994	2.1 - 1,357	0.85 (934)	5 - 1,234	466,573 - 115,235,298
4 (Improved grassland)	4,272 (551)	2002	20 (17)	2003	1.4 - 883.2	0.78 (573)	3.6 - 1,079	205,450 - 61,826,382
5 (Rough grassland)	62 (25)	1978	1 (1)	1994	0.04 - 247	0.46 (5)	1.2 - 824	582.2 - 411,746
6 (Neutral grassland)	2 (1)	1968	0 (0)	-	-	0.35 (0)	-	-
7 (Calcareous grassland)	19 (2)	2013	0 (0)	-	-	0.88 (2)	5.5 - 1,174	1,095 - 234,986
8 (Acid grassland)	243 (76)	1996	0 (0)	-	-	0.48 (10)	2.4 - 1,550	2,346 - 1,549,562
9 (Fen, Marsh, and Swamp)	0 (0)	-	0 (0)	-	-	-	-	-
10 (Heather)	69 (32)	1994	0 (0)	-	-	0.61 (16)	1.4 - 1,005	2,272 - 1,607,664
11 (Heather grassland)	309 (67)	1999	0 (0)	-	-	0.55 (15)	2.4 - 1,450	3,662 - 2,174,619
12 (Bog)	130 (52)	1986	0 (0)	-	-	0.42 (10)	2.5 - 1,104	2,498 - 1,104,380
13 (Montane habitat)	41 (10)	2004	0 (0)	-	-	0.26 (0)	-	-
14 (Inland rock)	2 (1)	1977	0 (0)	-	-	0.5 (0)	-	-
15 (Saltwater)	75 (8)	2010	0 (0)	-	-	0.75 (7)	0.3 - 225	173.7 - 157,552
16 (Freshwater)	42 (3)	2010	1 (0)	1986	0	0.76 (2)	4.6 - 1,265	910 - 252,972
17 (Supra-littoral rock)	0 (0)	-	0 (0)	-	-	0.07 (0)	-	-
18 (Supra-littoral sediment)	9 (3)	1981	0 (0)	-	-	0.6 (1)	0.9 - 566	92.7 - 56,573
19 (Littoral rock)	0 (0)	-	0 (0)	-	-	0.37 (1)	0.01 - 4.78	0.69 - 478
20 (Littoral sediment)	252 (25)	1998	0 (0)	-	-	0.77 (25)	0.8 - 360	2,107 - 899,355
21 (Saltmarsh)	0 (0)	-	0 (0)	-	-	-	-	-
22 (Urban)	37 (8)	2011	0 (0)	-	-	0.92 (8)	17.7 - 4,195	14,153 - 3,356,037
23 (Suburban)	1,147 (68)	2011	2 (2)	2003	55.8 - 1,039	0.89 (78)	9.4 - 2,145	73,588 - 16,731,433
Total	16,271 (1,831)	2002	47 (37)	1995	4.4 - 1,050	0.73 (1,790)	4.5 - 1,218	809,118 - 218,026,188

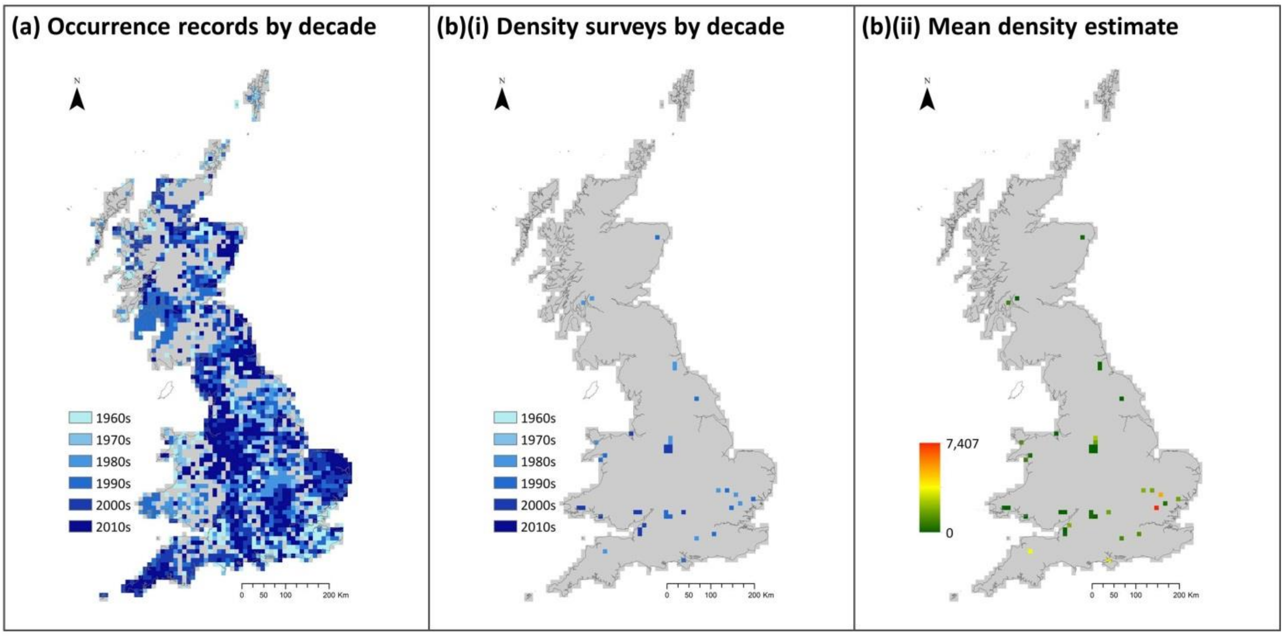

© Crown copyright and database rights 2016 Ordnance Survey 100051110. Data courtesy of the NBN Gateway with thanks to all data contributors. The NBN and its data contributors bear no responsibility for the further analysis or interpretation of this material, data and/or information.

Figure 1: 10km resolution raster maps based on BNG presenting the geographic description of available data. (a) shows the distribution of species occurrence obtained via the NBN Gateway categorised by the decade of last sighting. (b) shows information relating to density surveys identified via a search of published literature where: (i) categorises surveys by the decade of last survey; and (ii) shows the mean density estimate of surveys within grid cells (estimates assumed to be representative of entire cell, considered the upper limit of observed density).

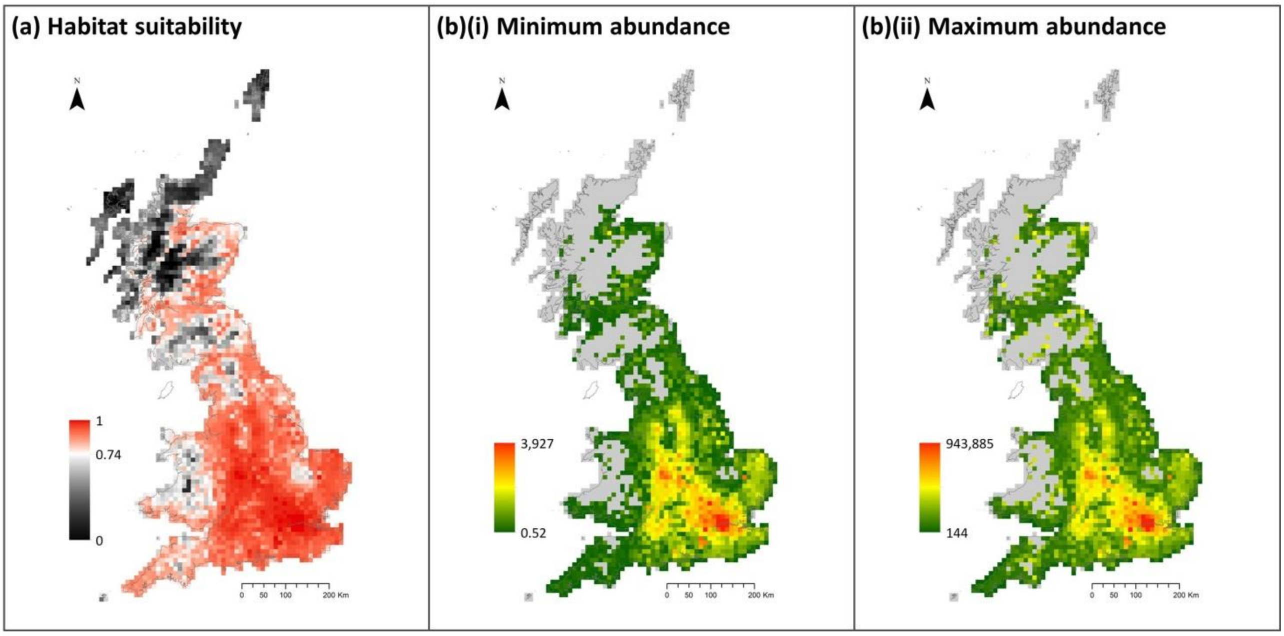

© Crown copyright and database rights 2016 Ordnance Survey 100051110. Data courtesy of the NBN Gateway with thanks to all data contributors. The NBN and its data contributors bear no responsibility for the further analysis or interpretation of this material, data and/or information.

Figure 2: Modelling predictions generated using systematic approach based on available data. (a) shows habitat suitability scores (the likelihood of observing the target species within each grid cell given variation environmental variables) determined by aggregating outputs from the "best" species distribution model (7 models compared) across 100 simulations. Here, the mid value on the scale denotes the threshold score above which occurrence is assumed. (b) shows: (i) the lower bound (Minimum); and (ii) the upper bound (Maximum); of abundance estimates determined by relating observed density (taking into account potential uncertainty) with habitat suitability scores using linear regression.
