# Supplementary material for: A systematic approach to estimate the distribution and total abundance of British mammals
Source: PLoS One. 2017 Jun 28;12(6):e0176339. doi: 10.1371/journal.pone.0176339 (PMC5489149; doi:10.1371/journal.pone.0176339)
Supplement: S9 File — Individual reports for each of the Rodentia species presenting analysis of the available data and subsequent model predictions based on a 10km raster grid. Reports also include expert comment assessing the reliability (and plausibility) of results in the context of existing evidence and popular opinion. (ZIP) [file pone.0176339.s009.zip › P Yellow-necked mouse.pdf]

Yellow-necked mouse (*Apodemus flavicollis*)

Order: *Rodentia*

Genus: *Apodemus*

Origin: Native

Status: Locally common

1995 abundance estimate: 750,000 (4)

Reported population trends: None

Data:

The available occurrence records indicate that the yellow-necked mouse is most widespread in the south of England and Wales with no sightings recorded in northern counties or Scotland (Figure 1a). However, the map highlights several areas where occurrence has not been reported for some time. Sightings were predominantly recorded in habitat dominated by arable and improved grassland.

From the literature review we identified several studies (Gelling et al. 2007; Kotzageorgis & Mason 1997; Tattersall et al. 2002) reporting density which were conducted on farmland between 1990 and 2003 across southern parts of England and Wales (Figure 1b). Estimates ranged between 3.25 and 47.27 per km^2 with the highest reported in habitat dominated by arable land cover (1.22 - 22.31 per km^2 accounting for uncertainty relating to unsurveyed areas within grid cells). Due to the limited coverage of these surveys estimates were unavailable for several dominant land covers where occurrence was reported (marked grey in Table 1).

Model predictions:

The habitat suitability map (Figure 2a) appears to reflect the underlying data reasonably well with the set of "best" models predicting presence (and absence) to a mean AUC of 0.67. However, the resulting distribution is noticeably larger than the area described by the observations alone (approximately 1.7 times). Overall, across 100 repetitions MaxEnt proved to be the most commonly selected modelling approach displaying the highest AUC 27% of the time followed by Random Forest (23%). By land cover the mean habitat suitability scores suggest observation is most likely in landscapes dominated by broadleaved woodland (Table 1) but, consistent with recorded sightings, the majority of occurrence is predicted in arable habitat (the most common dominant land covers at a 10km scale).

Most likely due to the limited number of density estimates linear regression suggested no correlation with habitat suitability. Instead, a constant mean estimate was applied across all cells where occurrence was predicted and summed to derive total abundance.

The predicted abundance range does not contain the estimate from Harris et al. (1995), instead suggesting a significant decrease in the total population. In the absence of recent trend analysis the most likely explanation for this underestimation, given that the predicted distribution shows no evidence of a range contraction, is that the assumed density estimates are relatively low compared with the mean or that the population was perhaps overestimated by Harris et al. (1995); the reliability of the estimate was consider relative low (score of 4 where 5 was the lowest). In order to fully explore these possibilities and to produce more accurate model predictions additional density estimates are required. Model analysis could also be based on a finer scale raster grid which would better represent the variations in habitat for smaller mammals. Unfortunately, at present this is too unreliable due to restrictions imposed on occurrence data.

Reliability (Expert comment):

The overall range for the species is largely unchanged from that reported by Arnold (1993), although it does appear from the observations recorded that over the last 20 years that the species is becoming more patchily distributed towards the southern limits of its range in Britain. This trend is potentially reflected in the abundance estimate reported here which, even at the upper bound is nearly 160,000 lower than that reported by Harris et al. (1995), although differences in the methods used for deriving these two estimates may limit comparability.

References:

Arnold, H. R. (1993). Atlas of mammals in Britain: HMSO.

Gelling, M., D. W. Macdonald and F. Mathews (2007). Are hedgerows the route to increased farmland small mammal density? Use of hedgerows in British pastoral habitats. *Landscape Ecology* 22(7): 1019-1032.

Harris, S. J., P. Morris, S. Wray and D. Yalden (1995). A review of British mammals: population estimates and conservation status of British mammals other than cetaceans, Joint Nature Conservation Committee, Peterborough, UK.

Kotzageorgis, G. C. and C. F. Mason (1997). Small mammal populations in relation to hedgerow structure in an arable landscape. *Journal of Zoology* 242(3): 425-434.

Tattersall, F. H., D. W. Macdonald, B. J. Hart, P. Johnson, W. Manley and R. Feber (2002). Is habitat linearity important for small mammal communities on farmland? *Journal of Applied Ecology* 39(4): 643-652.

Table 1: Summary of observed data and model predictions by land cover class (LCM2007 target classification). Values shown in brackets denote the spatial coverage based on a 10km resolution raster map (number of grid cells). Years represent the median of records within each land class. Ranges for density and abundance are derived using the respective minimum and maximum raster maps (lower bound is mean of values across minimum raster map with upper across the maximum) which capture the spatial uncertainty generate by projecting irregular polygons describing survey sites onto a raster grid.

LCM2007 class	Observed					Predicted		
	Occurrence		Density					
	Records	Year	Estimates	Year	Range	Habitat suitability	Density	Abundance
1 (Broadleaved woodland)	15 (7)	1971	0 (0)	-	-	0.77 (9)	1.02 - 10.79	916.3 - 9,709
2 (Coniferous woodland)	1 (1)	1970	0 (0)	-	-	0.25 (2)	1.02 - 10.79	203.6 - 2,158
3 (Arable and Horticultural)	1,070 (195)	1993	7 (5)	1997	1.22 - 22.31	0.62 (384)	0.98 - 10.43	37,801 - 400,545
4 (Improved grassland)	222 (96)	1985	2 (1)	1996	0.02 - 4.71	0.47 (159)	0.99 - 10.44	15,663 - 165,970
5 (Rough grassland)	0 (0)	-	0 (0)	-	-	0.19 (0)	-	-
6 (Neutral grassland)	0 (0)	-	0 (0)	-	-	0.09 (0)	-	-
7 (Calcareous grassland)	0 (0)	-	0 (0)	-	-	0.68 (0)	-	-
8 (Acid grassland)	8 (4)	1996	0 (0)	-	-	0.26 (0)	-	-
9 (Fen, Marsh, and Swamp)	0 (0)	-	0 (0)	-	-	-	-	-
10 (Heather)	0 (0)	-	0 (0)	-	-	0.24 (0)	-	-
11 (Heather grassland)	0 (0)	-	0 (0)	-	-	0.19 (0)	-	-
12 (Bog)	0 (0)	-	0 (0)	-	-	0.19 (0)	-	-
13 (Montane habitat)	0 (0)	-	0 (0)	-	-	0.22 (0)	-	-
14 (Inland rock)	0 (0)	-	0 (0)	-	-	0.18 (0)	-	-
15 (Saltwater)	0 (0)	-	0 (0)	-	-	0.39 (0)	-	-
16 (Freshwater)	0 (0)	-	0 (0)	-	-	0.2 (0)	-	-
17 (Supra-littoral rock)	0 (0)	-	0 (0)	-	-	0.17 (0)	-	-
18 (Supra-littoral sediment)	0 (0)	-	0 (0)	-	-	0.28 (0)	-	-
19 (Littoral rock)	0 (0)	-	0 (0)	-	-	0.24 (0)	-	-
20 (Littoral sediment)	0 (0)	-	0 (0)	-	-	0.37 (0)	-	-
21 (Saltmarsh)	0 (0)	-	0 (0)	-	-	-	-	-
22 (Urban)	2 (1)	1990	0 (0)	-	-	0.43 (0)	-	-
23 (Suburban)	30 (16)	1976	0 (0)	-	-	0.53 (13)	0.99 - 10.5	1,288 - 13,651
Total	1,348 (320)	1988	9 (6)	1996	1.02 - 19.38	0.44 (567)	0.99 - 10.44	55,873 - 592,033

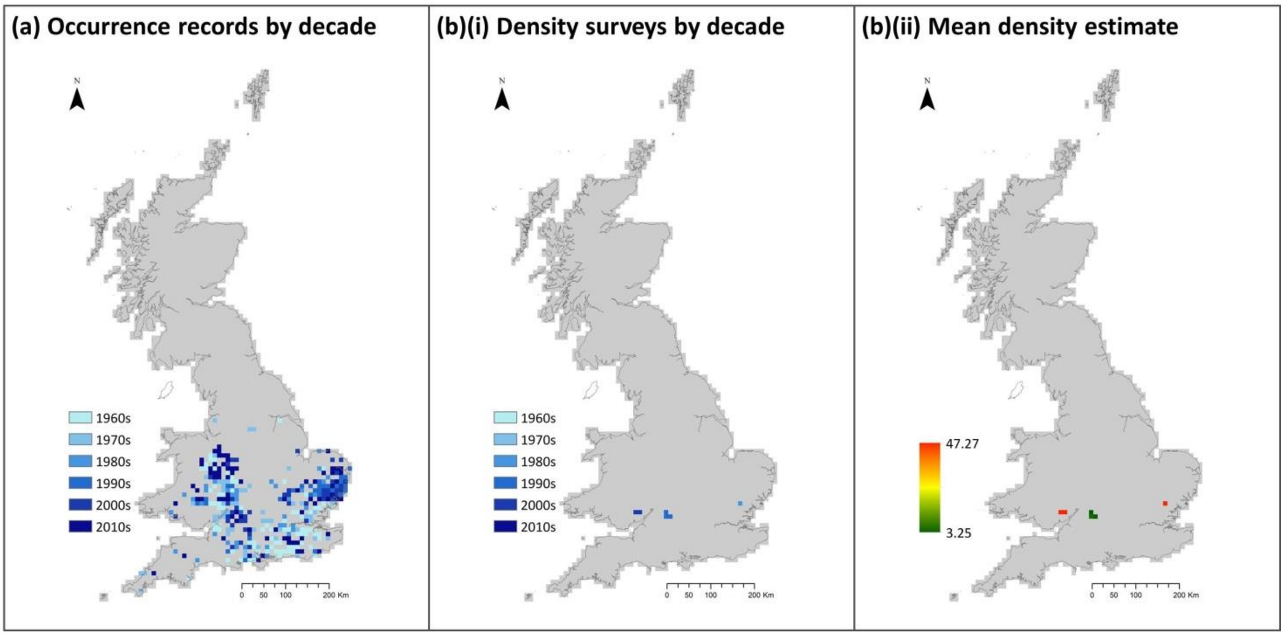

© Crown copyright and database rights 2016 Ordnance Survey 100051110. Data courtesy of the NBN Gateway with thanks to all data contributors. The NBN and its data contributors bear no responsibility for the further analysis or interpretation of this material, data and/or information.

Figure 1: 10km resolution raster maps based on BNG presenting the geographic description of available data. (a) shows the distribution of species occurrence obtained via the NBN Gateway categorised by the decade of last sighting. (b) shows information relating to density surveys identified via a search of published literature where: (i) categorises surveys by the decade of last survey; and (ii) shows the mean density estimate of surveys within grid cells (estimates assumed to be representative of entire cell, considered the upper limit of observed density).

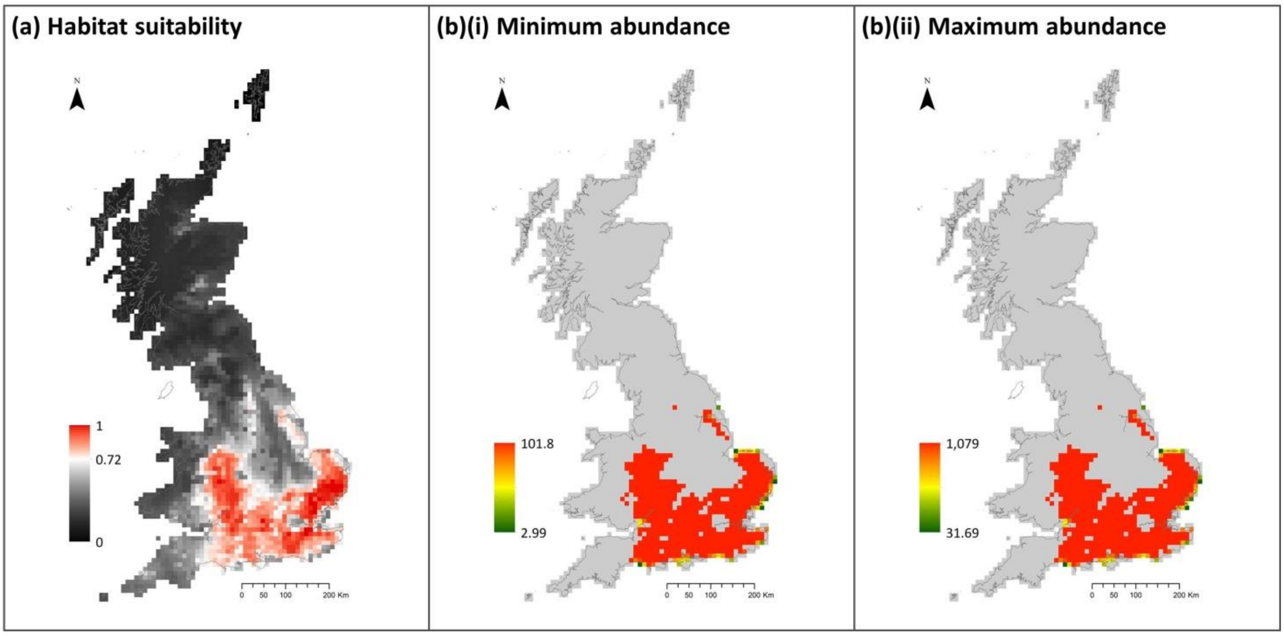

© Crown copyright and database rights 2016 Ordnance Survey 100051110. Data courtesy of the NBN Gateway with thanks to all data contributors. The NBN and its data contributors bear no responsibility for the further analysis or interpretation of this material, data and/or information.

Figure 2: Modelling predictions generated using systematic approach based on available data. (a) shows habitat suitability scores (the likelihood of observing the target species within each grid cell given variation environmental variables) determined by aggregating outputs from the "best" species distribution model (7 models compared) across 100 simulations. Here, the mid value on the scale denotes the threshold score above which occurrence is assumed. (b) shows: (i) the lower bound (Minimum); and (ii) the upper bound (Maximum); of abundance estimates determined by relating observed density (taking into account potential uncertainty) with habitat suitability scores using linear regression.
